# Early estimates of the indirect effects of the COVID-19 pandemic on maternal and child mortality in low-income and middle-income countries: a modelling study

**DOI:** 10.1016/S2214-109X(20)30229-1

**Published:** 2020-05-12

**Authors:** Timothy Roberton, Emily D Carter, Victoria B Chou, Angela R Stegmuller, Bianca D Jackson, Yvonne Tam, Talata Sawadogo-Lewis, Neff Walker

**Affiliations:** a Johns Hopkins Bloomberg School of Public Health, Johns Hopkins University, Baltimore, MD, USA

## Abstract

**Background:**

While the COVID-19 pandemic will increase mortality due to the virus, it is also likely to increase mortality indirectly. In this study, we estimate the additional maternal and under-5 child deaths resulting from the potential disruption of health systems and decreased access to food.

**Methods:**

We modelled three scenarios in which the coverage of essential maternal and child health interventions is reduced by 9·8–51·9% and the prevalence of wasting is increased by 10–50%. Although our scenarios are hypothetical, we sought to reflect real-world possibilities, given emerging reports of the supply-side and demand-side effects of the pandemic. We used the Lives Saved Tool to estimate the additional maternal and under-5 child deaths under each scenario, in 118 low-income and middle-income countries. We estimated additional deaths for a single month and extrapolated for 3 months, 6 months, and 12 months.

**Findings:**

Our least severe scenario (coverage reductions of 9·8–18·5% and wasting increase of 10%) over 6 months would result in 253 500 additional child deaths and 12 200 additional maternal deaths. Our most severe scenario (coverage reductions of 39·3–51·9% and wasting increase of 50%) over 6 months would result in 1 157 000 additional child deaths and 56 700 additional maternal deaths. These additional deaths would represent an increase of 9·8–44·7% in under-5 child deaths per month, and an 8·3–38·6% increase in maternal deaths per month, across the 118 countries. Across our three scenarios, the reduced coverage of four childbirth interventions (parenteral administration of uterotonics, antibiotics, and anticonvulsants, and clean birth environments) would account for approximately 60% of additional maternal deaths. The increase in wasting prevalence would account for 18–23% of additional child deaths and reduced coverage of antibiotics for pneumonia and neonatal sepsis and of oral rehydration solution for diarrhoea would together account for around 41% of additional child deaths.

**Interpretation:**

Our estimates are based on tentative assumptions and represent a wide range of outcomes. Nonetheless, they show that, if routine health care is disrupted and access to food is decreased (as a result of unavoidable shocks, health system collapse, or intentional choices made in responding to the pandemic), the increase in child and maternal deaths will be devastating. We hope these numbers add context as policy makers establish guidelines and allocate resources in the days and months to come.

**Funding:**

Bill & Melinda Gates Foundation, Global Affairs Canada.

## Introduction

The international community is mobilising to limit the spread of severe acute respiratory syndrome coronavirus 2 and reduce mortality from COVID-19. As of May 1, 2020, more than 237 000 people have died from COVID-19, and estimates of future deaths number in the millions.^1^, ^2^ Governments are responding at local, national, regional, and global levels, and health officials are developing guidance for health systems and the public.^3^ In weighing their options, policy makers must consider not only the immediate health effects of the pandemic but also the indirect effects of the pandemic and the response to it. An analysis of the 2014 outbreak of Ebola virus in west Africa showed that the indirect effects of the outbreak were more severe than the outbreak itself.^4^ Although mortality rates for COVID-19 appear to be low in children and in women of reproductive age,^5^ these groups might be disproportionately affected by the disruption of routine health services, particularly in low-income and middle-income countries (LMICs). With this in mind, we sought to quantify the potential indirect effects of the COVID-19 pandemic on maternal and child mortality.

In past epidemics, health systems have struggled to maintain routine services and utilisation of services has decreased.^6^ As WHO notes, “People, efforts, and medical supplies all shift to respond to the emergency. This often leads to the neglect of basic and regular essential health services. People with health problems unrelated to the epidemic find it harder to get access to health care services.”^7^ A study of the 2014 epidemic of Ebola virus disease estimated that, during the outbreak, antenatal care coverage decreased by 22 percentage points, and there were declines in the coverage of family planning (6 percentage points), facility delivery (8 percentage points), and postnatal care (13 percentage points).^8^ Qualitative studies suggest that these reductions were due to fear of contracting Ebola virus at health facilities, distrust of the health system, and rumours about the source of the disease.^9^ During the 2003 severe acute respiratory syndrome epidemic, ambulatory care decreased by 23·9% in Taiwan and inpatient care decreased by 35·2%.^10^ Simulated models of influenza pandemics also predict reductions in utilisation of health services.^11^

**Research in context**

**Evidence before this study**

The global community is responding in unprecedented ways to limit the spread of severe acute respiratory syndrome coronavirus 2 and reduce mortality from COVID-19. Global organisations have called for maintaining routine health services during the pandemic; however, the potential indirect effects on mortality from maternal and child health service disruption have not been quantified. Previous infectious disease outbreaks indirectly resulted in increases in mortality caused by reductions in the provision and use of routine health services. Notably, the 2014 Ebola virus epidemic resulted in a 27·6 percentage point decrease in service use and 44·3 percentage point decrease in inpatient services in high-incidence areas of west Africa. During the 2003 epidemic of severe acute respiratory syndrome, a 23·9% reduction in ambulatory care and a 35·2% reduction in inpatient care was observed in Taiwan. Similar indirect effects are plausible as a result of the COVID-19 pandemic and control efforts.

**Added value of this study**

We quantified the potential indirect effect of the COVID-19 pandemic and control efforts on reproductive, maternal, newborn, and child health in 118 low-income and middle-income countries (LMICs) using the Lives Saved Tool. We modelled the effects on maternal and under-5 mortality of three outbreak scenarios and attributed the excess mortality to reductions in specific interventions or increases in risk factors. Our analysis shows that, if the COVID-19 pandemic results in widespread disruption to health systems, LMICs can expect to see substantial increases in maternal and child deaths. Childbirth care and child curative services are particularly vulnerable to disruption and would account for the greatest number of additional maternal and child deaths.

**Implications of all the available evidence**

Our analysis does not aim to predict the trajectory of the pandemic response in LMICs. Quantification of the indirect effects of the pandemic is intended to serve as a benchmark for policy makers. The choices that governments make in responding to the pandemic will have consequences for the health and livelihoods of populations. In the context of these choices, our estimates highlight the need to consider how to mitigate the effect of health system disruptions and movement restrictions on maternal and child health. Our analysis provides a framework that policy makers can use to prioritise interventions and quantify the secondary effects of resource allocation and control measures, to inform decisions around health system continuity and stop-gap measures during and following the pandemic.

Already with COVID-19 we are seeing similar disruptions. The pandemic and the response to the pandemic are affecting both the provision and utilisation of reproductive, maternal, newborn, and child health (RMNCH) services.

Amid the pandemic, health workers, equipment, and facilities have been reassigned to address the influx of patients with COVID-19.^12^ Restructuring of the health system could result in the closure of peripheral health facilities, as seen in the 2014 Ebola virus outbreak.^13^ The health workforce has been further reduced by nosocomial COVID-19 infection and burnout.^14^ RMNCH interventions delivered through campaigns (eg, vaccinations, bednets, or vitamin A) are being paused or reduced in scale.^15^ COVID-19 has also disrupted the global pharmaceutical and medical supply chain. The low buying power of LMICs and their lack of infrastructure for domestic production are disadvantageous in ensuring a steady supply chain. Global reserves and international procurement mechanisms for essential RMNCH medicines could mitigate shortages;^16^ however, interruptions in global transport could affect these channels. In addition, local efforts to contain COVID-19 are likely to negatively impact domestic medical supply chains.

Governments are restricting population movement by closing borders, reducing public transport, halting non-essential activities, and issuing shelter-in-place orders. These restrictions are negatively affecting economies. Lost income, increased prices, and overburdened social safety nets will push vulnerable groups further into poverty and increase financial and other barriers to health-care access. Movement restrictions will reduce physical access, exacerbated by reduced transport availability and the real or perceived threat of prosecution for travelling in public spaces.^17^ Demand for RMNCH services might decline as concerns over COVID-19 transmission alter the perceived risk–benefit calculation for individuals deciding to seek care. In many settings, the broader socioeconomic effects of the pandemic will exacerbate food insecurity. Increased poverty, and disrupted food and agriculture systems, will increase reliance on staple foods and restrict access to diverse and nutritious diets.

Given these developments, it seems reasonable to expect disruption to maternal and child health services and increased undernutrition in the coming months. In many LMICs, maternal and child mortality remains high, and hard-won gains could falter without continued attention. Practitioners are already voicing such concerns.^18^ WHO released operational guidance for maintaining essential health services and adapting service delivery platforms to avoid interruptions.^3^

Statistical modelling can help to inform policy decisions related to the pandemic. Models have already been used to estimate the direct effects of COVID-19, including on pregnant women and infants.^19^ In this study, we add to existing models by estimating the indirect effects of the pandemic on maternal and child mortality in LMICs. While it is still early in the pandemic, a set of realistic, quantifiable estimates will provide a reference point for decision makers currently weighing response strategies.

## Methods

### Overview

**Figure 1:**
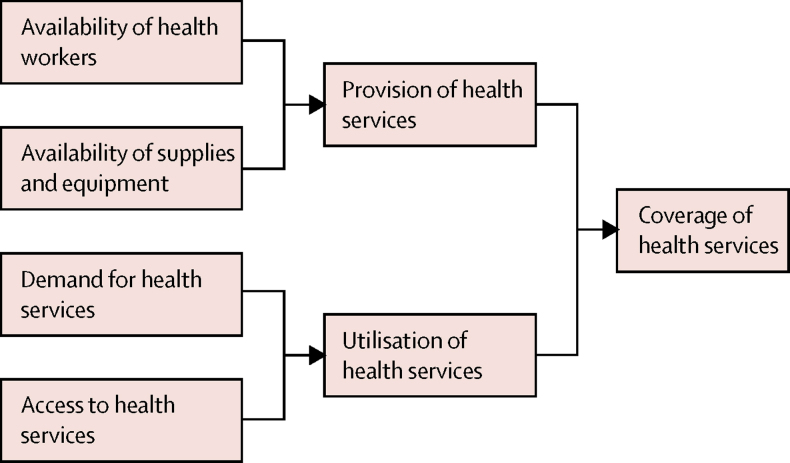
Framework for the effects of health system components on coverage of health services

We used the Lives Saved Tool (LiST) to estimate additional deaths due to reduced coverage of interventions, and increased prevalence of wasting, under three scenarios and for three time periods. To develop our assumptions, we adopted a simple health systems framework (figure 1). The framework assumes that four health system components affect coverage of services: availability of health workers, availability of supplies and equipment, demand for services, and access to services. In our analysis, coverage assumes that an intervention is delivered with sufficient quality to achieve its intended health effect.^20^ In our framework, availability of health workers and supplies address service readiness; however, other dimensions of service quality are not explicitly captured. Though simplistic, this framework gave us a structure with which to develop scenarios.

### Scenarios

We created three scenarios representing different possible futures that might unfold depending on the evolution of the pandemic and the response of governments. We sought to reflect real-world possibilities, based on past epidemics and media reports from the current pandemic. For each scenario, we developed assumptions for the four components in our framework, using the following reduction categories: none (0% reduction), small (5% reduction), moderate (10% reduction), and large (25% reduction). We assumed that provision of health services was a product of workforce and supplies, and that utilisation of health services was a product of demand and access. Coverage was assumed to be a product of provision of health services and utilisation of health services. We used a simple formula to translate component reductions to an overall coverage reduction:

$$\begin{array}{cc} \text{Coverage reduction}= & 1-([1-\text{workforce reduction}])\\ & \times [1-\text{supplies reduction}]\\ & \times [1-\text{demand reduction}]\\ & \times [1-\text{access reduction}] \end{array}$$

The intent of this calculation was to capture the idea that reductions in individual components will combine to produce a greater overall coverage reduction. A more comprehensive analysis would consider the interaction between components.

In addition to coverage reductions, we assumed an increase in the proportion of children who are wasted (low weight for height). Although changes in stunting (low height for age) will only occur and affect mortality in the long term, changes in wasting will affect mortality immediately. For our three scenarios, we assumed 10%, 20%, and 50% relative increases in wasting prevalence, respectively. Given the complex causal pathways for nutrition, these assumptions are necessarily speculative. However, we believe we would miss a large number of potential child deaths if we did not model wasting. The World Food Programme has warned that the number of people facing food crises could double because of the pandemic, so our upper assumption of a 50% relative increase seems within reason.^21^

For scenario 1 (table 1), we assume small reductions in the availability of health workers and supplies due to the reallocation of resources to the pandemic response. We also assume small reductions in the demand for and access to routine health services, due to movement restrictions, fear of infection, and economic pressure. People become disinclined to seek care for interventions deemed more acceptable to delay or forego, such as antenatal, postnatal, and preventive interventions. Care seekers have difficulty accessing services due to reduced income for out-of-pocket expenses or travel costs.

**Table 1:** Component and coverage reductions for three scenarios

	**Workforce reduction**	**Supplies reduction**	**Demand reduction**	**Access reduction**	**Coverage reduction**
Scenario 1*					
Family planning	None	Small	None	Small	9·8%
Antenatal care	Small	Small	Small	Small	18·5%
Childbirth care	Small	Small	None	Small	14·3%
Postnatal care	Small	Small	Small	Small	18·5%
Early child vaccinations	Small	Small	Small	Small	18·5%
Early child preventive	None	Small	Small	Small	14·3%
Early child curative	Small	Small	None	Small	14·3%
Scenario 2*					
Family planning	Small	Moderate	None	Small	18·8%
Antenatal care	Moderate	Moderate	Small	Small	26·9%
Childbirth care	Moderate	Moderate	None	Small	23·1%
Postnatal care	Moderate	Moderate	Small	Small	26·9%
Early child vaccinations	Moderate	Moderate	Small	Small	26·9%
Early child preventive	Small	Moderate	Small	Small	22·8%
Early child curative	Moderate	Moderate	None	Small	23·1%
Scenario 3*					
Family planning	Moderate	Moderate	None	Large	39·3%
Antenatal care	Large	Moderate	Small	Large	51·9%
Childbirth care	Large	Moderate	None	Large	49·4%
Postnatal care	Large	Moderate	Small	Large	51·9%
Early child vaccinations	Large	Moderate	Small	Large	51·9%
Early child preventive	Moderate	Moderate	Small	Large	42·3%
Early child curative	Large	Moderate	None	Large	49·4%

Small=5% reduction. Moderate=10% reduction. Large=25% reduction.

* In addition to coverage reductions, we assumed that the proportions of children with wasting would be increased by 10% in scenario 1, 20% in scenario 2, and 50% in scenario 3.

In scenario 2 (table 1), we assume greater disruptions to health systems due to workforce and supply chain issues. Some health workers are diverted to COVID-19 activities, while others become sick or are overwhelmed. Workforce shortages have a greater effect on interventions requiring skilled care (eg, antenatal, childbirth, and curative services); other interventions (eg, contraceptives and preventive services) are less dependent on skilled workers and can more easily be provided by lay health workers. Domestic supply chains are disrupted due to local bottlenecks, resulting in reduced availability of hormonal contraceptives, antenatal supplementation and malaria prevention, commodities for childbirth (eg, uterotonics, corticosteroids, or magnesium sulfate), routine child vaccines, and treatments for common childhood illnesses (eg, antibiotics, antimalarials, or oral rehydration solution).

For scenario 3 (table 1), in addition to disruptions in the health system, we assume that governments impose strict movement restrictions, forcing families and non-essential workers to stay home. Those with respiratory symptoms or suspected COVID-19 exposure are required to self-isolate. While isolation and shelter-in-place restrictions do not prohibit care seeking, the restrictions reduce access indirectly. Lack of trust in the official health system, and fear of nosocomial infection, prompts some individuals to stop seeking care or to seek care from alternative providers. Stay-at-home orders lead to greater lost income, reduced purchasing power, and inability to pay for services, compounding physical access issues. Travel becomes even more difficult for people who rely on public transport. Access restrictions felt by the broader public also affect health workers, further hampering the health workforce.

### Estimating additional deaths

We used LiST to estimate the additional deaths arising under each scenario. LiST is a causal model that estimates changes in mortality from changes in coverage of interventions.^22^ For 17 years, researchers and practitioners have used LiST to estimate the impacts of health programmes.^23^ One of LiST's strengths is its ability to model the effects of simultaneous changes in 77 interventions along the RMNCH continuum of care, including interventions related to antenatal, childbirth, and postnatal periods, and preventive and curative interventions in early childhood. Through its link to modules on demography and family planning, LiST also estimates the effects of changes in contraceptive prevalence and fertility. More information about LiST and its methodology is provided in the appendix (p 2).

We ran our models for 118 of the LMICs tracked by the Countdown to 2030 initiative (appendix p 5).^24^ By our estimates, these countries account for 97·7% of global deaths in children younger than 5 years and 99·6% of global maternal deaths.^25^, ^26^ We modelled each scenario for 1 month, and extrapolated for 3-month, 6-month, and 12-month periods, to aid interpretation. The number of additional deaths in our results represents the increase in deaths compared with a counterfactual of no change in the coverage of interventions or risk factors. We report deaths due to reduced coverage and risk factors only; we do not report deaths arising from an increase in population size from increased fertility driven by reduced contraceptive prevalence, although we do expect these effects in the long term. A link to all our LiST projection files is provided in the appendix (p 1).

We included all interventions in LiST for the categories in table 1. We did not include interventions related to breastfeeding or water and sanitation, as we assumed only a marginal reduction in these activities. As mentioned above, we included wasting, but we did not model stunting, as stunting will not occur in the short term and therefore has no immediate impact on mortality. In LiST, the effect of wasting on mortality is modelled indirectly as a risk factor that increases the likelihood of dying from other infectious causes. We did not include HIV because of the complexity of delivery systems for HIV prevention and treatment, and because HIV accounts for only a small proportion of global child deaths.

Across scenarios, we assumed similar reductions in vaccination coverage as other preventive health services (ie, antenatal and postnatal care). However, we anticipate that herd protection offered by high population vaccination coverage for rotavirus, *Haemophilus influenzae* type b vaccine, and pneumococcal conjugate vaccines will attenuate the effect of temporary reductions. We applied two assumptions in estimating the herd effect of these vaccines. First, 15% of vaccine-preventable child deaths occur in countries that do not have sufficient vaccination coverage for substantial herd protection (eg, Nigeria)^27^ and would experience the full immediate effects of decreased vaccination. Second, in countries with higher vaccination coverage, 80% of the unvaccinated cohort would remain protected by the herd effect.^28^ Because of the scarcity of evidence on the herd protection offered by meningococcal A vaccine and diphtheria–tetanus–pertussis vaccine, and the high coverage threshold for herd protection for measles vaccine, we did not attenuate the effects of these vaccines; rather, we assumed the full effect of the coverage reduction on mortality.

### Role of the funding source

The funders of the study had no role in study design, data analysis, data interpretation, or writing of the report. All authors had access to all the data in the study and had final responsibility for the decision to submit for publication.

## Results

Table 2 shows the estimated additional maternal and child deaths for each scenario among all 118 countries. These numbers represent the deaths due specifically to the reductions in coverage and increase in wasting—ie, deaths that would not occur if coverage and wasting instead stayed constant. We report child deaths excluding and including the effects of increased wasting. The estimates are reported for a 1-month period (per month) and extrapolated for 3-month, 6-month, and 12-month periods. The estimates do not reflect deaths during transition periods in which coverage is decreasing or returning to baseline. The number of additional deaths increases with the severity of the coverage reductions and wasting increases, with scenario 1 (smallest reductions) resulting in an additional 2030 maternal deaths and 42 240 child deaths per month, and scenario 3 (greatest reductions) resulting in an additional 9450 maternal deaths and 192 830 child deaths per month. Country-specific numbers are provided in the appendix (p 443).

**Table 2:** Additional deaths for each scenario among all modelled countries (n=118)

	**Per month**			**Additional deaths, n**		
	Baseline deaths, n	Additional deaths, n	Relative increase	3 months	6 months	12 months
**Maternal deaths**						
Scenario 1	24 500	2030	8·3%	6100	12 200	24 400
Scenario 2	24 500	3600	14·7%	10 800	21 600	43 100
Scenario 3	24 500	9450	38·6%	28 300	56 700	113 400
**Child deaths, excluding the effect of increased wasting**						
Scenario 1	431 690	34 750	8·0%	104 300	208 500	417 000
Scenario 2	431 690	58 910	13·6%	176 700	353 500	706 900
Scenario 3	431 690	148 870	34·5%	446 600	893 200	1 786 400
**Child deaths, including the effect of increased wasting**						
Scenario 1	431 690	42 240	9·8%	126 700	253 500	506 900
Scenario 2	431 690	74 530	17·3%	223 600	447 200	894 400
Scenario 3	431 690	192 830	44·7%	578 500	1 157 000	2 313 900

**Figure 2:**
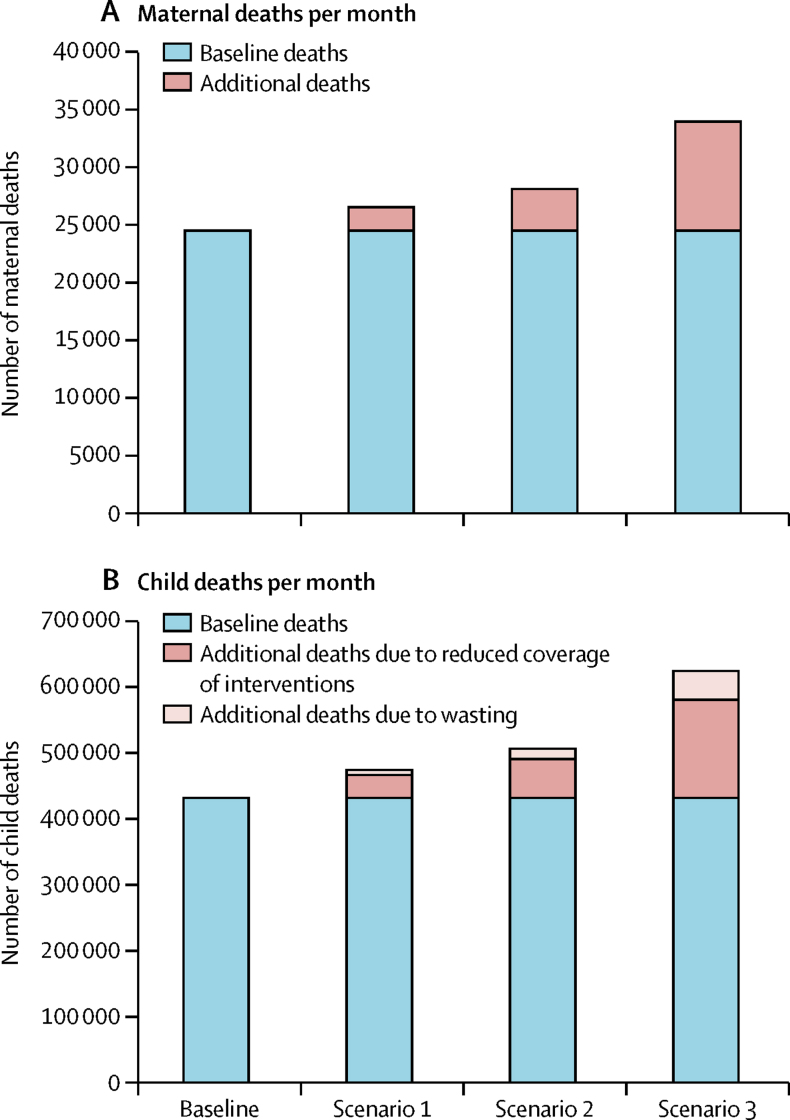
Baseline and additional maternal and child deaths per month by scenario See table 2 for values.

Table 2 and figure 2 show additional deaths compared with baseline deaths in a no-change scenario. Currently, there are approximately 24 500 maternal deaths and 431 690 child deaths per month in the 118 countries.^25^, ^26^ The additional maternal deaths would represent relative increases of 8·3% (scenario 1), 14·7% (scenario 2), and 38·6% (scenario 3) in maternal deaths per month. The additional child deaths would represent relative increases of 9·8% (scenario 1), 17·3% (scenario 2), and 44·7% (scenario 3) in child deaths per month.

Table 3, Table 4 show the contributions of individual interventions to total additional maternal and child deaths. This ranking is driven by the country-specific baseline coverage of each intervention, by the assumed coverage reductions for each intervention in our scenarios, and by the strength of each intervention on averting mortality (based on intervention efficacy and underlying country-specific causes of mortality). The reduced coverage of four childbirth interventions (namely, parenteral administration of uterotonics, antibiotics, and anticonvulsants, and clean birth environments, which reduce mortality due to post-partum haemorrhage, maternal sepsis, and eclampsia) would account for approximately 60% of additional maternal deaths (table 3). In children, an increase in wasting prevalence would account for 18–23% of additional deaths, depending on the scenario, while reduced coverage of antibiotics for pneumonia and neonatal sepsis and of oral rehydration solution for diarrhoea would together account for around 41% of additional child deaths (table 4).

**Table 3:** Additional maternal deaths per month by intervention among all modelled countries (n=118)

	**Category**	**Additional maternal deaths**		
		Scenario 1	Scenario 2	Scenario 3
Total additional deaths	..	2030	3600	9450
Parenteral administration of uterotonics	Childbirth	557 (28%)	1008 (28%)	2775 (29%)
Parenteral administration of antibiotics	Childbirth	236 (12%)	433 (12%)	1245 (13%)
Parenteral administration of anticonvulsants	Childbirth	194 (10%)	351 (10%)	968 (10%)
Clean birth environment	Childbirth	180 (9%)	328 (9%)	931 (10%)
Contraceptive use	Family planning	130 (6%)	247 (7%)	514 (5%)
Magnesium sulfate management of pre-eclampsia	Antenatal	135 (7%)	212 (6%)	483 (5%)
Micronutrient supplementation (iron and multiple micronutrients)	Antenatal	101 (5%)	159 (4%)	361 (4%)
Antibiotics for preterm or prolonged premature rupture of membranes	Childbirth	78 (4%)	143 (4%)	411 (4%)
Manual removal of placenta	Childbirth	50 (3%)	90 (3%)	245 (3%)
Removal of retained products of conception	Childbirth	46 (2%)	82 (2%)	223 (2%)
Hypertensive disorder case management	Antenatal	51 (3%)	80 (2%)	182 (2%)
Blood transfusion	Childbirth	41 (2%)	73 (2%)	198 (2%)
Households protected from malaria (insecticide-treated nets or indoor residual spraying)	Antenatal	39 (2%)	70 (2%)	152 (2%)
Safe abortion services	Family planning	29 (1%)	67 (2%)	193 (2%)
Tetanus toxoid vaccination	Antenatal	37 (2%)	59 (2%)	135 (1%)
Malaria case management	Antenatal	30 (2%)	48 (1%)	111 (1%)
Intermittent preventive treatment of malaria in pregnancy	Antenatal	28 (1%)	43 (1%)	102 (1%)
Assisted vaginal delivery	Childbirth	22 (1%)	39 (1%)	99 (1%)
Caesarean delivery	Childbirth	17 (1%)	31 (1%)	82 (1%)

Data are n (% of total deaths).

**Table 4:** Additional child deaths per month by intervention among all modelled countries (n=118)

	**Category**	**Additional child deaths**		
		Scenario 1	Scenario 2	Scenario 3
Total additional deaths	..	42 240	74 530	192 830
Increase in wasting prevalence	Wasting	7430 (18%)	15 550 (21%)	43 810 (23%)
Case management of neonatal sepsis or pneumonia	Curative	7770 (18%)	12 920 (17%)	34 390 (18%)
Oral antibiotics for pneumonia	Curative	6920 (16%)	11 760 (16%)	28 710 (15%)
Oral rehydration solution	Curative	3380 (8%)	5840 (8%)	14 800 (8%)
Thermal protection	Childbirth	2030 (5%)	3670 (5%)	9960 (5%)
Clean cord care	Childbirth	1760 (5%)	3280 (4%)	9730 (3%)
Tetanus toxoid vaccination	Antenatal	1910 (4%)	2970 (4%)	6610 (5%)
Neonatal resuscitation	Childbirth	1280 (3%)	2280 (3%)	6000 (3%)
Immediate drying and additional stimulation	Childbirth	1170 (3%)	2080 (3%)	5430 (3%)
Clean birth environment	Childbirth	890 (2%)	1630 (2%)	4600 (2%)
Measles vaccine	Vaccines	1030 (2%)	1540 (2%)	3160 (1%)
Vitamin A for treatment of measles	Curative	850 (2%)	1520 (2%)	4230 (2%)
Diphtheria–tetanus–pertussis vaccine	Vaccines	950 (2%)	1410 (2%)	2890 (2%)
Vitamin A supplementation	Preventive	830 (2%)	1350 (2%)	2550 (1%)
Assisted vaginal delivery	Childbirth	520 (1%)	920 (1%)	2400 (1%)
*Haemophilus influenzae* type b vaccine	Vaccines	560 (1%)	830 (1%)	1720 (1%)
Antibiotics for preterm or prolonged premature rupture of membranes	Childbirth	420 (1%)	750 (1%)	1960 (1%)
Parenteral administration of antibiotics	Childbirth	420 (1%)	750 (1%)	1960 (1%)
Pneumococcal conjugate vaccine	Vaccines	460 (1%)	690 (1%)	1410 (1%)
Artemisinin-based combination therapies for treatment of malaria	Curative	330 (1%)	530 (1%)	1170 (1%)
Zinc for treatment of diarrhoea	Curative	260 (1%)	450 (1%)	1140 (1%)
Antibiotics for treatment of dysentery	Curative	200 (<1%)	350 (<1%)	860 (<1%)
Caesarean delivery	Childbirth	180 (<1%)	320 (<1%)	840 (<1%)
Households protected from malaria (insecticide-treated nets or indoor residual spraying)	Preventive	130 (<1%)	230 (<1%)	520 (<1%)
Meningococcal A vaccine	Vaccines	130 (<1%)	190 (<1%)	380 (<1%)
Complementary feeding	Preventive	110 (<1%)	190 (<1%)	360 (<1%)
Maternal age and birth order	Family planning	70 (<1%)	160 (<1%)	410 (<1%)
Intermittent preventive treatment of malaria in pregnancy	Antenatal	90 (<1%)	140 (<1%)	330 (<1%)
Rotavirus vaccine	Vaccines	60 (<1%)	90 (<1%)	190 (<1%)
Syphilis detection and treatment	Antenatal	40 (<1%)	70 (<1%)	160 (<1%)

Data are n (% of total deaths).

## Discussion

Our analysis shows that if the COVID-19 pandemic results in widespread disruption to health systems and reduced access to food, LMICs can expect to see large increases in maternal and child deaths. Under our first scenario (coverage reductions of 9·8–18·5% and wasting increase of 10%), over 6 months there would be 253 500 additional child deaths and 12 200 additional maternal deaths. Under our third scenario (coverage reductions of 39·3–51·9% and wasting increase of 50%), over 6 months there would be 1 157 000 additional child deaths and 56 700 additional maternal deaths. These deaths would represent a 9·8–44·7% increase in under-5 child deaths per month, and an 8·3–38·6% increase in maternal deaths per month, across the 118 countries.

We do not intend our estimates as a prediction. Instead, we aim to show what could happen under scenarios of differing severity and duration. If countries are successful in minimising disruptions to their health systems and maintaining utilisation of RMNCH services, the number of additional deaths will be at the smaller end of our estimates. Although this might seem obvious, we see three important messages that come from this exercise.

First, the choices that governments make in responding to the pandemic will have consequences for maternal and child health. There has been debate around the trade-off between establishing movement restrictions and minimising disruptions to business and economies. Our results show that the indirect effects of the pandemic are not merely economic. If the delivery of health care is disrupted, many women and children will die. Thus, while public health experts are advocating for social distancing, there is also a public health case for ensuring access to routine care. Our estimates quantify the potential effect on RMNCH and provide a reference point for policy makers.

Second, not all health interventions are similarly susceptible to disruption or have the same effect. As policy makers consider plans to reallocate staff and resources, they might need to prioritise interventions. In our scenarios, maintaining coverage of four childbirth interventions (parenteral administration of uterotonics, antibiotics, and anticonvulsants, and clean birth environments) would save 60% of additional maternal deaths. Maintaining coverage of antibiotics for neonatal sepsis and pneumonia and oral rehydration solution for diarrhoea would save 41% of additional child deaths. Disruption of these interventions—childbirth and child curative services—cannot be mitigated through post-outbreak activities or easily averted through vertical health programmes outside of the public health system. The vulnerability of these interventions to disruption, and their substantial consequences for mortality, highlight the need to ensure provision of these services throughout the pandemic and support citizens in using these services as safely as possible.

In our scenarios, increases in childhood wasting accounted for 18–23% of additional child deaths. Although our assumptions for this were speculative, we are confident that, if wasting does increase, it will contribute greatly to child mortality. The drivers of wasting might lie outside of the health system, but there are interventions that health policy makers could consider (eg, ready-to-use therapeutic foods). More importantly, multisectoral action should be taken to mitigate increases in wasting by strengthening and expanding social safety nets and by supporting local food and agricultural systems.

Thanks to country prioritisation and the efforts of initiatives such as Gavi, the Vaccine Alliance, coverage of essential child immunisations is high in most LMICs. This high coverage grants herd protection for some vaccine-preventable diseases, attenuating the immediate effects of coverage declines on mortality. However, even short gaps in vaccination coverage can result in overall declines in population coverage, and catch-up campaigns should be prioritised in the aftermath of the pandemic.^15^

Third, once the pandemic is over, health systems must recover quickly. As life returns to normal, countries should advance resilient health systems and reinvigorate demand for routine care. The longer that coverage reductions continue, the more lives will be lost. Once care-seeking patterns are broken, they might be hard to reinstate. We should not delay in restoring health services as soon as possible if we are to minimise the lasting impact of otherwise temporary disruptions.

We stress that our scenarios are meant as hypothetical futures, to show the potential effects on maternal and child mortality if they were to occur. We are still early in the pandemic, and our assumptions could prove to be too severe or too conservative. There is not yet reliable empirical data for the effect of the pandemic on health service provision or utilisation. Moreover, our understanding of what might be possible is based largely on experiences in high-income countries; the effects of the pandemic in LMICs, and the response to it, will probably be different.^29^ For all these reasons, our results are intended to help understand the potential magnitude of the effect, not to offer exact or even approximate numbers.

We applied the same assumptions to 118 countries, and in our above results we only report aggregate numbers. Country-specific estimates can be found in the appendix (p 443). We hesitate to showcase our estimates by country because we did not incorporate information on country-specific response strategies and, without this, deeper comparisons by country are limited. However, we recognise that the principal actors who need our estimates are national decision makers. We encourage those considering country-specific scenarios to use LiST, and the projection files provided in the appendix, to investigate the indirect effects of the pandemic using assumptions tailored to their context.

LiST is constrained to a defined set of health-sector interventions, and does not estimate the effects of income, agriculture, or food markets on stunting and wasting (although changes to stunting and wasting can be entered directly). A more complex analysis might attempt to model these upstream factors. Similarly, a comprehensive analysis would consider in more detail the long-term effect of temporary reductions in vaccine coverage (assuming no catch-up campaigns), the long-term effect of fertility increases due to temporary reductions in contraceptive prevalence, and the additional deaths during a post-pandemic period in which coverage is returning to baseline. LiST does not capture individual infectious disease dynamics and therefore does not reflect the potential effects of secondary outbreaks in the absence of preventive interventions (eg, localised measles outbreaks due to a gap in measles vaccination), as modelled in other analyses.^30^ In general, we expect the pandemic to affect the health of women and children in more ways than we have captured, not fewer, including through causal pathways unknown to us now. If our estimates are overly conservative, they still highlight the need to consider maternal and child health amid the pandemic and the consequences at stake.
