# Supplementary material for: Early estimates of the indirect effects of the COVID-19 pandemic on maternal and child mortality in low-income and middle-income countries: a modelling study
Source: Lancet Glob Health. 2020 May 12;8(7):e901–8. doi: 10.1016/S2214-109X(20)30229-1 (PMC7217645; doi:10.1016/S2214-109X(20)30229-1)
Supplement: Supplementary appendix [file mmc1.pdf]

THE LANCET
Global Health

Supplementary appendix

This appendix formed part of the original submission and has been peer reviewed. We post it as supplied by the authors.

Supplement to: Roberton T, Carter ED, Chou VB. Early estimates of the indirect effects of the COVID-19 pandemic on maternal and child mortality in low-income and middle-income countries: a modelling study. *Lancet Glob Health* 2020; published online May 12. http://dx.doi.org/10.1016/S2214-109X(20)30229-1.

The Lives Saved Tool

Supplementary appendix

Overview of the Lives Saved Tool (LiST) modeling framework 2
Appendix A. Country list and data sources 5
Appendix B1. Country-specific coverage reductions for Scenario 1 9
Appendix B2. Country-specific coverage reductions for Scenario 2 172
Appendix B3. Country-specific coverage reductions for Scenario 3 306
Appendix C. Country-specific child mortality estimates for one month by scenario (including wasting) 443
Appendix D. Country-specific maternal mortality estimates for one month by scenario 447

The appendix includes supplemental tables which present the assumptions and impact estimates by country for the Lives Saved Tool (LiST) analysis examining the indirect effects of the coronavirus pandemic modeled in 118 low- and middle-income countries. Appendix A lists the countries which were included in the analysis and the most recent population-based survey which was used to estimate coverage for most interventions. Appendix B1-B3 presents the reductions in coverage of key maternal, neonatal, and child health interventions which were applied to estimate potential changes in mortality. Three different scenarios were modeled. Appendix C presents the monthly increase in the number of child deaths estimated by country under each scenario and Appendix D presents the monthly increase in the number of maternal deaths estimated by country under the same assumptions.

Additional resources and information about the Lives Saved Tool model are available via the project website

For more information, please contact: info@livessavedtool.org.

A complete set of SPECTRUM models which includes all analyzed countries (n=118) has been made available and can accessed here. For each country, four (4) models were created which include a baseline file and scenarios 1-3 which can be downloaded as separate projection files.

(https://drive.google.com/drive/folders/1ENwjV-Ybp2EoET05F91GSuLWuNRpqgcv)

Overview of the Lives Saved Tool (LiST) modeling framework

The Lives Saved Tool (LiST) is a free and publicly available model for estimating the mortality impact of changes in health intervention coverage in low- and middle-income countries (LMICs). LiST is developed by researchers at the Institute for International Programs at the Johns Hopkins Bloomberg School of Public Health, and the development and maintenance of the model is funded by the Bill & Melinda Gates Foundation. LiST has been used for over 17 years by researchers and practitioners to estimate that impact of health programs and policies. Below we summarize information about LiST as relevant to this analysis to produce early estimates of the indirect effects of the coronavirus pandemic on maternal and child mortality in LMICs (1).

Theoretical approach and basic modelling structure of LiST

LiST has been characterized as a linear, mathematical model that is deterministic. It describes fixed relationships between inputs and outputs that will produce the same outputs each time one runs the model. In LiST the primary inputs are coverage of interventions and the outputs are changes in population level of risk factors (such as wasting rates) and cause-specific mortality (such as neonatal, child mortality 1-59m, and maternal mortality). The relationship between an input with one or more outputs is specified in terms of the effectiveness of the intervention in reducing the probability of that outcome. The overarching assumption in LiST is that mortality rates and cause of death structure will not change except in response to changes in coverage of interventions. The model assumes that changes in distal variables, such as strengthening of health systems will affect mortality by increasing coverage of interventions or reducing risk factors. In the case of this analysis, the model assumes the disruption of health systems will affect mortality by decreasing coverage of interventions and increasing risk factors.

There are over 60+ health interventions available to model in LiST. Interventions included in this analysis have been grouped into categories of family planning, antenatal care, childbirth delivery care, postnatal care, vaccinations, early child preventative, and early child curative. While these health interventions have an impact on reducing stillbirths, neonatal mortality, mortality in children 1-59 months, maternal mortality or risk factors in the model, results of this paper focused on interventions' impact to reduce child (0-59 months) and maternal mortality. An intervention can have an impact to reduce multiple causes of death, and multiple interventions can have an impact on reducing the same cause of death. Some interventions reduce mortality via reducing risk factors. Figure 1 shows how interventions, risks factors, and causes of death in LiST relate to one another. More details within each categories of interventions, risk factors, and causes of death can be viewed at https://listvisualizer.org/.

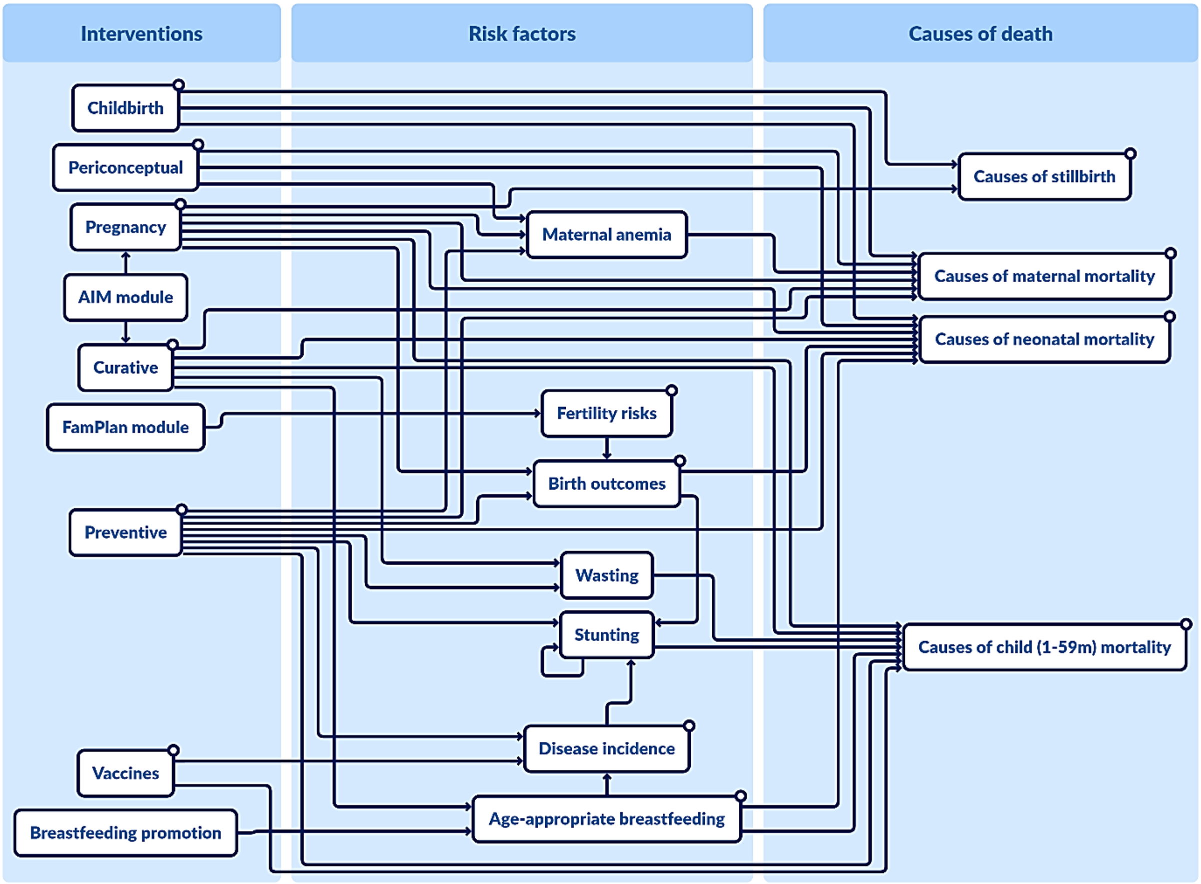

Figure 1. Impact pathways of health interventions reducing risks factors and causes of death in LiST

Age structure within LiST

LiST has a fairly simple age structure within the model that serves as a pseudo cohort. The age periods in LiST include pregnancy, 0-1, 1-5, 6-11, 12-23 and 24-59 months. Within the model impact at one age period has a cascading effect of what happens at the next.

For pregnancy, neonatal and 1-59 months there is a fixed cause of death structure in the base year (during pregnancy it is period of stillbirths). There is also a mortality rate that is applied to the age period. Within the 1-59 months period it is adjusted to reflect the higher mortality at earlier ages. Interventions within LiST can have an impact on one or more age periods.

Links to other modules in Spectrum

LiST is a linked module within the Spectrum computer software package, which houses a suite of other modules that provides necessary information for LiST to model impacts of health interventions on maternal and child mortality. The demographic module, **DemProj** contains the most recent population projections from the United Nations World Population Prospects (UNWPP) for over 150 countries. Other modules, including LiST, modifies the trends in mortality and fertility to project changes in population growth and structure in Demproj. The family planning module, **FamPlan** models impact of family planning on fertility. The HIV/AIDS module, **AIM** models impact of interventions on HIV/AIDS mortality. Contraceptive prevalence (CPR) changes for all methods among married women were included in this analysis but HIV/AIDS interventions were not.

Sources of assumptions of effectiveness of interventions in LiST

The development of the Lives Saved Tool was initially under the guidance of the Child Health Epidemiology Reference Group (CHERG) of WHO and UNICEF. CHERG, along with its institutional sponsors, developed rules of evidence to

decide what interventions should be included in the model as well as how to develop the estimates of efficacy and effectiveness used in the model (2). Since CHERG was dissolved by WHO and UNICEF, the assumptions in the model have primarily through organized reviews within topic areas. While the assumptions used within LiST are drawn from various sources, many of the assumptions about efficacy and effectiveness of interventions come from analyses published in a series of five journal supplements. CHERG also supported efforts to validate the modeled mortality impact from LiST to measured changes in coverage of interventions and mortality. Individual journal articles on effectiveness of interventions and validation studies of LiST can be found on the LiST website at https://www.livessavedtool.org/.

Process of creating a projection scenario in LiST and sources of default data

The basic process to create a projection scenario with default data is fairly simple. First, one must select a baseline year for a country. In that baseline year the country must be described in terms of a five broad sets of variables: mortality, exposure, risk factors, intervention coverage and demography. For mortality one must specify the neonatal, 1-59 months, stillbirth rates and maternal mortality rates, as well as the proportional causes of death (or stillbirths). Default mortality rates comes from the UN Inter-agency Group for Child Mortality Estimation (IGME), and the proportional causes of death come from the WHO Maternal and Child Epidemiology Estimation (MCEE) group. Exposure variables include factors such as exposure to falciparum, level of deficiency of vitamin A and zinc, and percent of the population living in poverty. Risk factors include stunting and wasting rates by age, birth outcomes, breastfeeding patterns and diarrhea and pneumonia incidence. Coverage of interventions must be provided for all intervention in LiST in the baseline year, and default coverage of most comes from Demographic and Health Surveys (DHS) or Multiple Indicator Cluster Survey (MICS).

Once a baseline year and data are set for a country, the user can then create a projection scenario by typically scaling up coverage of a single or multiple intervention over a time period. Or in the case of this analysis, scaling down coverage of interventions. Once one has created a scale up scenario, LiST then re-computes all of the inputs used in the base year based on the impact of the interventions in the scale up scenario. The levels of mortality, cause of deaths structure and levels of risk factors will be recomputed and applied to the new population structure that reflects not only the changes in DemProj but also any changes in intervention coverage from LiST, FamPlan and AIM modules.

Attribution of lives saved

A key feature of LiST is that it allows one to look at the impact of scaling up coverage of multiple interventions simultaneously, instead of a single intervention and one cause of death as is done in many natural history models. When a single intervention is scaled up, attribution is simple. However, when multiple interventions are scaled up which act on the same cause of death one must have an approach to make the attribution. In LiST we first do attribution to all preventive interventions (sequentially from periconceptual, through pregnancy, delivery and then other preventions), and then we do attribution to the treatment interventions. This means that if both a preventive and a treatment intervention are scaled up, the full effect of change in coverage of the preventive intervention is calculated and attributed to the preventive intervention. Then the residual deaths averted are attributed to the treatment scale up.

When there are two or more interventions either in preventive or treatment categories there is a second step in the attribution calculation. First, we compute the number of lives saved by applying all interventions within the preventive category in any given order. Then the attribution of lives saved to each intervention within the preventive category is based on the proportional impact of the preventive interventions, calculated as the increase in coverage times the effectiveness of the intervention.

References

1. Walker N, Tam Y, Friberg IK. Overview of the Lives Saved Tool (LiST). BMC Public Health. 2013;13 Suppl 3:S1.
2. Walker N, Fischer-Walker C, Bryce B, Bahl R, Cousens C. Standards for CHERG reviews of intervention effects on child survival. Int J Epidemiol. 2010;39.

The Lives Saved Tool

Appendix A. Country list and data sources

Listed below are the countries which were included (n=118) from the total roster of Countdown to 2030 countries (n=139). Countries without demographic or population-level coverage data were excluded from the Lives Saved Tool (LiST) analysis.

	Country	Data source
1	Afghanistan	DHS 2015
2	Albania	DHS 2017-2018
3	Algeria	MICS 2012
4	American Samoa	not in Spectrum
5	Angola	DHS 2015
6	Argentina	MICS 2011
7	Armenia	DHS 2015-2016
8	Azerbaijan	DHS 2006
9	Bangladesh	MICS 2019
10	Belarus	MICS 2012
11	Belize	MICS 2015
12	Benin	DHS 2017-2018
13	Bhutan	MICS 2010
14	Bolivia (Plurinational State of)	DHS 2008
15	Bosnia and Herzegovina	MICS 2011
16	Botswana	Family Health Survey 2007
17	Brazil	PHDS 2006
18	Bulgaria	no household survey
19	Burkina Faso	DHS 2010
20	Burundi	DHS 2016
21	Cabo Verde	DHS 2005
22	Cambodia	DHS 2014
23	Cameroon	MICS 2014
24	Central African Republic	MICS 2010
25	Chad	DHS 2014
26	China	no household survey
27	Colombia	DHS 2015
28	Comoros	DHS 2012
29	Congo	MICS 2014
30	Congo (Democratic Republic of the)	DHS 2013
31	Costa Rica	MICS 2011

	Country	Data source
32	Cuba	MICS 2014
33	Cote d'Ivoire	MICS 2016
34	Djibouti	MICS 2006
35	Dominica	no household survey
36	Dominican Republic	MICS 2014
37	Ecuador	NSS 2012
38	Egypt	DHS 2014
39	El Salvador	MICS 2014
40	Equatorial Guinea	DHS 2011
41	Eritrea	DHS 2002
42	Ethiopia	DHS 2016
43	Fiji	no household survey
44	Gabon	DHS 2012
45	Gambia	MICS 2018
46	Georgia	MICS 2018
47	Ghana	DHS 2014
48	Grenada	no household survey
49	Guatemala	DHS 2014
50	Guinea	MICS 2016
51	Guinea-Bissau	MICS 2014
52	Guyana	MICS 2014
53	Haiti	DHS 2016
54	Honduras	DHS 2011
55	India	DHS 2015
56	Indonesia	DHS 2012
57	Iran	no household survey
58	Iraq	MICS 2018
59	Jamaica	MICS 2011
60	Jordan	PFHS 2017-2018
61	Kazakhstan	MICS 2015
62	Kenya	DHS 2014
63	Kiribati	DHS 2009
64	Korea (Democratic People's Republic of)	MICS 2017
65	Kosovo	not in Spectrum
66	Kyrgyzstan	MICS 2018
67	Lao People's Democratic Republic	MICS 2017
68	Lebanon	no household survey
69	Lesotho	MICS 2018

	Country	Data source
70	Liberia	DHS 2013
71	Libya	no household survey
72	Madagascar	MDG Survey 2012
73	Malawi	DHS 2015
74	Malaysia	no household survey
75	Maldives	DHS 2016-2017
76	Mali	MICS 2015
77	Marshall Islands	not in Spectrum
78	Mauritania	MICS 2015
79	Mauritius	no household survey
80	Mexico	MICS 2015
81	Micronesia (Federated States of)	no household survey
82	Moldova	MICS 2012
83	Mongolia	MICS 2018
84	Montenegro	MICS 2018
85	Morocco	DHS 2003
86	Mozambique	DHS 2015
87	Myanmar	DHS 2015
88	Namibia	DHS 2013
89	Nauru	not in Spectrum
90	Nepal	DHS 2016
91	Nicaragua	RHS 2006
92	Niger	DHS 2012
93	Nigeria	DHS 2018
94	North Macedonia	MICS 2011
95	Pakistan	DHS 2017-2018
96	Panama	MICS 2013
97	Papua New Guinea	DHS 2016-2018
98	Paraguay	MICS 2016
99	Peru	DHS 2016
100	Philippines	DHS 2017
101	Romania	no household survey
102	Russian Federation	no household survey
103	Rwanda	DHS 2014
104	Saint Lucia	MICS 2012
105	Saint Vincent and the Grenadines	no household survey
106	Samoa	DHS 2009
107	Sao Tome and Principe	MICS 2014

	Country	Data source
108	Senegal	DHS 2017
109	Serbia	MICS 2014
110	Sierra Leone	MICS 2017
111	Solomon Islands	DHS 2006
112	Somalia	MICS 2006
113	South Africa	DHS 2016
114	South Sudan	MICS 2010
115	Sri Lanka	DHS 2016
116	State of Palestine	causes of death not available
117	Sudan	MICS 2014
118	Suriname	MICS 2010
119	Swaziland	MICS 2014
120	Syrian Arab Republic	MICS 2006
121	Tajikistan	DHS 2017-2018
122	Tanzania	DHS 2015
123	Thailand	MICS 2015
124	Timor-Leste	DHS 2016
125	Togo	DHS 2013
126	Tonga	DHS 2012
127	Tunisia	MICS 2018
128	Turkey	DHS 2013
129	Turkmenistan	MICS 2015
130	Tuvalu	not in Spectrum
131	Uganda	DHS 2016
132	Ukraine	MICS 2012
133	Uzbekistan	MICS 2006
134	Vanuatu	DHS 2013
135	Venezuela (Bolivarian Republic of)	no household survey
136	Vietnam	MICS 2013-2014
137	Yemen	DHS 2013
138	Zambia	DHS 2013
139	Zimbabwe	MICS 2019

Appendix B1. Country-specific coverage reductions for Scenario 1

**Country**	**ISO 3166-1 alpha-3**	**Intervention Name**	**Baseline coverage (%)**	**Ending coverage (%)**
Afghanistan	AFG	Safe abortion services	35.1	31.7
Afghanistan	AFG	TT - Tetanus toxoid vaccination	70.0	57.0
Afghanistan	AFG	Syphilis detection and treatment	14.6	11.9
Afghanistan	AFG	Iron supplementation in pregnancy	6.8	5.5
Afghanistan	AFG	Hypertensive disorder case management	4.3	3.5
Afghanistan	AFG	Diabetes case management	3.3	2.7
Afghanistan	AFG	Malaria case management	13.8	11.2
Afghanistan	AFG	MgSO4 management of pre-eclampsia	8.5	6.9
Afghanistan	AFG	Thermal protection	47.7	40.9
Afghanistan	AFG	Clean cord care	46.1	39.5
Afghanistan	AFG	Clean birth environment	39.6	34.0
Afghanistan	AFG	Immediate drying and additional stimulation	44.2	37.9
Afghanistan	AFG	Neonatal resuscitation	26.6	22.8
Afghanistan	AFG	Antibiotics for preterm or prolonged PROM	36.1	31.0
Afghanistan	AFG	Parenteral administration of anti-convulsants	34.5	29.6
Afghanistan	AFG	Parenteral administration of uterotonics	43.2	37.0
Afghanistan	AFG	Parenteral administration of antibiotics	36.1	31.0
Afghanistan	AFG	Assisted vaginal delivery	12.2	10.5
Afghanistan	AFG	Manual removal of placenta	18.1	15.5
Afghanistan	AFG	Removal of retained products of conception	16.0	13.7
Afghanistan	AFG	Cesarean delivery	4.2	3.6
Afghanistan	AFG	Blood transfusion	6.1	5.2
Afghanistan	AFG	Induction of labor for pregnancies lasting 41+ weeks	0.9	0.8
Afghanistan	AFG	Complementary feeding - education only	24.3	20.8
Afghanistan	AFG	Complementary feeding - supplementary feeding and education	24.3	20.8
Afghanistan	AFG	Vitamin A supplementation	95.0	81.5
Afghanistan	AFG	Improved sanitation - Utilization of latrines or toilets	43.4	43.4
Afghanistan	AFG	Improved water source	67.1	67.1
Afghanistan	AFG	Water connection in the home	14.5	14.5
Afghanistan	AFG	Hand washing with soap	45.6	45.6
Afghanistan	AFG	Hygienic disposal of children's stools	30.9	30.9

Afghanistan	AFG	ITN/IRS - Households protected from malaria	26.0	22.3
Afghanistan	AFG	Injectable antibiotics for neonatal sepsis	48.3	39.3
Afghanistan	AFG	ORS - oral rehydration solution	46.2	39.6
Afghanistan	AFG	Antibiotics for treatment of dysentery	8.2	7.0
Afghanistan	AFG	Zinc for treatment of diarrhea	9.6	8.2
Afghanistan	AFG	Oral antibiotics for pneumonia	61.4	52.6
Afghanistan	AFG	Vitamin A for treatment of measles	95.0	81.5
Afghanistan	AFG	SAM - treatment for severe acute malnutrition	9.2	7.9
Afghanistan	AFG	BCG vaccine	78.0	63.5
Afghanistan	AFG	Polio vaccine	73.0	59.5
Afghanistan	AFG	DPT vaccine	66.0	53.8
Afghanistan	AFG	H. influenzae type b vaccine	66.0	62.1
Afghanistan	AFG	HepB vaccine	66.0	53.8
Afghanistan	AFG	Pneumococcal vaccine	65.0	61.1
Afghanistan	AFG	Rotavirus vaccine	60.0	56.4
Afghanistan	AFG	Measles vaccine	64.0	52.1
Afghanistan	AFG	Global wasting (<-2 SD) rate	10.6	11.7
Afghanistan	AFG	Contraceptive prevalence (CPR)	27.8	25.1
Albania	ALB	Safe abortion services	100.0	90.3
Albania	ALB	TT - Tetanus toxoid vaccination	95.0	77.4
Albania	ALB	Syphilis detection and treatment	21.9	17.8
Albania	ALB	Iron supplementation in pregnancy	18.5	15.1
Albania	ALB	Hypertensive disorder case management	19.0	15.5
Albania	ALB	Diabetes case management	14.8	12.1
Albania	ALB	Malaria case management	61.2	49.8
Albania	ALB	MgSO4 management of pre-eclampsia	37.5	30.5
Albania	ALB	Thermal protection	97.4	83.5
Albania	ALB	Clean cord care	94.1	80.7
Albania	ALB	Clean birth environment	80.9	69.4
Albania	ALB	Immediate drying and additional stimulation	90.3	77.4
Albania	ALB	Neonatal resuscitation	54.2	46.5
Albania	ALB	Antibiotics for preterm or prolonged PROM	73.8	63.3
Albania	ALB	Parenteral administration of anti-convulsants	70.5	60.4
Albania	ALB	Parenteral administration of uterotonics	88.1	75.5
Albania	ALB	Parenteral administration of antibiotics	73.8	63.3

Albania	ALB	Assisted vaginal delivery	24.9	21.3
Albania	ALB	Manual removal of placenta	36.9	31.6
Albania	ALB	Removal of retained products of conception	32.7	28.0
Albania	ALB	Cesarean delivery	8.6	7.4
Albania	ALB	Blood transfusion	12.4	10.6
Albania	ALB	Induction of labor for pregnancies lasting 41+ weeks	1.7	1.5
Albania	ALB	Complementary feeding - education only	59.3	50.8
Albania	ALB	Complementary feeding - supplementary feeding and education	59.3	50.8
Albania	ALB	Improved sanitation - Utilization of latrines or toilets	97.7	97.7
Albania	ALB	Improved water source	91.0	91.0
Albania	ALB	Water connection in the home	78.5	78.5
Albania	ALB	Hygienic disposal of children's stools	13.5	13.5
Albania	ALB	Injectable antibiotics for neonatal sepsis	98.6	80.3
Albania	ALB	ORS - oral rehydration solution	34.5	29.6
Albania	ALB	Antibiotics for treatment of dysentery	38.7	33.2
Albania	ALB	Zinc for treatment of diarrhea	14.9	12.8
Albania	ALB	Oral antibiotics for pneumonia	81.8	70.1
Albania	ALB	BCG vaccine	99.0	80.6
Albania	ALB	Polio vaccine	99.0	80.6
Albania	ALB	DPT vaccine	99.0	80.6
Albania	ALB	H. influenzae type b vaccine	99.0	93.1
Albania	ALB	HepB vaccine	99.0	80.6
Albania	ALB	Pneumococcal vaccine	98.0	92.2
Albania	ALB	Measles vaccine	94.0	76.6
Albania	ALB	Global wasting (<-2 SD) rate	1.4	1.5
Albania	ALB	Contraceptive prevalence (CPR)	44.0	39.7
Algeria	DZA	Safe abortion services	2.2	2.0
Algeria	DZA	TT - Tetanus toxoid vaccination	98.0	79.8
Algeria	DZA	Syphilis detection and treatment	22.9	18.7
Algeria	DZA	Hypertensive disorder case management	16.2	13.2
Algeria	DZA	Diabetes case management	12.6	10.3
Algeria	DZA	Malaria case management	52.1	42.4
Algeria	DZA	MgSO4 management of pre-eclampsia	32.0	26.1
Algeria	DZA	Thermal protection	95.5	81.9

Algeria	DZA	Clean cord care	92.2	79.0
Algeria	DZA	Clean birth environment	79.2	67.9
Algeria	DZA	Immediate drying and additional stimulation	88.5	75.9
Algeria	DZA	Neonatal resuscitation	53.1	45.5
Algeria	DZA	Antibiotics for preterm or prolonged PROM	72.3	62.0
Algeria	DZA	Parenteral administration of anti-convulsants	69.1	59.2
Algeria	DZA	Parenteral administration of uterotonics	86.3	74.0
Algeria	DZA	Parenteral administration of antibiotics	72.3	62.0
Algeria	DZA	Assisted vaginal delivery	24.4	20.9
Algeria	DZA	Manual removal of placenta	36.1	31.0
Algeria	DZA	Removal of retained products of conception	32.0	27.4
Algeria	DZA	Cesarean delivery	8.4	7.2
Algeria	DZA	Blood transfusion	12.2	10.5
Algeria	DZA	Induction of labor for pregnancies lasting 41+ weeks	1.7	1.5
Algeria	DZA	Improved sanitation - Utilization of latrines or toilets	87.6	87.6
Algeria	DZA	Improved water source	93.6	93.6
Algeria	DZA	Water connection in the home	71.8	71.8
Algeria	DZA	Hand washing with soap	84.2	84.2
Algeria	DZA	Hygienic disposal of children's stools	17.7	17.7
Algeria	DZA	Injectable antibiotics for neonatal sepsis	96.6	78.7
Algeria	DZA	ORS - oral rehydration solution	33.9	29.1
Algeria	DZA	Oral antibiotics for pneumonia	66.4	56.9
Algeria	DZA	BCG vaccine	99.0	80.6
Algeria	DZA	Polio vaccine	91.0	74.1
Algeria	DZA	DPT vaccine	91.0	74.1
Algeria	DZA	H. influenzae type b vaccine	91.0	85.6
Algeria	DZA	HepB vaccine	91.0	74.1
Algeria	DZA	Pneumococcal vaccine	91.0	85.6
Algeria	DZA	Measles vaccine	80.0	65.2
Algeria	DZA	Global wasting (<-2 SD) rate	4.0	4.4
Algeria	DZA	Contraceptive prevalence (CPR)	61.05	55.1
Angola	AGO	TT - Tetanus toxoid vaccination	78.0	63.5
Angola	AGO	IPTp - Intermittent preventive treatment of malaria during pregnancy	38.4	31.3
Angola	AGO	Syphilis detection and treatment	20.0	16.3

Angola	AGO	Iron supplementation in pregnancy	32.1	26.1
Angola	AGO	Hypertensive disorder case management	14.5	11.8
Angola	AGO	Diabetes case management	11.3	9.2
Angola	AGO	Malaria case management	46.7	38.0
Angola	AGO	MgSO4 management of pre-eclampsia	28.6	23.3
Angola	AGO	Thermal protection	45.1	38.7
Angola	AGO	Clean cord care	43.5	37.3
Angola	AGO	Clean birth environment	37.4	32.1
Angola	AGO	Immediate drying and additional stimulation	41.8	35.8
Angola	AGO	Neonatal resuscitation	25.1	21.5
Angola	AGO	Antibiotics for preterm or prolonged PROM	34.1	29.2
Angola	AGO	Parenteral administration of anti-convulsants	32.6	28.0
Angola	AGO	Parenteral administration of uterotonics	40.8	35.0
Angola	AGO	Parenteral administration of antibiotics	34.1	29.2
Angola	AGO	Assisted vaginal delivery	11.5	9.9
Angola	AGO	Manual removal of placenta	17.1	14.7
Angola	AGO	Removal of retained products of conception	15.1	12.9
Angola	AGO	Cesarean delivery	4.0	3.4
Angola	AGO	Blood transfusion	5.7	4.9
Angola	AGO	Induction of labor for pregnancies lasting 41+ weeks	0.8	0.7
Angola	AGO	Complementary feeding - education only	33.3	28.6
Angola	AGO	Complementary feeding - supplementary feeding and education	33.3	28.6
Angola	AGO	Vitamin A supplementation	3.0	2.6
Angola	AGO	Improved sanitation - Utilization of latrines or toilets	49.9	49.9
Angola	AGO	Improved water source	55.8	55.8
Angola	AGO	Water connection in the home	22.6	22.6
Angola	AGO	Hand washing with soap	27.0	27.0
Angola	AGO	Hygienic disposal of children's stools	27.5	27.5
Angola	AGO	ITN/IRS - Households protected from malaria	31.8	27.3
Angola	AGO	Injectable antibiotics for neonatal sepsis	45.6	37.1
Angola	AGO	ORS - oral rehydration solution	42.6	36.5
Angola	AGO	Oral antibiotics for pneumonia	58.7	50.3
Angola	AGO	Vitamin A for treatment of measles	3.0	2.6

Angola	AGO	ACTs- Artemisinin compounds for treatment of malaria	9.0	7.7
Angola	AGO	SAM - treatment for severe acute malnutrition	1.4	1.2
Angola	AGO	BCG vaccine	86.0	70.0
Angola	AGO	Polio vaccine	56.0	45.6
Angola	AGO	DPT vaccine	59.0	48.1
Angola	AGO	H. influenzae type b vaccine	59.0	55.5
Angola	AGO	HepB vaccine	59.0	48.1
Angola	AGO	Pneumococcal vaccine	67.0	63.0
Angola	AGO	Rotavirus vaccine	65.0	61.1
Angola	AGO	Measles vaccine	50.0	40.7
Angola	AGO	Global wasting (<-2 SD) rate	4.9	5.4
Angola	AGO	Contraceptive prevalence (CPR)	16.25	14.7
Argentina	ARG	Safe abortion services	0.2	0.2
Argentina	ARG	Syphilis detection and treatment	24.2	19.7
Argentina	ARG	Hypertensive disorder case management	21.6	17.6
Argentina	ARG	Diabetes case management	16.8	13.7
Argentina	ARG	Malaria case management	69.6	56.7
Argentina	ARG	MgSO4 management of pre-eclampsia	42.7	34.8
Argentina	ARG	Thermal protection	97.1	83.3
Argentina	ARG	Clean cord care	93.7	80.3
Argentina	ARG	Clean birth environment	80.5	69.0
Argentina	ARG	Immediate drying and additional stimulation	89.9	77.1
Argentina	ARG	Neonatal resuscitation	54.0	46.3
Argentina	ARG	Antibiotics for preterm or prolonged PROM	73.5	63.0
Argentina	ARG	Parenteral administration of anti-convulsants	70.2	60.2
Argentina	ARG	Parenteral administration of uterotonics	87.8	75.3
Argentina	ARG	Parenteral administration of antibiotics	73.5	63.0
Argentina	ARG	Assisted vaginal delivery	24.8	21.3
Argentina	ARG	Manual removal of placenta	36.7	31.5
Argentina	ARG	Removal of retained products of conception	32.5	27.9
Argentina	ARG	Cesarean delivery	8.5	7.3
Argentina	ARG	Blood transfusion	12.4	10.6
Argentina	ARG	Induction of labor for pregnancies lasting 41+ weeks	1.7	1.5
Argentina	ARG	Improved sanitation - Utilization of latrines or toilets	94.3	94.3

Argentina	ARG	Improved water source	99.0	99.0
Argentina	ARG	Water connection in the home	95.7	95.7
Argentina	ARG	Injectable antibiotics for neonatal sepsis	98.2	80.0
Argentina	ARG	ORS - oral rehydration solution	17.5	15.0
Argentina	ARG	Oral antibiotics for pneumonia	94.5	81.0
Argentina	ARG	BCG vaccine	93.0	75.7
Argentina	ARG	Polio vaccine	84.0	68.4
Argentina	ARG	DPT vaccine	86.0	70.0
Argentina	ARG	H. influenzae type b vaccine	86.0	80.9
Argentina	ARG	HepB vaccine	86.0	70.0
Argentina	ARG	Pneumococcal vaccine	88.0	82.8
Argentina	ARG	Rotavirus vaccine	80.0	75.3
Argentina	ARG	Measles vaccine	89.0	72.5
Argentina	ARG	Global wasting (<-2 SD) rate	2.4	2.7
Argentina	ARG	Contraceptive prevalence (CPR)	63.95	57.7
Armenia	ARM	Safe abortion services	40.0	36.1
Armenia	ARM	Syphilis detection and treatment	24.7	20.1
Armenia	ARM	Iron supplementation in pregnancy	4.5	3.7
Armenia	ARM	Hypertensive disorder case management	23.2	18.9
Armenia	ARM	Diabetes case management	18.1	14.7
Armenia	ARM	Malaria case management	74.7	60.8
Armenia	ARM	MgSO4 management of pre-eclampsia	45.8	37.3
Armenia	ARM	Thermal protection	98.5	84.5
Armenia	ARM	Clean cord care	95.1	81.5
Armenia	ARM	Clean birth environment	81.8	70.1
Armenia	ARM	Immediate drying and additional stimulation	91.3	78.3
Armenia	ARM	Neonatal resuscitation	54.8	47.0
Armenia	ARM	Antibiotics for preterm or prolonged PROM	74.6	64.0
Armenia	ARM	Parenteral administration of anti-convulsants	71.3	61.1
Armenia	ARM	Parenteral administration of uterotonics	89.1	76.4
Armenia	ARM	Parenteral administration of antibiotics	74.6	64.0
Armenia	ARM	Assisted vaginal delivery	25.2	21.6
Armenia	ARM	Manual removal of placenta	37.3	32.0
Armenia	ARM	Removal of retained products of conception	33.0	28.3
Armenia	ARM	Cesarean delivery	8.7	7.5

Armenia	ARM	Blood transfusion	12.6	10.8
Armenia	ARM	Induction of labor for pregnancies lasting 41+ weeks	1.8	1.5
Armenia	ARM	Complementary feeding - education only	50.2	43.0
Armenia	ARM	Complementary feeding - supplementary feeding and education	50.2	43.0
Armenia	ARM	Improved sanitation - Utilization of latrines or toilets	93.6	93.6
Armenia	ARM	Improved water source	99.0	99.0
Armenia	ARM	Water connection in the home	97.9	97.9
Armenia	ARM	Hand washing with soap	95.8	95.8
Armenia	ARM	Hygienic disposal of children's stools	43.4	43.4
Armenia	ARM	Injectable antibiotics for neonatal sepsis	99.7	81.2
Armenia	ARM	ORS - oral rehydration solution	36.9	31.6
Armenia	ARM	Antibiotics for treatment of dysentery	1.3	1.1
Armenia	ARM	Zinc for treatment of diarrhea	3.7	3.2
Armenia	ARM	Oral antibiotics for pneumonia	91.7	78.6
Armenia	ARM	BCG vaccine	99.0	80.6
Armenia	ARM	Polio vaccine	92.0	74.9
Armenia	ARM	DPT vaccine	92.0	74.9
Armenia	ARM	H. influenzae type b vaccine	92.0	86.5
Armenia	ARM	HepB vaccine	92.0	74.9
Armenia	ARM	Pneumococcal vaccine	92.0	86.5
Armenia	ARM	Rotavirus vaccine	93.0	87.5
Armenia	ARM	Meningococcal A	22.0	17.9
Armenia	ARM	Measles vaccine	95.0	77.4
Armenia	ARM	Global wasting (<-2 SD) rate	4.5	4.9
Armenia	ARM	Contraceptive prevalence (CPR)	58.55	52.8
Azerbaijan	AZE	Safe abortion services	40.0	36.1
Azerbaijan	AZE	Syphilis detection and treatment	19.6	16.0
Azerbaijan	AZE	Iron supplementation in pregnancy	1.6	1.3
Azerbaijan	AZE	Hypertensive disorder case management	11.8	9.6
Azerbaijan	AZE	Diabetes case management	9.2	7.5
Azerbaijan	AZE	Malaria case management	38.1	31.0
Azerbaijan	AZE	MgSO4 management of pre-eclampsia	23.4	19.1
Azerbaijan	AZE	Thermal protection	76.8	65.8
Azerbaijan	AZE	Clean cord care	74.1	63.5

Azerbaijan	AZE	Clean birth environment	63.7	54.6
Azerbaijan	AZE	Immediate drying and additional stimulation	71.1	61.0
Azerbaijan	AZE	Neonatal resuscitation	42.7	36.6
Azerbaijan	AZE	Antibiotics for preterm or prolonged PROM	58.1	49.8
Azerbaijan	AZE	Parenteral administration of anti-convulsants	55.5	47.6
Azerbaijan	AZE	Parenteral administration of uterotonics	69.4	59.5
Azerbaijan	AZE	Parenteral administration of antibiotics	58.1	49.8
Azerbaijan	AZE	Assisted vaginal delivery	19.6	16.8
Azerbaijan	AZE	Manual removal of placenta	29.0	24.9
Azerbaijan	AZE	Removal of retained products of conception	25.7	22.0
Azerbaijan	AZE	Cesarean delivery	6.8	5.8
Azerbaijan	AZE	Blood transfusion	9.8	8.4
Azerbaijan	AZE	Induction of labor for pregnancies lasting 41+ weeks	1.4	1.2
Azerbaijan	AZE	Complementary feeding - education only	50.9	43.6
Azerbaijan	AZE	Complementary feeding - supplementary feeding and education	50.9	43.6
Azerbaijan	AZE	Vitamin A supplementation	41.0	35.2
Azerbaijan	AZE	Improved sanitation - Utilization of latrines or toilets	92.5	92.5
Azerbaijan	AZE	Improved water source	91.4	91.4
Azerbaijan	AZE	Water connection in the home	78.5	78.5
Azerbaijan	AZE	Injectable antibiotics for neonatal sepsis	77.7	63.3
Azerbaijan	AZE	ORS - oral rehydration solution	21.1	18.1
Azerbaijan	AZE	Oral antibiotics for pneumonia	32.5	27.9
Azerbaijan	AZE	Vitamin A for treatment of measles	41.0	35.2
Azerbaijan	AZE	BCG vaccine	97.0	79.0
Azerbaijan	AZE	Polio vaccine	96.0	78.2
Azerbaijan	AZE	DPT vaccine	95.0	77.4
Azerbaijan	AZE	H. influenzae type b vaccine	95.0	89.4
Azerbaijan	AZE	HepB vaccine	95.0	77.4
Azerbaijan	AZE	Pneumococcal vaccine	95.0	89.4
Azerbaijan	AZE	Measles vaccine	96.0	78.2
Azerbaijan	AZE	Global wasting (<-2 SD) rate	6.6	7.3
Azerbaijan	AZE	Contraceptive prevalence (CPR)	57	51.4
Bangladesh	BGD	Safe abortion services	35.1	31.7
Bangladesh	BGD	TT - Tetanus toxoid vaccination	98.0	79.8

Bangladesh	BGD	Syphilis detection and treatment	7.3	5.9
Bangladesh	BGD	Hypertensive disorder case management	8.9	7.2
Bangladesh	BGD	Diabetes case management	12.6	10.3
Bangladesh	BGD	Malaria case management	28.6	23.3
Bangladesh	BGD	MgSO4 management of pre-eclampsia	8.0	6.5
Bangladesh	BGD	Thermal protection	51.3	44.0
Bangladesh	BGD	Clean cord care	51.0	43.7
Bangladesh	BGD	Clean birth environment	37.8	32.4
Bangladesh	BGD	Immediate drying and additional stimulation	39.3	33.7
Bangladesh	BGD	Neonatal resuscitation	40.0	34.3
Bangladesh	BGD	Antibiotics for preterm or prolonged PROM	23.9	20.5
Bangladesh	BGD	Parenteral administration of anti-convulsants	37.8	32.4
Bangladesh	BGD	Parenteral administration of uterotonics	42.0	36.0
Bangladesh	BGD	Parenteral administration of antibiotics	23.9	20.5
Bangladesh	BGD	Assisted vaginal delivery	26.5	22.7
Bangladesh	BGD	Manual removal of placenta	12.2	10.5
Bangladesh	BGD	Removal of retained products of conception	30.2	25.9
Bangladesh	BGD	Cesarean delivery	7.6	6.5
Bangladesh	BGD	Blood transfusion	10.8	9.3
Bangladesh	BGD	Induction of labor for pregnancies lasting 41+ weeks	10.2	8.7
Bangladesh	BGD	Complementary feeding - education only	27.6	23.7
Bangladesh	BGD	Complementary feeding - supplementary feeding and education	27.6	23.7
Bangladesh	BGD	Vitamin A supplementation	99.0	84.9
Bangladesh	BGD	Improved sanitation - Utilization of latrines or toilets	48.2	48.2
Bangladesh	BGD	Improved water source	97.0	97.0
Bangladesh	BGD	Water connection in the home	14.5	14.5
Bangladesh	BGD	Hand washing with soap	74.8	74.8
Bangladesh	BGD	Hygienic disposal of children's stools	38.7	38.7
Bangladesh	BGD	Injectable antibiotics for neonatal sepsis	53.4	43.5
Bangladesh	BGD	ORS - oral rehydration solution	72.4	62.1
Bangladesh	BGD	Antibiotics for treatment of dysentery	9.7	8.3
Bangladesh	BGD	Zinc for treatment of diarrhea	43.6	37.4
Bangladesh	BGD	Oral antibiotics for pneumonia	46.4	39.8
Bangladesh	BGD	Vitamin A for treatment of measles	99.0	84.9

Bangladesh	BGD	SAM - treatment for severe acute malnutrition	0.3	0.3
Bangladesh	BGD	BCG vaccine	99.0	80.6
Bangladesh	BGD	Polio vaccine	98.0	79.8
Bangladesh	BGD	DPT vaccine	98.0	79.8
Bangladesh	BGD	H. influenzae type b vaccine	98.0	92.2
Bangladesh	BGD	HepB vaccine	98.0	79.8
Bangladesh	BGD	Pneumococcal vaccine	97.0	91.2
Bangladesh	BGD	Measles vaccine	97.0	79.0
Bangladesh	BGD	Global wasting (<-2 SD) rate	14.4	15.8
Bangladesh	BGD	Contraceptive prevalence (CPR)	65.15	58.8
Belarus	BLR	Safe abortion services	87.5	79.0
Belarus	BLR	Syphilis detection and treatment	24.6	20.0
Belarus	BLR	Hypertensive disorder case management	23.9	19.5
Belarus	BLR	Diabetes case management	18.7	15.2
Belarus	BLR	Malaria case management	77.3	63.0
Belarus	BLR	MgSO4 management of pre-eclampsia	47.4	38.6
Belarus	BLR	Thermal protection	98.7	84.6
Belarus	BLR	Clean cord care	95.3	81.7
Belarus	BLR	Clean birth environment	81.9	70.2
Belarus	BLR	Immediate drying and additional stimulation	91.5	78.4
Belarus	BLR	Neonatal resuscitation	54.9	47.1
Belarus	BLR	Antibiotics for preterm or prolonged PROM	74.8	64.1
Belarus	BLR	Parenteral administration of anti-convulsants	71.4	61.2
Belarus	BLR	Parenteral administration of uterotonics	89.3	76.6
Belarus	BLR	Parenteral administration of antibiotics	74.8	64.1
Belarus	BLR	Assisted vaginal delivery	25.3	21.7
Belarus	BLR	Manual removal of placenta	37.4	32.1
Belarus	BLR	Removal of retained products of conception	33.1	28.4
Belarus	BLR	Cesarean delivery	8.7	7.5
Belarus	BLR	Blood transfusion	12.6	10.8
Belarus	BLR	Induction of labor for pregnancies lasting 41+ weeks	1.8	1.5
Belarus	BLR	Improved sanitation - Utilization of latrines or toilets	97.8	97.8
Belarus	BLR	Improved water source	96.5	96.5
Belarus	BLR	Water connection in the home	89.6	89.6

Belarus	BLR	Hygienic disposal of children's stools	56.0	56.0
Belarus	BLR	Injectable antibiotics for neonatal sepsis	99.9	81.4
Belarus	BLR	ORS - oral rehydration solution	45.3	38.8
Belarus	BLR	Antibiotics for treatment of dysentery	22.3	19.1
Belarus	BLR	Oral antibiotics for pneumonia	93.4	80.1
Belarus	BLR	BCG vaccine	98.0	79.8
Belarus	BLR	Polio vaccine	98.0	79.8
Belarus	BLR	DPT vaccine	97.0	79.0
Belarus	BLR	H. influenzae type b vaccine	9.0	8.5
Belarus	BLR	HepB vaccine	98.0	79.8
Belarus	BLR	Measles vaccine	97.0	79.0
Belarus	BLR	Global wasting (<-2 SD) rate	2.2	2.4
Belarus	BLR	Contraceptive prevalence (CPR)	66.6	60.1
Belize	BLZ	Safe abortion services	0.6	0.5
Belize	BLZ	TT - Tetanus toxoid vaccination	91.0	74.1
Belize	BLZ	Syphilis detection and treatment	24.0	19.5
Belize	BLZ	Hypertensive disorder case management	22.3	18.2
Belize	BLZ	Diabetes case management	17.4	14.2
Belize	BLZ	Malaria case management	71.8	58.5
Belize	BLZ	MgSO4 management of pre-eclampsia	44.0	35.8
Belize	BLZ	Thermal protection	95.3	81.7
Belize	BLZ	Clean cord care	92.0	78.9
Belize	BLZ	Clean birth environment	79.1	67.8
Belize	BLZ	Immediate drying and additional stimulation	88.3	75.7
Belize	BLZ	Neonatal resuscitation	53.0	45.4
Belize	BLZ	Antibiotics for preterm or prolonged PROM	72.1	61.8
Belize	BLZ	Parenteral administration of anti-convulsants	68.9	59.1
Belize	BLZ	Parenteral administration of uterotonics	86.1	73.8
Belize	BLZ	Parenteral administration of antibiotics	72.1	61.8
Belize	BLZ	Assisted vaginal delivery	24.4	20.9
Belize	BLZ	Manual removal of placenta	36.1	31.0
Belize	BLZ	Removal of retained products of conception	31.9	27.4
Belize	BLZ	Cesarean delivery	8.4	7.2
Belize	BLZ	Blood transfusion	12.1	10.4
Belize	BLZ	Induction of labor for pregnancies lasting 41+ weeks	1.7	1.5

Belize	BLZ	Complementary feeding - education only	66.3	56.8
Belize	BLZ	Complementary feeding - supplementary feeding and education	66.3	56.8
Belize	BLZ	Vitamin A supplementation	44.0	37.7
Belize	BLZ	Improved sanitation - Utilization of latrines or toilets	87.9	87.9
Belize	BLZ	Improved water source	98.0	98.0
Belize	BLZ	Water connection in the home	84.1	84.1
Belize	BLZ	Hand washing with soap	90.5	90.5
Belize	BLZ	Hygienic disposal of children's stools	16.4	16.4
Belize	BLZ	Injectable antibiotics for neonatal sepsis	96.4	78.5
Belize	BLZ	ORS - oral rehydration solution	55.2	47.3
Belize	BLZ	Zinc for treatment of diarrhea	9.6	8.2
Belize	BLZ	Oral antibiotics for pneumonia	67.4	57.8
Belize	BLZ	Vitamin A for treatment of measles	44.0	37.7
Belize	BLZ	BCG vaccine	99.0	80.6
Belize	BLZ	Polio vaccine	96.0	78.2
Belize	BLZ	DPT vaccine	96.0	78.2
Belize	BLZ	H. influenzae type b vaccine	96.0	90.3
Belize	BLZ	HepB vaccine	96.0	78.2
Belize	BLZ	Measles vaccine	97.0	79.0
Belize	BLZ	Global wasting (<-2 SD) rate	1.9	2.1
Belize	BLZ	Contraceptive prevalence (CPR)	55.5	50.1
Benin	BEN	TT - Tetanus toxoid vaccination	85.0	69.2
Benin	BEN	IPTp - Intermittent preventive treatment of malaria during pregnancy	33.4	27.2
Benin	BEN	Syphilis detection and treatment	20.9	17.0
Benin	BEN	Iron supplementation in pregnancy	28.6	23.3
Benin	BEN	Hypertensive disorder case management	12.1	9.9
Benin	BEN	Diabetes case management	7.1	5.8
Benin	BEN	Malaria case management	42.7	34.8
Benin	BEN	MgSO4 management of pre-eclampsia	28.5	23.2
Benin	BEN	Thermal protection	77.2	66.2
Benin	BEN	Clean cord care	75.8	65.0
Benin	BEN	Clean birth environment	75.8	65.0
Benin	BEN	Immediate drying and additional stimulation	77.6	66.5
Benin	BEN	Neonatal resuscitation	42.9	36.8

Benin	BEN	Antibiotics for preterm or prolonged PROM	68.9	59.1
Benin	BEN	Parenteral administration of anti-convulsants	44.1	37.8
Benin	BEN	Parenteral administration of uterotonics	75.8	65.0
Benin	BEN	Parenteral administration of antibiotics	68.9	59.1
Benin	BEN	Assisted vaginal delivery	24.0	20.6
Benin	BEN	Manual removal of placenta	64.4	55.2
Benin	BEN	Removal of retained products of conception	49.9	42.8
Benin	BEN	Cesarean delivery	4.3	3.7
Benin	BEN	Blood transfusion	16.4	14.1
Benin	BEN	Induction of labor for pregnancies lasting 41+ weeks	1.5	1.3
Benin	BEN	Complementary feeding - education only	25.3	21.7
Benin	BEN	Complementary feeding - supplementary feeding and education	25.3	21.7
Benin	BEN	Vitamin A supplementation	99.0	84.9
Benin	BEN	Improved sanitation - Utilization of latrines or toilets	16.5	16.5
Benin	BEN	Improved water source	66.4	66.4
Benin	BEN	Water connection in the home	26.4	26.4
Benin	BEN	Hand washing with soap	8.4	8.4
Benin	BEN	Hygienic disposal of children's stools	34.8	34.8
Benin	BEN	ITN/IRS - Households protected from malaria	92.0	78.9
Benin	BEN	Injectable antibiotics for neonatal sepsis	78.1	63.6
Benin	BEN	ORS - oral rehydration solution	22.2	19.0
Benin	BEN	Antibiotics for treatment of dysentery	29.2	25.0
Benin	BEN	Zinc for treatment of diarrhea	17.0	14.6
Benin	BEN	Oral antibiotics for pneumonia	46.1	39.5
Benin	BEN	Vitamin A for treatment of measles	99.0	84.9
Benin	BEN	ACTs- Artemisinin compounds for treatment of malaria	2.5	2.1
Benin	BEN	SAM - treatment for severe acute malnutrition	0.8	0.7
Benin	BEN	BCG vaccine	89.0	72.5
Benin	BEN	Polio vaccine	75.0	61.1
Benin	BEN	DPT vaccine	76.0	61.9
Benin	BEN	H. influenzae type b vaccine	76.0	71.5
Benin	BEN	HepB vaccine	76.0	61.9
Benin	BEN	Pneumococcal vaccine	73.0	68.7

Benin	BEN	Measles vaccine	71.0	57.8
Benin	BEN	Global wasting (<-2 SD) rate	4.5	4.9
Benin	BEN	Contraceptive prevalence (CPR)	17.3	15.6
Bhutan	BTN	Safe abortion services	35.1	31.7
Bhutan	BTN	TT - Tetanus toxoid vaccination	89.0	72.5
Bhutan	BTN	Syphilis detection and treatment	24.0	19.5
Bhutan	BTN	Hypertensive disorder case management	18.6	15.1
Bhutan	BTN	Diabetes case management	14.5	11.8
Bhutan	BTN	Malaria case management	59.9	48.8
Bhutan	BTN	MgSO4 management of pre-eclampsia	36.8	30.0
Bhutan	BTN	Thermal protection	62.4	53.5
Bhutan	BTN	Clean cord care	60.2	51.6
Bhutan	BTN	Clean birth environment	51.8	44.4
Bhutan	BTN	Immediate drying and additional stimulation	57.8	49.6
Bhutan	BTN	Neonatal resuscitation	34.7	29.8
Bhutan	BTN	Antibiotics for preterm or prolonged PROM	47.2	40.5
Bhutan	BTN	Parenteral administration of anti-convulsants	45.1	38.7
Bhutan	BTN	Parenteral administration of uterotonics	56.4	48.4
Bhutan	BTN	Parenteral administration of antibiotics	47.2	40.5
Bhutan	BTN	Assisted vaginal delivery	16.0	13.7
Bhutan	BTN	Manual removal of placenta	23.6	20.2
Bhutan	BTN	Removal of retained products of conception	20.9	17.9
Bhutan	BTN	Cesarean delivery	5.5	4.7
Bhutan	BTN	Blood transfusion	7.9	6.8
Bhutan	BTN	Induction of labor for pregnancies lasting 41+ weeks	1.1	0.9
Bhutan	BTN	Complementary feeding - education only	88.7	76.0
Bhutan	BTN	Complementary feeding - supplementary feeding and education	88.7	76.0
Bhutan	BTN	Vitamin A supplementation	45.0	38.6
Bhutan	BTN	Improved sanitation - Utilization of latrines or toilets	69.3	69.3
Bhutan	BTN	Improved water source	97.2	97.2
Bhutan	BTN	Water connection in the home	96.3	96.3
Bhutan	BTN	Hand washing with soap	79.4	79.4
Bhutan	BTN	Hygienic disposal of children's stools	57.5	57.5
Bhutan	BTN	Injectable antibiotics for neonatal sepsis	63.1	51.4

Bhutan	BTN	ORS - oral rehydration solution	60.9	52.2
Bhutan	BTN	Zinc for treatment of diarrhea	0.8	0.7
Bhutan	BTN	Oral antibiotics for pneumonia	74.2	63.6
Bhutan	BTN	Vitamin A for treatment of measles	45.0	38.6
Bhutan	BTN	BCG vaccine	99.0	80.6
Bhutan	BTN	Polio vaccine	97.0	79.0
Bhutan	BTN	DPT vaccine	97.0	79.0
Bhutan	BTN	H. influenzae type b vaccine	97.0	91.2
Bhutan	BTN	HepB vaccine	97.0	79.0
Bhutan	BTN	Measles vaccine	97.0	79.0
Bhutan	BTN	Global wasting (<-2 SD) rate	6.1	6.7
Bhutan	BTN	Contraceptive prevalence (CPR)	68.35	61.7
Bolivia	BOL	Safe abortion services	0.2	0.2
Bolivia	BOL	TT - Tetanus toxoid vaccination	87.0	70.9
Bolivia	BOL	Syphilis detection and treatment	22.3	18.2
Bolivia	BOL	Iron supplementation in pregnancy	25.1	20.4
Bolivia	BOL	Hypertensive disorder case management	17.2	14.0
Bolivia	BOL	Diabetes case management	13.4	10.9
Bolivia	BOL	Malaria case management	55.4	45.1
Bolivia	BOL	MgSO4 management of pre-eclampsia	34.0	27.7
Bolivia	BOL	Thermal protection	66.7	57.2
Bolivia	BOL	Clean cord care	64.4	55.2
Bolivia	BOL	Clean birth environment	55.4	47.5
Bolivia	BOL	Immediate drying and additional stimulation	61.8	53.0
Bolivia	BOL	Neonatal resuscitation	37.1	31.8
Bolivia	BOL	Antibiotics for preterm or prolonged PROM	50.5	43.3
Bolivia	BOL	Parenteral administration of anti-convulsants	48.3	41.4
Bolivia	BOL	Parenteral administration of uterotonics	60.3	51.7
Bolivia	BOL	Parenteral administration of antibiotics	50.5	43.3
Bolivia	BOL	Assisted vaginal delivery	17.1	14.7
Bolivia	BOL	Manual removal of placenta	25.2	21.6
Bolivia	BOL	Removal of retained products of conception	22.4	19.2
Bolivia	BOL	Cesarean delivery	5.9	5.1
Bolivia	BOL	Blood transfusion	8.5	7.3
Bolivia	BOL	Induction of labor for pregnancies lasting 41+ weeks	1.2	1.0

Bolivia	BOL	Complementary feeding - education only	70.8	60.7
Bolivia	BOL	Complementary feeding - supplementary feeding and education	70.8	60.7
Bolivia	BOL	Vitamin A supplementation	31.0	26.6
Bolivia	BOL	Improved sanitation - Utilization of latrines or toilets	60.7	60.7
Bolivia	BOL	Improved water source	92.8	92.8
Bolivia	BOL	Water connection in the home	65.9	65.9
Bolivia	BOL	Hygienic disposal of children's stools	16.6	16.6
Bolivia	BOL	Injectable antibiotics for neonatal sepsis	67.5	55.0
Bolivia	BOL	ORS - oral rehydration solution	34.9	29.9
Bolivia	BOL	Antibiotics for treatment of dysentery	43.9	37.6
Bolivia	BOL	Vitamin A for treatment of measles	31.0	26.6
Bolivia	BOL	BCG vaccine	90.0	73.3
Bolivia	BOL	Polio vaccine	83.0	67.6
Bolivia	BOL	DPT vaccine	83.0	67.6
Bolivia	BOL	H. influenzae type b vaccine	83.0	78.1
Bolivia	BOL	HepB vaccine	83.0	67.6
Bolivia	BOL	Pneumococcal vaccine	83.0	78.1
Bolivia	BOL	Rotavirus vaccine	87.0	81.8
Bolivia	BOL	Measles vaccine	89.0	72.5
Bolivia	BOL	Global wasting (<-2 SD) rate	1.4	1.6
Bolivia	BOL	Contraceptive prevalence (CPR)	66.1	59.7
Bosnia and Herzegovina	BIH	Safe abortion services	100.0	90.3
Bosnia and Herzegovina	BIH	Syphilis detection and treatment	21.5	17.5
Bosnia and Herzegovina	BIH	Hypertensive disorder case management	20.2	16.5
Bosnia and Herzegovina	BIH	Diabetes case management	15.8	12.9
Bosnia and Herzegovina	BIH	Malaria case management	65.3	53.2
Bosnia and Herzegovina	BIH	MgSO4 management of pre-eclampsia	40.0	32.6
Bosnia and Herzegovina	BIH	Thermal protection	98.6	84.5
Bosnia and Herzegovina	BIH	Clean cord care	95.2	81.6

Bosnia and Herzegovina	BIH	Clean birth environment	81.8	70.1
Bosnia and Herzegovina	BIH	Immediate drying and additional stimulation	91.3	78.3
Bosnia and Herzegovina	BIH	Neonatal resuscitation	54.8	47.0
Bosnia and Herzegovina	BIH	Antibiotics for preterm or prolonged PROM	74.6	64.0
Bosnia and Herzegovina	BIH	Parenteral administration of anti-convulsants	71.3	61.1
Bosnia and Herzegovina	BIH	Parenteral administration of uterotonics	89.1	76.4
Bosnia and Herzegovina	BIH	Parenteral administration of antibiotics	74.6	64.0
Bosnia and Herzegovina	BIH	Assisted vaginal delivery	25.2	21.6
Bosnia and Herzegovina	BIH	Manual removal of placenta	37.3	32.0
Bosnia and Herzegovina	BIH	Removal of retained products of conception	33.0	28.3
Bosnia and Herzegovina	BIH	Cesarean delivery	8.7	7.5
Bosnia and Herzegovina	BIH	Blood transfusion	12.6	10.8
Bosnia and Herzegovina	BIH	Induction of labor for pregnancies lasting 41+ weeks	1.8	1.5
Bosnia and Herzegovina	BIH	Improved sanitation - Utilization of latrines or toilets	95.4	95.4
Bosnia and Herzegovina	BIH	Improved water source	96.1	96.1
Bosnia and Herzegovina	BIH	Water connection in the home	91.5	91.5
Bosnia and Herzegovina	BIH	Hand washing with soap	97.0	97.0
Bosnia and Herzegovina	BIH	Hygienic disposal of children's stools	19.6	19.6
Bosnia and Herzegovina	BIH	Injectable antibiotics for neonatal sepsis	99.7	81.2
Bosnia and Herzegovina	BIH	ORS - oral rehydration solution	36.3	31.1
Bosnia and Herzegovina	BIH	Antibiotics for treatment of dysentery	2.9	2.5

Bosnia and Herzegovina	BIH	Oral antibiotics for pneumonia	86.9	74.5
Bosnia and Herzegovina	BIH	BCG vaccine	95.0	77.4
Bosnia and Herzegovina	BIH	Polio vaccine	73.0	59.5
Bosnia and Herzegovina	BIH	DPT vaccine	73.0	59.5
Bosnia and Herzegovina	BIH	H. influenzae type b vaccine	62.0	58.3
Bosnia and Herzegovina	BIH	HepB vaccine	80.0	65.2
Bosnia and Herzegovina	BIH	Measles vaccine	68.0	55.4
Bosnia and Herzegovina	BIH	Global wasting (<-2 SD) rate	2.4	2.6
Bosnia and Herzegovina	BIH	Contraceptive prevalence (CPR)	48.6	43.9
Botswana	BWA	Safe abortion services	42.4	38.3
Botswana	BWA	TT - Tetanus toxoid vaccination	93.0	75.7
Botswana	BWA	Syphilis detection and treatment	18.1	14.7
Botswana	BWA	Hypertensive disorder case management	17.6	14.3
Botswana	BWA	Diabetes case management	13.7	11.2
Botswana	BWA	Malaria case management	56.8	46.3
Botswana	BWA	MgSO4 management of pre-eclampsia	34.8	28.3
Botswana	BWA	Thermal protection	92.7	79.5
Botswana	BWA	Clean cord care	89.5	76.7
Botswana	BWA	Clean birth environment	76.9	65.9
Botswana	BWA	Immediate drying and additional stimulation	85.9	73.6
Botswana	BWA	Neonatal resuscitation	51.6	44.2
Botswana	BWA	Antibiotics for preterm or prolonged PROM	70.2	60.2
Botswana	BWA	Parenteral administration of anti-convulsants	67.1	57.5
Botswana	BWA	Parenteral administration of uterotonics	83.8	71.8
Botswana	BWA	Parenteral administration of antibiotics	70.2	60.2
Botswana	BWA	Assisted vaginal delivery	23.7	20.3
Botswana	BWA	Manual removal of placenta	35.1	30.1
Botswana	BWA	Removal of retained products of conception	31.1	26.7
Botswana	BWA	Cesarean delivery	8.2	7.0
Botswana	BWA	Blood transfusion	11.8	10.1

Botswana	BWA	Induction of labor for pregnancies lasting 41+ weeks	1.7	1.5
Botswana	BWA	Complementary feeding - education only	45.5	39.0
Botswana	BWA	Complementary feeding - supplementary feeding and education	45.5	39.0
Botswana	BWA	Vitamin A supplementation	83.0	71.2
Botswana	BWA	Improved sanitation - Utilization of latrines or toilets	77.3	77.3
Botswana	BWA	Improved water source	90.3	90.3
Botswana	BWA	Water connection in the home	81.7	81.7
Botswana	BWA	Injectable antibiotics for neonatal sepsis	93.8	76.4
Botswana	BWA	ORS - oral rehydration solution	42.9	36.8
Botswana	BWA	Oral antibiotics for pneumonia	14.0	12.0
Botswana	BWA	Vitamin A for treatment of measles	83.0	71.2
Botswana	BWA	BCG vaccine	98.0	79.8
Botswana	BWA	Polio vaccine	96.0	78.2
Botswana	BWA	DPT vaccine	95.0	77.4
Botswana	BWA	H. influenzae type b vaccine	95.0	89.4
Botswana	BWA	HepB vaccine	95.0	77.4
Botswana	BWA	Pneumococcal vaccine	91.0	85.6
Botswana	BWA	Rotavirus vaccine	87.0	81.8
Botswana	BWA	Measles vaccine	97.0	79.0
Botswana	BWA	Global wasting (<-2 SD) rate	4.9	5.4
Botswana	BWA	Contraceptive prevalence (CPR)	61.45	55.5
Brazil	BRA	Safe abortion services	0.2	0.2
Brazil	BRA	TT - Tetanus toxoid vaccination	94.0	76.6
Brazil	BRA	Syphilis detection and treatment	22.5	18.3
Brazil	BRA	Hypertensive disorder case management	21.8	17.8
Brazil	BRA	Diabetes case management	17.0	13.8
Brazil	BRA	Malaria case management	70.5	57.4
Brazil	BRA	MgSO4 management of pre-eclampsia	43.2	35.2
Brazil	BRA	Thermal protection	97.0	83.2
Brazil	BRA	Clean cord care	93.6	80.3
Brazil	BRA	Clean birth environment	80.5	69.0
Brazil	BRA	Immediate drying and additional stimulation	89.8	77.0
Brazil	BRA	Neonatal resuscitation	53.9	46.2
Brazil	BRA	Antibiotics for preterm or prolonged PROM	73.4	62.9

Brazil	BRA	Parenteral administration of anti-convulsants	70.1	60.1
Brazil	BRA	Parenteral administration of uterotonics	87.7	75.2
Brazil	BRA	Parenteral administration of antibiotics	73.4	62.9
Brazil	BRA	Assisted vaginal delivery	24.8	21.3
Brazil	BRA	Manual removal of placenta	36.7	31.5
Brazil	BRA	Removal of retained products of conception	32.5	27.9
Brazil	BRA	Cesarean delivery	8.5	7.3
Brazil	BRA	Blood transfusion	12.4	10.6
Brazil	BRA	Induction of labor for pregnancies lasting 41+ weeks	1.7	1.5
Brazil	BRA	Complementary feeding - education only	56.7	48.6
Brazil	BRA	Complementary feeding - supplementary feeding and education	56.7	48.6
Brazil	BRA	Improved sanitation - Utilization of latrines or toilets	88.3	88.3
Brazil	BRA	Improved water source	98.2	98.2
Brazil	BRA	Water connection in the home	95.5	95.5
Brazil	BRA	Injectable antibiotics for neonatal sepsis	98.1	79.9
Brazil	BRA	ORS - oral rehydration solution	43.6	37.4
Brazil	BRA	Antibiotics for treatment of dysentery	7.9	6.8
Brazil	BRA	Oral antibiotics for pneumonia	49.7	42.6
Brazil	BRA	BCG vaccine	90.0	73.3
Brazil	BRA	Polio vaccine	85.0	69.2
Brazil	BRA	DPT vaccine	83.0	67.6
Brazil	BRA	H. influenzae type b vaccine	83.0	78.1
Brazil	BRA	HepB vaccine	83.0	67.6
Brazil	BRA	Pneumococcal vaccine	84.0	79.0
Brazil	BRA	Rotavirus vaccine	80.0	75.3
Brazil	BRA	Meningococcal A	84.0	68.4
Brazil	BRA	Measles vaccine	84.0	68.4
Brazil	BRA	Global wasting (<-2 SD) rate	2.7	2.9
Brazil	BRA	Contraceptive prevalence (CPR)	79.9	72.1
Burkina Faso	BFA	TT - Tetanus toxoid vaccination	92.0	74.9
Burkina Faso	BFA	IPTp - Intermittent preventive treatment of malaria during pregnancy	47.6	38.8
Burkina Faso	BFA	Syphilis detection and treatment	8.4	6.8
Burkina Faso	BFA	Iron supplementation in pregnancy	50.2	40.9

Burkina Faso	BFA	Hypertensive disorder case management	6.4	5.2
Burkina Faso	BFA	Diabetes case management	0.7	0.6
Burkina Faso	BFA	Malaria case management	22.6	18.4
Burkina Faso	BFA	MgSO4 management of pre-eclampsia	6.9	5.6
Burkina Faso	BFA	Thermal protection	65.6	56.2
Burkina Faso	BFA	Clean cord care	65.5	56.2
Burkina Faso	BFA	Clean birth environment	64.0	54.9
Burkina Faso	BFA	Immediate drying and additional stimulation	63.9	54.8
Burkina Faso	BFA	Neonatal resuscitation	13.0	11.1
Burkina Faso	BFA	Antibiotics for preterm or prolonged PROM	52.6	45.1
Burkina Faso	BFA	Parenteral administration of anti-convulsants	15.9	13.6
Burkina Faso	BFA	Parenteral administration of uterotonics	64.1	55.0
Burkina Faso	BFA	Parenteral administration of antibiotics	52.6	45.1
Burkina Faso	BFA	Assisted vaginal delivery	7.0	6.0
Burkina Faso	BFA	Manual removal of placenta	49.1	42.1
Burkina Faso	BFA	Removal of retained products of conception	11.1	9.5
Burkina Faso	BFA	Cesarean delivery	0.8	0.7
Burkina Faso	BFA	Blood transfusion	5.8	5.0
Burkina Faso	BFA	Induction of labor for pregnancies lasting 41+ weeks	0.9	0.8
Burkina Faso	BFA	Complementary feeding - education only	5.9	5.1
Burkina Faso	BFA	Complementary feeding - supplementary feeding and education	5.9	5.1
Burkina Faso	BFA	Improved sanitation - Utilization of latrines or toilets	19.4	19.4
Burkina Faso	BFA	Improved water source	47.9	47.9
Burkina Faso	BFA	Water connection in the home	12.2	12.2
Burkina Faso	BFA	Hand washing with soap	12.8	12.8
Burkina Faso	BFA	Hygienic disposal of children's stools	20.8	20.8
Burkina Faso	BFA	ITN/IRS - Households protected from malaria	75.3	64.6
Burkina Faso	BFA	Injectable antibiotics for neonatal sepsis	66.3	54.0
Burkina Faso	BFA	ORS - oral rehydration solution	21.2	18.2
Burkina Faso	BFA	Antibiotics for treatment of dysentery	34.5	29.6
Burkina Faso	BFA	Zinc for treatment of diarrhea	0.4	0.3
Burkina Faso	BFA	Oral antibiotics for pneumonia	55.5	47.6
Burkina Faso	BFA	ACTs- Artemisinin compounds for treatment of malaria	5.5	4.7

Burkina Faso	BFA	SAM - treatment for severe acute malnutrition	26.1	22.4
Burkina Faso	BFA	BCG vaccine	98.0	79.8
Burkina Faso	BFA	Polio vaccine	91.0	74.1
Burkina Faso	BFA	DPT vaccine	91.0	74.1
Burkina Faso	BFA	H. influenzae type b vaccine	91.0	85.6
Burkina Faso	BFA	HepB vaccine	91.0	74.1
Burkina Faso	BFA	Pneumococcal vaccine	91.0	85.6
Burkina Faso	BFA	Rotavirus vaccine	91.0	85.6
Burkina Faso	BFA	Meningococcal A	87.0	70.9
Burkina Faso	BFA	Measles vaccine	88.0	71.7
Burkina Faso	BFA	Global wasting (<-2 SD) rate	15.5	17.1
Burkina Faso	BFA	Contraceptive prevalence (CPR)	27.95	25.2
Burundi	BDI	Safe abortion services	3.3	3.0
Burundi	BDI	TT - Tetanus toxoid vaccination	87.0	70.9
Burundi	BDI	IPTp - Intermittent preventive treatment of malaria during pregnancy	20.7	16.9
Burundi	BDI	Syphilis detection and treatment	24.5	20.0
Burundi	BDI	Iron supplementation in pregnancy	1.4	1.1
Burundi	BDI	Hypertensive disorder case management	12.2	9.9
Burundi	BDI	Diabetes case management	9.5	7.7
Burundi	BDI	Malaria case management	39.3	32.0
Burundi	BDI	MgSO4 management of pre-eclampsia	24.1	19.6
Burundi	BDI	Thermal protection	83.0	71.2
Burundi	BDI	Clean cord care	80.1	68.7
Burundi	BDI	Clean birth environment	68.8	59.0
Burundi	BDI	Immediate drying and additional stimulation	76.9	65.9
Burundi	BDI	Neonatal resuscitation	46.2	39.6
Burundi	BDI	Antibiotics for preterm or prolonged PROM	62.8	53.8
Burundi	BDI	Parenteral administration of anti-convulsants	60.0	51.4
Burundi	BDI	Parenteral administration of uterotonics	75.0	64.3
Burundi	BDI	Parenteral administration of antibiotics	62.8	53.8
Burundi	BDI	Assisted vaginal delivery	21.2	18.2
Burundi	BDI	Manual removal of placenta	31.4	26.9
Burundi	BDI	Removal of retained products of conception	27.8	23.8
Burundi	BDI	Cesarean delivery	7.3	6.3
Burundi	BDI	Blood transfusion	10.6	9.1

Burundi	BDI	Induction of labor for pregnancies lasting 41+ weeks	1.5	1.3
Burundi	BDI	Complementary feeding - education only	19.3	16.5
Burundi	BDI	Complementary feeding - supplementary feeding and education	19.3	16.5
Burundi	BDI	Vitamin A supplementation	79.0	67.7
Burundi	BDI	Improved sanitation - Utilization of latrines or toilets	45.8	45.8
Burundi	BDI	Improved water source	60.8	60.8
Burundi	BDI	Water connection in the home	21.6	21.6
Burundi	BDI	Hand washing with soap	5.8	5.8
Burundi	BDI	Hygienic disposal of children's stools	73.7	73.7
Burundi	BDI	ITN/IRS - Households protected from malaria	46.8	40.1
Burundi	BDI	Injectable antibiotics for neonatal sepsis	83.9	68.3
Burundi	BDI	ORS - oral rehydration solution	35.6	30.5
Burundi	BDI	Antibiotics for treatment of dysentery	32.9	28.2
Burundi	BDI	Zinc for treatment of diarrhea	15.0	12.9
Burundi	BDI	Oral antibiotics for pneumonia	58.5	50.2
Burundi	BDI	Vitamin A for treatment of measles	79.0	67.7
Burundi	BDI	ACTs- Artemisinin compounds for treatment of malaria	5.0	4.3
Burundi	BDI	SAM - treatment for severe acute malnutrition	6.9	5.9
Burundi	BDI	BCG vaccine	91.0	74.1
Burundi	BDI	Polio vaccine	90.0	73.3
Burundi	BDI	DPT vaccine	90.0	73.3
Burundi	BDI	H. influenzae type b vaccine	90.0	84.7
Burundi	BDI	HepB vaccine	90.0	73.3
Burundi	BDI	Pneumococcal vaccine	90.0	84.7
Burundi	BDI	Rotavirus vaccine	92.0	86.5
Burundi	BDI	Measles vaccine	88.0	71.7
Burundi	BDI	Global wasting (<-2 SD) rate	5.0	5.5
Burundi	BDI	Contraceptive prevalence (CPR)	31.85	28.7
Cambodia	KHM	Safe abortion services	39.0	35.2
Cambodia	KHM	TT - Tetanus toxoid vaccination	93.0	75.7
Cambodia	KHM	Syphilis detection and treatment	23.7	19.3
Cambodia	KHM	Iron supplementation in pregnancy	75.5	61.5
Cambodia	KHM	Hypertensive disorder case management	18.2	14.8

Cambodia	KHM	Diabetes case management	14.2	11.6
Cambodia	KHM	Malaria case management	58.7	47.8
Cambodia	KHM	MgSO4 management of pre-eclampsia	36.0	29.3
Cambodia	KHM	Thermal protection	82.3	70.6
Cambodia	KHM	Clean cord care	79.4	68.1
Cambodia	KHM	Clean birth environment	68.3	58.6
Cambodia	KHM	Immediate drying and additional stimulation	76.2	65.3
Cambodia	KHM	Neonatal resuscitation	45.8	39.3
Cambodia	KHM	Antibiotics for preterm or prolonged PROM	62.3	53.4
Cambodia	KHM	Parenteral administration of anti-convulsants	59.5	51.0
Cambodia	KHM	Parenteral administration of uterotonics	74.4	63.8
Cambodia	KHM	Parenteral administration of antibiotics	62.3	53.4
Cambodia	KHM	Assisted vaginal delivery	21.0	18.0
Cambodia	KHM	Manual removal of placenta	31.1	26.7
Cambodia	KHM	Removal of retained products of conception	27.6	23.7
Cambodia	KHM	Cesarean delivery	7.2	6.2
Cambodia	KHM	Blood transfusion	10.5	9.0
Cambodia	KHM	Induction of labor for pregnancies lasting 41+ weeks	1.5	1.3
Cambodia	KHM	Complementary feeding - education only	47.7	40.9
Cambodia	KHM	Complementary feeding - supplementary feeding and education	47.7	40.9
Cambodia	KHM	Vitamin A supplementation	73.0	62.6
Cambodia	KHM	Improved sanitation - Utilization of latrines or toilets	59.2	59.2
Cambodia	KHM	Improved water source	78.5	78.5
Cambodia	KHM	Water connection in the home	20.5	20.5
Cambodia	KHM	Hand washing with soap	67.9	67.9
Cambodia	KHM	Hygienic disposal of children's stools	22.6	22.6
Cambodia	KHM	ITN/IRS - Households protected from malaria	4.5	3.9
Cambodia	KHM	Injectable antibiotics for neonatal sepsis	83.2	67.8
Cambodia	KHM	ORS - oral rehydration solution	35.2	30.2
Cambodia	KHM	Antibiotics for treatment of dysentery	7.0	6.0
Cambodia	KHM	Zinc for treatment of diarrhea	5.4	4.6
Cambodia	KHM	Oral antibiotics for pneumonia	68.8	59.0
Cambodia	KHM	Vitamin A for treatment of measles	73.0	62.6
Cambodia	KHM	SAM - treatment for severe acute malnutrition	2.5	2.1

Cambodia	KHM	BCG vaccine	93.0	75.7
Cambodia	KHM	Polio vaccine	90.0	73.3
Cambodia	KHM	DPT vaccine	92.0	74.9
Cambodia	KHM	H. influenzae type b vaccine	92.0	86.5
Cambodia	KHM	HepB vaccine	92.0	74.9
Cambodia	KHM	Pneumococcal vaccine	84.0	79.0
Cambodia	KHM	Measles vaccine	84.0	68.4
Cambodia	KHM	Global wasting (<-2 SD) rate	9.9	10.8
Cambodia	KHM	Contraceptive prevalence (CPR)	61.2	55.2
Cameroon	CMR	TT - Tetanus toxoid vaccination	85.0	69.2
Cameroon	CMR	IPTp - Intermittent preventive treatment of malaria during pregnancy	44.3	36.1
Cameroon	CMR	Syphilis detection and treatment	20.5	16.7
Cameroon	CMR	Iron supplementation in pregnancy	54.1	44.1
Cameroon	CMR	Hypertensive disorder case management	14.1	11.5
Cameroon	CMR	Diabetes case management	11.0	9.0
Cameroon	CMR	Malaria case management	45.6	37.1
Cameroon	CMR	MgSO4 management of pre-eclampsia	28.0	22.8
Cameroon	CMR	Thermal protection	60.5	51.9
Cameroon	CMR	Clean cord care	58.5	50.2
Cameroon	CMR	Clean birth environment	50.2	43.0
Cameroon	CMR	Immediate drying and additional stimulation	56.1	48.1
Cameroon	CMR	Neonatal resuscitation	33.7	28.9
Cameroon	CMR	Antibiotics for preterm or prolonged PROM	45.8	39.3
Cameroon	CMR	Parenteral administration of anti-convulsants	43.8	37.6
Cameroon	CMR	Parenteral administration of uterotonics	54.7	46.9
Cameroon	CMR	Parenteral administration of antibiotics	45.8	39.3
Cameroon	CMR	Assisted vaginal delivery	15.5	13.3
Cameroon	CMR	Manual removal of placenta	22.9	19.6
Cameroon	CMR	Removal of retained products of conception	20.3	17.4
Cameroon	CMR	Cesarean delivery	5.3	4.5
Cameroon	CMR	Blood transfusion	7.7	6.6
Cameroon	CMR	Induction of labor for pregnancies lasting 41+ weeks	1.1	0.9
Cameroon	CMR	Complementary feeding - education only	32.9	28.2
Cameroon	CMR	Complementary feeding - supplementary feeding and education	32.9	28.2

Cameroon	CMR	Vitamin A supplementation	9.0	7.7
Cameroon	CMR	Improved sanitation - Utilization of latrines or toilets	39.1	39.1
Cameroon	CMR	Improved water source	60.4	60.4
Cameroon	CMR	Water connection in the home	24.0	24.0
Cameroon	CMR	Hand washing with soap	14.9	14.9
Cameroon	CMR	Hygienic disposal of children's stools	70.6	70.6
Cameroon	CMR	ITN/IRS - Households protected from malaria	70.9	60.8
Cameroon	CMR	Injectable antibiotics for neonatal sepsis	61.3	49.9
Cameroon	CMR	ORS - oral rehydration solution	15.8	13.5
Cameroon	CMR	Antibiotics for treatment of dysentery	15.0	12.9
Cameroon	CMR	Zinc for treatment of diarrhea	12.0	10.3
Cameroon	CMR	Oral antibiotics for pneumonia	26.7	22.9
Cameroon	CMR	Vitamin A for treatment of measles	9.0	7.7
Cameroon	CMR	ACTs- Artemisinin compounds for treatment of malaria	3.6	3.1
Cameroon	CMR	SAM - treatment for severe acute malnutrition	11.3	9.7
Cameroon	CMR	BCG vaccine	88.0	71.7
Cameroon	CMR	Polio vaccine	78.0	63.5
Cameroon	CMR	DPT vaccine	79.0	64.3
Cameroon	CMR	H. influenzae type b vaccine	79.0	74.3
Cameroon	CMR	HepB vaccine	79.0	64.3
Cameroon	CMR	Pneumococcal vaccine	79.0	74.3
Cameroon	CMR	Rotavirus vaccine	78.0	73.4
Cameroon	CMR	Measles vaccine	71.0	57.8
Cameroon	CMR	Global wasting (<-2 SD) rate	5.0	5.5
Cameroon	CMR	Contraceptive prevalence (CPR)	37.95	34.2
Cape Verde	CPV	TT - Tetanus toxoid vaccination	92.0	74.9
Cape Verde	CPV	Syphilis detection and treatment	22.3	18.2
Cape Verde	CPV	Iron supplementation in pregnancy	10.2	8.3
Cape Verde	CPV	Hypertensive disorder case management	17.4	14.2
Cape Verde	CPV	Diabetes case management	13.5	11.0
Cape Verde	CPV	Malaria case management	56.0	45.6
Cape Verde	CPV	MgSO4 management of pre-eclampsia	34.4	28.0
Cape Verde	CPV	Thermal protection	76.6	65.7
Cape Verde	CPV	Clean cord care	74.0	63.4

Cape Verde	CPV	Clean birth environment	63.6	54.5
Cape Verde	CPV	Immediate drying and additional stimulation	71.0	60.9
Cape Verde	CPV	Neonatal resuscitation	42.6	36.5
Cape Verde	CPV	Antibiotics for preterm or prolonged PROM	58.0	49.7
Cape Verde	CPV	Parenteral administration of anti-convulsants	55.4	47.5
Cape Verde	CPV	Parenteral administration of uterotonics	69.3	59.4
Cape Verde	CPV	Parenteral administration of antibiotics	58.0	49.7
Cape Verde	CPV	Assisted vaginal delivery	19.6	16.8
Cape Verde	CPV	Manual removal of placenta	29.0	24.9
Cape Verde	CPV	Removal of retained products of conception	25.7	22.0
Cape Verde	CPV	Cesarean delivery	6.7	5.7
Cape Verde	CPV	Blood transfusion	9.8	8.4
Cape Verde	CPV	Induction of labor for pregnancies lasting 41+ weeks	1.4	1.2
Cape Verde	CPV	Improved sanitation - Utilization of latrines or toilets	73.9	73.9
Cape Verde	CPV	Improved water source	87.1	87.1
Cape Verde	CPV	Water connection in the home	77.1	77.1
Cape Verde	CPV	Injectable antibiotics for neonatal sepsis	77.5	63.1
Cape Verde	CPV	ORS - oral rehydration solution	99.8	85.6
Cape Verde	CPV	BCG vaccine	96.0	78.2
Cape Verde	CPV	Polio vaccine	98.0	79.8
Cape Verde	CPV	DPT vaccine	98.0	79.8
Cape Verde	CPV	H. influenzae type b vaccine	99.0	93.1
Cape Verde	CPV	HepB vaccine	99.0	80.6
Cape Verde	CPV	Measles vaccine	99.0	80.6
Cape Verde	CPV	Global wasting (<-2 SD) rate	10.0	11.0
Cape Verde	CPV	Contraceptive prevalence (CPR)	66.5	60.0
Central African Republic	CAF	TT - Tetanus toxoid vaccination	60.0	48.9
Central African Republic	CAF	IPTp - Intermittent preventive treatment of malaria during pregnancy	26.4	21.5
Central African Republic	CAF	Syphilis detection and treatment	16.9	13.8
Central African Republic	CAF	Hypertensive disorder case management	9.2	7.5
Central African Republic	CAF	Diabetes case management	7.1	5.8

Central African Republic	CAF	Malaria case management	29.6	24.1
Central African Republic	CAF	MgSO4 management of pre-eclampsia	18.1	14.7
Central African Republic	CAF	Thermal protection	51.5	44.2
Central African Republic	CAF	Clean cord care	49.7	42.6
Central African Republic	CAF	Clean birth environment	42.7	36.6
Central African Republic	CAF	Immediate drying and additional stimulation	47.7	40.9
Central African Republic	CAF	Neonatal resuscitation	28.6	24.5
Central African Republic	CAF	Antibiotics for preterm or prolonged PROM	39.0	33.4
Central African Republic	CAF	Parenteral administration of anti-convulsants	37.2	31.9
Central African Republic	CAF	Parenteral administration of uterotonics	46.5	39.9
Central African Republic	CAF	Parenteral administration of antibiotics	39.0	33.4
Central African Republic	CAF	Assisted vaginal delivery	13.2	11.3
Central African Republic	CAF	Manual removal of placenta	19.5	16.7
Central African Republic	CAF	Removal of retained products of conception	17.3	14.8
Central African Republic	CAF	Cesarean delivery	4.5	3.9
Central African Republic	CAF	Blood transfusion	6.6	5.7
Central African Republic	CAF	Induction of labor for pregnancies lasting 41+ weeks	0.9	0.8
Central African Republic	CAF	Complementary feeding - education only	33.9	29.1
Central African Republic	CAF	Complementary feeding - supplementary feeding and education	33.9	29.1
Central African Republic	CAF	Improved sanitation - Utilization of latrines or toilets	25.3	25.3
Central African Republic	CAF	Improved water source	46.3	46.3

Central African Republic	CAF	Water connection in the home	8.4	8.4
Central African Republic	CAF	Hand washing with soap	15.4	15.4
Central African Republic	CAF	Hygienic disposal of children's stools	50.2	50.2
Central African Republic	CAF	ITN/IRS - Households protected from malaria	47.2	40.5
Central African Republic	CAF	Injectable antibiotics for neonatal sepsis	52.1	42.4
Central African Republic	CAF	ORS - oral rehydration solution	15.6	13.4
Central African Republic	CAF	Antibiotics for treatment of dysentery	4.4	3.8
Central African Republic	CAF	Zinc for treatment of diarrhea	0.5	0.4
Central African Republic	CAF	Oral antibiotics for pneumonia	29.8	25.5
Central African Republic	CAF	ACTs- Artemisinin compounds for treatment of malaria	2.3	2.0
Central African Republic	CAF	SAM - treatment for severe acute malnutrition	17.6	15.1
Central African Republic	CAF	BCG vaccine	74.0	60.3
Central African Republic	CAF	Polio vaccine	47.0	38.3
Central African Republic	CAF	DPT vaccine	47.0	38.3
Central African Republic	CAF	H. influenzae type b vaccine	47.0	44.2
Central African Republic	CAF	HepB vaccine	47.0	38.3
Central African Republic	CAF	Pneumococcal vaccine	47.0	44.2
Central African Republic	CAF	Meningococcal A	66.0	53.8
Central African Republic	CAF	Measles vaccine	49.0	39.9
Central African Republic	CAF	Global wasting (<-2 SD) rate	7.3	8.0
Central African Republic	CAF	Contraceptive prevalence (CPR)	23.5	21.2
Chad	TCD	TT - Tetanus toxoid vaccination	78.0	63.5

Chad	TCD	IPTp - Intermittent preventive treatment of malaria during pregnancy	18.1	14.7
Chad	TCD	Syphilis detection and treatment	13.7	11.2
Chad	TCD	Iron supplementation in pregnancy	11.0	9.0
Chad	TCD	Hypertensive disorder case management	7.5	6.1
Chad	TCD	Diabetes case management	5.9	4.8
Chad	TCD	Malaria case management	24.3	19.8
Chad	TCD	MgSO4 management of pre-eclampsia	14.9	12.1
Chad	TCD	Thermal protection	21.6	18.5
Chad	TCD	Clean cord care	20.9	17.9
Chad	TCD	Clean birth environment	17.9	15.3
Chad	TCD	Immediate drying and additional stimulation	20.0	17.1
Chad	TCD	Neonatal resuscitation	12.0	10.3
Chad	TCD	Antibiotics for preterm or prolonged PROM	16.4	14.1
Chad	TCD	Parenteral administration of anti-convulsants	15.6	13.4
Chad	TCD	Parenteral administration of uterotonics	19.5	16.7
Chad	TCD	Parenteral administration of antibiotics	16.4	14.1
Chad	TCD	Assisted vaginal delivery	5.5	4.7
Chad	TCD	Manual removal of placenta	8.2	7.0
Chad	TCD	Removal of retained products of conception	7.2	6.2
Chad	TCD	Cesarean delivery	1.9	1.6
Chad	TCD	Blood transfusion	2.8	2.4
Chad	TCD	Induction of labor for pregnancies lasting 41+ weeks	0.4	0.3
Chad	TCD	Complementary feeding - education only	10.4	8.9
Chad	TCD	Complementary feeding - supplementary feeding and education	10.4	8.9
Chad	TCD	Vitamin A supplementation	67.0	57.4
Chad	TCD	Improved sanitation - Utilization of latrines or toilets	8.3	8.3
Chad	TCD	Improved water source	38.7	38.7
Chad	TCD	Water connection in the home	7.4	7.4
Chad	TCD	Hand washing with soap	23.7	23.7
Chad	TCD	Hygienic disposal of children's stools	18.9	18.9
Chad	TCD	ITN/IRS - Households protected from malaria	77.3	66.3
Chad	TCD	Injectable antibiotics for neonatal sepsis	21.9	17.8
Chad	TCD	ORS - oral rehydration solution	20.4	17.5

Chad	TCD	Antibiotics for treatment of dysentery	15.9	13.6
Chad	TCD	Zinc for treatment of diarrhea	1.3	1.1
Chad	TCD	Oral antibiotics for pneumonia	25.8	22.1
Chad	TCD	Vitamin A for treatment of measles	67.0	57.4
Chad	TCD	ACTs- Artemisinin compounds for treatment of malaria	1.7	1.5
Chad	TCD	SAM - treatment for severe acute malnutrition	18.3	15.7
Chad	TCD	BCG vaccine	59.0	48.1
Chad	TCD	Polio vaccine	44.0	35.8
Chad	TCD	DPT vaccine	41.0	33.4
Chad	TCD	H. influenzae type b vaccine	41.0	38.6
Chad	TCD	HepB vaccine	41.0	33.4
Chad	TCD	Meningococcal A	70.0	57.0
Chad	TCD	Measles vaccine	37.0	30.1
Chad	TCD	Global wasting (<-2 SD) rate	13.5	14.9
Chad	TCD	Contraceptive prevalence (CPR)	7.25	6.5
Colombia	COL	Safe abortion services	0.2	0.2
Colombia	COL	TT - Tetanus toxoid vaccination	95.0	77.4
Colombia	COL	Syphilis detection and treatment	24.0	19.5
Colombia	COL	Iron supplementation in pregnancy	80.7	65.7
Colombia	COL	Hypertensive disorder case management	21.5	17.5
Colombia	COL	Diabetes case management	16.8	13.7
Colombia	COL	Malaria case management	69.5	56.6
Colombia	COL	MgSO4 management of pre-eclampsia	42.6	34.7
Colombia	COL	Thermal protection	95.2	81.6
Colombia	COL	Clean cord care	91.9	78.8
Colombia	COL	Clean birth environment	79.0	67.7
Colombia	COL	Immediate drying and additional stimulation	88.2	75.6
Colombia	COL	Neonatal resuscitation	53.0	45.4
Colombia	COL	Antibiotics for preterm or prolonged PROM	72.1	61.8
Colombia	COL	Parenteral administration of anti-convulsants	68.9	59.1
Colombia	COL	Parenteral administration of uterotonics	86.1	73.8
Colombia	COL	Parenteral administration of antibiotics	72.1	61.8
Colombia	COL	Assisted vaginal delivery	24.3	20.8
Colombia	COL	Manual removal of placenta	36.0	30.9
Colombia	COL	Removal of retained products of conception	31.9	27.4

Colombia	COL	Cesarean delivery	8.4	7.2
Colombia	COL	Blood transfusion	12.1	10.4
Colombia	COL	Induction of labor for pregnancies lasting 41+ weeks	1.7	1.5
Colombia	COL	Complementary feeding - education only	72.3	62.0
Colombia	COL	Complementary feeding - supplementary feeding and education	72.3	62.0
Colombia	COL	Improved sanitation - Utilization of latrines or toilets	89.6	89.6
Colombia	COL	Improved water source	97.3	97.3
Colombia	COL	Water connection in the home	86.3	86.3
Colombia	COL	Hygienic disposal of children's stools	14.4	14.4
Colombia	COL	Injectable antibiotics for neonatal sepsis	96.3	78.4
Colombia	COL	ORS - oral rehydration solution	54.1	46.4
Colombia	COL	Antibiotics for treatment of dysentery	5.8	5.0
Colombia	COL	SAM - treatment for severe acute malnutrition	0.1	0.1
Colombia	COL	BCG vaccine	89.0	72.5
Colombia	COL	Polio vaccine	92.0	74.9
Colombia	COL	DPT vaccine	92.0	74.9
Colombia	COL	H. influenzae type b vaccine	92.0	86.5
Colombia	COL	HepB vaccine	92.0	74.9
Colombia	COL	Pneumococcal vaccine	94.0	88.4
Colombia	COL	Rotavirus vaccine	90.0	84.7
Colombia	COL	Measles vaccine	95.0	77.4
Colombia	COL	Global wasting (<-2 SD) rate	0.9	1.0
Colombia	COL	Contraceptive prevalence (CPR)	81.4	73.5
Comoros	COM	Safe abortion services	3.3	3.0
Comoros	COM	TT - Tetanus toxoid vaccination	85.0	69.2
Comoros	COM	IPTp - Intermittent preventive treatment of malaria during pregnancy	30.8	25.1
Comoros	COM	Syphilis detection and treatment	22.8	18.6
Comoros	COM	Iron supplementation in pregnancy	12.6	10.3
Comoros	COM	Hypertensive disorder case management	11.8	9.6
Comoros	COM	Diabetes case management	9.2	7.5
Comoros	COM	Malaria case management	38.2	31.1
Comoros	COM	MgSO4 management of pre-eclampsia	23.4	19.1
Comoros	COM	Thermal protection	75.3	64.6

Comoros	COM	Clean cord care	72.7	62.3
Comoros	COM	Clean birth environment	62.4	53.5
Comoros	COM	Immediate drying and additional stimulation	69.7	59.8
Comoros	COM	Neonatal resuscitation	41.9	35.9
Comoros	COM	Antibiotics for preterm or prolonged PROM	57.0	48.9
Comoros	COM	Parenteral administration of anti-convulsants	54.4	46.6
Comoros	COM	Parenteral administration of uterotonics	68.0	58.3
Comoros	COM	Parenteral administration of antibiotics	57.0	48.9
Comoros	COM	Assisted vaginal delivery	19.2	16.5
Comoros	COM	Manual removal of placenta	28.5	24.4
Comoros	COM	Removal of retained products of conception	25.2	21.6
Comoros	COM	Cesarean delivery	6.6	5.7
Comoros	COM	Blood transfusion	9.6	8.2
Comoros	COM	Induction of labor for pregnancies lasting 41+ weeks	1.3	1.1
Comoros	COM	Complementary feeding - education only	25.2	21.6
Comoros	COM	Complementary feeding - supplementary feeding and education	25.2	21.6
Comoros	COM	Vitamin A supplementation	21.0	18.0
Comoros	COM	Improved sanitation - Utilization of latrines or toilets	35.9	35.9
Comoros	COM	Improved water source	80.2	80.2
Comoros	COM	Water connection in the home	47.6	47.6
Comoros	COM	Hand washing with soap	20.0	20.0
Comoros	COM	Hygienic disposal of children's stools	60.4	60.4
Comoros	COM	ITN/IRS - Households protected from malaria	60.6	52.0
Comoros	COM	Injectable antibiotics for neonatal sepsis	76.1	62.0
Comoros	COM	ORS - oral rehydration solution	37.5	32.2
Comoros	COM	Antibiotics for treatment of dysentery	19.4	16.6
Comoros	COM	Zinc for treatment of diarrhea	0.4	0.3
Comoros	COM	Oral antibiotics for pneumonia	38.1	32.7
Comoros	COM	Vitamin A for treatment of measles	21.0	18.0
Comoros	COM	ACTs- Artemisinin compounds for treatment of malaria	3.9	3.3
Comoros	COM	SAM - treatment for severe acute malnutrition	25.0	21.4
Comoros	COM	BCG vaccine	94.0	76.6
Comoros	COM	Polio vaccine	94.0	76.6

Comoros	COM	DPT vaccine	91.0	74.1
Comoros	COM	H. influenzae type b vaccine	91.0	85.6
Comoros	COM	HepB vaccine	91.0	74.1
Comoros	COM	Measles vaccine	90.0	73.3
Comoros	COM	Global wasting (<-2 SD) rate	11.2	12.3
Comoros	COM	Contraceptive prevalence (CPR)	26.4	23.8
Congo	COG	TT - Tetanus toxoid vaccination	85.0	69.2
Congo	COG	IPTp - Intermittent preventive treatment of malaria during pregnancy	23.2	18.9
Congo	COG	Syphilis detection and treatment	23.0	18.7
Congo	COG	Iron supplementation in pregnancy	42.9	34.9
Congo	COG	Hypertensive disorder case management	19.0	15.5
Congo	COG	Diabetes case management	14.8	12.1
Congo	COG	Malaria case management	61.2	49.8
Congo	COG	MgSO4 management of pre-eclampsia	37.5	30.5
Congo	COG	Thermal protection	90.4	77.5
Congo	COG	Clean cord care	87.3	74.8
Congo	COG	Clean birth environment	75.0	64.3
Congo	COG	Immediate drying and additional stimulation	83.8	71.8
Congo	COG	Neonatal resuscitation	50.3	43.1
Congo	COG	Antibiotics for preterm or prolonged PROM	68.5	58.7
Congo	COG	Parenteral administration of anti-convulsants	65.4	56.1
Congo	COG	Parenteral administration of uterotonics	81.8	70.1
Congo	COG	Parenteral administration of antibiotics	68.5	58.7
Congo	COG	Assisted vaginal delivery	23.1	19.8
Congo	COG	Manual removal of placenta	34.2	29.3
Congo	COG	Removal of retained products of conception	30.3	26.0
Congo	COG	Cesarean delivery	8.0	6.9
Congo	COG	Blood transfusion	11.5	9.9
Congo	COG	Induction of labor for pregnancies lasting 41+ weeks	1.6	1.4
Congo	COG	Complementary feeding - education only	23.9	20.5
Congo	COG	Complementary feeding - supplementary feeding and education	23.9	20.5
Congo	COG	Vitamin A supplementation	12.0	10.3
Congo	COG	Improved sanitation - Utilization of latrines or toilets	20.2	20.2

Congo	COG	Improved water source	73.2	73.2
Congo	COG	Water connection in the home	38.0	38.0
Congo	COG	Hand washing with soap	49.2	49.2
Congo	COG	Hygienic disposal of children's stools	62.8	62.8
Congo	COG	ITN/IRS - Households protected from malaria	66.1	56.7
Congo	COG	Injectable antibiotics for neonatal sepsis	91.5	74.5
Congo	COG	ORS - oral rehydration solution	26.9	23.1
Congo	COG	Antibiotics for treatment of dysentery	4.9	4.2
Congo	COG	Zinc for treatment of diarrhea	16.0	13.7
Congo	COG	Oral antibiotics for pneumonia	28.2	24.2
Congo	COG	Vitamin A for treatment of measles	12.0	10.3
Congo	COG	ACTs- Artemisinin compounds for treatment of malaria	8.8	7.5
Congo	COG	SAM - treatment for severe acute malnutrition	1.0	0.9
Congo	COG	BCG vaccine	81.0	66.0
Congo	COG	Polio vaccine	75.0	61.1
Congo	COG	DPT vaccine	75.0	61.1
Congo	COG	H. influenzae type b vaccine	75.0	70.5
Congo	COG	HepB vaccine	75.0	61.1
Congo	COG	Pneumococcal vaccine	73.0	68.7
Congo	COG	Rotavirus vaccine	72.0	67.7
Congo	COG	Meningococcal A	83.0	67.6
Congo	COG	Measles vaccine	75.0	61.1
Congo	COG	Global wasting (<-2 SD) rate	8.1	9.0
Congo	COG	Contraceptive prevalence (CPR)	38.6	34.8
Costa Rica	CRI	Safe abortion services	0.6	0.5
Costa Rica	CRI	Syphilis detection and treatment	24.2	19.7
Costa Rica	CRI	Hypertensive disorder case management	21.7	17.7
Costa Rica	CRI	Diabetes case management	16.9	13.8
Costa Rica	CRI	Malaria case management	69.9	56.9
Costa Rica	CRI	MgSO4 management of pre-eclampsia	42.9	34.9
Costa Rica	CRI	Thermal protection	97.0	83.2
Costa Rica	CRI	Clean cord care	93.6	80.3
Costa Rica	CRI	Clean birth environment	80.5	69.0
Costa Rica	CRI	Immediate drying and additional stimulation	89.8	77.0
Costa Rica	CRI	Neonatal resuscitation	53.9	46.2

Costa Rica	CRI	Antibiotics for preterm or prolonged PROM	73.4	62.9
Costa Rica	CRI	Parenteral administration of anti-convulsants	70.1	60.1
Costa Rica	CRI	Parenteral administration of uterotonics	87.7	75.2
Costa Rica	CRI	Parenteral administration of antibiotics	73.4	62.9
Costa Rica	CRI	Assisted vaginal delivery	24.8	21.3
Costa Rica	CRI	Manual removal of placenta	36.7	31.5
Costa Rica	CRI	Removal of retained products of conception	32.5	27.9
Costa Rica	CRI	Cesarean delivery	8.5	7.3
Costa Rica	CRI	Blood transfusion	12.4	10.6
Costa Rica	CRI	Induction of labor for pregnancies lasting 41+ weeks	1.7	1.5
Costa Rica	CRI	Improved sanitation - Utilization of latrines or toilets	97.8	97.8
Costa Rica	CRI	Improved water source	99.0	99.0
Costa Rica	CRI	Water connection in the home	99.0	99.0
Costa Rica	CRI	Hand washing with soap	89.4	89.4
Costa Rica	CRI	Hygienic disposal of children's stools	27.9	27.9
Costa Rica	CRI	Injectable antibiotics for neonatal sepsis	98.1	79.9
Costa Rica	CRI	ORS - oral rehydration solution	40.0	34.3
Costa Rica	CRI	Antibiotics for treatment of dysentery	8.7	7.5
Costa Rica	CRI	Zinc for treatment of diarrhea	1.3	1.1
Costa Rica	CRI	Oral antibiotics for pneumonia	77.2	66.2
Costa Rica	CRI	BCG vaccine	92.0	74.9
Costa Rica	CRI	Polio vaccine	94.0	76.6
Costa Rica	CRI	DPT vaccine	94.0	76.6
Costa Rica	CRI	H. influenzae type b vaccine	94.0	88.4
Costa Rica	CRI	HepB vaccine	98.0	79.8
Costa Rica	CRI	Pneumococcal vaccine	96.0	90.3
Costa Rica	CRI	Measles vaccine	94.0	76.6
Costa Rica	CRI	Global wasting (<-2 SD) rate	2.4	2.7
Costa Rica	CRI	Contraceptive prevalence (CPR)	77.45	69.9
Côte d'Ivoire	CIV	TT - Tetanus toxoid vaccination	85.0	69.2
Côte d'Ivoire	CIV	IPTp - Intermittent preventive treatment of malaria during pregnancy	43.8	35.7
Côte d'Ivoire	CIV	Syphilis detection and treatment	23.0	18.7
Côte d'Ivoire	CIV	Iron supplementation in pregnancy	25.0	20.4
Côte d'Ivoire	CIV	Hypertensive disorder case management	12.3	10.0

Côte d'Ivoire	CIV	Diabetes case management	9.6	7.8
Côte d'Ivoire	CIV	Malaria case management	39.7	32.3
Côte d'Ivoire	CIV	MgSO4 management of pre-eclampsia	24.4	19.9
Côte d'Ivoire	CIV	Thermal protection	69.0	59.2
Côte d'Ivoire	CIV	Clean cord care	66.6	57.1
Côte d'Ivoire	CIV	Clean birth environment	57.2	49.0
Côte d'Ivoire	CIV	Immediate drying and additional stimulation	63.9	54.8
Côte d'Ivoire	CIV	Neonatal resuscitation	38.4	32.9
Côte d'Ivoire	CIV	Antibiotics for preterm or prolonged PROM	52.2	44.8
Côte d'Ivoire	CIV	Parenteral administration of anti-convulsants	49.9	42.8
Côte d'Ivoire	CIV	Parenteral administration of uterotonics	62.4	53.5
Côte d'Ivoire	CIV	Parenteral administration of antibiotics	52.2	44.8
Côte d'Ivoire	CIV	Assisted vaginal delivery	17.6	15.1
Côte d'Ivoire	CIV	Manual removal of placenta	26.1	22.4
Côte d'Ivoire	CIV	Removal of retained products of conception	23.1	19.8
Côte d'Ivoire	CIV	Cesarean delivery	6.1	5.2
Côte d'Ivoire	CIV	Blood transfusion	8.8	7.5
Côte d'Ivoire	CIV	Induction of labor for pregnancies lasting 41+ weeks	1.2	1.0
Côte d'Ivoire	CIV	Complementary feeding - education only	26.0	22.3
Côte d'Ivoire	CIV	Complementary feeding - supplementary feeding and education	26.0	22.3
Côte d'Ivoire	CIV	Vitamin A supplementation	94.0	80.6
Côte d'Ivoire	CIV	Improved sanitation - Utilization of latrines or toilets	32.1	32.1
Côte d'Ivoire	CIV	Improved water source	72.9	72.9
Côte d'Ivoire	CIV	Water connection in the home	30.2	30.2
Côte d'Ivoire	CIV	Hand washing with soap	18.0	18.0
Côte d'Ivoire	CIV	Hygienic disposal of children's stools	54.4	54.4
Côte d'Ivoire	CIV	ITN/IRS - Households protected from malaria	75.8	65.0
Côte d'Ivoire	CIV	Injectable antibiotics for neonatal sepsis	69.8	56.9
Côte d'Ivoire	CIV	ORS - oral rehydration solution	16.5	14.1
Côte d'Ivoire	CIV	Antibiotics for treatment of dysentery	17.0	14.6
Côte d'Ivoire	CIV	Zinc for treatment of diarrhea	18.3	15.7
Côte d'Ivoire	CIV	Oral antibiotics for pneumonia	44.0	37.7
Côte d'Ivoire	CIV	Vitamin A for treatment of measles	94.0	80.6

Côte d'Ivoire	CIV	ACTs- Artemisinin compounds for treatment of malaria	0.3	0.3
Côte d'Ivoire	CIV	BCG vaccine	98.0	79.8
Côte d'Ivoire	CIV	Polio vaccine	82.0	66.8
Côte d'Ivoire	CIV	DPT vaccine	82.0	66.8
Côte d'Ivoire	CIV	H. influenzae type b vaccine	82.0	77.1
Côte d'Ivoire	CIV	HepB vaccine	82.0	66.8
Côte d'Ivoire	CIV	Pneumococcal vaccine	81.0	76.2
Côte d'Ivoire	CIV	Rotavirus vaccine	59.0	55.5
Côte d'Ivoire	CIV	Measles vaccine	71.0	57.8
Côte d'Ivoire	CIV	Global wasting (<-2 SD) rate	6.0	6.6
Côte d'Ivoire	CIV	Contraceptive prevalence (CPR)	21.25	19.2
Cuba	CUB	Safe abortion services	53.7	48.5
Cuba	CUB	Syphilis detection and treatment	24.3	19.8
Cuba	CUB	Hypertensive disorder case management	23.5	19.1
Cuba	CUB	Diabetes case management	18.3	14.9
Cuba	CUB	Malaria case management	75.8	61.7
Cuba	CUB	MgSO4 management of pre-eclampsia	46.5	37.9
Cuba	CUB	Thermal protection	97.9	83.9
Cuba	CUB	Clean cord care	94.5	81.0
Cuba	CUB	Clean birth environment	81.2	69.6
Cuba	CUB	Immediate drying and additional stimulation	90.7	77.8
Cuba	CUB	Neonatal resuscitation	54.5	46.7
Cuba	CUB	Antibiotics for preterm or prolonged PROM	74.1	63.5
Cuba	CUB	Parenteral administration of anti-convulsants	70.8	60.7
Cuba	CUB	Parenteral administration of uterotonics	88.5	75.9
Cuba	CUB	Parenteral administration of antibiotics	74.1	63.5
Cuba	CUB	Assisted vaginal delivery	25.0	21.4
Cuba	CUB	Manual removal of placenta	37.0	31.7
Cuba	CUB	Removal of retained products of conception	32.8	28.1
Cuba	CUB	Cesarean delivery	8.6	7.4
Cuba	CUB	Blood transfusion	12.5	10.7
Cuba	CUB	Induction of labor for pregnancies lasting 41+ weeks	1.7	1.5
Cuba	CUB	Complementary feeding - education only	80.3	68.8
Cuba	CUB	Complementary feeding - supplementary feeding and education	80.3	68.8

Cuba	CUB	Improved sanitation - Utilization of latrines or toilets	92.8	92.8
Cuba	CUB	Improved water source	95.3	95.3
Cuba	CUB	Water connection in the home	75.8	75.8
Cuba	CUB	Hand washing with soap	85.2	85.2
Cuba	CUB	Hygienic disposal of children's stools	87.9	87.9
Cuba	CUB	Injectable antibiotics for neonatal sepsis	99.0	80.6
Cuba	CUB	ORS - oral rehydration solution	60.9	52.2
Cuba	CUB	Antibiotics for treatment of dysentery	2.0	1.7
Cuba	CUB	Zinc for treatment of diarrhea	17.3	14.8
Cuba	CUB	Oral antibiotics for pneumonia	92.6	79.4
Cuba	CUB	BCG vaccine	99.0	80.6
Cuba	CUB	Polio vaccine	99.0	80.6
Cuba	CUB	DPT vaccine	99.0	80.6
Cuba	CUB	H. influenzae type b vaccine	99.0	93.1
Cuba	CUB	HepB vaccine	99.0	80.6
Cuba	CUB	Measles vaccine	99.0	80.6
Cuba	CUB	Global wasting (<-2 SD) rate	2.2	2.4
Cuba	CUB	Contraceptive prevalence (CPR)	74.4	67.1
Dem. People's Republic of Korea	PRK	Safe abortion services	100.0	90.3
Dem. People's Republic of Korea	PRK	TT - Tetanus toxoid vaccination	98.0	79.8
Dem. People's Republic of Korea	PRK	Syphilis detection and treatment	24.4	19.9
Dem. People's Republic of Korea	PRK	Hypertensive disorder case management	22.5	18.3
Dem. People's Republic of Korea	PRK	Diabetes case management	17.6	14.3
Dem. People's Republic of Korea	PRK	Malaria case management	72.6	59.1
Dem. People's Republic of Korea	PRK	MgSO4 management of pre-eclampsia	44.5	36.2
Dem. People's Republic of Korea	PRK	Thermal protection	91.1	78.1
Dem. People's Republic of Korea	PRK	Clean cord care	88.0	75.4
Dem. People's Republic of Korea	PRK	Clean birth environment	75.6	64.8

Dem. People's Republic of Korea	PRK	Immediate drying and additional stimulation	84.4	72.4
Dem. People's Republic of Korea	PRK	Neonatal resuscitation	50.7	43.5
Dem. People's Republic of Korea	PRK	Antibiotics for preterm or prolonged PROM	69.0	59.2
Dem. People's Republic of Korea	PRK	Parenteral administration of anti-convulsants	65.9	56.5
Dem. People's Republic of Korea	PRK	Parenteral administration of uterotonics	82.4	70.6
Dem. People's Republic of Korea	PRK	Parenteral administration of antibiotics	69.0	59.2
Dem. People's Republic of Korea	PRK	Assisted vaginal delivery	23.3	20.0
Dem. People's Republic of Korea	PRK	Manual removal of placenta	34.5	29.6
Dem. People's Republic of Korea	PRK	Removal of retained products of conception	30.6	26.2
Dem. People's Republic of Korea	PRK	Cesarean delivery	8.0	6.9
Dem. People's Republic of Korea	PRK	Blood transfusion	11.6	9.9
Dem. People's Republic of Korea	PRK	Induction of labor for pregnancies lasting 41+ weeks	1.6	1.4
Dem. People's Republic of Korea	PRK	Complementary feeding - education only	48.7	41.8
Dem. People's Republic of Korea	PRK	Complementary feeding - supplementary feeding and education	48.7	41.8
Dem. People's Republic of Korea	PRK	Vitamin A supplementation	90.0	77.2
Dem. People's Republic of Korea	PRK	Improved sanitation - Utilization of latrines or toilets	83.2	83.2
Dem. People's Republic of Korea	PRK	Improved water source	99.0	99.0
Dem. People's Republic of Korea	PRK	Water connection in the home	63.8	63.8
Dem. People's Republic of Korea	PRK	Hygienic disposal of children's stools	74.2	74.2
Dem. People's Republic of Korea	PRK	Injectable antibiotics for neonatal sepsis	92.2	75.1
Dem. People's Republic of Korea	PRK	ORS - oral rehydration solution	74.1	63.5

Dem. People's Republic of Korea	PRK	Zinc for treatment of diarrhea	50.7	43.5
Dem. People's Republic of Korea	PRK	Oral antibiotics for pneumonia	79.8	68.4
Dem. People's Republic of Korea	PRK	Vitamin A for treatment of measles	90.0	77.2
Dem. People's Republic of Korea	PRK	BCG vaccine	96.0	78.2
Dem. People's Republic of Korea	PRK	Polio vaccine	99.0	80.6
Dem. People's Republic of Korea	PRK	DPT vaccine	97.0	79.0
Dem. People's Republic of Korea	PRK	H. influenzae type b vaccine	97.0	91.2
Dem. People's Republic of Korea	PRK	HepB vaccine	97.0	79.0
Dem. People's Republic of Korea	PRK	Measles vaccine	98.0	79.8
Dem. People's Republic of Korea	PRK	Global wasting (<-2 SD) rate	10.6	11.7
Dem. People's Republic of Korea	PRK	Contraceptive prevalence (CPR)	74.1	66.9
Democratic Republic of the Congo	COD	TT - Tetanus toxoid vaccination	85.0	69.2
Democratic Republic of the Congo	COD	IPTp - Intermittent preventive treatment of malaria during pregnancy	15.0	12.2
Democratic Republic of the Congo	COD	Syphilis detection and treatment	34.9	28.4
Democratic Republic of the Congo	COD	Iron supplementation in pregnancy	4.7	3.8
Democratic Republic of the Congo	COD	Hypertensive disorder case management	4.6	3.7
Democratic Republic of the Congo	COD	Diabetes case management	4.7	3.8
Democratic Republic of the Congo	COD	Malaria case management	31.9	26.0
Democratic Republic of the Congo	COD	MgSO4 management of pre-eclampsia	6.6	5.4
Democratic Republic of the Congo	COD	Thermal protection	78.9	67.6
Democratic Republic of the Congo	COD	Clean cord care	66.6	57.1

Democratic Republic of the Congo	COD	Clean birth environment	40.9	35.1
Democratic Republic of the Congo	COD	Immediate drying and additional stimulation	63.2	54.2
Democratic Republic of the Congo	COD	Neonatal resuscitation	4.4	3.8
Democratic Republic of the Congo	COD	Antibiotics for preterm or prolonged PROM	12.2	10.5
Democratic Republic of the Congo	COD	Parenteral administration of anti-convulsants	14.2	12.2
Democratic Republic of the Congo	COD	Parenteral administration of uterotonics	61.3	52.6
Democratic Republic of the Congo	COD	Parenteral administration of antibiotics	12.2	10.5
Democratic Republic of the Congo	COD	Assisted vaginal delivery	8.9	7.6
Democratic Republic of the Congo	COD	Manual removal of placenta	21.2	18.2
Democratic Republic of the Congo	COD	Removal of retained products of conception	19.9	17.1
Democratic Republic of the Congo	COD	Cesarean delivery	0.8	0.7
Democratic Republic of the Congo	COD	Blood transfusion	6.6	5.7
Democratic Republic of the Congo	COD	Induction of labor for pregnancies lasting 41+ weeks	1.2	1.0
Democratic Republic of the Congo	COD	Complementary feeding - education only	19.9	17.1
Democratic Republic of the Congo	COD	Complementary feeding - supplementary feeding and education	19.9	17.1
Democratic Republic of the Congo	COD	Vitamin A supplementation	1.0	0.9
Democratic Republic of the Congo	COD	Improved sanitation - Utilization of latrines or toilets	20.5	20.5
Democratic Republic of the Congo	COD	Improved water source	43.2	43.2
Democratic Republic of the Congo	COD	Water connection in the home	13.9	13.9
Democratic Republic of the Congo	COD	Hand washing with soap	4.4	4.4
Democratic Republic of the Congo	COD	Hygienic disposal of children's stools	61.1	61.1

Democratic Republic of the Congo	COD	ITN/IRS - Households protected from malaria	70.0	60.0
Democratic Republic of the Congo	COD	Injectable antibiotics for neonatal sepsis	79.9	65.1
Democratic Republic of the Congo	COD	ORS - oral rehydration solution	39.1	33.5
Democratic Republic of the Congo	COD	Antibiotics for treatment of dysentery	38.4	32.9
Democratic Republic of the Congo	COD	Zinc for treatment of diarrhea	2.4	2.1
Democratic Republic of the Congo	COD	Oral antibiotics for pneumonia	41.6	35.7
Democratic Republic of the Congo	COD	Vitamin A for treatment of measles	1.0	0.9
Democratic Republic of the Congo	COD	ACTs- Artemisinin compounds for treatment of malaria	2.0	1.7
Democratic Republic of the Congo	COD	SAM - treatment for severe acute malnutrition	11.2	9.6
Democratic Republic of the Congo	COD	BCG vaccine	83.0	67.6
Democratic Republic of the Congo	COD	Polio vaccine	79.0	64.3
Democratic Republic of the Congo	COD	DPT vaccine	81.0	66.0
Democratic Republic of the Congo	COD	H. influenzae type b vaccine	81.0	76.2
Democratic Republic of the Congo	COD	HepB vaccine	81.0	66.0
Democratic Republic of the Congo	COD	Pneumococcal vaccine	81.0	76.2
Democratic Republic of the Congo	COD	Meningococcal A	99.0	80.6
Democratic Republic of the Congo	COD	Measles vaccine	80.0	65.2
Democratic Republic of the Congo	COD	Global wasting (<-2 SD) rate	8.1	8.9
Democratic Republic of the Congo	COD	Contraceptive prevalence (CPR)	24.55	22.2
Djibouti	DJI	Safe abortion services	3.3	3.0
Djibouti	DJI	TT - Tetanus toxoid vaccination	98.0	79.8
Djibouti	DJI	Syphilis detection and treatment	5.6	4.6
Djibouti	DJI	Hypertensive disorder case management	5.4	4.4

Djibouti	DJI	Diabetes case management	4.2	3.4
Djibouti	DJI	Malaria case management	17.5	14.3
Djibouti	DJI	MgSO4 management of pre-eclampsia	10.7	8.7
Djibouti	DJI	Thermal protection	86.4	74.1
Djibouti	DJI	Clean cord care	83.4	71.5
Djibouti	DJI	Clean birth environment	71.7	61.5
Djibouti	DJI	Immediate drying and additional stimulation	80.0	68.6
Djibouti	DJI	Neonatal resuscitation	48.1	41.2
Djibouti	DJI	Antibiotics for preterm or prolonged PROM	65.4	56.1
Djibouti	DJI	Parenteral administration of anti-convulsants	62.5	53.6
Djibouti	DJI	Parenteral administration of uterotonics	78.1	67.0
Djibouti	DJI	Parenteral administration of antibiotics	65.4	56.1
Djibouti	DJI	Assisted vaginal delivery	22.1	18.9
Djibouti	DJI	Manual removal of placenta	32.7	28.0
Djibouti	DJI	Removal of retained products of conception	29.0	24.9
Djibouti	DJI	Cesarean delivery	7.6	6.5
Djibouti	DJI	Blood transfusion	11.0	9.4
Djibouti	DJI	Induction of labor for pregnancies lasting 41+ weeks	1.5	1.3
Djibouti	DJI	Complementary feeding - education only	23.1	19.8
Djibouti	DJI	Complementary feeding - supplementary feeding and education	23.1	19.8
Djibouti	DJI	Vitamin A supplementation	78.0	66.9
Djibouti	DJI	Improved sanitation - Utilization of latrines or toilets	63.6	63.6
Djibouti	DJI	Improved water source	75.6	75.6
Djibouti	DJI	Water connection in the home	61.8	61.8
Djibouti	DJI	ITN/IRS - Households protected from malaria	30.2	25.9
Djibouti	DJI	Injectable antibiotics for neonatal sepsis	87.4	71.2
Djibouti	DJI	ORS - oral rehydration solution	61.6	52.8
Djibouti	DJI	Oral antibiotics for pneumonia	62.1	53.2
Djibouti	DJI	Vitamin A for treatment of measles	78.0	66.9
Djibouti	DJI	SAM - treatment for severe acute malnutrition	32.4	27.8
Djibouti	DJI	BCG vaccine	93.0	75.7
Djibouti	DJI	Polio vaccine	84.0	68.4
Djibouti	DJI	DPT vaccine	84.0	68.4
Djibouti	DJI	H. influenzae type b vaccine	84.0	79.0

Djibouti	DJI	HepB vaccine	84.0	68.4
Djibouti	DJI	Pneumococcal vaccine	84.0	79.0
Djibouti	DJI	Rotavirus vaccine	87.0	81.8
Djibouti	DJI	Measles vaccine	86.0	70.0
Djibouti	DJI	Global wasting (<-2 SD) rate	26.8	29.4
Djibouti	DJI	Contraceptive prevalence (CPR)	27.6	24.9
Dominican Republic	DOM	Safe abortion services	53.7	48.5
Dominican Republic	DOM	TT - Tetanus toxoid vaccination	99.0	80.6
Dominican Republic	DOM	Syphilis detection and treatment	24.2	19.7
Dominican Republic	DOM	Iron supplementation in pregnancy	81.5	66.4
Dominican Republic	DOM	Hypertensive disorder case management	22.3	18.2
Dominican Republic	DOM	Diabetes case management	17.4	14.2
Dominican Republic	DOM	Malaria case management	72.0	58.6
Dominican Republic	DOM	MgSO4 management of pre-eclampsia	44.2	36.0
Dominican Republic	DOM	Thermal protection	96.7	82.9
Dominican Republic	DOM	Clean cord care	93.4	80.1
Dominican Republic	DOM	Clean birth environment	80.3	68.8
Dominican Republic	DOM	Immediate drying and additional stimulation	89.6	76.8
Dominican Republic	DOM	Neonatal resuscitation	53.8	46.1
Dominican Republic	DOM	Antibiotics for preterm or prolonged PROM	73.2	62.8
Dominican Republic	DOM	Parenteral administration of anti-convulsants	70.0	60.0
Dominican Republic	DOM	Parenteral administration of uterotonics	87.5	75.0
Dominican Republic	DOM	Parenteral administration of antibiotics	73.2	62.8
Dominican Republic	DOM	Assisted vaginal delivery	24.7	21.2
Dominican Republic	DOM	Manual removal of placenta	36.6	31.4
Dominican Republic	DOM	Removal of retained products of conception	32.4	27.8
Dominican Republic	DOM	Cesarean delivery	8.5	7.3
Dominican Republic	DOM	Blood transfusion	12.3	10.5
Dominican Republic	DOM	Induction of labor for pregnancies lasting 41+ weeks	1.7	1.5
Dominican Republic	DOM	Complementary feeding - education only	68.5	58.7
Dominican Republic	DOM	Complementary feeding - supplementary feeding and education	68.5	58.7
Dominican Republic	DOM	Improved sanitation - Utilization of latrines or toilets	83.9	83.9
Dominican Republic	DOM	Improved water source	96.7	96.7
Dominican Republic	DOM	Water connection in the home	77.9	77.9

Dominican Republic	DOM	Hand washing with soap	57.1	57.1
Dominican Republic	DOM	Hygienic disposal of children's stools	27.4	27.4
Dominican Republic	DOM	Injectable antibiotics for neonatal sepsis	97.9	79.7
Dominican Republic	DOM	ORS - oral rehydration solution	47.5	40.7
Dominican Republic	DOM	Antibiotics for treatment of dysentery	30.5	26.1
Dominican Republic	DOM	Oral antibiotics for pneumonia	72.4	62.1
Dominican Republic	DOM	BCG vaccine	99.0	80.6
Dominican Republic	DOM	Polio vaccine	89.0	72.5
Dominican Republic	DOM	DPT vaccine	94.0	76.6
Dominican Republic	DOM	H. influenzae type b vaccine	90.0	84.7
Dominican Republic	DOM	HepB vaccine	92.0	74.9
Dominican Republic	DOM	Pneumococcal vaccine	70.0	65.8
Dominican Republic	DOM	Rotavirus vaccine	82.0	77.1
Dominican Republic	DOM	Measles vaccine	95.0	77.4
Dominican Republic	DOM	Global wasting (<-2 SD) rate	2.5	2.7
Dominican Republic	DOM	Contraceptive prevalence (CPR)	71.15	64.2
Ecuador	ECU	Safe abortion services	0.2	0.2
Ecuador	ECU	TT - Tetanus toxoid vaccination	88.0	71.7
Ecuador	ECU	Syphilis detection and treatment	23.9	19.5
Ecuador	ECU	Hypertensive disorder case management	21.2	17.3
Ecuador	ECU	Diabetes case management	16.5	13.4
Ecuador	ECU	Malaria case management	68.4	55.7
Ecuador	ECU	MgSO4 management of pre-eclampsia	41.9	34.1
Ecuador	ECU	Thermal protection	89.3	76.6
Ecuador	ECU	Clean cord care	86.2	73.9
Ecuador	ECU	Clean birth environment	74.1	63.5
Ecuador	ECU	Immediate drying and additional stimulation	82.7	70.9
Ecuador	ECU	Neonatal resuscitation	49.7	42.6
Ecuador	ECU	Antibiotics for preterm or prolonged PROM	67.6	58.0
Ecuador	ECU	Parenteral administration of anti-convulsants	64.6	55.4
Ecuador	ECU	Parenteral administration of uterotonics	80.8	69.3
Ecuador	ECU	Parenteral administration of antibiotics	67.6	58.0
Ecuador	ECU	Assisted vaginal delivery	22.8	19.5
Ecuador	ECU	Manual removal of placenta	33.8	29.0
Ecuador	ECU	Removal of retained products of conception	29.9	25.6

Ecuador	ECU	Cesarean delivery	7.9	6.8
Ecuador	ECU	Blood transfusion	11.4	9.8
Ecuador	ECU	Induction of labor for pregnancies lasting 41+ weeks	1.6	1.4
Ecuador	ECU	Complementary feeding - education only	69.4	59.5
Ecuador	ECU	Complementary feeding - supplementary feeding and education	69.4	59.5
Ecuador	ECU	Improved sanitation - Utilization of latrines or toilets	88.0	88.0
Ecuador	ECU	Improved water source	94.0	94.0
Ecuador	ECU	Water connection in the home	82.6	82.6
Ecuador	ECU	Injectable antibiotics for neonatal sepsis	90.4	73.6
Ecuador	ECU	ORS - oral rehydration solution	27.3	23.4
Ecuador	ECU	Antibiotics for treatment of dysentery	32.4	27.8
Ecuador	ECU	BCG vaccine	90.0	73.3
Ecuador	ECU	Polio vaccine	85.0	69.2
Ecuador	ECU	DPT vaccine	85.0	69.2
Ecuador	ECU	H. influenzae type b vaccine	85.0	80.0
Ecuador	ECU	HepB vaccine	85.0	69.2
Ecuador	ECU	Pneumococcal vaccine	85.0	80.0
Ecuador	ECU	Rotavirus vaccine	85.0	80.0
Ecuador	ECU	Measles vaccine	83.0	67.6
Ecuador	ECU	Global wasting (<-2 SD) rate	2.5	2.8
Ecuador	ECU	Contraceptive prevalence (CPR)	79.35	71.6
Egypt	EGY	Safe abortion services	2.2	2.0
Egypt	EGY	TT - Tetanus toxoid vaccination	86.0	70.0
Egypt	EGY	Syphilis detection and treatment	22.6	18.4
Egypt	EGY	Iron supplementation in pregnancy	36.1	29.4
Egypt	EGY	Hypertensive disorder case management	20.1	16.4
Egypt	EGY	Diabetes case management	15.7	12.8
Egypt	EGY	Malaria case management	65.0	52.9
Egypt	EGY	MgSO4 management of pre-eclampsia	39.8	32.4
Egypt	EGY	Thermal protection	85.7	73.5
Egypt	EGY	Clean cord care	82.8	71.0
Egypt	EGY	Clean birth environment	71.1	61.0
Egypt	EGY	Immediate drying and additional stimulation	79.4	68.1
Egypt	EGY	Neonatal resuscitation	47.7	40.9

Egypt	EGY	Antibiotics for preterm or prolonged PROM	64.9	55.6
Egypt	EGY	Parenteral administration of anti-convulsants	62.0	53.2
Egypt	EGY	Parenteral administration of uterotonics	77.5	66.4
Egypt	EGY	Parenteral administration of antibiotics	64.9	55.6
Egypt	EGY	Assisted vaginal delivery	21.9	18.8
Egypt	EGY	Manual removal of placenta	32.4	27.8
Egypt	EGY	Removal of retained products of conception	28.7	24.6
Egypt	EGY	Cesarean delivery	7.5	6.4
Egypt	EGY	Blood transfusion	10.9	9.3
Egypt	EGY	Induction of labor for pregnancies lasting 41+ weeks	1.5	1.3
Egypt	EGY	Complementary feeding - education only	43.2	37.0
Egypt	EGY	Complementary feeding - supplementary feeding and education	43.2	37.0
Egypt	EGY	Vitamin A supplementation	68.0	58.3
Egypt	EGY	Improved sanitation - Utilization of latrines or toilets	94.2	94.2
Egypt	EGY	Improved water source	99.0	99.0
Egypt	EGY	Water connection in the home	97.2	97.2
Egypt	EGY	Hand washing with soap	89.0	89.0
Egypt	EGY	Hygienic disposal of children's stools	35.7	35.7
Egypt	EGY	Injectable antibiotics for neonatal sepsis	86.7	70.6
Egypt	EGY	ORS - oral rehydration solution	28.4	24.3
Egypt	EGY	Antibiotics for treatment of dysentery	13.2	11.3
Egypt	EGY	Zinc for treatment of diarrhea	1.7	1.5
Egypt	EGY	Oral antibiotics for pneumonia	67.7	58.0
Egypt	EGY	Vitamin A for treatment of measles	68.0	58.3
Egypt	EGY	BCG vaccine	95.0	77.4
Egypt	EGY	Polio vaccine	95.0	77.4
Egypt	EGY	DPT vaccine	95.0	77.4
Egypt	EGY	H. influenzae type b vaccine	95.0	89.4
Egypt	EGY	HepB vaccine	95.0	77.4
Egypt	EGY	Measles vaccine	94.0	76.6
Egypt	EGY	Global wasting (<-2 SD) rate	9.4	10.3
Egypt	EGY	Contraceptive prevalence (CPR)	60.95	55.0
El Salvador	SLV	Safe abortion services	0.6	0.5
El Salvador	SLV	TT - Tetanus toxoid vaccination	92.0	74.9

El Salvador	SLV	Syphilis detection and treatment	23.7	19.3
El Salvador	SLV	Hypertensive disorder case management	21.6	17.6
El Salvador	SLV	Diabetes case management	16.9	13.8
El Salvador	SLV	Malaria case management	69.9	56.9
El Salvador	SLV	MgSO4 management of pre-eclampsia	42.8	34.9
El Salvador	SLV	Thermal protection	96.4	82.7
El Salvador	SLV	Clean cord care	93.1	79.8
El Salvador	SLV	Clean birth environment	80.0	68.6
El Salvador	SLV	Immediate drying and additional stimulation	89.3	76.6
El Salvador	SLV	Neonatal resuscitation	53.6	46.0
El Salvador	SLV	Antibiotics for preterm or prolonged PROM	73.0	62.6
El Salvador	SLV	Parenteral administration of anti-convulsants	69.7	59.8
El Salvador	SLV	Parenteral administration of uterotonics	87.2	74.8
El Salvador	SLV	Parenteral administration of antibiotics	73.0	62.6
El Salvador	SLV	Assisted vaginal delivery	24.7	21.2
El Salvador	SLV	Manual removal of placenta	36.5	31.3
El Salvador	SLV	Removal of retained products of conception	32.3	27.7
El Salvador	SLV	Cesarean delivery	8.5	7.3
El Salvador	SLV	Blood transfusion	12.3	10.5
El Salvador	SLV	Induction of labor for pregnancies lasting 41+ weeks	1.7	1.5
El Salvador	SLV	Complementary feeding - education only	78.2	67.0
El Salvador	SLV	Complementary feeding - supplementary feeding and education	78.2	67.0
El Salvador	SLV	Vitamin A supplementation	81.0	69.4
El Salvador	SLV	Improved sanitation - Utilization of latrines or toilets	87.4	87.4
El Salvador	SLV	Improved water source	97.4	97.4
El Salvador	SLV	Water connection in the home	87.4	87.4
El Salvador	SLV	Hand washing with soap	91.3	91.3
El Salvador	SLV	Hygienic disposal of children's stools	41.8	41.8
El Salvador	SLV	Injectable antibiotics for neonatal sepsis	97.5	79.4
El Salvador	SLV	ORS - oral rehydration solution	69.8	59.8
El Salvador	SLV	Antibiotics for treatment of dysentery	40.8	35.0
El Salvador	SLV	Zinc for treatment of diarrhea	32.7	28.0
El Salvador	SLV	Oral antibiotics for pneumonia	79.7	68.3
El Salvador	SLV	Vitamin A for treatment of measles	81.0	69.4

El Salvador	SLV	SAM - treatment for severe acute malnutrition	1.9	1.6
El Salvador	SLV	BCG vaccine	81.0	66.0
El Salvador	SLV	Polio vaccine	83.0	67.6
El Salvador	SLV	DPT vaccine	81.0	66.0
El Salvador	SLV	H. influenzae type b vaccine	81.0	76.2
El Salvador	SLV	HepB vaccine	81.0	66.0
El Salvador	SLV	Pneumococcal vaccine	75.0	70.5
El Salvador	SLV	Rotavirus vaccine	82.0	77.1
El Salvador	SLV	Measles vaccine	81.0	66.0
El Salvador	SLV	Global wasting (<-2 SD) rate	2.2	2.4
El Salvador	SLV	Contraceptive prevalence (CPR)	73.05	65.9
Equatorial Guinea	GNQ	TT - Tetanus toxoid vaccination	70.0	57.0
Equatorial Guinea	GNQ	IPTp - Intermittent preventive treatment of malaria during pregnancy	27.6	22.5
Equatorial Guinea	GNQ	Syphilis detection and treatment	21.9	17.8
Equatorial Guinea	GNQ	Iron supplementation in pregnancy	8.7	7.1
Equatorial Guinea	GNQ	Hypertensive disorder case management	16.1	13.1
Equatorial Guinea	GNQ	Diabetes case management	12.5	10.2
Equatorial Guinea	GNQ	Malaria case management	51.9	42.3
Equatorial Guinea	GNQ	MgSO4 management of pre-eclampsia	31.8	25.9
Equatorial Guinea	GNQ	Thermal protection	66.5	57.0
Equatorial Guinea	GNQ	Clean cord care	64.2	55.0
Equatorial Guinea	GNQ	Clean birth environment	55.2	47.3
Equatorial Guinea	GNQ	Immediate drying and additional stimulation	61.6	52.8
Equatorial Guinea	GNQ	Neonatal resuscitation	37.0	31.7
Equatorial Guinea	GNQ	Antibiotics for preterm or prolonged PROM	50.4	43.2
Equatorial Guinea	GNQ	Parenteral administration of anti-convulsants	48.1	41.2
Equatorial Guinea	GNQ	Parenteral administration of uterotonics	60.1	51.5
Equatorial Guinea	GNQ	Parenteral administration of antibiotics	50.4	43.2
Equatorial Guinea	GNQ	Assisted vaginal delivery	17.0	14.6
Equatorial Guinea	GNQ	Manual removal of placenta	25.2	21.6
Equatorial Guinea	GNQ	Removal of retained products of conception	22.3	19.1
Equatorial Guinea	GNQ	Cesarean delivery	5.9	5.1
Equatorial Guinea	GNQ	Blood transfusion	8.5	7.3
Equatorial Guinea	GNQ	Induction of labor for pregnancies lasting 41+ weeks	1.2	1.0

Equatorial Guinea	GNQ	Complementary feeding - education only	40.7	34.9
Equatorial Guinea	GNQ	Complementary feeding - supplementary feeding and education	40.7	34.9
Equatorial Guinea	GNQ	Vitamin A supplementation	30.0	25.7
Equatorial Guinea	GNQ	Improved sanitation - Utilization of latrines or toilets	66.3	66.3
Equatorial Guinea	GNQ	Improved water source	64.7	64.7
Equatorial Guinea	GNQ	Water connection in the home	26.4	26.4
Equatorial Guinea	GNQ	ITN/IRS - Households protected from malaria	63.7	54.6
Equatorial Guinea	GNQ	Injectable antibiotics for neonatal sepsis	67.3	54.8
Equatorial Guinea	GNQ	ORS - oral rehydration solution	40.4	34.6
Equatorial Guinea	GNQ	Antibiotics for treatment of dysentery	9.3	8.0
Equatorial Guinea	GNQ	Zinc for treatment of diarrhea	0.2	0.2
Equatorial Guinea	GNQ	Oral antibiotics for pneumonia	54.3	46.6
Equatorial Guinea	GNQ	Vitamin A for treatment of measles	30.0	25.7
Equatorial Guinea	GNQ	ACTs- Artemisinin compounds for treatment of malaria	9.0	7.7
Equatorial Guinea	GNQ	BCG vaccine	63.0	51.3
Equatorial Guinea	GNQ	Polio vaccine	27.0	22.0
Equatorial Guinea	GNQ	DPT vaccine	25.0	20.4
Equatorial Guinea	GNQ	H. influenzae type b vaccine	25.0	23.5
Equatorial Guinea	GNQ	HepB vaccine	25.0	20.4
Equatorial Guinea	GNQ	Measles vaccine	30.0	24.4
Equatorial Guinea	GNQ	Global wasting (<-2 SD) rate	10.0	11.0
Equatorial Guinea	GNQ	Contraceptive prevalence (CPR)	17.35	15.7
Eritrea	ERI	Safe abortion services	3.3	3.0
Eritrea	ERI	TT - Tetanus toxoid vaccination	99.0	80.6
Eritrea	ERI	Syphilis detection and treatment	17.5	14.3
Eritrea	ERI	Hypertensive disorder case management	13.8	11.2
Eritrea	ERI	Diabetes case management	10.8	8.8
Eritrea	ERI	Malaria case management	44.5	36.2
Eritrea	ERI	MgSO4 management of pre-eclampsia	27.3	22.2
Eritrea	ERI	Thermal protection	33.2	28.5
Eritrea	ERI	Clean cord care	32.1	27.5
Eritrea	ERI	Clean birth environment	27.6	23.7
Eritrea	ERI	Immediate drying and additional stimulation	30.8	26.4
Eritrea	ERI	Neonatal resuscitation	18.5	15.9

Eritrea	ERI	Antibiotics for preterm or prolonged PROM	25.1	21.5
Eritrea	ERI	Parenteral administration of anti-convulsants	24.0	20.6
Eritrea	ERI	Parenteral administration of uterotonics	30.0	25.7
Eritrea	ERI	Parenteral administration of antibiotics	25.1	21.5
Eritrea	ERI	Assisted vaginal delivery	8.5	7.3
Eritrea	ERI	Manual removal of placenta	12.6	10.8
Eritrea	ERI	Removal of retained products of conception	11.1	9.5
Eritrea	ERI	Cesarean delivery	2.9	2.5
Eritrea	ERI	Blood transfusion	4.2	3.6
Eritrea	ERI	Induction of labor for pregnancies lasting 41+ weeks	0.6	0.5
Eritrea	ERI	Complementary feeding - education only	42.5	36.4
Eritrea	ERI	Complementary feeding - supplementary feeding and education	42.5	36.4
Eritrea	ERI	Vitamin A supplementation	51.0	43.7
Eritrea	ERI	Improved sanitation - Utilization of latrines or toilets	11.9	11.9
Eritrea	ERI	Improved water source	51.8	51.8
Eritrea	ERI	Water connection in the home	26.8	26.8
Eritrea	ERI	Hygienic disposal of children's stools	26.8	26.8
Eritrea	ERI	ITN/IRS - Households protected from malaria	70.9	60.8
Eritrea	ERI	Injectable antibiotics for neonatal sepsis	33.6	27.4
Eritrea	ERI	ORS - oral rehydration solution	43.4	37.2
Eritrea	ERI	Vitamin A for treatment of measles	51.0	43.7
Eritrea	ERI	BCG vaccine	97.0	79.0
Eritrea	ERI	Polio vaccine	95.0	77.4
Eritrea	ERI	DPT vaccine	95.0	77.4
Eritrea	ERI	H. influenzae type b vaccine	95.0	89.4
Eritrea	ERI	HepB vaccine	95.0	77.4
Eritrea	ERI	Pneumococcal vaccine	95.0	89.4
Eritrea	ERI	Rotavirus vaccine	96.0	90.3
Eritrea	ERI	Measles vaccine	99.0	80.6
Eritrea	ERI	Global wasting (<-2 SD) rate	7.2	7.9
Eritrea	ERI	Contraceptive prevalence (CPR)	13.8	12.5
Ethiopia	ETH	Safe abortion services	3.3	3.0
Ethiopia	ETH	TT - Tetanus toxoid vaccination	93.0	75.7

Ethiopia	ETH	IPTp - Intermittent preventive treatment of malaria during pregnancy	1.2	1.0
Ethiopia	ETH	Syphilis detection and treatment	15.7	12.8
Ethiopia	ETH	Iron supplementation in pregnancy	5.1	4.2
Ethiopia	ETH	Hypertensive disorder case management	7.9	6.4
Ethiopia	ETH	Diabetes case management	6.1	5.0
Ethiopia	ETH	Malaria case management	25.4	20.7
Ethiopia	ETH	MgSO4 management of pre-eclampsia	15.6	12.7
Ethiopia	ETH	Thermal protection	25.9	22.2
Ethiopia	ETH	Clean cord care	25.0	21.4
Ethiopia	ETH	Clean birth environment	21.5	18.4
Ethiopia	ETH	Immediate drying and additional stimulation	24.0	20.6
Ethiopia	ETH	Neonatal resuscitation	14.4	12.3
Ethiopia	ETH	Antibiotics for preterm or prolonged PROM	19.6	16.8
Ethiopia	ETH	Parenteral administration of anti-convulsants	18.8	16.1
Ethiopia	ETH	Parenteral administration of uterotonics	23.4	20.1
Ethiopia	ETH	Parenteral administration of antibiotics	19.6	16.8
Ethiopia	ETH	Assisted vaginal delivery	6.6	5.7
Ethiopia	ETH	Manual removal of placenta	9.8	8.4
Ethiopia	ETH	Removal of retained products of conception	8.7	7.5
Ethiopia	ETH	Cesarean delivery	2.3	2.0
Ethiopia	ETH	Blood transfusion	3.3	2.8
Ethiopia	ETH	Induction of labor for pregnancies lasting 41+ weeks	0.5	0.4
Ethiopia	ETH	Complementary feeding - education only	13.8	11.8
Ethiopia	ETH	Complementary feeding - supplementary feeding and education	13.8	11.8
Ethiopia	ETH	Vitamin A supplementation	77.0	66.0
Ethiopia	ETH	Improved sanitation - Utilization of latrines or toilets	7.3	7.3
Ethiopia	ETH	Improved water source	41.1	41.1
Ethiopia	ETH	Water connection in the home	14.8	14.8
Ethiopia	ETH	Hand washing with soap	8.3	8.3
Ethiopia	ETH	Hygienic disposal of children's stools	36.9	36.9
Ethiopia	ETH	ITN/IRS - Households protected from malaria	70.5	60.4
Ethiopia	ETH	Injectable antibiotics for neonatal sepsis	26.2	21.3
Ethiopia	ETH	ORS - oral rehydration solution	29.5	25.3

Ethiopia	ETH	Antibiotics for treatment of dysentery	9.3	8.0
Ethiopia	ETH	Zinc for treatment of diarrhea	33.3	28.6
Ethiopia	ETH	Oral antibiotics for pneumonia	29.4	25.2
Ethiopia	ETH	Vitamin A for treatment of measles	77.0	66.0
Ethiopia	ETH	SAM - treatment for severe acute malnutrition	12.0	10.3
Ethiopia	ETH	BCG vaccine	85.0	69.2
Ethiopia	ETH	Polio vaccine	67.0	54.6
Ethiopia	ETH	DPT vaccine	72.0	58.6
Ethiopia	ETH	H. influenzae type b vaccine	72.0	67.7
Ethiopia	ETH	HepB vaccine	72.0	58.6
Ethiopia	ETH	Pneumococcal vaccine	67.0	63.0
Ethiopia	ETH	Rotavirus vaccine	79.0	74.3
Ethiopia	ETH	Measles vaccine	61.0	49.7
Ethiopia	ETH	Global wasting (<-2 SD) rate	10.0	11.0
Ethiopia	ETH	Contraceptive prevalence (CPR)	39.95	36.1
Gabon	GAB	TT - Tetanus toxoid vaccination	85.0	69.2
Gabon	GAB	IPTp - Intermittent preventive treatment of malaria during pregnancy	13.1	10.7
Gabon	GAB	Syphilis detection and treatment	23.4	19.1
Gabon	GAB	Iron supplementation in pregnancy	56.8	46.3
Gabon	GAB	Hypertensive disorder case management	18.5	15.1
Gabon	GAB	Diabetes case management	14.5	11.8
Gabon	GAB	Malaria case management	59.8	48.7
Gabon	GAB	MgSO4 management of pre-eclampsia	36.7	29.9
Gabon	GAB	Thermal protection	89.2	76.5
Gabon	GAB	Clean cord care	86.1	73.8
Gabon	GAB	Clean birth environment	74.0	63.4
Gabon	GAB	Immediate drying and additional stimulation	82.6	70.8
Gabon	GAB	Neonatal resuscitation	49.6	42.5
Gabon	GAB	Antibiotics for preterm or prolonged PROM	67.5	57.9
Gabon	GAB	Parenteral administration of anti-convulsants	64.5	55.3
Gabon	GAB	Parenteral administration of uterotonics	80.6	69.1
Gabon	GAB	Parenteral administration of antibiotics	67.5	57.9
Gabon	GAB	Assisted vaginal delivery	22.8	19.5
Gabon	GAB	Manual removal of placenta	33.7	28.9
Gabon	GAB	Removal of retained products of conception	29.9	25.6

Gabon	GAB	Cesarean delivery	7.9	6.8
Gabon	GAB	Blood transfusion	11.4	9.8
Gabon	GAB	Induction of labor for pregnancies lasting 41+ weeks	1.6	1.4
Gabon	GAB	Complementary feeding - education only	29.9	25.6
Gabon	GAB	Complementary feeding - supplementary feeding and education	29.9	25.6
Gabon	GAB	Improved sanitation - Utilization of latrines or toilets	47.4	47.4
Gabon	GAB	Improved water source	85.8	85.8
Gabon	GAB	Water connection in the home	74.4	74.4
Gabon	GAB	ITN/IRS - Households protected from malaria	38.9	33.4
Gabon	GAB	Injectable antibiotics for neonatal sepsis	90.2	73.5
Gabon	GAB	ORS - oral rehydration solution	26.1	22.4
Gabon	GAB	Antibiotics for treatment of dysentery	0.5	0.4
Gabon	GAB	Oral antibiotics for pneumonia	49.5	42.4
Gabon	GAB	ACTs- Artemisinin compounds for treatment of malaria	4.4	3.8
Gabon	GAB	BCG vaccine	87.0	70.9
Gabon	GAB	Polio vaccine	64.0	52.1
Gabon	GAB	DPT vaccine	70.0	57.0
Gabon	GAB	H. influenzae type b vaccine	70.0	65.8
Gabon	GAB	HepB vaccine	70.0	57.0
Gabon	GAB	Measles vaccine	59.0	48.1
Gabon	GAB	Global wasting (<-2 SD) rate	3.4	3.8
Gabon	GAB	Contraceptive prevalence (CPR)	37.15	33.5
Gambia	GMB	TT - Tetanus toxoid vaccination	92.0	74.9
Gambia	GMB	IPTp - Intermittent preventive treatment of malaria during pregnancy	75.2	61.3
Gambia	GMB	Syphilis detection and treatment	24.5	20.0
Gambia	GMB	Iron supplementation in pregnancy	44.6	36.3
Gambia	GMB	Hypertensive disorder case management	18.2	14.8
Gambia	GMB	Diabetes case management	14.2	11.6
Gambia	GMB	Malaria case management	58.6	47.7
Gambia	GMB	MgSO4 management of pre-eclampsia	35.9	29.2
Gambia	GMB	Thermal protection	80.6	69.1
Gambia	GMB	Clean cord care	77.8	66.7

Gambia	GMB	Clean birth environment	66.8	57.3
Gambia	GMB	Immediate drying and additional stimulation	74.6	64.0
Gambia	GMB	Neonatal resuscitation	44.8	38.4
Gambia	GMB	Antibiotics for preterm or prolonged PROM	61.0	52.3
Gambia	GMB	Parenteral administration of anti-convulsants	58.3	50.0
Gambia	GMB	Parenteral administration of uterotonics	72.8	62.4
Gambia	GMB	Parenteral administration of antibiotics	61.0	52.3
Gambia	GMB	Assisted vaginal delivery	20.6	17.7
Gambia	GMB	Manual removal of placenta	30.5	26.1
Gambia	GMB	Removal of retained products of conception	27.0	23.1
Gambia	GMB	Cesarean delivery	7.1	6.1
Gambia	GMB	Blood transfusion	10.3	8.8
Gambia	GMB	Induction of labor for pregnancies lasting 41+ weeks	1.4	1.2
Gambia	GMB	Complementary feeding - education only	13.0	11.1
Gambia	GMB	Complementary feeding - supplementary feeding and education	13.0	11.1
Gambia	GMB	Vitamin A supplementation	32.0	27.4
Gambia	GMB	Improved sanitation - Utilization of latrines or toilets	39.2	39.2
Gambia	GMB	Improved water source	78.0	78.0
Gambia	GMB	Water connection in the home	56.1	56.1
Gambia	GMB	Hand washing with soap	6.3	6.3
Gambia	GMB	Hygienic disposal of children's stools	76.8	76.8
Gambia	GMB	ITN/IRS - Households protected from malaria	71.2	61.0
Gambia	GMB	Injectable antibiotics for neonatal sepsis	81.5	66.4
Gambia	GMB	ORS - oral rehydration solution	43.9	37.6
Gambia	GMB	Antibiotics for treatment of dysentery	35.7	30.6
Gambia	GMB	Zinc for treatment of diarrhea	23.1	19.8
Gambia	GMB	Oral antibiotics for pneumonia	68.0	58.3
Gambia	GMB	Vitamin A for treatment of measles	32.0	27.4
Gambia	GMB	ACTs- Artemisinin compounds for treatment of malaria	0.8	0.7
Gambia	GMB	SAM - treatment for severe acute malnutrition	25.1	21.5
Gambia	GMB	BCG vaccine	94.0	76.6
Gambia	GMB	Polio vaccine	93.0	75.7
Gambia	GMB	DPT vaccine	93.0	75.7

Gambia	GMB	H. influenzae type b vaccine	93.0	87.5
Gambia	GMB	HepB vaccine	93.0	75.7
Gambia	GMB	Pneumococcal vaccine	93.0	87.5
Gambia	GMB	Rotavirus vaccine	93.0	87.5
Gambia	GMB	Measles vaccine	91.0	74.1
Gambia	GMB	Global wasting (<-2 SD) rate	10.8	11.9
Gambia	GMB	Contraceptive prevalence (CPR)	14.25	12.9
Georgia	GEO	Safe abortion services	40.0	36.1
Georgia	GEO	Syphilis detection and treatment	20.8	16.9
Georgia	GEO	Hypertensive disorder case management	20.2	16.5
Georgia	GEO	Diabetes case management	15.8	12.9
Georgia	GEO	Malaria case management	65.3	53.2
Georgia	GEO	MgSO4 management of pre-eclampsia	40.0	32.6
Georgia	GEO	Thermal protection	98.2	84.2
Georgia	GEO	Clean cord care	94.9	81.4
Georgia	GEO	Clean birth environment	81.5	69.9
Georgia	GEO	Immediate drying and additional stimulation	91.0	78.0
Georgia	GEO	Neonatal resuscitation	54.7	46.9
Georgia	GEO	Antibiotics for preterm or prolonged PROM	74.4	63.8
Georgia	GEO	Parenteral administration of anti-convulsants	71.1	61.0
Georgia	GEO	Parenteral administration of uterotonics	88.8	76.1
Georgia	GEO	Parenteral administration of antibiotics	74.4	63.8
Georgia	GEO	Assisted vaginal delivery	25.1	21.5
Georgia	GEO	Manual removal of placenta	37.2	31.9
Georgia	GEO	Removal of retained products of conception	32.9	28.2
Georgia	GEO	Cesarean delivery	8.7	7.5
Georgia	GEO	Blood transfusion	12.5	10.7
Georgia	GEO	Induction of labor for pregnancies lasting 41+ weeks	1.7	1.5
Georgia	GEO	Complementary feeding - education only	49.9	42.8
Georgia	GEO	Complementary feeding - supplementary feeding and education	49.9	42.8
Georgia	GEO	Improved sanitation - Utilization of latrines or toilets	90.0	90.0
Georgia	GEO	Improved water source	98.4	98.4
Georgia	GEO	Water connection in the home	79.1	79.1
Georgia	GEO	Hygienic disposal of children's stools	57.1	57.1

Georgia	GEO	Injectable antibiotics for neonatal sepsis	99.4	81.0
Georgia	GEO	ORS - oral rehydration solution	42.4	36.4
Georgia	GEO	Zinc for treatment of diarrhea	10.9	9.3
Georgia	GEO	Oral antibiotics for pneumonia	73.6	63.1
Georgia	GEO	BCG vaccine	97.0	79.0
Georgia	GEO	Polio vaccine	93.0	75.7
Georgia	GEO	DPT vaccine	93.0	75.7
Georgia	GEO	H. influenzae type b vaccine	93.0	87.5
Georgia	GEO	HepB vaccine	93.0	75.7
Georgia	GEO	Pneumococcal vaccine	81.0	76.2
Georgia	GEO	Rotavirus vaccine	79.0	74.3
Georgia	GEO	Measles vaccine	98.0	79.8
Georgia	GEO	Global wasting (<-2 SD) rate	3.0	3.3
Georgia	GEO	Contraceptive prevalence (CPR)	47.3	42.7
Ghana	GHA	TT - Tetanus toxoid vaccination	89.0	72.5
Ghana	GHA	IPTp - Intermittent preventive treatment of malaria during pregnancy	78.0	63.5
Ghana	GHA	Syphilis detection and treatment	24.1	19.6
Ghana	GHA	Iron supplementation in pregnancy	59.4	48.4
Ghana	GHA	Hypertensive disorder case management	21.5	17.5
Ghana	GHA	Diabetes case management	16.7	13.6
Ghana	GHA	Malaria case management	69.2	56.4
Ghana	GHA	MgSO4 management of pre-eclampsia	42.4	34.5
Ghana	GHA	Thermal protection	77.8	66.7
Ghana	GHA	Clean cord care	75.1	64.4
Ghana	GHA	Clean birth environment	64.5	55.3
Ghana	GHA	Immediate drying and additional stimulation	72.1	61.8
Ghana	GHA	Neonatal resuscitation	43.3	37.1
Ghana	GHA	Antibiotics for preterm or prolonged PROM	58.9	50.5
Ghana	GHA	Parenteral administration of anti-convulsants	56.3	48.3
Ghana	GHA	Parenteral administration of uterotonics	70.3	60.3
Ghana	GHA	Parenteral administration of antibiotics	58.9	50.5
Ghana	GHA	Assisted vaginal delivery	19.9	17.1
Ghana	GHA	Manual removal of placenta	29.4	25.2
Ghana	GHA	Removal of retained products of conception	26.1	22.4
Ghana	GHA	Cesarean delivery	6.8	5.8

Ghana	GHA	Blood transfusion	9.9	8.5
Ghana	GHA	Induction of labor for pregnancies lasting 41+ weeks	1.4	1.2
Ghana	GHA	Complementary feeding - education only	28.1	24.1
Ghana	GHA	Complementary feeding - supplementary feeding and education	28.1	24.1
Ghana	GHA	Vitamin A supplementation	50.0	42.9
Ghana	GHA	Improved sanitation - Utilization of latrines or toilets	18.5	18.5
Ghana	GHA	Improved water source	81.5	81.5
Ghana	GHA	Water connection in the home	26.6	26.6
Ghana	GHA	Hand washing with soap	23.6	23.6
Ghana	GHA	Hygienic disposal of children's stools	24.5	24.5
Ghana	GHA	ITN/IRS - Households protected from malaria	73.0	62.6
Ghana	GHA	Injectable antibiotics for neonatal sepsis	78.7	64.1
Ghana	GHA	ORS - oral rehydration solution	48.6	41.7
Ghana	GHA	Antibiotics for treatment of dysentery	42.4	36.4
Ghana	GHA	Zinc for treatment of diarrhea	7.4	6.3
Ghana	GHA	Oral antibiotics for pneumonia	52.6	45.1
Ghana	GHA	Vitamin A for treatment of measles	50.0	42.9
Ghana	GHA	ACTs- Artemisinin compounds for treatment of malaria	26.2	22.5
Ghana	GHA	BCG vaccine	98.0	79.8
Ghana	GHA	Polio vaccine	98.0	79.8
Ghana	GHA	DPT vaccine	97.0	79.0
Ghana	GHA	H. influenzae type b vaccine	97.0	91.2
Ghana	GHA	HepB vaccine	97.0	79.0
Ghana	GHA	Pneumococcal vaccine	96.0	90.3
Ghana	GHA	Rotavirus vaccine	94.0	88.4
Ghana	GHA	Meningococcal A	83.0	67.6
Ghana	GHA	Measles vaccine	92.0	74.9
Ghana	GHA	Global wasting (<-2 SD) rate	4.7	5.2
Ghana	GHA	Contraceptive prevalence (CPR)	32.5	29.3
Guatemala	GTM	Safe abortion services	0.6	0.5
Guatemala	GTM	TT - Tetanus toxoid vaccination	90.0	73.3
Guatemala	GTM	Syphilis detection and treatment	22.6	18.4
Guatemala	GTM	Iron supplementation in pregnancy	29.3	23.9

Guatemala	GTM	Hypertensive disorder case management	20.6	16.8
Guatemala	GTM	Diabetes case management	16.1	13.1
Guatemala	GTM	Malaria case management	66.6	54.2
Guatemala	GTM	MgSO4 management of pre-eclampsia	40.8	33.2
Guatemala	GTM	Thermal protection	64.2	55.0
Guatemala	GTM	Clean cord care	62.0	53.2
Guatemala	GTM	Clean birth environment	53.3	45.7
Guatemala	GTM	Immediate drying and additional stimulation	59.5	51.0
Guatemala	GTM	Neonatal resuscitation	35.7	30.6
Guatemala	GTM	Antibiotics for preterm or prolonged PROM	48.6	41.7
Guatemala	GTM	Parenteral administration of anti-convulsants	46.5	39.9
Guatemala	GTM	Parenteral administration of uterotonics	58.1	49.8
Guatemala	GTM	Parenteral administration of antibiotics	48.6	41.7
Guatemala	GTM	Assisted vaginal delivery	16.4	14.1
Guatemala	GTM	Manual removal of placenta	24.3	20.8
Guatemala	GTM	Removal of retained products of conception	21.5	18.4
Guatemala	GTM	Cesarean delivery	5.7	4.9
Guatemala	GTM	Blood transfusion	8.2	7.0
Guatemala	GTM	Induction of labor for pregnancies lasting 41+ weeks	1.1	0.9
Guatemala	GTM	Complementary feeding - education only	62.6	53.7
Guatemala	GTM	Complementary feeding - supplementary feeding and education	62.6	53.7
Guatemala	GTM	Vitamin A supplementation	26.0	22.3
Guatemala	GTM	Improved sanitation - Utilization of latrines or toilets	65.1	65.1
Guatemala	GTM	Improved water source	94.2	94.2
Guatemala	GTM	Water connection in the home	75.5	75.5
Guatemala	GTM	Hand washing with soap	78.5	78.5
Guatemala	GTM	Hygienic disposal of children's stools	38.2	38.2
Guatemala	GTM	Injectable antibiotics for neonatal sepsis	65.0	52.9
Guatemala	GTM	ORS - oral rehydration solution	48.8	41.8
Guatemala	GTM	Antibiotics for treatment of dysentery	7.0	6.0
Guatemala	GTM	Zinc for treatment of diarrhea	1.3	1.1
Guatemala	GTM	Oral antibiotics for pneumonia	52.0	44.6
Guatemala	GTM	Vitamin A for treatment of measles	26.0	22.3
Guatemala	GTM	SAM - treatment for severe acute malnutrition	1.4	1.2

Guatemala	GTM	BCG vaccine	88.0	71.7
Guatemala	GTM	Polio vaccine	85.0	69.2
Guatemala	GTM	DPT vaccine	86.0	70.0
Guatemala	GTM	H. influenzae type b vaccine	86.0	80.9
Guatemala	GTM	HepB vaccine	86.0	70.0
Guatemala	GTM	Pneumococcal vaccine	85.0	80.0
Guatemala	GTM	Rotavirus vaccine	87.0	81.8
Guatemala	GTM	Measles vaccine	86.0	70.0
Guatemala	GTM	Global wasting (<-2 SD) rate	0.8	0.8
Guatemala	GTM	Contraceptive prevalence (CPR)	63.55	57.4
Guinea	GIN	TT - Tetanus toxoid vaccination	80.0	65.2
Guinea	GIN	IPTp - Intermittent preventive treatment of malaria during pregnancy	62.7	51.1
Guinea	GIN	Syphilis detection and treatment	20.6	16.8
Guinea	GIN	Iron supplementation in pregnancy	41.5	33.8
Guinea	GIN	Hypertensive disorder case management	8.5	6.9
Guinea	GIN	Diabetes case management	6.6	5.4
Guinea	GIN	Malaria case management	27.4	22.3
Guinea	GIN	MgSO4 management of pre-eclampsia	16.8	13.7
Guinea	GIN	Thermal protection	52.0	44.6
Guinea	GIN	Clean cord care	50.2	43.0
Guinea	GIN	Clean birth environment	43.1	37.0
Guinea	GIN	Immediate drying and additional stimulation	48.2	41.3
Guinea	GIN	Neonatal resuscitation	28.9	24.8
Guinea	GIN	Antibiotics for preterm or prolonged PROM	39.4	33.8
Guinea	GIN	Parenteral administration of anti-convulsants	37.6	32.2
Guinea	GIN	Parenteral administration of uterotonics	47.0	40.3
Guinea	GIN	Parenteral administration of antibiotics	39.4	33.8
Guinea	GIN	Assisted vaginal delivery	13.3	11.4
Guinea	GIN	Manual removal of placenta	19.7	16.9
Guinea	GIN	Removal of retained products of conception	17.4	14.9
Guinea	GIN	Cesarean delivery	4.6	3.9
Guinea	GIN	Blood transfusion	6.6	5.7
Guinea	GIN	Induction of labor for pregnancies lasting 41+ weeks	0.9	0.8
Guinea	GIN	Complementary feeding - education only	15.9	13.6

Guinea	GIN	Complementary feeding - supplementary feeding and education	15.9	13.6
Guinea	GIN	Vitamin A supplementation	64.0	54.9
Guinea	GIN	Improved sanitation - Utilization of latrines or toilets	22.7	22.7
Guinea	GIN	Improved water source	61.9	61.9
Guinea	GIN	Water connection in the home	17.4	17.4
Guinea	GIN	Hand washing with soap	20.9	20.9
Guinea	GIN	Hygienic disposal of children's stools	57.9	57.9
Guinea	GIN	ITN/IRS - Households protected from malaria	43.9	37.6
Guinea	GIN	Injectable antibiotics for neonatal sepsis	52.6	42.8
Guinea	GIN	ORS - oral rehydration solution	54.9	47.1
Guinea	GIN	Antibiotics for treatment of dysentery	1.5	1.3
Guinea	GIN	Zinc for treatment of diarrhea	26.0	22.3
Guinea	GIN	Oral antibiotics for pneumonia	30.1	25.8
Guinea	GIN	Vitamin A for treatment of measles	64.0	54.9
Guinea	GIN	ACTs- Artemisinin compounds for treatment of malaria	2.1	1.8
Guinea	GIN	SAM - treatment for severe acute malnutrition	13.1	11.2
Guinea	GIN	BCG vaccine	72.0	58.6
Guinea	GIN	Polio vaccine	45.0	36.7
Guinea	GIN	DPT vaccine	45.0	36.7
Guinea	GIN	H. influenzae type b vaccine	45.0	42.3
Guinea	GIN	HepB vaccine	45.0	36.7
Guinea	GIN	Measles vaccine	48.0	39.1
Guinea	GIN	Global wasting (<-2 SD) rate	8.2	9.0
Guinea	GIN	Contraceptive prevalence (CPR)	10.3	9.3
Guinea-Bissau	GNB	TT - Tetanus toxoid vaccination	83.0	67.6
Guinea-Bissau	GNB	IPTp - Intermittent preventive treatment of malaria during pregnancy	45.5	37.1
Guinea-Bissau	GNB	Syphilis detection and treatment	22.9	18.7
Guinea-Bissau	GNB	Hypertensive disorder case management	15.6	12.7
Guinea-Bissau	GNB	Diabetes case management	12.2	9.9
Guinea-Bissau	GNB	Malaria case management	50.3	41.0
Guinea-Bissau	GNB	MgSO4 management of pre-eclampsia	30.8	25.1
Guinea-Bissau	GNB	Thermal protection	43.5	37.3
Guinea-Bissau	GNB	Clean cord care	42.0	36.0

Guinea-Bissau	GNB	Clean birth environment	36.1	31.0
Guinea-Bissau	GNB	Immediate drying and additional stimulation	40.3	34.6
Guinea-Bissau	GNB	Neonatal resuscitation	24.2	20.7
Guinea-Bissau	GNB	Antibiotics for preterm or prolonged PROM	32.9	28.2
Guinea-Bissau	GNB	Parenteral administration of anti-convulsants	31.4	26.9
Guinea-Bissau	GNB	Parenteral administration of uterotonics	39.3	33.7
Guinea-Bissau	GNB	Parenteral administration of antibiotics	32.9	28.2
Guinea-Bissau	GNB	Assisted vaginal delivery	11.1	9.5
Guinea-Bissau	GNB	Manual removal of placenta	16.4	14.1
Guinea-Bissau	GNB	Removal of retained products of conception	14.6	12.5
Guinea-Bissau	GNB	Cesarean delivery	3.8	3.3
Guinea-Bissau	GNB	Blood transfusion	5.5	4.7
Guinea-Bissau	GNB	Induction of labor for pregnancies lasting 41+ weeks	0.8	0.7
Guinea-Bissau	GNB	Complementary feeding - education only	12.7	10.9
Guinea-Bissau	GNB	Complementary feeding - supplementary feeding and education	12.7	10.9
Guinea-Bissau	GNB	Vitamin A supplementation	95.0	81.5
Guinea-Bissau	GNB	Improved sanitation - Utilization of latrines or toilets	20.5	20.5
Guinea-Bissau	GNB	Improved water source	66.6	66.6
Guinea-Bissau	GNB	Water connection in the home	11.9	11.9
Guinea-Bissau	GNB	Hand washing with soap	10.6	10.6
Guinea-Bissau	GNB	Hygienic disposal of children's stools	62.6	62.6
Guinea-Bissau	GNB	ITN/IRS - Households protected from malaria	90.1	77.2
Guinea-Bissau	GNB	Injectable antibiotics for neonatal sepsis	44.0	35.8
Guinea-Bissau	GNB	ORS - oral rehydration solution	35.1	30.1
Guinea-Bissau	GNB	Zinc for treatment of diarrhea	27.8	23.8
Guinea-Bissau	GNB	Oral antibiotics for pneumonia	34.3	29.4
Guinea-Bissau	GNB	Vitamin A for treatment of measles	95.0	81.5
Guinea-Bissau	GNB	ACTs- Artemisinin compounds for treatment of malaria	10.0	8.6
Guinea-Bissau	GNB	SAM - treatment for severe acute malnutrition	2.7	2.3
Guinea-Bissau	GNB	BCG vaccine	91.0	74.1
Guinea-Bissau	GNB	Polio vaccine	89.0	72.5
Guinea-Bissau	GNB	DPT vaccine	88.0	71.7
Guinea-Bissau	GNB	H. influenzae type b vaccine	88.0	82.8

Guinea-Bissau	GNB	HepB vaccine	88.0	71.7
Guinea-Bissau	GNB	Pneumococcal vaccine	88.0	82.8
Guinea-Bissau	GNB	Rotavirus vaccine	88.0	82.8
Guinea-Bissau	GNB	Measles vaccine	86.0	70.0
Guinea-Bissau	GNB	Global wasting (<-2 SD) rate	5.9	6.5
Guinea-Bissau	GNB	Contraceptive prevalence (CPR)	19.15	17.3
Guyana	GUY	Safe abortion services	0.2	0.2
Guyana	GUY	TT - Tetanus toxoid vaccination	99.0	80.6
Guyana	GUY	IPTp - Intermittent preventive treatment of malaria during pregnancy	0.1	0.1
Guyana	GUY	Syphilis detection and treatment	22.4	18.2
Guyana	GUY	Iron supplementation in pregnancy	34.2	27.9
Guyana	GUY	Hypertensive disorder case management	20.8	16.9
Guyana	GUY	Diabetes case management	16.2	13.2
Guyana	GUY	Malaria case management	67.2	54.7
Guyana	GUY	MgSO4 management of pre-eclampsia	41.2	33.6
Guyana	GUY	Thermal protection	91.4	78.4
Guyana	GUY	Clean cord care	88.2	75.6
Guyana	GUY	Clean birth environment	75.8	65.0
Guyana	GUY	Immediate drying and additional stimulation	84.6	72.5
Guyana	GUY	Neonatal resuscitation	50.8	43.6
Guyana	GUY	Antibiotics for preterm or prolonged PROM	69.2	59.3
Guyana	GUY	Parenteral administration of anti-convulsants	66.1	56.7
Guyana	GUY	Parenteral administration of uterotonics	82.6	70.8
Guyana	GUY	Parenteral administration of antibiotics	69.2	59.3
Guyana	GUY	Assisted vaginal delivery	23.4	20.1
Guyana	GUY	Manual removal of placenta	34.6	29.7
Guyana	GUY	Removal of retained products of conception	30.6	26.2
Guyana	GUY	Cesarean delivery	8.0	6.9
Guyana	GUY	Blood transfusion	11.6	9.9
Guyana	GUY	Induction of labor for pregnancies lasting 41+ weeks	1.6	1.4
Guyana	GUY	Complementary feeding - education only	52.6	45.1
Guyana	GUY	Complementary feeding - supplementary feeding and education	52.6	45.1
Guyana	GUY	Improved sanitation - Utilization of latrines or toilets	85.8	85.8

Guyana	GUY	Improved water source	95.5	95.5
Guyana	GUY	Water connection in the home	62.4	62.4
Guyana	GUY	Hand washing with soap	78.8	78.8
Guyana	GUY	Hygienic disposal of children's stools	43.0	43.0
Guyana	GUY	ITN/IRS - Households protected from malaria	5.3	4.5
Guyana	GUY	Injectable antibiotics for neonatal sepsis	92.4	75.3
Guyana	GUY	ORS - oral rehydration solution	42.5	36.4
Guyana	GUY	Antibiotics for treatment of dysentery	12.2	10.5
Guyana	GUY	Zinc for treatment of diarrhea	1.3	1.1
Guyana	GUY	Oral antibiotics for pneumonia	83.6	71.7
Guyana	GUY	BCG vaccine	99.0	80.6
Guyana	GUY	Polio vaccine	94.0	76.6
Guyana	GUY	DPT vaccine	95.0	77.4
Guyana	GUY	H. influenzae type b vaccine	95.0	89.4
Guyana	GUY	HepB vaccine	95.0	77.4
Guyana	GUY	Pneumococcal vaccine	91.0	85.6
Guyana	GUY	Rotavirus vaccine	91.0	85.6
Guyana	GUY	Measles vaccine	98.0	79.8
Guyana	GUY	Global wasting (<-2 SD) rate	6.4	7.1
Guyana	GUY	Contraceptive prevalence (CPR)	41.95	37.9
Haiti	HTI	Safe abortion services	53.7	48.5
Haiti	HTI	TT - Tetanus toxoid vaccination	81.0	66.0
Haiti	HTI	IPTp - Intermittent preventive treatment of malaria during pregnancy	0.2	0.2
Haiti	HTI	Syphilis detection and treatment	19.4	15.8
Haiti	HTI	Iron supplementation in pregnancy	43.2	35.2
Haiti	HTI	Hypertensive disorder case management	15.5	12.6
Haiti	HTI	Diabetes case management	15.0	12.2
Haiti	HTI	Malaria case management	32.8	26.7
Haiti	HTI	MgSO4 management of pre-eclampsia	17.1	13.9
Haiti	HTI	Thermal protection	37.8	32.4
Haiti	HTI	Clean cord care	38.2	32.8
Haiti	HTI	Clean birth environment	33.8	29.0
Haiti	HTI	Immediate drying and additional stimulation	32.6	28.0
Haiti	HTI	Neonatal resuscitation	14.9	12.8
Haiti	HTI	Antibiotics for preterm or prolonged PROM	21.4	18.3

Haiti	HTI	Parenteral administration of anti-convulsants	28.2	24.2
Haiti	HTI	Parenteral administration of uterotonics	25.5	21.9
Haiti	HTI	Parenteral administration of antibiotics	21.4	18.3
Haiti	HTI	Assisted vaginal delivery	7.7	6.6
Haiti	HTI	Manual removal of placenta	14.8	12.7
Haiti	HTI	Removal of retained products of conception	11.0	9.4
Haiti	HTI	Cesarean delivery	9.4	8.1
Haiti	HTI	Blood transfusion	5.0	4.3
Haiti	HTI	Induction of labor for pregnancies lasting 41+ weeks	0.6	0.5
Haiti	HTI	Complementary feeding - education only	25.4	21.8
Haiti	HTI	Complementary feeding - supplementary feeding and education	25.4	21.8
Haiti	HTI	Vitamin A supplementation	17.0	14.6
Haiti	HTI	Improved sanitation - Utilization of latrines or toilets	34.7	34.7
Haiti	HTI	Improved water source	65.5	65.5
Haiti	HTI	Water connection in the home	14.8	14.8
Haiti	HTI	Hand washing with soap	21.5	21.5
Haiti	HTI	Hygienic disposal of children's stools	63.9	63.9
Haiti	HTI	ITN/IRS - Households protected from malaria	32.0	27.4
Haiti	HTI	Injectable antibiotics for neonatal sepsis	39.4	32.1
Haiti	HTI	ORS - oral rehydration solution	39.3	33.7
Haiti	HTI	Antibiotics for treatment of dysentery	16.1	13.8
Haiti	HTI	Zinc for treatment of diarrhea	6.2	5.3
Haiti	HTI	Oral antibiotics for pneumonia	39.4	33.8
Haiti	HTI	Vitamin A for treatment of measles	17.0	14.6
Haiti	HTI	SAM - treatment for severe acute malnutrition	18.1	15.5
Haiti	HTI	BCG vaccine	83.0	67.6
Haiti	HTI	Polio vaccine	64.0	52.1
Haiti	HTI	DPT vaccine	64.0	52.1
Haiti	HTI	H. influenzae type b vaccine	64.0	60.2
Haiti	HTI	HepB vaccine	64.0	52.1
Haiti	HTI	Pneumococcal vaccine	1.0	0.9
Haiti	HTI	Rotavirus vaccine	58.0	54.6
Haiti	HTI	Measles vaccine	69.0	56.2
Haiti	HTI	Global wasting (<-2 SD) rate	3.7	4.1

Haiti	HTI	Contraceptive prevalence (CPR)	37.5	33.8
Honduras	HND	Safe abortion services	0.6	0.5
Honduras	HND	TT - Tetanus toxoid vaccination	99.0	80.6
Honduras	HND	Syphilis detection and treatment	23.9	19.5
Honduras	HND	Iron supplementation in pregnancy	37.4	30.5
Honduras	HND	Hypertensive disorder case management	21.2	17.3
Honduras	HND	Diabetes case management	16.6	13.5
Honduras	HND	Malaria case management	68.5	55.8
Honduras	HND	MgSO4 management of pre-eclampsia	42.0	34.2
Honduras	HND	Thermal protection	81.8	70.1
Honduras	HND	Clean cord care	79.0	67.7
Honduras	HND	Clean birth environment	67.9	58.2
Honduras	HND	Immediate drying and additional stimulation	75.8	65.0
Honduras	HND	Neonatal resuscitation	45.5	39.0
Honduras	HND	Antibiotics for preterm or prolonged PROM	61.9	53.1
Honduras	HND	Parenteral administration of anti-convulsants	59.2	50.8
Honduras	HND	Parenteral administration of uterotonics	73.9	63.4
Honduras	HND	Parenteral administration of antibiotics	61.9	53.1
Honduras	HND	Assisted vaginal delivery	20.9	17.9
Honduras	HND	Manual removal of placenta	30.9	26.5
Honduras	HND	Removal of retained products of conception	27.4	23.5
Honduras	HND	Cesarean delivery	7.2	6.2
Honduras	HND	Blood transfusion	10.4	8.9
Honduras	HND	Induction of labor for pregnancies lasting 41+ weeks	1.5	1.3
Honduras	HND	Complementary feeding - education only	68.2	58.5
Honduras	HND	Complementary feeding - supplementary feeding and education	68.2	58.5
Honduras	HND	Vitamin A supplementation	40.0	34.3
Honduras	HND	Improved sanitation - Utilization of latrines or toilets	81.3	81.3
Honduras	HND	Improved water source	94.8	94.8
Honduras	HND	Water connection in the home	85.5	85.5
Honduras	HND	Hand washing with soap	86.3	86.3
Honduras	HND	Hygienic disposal of children's stools	16.1	16.1
Honduras	HND	Injectable antibiotics for neonatal sepsis	82.7	67.4
Honduras	HND	ORS - oral rehydration solution	59.7	51.2

Honduras	HND	Antibiotics for treatment of dysentery	43.6	37.4
Honduras	HND	Zinc for treatment of diarrhea	0.4	0.3
Honduras	HND	Oral antibiotics for pneumonia	63.9	54.8
Honduras	HND	Vitamin A for treatment of measles	40.0	34.3
Honduras	HND	BCG vaccine	94.0	76.6
Honduras	HND	Polio vaccine	90.0	73.3
Honduras	HND	DPT vaccine	90.0	73.3
Honduras	HND	H. influenzae type b vaccine	90.0	84.7
Honduras	HND	HepB vaccine	90.0	73.3
Honduras	HND	Pneumococcal vaccine	90.0	84.7
Honduras	HND	Rotavirus vaccine	91.0	85.6
Honduras	HND	Measles vaccine	89.0	72.5
Honduras	HND	Global wasting (<-2 SD) rate	1.4	1.5
Honduras	HND	Contraceptive prevalence (CPR)	74.9	67.6
India	IND	Safe abortion services	35.1	31.7
India	IND	TT - Tetanus toxoid vaccination	90.0	73.3
India	IND	Syphilis detection and treatment	19.7	16.0
India	IND	Iron supplementation in pregnancy	38.8	31.6
India	IND	Hypertensive disorder case management	12.2	9.9
India	IND	Diabetes case management	9.5	7.7
India	IND	Malaria case management	39.3	32.0
India	IND	MgSO4 management of pre-eclampsia	24.1	19.6
India	IND	Thermal protection	78.0	66.9
India	IND	Clean cord care	75.3	64.6
India	IND	Clean birth environment	64.7	55.5
India	IND	Immediate drying and additional stimulation	72.3	62.0
India	IND	Neonatal resuscitation	43.4	37.2
India	IND	Antibiotics for preterm or prolonged PROM	59.1	50.7
India	IND	Parenteral administration of anti-convulsants	56.4	48.4
India	IND	Parenteral administration of uterotonics	70.5	60.4
India	IND	Parenteral administration of antibiotics	59.1	50.7
India	IND	Assisted vaginal delivery	20.0	17.1
India	IND	Manual removal of placenta	29.5	25.3
India	IND	Removal of retained products of conception	26.2	22.5
India	IND	Cesarean delivery	6.9	5.9

India	IND	Blood transfusion	9.9	8.5
India	IND	Induction of labor for pregnancies lasting 41+ weeks	1.4	1.2
India	IND	Complementary feeding - education only	22.0	18.9
India	IND	Complementary feeding - supplementary feeding and education	22.0	18.9
India	IND	Vitamin A supplementation	71.0	60.9
India	IND	Improved sanitation - Utilization of latrines or toilets	59.5	59.5
India	IND	Improved water source	92.7	92.7
India	IND	Water connection in the home	40.5	40.5
India	IND	Hand washing with soap	67.6	67.6
India	IND	Hygienic disposal of children's stools	26.0	26.0
India	IND	Injectable antibiotics for neonatal sepsis	78.9	64.3
India	IND	ORS - oral rehydration solution	50.6	43.4
India	IND	Antibiotics for treatment of dysentery	21.8	18.7
India	IND	Zinc for treatment of diarrhea	20.3	17.4
India	IND	Oral antibiotics for pneumonia	78.1	67.0
India	IND	Vitamin A for treatment of measles	71.0	60.9
India	IND	ACTs- Artemisinin compounds for treatment of malaria	1.6	1.4
India	IND	BCG vaccine	92.0	74.9
India	IND	Polio vaccine	89.0	72.5
India	IND	DPT vaccine	89.0	72.5
India	IND	H. influenzae type b vaccine	89.0	83.7
India	IND	HepB vaccine	89.0	72.5
India	IND	Pneumococcal vaccine	6.0	5.6
India	IND	Rotavirus vaccine	35.0	32.9
India	IND	Measles vaccine	90.0	73.3
India	IND	Global wasting (<-2 SD) rate	21.0	23.1
India	IND	Contraceptive prevalence (CPR)	56.65	51.1
Indonesia	IDN	Safe abortion services	39.0	35.2
Indonesia	IDN	TT - Tetanus toxoid vaccination	90.0	73.3
Indonesia	IDN	Syphilis detection and treatment	23.7	19.3
Indonesia	IDN	Iron supplementation in pregnancy	32.9	26.8
Indonesia	IDN	Hypertensive disorder case management	21.0	17.1
Indonesia	IDN	Diabetes case management	16.4	13.4

Indonesia	IDN	Malaria case management	67.7	55.1
Indonesia	IDN	MgSO4 management of pre-eclampsia	41.5	33.8
Indonesia	IDN	Thermal protection	62.9	53.9
Indonesia	IDN	Clean cord care	60.7	52.0
Indonesia	IDN	Clean birth environment	52.2	44.8
Indonesia	IDN	Immediate drying and additional stimulation	58.2	49.9
Indonesia	IDN	Neonatal resuscitation	35.0	30.0
Indonesia	IDN	Antibiotics for preterm or prolonged PROM	47.6	40.8
Indonesia	IDN	Parenteral administration of anti-convulsants	45.5	39.0
Indonesia	IDN	Parenteral administration of uterotonics	56.8	48.7
Indonesia	IDN	Parenteral administration of antibiotics	47.6	40.8
Indonesia	IDN	Assisted vaginal delivery	16.1	13.8
Indonesia	IDN	Manual removal of placenta	23.8	20.4
Indonesia	IDN	Removal of retained products of conception	21.1	18.1
Indonesia	IDN	Cesarean delivery	5.5	4.7
Indonesia	IDN	Blood transfusion	8.0	6.9
Indonesia	IDN	Induction of labor for pregnancies lasting 41+ weeks	1.1	0.9
Indonesia	IDN	Complementary feeding - education only	58.2	49.9
Indonesia	IDN	Complementary feeding - supplementary feeding and education	58.2	49.9
Indonesia	IDN	Vitamin A supplementation	62.0	53.2
Indonesia	IDN	Improved sanitation - Utilization of latrines or toilets	73.1	73.1
Indonesia	IDN	Improved water source	89.3	89.3
Indonesia	IDN	Water connection in the home	16.3	16.3
Indonesia	IDN	Hand washing with soap	75.6	75.6
Indonesia	IDN	Hygienic disposal of children's stools	43.7	43.7
Indonesia	IDN	ITN/IRS - Households protected from malaria	3.1	2.7
Indonesia	IDN	Injectable antibiotics for neonatal sepsis	63.6	51.8
Indonesia	IDN	ORS - oral rehydration solution	38.8	33.3
Indonesia	IDN	Antibiotics for treatment of dysentery	19.7	16.9
Indonesia	IDN	Zinc for treatment of diarrhea	1.1	0.9
Indonesia	IDN	Oral antibiotics for pneumonia	75.3	64.6
Indonesia	IDN	Vitamin A for treatment of measles	62.0	53.2
Indonesia	IDN	BCG vaccine	81.0	66.0
Indonesia	IDN	Polio vaccine	80.0	65.2

Indonesia	IDN	DPT vaccine	79.0	64.3
Indonesia	IDN	H. influenzae type b vaccine	79.0	74.3
Indonesia	IDN	HepB vaccine	79.0	64.3
Indonesia	IDN	Pneumococcal vaccine	8.0	7.5
Indonesia	IDN	Measles vaccine	75.0	61.1
Indonesia	IDN	Global wasting (<-2 SD) rate	10.6	11.7
Indonesia	IDN	Contraceptive prevalence (CPR)	63.2	57.0
Iraq	IRQ	Safe abortion services	40.0	36.1
Iraq	IRQ	TT - Tetanus toxoid vaccination	75.0	61.1
Iraq	IRQ	Syphilis detection and treatment	21.7	17.7
Iraq	IRQ	Hypertensive disorder case management	16.3	13.3
Iraq	IRQ	Diabetes case management	12.7	10.3
Iraq	IRQ	Malaria case management	52.7	42.9
Iraq	IRQ	MgSO4 management of pre-eclampsia	32.3	26.3
Iraq	IRQ	Thermal protection	85.6	73.4
Iraq	IRQ	Clean cord care	82.6	70.8
Iraq	IRQ	Clean birth environment	71.0	60.9
Iraq	IRQ	Immediate drying and additional stimulation	79.3	68.0
Iraq	IRQ	Neonatal resuscitation	47.6	40.8
Iraq	IRQ	Antibiotics for preterm or prolonged PROM	64.8	55.6
Iraq	IRQ	Parenteral administration of anti-convulsants	61.9	53.1
Iraq	IRQ	Parenteral administration of uterotonics	77.4	66.4
Iraq	IRQ	Parenteral administration of antibiotics	64.8	55.6
Iraq	IRQ	Assisted vaginal delivery	21.9	18.8
Iraq	IRQ	Manual removal of placenta	32.4	27.8
Iraq	IRQ	Removal of retained products of conception	28.7	24.6
Iraq	IRQ	Cesarean delivery	7.5	6.4
Iraq	IRQ	Blood transfusion	10.9	9.3
Iraq	IRQ	Induction of labor for pregnancies lasting 41+ weeks	1.5	1.3
Iraq	IRQ	Complementary feeding - education only	60.1	51.5
Iraq	IRQ	Complementary feeding - supplementary feeding and education	60.1	51.5
Iraq	IRQ	Improved sanitation - Utilization of latrines or toilets	94.1	94.1
Iraq	IRQ	Improved water source	96.5	96.5
Iraq	IRQ	Water connection in the home	76.8	76.8

Iraq	IRQ	Hand washing with soap	97.7	97.7
Iraq	IRQ	Hygienic disposal of children's stools	15.8	15.8
Iraq	IRQ	Injectable antibiotics for neonatal sepsis	86.6	70.5
Iraq	IRQ	ORS - oral rehydration solution	25.4	21.8
Iraq	IRQ	Zinc for treatment of diarrhea	7.4	6.3
Iraq	IRQ	Oral antibiotics for pneumonia	40.3	34.6
Iraq	IRQ	BCG vaccine	95.0	77.4
Iraq	IRQ	Polio vaccine	71.0	57.8
Iraq	IRQ	DPT vaccine	84.0	68.4
Iraq	IRQ	H. influenzae type b vaccine	84.0	79.0
Iraq	IRQ	HepB vaccine	84.0	68.4
Iraq	IRQ	Pneumococcal vaccine	32.0	30.1
Iraq	IRQ	Rotavirus vaccine	60.0	56.4
Iraq	IRQ	Measles vaccine	83.0	67.6
Iraq	IRQ	Global wasting (<-2 SD) rate	3.1	3.5
Iraq	IRQ	Contraceptive prevalence (CPR)	47.05	42.5
Jamaica	JAM	Safe abortion services	53.7	48.5
Jamaica	JAM	TT - Tetanus toxoid vaccination	90.0	73.3
Jamaica	JAM	Syphilis detection and treatment	24.1	19.6
Jamaica	JAM	Hypertensive disorder case management	20.6	16.8
Jamaica	JAM	Diabetes case management	16.0	13.0
Jamaica	JAM	Malaria case management	66.3	54.0
Jamaica	JAM	MgSO4 management of pre-eclampsia	40.7	33.2
Jamaica	JAM	Thermal protection	97.4	83.5
Jamaica	JAM	Clean cord care	94.0	80.6
Jamaica	JAM	Clean birth environment	80.8	69.3
Jamaica	JAM	Immediate drying and additional stimulation	90.2	77.3
Jamaica	JAM	Neonatal resuscitation	54.2	46.5
Jamaica	JAM	Antibiotics for preterm or prolonged PROM	73.8	63.3
Jamaica	JAM	Parenteral administration of anti-convulsants	70.5	60.4
Jamaica	JAM	Parenteral administration of uterotonics	88.1	75.5
Jamaica	JAM	Parenteral administration of antibiotics	73.8	63.3
Jamaica	JAM	Assisted vaginal delivery	24.9	21.3
Jamaica	JAM	Manual removal of placenta	36.9	31.6
Jamaica	JAM	Removal of retained products of conception	32.7	28.0

Jamaica	JAM	Cesarean delivery	8.6	7.4
Jamaica	JAM	Blood transfusion	12.4	10.6
Jamaica	JAM	Induction of labor for pregnancies lasting 41+ weeks	1.7	1.5
Jamaica	JAM	Improved sanitation - Utilization of latrines or toilets	87.3	87.3
Jamaica	JAM	Improved water source	90.6	90.6
Jamaica	JAM	Water connection in the home	76.0	76.0
Jamaica	JAM	Hand washing with soap	74.7	74.7
Jamaica	JAM	Hygienic disposal of children's stools	28.2	28.2
Jamaica	JAM	Injectable antibiotics for neonatal sepsis	98.6	80.3
Jamaica	JAM	ORS - oral rehydration solution	64.1	55.0
Jamaica	JAM	Oral antibiotics for pneumonia	82.3	70.6
Jamaica	JAM	BCG vaccine	93.0	75.7
Jamaica	JAM	Polio vaccine	98.0	79.8
Jamaica	JAM	DPT vaccine	97.0	79.0
Jamaica	JAM	H. influenzae type b vaccine	98.0	92.2
Jamaica	JAM	HepB vaccine	97.0	79.0
Jamaica	JAM	Measles vaccine	89.0	72.5
Jamaica	JAM	Global wasting (<-2 SD) rate	2.4	2.7
Jamaica	JAM	Contraceptive prevalence (CPR)	70.95	64.0
Jordan	JOR	Safe abortion services	40.0	36.1
Jordan	JOR	TT - Tetanus toxoid vaccination	90.0	73.3
Jordan	JOR	Syphilis detection and treatment	24.2	19.7
Jordan	JOR	Iron supplementation in pregnancy	48.9	39.8
Jordan	JOR	Hypertensive disorder case management	22.2	18.1
Jordan	JOR	Diabetes case management	17.3	14.1
Jordan	JOR	Malaria case management	71.6	58.3
Jordan	JOR	MgSO4 management of pre-eclampsia	43.9	35.8
Jordan	JOR	Thermal protection	96.9	83.1
Jordan	JOR	Clean cord care	93.6	80.3
Jordan	JOR	Clean birth environment	80.4	68.9
Jordan	JOR	Immediate drying and additional stimulation	89.8	77.0
Jordan	JOR	Neonatal resuscitation	53.9	46.2
Jordan	JOR	Antibiotics for preterm or prolonged PROM	73.4	62.9
Jordan	JOR	Parenteral administration of anti-convulsants	70.1	60.1

Jordan	JOR	Parenteral administration of uterotonics	87.6	75.1
Jordan	JOR	Parenteral administration of antibiotics	73.4	62.9
Jordan	JOR	Assisted vaginal delivery	24.8	21.3
Jordan	JOR	Manual removal of placenta	36.7	31.5
Jordan	JOR	Removal of retained products of conception	32.5	27.9
Jordan	JOR	Cesarean delivery	8.5	7.3
Jordan	JOR	Blood transfusion	12.3	10.5
Jordan	JOR	Induction of labor for pregnancies lasting 41+ weeks	1.7	1.5
Jordan	JOR	Complementary feeding - education only	51.4	44.1
Jordan	JOR	Complementary feeding - supplementary feeding and education	51.4	44.1
Jordan	JOR	Vitamin A supplementation	17.0	14.6
Jordan	JOR	Improved sanitation - Utilization of latrines or toilets	97.3	97.3
Jordan	JOR	Improved water source	98.9	98.9
Jordan	JOR	Water connection in the home	86.9	86.9
Jordan	JOR	Injectable antibiotics for neonatal sepsis	98.1	79.9
Jordan	JOR	ORS - oral rehydration solution	44.4	38.1
Jordan	JOR	Antibiotics for treatment of dysentery	54.5	46.7
Jordan	JOR	Oral antibiotics for pneumonia	60.9	52.2
Jordan	JOR	Vitamin A for treatment of measles	17.0	14.6
Jordan	JOR	BCG vaccine	94.0	76.6
Jordan	JOR	Polio vaccine	92.0	74.9
Jordan	JOR	DPT vaccine	96.0	78.2
Jordan	JOR	H. influenzae type b vaccine	96.0	90.3
Jordan	JOR	HepB vaccine	96.0	78.2
Jordan	JOR	Rotavirus vaccine	93.0	87.5
Jordan	JOR	Measles vaccine	92.0	74.9
Jordan	JOR	Global wasting (<-2 SD) rate	2.5	2.7
Jordan	JOR	Contraceptive prevalence (CPR)	53.2	48.0
Kazakhstan	KAZ	Safe abortion services	35.1	31.7
Kazakhstan	KAZ	Syphilis detection and treatment	24.5	20.0
Kazakhstan	KAZ	Hypertensive disorder case management	22.8	18.6
Kazakhstan	KAZ	Diabetes case management	17.8	14.5
Kazakhstan	KAZ	Malaria case management	73.7	60.0
Kazakhstan	KAZ	MgSO4 management of pre-eclampsia	45.2	36.8

Kazakhstan	KAZ	Thermal protection	98.2	84.2
Kazakhstan	KAZ	Clean cord care	94.8	81.3
Kazakhstan	KAZ	Clean birth environment	81.5	69.9
Kazakhstan	KAZ	Immediate drying and additional stimulation	91.0	78.0
Kazakhstan	KAZ	Neonatal resuscitation	54.6	46.8
Kazakhstan	KAZ	Antibiotics for preterm or prolonged PROM	74.3	63.7
Kazakhstan	KAZ	Parenteral administration of anti-convulsants	71.0	60.9
Kazakhstan	KAZ	Parenteral administration of uterotonics	88.8	76.1
Kazakhstan	KAZ	Parenteral administration of antibiotics	74.3	63.7
Kazakhstan	KAZ	Assisted vaginal delivery	25.1	21.5
Kazakhstan	KAZ	Manual removal of placenta	37.2	31.9
Kazakhstan	KAZ	Removal of retained products of conception	32.9	28.2
Kazakhstan	KAZ	Cesarean delivery	8.6	7.4
Kazakhstan	KAZ	Blood transfusion	12.5	10.7
Kazakhstan	KAZ	Induction of labor for pregnancies lasting 41+ weeks	1.7	1.5
Kazakhstan	KAZ	Complementary feeding - education only	64.2	55.0
Kazakhstan	KAZ	Complementary feeding - supplementary feeding and education	64.2	55.0
Kazakhstan	KAZ	Improved sanitation - Utilization of latrines or toilets	97.9	97.9
Kazakhstan	KAZ	Improved water source	95.6	95.6
Kazakhstan	KAZ	Water connection in the home	77.3	77.3
Kazakhstan	KAZ	Hand washing with soap	99.0	99.0
Kazakhstan	KAZ	Hygienic disposal of children's stools	66.7	66.7
Kazakhstan	KAZ	Injectable antibiotics for neonatal sepsis	99.3	80.9
Kazakhstan	KAZ	ORS - oral rehydration solution	61.8	53.0
Kazakhstan	KAZ	Antibiotics for treatment of dysentery	17.2	14.7
Kazakhstan	KAZ	Oral antibiotics for pneumonia	81.2	69.6
Kazakhstan	KAZ	BCG vaccine	95.0	77.4
Kazakhstan	KAZ	Polio vaccine	98.0	79.8
Kazakhstan	KAZ	DPT vaccine	98.0	79.8
Kazakhstan	KAZ	H. influenzae type b vaccine	98.0	92.2
Kazakhstan	KAZ	HepB vaccine	98.0	79.8
Kazakhstan	KAZ	Pneumococcal vaccine	95.0	89.4
Kazakhstan	KAZ	Measles vaccine	99.0	80.6
Kazakhstan	KAZ	Global wasting (<-2 SD) rate	3.3	3.6

Kazakhstan	KAZ	Contraceptive prevalence (CPR)	56.2	50.7
Kenya	KEN	Safe abortion services	3.3	3.0
Kenya	KEN	TT - Tetanus toxoid vaccination	88.0	71.7
Kenya	KEN	IPTp - Intermittent preventive treatment of malaria during pregnancy	34.7	28.3
Kenya	KEN	Syphilis detection and treatment	52.9	43.1
Kenya	KEN	Iron supplementation in pregnancy	7.5	6.1
Kenya	KEN	Hypertensive disorder case management	14.0	11.4
Kenya	KEN	Diabetes case management	12.5	10.2
Kenya	KEN	Malaria case management	32.7	26.6
Kenya	KEN	MgSO4 management of pre-eclampsia	24.6	20.0
Kenya	KEN	Thermal protection	60.7	52.0
Kenya	KEN	Clean cord care	58.7	50.3
Kenya	KEN	Clean birth environment	50.4	43.2
Kenya	KEN	Immediate drying and additional stimulation	53.6	46.0
Kenya	KEN	Neonatal resuscitation	50.9	43.6
Kenya	KEN	Antibiotics for preterm or prolonged PROM	47.0	40.3
Kenya	KEN	Parenteral administration of anti-convulsants	60.0	51.4
Kenya	KEN	Parenteral administration of uterotonics	46.6	40.0
Kenya	KEN	Parenteral administration of antibiotics	47.0	40.3
Kenya	KEN	Assisted vaginal delivery	5.2	4.5
Kenya	KEN	Manual removal of placenta	42.8	36.7
Kenya	KEN	Removal of retained products of conception	31.3	26.8
Kenya	KEN	Cesarean delivery	11.3	9.7
Kenya	KEN	Blood transfusion	7.7	6.6
Kenya	KEN	Induction of labor for pregnancies lasting 41+ weeks	1.1	0.9
Kenya	KEN	Complementary feeding - education only	40.9	35.1
Kenya	KEN	Complementary feeding - supplementary feeding and education	40.9	35.1
Kenya	KEN	Vitamin A supplementation	44.0	37.7
Kenya	KEN	Improved sanitation - Utilization of latrines or toilets	29.1	29.1
Kenya	KEN	Improved water source	58.9	58.9
Kenya	KEN	Water connection in the home	18.9	18.9
Kenya	KEN	Hand washing with soap	25.2	25.2
Kenya	KEN	Hygienic disposal of children's stools	70.2	70.2

Kenya	KEN	ITN/IRS - Households protected from malaria	62.5	53.6
Kenya	KEN	Injectable antibiotics for neonatal sepsis	61.5	50.1
Kenya	KEN	ORS - oral rehydration solution	53.8	46.1
Kenya	KEN	Antibiotics for treatment of dysentery	15.7	13.5
Kenya	KEN	Zinc for treatment of diarrhea	8.1	6.9
Kenya	KEN	Oral antibiotics for pneumonia	65.7	56.3
Kenya	KEN	Vitamin A for treatment of measles	44.0	37.7
Kenya	KEN	ACTs- Artemisinin compounds for treatment of malaria	15.0	12.9
Kenya	KEN	SAM - treatment for severe acute malnutrition	7.2	6.2
Kenya	KEN	BCG vaccine	95.0	77.4
Kenya	KEN	Polio vaccine	81.0	66.0
Kenya	KEN	DPT vaccine	92.0	74.9
Kenya	KEN	H. influenzae type b vaccine	92.0	86.5
Kenya	KEN	HepB vaccine	92.0	74.9
Kenya	KEN	Pneumococcal vaccine	81.0	76.2
Kenya	KEN	Rotavirus vaccine	78.0	73.4
Kenya	KEN	Measles vaccine	89.0	72.5
Kenya	KEN	Global wasting (<-2 SD) rate	4.2	4.6
Kenya	KEN	Contraceptive prevalence (CPR)	61.55	55.5
Kyrgyzstan	KGZ	Safe abortion services	35.1	31.7
Kyrgyzstan	KGZ	Syphilis detection and treatment	24.7	20.1
Kyrgyzstan	KGZ	Iron supplementation in pregnancy	2.3	1.9
Kyrgyzstan	KGZ	Hypertensive disorder case management	24.0	19.5
Kyrgyzstan	KGZ	Diabetes case management	18.7	15.2
Kyrgyzstan	KGZ	Malaria case management	77.4	63.0
Kyrgyzstan	KGZ	MgSO4 management of pre-eclampsia	47.4	38.6
Kyrgyzstan	KGZ	Thermal protection	98.4	84.4
Kyrgyzstan	KGZ	Clean cord care	95.0	81.5
Kyrgyzstan	KGZ	Clean birth environment	81.7	70.0
Kyrgyzstan	KGZ	Immediate drying and additional stimulation	91.2	78.2
Kyrgyzstan	KGZ	Neonatal resuscitation	54.8	47.0
Kyrgyzstan	KGZ	Antibiotics for preterm or prolonged PROM	74.5	63.9
Kyrgyzstan	KGZ	Parenteral administration of anti-convulsants	71.2	61.0
Kyrgyzstan	KGZ	Parenteral administration of uterotonics	89.0	76.3
Kyrgyzstan	KGZ	Parenteral administration of antibiotics	74.5	63.9

Kyrgyzstan	KGZ	Assisted vaginal delivery	25.2	21.6
Kyrgyzstan	KGZ	Manual removal of placenta	37.3	32.0
Kyrgyzstan	KGZ	Removal of retained products of conception	33.0	28.3
Kyrgyzstan	KGZ	Cesarean delivery	8.7	7.5
Kyrgyzstan	KGZ	Blood transfusion	12.5	10.7
Kyrgyzstan	KGZ	Induction of labor for pregnancies lasting 41+ weeks	1.8	1.5
Kyrgyzstan	KGZ	Complementary feeding - education only	61.7	52.9
Kyrgyzstan	KGZ	Complementary feeding - supplementary feeding and education	61.7	52.9
Kyrgyzstan	KGZ	Vitamin A supplementation	97.0	83.2
Kyrgyzstan	KGZ	Improved sanitation - Utilization of latrines or toilets	96.5	96.5
Kyrgyzstan	KGZ	Improved water source	87.5	87.5
Kyrgyzstan	KGZ	Water connection in the home	78.1	78.1
Kyrgyzstan	KGZ	Hand washing with soap	98.1	98.1
Kyrgyzstan	KGZ	Hygienic disposal of children's stools	75.8	75.8
Kyrgyzstan	KGZ	Injectable antibiotics for neonatal sepsis	99.6	81.1
Kyrgyzstan	KGZ	ORS - oral rehydration solution	36.4	31.2
Kyrgyzstan	KGZ	Zinc for treatment of diarrhea	21.8	18.7
Kyrgyzstan	KGZ	Oral antibiotics for pneumonia	59.7	51.2
Kyrgyzstan	KGZ	Vitamin A for treatment of measles	97.0	83.2
Kyrgyzstan	KGZ	BCG vaccine	97.0	79.0
Kyrgyzstan	KGZ	Polio vaccine	92.0	74.9
Kyrgyzstan	KGZ	DPT vaccine	94.0	76.6
Kyrgyzstan	KGZ	H. influenzae type b vaccine	92.0	86.5
Kyrgyzstan	KGZ	HepB vaccine	92.0	74.9
Kyrgyzstan	KGZ	Pneumococcal vaccine	92.0	86.5
Kyrgyzstan	KGZ	Measles vaccine	96.0	78.2
Kyrgyzstan	KGZ	Global wasting (<-2 SD) rate	2.8	3.1
Kyrgyzstan	KGZ	Contraceptive prevalence (CPR)	41.6	37.5
Lao People's Democratic Republic	LAO	Safe abortion services	39.0	35.2
Lao People's Democratic Republic	LAO	TT - Tetanus toxoid vaccination	90.0	73.3
Lao People's Democratic Republic	LAO	IPTp - Intermittent preventive treatment of malaria during pregnancy	2.5	2.0

Lao People's Democratic Republic	LAO	Syphilis detection and treatment	19.4	15.8
Lao People's Democratic Republic	LAO	Iron supplementation in pregnancy	55.1	44.9
Lao People's Democratic Republic	LAO	Hypertensive disorder case management	14.9	12.1
Lao People's Democratic Republic	LAO	Diabetes case management	11.7	9.5
Lao People's Democratic Republic	LAO	Malaria case management	48.2	39.3
Lao People's Democratic Republic	LAO	MgSO4 management of pre-eclampsia	29.6	24.1
Lao People's Democratic Republic	LAO	Thermal protection	63.7	54.6
Lao People's Democratic Republic	LAO	Clean cord care	61.5	52.7
Lao People's Democratic Republic	LAO	Clean birth environment	52.8	45.3
Lao People's Democratic Republic	LAO	Immediate drying and additional stimulation	59.0	50.6
Lao People's Democratic Republic	LAO	Neonatal resuscitation	35.4	30.4
Lao People's Democratic Republic	LAO	Antibiotics for preterm or prolonged PROM	48.2	41.3
Lao People's Democratic Republic	LAO	Parenteral administration of anti-convulsants	46.0	39.4
Lao People's Democratic Republic	LAO	Parenteral administration of uterotonics	57.5	49.3
Lao People's Democratic Republic	LAO	Parenteral administration of antibiotics	48.2	41.3
Lao People's Democratic Republic	LAO	Assisted vaginal delivery	16.3	14.0
Lao People's Democratic Republic	LAO	Manual removal of placenta	24.1	20.7
Lao People's Democratic Republic	LAO	Removal of retained products of conception	21.3	18.3
Lao People's Democratic Republic	LAO	Cesarean delivery	5.6	4.8
Lao People's Democratic Republic	LAO	Blood transfusion	8.1	6.9
Lao People's Democratic Republic	LAO	Induction of labor for pregnancies lasting 41+ weeks	1.1	0.9

Lao People's Democratic Republic	LAO	Complementary feeding - education only	33.5	28.7
Lao People's Democratic Republic	LAO	Complementary feeding - supplementary feeding and education	33.5	28.7
Lao People's Democratic Republic	LAO	Vitamin A supplementation	57.0	48.9
Lao People's Democratic Republic	LAO	Improved sanitation - Utilization of latrines or toilets	74.5	74.5
Lao People's Democratic Republic	LAO	Improved water source	82.1	82.1
Lao People's Democratic Republic	LAO	Water connection in the home	40.6	40.6
Lao People's Democratic Republic	LAO	Hand washing with soap	91.8	91.8
Lao People's Democratic Republic	LAO	Hygienic disposal of children's stools	27.9	27.9
Lao People's Democratic Republic	LAO	ITN/IRS - Households protected from malaria	62.5	53.6
Lao People's Democratic Republic	LAO	Injectable antibiotics for neonatal sepsis	64.4	52.5
Lao People's Democratic Republic	LAO	ORS - oral rehydration solution	56.1	48.1
Lao People's Democratic Republic	LAO	Zinc for treatment of diarrhea	15.2	13.0
Lao People's Democratic Republic	LAO	Oral antibiotics for pneumonia	38.5	33.0
Lao People's Democratic Republic	LAO	Vitamin A for treatment of measles	57.0	48.9
Lao People's Democratic Republic	LAO	ACTs- Artemisinin compounds for treatment of malaria	3.5	3.0
Lao People's Democratic Republic	LAO	SAM - treatment for severe acute malnutrition	2.4	2.1
Lao People's Democratic Republic	LAO	BCG vaccine	79.0	64.3
Lao People's Democratic Republic	LAO	Polio vaccine	67.0	54.6
Lao People's Democratic Republic	LAO	DPT vaccine	68.0	55.4
Lao People's Democratic Republic	LAO	H. influenzae type b vaccine	68.0	64.0
Lao People's Democratic Republic	LAO	HepB vaccine	68.0	55.4

Lao People's Democratic Republic	LAO	Pneumococcal vaccine	56.0	52.7
Lao People's Democratic Republic	LAO	Measles vaccine	82.0	66.8
Lao People's Democratic Republic	LAO	Global wasting (<-2 SD) rate	9.1	10.0
Lao People's Democratic Republic	LAO	Contraceptive prevalence (CPR)	57.2	51.6
Lesotho	LSO	Safe abortion services	42.5	38.4
Lesotho	LSO	TT - Tetanus toxoid vaccination	85.0	69.2
Lesotho	LSO	Syphilis detection and treatment	23.5	19.1
Lesotho	LSO	Iron supplementation in pregnancy	51.4	41.9
Lesotho	LSO	Hypertensive disorder case management	18.4	15.0
Lesotho	LSO	Diabetes case management	14.4	11.7
Lesotho	LSO	Malaria case management	59.4	48.4
Lesotho	LSO	MgSO4 management of pre-eclampsia	36.4	29.6
Lesotho	LSO	Thermal protection	88.4	75.8
Lesotho	LSO	Clean cord care	85.3	73.1
Lesotho	LSO	Clean birth environment	73.3	62.8
Lesotho	LSO	Immediate drying and additional stimulation	81.9	70.2
Lesotho	LSO	Neonatal resuscitation	49.2	42.2
Lesotho	LSO	Antibiotics for preterm or prolonged PROM	66.9	57.4
Lesotho	LSO	Parenteral administration of anti-convulsants	63.9	54.8
Lesotho	LSO	Parenteral administration of uterotonics	79.9	68.5
Lesotho	LSO	Parenteral administration of antibiotics	66.9	57.4
Lesotho	LSO	Assisted vaginal delivery	22.6	19.4
Lesotho	LSO	Manual removal of placenta	33.4	28.6
Lesotho	LSO	Removal of retained products of conception	29.6	25.4
Lesotho	LSO	Cesarean delivery	7.8	6.7
Lesotho	LSO	Blood transfusion	11.3	9.7
Lesotho	LSO	Induction of labor for pregnancies lasting 41+ weeks	1.6	1.4
Lesotho	LSO	Complementary feeding - education only	23.1	19.8
Lesotho	LSO	Complementary feeding - supplementary feeding and education	23.1	19.8
Lesotho	LSO	Vitamin A supplementation	18.0	15.4
Lesotho	LSO	Improved sanitation - Utilization of latrines or toilets	42.8	42.8

Lesotho	LSO	Improved water source	68.6	68.6
Lesotho	LSO	Water connection in the home	43.5	43.5
Lesotho	LSO	Hand washing with soap	36.3	36.3
Lesotho	LSO	Hygienic disposal of children's stools	54.6	54.6
Lesotho	LSO	Injectable antibiotics for neonatal sepsis	89.4	72.8
Lesotho	LSO	ORS - oral rehydration solution	39.7	34.0
Lesotho	LSO	Antibiotics for treatment of dysentery	26.1	22.4
Lesotho	LSO	Zinc for treatment of diarrhea	17.4	14.9
Lesotho	LSO	Oral antibiotics for pneumonia	57.5	49.3
Lesotho	LSO	Vitamin A for treatment of measles	18.0	15.4
Lesotho	LSO	SAM - treatment for severe acute malnutrition	6.2	5.3
Lesotho	LSO	BCG vaccine	98.0	79.8
Lesotho	LSO	Polio vaccine	90.0	73.3
Lesotho	LSO	DPT vaccine	93.0	75.7
Lesotho	LSO	H. influenzae type b vaccine	93.0	87.5
Lesotho	LSO	HepB vaccine	93.0	75.7
Lesotho	LSO	Pneumococcal vaccine	93.0	87.5
Lesotho	LSO	Rotavirus vaccine	70.0	65.8
Lesotho	LSO	Measles vaccine	90.0	73.3
Lesotho	LSO	Global wasting (<-2 SD) rate	3.0	3.3
Lesotho	LSO	Contraceptive prevalence (CPR)	65.45	59.1
Liberia	LBR	TT - Tetanus toxoid vaccination	89.0	72.5
Liberia	LBR	IPTp - Intermittent preventive treatment of malaria during pregnancy	54.5	44.4
Liberia	LBR	Syphilis detection and treatment	23.7	19.3
Liberia	LBR	Iron supplementation in pregnancy	21.2	17.3
Liberia	LBR	Hypertensive disorder case management	18.6	15.1
Liberia	LBR	Diabetes case management	14.5	11.8
Liberia	LBR	Malaria case management	60.1	49.0
Liberia	LBR	MgSO4 management of pre-eclampsia	36.9	30.1
Liberia	LBR	Thermal protection	55.2	47.3
Liberia	LBR	Clean cord care	53.3	45.7
Liberia	LBR	Clean birth environment	45.8	39.3
Liberia	LBR	Immediate drying and additional stimulation	51.1	43.8
Liberia	LBR	Neonatal resuscitation	30.7	26.3
Liberia	LBR	Antibiotics for preterm or prolonged PROM	41.8	35.8

Liberia	LBR	Parenteral administration of anti-convulsants	39.9	34.2
Liberia	LBR	Parenteral administration of uterotonics	49.9	42.8
Liberia	LBR	Parenteral administration of antibiotics	41.8	35.8
Liberia	LBR	Assisted vaginal delivery	14.1	12.1
Liberia	LBR	Manual removal of placenta	20.9	17.9
Liberia	LBR	Removal of retained products of conception	18.5	15.9
Liberia	LBR	Cesarean delivery	4.9	4.2
Liberia	LBR	Blood transfusion	7.0	6.0
Liberia	LBR	Induction of labor for pregnancies lasting 41+ weeks	1.0	0.9
Liberia	LBR	Complementary feeding - education only	13.5	11.6
Liberia	LBR	Complementary feeding - supplementary feeding and education	13.5	11.6
Liberia	LBR	Vitamin A supplementation	97.0	83.2
Liberia	LBR	Improved sanitation - Utilization of latrines or toilets	17.0	17.0
Liberia	LBR	Improved water source	72.9	72.9
Liberia	LBR	Water connection in the home	3.4	3.4
Liberia	LBR	Hand washing with soap	3.8	3.8
Liberia	LBR	Hygienic disposal of children's stools	23.2	23.2
Liberia	LBR	ITN/IRS - Households protected from malaria	62.1	53.2
Liberia	LBR	Injectable antibiotics for neonatal sepsis	55.8	45.4
Liberia	LBR	ORS - oral rehydration solution	60.4	51.8
Liberia	LBR	Antibiotics for treatment of dysentery	18.7	16.0
Liberia	LBR	Zinc for treatment of diarrhea	3.1	2.7
Liberia	LBR	Oral antibiotics for pneumonia	50.7	43.5
Liberia	LBR	Vitamin A for treatment of measles	97.0	83.2
Liberia	LBR	ACTs- Artemisinin compounds for treatment of malaria	16.7	14.3
Liberia	LBR	SAM - treatment for severe acute malnutrition	15.1	12.9
Liberia	LBR	BCG vaccine	92.0	74.9
Liberia	LBR	Polio vaccine	84.0	68.4
Liberia	LBR	DPT vaccine	84.0	68.4
Liberia	LBR	H. influenzae type b vaccine	84.0	79.0
Liberia	LBR	HepB vaccine	84.0	68.4
Liberia	LBR	Pneumococcal vaccine	84.0	79.0
Liberia	LBR	Rotavirus vaccine	74.0	69.6

Liberia	LBR	Measles vaccine	87.0	70.9
Liberia	LBR	Global wasting (<-2 SD) rate	5.4	5.9
Liberia	LBR	Contraceptive prevalence (CPR)	30.45	27.5
Madagascar	MDG	Safe abortion services	3.3	3.0
Madagascar	MDG	TT - Tetanus toxoid vaccination	78.0	63.5
Madagascar	MDG	IPTp - Intermittent preventive treatment of malaria during pregnancy	22.3	18.2
Madagascar	MDG	Syphilis detection and treatment	12.6	10.3
Madagascar	MDG	Iron supplementation in pregnancy	7.6	6.2
Madagascar	MDG	Hypertensive disorder case management	11.3	9.2
Madagascar	MDG	Diabetes case management	8.8	7.2
Madagascar	MDG	Malaria case management	36.6	29.8
Madagascar	MDG	MgSO4 management of pre-eclampsia	22.4	18.2
Madagascar	MDG	Thermal protection	37.5	32.2
Madagascar	MDG	Clean cord care	36.2	31.0
Madagascar	MDG	Clean birth environment	31.1	26.7
Madagascar	MDG	Immediate drying and additional stimulation	34.7	29.8
Madagascar	MDG	Neonatal resuscitation	20.8	17.8
Madagascar	MDG	Antibiotics for preterm or prolonged PROM	28.4	24.3
Madagascar	MDG	Parenteral administration of anti-convulsants	27.1	23.2
Madagascar	MDG	Parenteral administration of uterotonics	33.9	29.1
Madagascar	MDG	Parenteral administration of antibiotics	28.4	24.3
Madagascar	MDG	Assisted vaginal delivery	9.6	8.2
Madagascar	MDG	Manual removal of placenta	14.2	12.2
Madagascar	MDG	Removal of retained products of conception	12.6	10.8
Madagascar	MDG	Cesarean delivery	3.3	2.8
Madagascar	MDG	Blood transfusion	4.8	4.1
Madagascar	MDG	Induction of labor for pregnancies lasting 41+ weeks	0.7	0.6
Madagascar	MDG	Complementary feeding - education only	24.1	20.7
Madagascar	MDG	Complementary feeding - supplementary feeding and education	24.1	20.7
Madagascar	MDG	Vitamin A supplementation	87.0	74.6
Madagascar	MDG	Improved sanitation - Utilization of latrines or toilets	10.5	10.5
Madagascar	MDG	Improved water source	54.4	54.4
Madagascar	MDG	Water connection in the home	19.3	19.3

Madagascar	MDG	Hand washing with soap	4.0	4.0
Madagascar	MDG	Hygienic disposal of children's stools	39.0	39.0
Madagascar	MDG	ITN/IRS - Households protected from malaria	79.5	68.2
Madagascar	MDG	Injectable antibiotics for neonatal sepsis	37.9	30.9
Madagascar	MDG	ORS - oral rehydration solution	44.5	38.2
Madagascar	MDG	Antibiotics for treatment of dysentery	34.1	29.2
Madagascar	MDG	Zinc for treatment of diarrhea	3.0	2.6
Madagascar	MDG	Oral antibiotics for pneumonia	42.0	36.0
Madagascar	MDG	Vitamin A for treatment of measles	87.0	74.6
Madagascar	MDG	ACTs- Artemisinin compounds for treatment of malaria	3.4	2.9
Madagascar	MDG	SAM - treatment for severe acute malnutrition	1.4	1.2
Madagascar	MDG	BCG vaccine	70.0	57.0
Madagascar	MDG	Polio vaccine	76.0	61.9
Madagascar	MDG	DPT vaccine	75.0	61.1
Madagascar	MDG	H. influenzae type b vaccine	75.0	70.5
Madagascar	MDG	HepB vaccine	75.0	61.1
Madagascar	MDG	Pneumococcal vaccine	75.0	70.5
Madagascar	MDG	Rotavirus vaccine	78.0	73.4
Madagascar	MDG	Measles vaccine	58.0	47.2
Madagascar	MDG	Global wasting (<-2 SD) rate	14.5	15.9
Madagascar	MDG	Contraceptive prevalence (CPR)	48.2	43.5
Malawi	MWI	Safe abortion services	3.3	3.0
Malawi	MWI	TT - Tetanus toxoid vaccination	89.0	72.5
Malawi	MWI	IPTp - Intermittent preventive treatment of malaria during pregnancy	76.1	62.0
Malawi	MWI	Syphilis detection and treatment	15.2	12.4
Malawi	MWI	Iron supplementation in pregnancy	33.4	27.2
Malawi	MWI	Hypertensive disorder case management	5.0	4.1
Malawi	MWI	Diabetes case management	6.4	5.2
Malawi	MWI	Malaria case management	41.0	33.4
Malawi	MWI	MgSO4 management of pre-eclampsia	40.6	33.1
Malawi	MWI	Thermal protection	90.5	77.6
Malawi	MWI	Clean cord care	84.1	72.1
Malawi	MWI	Clean birth environment	64.6	55.4
Malawi	MWI	Immediate drying and additional stimulation	83.0	71.2

Malawi	MWI	Neonatal resuscitation	83.7	71.8
Malawi	MWI	Antibiotics for preterm or prolonged PROM	80.3	68.8
Malawi	MWI	Parenteral administration of anti-convulsants	88.0	75.4
Malawi	MWI	Parenteral administration of uterotonics	89.5	76.7
Malawi	MWI	Parenteral administration of antibiotics	80.3	68.8
Malawi	MWI	Assisted vaginal delivery	46.1	39.5
Malawi	MWI	Manual removal of placenta	4.5	3.9
Malawi	MWI	Removal of retained products of conception	30.0	25.7
Malawi	MWI	Cesarean delivery	1.9	1.6
Malawi	MWI	Blood transfusion	12.2	10.5
Malawi	MWI	Induction of labor for pregnancies lasting 41+ weeks	6.1	5.2
Malawi	MWI	Complementary feeding - education only	25.0	21.4
Malawi	MWI	Complementary feeding - supplementary feeding and education	25.0	21.4
Malawi	MWI	Vitamin A supplementation	91.0	78.0
Malawi	MWI	Improved sanitation - Utilization of latrines or toilets	26.2	26.2
Malawi	MWI	Improved water source	68.8	68.8
Malawi	MWI	Water connection in the home	14.9	14.9
Malawi	MWI	Hand washing with soap	10.7	10.7
Malawi	MWI	Hygienic disposal of children's stools	85.5	85.5
Malawi	MWI	ITN/IRS - Households protected from malaria	82.1	70.4
Malawi	MWI	Injectable antibiotics for neonatal sepsis	90.7	73.9
Malawi	MWI	ORS - oral rehydration solution	64.7	55.5
Malawi	MWI	Antibiotics for treatment of dysentery	19.5	16.7
Malawi	MWI	Zinc for treatment of diarrhea	28.1	24.1
Malawi	MWI	Oral antibiotics for pneumonia	74.4	63.8
Malawi	MWI	Vitamin A for treatment of measles	91.0	78.0
Malawi	MWI	ACTs- Artemisinin compounds for treatment of malaria	27.8	23.8
Malawi	MWI	SAM - treatment for severe acute malnutrition	7.2	6.2
Malawi	MWI	BCG vaccine	92.0	74.9
Malawi	MWI	Polio vaccine	91.0	74.1
Malawi	MWI	DPT vaccine	92.0	74.9
Malawi	MWI	H. influenzae type b vaccine	92.0	86.5
Malawi	MWI	HepB vaccine	92.0	74.9

Malawi	MWI	Pneumococcal vaccine	92.0	86.5
Malawi	MWI	Rotavirus vaccine	90.0	84.7
Malawi	MWI	Measles vaccine	83.0	67.6
Malawi	MWI	Global wasting (<-2 SD) rate	2.8	3.1
Malawi	MWI	Contraceptive prevalence (CPR)	63	56.9
Maldives	MDV	Safe abortion services	35.1	31.7
Maldives	MDV	TT - Tetanus toxoid vaccination	99.0	80.6
Maldives	MDV	Syphilis detection and treatment	24.4	19.9
Maldives	MDV	Iron supplementation in pregnancy	64.6	52.6
Maldives	MDV	Hypertensive disorder case management	19.7	16.0
Maldives	MDV	Diabetes case management	15.3	12.5
Maldives	MDV	Malaria case management	63.5	51.7
Maldives	MDV	MgSO4 management of pre-eclampsia	38.9	31.7
Maldives	MDV	Thermal protection	98.3	84.3
Maldives	MDV	Clean cord care	94.9	81.4
Maldives	MDV	Clean birth environment	81.6	70.0
Maldives	MDV	Immediate drying and additional stimulation	91.1	78.1
Maldives	MDV	Neonatal resuscitation	54.7	46.9
Maldives	MDV	Antibiotics for preterm or prolonged PROM	74.4	63.8
Maldives	MDV	Parenteral administration of anti-convulsants	71.1	61.0
Maldives	MDV	Parenteral administration of uterotonics	88.9	76.2
Maldives	MDV	Parenteral administration of antibiotics	74.4	63.8
Maldives	MDV	Assisted vaginal delivery	25.1	21.5
Maldives	MDV	Manual removal of placenta	37.2	31.9
Maldives	MDV	Removal of retained products of conception	33.0	28.3
Maldives	MDV	Cesarean delivery	8.7	7.5
Maldives	MDV	Blood transfusion	12.5	10.7
Maldives	MDV	Induction of labor for pregnancies lasting 41+ weeks	1.8	1.5
Maldives	MDV	Complementary feeding - education only	75.8	65.0
Maldives	MDV	Complementary feeding - supplementary feeding and education	75.8	65.0
Maldives	MDV	Vitamin A supplementation	69.0	59.2
Maldives	MDV	Improved sanitation - Utilization of latrines or toilets	99.0	99.0
Maldives	MDV	Improved water source	99.0	99.0
Maldives	MDV	Water connection in the home	47.3	47.3

Maldives	MDV	Hand washing with soap	95.8	95.8
Maldives	MDV	Hygienic disposal of children's stools	9.1	9.1
Maldives	MDV	Injectable antibiotics for neonatal sepsis	99.5	81.0
Maldives	MDV	ORS - oral rehydration solution	74.8	64.1
Maldives	MDV	Zinc for treatment of diarrhea	48.3	41.4
Maldives	MDV	Oral antibiotics for pneumonia	74.1	63.5
Maldives	MDV	Vitamin A for treatment of measles	69.0	59.2
Maldives	MDV	BCG vaccine	99.0	80.6
Maldives	MDV	Polio vaccine	99.0	80.6
Maldives	MDV	DPT vaccine	99.0	80.6
Maldives	MDV	H. influenzae type b vaccine	99.0	93.1
Maldives	MDV	HepB vaccine	99.0	80.6
Maldives	MDV	Measles vaccine	99.0	80.6
Maldives	MDV	Global wasting (<-2 SD) rate	9.1	10.0
Maldives	MDV	Contraceptive prevalence (CPR)	34.55	31.2
Mali	MLI	TT - Tetanus toxoid vaccination	85.0	69.2
Mali	MLI	IPTp - Intermittent preventive treatment of malaria during pregnancy	55.4	45.1
Mali	MLI	Syphilis detection and treatment	19.4	15.8
Mali	MLI	Iron supplementation in pregnancy	18.3	14.9
Mali	MLI	Hypertensive disorder case management	10.4	8.5
Mali	MLI	Diabetes case management	8.1	6.6
Mali	MLI	Malaria case management	33.6	27.4
Mali	MLI	MgSO4 management of pre-eclampsia	20.6	16.8
Mali	MLI	Thermal protection	66.0	56.6
Mali	MLI	Clean cord care	63.7	54.6
Mali	MLI	Clean birth environment	54.8	47.0
Mali	MLI	Immediate drying and additional stimulation	61.2	52.5
Mali	MLI	Neonatal resuscitation	36.7	31.5
Mali	MLI	Antibiotics for preterm or prolonged PROM	50.0	42.9
Mali	MLI	Parenteral administration of anti-convulsants	47.8	41.0
Mali	MLI	Parenteral administration of uterotonics	59.7	51.2
Mali	MLI	Parenteral administration of antibiotics	50.0	42.9
Mali	MLI	Assisted vaginal delivery	16.9	14.5
Mali	MLI	Manual removal of placenta	25.0	21.4
Mali	MLI	Removal of retained products of conception	22.1	18.9

Mali	MLI	Cesarean delivery	5.8	5.0
Mali	MLI	Blood transfusion	8.4	7.2
Mali	MLI	Induction of labor for pregnancies lasting 41+ weeks	1.2	1.0
Mali	MLI	Complementary feeding - education only	21.8	18.7
Mali	MLI	Complementary feeding - supplementary feeding and education	21.8	18.7
Mali	MLI	Vitamin A supplementation	9.0	7.7
Mali	MLI	Improved sanitation - Utilization of latrines or toilets	39.3	39.3
Mali	MLI	Improved water source	78.3	78.3
Mali	MLI	Water connection in the home	35.6	35.6
Mali	MLI	Hand washing with soap	60.9	60.9
Mali	MLI	Hygienic disposal of children's stools	65.0	65.0
Mali	MLI	ITN/IRS - Households protected from malaria	89.8	77.0
Mali	MLI	Injectable antibiotics for neonatal sepsis	66.8	54.4
Mali	MLI	ORS - oral rehydration solution	21.4	18.3
Mali	MLI	Antibiotics for treatment of dysentery	18.5	15.9
Mali	MLI	Zinc for treatment of diarrhea	15.4	13.2
Mali	MLI	Oral antibiotics for pneumonia	70.9	60.8
Mali	MLI	Vitamin A for treatment of measles	9.0	7.7
Mali	MLI	ACTs- Artemisinin compounds for treatment of malaria	0.9	0.8
Mali	MLI	SAM - treatment for severe acute malnutrition	41.8	35.8
Mali	MLI	BCG vaccine	83.0	67.6
Mali	MLI	Polio vaccine	73.0	59.5
Mali	MLI	DPT vaccine	71.0	57.8
Mali	MLI	H. influenzae type b vaccine	71.0	66.8
Mali	MLI	HepB vaccine	71.0	57.8
Mali	MLI	Pneumococcal vaccine	68.0	64.0
Mali	MLI	Rotavirus vaccine	55.0	51.7
Mali	MLI	Meningococcal A	70.0	57.0
Mali	MLI	Measles vaccine	61.0	49.7
Mali	MLI	Global wasting (<-2 SD) rate	13.4	14.7
Mali	MLI	Contraceptive prevalence (CPR)	17.05	15.4
Mauritania	MRT	TT - Tetanus toxoid vaccination	80.0	65.2

Mauritania	MRT	IPTp - Intermittent preventive treatment of malaria during pregnancy	25.2	20.5
Mauritania	MRT	Syphilis detection and treatment	21.5	17.5
Mauritania	MRT	Iron supplementation in pregnancy	6.1	5.0
Mauritania	MRT	Hypertensive disorder case management	15.1	12.3
Mauritania	MRT	Diabetes case management	11.8	9.6
Mauritania	MRT	Malaria case management	48.8	39.7
Mauritania	MRT	MgSO4 management of pre-eclampsia	29.9	24.4
Mauritania	MRT	Thermal protection	68.5	58.7
Mauritania	MRT	Clean cord care	55.2	47.3
Mauritania	MRT	Clean birth environment	44.0	37.7
Mauritania	MRT	Immediate drying and additional stimulation	63.7	54.6
Mauritania	MRT	Neonatal resuscitation	32.4	27.8
Mauritania	MRT	Antibiotics for preterm or prolonged PROM	47.5	40.7
Mauritania	MRT	Parenteral administration of anti-convulsants	25.5	21.9
Mauritania	MRT	Parenteral administration of uterotonics	54.8	47.0
Mauritania	MRT	Parenteral administration of antibiotics	47.5	40.7
Mauritania	MRT	Assisted vaginal delivery	23.3	20.0
Mauritania	MRT	Manual removal of placenta	46.2	39.6
Mauritania	MRT	Removal of retained products of conception	28.1	24.1
Mauritania	MRT	Cesarean delivery	8.4	7.2
Mauritania	MRT	Blood transfusion	7.6	6.5
Mauritania	MRT	Induction of labor for pregnancies lasting 41+ weeks	6.7	5.7
Mauritania	MRT	Complementary feeding - education only	34.3	29.4
Mauritania	MRT	Complementary feeding - supplementary feeding and education	34.3	29.4
Mauritania	MRT	Improved sanitation - Utilization of latrines or toilets	48.4	48.4
Mauritania	MRT	Improved water source	70.7	70.7
Mauritania	MRT	Water connection in the home	37.0	37.0
Mauritania	MRT	Hand washing with soap	41.0	41.0
Mauritania	MRT	Hygienic disposal of children's stools	20.2	20.2
Mauritania	MRT	ITN/IRS - Households protected from malaria	48.9	41.9
Mauritania	MRT	Injectable antibiotics for neonatal sepsis	69.3	56.4
Mauritania	MRT	ORS - oral rehydration solution	25.4	21.8
Mauritania	MRT	Zinc for treatment of diarrhea	23.2	19.9

Mauritania	MRT	Oral antibiotics for pneumonia	33.7	28.9
Mauritania	MRT	ACTs- Artemisinin compounds for treatment of malaria	0.7	0.6
Mauritania	MRT	SAM - treatment for severe acute malnutrition	22.4	19.2
Mauritania	MRT	BCG vaccine	90.0	73.3
Mauritania	MRT	Polio vaccine	81.0	66.0
Mauritania	MRT	DPT vaccine	81.0	66.0
Mauritania	MRT	H. influenzae type b vaccine	81.0	76.2
Mauritania	MRT	HepB vaccine	81.0	66.0
Mauritania	MRT	Pneumococcal vaccine	77.0	72.4
Mauritania	MRT	Rotavirus vaccine	76.0	71.5
Mauritania	MRT	Measles vaccine	78.0	63.5
Mauritania	MRT	Global wasting (<-2 SD) rate	14.9	16.4
Mauritania	MRT	Contraceptive prevalence (CPR)	18.1	16.3
Mexico	MEX	Safe abortion services	0.6	0.5
Mexico	MEX	TT - Tetanus toxoid vaccination	96.0	78.2
Mexico	MEX	Syphilis detection and treatment	24.3	19.8
Mexico	MEX	Hypertensive disorder case management	22.6	18.4
Mexico	MEX	Diabetes case management	17.7	14.4
Mexico	MEX	Malaria case management	73.1	59.5
Mexico	MEX	MgSO4 management of pre-eclampsia	44.8	36.5
Mexico	MEX	Thermal protection	95.8	82.1
Mexico	MEX	Clean cord care	92.5	79.3
Mexico	MEX	Clean birth environment	79.5	68.2
Mexico	MEX	Immediate drying and additional stimulation	88.8	76.1
Mexico	MEX	Neonatal resuscitation	53.3	45.7
Mexico	MEX	Antibiotics for preterm or prolonged PROM	72.5	62.2
Mexico	MEX	Parenteral administration of anti-convulsants	69.3	59.4
Mexico	MEX	Parenteral administration of uterotonics	86.6	74.2
Mexico	MEX	Parenteral administration of antibiotics	72.5	62.2
Mexico	MEX	Assisted vaginal delivery	24.5	21.0
Mexico	MEX	Manual removal of placenta	36.3	31.1
Mexico	MEX	Removal of retained products of conception	32.1	27.5
Mexico	MEX	Cesarean delivery	8.4	7.2
Mexico	MEX	Blood transfusion	12.2	10.5

Mexico	MEX	Induction of labor for pregnancies lasting 41+ weeks	1.7	1.5
Mexico	MEX	Complementary feeding - education only	72.1	61.8
Mexico	MEX	Complementary feeding - supplementary feeding and education	72.1	61.8
Mexico	MEX	Improved sanitation - Utilization of latrines or toilets	91.2	91.2
Mexico	MEX	Improved water source	99.0	99.0
Mexico	MEX	Water connection in the home	95.0	95.0
Mexico	MEX	Hand washing with soap	90.3	90.3
Mexico	MEX	Hygienic disposal of children's stools	14.0	14.0
Mexico	MEX	Injectable antibiotics for neonatal sepsis	96.9	78.9
Mexico	MEX	ORS - oral rehydration solution	61.4	52.6
Mexico	MEX	Zinc for treatment of diarrhea	10.8	9.3
Mexico	MEX	Oral antibiotics for pneumonia	73.1	62.7
Mexico	MEX	BCG vaccine	96.0	78.2
Mexico	MEX	Polio vaccine	88.0	71.7
Mexico	MEX	DPT vaccine	88.0	71.7
Mexico	MEX	H. influenzae type b vaccine	88.0	82.8
Mexico	MEX	HepB vaccine	55.0	44.8
Mexico	MEX	Pneumococcal vaccine	88.0	82.8
Mexico	MEX	Rotavirus vaccine	77.0	72.4
Mexico	MEX	Measles vaccine	96.0	78.2
Mexico	MEX	Global wasting (<-2 SD) rate	1.1	1.2
Mexico	MEX	Contraceptive prevalence (CPR)	69.75	62.9
Mongolia	MNG	Safe abortion services	100.0	90.3
Mongolia	MNG	Syphilis detection and treatment	24.5	20.0
Mongolia	MNG	Iron supplementation in pregnancy	25.9	21.1
Mongolia	MNG	Hypertensive disorder case management	21.3	17.3
Mongolia	MNG	Diabetes case management	16.6	13.5
Mongolia	MNG	Malaria case management	68.6	55.9
Mongolia	MNG	MgSO4 management of pre-eclampsia	42.1	34.3
Mongolia	MNG	Thermal protection	97.3	83.4
Mongolia	MNG	Clean cord care	93.9	80.5
Mongolia	MNG	Clean birth environment	80.7	69.2
Mongolia	MNG	Immediate drying and additional stimulation	90.1	77.2
Mongolia	MNG	Neonatal resuscitation	54.1	46.4

Mongolia	MNG	Antibiotics for preterm or prolonged PROM	73.6	63.1
Mongolia	MNG	Parenteral administration of anti-convulsants	70.4	60.4
Mongolia	MNG	Parenteral administration of uterotonics	87.9	75.4
Mongolia	MNG	Parenteral administration of antibiotics	73.6	63.1
Mongolia	MNG	Assisted vaginal delivery	24.9	21.3
Mongolia	MNG	Manual removal of placenta	36.8	31.6
Mongolia	MNG	Removal of retained products of conception	32.6	28.0
Mongolia	MNG	Cesarean delivery	8.6	7.4
Mongolia	MNG	Blood transfusion	12.4	10.6
Mongolia	MNG	Induction of labor for pregnancies lasting 41+ weeks	1.7	1.5
Mongolia	MNG	Complementary feeding - education only	50.8	43.6
Mongolia	MNG	Complementary feeding - supplementary feeding and education	50.8	43.6
Mongolia	MNG	Vitamin A supplementation	83.0	71.2
Mongolia	MNG	Improved sanitation - Utilization of latrines or toilets	58.5	58.5
Mongolia	MNG	Improved water source	83.3	83.3
Mongolia	MNG	Water connection in the home	20.9	20.9
Mongolia	MNG	Hand washing with soap	78.9	78.9
Mongolia	MNG	Hygienic disposal of children's stools	51.3	51.3
Mongolia	MNG	Injectable antibiotics for neonatal sepsis	98.4	80.1
Mongolia	MNG	ORS - oral rehydration solution	58.2	49.9
Mongolia	MNG	Antibiotics for treatment of dysentery	20.1	17.2
Mongolia	MNG	Zinc for treatment of diarrhea	16.5	14.1
Mongolia	MNG	Oral antibiotics for pneumonia	70.3	60.3
Mongolia	MNG	Vitamin A for treatment of measles	83.0	71.2
Mongolia	MNG	BCG vaccine	99.0	80.6
Mongolia	MNG	Polio vaccine	99.0	80.6
Mongolia	MNG	DPT vaccine	99.0	80.6
Mongolia	MNG	H. influenzae type b vaccine	99.0	93.1
Mongolia	MNG	HepB vaccine	99.0	80.6
Mongolia	MNG	Pneumococcal vaccine	26.0	24.5
Mongolia	MNG	Measles vaccine	99.0	80.6
Mongolia	MNG	Global wasting (<-2 SD) rate	1.0	1.1
Mongolia	MNG	Contraceptive prevalence (CPR)	55.4	50.0
Montenegro	MNE	Safe abortion services	100.0	90.3

Montenegro	MNE	Syphilis detection and treatment	24.0	19.5
Montenegro	MNE	Hypertensive disorder case management	22.6	18.4
Montenegro	MNE	Diabetes case management	17.6	14.3
Montenegro	MNE	Malaria case management	73.0	59.5
Montenegro	MNE	MgSO4 management of pre-eclampsia	44.8	36.5
Montenegro	MNE	Thermal protection	97.5	83.6
Montenegro	MNE	Clean cord care	94.1	80.7
Montenegro	MNE	Clean birth environment	80.9	69.4
Montenegro	MNE	Immediate drying and additional stimulation	90.3	77.4
Montenegro	MNE	Neonatal resuscitation	54.2	46.5
Montenegro	MNE	Antibiotics for preterm or prolonged PROM	73.8	63.3
Montenegro	MNE	Parenteral administration of anti-convulsants	70.5	60.4
Montenegro	MNE	Parenteral administration of uterotonics	88.1	75.5
Montenegro	MNE	Parenteral administration of antibiotics	73.8	63.3
Montenegro	MNE	Assisted vaginal delivery	24.9	21.3
Montenegro	MNE	Manual removal of placenta	36.9	31.6
Montenegro	MNE	Removal of retained products of conception	32.7	28.0
Montenegro	MNE	Cesarean delivery	8.6	7.4
Montenegro	MNE	Blood transfusion	12.4	10.6
Montenegro	MNE	Induction of labor for pregnancies lasting 41+ weeks	1.7	1.5
Montenegro	MNE	Complementary feeding - education only	81.3	69.7
Montenegro	MNE	Complementary feeding - supplementary feeding and education	81.3	69.7
Montenegro	MNE	Improved sanitation - Utilization of latrines or toilets	97.8	97.8
Montenegro	MNE	Improved water source	97.0	97.0
Montenegro	MNE	Water connection in the home	82.4	82.4
Montenegro	MNE	Hand washing with soap	99.6	99.6
Montenegro	MNE	Hygienic disposal of children's stools	21.3	21.3
Montenegro	MNE	Injectable antibiotics for neonatal sepsis	98.6	80.3
Montenegro	MNE	ORS - oral rehydration solution	31.9	27.4
Montenegro	MNE	Oral antibiotics for pneumonia	89.4	76.6
Montenegro	MNE	BCG vaccine	83.0	67.6
Montenegro	MNE	Polio vaccine	87.0	70.9
Montenegro	MNE	DPT vaccine	87.0	70.9
Montenegro	MNE	H. influenzae type b vaccine	87.0	81.8

Montenegro	MNE	HepB vaccine	73.0	59.5
Montenegro	MNE	Measles vaccine	58.0	47.2
Montenegro	MNE	Global wasting (<-2 SD) rate	2.9	3.2
Montenegro	MNE	Contraceptive prevalence (CPR)	27.3	24.6
Morocco	MAR	Safe abortion services	2.2	2.0
Morocco	MAR	TT - Tetanus toxoid vaccination	88.0	71.7
Morocco	MAR	Syphilis detection and treatment	16.7	13.6
Morocco	MAR	Iron supplementation in pregnancy	5.2	4.2
Morocco	MAR	Hypertensive disorder case management	7.2	5.9
Morocco	MAR	Diabetes case management	5.6	4.6
Morocco	MAR	Malaria case management	23.3	19.0
Morocco	MAR	MgSO4 management of pre-eclampsia	14.3	11.6
Morocco	MAR	Thermal protection	71.9	61.6
Morocco	MAR	Clean cord care	69.4	59.5
Morocco	MAR	Clean birth environment	59.6	51.1
Morocco	MAR	Immediate drying and additional stimulation	66.6	57.1
Morocco	MAR	Neonatal resuscitation	40.0	34.3
Morocco	MAR	Antibiotics for preterm or prolonged PROM	54.4	46.6
Morocco	MAR	Parenteral administration of anti-convulsants	52.0	44.6
Morocco	MAR	Parenteral administration of uterotonics	65.0	55.7
Morocco	MAR	Parenteral administration of antibiotics	54.4	46.6
Morocco	MAR	Assisted vaginal delivery	18.4	15.8
Morocco	MAR	Manual removal of placenta	27.2	23.3
Morocco	MAR	Removal of retained products of conception	24.1	20.7
Morocco	MAR	Cesarean delivery	6.3	5.4
Morocco	MAR	Blood transfusion	9.2	7.9
Morocco	MAR	Induction of labor for pregnancies lasting 41+ weeks	1.3	1.1
Morocco	MAR	Complementary feeding - education only	22.3	19.1
Morocco	MAR	Complementary feeding - supplementary feeding and education	22.3	19.1
Morocco	MAR	Vitamin A supplementation	99.0	84.9
Morocco	MAR	Improved sanitation - Utilization of latrines or toilets	88.5	88.5
Morocco	MAR	Improved water source	86.8	86.8
Morocco	MAR	Water connection in the home	67.7	67.7
Morocco	MAR	Hygienic disposal of children's stools	6.3	6.3

Morocco	MAR	Injectable antibiotics for neonatal sepsis	72.7	59.2
Morocco	MAR	ORS - oral rehydration solution	22.9	19.6
Morocco	MAR	Vitamin A for treatment of measles	99.0	84.9
Morocco	MAR	BCG vaccine	99.0	80.6
Morocco	MAR	Polio vaccine	99.0	80.6
Morocco	MAR	DPT vaccine	99.0	80.6
Morocco	MAR	H. influenzae type b vaccine	99.0	93.1
Morocco	MAR	HepB vaccine	99.0	80.6
Morocco	MAR	Pneumococcal vaccine	99.0	93.1
Morocco	MAR	Rotavirus vaccine	99.0	93.1
Morocco	MAR	Measles vaccine	99.0	80.6
Morocco	MAR	Global wasting (<-2 SD) rate	10.1	11.1
Morocco	MAR	Contraceptive prevalence (CPR)	69.85	63.0
Mozambique	MOZ	Safe abortion services	3.3	3.0
Mozambique	MOZ	TT - Tetanus toxoid vaccination	86.0	70.0
Mozambique	MOZ	IPTp - Intermittent preventive treatment of malaria during pregnancy	35.8	29.2
Mozambique	MOZ	Syphilis detection and treatment	22.4	18.2
Mozambique	MOZ	Iron supplementation in pregnancy	25.9	21.1
Mozambique	MOZ	Hypertensive disorder case management	12.9	10.5
Mozambique	MOZ	Diabetes case management	10.1	8.2
Mozambique	MOZ	Malaria case management	41.7	34.0
Mozambique	MOZ	MgSO4 management of pre-eclampsia	25.6	20.9
Mozambique	MOZ	Thermal protection	64.1	55.0
Mozambique	MOZ	Clean cord care	61.9	53.1
Mozambique	MOZ	Clean birth environment	53.2	45.6
Mozambique	MOZ	Immediate drying and additional stimulation	59.4	50.9
Mozambique	MOZ	Neonatal resuscitation	35.7	30.6
Mozambique	MOZ	Antibiotics for preterm or prolonged PROM	48.5	41.6
Mozambique	MOZ	Parenteral administration of anti-convulsants	46.4	39.8
Mozambique	MOZ	Parenteral administration of uterotonics	57.9	49.6
Mozambique	MOZ	Parenteral administration of antibiotics	48.5	41.6
Mozambique	MOZ	Assisted vaginal delivery	16.4	14.1
Mozambique	MOZ	Manual removal of placenta	24.3	20.8
Mozambique	MOZ	Removal of retained products of conception	21.5	18.4
Mozambique	MOZ	Cesarean delivery	5.6	4.8

Mozambique	MOZ	Blood transfusion	8.2	7.0
Mozambique	MOZ	Induction of labor for pregnancies lasting 41+ weeks	1.1	0.9
Mozambique	MOZ	Complementary feeding - education only	30.4	26.1
Mozambique	MOZ	Complementary feeding - supplementary feeding and education	30.4	26.1
Mozambique	MOZ	Vitamin A supplementation	61.0	52.3
Mozambique	MOZ	Improved sanitation - Utilization of latrines or toilets	29.4	29.4
Mozambique	MOZ	Improved water source	55.7	55.7
Mozambique	MOZ	Water connection in the home	19.8	19.8
Mozambique	MOZ	Hand washing with soap	31.7	31.7
Mozambique	MOZ	Hygienic disposal of children's stools	45.0	45.0
Mozambique	MOZ	ITN/IRS - Households protected from malaria	68.7	58.9
Mozambique	MOZ	Injectable antibiotics for neonatal sepsis	64.8	52.8
Mozambique	MOZ	ORS - oral rehydration solution	45.9	39.4
Mozambique	MOZ	Antibiotics for treatment of dysentery	31.6	27.1
Mozambique	MOZ	Zinc for treatment of diarrhea	30.9	26.5
Mozambique	MOZ	Oral antibiotics for pneumonia	59.4	50.9
Mozambique	MOZ	Vitamin A for treatment of measles	61.0	52.3
Mozambique	MOZ	ACTs- Artemisinin compounds for treatment of malaria	15.4	13.2
Mozambique	MOZ	BCG vaccine	95.0	77.4
Mozambique	MOZ	Polio vaccine	80.0	65.2
Mozambique	MOZ	DPT vaccine	80.0	65.2
Mozambique	MOZ	H. influenzae type b vaccine	80.0	75.3
Mozambique	MOZ	HepB vaccine	80.0	65.2
Mozambique	MOZ	Pneumococcal vaccine	80.0	75.3
Mozambique	MOZ	Rotavirus vaccine	80.0	75.3
Mozambique	MOZ	Measles vaccine	85.0	69.2
Mozambique	MOZ	Global wasting (<-2 SD) rate	6.0	6.6
Mozambique	MOZ	Contraceptive prevalence (CPR)	29.95	27.0
Myanmar	MMR	Safe abortion services	39.0	35.2
Myanmar	MMR	TT - Tetanus toxoid vaccination	90.0	73.3
Myanmar	MMR	Syphilis detection and treatment	20.5	16.7
Myanmar	MMR	Iron supplementation in pregnancy	59.3	48.3
Myanmar	MMR	Hypertensive disorder case management	14.3	11.6

Myanmar	MMR	Diabetes case management	11.2	9.1
Myanmar	MMR	Malaria case management	46.1	37.5
Myanmar	MMR	MgSO4 management of pre-eclampsia	28.3	23.1
Myanmar	MMR	Thermal protection	36.6	31.4
Myanmar	MMR	Clean cord care	35.4	30.4
Myanmar	MMR	Clean birth environment	30.4	26.1
Myanmar	MMR	Immediate drying and additional stimulation	33.9	29.1
Myanmar	MMR	Neonatal resuscitation	20.4	17.5
Myanmar	MMR	Antibiotics for preterm or prolonged PROM	27.7	23.7
Myanmar	MMR	Parenteral administration of anti-convulsants	26.5	22.7
Myanmar	MMR	Parenteral administration of uterotonics	33.1	28.4
Myanmar	MMR	Parenteral administration of antibiotics	27.7	23.7
Myanmar	MMR	Assisted vaginal delivery	9.4	8.1
Myanmar	MMR	Manual removal of placenta	13.9	11.9
Myanmar	MMR	Removal of retained products of conception	12.3	10.5
Myanmar	MMR	Cesarean delivery	3.2	2.7
Myanmar	MMR	Blood transfusion	4.7	4.0
Myanmar	MMR	Induction of labor for pregnancies lasting 41+ weeks	0.7	0.6
Myanmar	MMR	Complementary feeding - education only	24.8	21.3
Myanmar	MMR	Complementary feeding - supplementary feeding and education	24.8	21.3
Myanmar	MMR	Vitamin A supplementation	89.0	76.3
Myanmar	MMR	Improved sanitation - Utilization of latrines or toilets	64.3	64.3
Myanmar	MMR	Improved water source	81.8	81.8
Myanmar	MMR	Water connection in the home	20.1	20.1
Myanmar	MMR	Hand washing with soap	79.9	79.9
Myanmar	MMR	Hygienic disposal of children's stools	42.1	42.1
Myanmar	MMR	ITN/IRS - Households protected from malaria	26.8	23.0
Myanmar	MMR	Injectable antibiotics for neonatal sepsis	37.1	30.2
Myanmar	MMR	ORS - oral rehydration solution	61.9	53.1
Myanmar	MMR	Zinc for treatment of diarrhea	8.4	7.2
Myanmar	MMR	Oral antibiotics for pneumonia	58.6	50.2
Myanmar	MMR	Vitamin A for treatment of measles	89.0	76.3
Myanmar	MMR	ACTs- Artemisinin compounds for treatment of malaria	0.2	0.2

Myanmar	MMR	SAM - treatment for severe acute malnutrition	3.5	3.0
Myanmar	MMR	BCG vaccine	90.0	73.3
Myanmar	MMR	Polio vaccine	91.0	74.1
Myanmar	MMR	DPT vaccine	91.0	74.1
Myanmar	MMR	H. influenzae type b vaccine	91.0	85.6
Myanmar	MMR	HepB vaccine	91.0	74.1
Myanmar	MMR	Pneumococcal vaccine	91.0	85.6
Myanmar	MMR	Measles vaccine	83.0	67.6
Myanmar	MMR	Global wasting (<-2 SD) rate	6.8	7.4
Myanmar	MMR	Contraceptive prevalence (CPR)	56.65	51.1
Namibia	NAM	Safe abortion services	42.5	38.4
Namibia	NAM	TT - Tetanus toxoid vaccination	88.0	71.7
Namibia	NAM	IPTp - Intermittent preventive treatment of malaria during pregnancy	5.1	4.2
Namibia	NAM	Syphilis detection and treatment	92.6	75.4
Namibia	NAM	Iron supplementation in pregnancy	38.6	31.4
Namibia	NAM	Hypertensive disorder case management	42.1	34.3
Namibia	NAM	Diabetes case management	32.2	26.2
Namibia	NAM	Malaria case management	45.5	37.1
Namibia	NAM	MgSO4 management of pre-eclampsia	31.8	25.9
Namibia	NAM	Thermal protection	86.7	74.3
Namibia	NAM	Clean cord care	87.2	74.8
Namibia	NAM	Clean birth environment	69.1	59.2
Namibia	NAM	Immediate drying and additional stimulation	86.5	74.2
Namibia	NAM	Neonatal resuscitation	78.2	67.0
Namibia	NAM	Antibiotics for preterm or prolonged PROM	81.9	70.2
Namibia	NAM	Parenteral administration of anti-convulsants	86.0	73.7
Namibia	NAM	Parenteral administration of uterotonics	76.9	65.9
Namibia	NAM	Parenteral administration of antibiotics	81.9	70.2
Namibia	NAM	Assisted vaginal delivery	39.4	33.8
Namibia	NAM	Manual removal of placenta	65.3	56.0
Namibia	NAM	Removal of retained products of conception	23.0	19.7
Namibia	NAM	Cesarean delivery	41.8	35.8
Namibia	NAM	Blood transfusion	18.9	16.2
Namibia	NAM	Induction of labor for pregnancies lasting 41+ weeks	1.5	1.3

Namibia	NAM	Complementary feeding - education only	31.1	26.7
Namibia	NAM	Complementary feeding - supplementary feeding and education	31.1	26.7
Namibia	NAM	Vitamin A supplementation	27.0	23.1
Namibia	NAM	Improved sanitation - Utilization of latrines or toilets	34.5	34.5
Namibia	NAM	Improved water source	82.5	82.5
Namibia	NAM	Water connection in the home	65.4	65.4
Namibia	NAM	Hand washing with soap	54.1	54.1
Namibia	NAM	Hygienic disposal of children's stools	12.6	12.6
Namibia	NAM	ITN/IRS - Households protected from malaria	32.7	28.0
Namibia	NAM	Injectable antibiotics for neonatal sepsis	87.7	71.4
Namibia	NAM	ORS - oral rehydration solution	71.6	61.4
Namibia	NAM	Antibiotics for treatment of dysentery	13.4	11.5
Namibia	NAM	Zinc for treatment of diarrhea	0.2	0.2
Namibia	NAM	Oral antibiotics for pneumonia	67.7	58.0
Namibia	NAM	Vitamin A for treatment of measles	27.0	23.1
Namibia	NAM	ACTs- Artemisinin compounds for treatment of malaria	3.2	2.7
Namibia	NAM	BCG vaccine	94.0	76.6
Namibia	NAM	Polio vaccine	84.0	68.4
Namibia	NAM	DPT vaccine	89.0	72.5
Namibia	NAM	H. influenzae type b vaccine	89.0	83.7
Namibia	NAM	HepB vaccine	89.0	72.5
Namibia	NAM	Pneumococcal vaccine	61.0	57.4
Namibia	NAM	Rotavirus vaccine	92.0	86.5
Namibia	NAM	Measles vaccine	80.0	65.2
Namibia	NAM	Global wasting (<-2 SD) rate	7.1	7.9
Namibia	NAM	Contraceptive prevalence (CPR)	60.3	54.4
Nepal	NPL	Safe abortion services	35.1	31.7
Nepal	NPL	TT - Tetanus toxoid vaccination	89.0	72.5
Nepal	NPL	Syphilis detection and treatment	8.2	6.7
Nepal	NPL	Iron supplementation in pregnancy	70.9	57.7
Nepal	NPL	Hypertensive disorder case management	15.2	12.4
Nepal	NPL	Diabetes case management	8.5	6.9
Nepal	NPL	Malaria case management	21.8	17.8
Nepal	NPL	MgSO4 management of pre-eclampsia	41.6	33.9

Nepal	NPL	Thermal protection	56.7	48.6
Nepal	NPL	Clean cord care	56.6	48.5
Nepal	NPL	Clean birth environment	54.1	46.4
Nepal	NPL	Immediate drying and additional stimulation	55.5	47.6
Nepal	NPL	Neonatal resuscitation	53.3	45.7
Nepal	NPL	Antibiotics for preterm or prolonged PROM	44.3	38.0
Nepal	NPL	Parenteral administration of anti-convulsants	52.8	45.3
Nepal	NPL	Parenteral administration of uterotonics	55.8	47.8
Nepal	NPL	Parenteral administration of antibiotics	44.3	38.0
Nepal	NPL	Assisted vaginal delivery	39.9	34.2
Nepal	NPL	Manual removal of placenta	15.3	13.1
Nepal	NPL	Removal of retained products of conception	37.5	32.2
Nepal	NPL	Cesarean delivery	7.8	6.7
Nepal	NPL	Blood transfusion	13.8	11.8
Nepal	NPL	Induction of labor for pregnancies lasting 41+ weeks	18.9	16.2
Nepal	NPL	Complementary feeding - education only	46.5	39.9
Nepal	NPL	Complementary feeding - supplementary feeding and education	46.5	39.9
Nepal	NPL	Vitamin A supplementation	81.0	69.4
Nepal	NPL	Improved sanitation - Utilization of latrines or toilets	62.1	62.1
Nepal	NPL	Improved water source	88.8	88.8
Nepal	NPL	Water connection in the home	42.1	42.1
Nepal	NPL	Hand washing with soap	72.5	72.5
Nepal	NPL	Hygienic disposal of children's stools	44.4	44.4
Nepal	NPL	Injectable antibiotics for neonatal sepsis	57.4	46.8
Nepal	NPL	ORS - oral rehydration solution	37.0	31.7
Nepal	NPL	Antibiotics for treatment of dysentery	21.3	18.3
Nepal	NPL	Zinc for treatment of diarrhea	17.6	15.1
Nepal	NPL	Oral antibiotics for pneumonia	55.1	47.2
Nepal	NPL	Vitamin A for treatment of measles	81.0	69.4
Nepal	NPL	ACTs- Artemisinin compounds for treatment of malaria	0.2	0.2
Nepal	NPL	SAM - treatment for severe acute malnutrition	0.9	0.8
Nepal	NPL	BCG vaccine	96.0	78.2
Nepal	NPL	Polio vaccine	91.0	74.1

Nepal	NPL	DPT vaccine	91.0	74.1
Nepal	NPL	H. influenzae type b vaccine	91.0	85.6
Nepal	NPL	HepB vaccine	91.0	74.1
Nepal	NPL	Pneumococcal vaccine	82.0	77.1
Nepal	NPL	Measles vaccine	90.0	73.3
Nepal	NPL	Global wasting (<-2 SD) rate	9.8	10.8
Nepal	NPL	Contraceptive prevalence (CPR)	55.1	49.7
Nicaragua	NIC	Safe abortion services	0.6	0.5
Nicaragua	NIC	TT - Tetanus toxoid vaccination	90.0	73.3
Nicaragua	NIC	Syphilis detection and treatment	21.7	17.7
Nicaragua	NIC	Iron supplementation in pregnancy	61.6	50.2
Nicaragua	NIC	Hypertensive disorder case management	21.1	17.2
Nicaragua	NIC	Diabetes case management	16.4	13.4
Nicaragua	NIC	Malaria case management	68.1	55.5
Nicaragua	NIC	MgSO4 management of pre-eclampsia	41.7	34.0
Nicaragua	NIC	Thermal protection	74.4	63.8
Nicaragua	NIC	Clean cord care	71.8	61.6
Nicaragua	NIC	Clean birth environment	61.7	52.9
Nicaragua	NIC	Immediate drying and additional stimulation	68.9	59.1
Nicaragua	NIC	Neonatal resuscitation	41.4	35.5
Nicaragua	NIC	Antibiotics for preterm or prolonged PROM	56.3	48.3
Nicaragua	NIC	Parenteral administration of anti-convulsants	53.8	46.1
Nicaragua	NIC	Parenteral administration of uterotonics	67.2	57.6
Nicaragua	NIC	Parenteral administration of antibiotics	56.3	48.3
Nicaragua	NIC	Assisted vaginal delivery	19.0	16.3
Nicaragua	NIC	Manual removal of placenta	28.1	24.1
Nicaragua	NIC	Removal of retained products of conception	24.9	21.3
Nicaragua	NIC	Cesarean delivery	6.5	5.6
Nicaragua	NIC	Blood transfusion	9.5	8.1
Nicaragua	NIC	Induction of labor for pregnancies lasting 41+ weeks	1.3	1.1
Nicaragua	NIC	Vitamin A supplementation	3.0	2.6
Nicaragua	NIC	Improved sanitation - Utilization of latrines or toilets	74.4	74.4
Nicaragua	NIC	Improved water source	81.5	81.5
Nicaragua	NIC	Water connection in the home	56.5	56.5

Nicaragua	NIC	Hygienic disposal of children's stools	43.2	43.2
Nicaragua	NIC	Injectable antibiotics for neonatal sepsis	75.2	61.3
Nicaragua	NIC	ORS - oral rehydration solution	65.4	56.1
Nicaragua	NIC	Antibiotics for treatment of dysentery	10.6	9.1
Nicaragua	NIC	Vitamin A for treatment of measles	3.0	2.6
Nicaragua	NIC	BCG vaccine	98.0	79.8
Nicaragua	NIC	Polio vaccine	99.0	80.6
Nicaragua	NIC	DPT vaccine	98.0	79.8
Nicaragua	NIC	H. influenzae type b vaccine	98.0	92.2
Nicaragua	NIC	HepB vaccine	98.0	79.8
Nicaragua	NIC	Pneumococcal vaccine	98.0	92.2
Nicaragua	NIC	Rotavirus vaccine	98.0	92.2
Nicaragua	NIC	Measles vaccine	99.0	80.6
Nicaragua	NIC	Global wasting (<-2 SD) rate	1.4	1.6
Nicaragua	NIC	Contraceptive prevalence (CPR)	81.7	73.7
Niger	NER	TT - Tetanus toxoid vaccination	81.0	66.0
Niger	NER	IPTp - Intermittent preventive treatment of malaria during pregnancy	36.9	30.1
Niger	NER	Syphilis detection and treatment	20.8	16.9
Niger	NER	Iron supplementation in pregnancy	28.6	23.3
Niger	NER	Hypertensive disorder case management	8.0	6.5
Niger	NER	Diabetes case management	6.2	5.0
Niger	NER	Malaria case management	25.8	21.0
Niger	NER	MgSO4 management of pre-eclampsia	15.8	12.9
Niger	NER	Thermal protection	29.4	25.2
Niger	NER	Clean cord care	28.4	24.3
Niger	NER	Clean birth environment	24.4	20.9
Niger	NER	Immediate drying and additional stimulation	27.2	23.3
Niger	NER	Neonatal resuscitation	16.4	14.1
Niger	NER	Antibiotics for preterm or prolonged PROM	22.3	19.1
Niger	NER	Parenteral administration of anti-convulsants	21.3	18.3
Niger	NER	Parenteral administration of uterotonics	26.6	22.8
Niger	NER	Parenteral administration of antibiotics	22.3	19.1
Niger	NER	Assisted vaginal delivery	7.5	6.4
Niger	NER	Manual removal of placenta	11.1	9.5
Niger	NER	Removal of retained products of conception	9.9	8.5

Niger	NER	Cesarean delivery	2.6	2.2
Niger	NER	Blood transfusion	3.7	3.2
Niger	NER	Induction of labor for pregnancies lasting 41+ weeks	0.5	0.4
Niger	NER	Complementary feeding - education only	9.9	8.5
Niger	NER	Complementary feeding - supplementary feeding and education	9.9	8.5
Niger	NER	Vitamin A supplementation	53.0	45.4
Niger	NER	Improved sanitation - Utilization of latrines or toilets	13.6	13.6
Niger	NER	Improved water source	50.3	50.3
Niger	NER	Water connection in the home	16.3	16.3
Niger	NER	Hygienic disposal of children's stools	19.4	19.4
Niger	NER	ITN/IRS - Households protected from malaria	61.5	52.7
Niger	NER	Injectable antibiotics for neonatal sepsis	29.8	24.3
Niger	NER	ORS - oral rehydration solution	44.3	38.0
Niger	NER	Antibiotics for treatment of dysentery	16.8	14.4
Niger	NER	Zinc for treatment of diarrhea	10.3	8.8
Niger	NER	Oral antibiotics for pneumonia	53.2	45.6
Niger	NER	Vitamin A for treatment of measles	53.0	45.4
Niger	NER	ACTs- Artemisinin compounds for treatment of malaria	11.6	9.9
Niger	NER	SAM - treatment for severe acute malnutrition	40.0	34.3
Niger	NER	BCG vaccine	87.0	70.9
Niger	NER	Polio vaccine	79.0	64.3
Niger	NER	DPT vaccine	79.0	64.3
Niger	NER	H. influenzae type b vaccine	79.0	74.3
Niger	NER	HepB vaccine	79.0	64.3
Niger	NER	Pneumococcal vaccine	79.0	74.3
Niger	NER	Rotavirus vaccine	79.0	74.3
Niger	NER	Meningococcal A	80.0	65.2
Niger	NER	Measles vaccine	78.0	63.5
Niger	NER	Global wasting (<-2 SD) rate	18.5	20.3
Niger	NER	Contraceptive prevalence (CPR)	17.75	16.0
Nigeria	NGA	TT - Tetanus toxoid vaccination	55.0	44.8
Nigeria	NGA	IPTp - Intermittent preventive treatment of malaria during pregnancy	40.4	32.9

Nigeria	NGA	Syphilis detection and treatment	18.3	14.9
Nigeria	NGA	Iron supplementation in pregnancy	20.5	16.7
Nigeria	NGA	Hypertensive disorder case management	13.6	11.1
Nigeria	NGA	Diabetes case management	10.6	8.6
Nigeria	NGA	Malaria case management	44.0	35.8
Nigeria	NGA	MgSO4 management of pre-eclampsia	27.0	22.0
Nigeria	NGA	Thermal protection	38.9	33.4
Nigeria	NGA	Clean cord care	37.6	32.2
Nigeria	NGA	Clean birth environment	32.3	27.7
Nigeria	NGA	Immediate drying and additional stimulation	36.1	31.0
Nigeria	NGA	Neonatal resuscitation	21.7	18.6
Nigeria	NGA	Antibiotics for preterm or prolonged PROM	29.5	25.3
Nigeria	NGA	Parenteral administration of anti-convulsants	28.2	24.2
Nigeria	NGA	Parenteral administration of uterotonics	35.2	30.2
Nigeria	NGA	Parenteral administration of antibiotics	29.5	25.3
Nigeria	NGA	Assisted vaginal delivery	10.0	8.6
Nigeria	NGA	Manual removal of placenta	14.7	12.6
Nigeria	NGA	Removal of retained products of conception	13.1	11.2
Nigeria	NGA	Cesarean delivery	3.4	2.9
Nigeria	NGA	Blood transfusion	5.0	4.3
Nigeria	NGA	Induction of labor for pregnancies lasting 41+ weeks	0.7	0.6
Nigeria	NGA	Complementary feeding - education only	22.6	19.4
Nigeria	NGA	Complementary feeding - supplementary feeding and education	22.6	19.4
Nigeria	NGA	Vitamin A supplementation	83.0	71.2
Nigeria	NGA	Improved sanitation - Utilization of latrines or toilets	39.2	39.2
Nigeria	NGA	Improved water source	71.4	71.4
Nigeria	NGA	Water connection in the home	8.0	8.0
Nigeria	NGA	Hand washing with soap	29.3	29.3
Nigeria	NGA	Hygienic disposal of children's stools	56.5	56.5
Nigeria	NGA	ITN/IRS - Households protected from malaria	60.6	52.0
Nigeria	NGA	Injectable antibiotics for neonatal sepsis	39.4	32.1
Nigeria	NGA	ORS - oral rehydration solution	40.0	34.3
Nigeria	NGA	Antibiotics for treatment of dysentery	40.8	35.0
Nigeria	NGA	Zinc for treatment of diarrhea	31.1	26.7

Nigeria	NGA	Oral antibiotics for pneumonia	74.5	63.9
Nigeria	NGA	Vitamin A for treatment of measles	83.0	71.2
Nigeria	NGA	ACTs- Artemisinin compounds for treatment of malaria	5.6	4.8
Nigeria	NGA	SAM - treatment for severe acute malnutrition	5.8	5.0
Nigeria	NGA	BCG vaccine	53.0	43.2
Nigeria	NGA	Polio vaccine	57.0	46.4
Nigeria	NGA	DPT vaccine	57.0	46.4
Nigeria	NGA	H. influenzae type b vaccine	57.0	53.6
Nigeria	NGA	HepB vaccine	57.0	46.4
Nigeria	NGA	Pneumococcal vaccine	57.0	53.6
Nigeria	NGA	Measles vaccine	42.0	34.2
Nigeria	NGA	Global wasting (<-2 SD) rate	10.9	12.0
Nigeria	NGA	Contraceptive prevalence (CPR)	22.3	20.1
Pakistan	PAK	Safe abortion services	35.1	31.7
Pakistan	PAK	TT - Tetanus toxoid vaccination	85.0	69.2
Pakistan	PAK	Syphilis detection and treatment	21.6	17.6
Pakistan	PAK	Iron supplementation in pregnancy	29.4	23.9
Pakistan	PAK	Hypertensive disorder case management	12.6	10.3
Pakistan	PAK	Diabetes case management	9.8	8.0
Pakistan	PAK	Malaria case management	40.5	33.0
Pakistan	PAK	MgSO4 management of pre-eclampsia	24.8	20.2
Pakistan	PAK	Thermal protection	65.1	55.8
Pakistan	PAK	Clean cord care	62.9	53.9
Pakistan	PAK	Clean birth environment	54.0	46.3
Pakistan	PAK	Immediate drying and additional stimulation	60.3	51.7
Pakistan	PAK	Neonatal resuscitation	36.2	31.0
Pakistan	PAK	Antibiotics for preterm or prolonged PROM	49.3	42.3
Pakistan	PAK	Parenteral administration of anti-convulsants	47.1	40.4
Pakistan	PAK	Parenteral administration of uterotonics	58.9	50.5
Pakistan	PAK	Parenteral administration of antibiotics	49.3	42.3
Pakistan	PAK	Assisted vaginal delivery	16.7	14.3
Pakistan	PAK	Manual removal of placenta	24.6	21.1
Pakistan	PAK	Removal of retained products of conception	21.8	18.7
Pakistan	PAK	Cesarean delivery	5.7	4.9
Pakistan	PAK	Blood transfusion	8.3	7.1

Pakistan	PAK	Induction of labor for pregnancies lasting 41+ weeks	1.2	1.0
Pakistan	PAK	Complementary feeding - education only	20.8	17.8
Pakistan	PAK	Complementary feeding - supplementary feeding and education	20.8	17.8
Pakistan	PAK	Vitamin A supplementation	92.0	78.9
Pakistan	PAK	Improved sanitation - Utilization of latrines or toilets	59.9	59.9
Pakistan	PAK	Improved water source	91.5	91.5
Pakistan	PAK	Water connection in the home	26.0	26.0
Pakistan	PAK	Hand washing with soap	65.6	65.6
Pakistan	PAK	Hygienic disposal of children's stools	35.7	35.7
Pakistan	PAK	ITN/IRS - Households protected from malaria	8.4	7.2
Pakistan	PAK	Injectable antibiotics for neonatal sepsis	65.9	53.7
Pakistan	PAK	ORS - oral rehydration solution	37.4	32.1
Pakistan	PAK	Antibiotics for treatment of dysentery	33.7	28.9
Pakistan	PAK	Zinc for treatment of diarrhea	12.5	10.7
Pakistan	PAK	Oral antibiotics for pneumonia	78.6	67.4
Pakistan	PAK	Vitamin A for treatment of measles	92.0	78.9
Pakistan	PAK	BCG vaccine	86.0	70.0
Pakistan	PAK	Polio vaccine	75.0	61.1
Pakistan	PAK	DPT vaccine	75.0	61.1
Pakistan	PAK	H. influenzae type b vaccine	75.0	70.5
Pakistan	PAK	HepB vaccine	75.0	61.1
Pakistan	PAK	Pneumococcal vaccine	79.0	74.3
Pakistan	PAK	Rotavirus vaccine	58.0	54.6
Pakistan	PAK	Measles vaccine	76.0	61.9
Pakistan	PAK	Global wasting (<-2 SD) rate	7.0	7.7
Pakistan	PAK	Contraceptive prevalence (CPR)	37.05	33.4
Panama	PAN	Safe abortion services	0.6	0.5
Panama	PAN	Syphilis detection and treatment	23.1	18.8
Panama	PAN	Hypertensive disorder case management	21.1	17.2
Panama	PAN	Diabetes case management	16.5	13.4
Panama	PAN	Malaria case management	68.1	55.5
Panama	PAN	MgSO4 management of pre-eclampsia	41.8	34.0
Panama	PAN	Thermal protection	90.2	77.3
Panama	PAN	Clean cord care	87.0	74.6

Panama	PAN	Clean birth environment	74.8	64.1
Panama	PAN	Immediate drying and additional stimulation	83.5	71.6
Panama	PAN	Neonatal resuscitation	50.2	43.0
Panama	PAN	Antibiotics for preterm or prolonged PROM	68.3	58.6
Panama	PAN	Parenteral administration of anti-convulsants	65.2	55.9
Panama	PAN	Parenteral administration of uterotonics	81.5	69.9
Panama	PAN	Parenteral administration of antibiotics	68.3	58.6
Panama	PAN	Assisted vaginal delivery	23.1	19.8
Panama	PAN	Manual removal of placenta	34.1	29.2
Panama	PAN	Removal of retained products of conception	30.2	25.9
Panama	PAN	Cesarean delivery	7.9	6.8
Panama	PAN	Blood transfusion	11.5	9.9
Panama	PAN	Induction of labor for pregnancies lasting 41+ weeks	1.6	1.4
Panama	PAN	Improved sanitation - Utilization of latrines or toilets	83.3	83.3
Panama	PAN	Improved water source	96.4	96.4
Panama	PAN	Water connection in the home	89.6	89.6
Panama	PAN	Hygienic disposal of children's stools	42.6	42.6
Panama	PAN	Injectable antibiotics for neonatal sepsis	91.2	74.3
Panama	PAN	ORS - oral rehydration solution	52.4	44.9
Panama	PAN	Oral antibiotics for pneumonia	81.6	70.0
Panama	PAN	SAM - treatment for severe acute malnutrition	4.5	3.9
Panama	PAN	BCG vaccine	99.0	80.6
Panama	PAN	Polio vaccine	88.0	71.7
Panama	PAN	DPT vaccine	88.0	71.7
Panama	PAN	H. influenzae type b vaccine	88.0	82.8
Panama	PAN	HepB vaccine	88.0	71.7
Panama	PAN	Pneumococcal vaccine	92.0	86.5
Panama	PAN	Rotavirus vaccine	95.0	89.4
Panama	PAN	Measles vaccine	98.0	79.8
Panama	PAN	Global wasting (<-2 SD) rate	2.4	2.7
Panama	PAN	Contraceptive prevalence (CPR)	64	57.8
Papua New Guinea	PNG	Safe abortion services	85.0	76.7
Papua New Guinea	PNG	TT - Tetanus toxoid vaccination	70.0	57.0
Papua New Guinea	PNG	Syphilis detection and treatment	17.9	14.6

Papua New Guinea	PNG	Hypertensive disorder case management	11.8	9.6
Papua New Guinea	PNG	Diabetes case management	9.2	7.5
Papua New Guinea	PNG	Malaria case management	38.0	31.0
Papua New Guinea	PNG	MgSO4 management of pre-eclampsia	23.3	19.0
Papua New Guinea	PNG	Thermal protection	54.1	46.4
Papua New Guinea	PNG	Clean cord care	52.2	44.8
Papua New Guinea	PNG	Clean birth environment	44.9	38.5
Papua New Guinea	PNG	Immediate drying and additional stimulation	50.1	43.0
Papua New Guinea	PNG	Neonatal resuscitation	30.1	25.8
Papua New Guinea	PNG	Antibiotics for preterm or prolonged PROM	40.9	35.1
Papua New Guinea	PNG	Parenteral administration of anti-convulsants	39.1	33.5
Papua New Guinea	PNG	Parenteral administration of uterotonics	48.9	41.9
Papua New Guinea	PNG	Parenteral administration of antibiotics	40.9	35.1
Papua New Guinea	PNG	Assisted vaginal delivery	13.8	11.8
Papua New Guinea	PNG	Manual removal of placenta	20.5	17.6
Papua New Guinea	PNG	Removal of retained products of conception	18.1	15.5
Papua New Guinea	PNG	Cesarean delivery	4.8	4.1
Papua New Guinea	PNG	Blood transfusion	6.9	5.9
Papua New Guinea	PNG	Induction of labor for pregnancies lasting 41+ weeks	1.0	0.9
Papua New Guinea	PNG	Complementary feeding - education only	57.1	49.0
Papua New Guinea	PNG	Complementary feeding - supplementary feeding and education	57.1	49.0
Papua New Guinea	PNG	Vitamin A supplementation	15.0	12.9
Papua New Guinea	PNG	Improved sanitation - Utilization of latrines or toilets	12.9	12.9
Papua New Guinea	PNG	Improved water source	41.3	41.3
Papua New Guinea	PNG	Water connection in the home	7.6	7.6
Papua New Guinea	PNG	Hygienic disposal of children's stools	33.2	33.2
Papua New Guinea	PNG	ITN/IRS - Households protected from malaria	32.9	28.2
Papua New Guinea	PNG	Injectable antibiotics for neonatal sepsis	54.7	44.6
Papua New Guinea	PNG	ORS - oral rehydration solution	30.0	25.7
Papua New Guinea	PNG	Zinc for treatment of diarrhea	7.3	6.3
Papua New Guinea	PNG	Oral antibiotics for pneumonia	63.0	54.0
Papua New Guinea	PNG	Vitamin A for treatment of measles	15.0	12.9
Papua New Guinea	PNG	SAM - treatment for severe acute malnutrition	1.7	1.5
Papua New Guinea	PNG	BCG vaccine	69.0	56.2

Papua New Guinea	PNG	Polio vaccine	67.0	54.6
Papua New Guinea	PNG	DPT vaccine	61.0	49.7
Papua New Guinea	PNG	H. influenzae type b vaccine	61.0	57.4
Papua New Guinea	PNG	HepB vaccine	61.0	49.7
Papua New Guinea	PNG	Pneumococcal vaccine	43.0	40.4
Papua New Guinea	PNG	Measles vaccine	62.0	50.5
Papua New Guinea	PNG	Global wasting (<-2 SD) rate	10.6	11.7
Papua New Guinea	PNG	Contraceptive prevalence (CPR)	38.05	34.3
Paraguay	PRY	Safe abortion services	0.2	0.2
Paraguay	PRY	TT - Tetanus toxoid vaccination	95.0	77.4
Paraguay	PRY	Syphilis detection and treatment	24.4	19.9
Paraguay	PRY	Hypertensive disorder case management	22.5	18.3
Paraguay	PRY	Diabetes case management	17.5	14.3
Paraguay	PRY	Malaria case management	72.5	59.1
Paraguay	PRY	MgSO4 management of pre-eclampsia	44.5	36.2
Paraguay	PRY	Thermal protection	92.1	79.0
Paraguay	PRY	Clean cord care	88.9	76.2
Paraguay	PRY	Clean birth environment	76.4	65.5
Paraguay	PRY	Immediate drying and additional stimulation	85.3	73.1
Paraguay	PRY	Neonatal resuscitation	51.3	44.0
Paraguay	PRY	Antibiotics for preterm or prolonged PROM	69.8	59.8
Paraguay	PRY	Parenteral administration of anti-convulsants	66.7	57.2
Paraguay	PRY	Parenteral administration of uterotonics	83.3	71.4
Paraguay	PRY	Parenteral administration of antibiotics	69.8	59.8
Paraguay	PRY	Assisted vaginal delivery	23.6	20.2
Paraguay	PRY	Manual removal of placenta	34.9	29.9
Paraguay	PRY	Removal of retained products of conception	30.9	26.5
Paraguay	PRY	Cesarean delivery	8.1	6.9
Paraguay	PRY	Blood transfusion	11.7	10.0
Paraguay	PRY	Induction of labor for pregnancies lasting 41+ weeks	1.6	1.4
Paraguay	PRY	Complementary feeding - education only	70.4	60.4
Paraguay	PRY	Complementary feeding - supplementary feeding and education	70.4	60.4
Paraguay	PRY	Improved sanitation - Utilization of latrines or toilets	89.8	89.8
Paraguay	PRY	Improved water source	99.0	99.0

Paraguay	PRY	Water connection in the home	92.7	92.7
Paraguay	PRY	Hygienic disposal of children's stools	22.2	22.2
Paraguay	PRY	Injectable antibiotics for neonatal sepsis	93.2	75.9
Paraguay	PRY	ORS - oral rehydration solution	28.2	24.2
Paraguay	PRY	Zinc for treatment of diarrhea	7.1	6.1
Paraguay	PRY	Oral antibiotics for pneumonia	89.4	76.6
Paraguay	PRY	BCG vaccine	91.0	74.1
Paraguay	PRY	Polio vaccine	88.0	71.7
Paraguay	PRY	DPT vaccine	88.0	71.7
Paraguay	PRY	H. influenzae type b vaccine	88.0	82.8
Paraguay	PRY	HepB vaccine	88.0	71.7
Paraguay	PRY	Pneumococcal vaccine	94.0	88.4
Paraguay	PRY	Rotavirus vaccine	91.0	85.6
Paraguay	PRY	Measles vaccine	92.0	74.9
Paraguay	PRY	Global wasting (<-2 SD) rate	1.0	1.1
Paraguay	PRY	Contraceptive prevalence (CPR)	69.85	63.0
Peru	PER	Safe abortion services	0.2	0.2
Peru	PER	TT - Tetanus toxoid vaccination	95.0	77.4
Peru	PER	Syphilis detection and treatment	24.1	19.6
Peru	PER	Iron supplementation in pregnancy	59.7	48.6
Peru	PER	Hypertensive disorder case management	23.0	18.7
Peru	PER	Diabetes case management	17.9	14.6
Peru	PER	Malaria case management	74.2	60.4
Peru	PER	MgSO4 management of pre-eclampsia	45.5	37.1
Peru	PER	Thermal protection	89.7	76.9
Peru	PER	Clean cord care	86.6	74.2
Peru	PER	Clean birth environment	74.4	63.8
Peru	PER	Immediate drying and additional stimulation	83.1	71.2
Peru	PER	Neonatal resuscitation	49.9	42.8
Peru	PER	Antibiotics for preterm or prolonged PROM	67.9	58.2
Peru	PER	Parenteral administration of anti-convulsants	64.9	55.6
Peru	PER	Parenteral administration of uterotonics	81.1	69.5
Peru	PER	Parenteral administration of antibiotics	67.9	58.2
Peru	PER	Assisted vaginal delivery	22.9	19.6
Peru	PER	Manual removal of placenta	33.9	29.1

Peru	PER	Removal of retained products of conception	30.1	25.8
Peru	PER	Cesarean delivery	7.9	6.8
Peru	PER	Blood transfusion	11.4	9.8
Peru	PER	Induction of labor for pregnancies lasting 41+ weeks	1.6	1.4
Peru	PER	Complementary feeding - education only	82.6	70.8
Peru	PER	Complementary feeding - supplementary feeding and education	82.6	70.8
Peru	PER	Vitamin A supplementation	4.5	3.9
Peru	PER	Improved sanitation - Utilization of latrines or toilets	74.3	74.3
Peru	PER	Improved water source	91.1	91.1
Peru	PER	Water connection in the home	79.4	79.4
Peru	PER	Hand washing with soap	14.0	14.0
Peru	PER	Hygienic disposal of children's stools	12.8	12.8
Peru	PER	Injectable antibiotics for neonatal sepsis	90.7	73.9
Peru	PER	ORS - oral rehydration solution	34.6	29.7
Peru	PER	Antibiotics for treatment of dysentery	18.9	16.2
Peru	PER	Zinc for treatment of diarrhea	0.9	0.8
Peru	PER	Oral antibiotics for pneumonia	67.5	57.9
Peru	PER	Vitamin A for treatment of measles	4.5	3.9
Peru	PER	BCG vaccine	81.0	66.0
Peru	PER	Polio vaccine	83.0	67.6
Peru	PER	DPT vaccine	84.0	68.4
Peru	PER	H. influenzae type b vaccine	84.0	79.0
Peru	PER	HepB vaccine	84.0	68.4
Peru	PER	Pneumococcal vaccine	82.0	77.1
Peru	PER	Rotavirus vaccine	85.0	80.0
Peru	PER	Measles vaccine	83.0	67.6
Peru	PER	Global wasting (<-2 SD) rate	0.7	0.7
Peru	PER	Contraceptive prevalence (CPR)	75.5	68.1
Philippines	PHL	Safe abortion services	39.0	35.2
Philippines	PHL	TT - Tetanus toxoid vaccination	90.0	73.3
Philippines	PHL	Syphilis detection and treatment	23.1	18.8
Philippines	PHL	Iron supplementation in pregnancy	50.6	41.2
Philippines	PHL	Hypertensive disorder case management	20.7	16.9
Philippines	PHL	Diabetes case management	16.2	13.2

Philippines	PHL	Malaria case management	66.8	54.4
Philippines	PHL	MgSO4 management of pre-eclampsia	41.0	33.4
Philippines	PHL	Thermal protection	76.8	65.8
Philippines	PHL	Clean cord care	74.2	63.6
Philippines	PHL	Clean birth environment	63.8	54.7
Philippines	PHL	Immediate drying and additional stimulation	71.2	61.0
Philippines	PHL	Neonatal resuscitation	42.8	36.7
Philippines	PHL	Antibiotics for preterm or prolonged PROM	58.2	49.9
Philippines	PHL	Parenteral administration of anti-convulsants	55.6	47.7
Philippines	PHL	Parenteral administration of uterotonics	69.5	59.6
Philippines	PHL	Parenteral administration of antibiotics	58.2	49.9
Philippines	PHL	Assisted vaginal delivery	19.7	16.9
Philippines	PHL	Manual removal of placenta	29.1	24.9
Philippines	PHL	Removal of retained products of conception	25.8	22.1
Philippines	PHL	Cesarean delivery	6.8	5.8
Philippines	PHL	Blood transfusion	9.8	8.4
Philippines	PHL	Induction of labor for pregnancies lasting 41+ weeks	1.4	1.2
Philippines	PHL	Complementary feeding - education only	64.8	55.6
Philippines	PHL	Complementary feeding - supplementary feeding and education	64.8	55.6
Philippines	PHL	Vitamin A supplementation	68.0	58.3
Philippines	PHL	Improved sanitation - Utilization of latrines or toilets	76.5	76.5
Philippines	PHL	Improved water source	93.6	93.6
Philippines	PHL	Water connection in the home	37.5	37.5
Philippines	PHL	Hand washing with soap	86.6	86.6
Philippines	PHL	Hygienic disposal of children's stools	10.2	10.2
Philippines	PHL	Injectable antibiotics for neonatal sepsis	77.7	63.3
Philippines	PHL	ORS - oral rehydration solution	44.8	38.4
Philippines	PHL	Antibiotics for treatment of dysentery	38.7	33.2
Philippines	PHL	Zinc for treatment of diarrhea	24.4	20.9
Philippines	PHL	Oral antibiotics for pneumonia	66.6	57.1
Philippines	PHL	Vitamin A for treatment of measles	68.0	58.3
Philippines	PHL	SAM - treatment for severe acute malnutrition	0.1	0.1
Philippines	PHL	BCG vaccine	75.0	61.1
Philippines	PHL	Polio vaccine	66.0	53.8

Philippines	PHL	DPT vaccine	65.0	52.9
Philippines	PHL	H. influenzae type b vaccine	65.0	61.1
Philippines	PHL	HepB vaccine	65.0	52.9
Philippines	PHL	Pneumococcal vaccine	43.0	40.4
Philippines	PHL	Rotavirus vaccine	2.0	1.9
Philippines	PHL	Measles vaccine	89.0	72.5
Philippines	PHL	Global wasting (<-2 SD) rate	6.9	7.6
Philippines	PHL	Contraceptive prevalence (CPR)	56	50.5
Republic of Moldova	MDA	Safe abortion services	87.5	79.0
Republic of Moldova	MDA	Syphilis detection and treatment	24.4	19.9
Republic of Moldova	MDA	Iron supplementation in pregnancy	40.9	33.3
Republic of Moldova	MDA	Hypertensive disorder case management	22.9	18.7
Republic of Moldova	MDA	Diabetes case management	17.9	14.6
Republic of Moldova	MDA	Malaria case management	73.9	60.2
Republic of Moldova	MDA	MgSO4 management of pre-eclampsia	45.3	36.9
Republic of Moldova	MDA	Thermal protection	97.7	83.8
Republic of Moldova	MDA	Clean cord care	94.3	80.9
Republic of Moldova	MDA	Clean birth environment	81.1	69.5
Republic of Moldova	MDA	Immediate drying and additional stimulation	90.5	77.6
Republic of Moldova	MDA	Neonatal resuscitation	54.4	46.6
Republic of Moldova	MDA	Antibiotics for preterm or prolonged PROM	74.0	63.4
Republic of Moldova	MDA	Parenteral administration of anti-convulsants	70.7	60.6
Republic of Moldova	MDA	Parenteral administration of uterotonics	88.3	75.7
Republic of Moldova	MDA	Parenteral administration of antibiotics	74.0	63.4
Republic of Moldova	MDA	Assisted vaginal delivery	25.0	21.4
Republic of Moldova	MDA	Manual removal of placenta	37.0	31.7
Republic of Moldova	MDA	Removal of retained products of conception	32.8	28.1
Republic of Moldova	MDA	Cesarean delivery	8.6	7.4
Republic of Moldova	MDA	Blood transfusion	12.4	10.6
Republic of Moldova	MDA	Induction of labor for pregnancies lasting 41+ weeks	1.7	1.5
Republic of Moldova	MDA	Complementary feeding - education only	82.0	70.3
Republic of Moldova	MDA	Complementary feeding - supplementary feeding and education	82.0	70.3
Republic of Moldova	MDA	Improved sanitation - Utilization of latrines or toilets	76.3	76.3
Republic of Moldova	MDA	Improved water source	89.1	89.1

Republic of Moldova	MDA	Water connection in the home	58.9	58.9
Republic of Moldova	MDA	Hand washing with soap	89.4	89.4
Republic of Moldova	MDA	Hygienic disposal of children's stools	45.9	45.9
Republic of Moldova	MDA	Injectable antibiotics for neonatal sepsis	98.9	80.6
Republic of Moldova	MDA	ORS - oral rehydration solution	41.5	35.6
Republic of Moldova	MDA	Antibiotics for treatment of dysentery	24.0	20.6
Republic of Moldova	MDA	Oral antibiotics for pneumonia	79.2	67.9
Republic of Moldova	MDA	BCG vaccine	96.0	78.2
Republic of Moldova	MDA	Polio vaccine	94.0	76.6
Republic of Moldova	MDA	DPT vaccine	93.0	75.7
Republic of Moldova	MDA	H. influenzae type b vaccine	92.0	86.5
Republic of Moldova	MDA	HepB vaccine	94.0	76.6
Republic of Moldova	MDA	Pneumococcal vaccine	94.0	88.4
Republic of Moldova	MDA	Rotavirus vaccine	75.0	70.5
Republic of Moldova	MDA	Measles vaccine	93.0	75.7
Republic of Moldova	MDA	Global wasting (<-2 SD) rate	1.9	2.1
Republic of Moldova	MDA	Contraceptive prevalence (CPR)	62.9	56.8
Republic of North Macedonia	MKD	Safe abortion services	100.0	90.3
Republic of North Macedonia	MKD	Syphilis detection and treatment	24.4	19.9
Republic of North Macedonia	MKD	Hypertensive disorder case management	22.6	18.4
Republic of North Macedonia	MKD	Diabetes case management	17.6	14.3
Republic of North Macedonia	MKD	Malaria case management	72.8	59.3
Republic of North Macedonia	MKD	MgSO4 management of pre-eclampsia	44.6	36.3
Republic of North Macedonia	MKD	Thermal protection	97.1	83.3
Republic of North Macedonia	MKD	Clean cord care	93.8	80.4
Republic of North Macedonia	MKD	Clean birth environment	80.6	69.1
Republic of North Macedonia	MKD	Immediate drying and additional stimulation	90.0	77.2
Republic of North Macedonia	MKD	Neonatal resuscitation	54.0	46.3

Republic of North Macedonia	MKD	Antibiotics for preterm or prolonged PROM	73.5	63.0
Republic of North Macedonia	MKD	Parenteral administration of anti-convulsants	70.3	60.3
Republic of North Macedonia	MKD	Parenteral administration of uterotonics	87.8	75.3
Republic of North Macedonia	MKD	Parenteral administration of antibiotics	73.5	63.0
Republic of North Macedonia	MKD	Assisted vaginal delivery	24.8	21.3
Republic of North Macedonia	MKD	Manual removal of placenta	36.8	31.6
Republic of North Macedonia	MKD	Removal of retained products of conception	32.6	28.0
Republic of North Macedonia	MKD	Cesarean delivery	8.6	7.4
Republic of North Macedonia	MKD	Blood transfusion	12.4	10.6
Republic of North Macedonia	MKD	Induction of labor for pregnancies lasting 41+ weeks	1.7	1.5
Republic of North Macedonia	MKD	Improved sanitation - Utilization of latrines or toilets	99.0	99.0
Republic of North Macedonia	MKD	Improved water source	93.1	93.1
Republic of North Macedonia	MKD	Water connection in the home	86.2	86.2
Republic of North Macedonia	MKD	Hygienic disposal of children's stools	17.3	17.3
Republic of North Macedonia	MKD	Injectable antibiotics for neonatal sepsis	98.3	80.1
Republic of North Macedonia	MKD	ORS - oral rehydration solution	62.0	53.2
Republic of North Macedonia	MKD	Oral antibiotics for pneumonia	77.4	66.4
Republic of North Macedonia	MKD	BCG vaccine	97.0	79.0
Republic of North Macedonia	MKD	Polio vaccine	91.0	74.1
Republic of North Macedonia	MKD	DPT vaccine	91.0	74.1
Republic of North Macedonia	MKD	H. influenzae type b vaccine	91.0	85.6

Republic of North Macedonia	MKD	HepB vaccine	91.0	74.1
Republic of North Macedonia	MKD	Measles vaccine	83.0	67.6
Republic of North Macedonia	MKD	Global wasting (<-2 SD) rate	1.8	2.0
Republic of North Macedonia	MKD	Contraceptive prevalence (CPR)	46.35	41.8
Rwanda	RWA	Safe abortion services	3.3	3.0
Rwanda	RWA	TT - Tetanus toxoid vaccination	90.0	73.3
Rwanda	RWA	IPTp - Intermittent preventive treatment of malaria during pregnancy	17.7	14.4
Rwanda	RWA	Syphilis detection and treatment	52.0	42.4
Rwanda	RWA	Iron supplementation in pregnancy	3.4	2.8
Rwanda	RWA	Hypertensive disorder case management	1.9	1.5
Rwanda	RWA	Diabetes case management	8.3	6.8
Rwanda	RWA	Malaria case management	34.4	28.0
Rwanda	RWA	MgSO4 management of pre-eclampsia	7.7	6.3
Rwanda	RWA	Thermal protection	89.6	76.8
Rwanda	RWA	Clean cord care	81.4	69.8
Rwanda	RWA	Clean birth environment	78.6	67.4
Rwanda	RWA	Immediate drying and additional stimulation	73.4	62.9
Rwanda	RWA	Neonatal resuscitation	36.2	31.0
Rwanda	RWA	Antibiotics for preterm or prolonged PROM	36.7	31.5
Rwanda	RWA	Parenteral administration of anti-convulsants	60.1	51.5
Rwanda	RWA	Parenteral administration of uterotonics	33.5	28.7
Rwanda	RWA	Parenteral administration of antibiotics	36.7	31.5
Rwanda	RWA	Assisted vaginal delivery	20.3	17.4
Rwanda	RWA	Manual removal of placenta	25.7	22.0
Rwanda	RWA	Removal of retained products of conception	30.0	25.7
Rwanda	RWA	Cesarean delivery	12.1	10.4
Rwanda	RWA	Blood transfusion	6.0	5.1
Rwanda	RWA	Induction of labor for pregnancies lasting 41+ weeks	1.6	1.4
Rwanda	RWA	Complementary feeding - education only	30.1	25.8
Rwanda	RWA	Complementary feeding - supplementary feeding and education	30.1	25.8
Rwanda	RWA	Vitamin A supplementation	98.0	84.0

Rwanda	RWA	Improved sanitation - Utilization of latrines or toilets	66.6	66.6
Rwanda	RWA	Improved water source	57.7	57.7
Rwanda	RWA	Water connection in the home	21.5	21.5
Rwanda	RWA	Hand washing with soap	37.6	37.6
Rwanda	RWA	Hygienic disposal of children's stools	77.9	77.9
Rwanda	RWA	ITN/IRS - Households protected from malaria	84.1	72.1
Rwanda	RWA	Injectable antibiotics for neonatal sepsis	90.7	73.9
Rwanda	RWA	ORS - oral rehydration solution	27.5	23.6
Rwanda	RWA	Antibiotics for treatment of dysentery	15.1	12.9
Rwanda	RWA	Zinc for treatment of diarrhea	0.2	0.2
Rwanda	RWA	Oral antibiotics for pneumonia	53.9	46.2
Rwanda	RWA	Vitamin A for treatment of measles	98.0	84.0
Rwanda	RWA	ACTs- Artemisinin compounds for treatment of malaria	7.4	6.3
Rwanda	RWA	BCG vaccine	97.0	79.0
Rwanda	RWA	Polio vaccine	97.0	79.0
Rwanda	RWA	DPT vaccine	89.0	72.5
Rwanda	RWA	H. influenzae type b vaccine	97.0	91.2
Rwanda	RWA	HepB vaccine	97.0	79.0
Rwanda	RWA	Pneumococcal vaccine	97.0	91.2
Rwanda	RWA	Rotavirus vaccine	98.0	92.2
Rwanda	RWA	Measles vaccine	95.0	77.4
Rwanda	RWA	Global wasting (<-2 SD) rate	2.3	2.6
Rwanda	RWA	Contraceptive prevalence (CPR)	57.35	51.8
Saint Lucia	LCA	Safe abortion services	53.7	48.5
Saint Lucia	LCA	Syphilis detection and treatment	24.0	19.5
Saint Lucia	LCA	Hypertensive disorder case management	21.7	17.7
Saint Lucia	LCA	Diabetes case management	16.9	13.8
Saint Lucia	LCA	Malaria case management	70.0	57.0
Saint Lucia	LCA	MgSO4 management of pre-eclampsia	42.9	34.9
Saint Lucia	LCA	Thermal protection	97.6	83.7
Saint Lucia	LCA	Clean cord care	94.2	80.8
Saint Lucia	LCA	Clean birth environment	81.0	69.4
Saint Lucia	LCA	Immediate drying and additional stimulation	90.4	77.5
Saint Lucia	LCA	Neonatal resuscitation	54.3	46.6

Saint Lucia	LCA	Antibiotics for preterm or prolonged PROM	73.9	63.4
Saint Lucia	LCA	Parenteral administration of anti-convulsants	70.6	60.5
Saint Lucia	LCA	Parenteral administration of uterotonics	88.2	75.6
Saint Lucia	LCA	Parenteral administration of antibiotics	73.9	63.4
Saint Lucia	LCA	Assisted vaginal delivery	25.0	21.4
Saint Lucia	LCA	Manual removal of placenta	36.9	31.6
Saint Lucia	LCA	Removal of retained products of conception	32.7	28.0
Saint Lucia	LCA	Cesarean delivery	8.6	7.4
Saint Lucia	LCA	Blood transfusion	12.4	10.6
Saint Lucia	LCA	Induction of labor for pregnancies lasting 41+ weeks	1.7	1.5
Saint Lucia	LCA	Improved sanitation - Utilization of latrines or toilets	88.4	88.4
Saint Lucia	LCA	Improved water source	98.2	98.2
Saint Lucia	LCA	Water connection in the home	94.2	94.2
Saint Lucia	LCA	Hand washing with soap	89.0	89.0
Saint Lucia	LCA	Hygienic disposal of children's stools	26.6	26.6
Saint Lucia	LCA	Injectable antibiotics for neonatal sepsis	98.7	80.4
Saint Lucia	LCA	Oral antibiotics for pneumonia	27.0	23.1
Saint Lucia	LCA	BCG vaccine	99.0	80.6
Saint Lucia	LCA	Polio vaccine	95.0	77.4
Saint Lucia	LCA	DPT vaccine	95.0	77.4
Saint Lucia	LCA	H. influenzae type b vaccine	95.0	89.4
Saint Lucia	LCA	HepB vaccine	95.0	77.4
Saint Lucia	LCA	Measles vaccine	87.0	70.9
Saint Lucia	LCA	Global wasting (<-2 SD) rate	2.4	2.7
Saint Lucia	LCA	Contraceptive prevalence (CPR)	59.4	53.6
Samoa	WSM	Safe abortion services	85.0	76.7
Samoa	WSM	Syphilis detection and treatment	14.4	11.7
Samoa	WSM	Iron supplementation in pregnancy	3.4	2.8
Samoa	WSM	Hypertensive disorder case management	14.0	11.4
Samoa	WSM	Diabetes case management	10.9	8.9
Samoa	WSM	Malaria case management	45.3	36.9
Samoa	WSM	MgSO4 management of pre-eclampsia	27.8	22.6
Samoa	WSM	Thermal protection	79.6	68.2
Samoa	WSM	Clean cord care	76.8	65.8

Samoa	WSM	Clean birth environment	66.0	56.6
Samoa	WSM	Immediate drying and additional stimulation	73.7	63.2
Samoa	WSM	Neonatal resuscitation	44.3	38.0
Samoa	WSM	Antibiotics for preterm or prolonged PROM	60.2	51.6
Samoa	WSM	Parenteral administration of anti-convulsants	57.6	49.4
Samoa	WSM	Parenteral administration of uterotonics	71.9	61.6
Samoa	WSM	Parenteral administration of antibiotics	60.2	51.6
Samoa	WSM	Assisted vaginal delivery	20.3	17.4
Samoa	WSM	Manual removal of placenta	30.1	25.8
Samoa	WSM	Removal of retained products of conception	26.7	22.9
Samoa	WSM	Cesarean delivery	7.0	6.0
Samoa	WSM	Blood transfusion	10.1	8.7
Samoa	WSM	Induction of labor for pregnancies lasting 41+ weeks	1.4	1.2
Samoa	WSM	Improved sanitation - Utilization of latrines or toilets	98.2	98.2
Samoa	WSM	Improved water source	97.4	97.4
Samoa	WSM	Water connection in the home	82.7	82.7
Samoa	WSM	Hygienic disposal of children's stools	38.0	38.0
Samoa	WSM	Injectable antibiotics for neonatal sepsis	80.5	65.6
Samoa	WSM	ORS - oral rehydration solution	68.0	58.3
Samoa	WSM	Antibiotics for treatment of dysentery	1.0	0.9
Samoa	WSM	BCG vaccine	62.0	50.5
Samoa	WSM	Polio vaccine	31.0	25.2
Samoa	WSM	DPT vaccine	34.0	27.7
Samoa	WSM	H. influenzae type b vaccine	34.0	32.0
Samoa	WSM	HepB vaccine	34.0	27.7
Samoa	WSM	Measles vaccine	58.0	47.2
Samoa	WSM	Global wasting (<-2 SD) rate	10.6	11.7
Samoa	WSM	Contraceptive prevalence (CPR)	30.2	27.3
São Tomé and Príncipe	STP	TT - Tetanus toxoid vaccination	99.0	80.6
São Tomé and Príncipe	STP	IPTp - Intermittent preventive treatment of malaria during pregnancy	56.9	46.3
São Tomé and Príncipe	STP	Syphilis detection and treatment	24.1	19.6
São Tomé and Príncipe	STP	Iron supplementation in pregnancy	53.5	43.6
São Tomé and Príncipe	STP	Hypertensive disorder case management	20.1	16.4
São Tomé and Príncipe	STP	Diabetes case management	15.7	12.8

São Tomé and Príncipe	STP	Malaria case management	64.8	52.8
São Tomé and Príncipe	STP	MgSO4 management of pre-eclampsia	39.8	32.4
São Tomé and Príncipe	STP	Thermal protection	89.9	77.1
São Tomé and Príncipe	STP	Clean cord care	86.8	74.4
São Tomé and Príncipe	STP	Clean birth environment	74.6	64.0
São Tomé and Príncipe	STP	Immediate drying and additional stimulation	83.3	71.4
São Tomé and Príncipe	STP	Neonatal resuscitation	50.0	42.9
São Tomé and Príncipe	STP	Antibiotics for preterm or prolonged PROM	68.1	58.4
São Tomé and Príncipe	STP	Parenteral administration of anti-convulsants	65.1	55.8
São Tomé and Príncipe	STP	Parenteral administration of uterotonics	81.3	69.7
São Tomé and Príncipe	STP	Parenteral administration of antibiotics	68.1	58.4
São Tomé and Príncipe	STP	Assisted vaginal delivery	23.0	19.7
São Tomé and Príncipe	STP	Manual removal of placenta	34.0	29.2
São Tomé and Príncipe	STP	Removal of retained products of conception	30.1	25.8
São Tomé and Príncipe	STP	Cesarean delivery	7.9	6.8
São Tomé and Príncipe	STP	Blood transfusion	11.5	9.9
São Tomé and Príncipe	STP	Induction of labor for pregnancies lasting 41+ weeks	1.6	1.4
São Tomé and Príncipe	STP	Complementary feeding - education only	47.4	40.6
São Tomé and Príncipe	STP	Complementary feeding - supplementary feeding and education	47.4	40.6
São Tomé and Príncipe	STP	Vitamin A supplementation	23.0	19.7
São Tomé and Príncipe	STP	Improved sanitation - Utilization of latrines or toilets	43.0	43.0
São Tomé and Príncipe	STP	Improved water source	84.3	84.3
São Tomé and Príncipe	STP	Water connection in the home	79.0	79.0
São Tomé and Príncipe	STP	Hand washing with soap	40.6	40.6
São Tomé and Príncipe	STP	Hygienic disposal of children's stools	28.9	28.9
São Tomé and Príncipe	STP	ITN/IRS - Households protected from malaria	96.7	82.9
São Tomé and Príncipe	STP	Injectable antibiotics for neonatal sepsis	91.0	74.1
São Tomé and Príncipe	STP	ORS - oral rehydration solution	49.1	42.1
São Tomé and Príncipe	STP	Oral antibiotics for pneumonia	68.9	59.1
São Tomé and Príncipe	STP	Vitamin A for treatment of measles	23.0	19.7
São Tomé and Príncipe	STP	ACTs- Artemisinin compounds for treatment of malaria	0.1	0.1
São Tomé and Príncipe	STP	BCG vaccine	96.0	78.2
São Tomé and Príncipe	STP	Polio vaccine	95.0	77.4

São Tomé and Príncipe	STP	DPT vaccine	95.0	77.4
São Tomé and Príncipe	STP	H. influenzae type b vaccine	95.0	89.4
São Tomé and Príncipe	STP	HepB vaccine	95.0	77.4
São Tomé and Príncipe	STP	Pneumococcal vaccine	95.0	89.4
São Tomé and Príncipe	STP	Rotavirus vaccine	95.0	89.4
São Tomé and Príncipe	STP	Measles vaccine	90.0	73.3
São Tomé and Príncipe	STP	Global wasting (<-2 SD) rate	4.2	4.6
São Tomé and Príncipe	STP	Contraceptive prevalence (CPR)	45.3	40.9
Senegal	SEN	TT - Tetanus toxoid vaccination	95.0	77.4
Senegal	SEN	IPTp - Intermittent preventive treatment of malaria during pregnancy	62.6	51.0
Senegal	SEN	Syphilis detection and treatment	5.9	4.8
Senegal	SEN	Iron supplementation in pregnancy	63.1	51.4
Senegal	SEN	Hypertensive disorder case management	0.3	0.2
Senegal	SEN	Diabetes case management	33.5	27.3
Senegal	SEN	Malaria case management	47.7	38.9
Senegal	SEN	MgSO4 management of pre-eclampsia	36.5	29.7
Senegal	SEN	Thermal protection	69.1	59.2
Senegal	SEN	Clean cord care	68.7	58.9
Senegal	SEN	Clean birth environment	68.5	58.7
Senegal	SEN	Immediate drying and additional stimulation	68.9	59.1
Senegal	SEN	Neonatal resuscitation	50.5	43.3
Senegal	SEN	Antibiotics for preterm or prolonged PROM	52.3	44.8
Senegal	SEN	Parenteral administration of anti-convulsants	59.2	50.8
Senegal	SEN	Parenteral administration of uterotonics	61.1	52.4
Senegal	SEN	Parenteral administration of antibiotics	52.3	44.8
Senegal	SEN	Assisted vaginal delivery	7.4	6.3
Senegal	SEN	Manual removal of placenta	36.0	30.9
Senegal	SEN	Removal of retained products of conception	35.1	30.1
Senegal	SEN	Cesarean delivery	6.4	5.5
Senegal	SEN	Blood transfusion	7.6	6.5
Senegal	SEN	Induction of labor for pregnancies lasting 41+ weeks	8.1	6.9
Senegal	SEN	Complementary feeding - education only	25.3	21.7
Senegal	SEN	Complementary feeding - supplementary feeding and education	25.3	21.7
Senegal	SEN	Vitamin A supplementation	58.0	49.7

Senegal	SEN	Improved sanitation - Utilization of latrines or toilets	51.5	51.5
Senegal	SEN	Improved water source	80.7	80.7
Senegal	SEN	Water connection in the home	58.9	58.9
Senegal	SEN	Hand washing with soap	28.5	28.5
Senegal	SEN	Hygienic disposal of children's stools	57.0	57.0
Senegal	SEN	ITN/IRS - Households protected from malaria	84.5	72.4
Senegal	SEN	Injectable antibiotics for neonatal sepsis	69.9	56.9
Senegal	SEN	ORS - oral rehydration solution	27.9	23.9
Senegal	SEN	Antibiotics for treatment of dysentery	12.8	11.0
Senegal	SEN	Zinc for treatment of diarrhea	26.9	23.1
Senegal	SEN	Oral antibiotics for pneumonia	52.0	44.6
Senegal	SEN	Vitamin A for treatment of measles	58.0	49.7
Senegal	SEN	ACTs- Artemisinin compounds for treatment of malaria	1.5	1.3
Senegal	SEN	BCG vaccine	83.0	67.6
Senegal	SEN	Polio vaccine	81.0	66.0
Senegal	SEN	DPT vaccine	81.0	66.0
Senegal	SEN	H. influenzae type b vaccine	82.0	77.1
Senegal	SEN	HepB vaccine	82.0	66.8
Senegal	SEN	Pneumococcal vaccine	81.0	76.2
Senegal	SEN	Rotavirus vaccine	80.0	75.3
Senegal	SEN	Measles vaccine	90.0	73.3
Senegal	SEN	Global wasting (<-2 SD) rate	9.0	9.8
Senegal	SEN	Contraceptive prevalence (CPR)	29.95	27.0
Serbia	SRB	Safe abortion services	100.0	90.3
Serbia	SRB	Syphilis detection and treatment	6.0	4.9
Serbia	SRB	Hypertensive disorder case management	0.6	0.5
Serbia	SRB	Diabetes case management	56.3	45.9
Serbia	SRB	Malaria case management	80.1	65.2
Serbia	SRB	MgSO4 management of pre-eclampsia	61.2	49.8
Serbia	SRB	Thermal protection	97.1	83.3
Serbia	SRB	Clean cord care	93.8	80.4
Serbia	SRB	Clean birth environment	80.6	69.1
Serbia	SRB	Immediate drying and additional stimulation	90.0	77.2
Serbia	SRB	Neonatal resuscitation	54.0	46.3

Serbia	SRB	Antibiotics for preterm or prolonged PROM	73.5	63.0
Serbia	SRB	Parenteral administration of anti-convulsants	70.3	60.3
Serbia	SRB	Parenteral administration of uterotonics	87.8	75.3
Serbia	SRB	Parenteral administration of antibiotics	73.5	63.0
Serbia	SRB	Assisted vaginal delivery	24.8	21.3
Serbia	SRB	Manual removal of placenta	36.8	31.6
Serbia	SRB	Removal of retained products of conception	32.6	28.0
Serbia	SRB	Cesarean delivery	8.6	7.4
Serbia	SRB	Blood transfusion	12.4	10.6
Serbia	SRB	Induction of labor for pregnancies lasting 41+ weeks	1.7	1.5
Serbia	SRB	Complementary feeding - education only	89.6	76.8
Serbia	SRB	Complementary feeding - supplementary feeding and education	89.6	76.8
Serbia	SRB	Improved sanitation - Utilization of latrines or toilets	97.6	97.6
Serbia	SRB	Improved water source	85.5	85.5
Serbia	SRB	Water connection in the home	82.1	82.1
Serbia	SRB	Hand washing with soap	98.6	98.6
Serbia	SRB	Hygienic disposal of children's stools	25.7	25.7
Serbia	SRB	Injectable antibiotics for neonatal sepsis	98.3	80.1
Serbia	SRB	ORS - oral rehydration solution	36.0	30.9
Serbia	SRB	Oral antibiotics for pneumonia	89.7	76.9
Serbia	SRB	BCG vaccine	98.0	79.8
Serbia	SRB	Polio vaccine	96.0	78.2
Serbia	SRB	DPT vaccine	96.0	78.2
Serbia	SRB	H. influenzae type b vaccine	96.0	90.3
Serbia	SRB	HepB vaccine	91.0	74.1
Serbia	SRB	Pneumococcal vaccine	48.0	45.2
Serbia	SRB	Measles vaccine	86.0	70.0
Serbia	SRB	Global wasting (<-2 SD) rate	3.8	4.2
Serbia	SRB	Contraceptive prevalence (CPR)	59.25	53.5
Sierra Leone	SLE	TT - Tetanus toxoid vaccination	90.0	73.3
Sierra Leone	SLE	IPTp - Intermittent preventive treatment of malaria during pregnancy	66.6	54.2
Sierra Leone	SLE	Syphilis detection and treatment	19.9	16.2
Sierra Leone	SLE	Iron supplementation in pregnancy	30.0	24.4

Sierra Leone	SLE	Hypertensive disorder case management	40.8	33.2
Sierra Leone	SLE	Diabetes case management	11.4	9.3
Sierra Leone	SLE	Malaria case management	70.0	57.0
Sierra Leone	SLE	MgSO4 management of pre-eclampsia	67.1	54.7
Sierra Leone	SLE	Thermal protection	75.7	64.9
Sierra Leone	SLE	Clean cord care	69.8	59.8
Sierra Leone	SLE	Clean birth environment	65.3	56.0
Sierra Leone	SLE	Immediate drying and additional stimulation	71.3	61.1
Sierra Leone	SLE	Neonatal resuscitation	32.5	27.9
Sierra Leone	SLE	Antibiotics for preterm or prolonged PROM	66.2	56.8
Sierra Leone	SLE	Parenteral administration of anti-convulsants	67.6	58.0
Sierra Leone	SLE	Parenteral administration of uterotonics	69.5	59.6
Sierra Leone	SLE	Parenteral administration of antibiotics	66.2	56.8
Sierra Leone	SLE	Assisted vaginal delivery	43.5	37.3
Sierra Leone	SLE	Manual removal of placenta	16.2	13.9
Sierra Leone	SLE	Removal of retained products of conception	34.4	29.5
Sierra Leone	SLE	Cesarean delivery	1.7	1.5
Sierra Leone	SLE	Blood transfusion	11.7	10.0
Sierra Leone	SLE	Induction of labor for pregnancies lasting 41+ weeks	0.8	0.7
Sierra Leone	SLE	Complementary feeding - education only	24.2	20.7
Sierra Leone	SLE	Complementary feeding - supplementary feeding and education	24.2	20.7
Sierra Leone	SLE	Vitamin A supplementation	98.0	84.0
Sierra Leone	SLE	Improved sanitation - Utilization of latrines or toilets	15.7	15.7
Sierra Leone	SLE	Improved water source	60.8	60.8
Sierra Leone	SLE	Water connection in the home	14.9	14.9
Sierra Leone	SLE	Hand washing with soap	34.3	34.3
Sierra Leone	SLE	Hygienic disposal of children's stools	63.0	63.0
Sierra Leone	SLE	ITN/IRS - Households protected from malaria	72.0	61.7
Sierra Leone	SLE	Injectable antibiotics for neonatal sepsis	76.6	62.4
Sierra Leone	SLE	ORS - oral rehydration solution	77.7	66.6
Sierra Leone	SLE	Antibiotics for treatment of dysentery	50.5	43.3
Sierra Leone	SLE	Zinc for treatment of diarrhea	50.0	42.9
Sierra Leone	SLE	Oral antibiotics for pneumonia	73.3	62.8
Sierra Leone	SLE	Vitamin A for treatment of measles	98.0	84.0

Sierra Leone	SLE	ACTs- Artemisinin compounds for treatment of malaria	13.9	11.9
Sierra Leone	SLE	BCG vaccine	90.0	73.3
Sierra Leone	SLE	Polio vaccine	90.0	73.3
Sierra Leone	SLE	DPT vaccine	90.0	73.3
Sierra Leone	SLE	H. influenzae type b vaccine	90.0	84.7
Sierra Leone	SLE	HepB vaccine	90.0	73.3
Sierra Leone	SLE	Pneumococcal vaccine	90.0	84.7
Sierra Leone	SLE	Rotavirus vaccine	92.0	86.5
Sierra Leone	SLE	Measles vaccine	80.0	65.2
Sierra Leone	SLE	Global wasting (<-2 SD) rate	6.9	7.6
Sierra Leone	SLE	Contraceptive prevalence (CPR)	23.85	21.5
Solomon Islands	SLB	Safe abortion services	85.0	76.7
Solomon Islands	SLB	TT - Tetanus toxoid vaccination	85.0	69.2
Solomon Islands	SLB	IPTp - Intermittent preventive treatment of malaria during pregnancy	1.2	1.0
Solomon Islands	SLB	Syphilis detection and treatment	19.8	16.1
Solomon Islands	SLB	Hypertensive disorder case management	15.5	12.6
Solomon Islands	SLB	Diabetes case management	12.1	9.9
Solomon Islands	SLB	Malaria case management	50.1	40.8
Solomon Islands	SLB	MgSO4 management of pre-eclampsia	30.7	25.0
Solomon Islands	SLB	Thermal protection	83.5	71.6
Solomon Islands	SLB	Clean cord care	80.6	69.1
Solomon Islands	SLB	Clean birth environment	69.3	59.4
Solomon Islands	SLB	Immediate drying and additional stimulation	77.4	66.4
Solomon Islands	SLB	Neonatal resuscitation	46.5	39.9
Solomon Islands	SLB	Antibiotics for preterm or prolonged PROM	63.2	54.2
Solomon Islands	SLB	Parenteral administration of anti-convulsants	60.4	51.8
Solomon Islands	SLB	Parenteral administration of uterotonics	75.5	64.7
Solomon Islands	SLB	Parenteral administration of antibiotics	63.2	54.2
Solomon Islands	SLB	Assisted vaginal delivery	21.4	18.3
Solomon Islands	SLB	Manual removal of placenta	31.6	27.1
Solomon Islands	SLB	Removal of retained products of conception	28.0	24.0
Solomon Islands	SLB	Cesarean delivery	7.4	6.3
Solomon Islands	SLB	Blood transfusion	10.6	9.1
Solomon Islands	SLB	Induction of labor for pregnancies lasting 41+ weeks	1.5	1.3

Solomon Islands	SLB	Complementary feeding - education only	36.9	31.6
Solomon Islands	SLB	Complementary feeding - supplementary feeding and education	36.9	31.6
Solomon Islands	SLB	Vitamin A supplementation	7.4	6.3
Solomon Islands	SLB	Improved sanitation - Utilization of latrines or toilets	33.5	33.5
Solomon Islands	SLB	Improved water source	67.8	67.8
Solomon Islands	SLB	Water connection in the home	31.3	31.3
Solomon Islands	SLB	Hygienic disposal of children's stools	29.4	29.4
Solomon Islands	SLB	ITN/IRS - Households protected from malaria	48.5	41.6
Solomon Islands	SLB	Injectable antibiotics for neonatal sepsis	84.5	68.8
Solomon Islands	SLB	ORS - oral rehydration solution	37.7	32.3
Solomon Islands	SLB	Antibiotics for treatment of dysentery	3.9	3.3
Solomon Islands	SLB	Vitamin A for treatment of measles	7.4	6.3
Solomon Islands	SLB	BCG vaccine	83.0	67.6
Solomon Islands	SLB	Polio vaccine	85.0	69.2
Solomon Islands	SLB	DPT vaccine	85.0	69.2
Solomon Islands	SLB	H. influenzae type b vaccine	85.0	80.0
Solomon Islands	SLB	HepB vaccine	85.0	69.2
Solomon Islands	SLB	Pneumococcal vaccine	84.0	79.0
Solomon Islands	SLB	Measles vaccine	84.0	68.4
Solomon Islands	SLB	Global wasting (<-2 SD) rate	10.6	11.7
Solomon Islands	SLB	Contraceptive prevalence (CPR)	33.8	30.5
Somalia	SOM	Safe abortion services	3.3	3.0
Somalia	SOM	TT - Tetanus toxoid vaccination	67.0	54.6
Somalia	SOM	IPTp - Intermittent preventive treatment of malaria during pregnancy	0.9	0.7
Somalia	SOM	Syphilis detection and treatment	6.5	5.3
Somalia	SOM	Hypertensive disorder case management	0.5	0.4
Somalia	SOM	Diabetes case management	0.4	0.3
Somalia	SOM	Malaria case management	1.5	1.2
Somalia	SOM	MgSO4 management of pre-eclampsia	0.9	0.7
Somalia	SOM	Thermal protection	9.3	8.0
Somalia	SOM	Clean cord care	8.9	7.6
Somalia	SOM	Clean birth environment	7.7	6.6
Somalia	SOM	Immediate drying and additional stimulation	8.6	7.4
Somalia	SOM	Neonatal resuscitation	5.1	4.4

Somalia	SOM	Antibiotics for preterm or prolonged PROM	7.0	6.0
Somalia	SOM	Parenteral administration of anti-convulsants	6.7	5.7
Somalia	SOM	Parenteral administration of uterotonics	8.4	7.2
Somalia	SOM	Parenteral administration of antibiotics	7.0	6.0
Somalia	SOM	Assisted vaginal delivery	2.4	2.1
Somalia	SOM	Manual removal of placenta	3.5	3.0
Somalia	SOM	Removal of retained products of conception	3.1	2.7
Somalia	SOM	Cesarean delivery	0.8	0.7
Somalia	SOM	Blood transfusion	1.2	1.0
Somalia	SOM	Induction of labor for pregnancies lasting 41+ weeks	0.2	0.2
Somalia	SOM	Complementary feeding - education only	11.0	9.4
Somalia	SOM	Complementary feeding - supplementary feeding and education	11.0	9.4
Somalia	SOM	Vitamin A supplementation	11.0	9.4
Somalia	SOM	Improved sanitation - Utilization of latrines or toilets	38.3	38.3
Somalia	SOM	Improved water source	52.4	52.4
Somalia	SOM	Water connection in the home	21.7	21.7
Somalia	SOM	Hygienic disposal of children's stools	34.7	34.7
Somalia	SOM	ITN/IRS - Households protected from malaria	12.2	10.5
Somalia	SOM	Injectable antibiotics for neonatal sepsis	9.4	7.7
Somalia	SOM	ORS - oral rehydration solution	13.2	11.3
Somalia	SOM	Oral antibiotics for pneumonia	13.0	11.1
Somalia	SOM	Vitamin A for treatment of measles	11.0	9.4
Somalia	SOM	ACTs- Artemisinin compounds for treatment of malaria	0.2	0.2
Somalia	SOM	SAM - treatment for severe acute malnutrition	20.1	17.2
Somalia	SOM	BCG vaccine	37.0	30.1
Somalia	SOM	Polio vaccine	47.0	38.3
Somalia	SOM	DPT vaccine	42.0	34.2
Somalia	SOM	H. influenzae type b vaccine	42.0	39.5
Somalia	SOM	HepB vaccine	42.0	34.2
Somalia	SOM	Measles vaccine	46.0	37.5
Somalia	SOM	Global wasting (<-2 SD) rate	13.3	14.6
Somalia	SOM	Contraceptive prevalence (CPR)	26.75	24.1
South Africa	ZAF	Safe abortion services	42.5	38.4

South Africa	ZAF	TT - Tetanus toxoid vaccination	90.0	73.3
South Africa	ZAF	Syphilis detection and treatment	23.2	18.9
South Africa	ZAF	Iron supplementation in pregnancy	50.5	41.1
South Africa	ZAF	Hypertensive disorder case management	18.4	15.0
South Africa	ZAF	Diabetes case management	14.4	11.7
South Africa	ZAF	Malaria case management	59.4	48.4
South Africa	ZAF	MgSO4 management of pre-eclampsia	36.4	29.6
South Africa	ZAF	Thermal protection	94.8	81.3
South Africa	ZAF	Clean cord care	91.6	78.5
South Africa	ZAF	Clean birth environment	78.7	67.5
South Africa	ZAF	Immediate drying and additional stimulation	87.8	75.3
South Africa	ZAF	Neonatal resuscitation	52.8	45.3
South Africa	ZAF	Antibiotics for preterm or prolonged PROM	71.8	61.6
South Africa	ZAF	Parenteral administration of anti-convulsants	68.6	58.8
South Africa	ZAF	Parenteral administration of uterotonics	85.7	73.5
South Africa	ZAF	Parenteral administration of antibiotics	71.8	61.6
South Africa	ZAF	Assisted vaginal delivery	24.3	20.8
South Africa	ZAF	Manual removal of placenta	35.9	30.8
South Africa	ZAF	Removal of retained products of conception	31.8	27.3
South Africa	ZAF	Cesarean delivery	8.4	7.2
South Africa	ZAF	Blood transfusion	12.1	10.4
South Africa	ZAF	Induction of labor for pregnancies lasting 41+ weeks	1.7	1.5
South Africa	ZAF	Complementary feeding - education only	49.3	42.3
South Africa	ZAF	Complementary feeding - supplementary feeding and education	49.3	42.3
South Africa	ZAF	Vitamin A supplementation	47.0	40.3
South Africa	ZAF	Improved sanitation - Utilization of latrines or toilets	75.7	75.7
South Africa	ZAF	Improved water source	92.7	92.7
South Africa	ZAF	Water connection in the home	83.4	83.4
South Africa	ZAF	Injectable antibiotics for neonatal sepsis	95.9	78.1
South Africa	ZAF	ORS - oral rehydration solution	51.4	44.1
South Africa	ZAF	Antibiotics for treatment of dysentery	8.0	6.9
South Africa	ZAF	Zinc for treatment of diarrhea	36.8	31.6
South Africa	ZAF	Oral antibiotics for pneumonia	65.7	56.3
South Africa	ZAF	Vitamin A for treatment of measles	47.0	40.3

South Africa	ZAF	BCG vaccine	70.0	57.0
South Africa	ZAF	Polio vaccine	74.0	60.3
South Africa	ZAF	DPT vaccine	74.0	60.3
South Africa	ZAF	H. influenzae type b vaccine	74.0	69.6
South Africa	ZAF	HepB vaccine	74.0	60.3
South Africa	ZAF	Pneumococcal vaccine	73.0	68.7
South Africa	ZAF	Rotavirus vaccine	70.0	65.8
South Africa	ZAF	Measles vaccine	60.0	48.9
South Africa	ZAF	Global wasting (<-2 SD) rate	2.6	2.8
South Africa	ZAF	Contraceptive prevalence (CPR)	56.95	51.4
South Sudan	SSD	Safe abortion services	2.2	2.0
South Sudan	SSD	TT - Tetanus toxoid vaccination	68.0	55.4
South Sudan	SSD	IPTp - Intermittent preventive treatment of malaria during pregnancy	10.2	8.3
South Sudan	SSD	Syphilis detection and treatment	10.0	8.1
South Sudan	SSD	Hypertensive disorder case management	4.2	3.4
South Sudan	SSD	Diabetes case management	3.2	2.6
South Sudan	SSD	Malaria case management	13.4	10.9
South Sudan	SSD	MgSO4 management of pre-eclampsia	8.2	6.7
South Sudan	SSD	Thermal protection	11.5	9.9
South Sudan	SSD	Clean cord care	11.1	9.5
South Sudan	SSD	Clean birth environment	9.6	8.2
South Sudan	SSD	Immediate drying and additional stimulation	10.7	9.2
South Sudan	SSD	Neonatal resuscitation	6.4	5.5
South Sudan	SSD	Antibiotics for preterm or prolonged PROM	8.7	7.5
South Sudan	SSD	Parenteral administration of anti-convulsants	8.3	7.1
South Sudan	SSD	Parenteral administration of uterotonics	10.4	8.9
South Sudan	SSD	Parenteral administration of antibiotics	8.7	7.5
South Sudan	SSD	Assisted vaginal delivery	2.9	2.5
South Sudan	SSD	Manual removal of placenta	4.4	3.8
South Sudan	SSD	Removal of retained products of conception	3.9	3.3
South Sudan	SSD	Cesarean delivery	1.0	0.9
South Sudan	SSD	Blood transfusion	1.5	1.3
South Sudan	SSD	Induction of labor for pregnancies lasting 41+ weeks	0.2	0.2
South Sudan	SSD	Complementary feeding - education only	49.4	42.4

South Sudan	SSD	Complementary feeding - supplementary feeding and education	49.4	42.4
South Sudan	SSD	Vitamin A supplementation	51.0	43.7
South Sudan	SSD	Improved sanitation - Utilization of latrines or toilets	11.3	11.3
South Sudan	SSD	Improved water source	40.7	40.7
South Sudan	SSD	Water connection in the home	1.3	1.3
South Sudan	SSD	Hygienic disposal of children's stools	15.7	15.7
South Sudan	SSD	ITN/IRS - Households protected from malaria	38.9	33.4
South Sudan	SSD	Injectable antibiotics for neonatal sepsis	11.7	9.5
South Sudan	SSD	ORS - oral rehydration solution	38.6	33.1
South Sudan	SSD	Zinc for treatment of diarrhea	3.1	2.7
South Sudan	SSD	Oral antibiotics for pneumonia	47.6	40.8
South Sudan	SSD	Vitamin A for treatment of measles	51.0	43.7
South Sudan	SSD	ACTs- Artemisinin compounds for treatment of malaria	2.8	2.4
South Sudan	SSD	SAM - treatment for severe acute malnutrition	46.1	39.5
South Sudan	SSD	BCG vaccine	47.0	38.3
South Sudan	SSD	Polio vaccine	50.0	40.7
South Sudan	SSD	DPT vaccine	49.0	39.9
South Sudan	SSD	H. influenzae type b vaccine	49.0	46.1
South Sudan	SSD	HepB vaccine	49.0	39.9
South Sudan	SSD	Measles vaccine	20.0	16.3
South Sudan	SSD	Global wasting (<-2 SD) rate	22.3	24.5
South Sudan	SSD	Contraceptive prevalence (CPR)	6.7	6.0
Sri Lanka	LKA	Safe abortion services	35.1	31.7
Sri Lanka	LKA	TT - Tetanus toxoid vaccination	99.0	80.6
Sri Lanka	LKA	Syphilis detection and treatment	24.4	19.9
Sri Lanka	LKA	Hypertensive disorder case management	22.2	18.1
Sri Lanka	LKA	Diabetes case management	17.3	14.1
Sri Lanka	LKA	Malaria case management	71.7	58.4
Sri Lanka	LKA	MgSO4 management of pre-eclampsia	44.0	35.8
Sri Lanka	LKA	Thermal protection	98.3	84.3
Sri Lanka	LKA	Clean cord care	94.9	81.4
Sri Lanka	LKA	Clean birth environment	81.6	70.0
Sri Lanka	LKA	Immediate drying and additional stimulation	91.1	78.1
Sri Lanka	LKA	Neonatal resuscitation	54.7	46.9

Sri Lanka	LKA	Antibiotics for preterm or prolonged PROM	74.5	63.9
Sri Lanka	LKA	Parenteral administration of anti-convulsants	71.1	61.0
Sri Lanka	LKA	Parenteral administration of uterotonics	88.9	76.2
Sri Lanka	LKA	Parenteral administration of antibiotics	74.5	63.9
Sri Lanka	LKA	Assisted vaginal delivery	25.2	21.6
Sri Lanka	LKA	Manual removal of placenta	37.2	31.9
Sri Lanka	LKA	Removal of retained products of conception	33.0	28.3
Sri Lanka	LKA	Cesarean delivery	8.7	7.5
Sri Lanka	LKA	Blood transfusion	12.5	10.7
Sri Lanka	LKA	Induction of labor for pregnancies lasting 41+ weeks	1.8	1.5
Sri Lanka	LKA	Complementary feeding - education only	72.6	62.2
Sri Lanka	LKA	Complementary feeding - supplementary feeding and education	72.6	62.2
Sri Lanka	LKA	Vitamin A supplementation	93.0	79.7
Sri Lanka	LKA	Improved sanitation - Utilization of latrines or toilets	95.8	95.8
Sri Lanka	LKA	Improved water source	89.4	89.4
Sri Lanka	LKA	Water connection in the home	34.2	34.2
Sri Lanka	LKA	Hygienic disposal of children's stools	90.6	90.6
Sri Lanka	LKA	ITN/IRS - Households protected from malaria	6.4	5.5
Sri Lanka	LKA	Injectable antibiotics for neonatal sepsis	99.5	81.0
Sri Lanka	LKA	ORS - oral rehydration solution	54.0	46.3
Sri Lanka	LKA	Oral antibiotics for pneumonia	52.3	44.8
Sri Lanka	LKA	Vitamin A for treatment of measles	93.0	79.7
Sri Lanka	LKA	BCG vaccine	99.0	80.6
Sri Lanka	LKA	Polio vaccine	99.0	80.6
Sri Lanka	LKA	DPT vaccine	99.0	80.6
Sri Lanka	LKA	H. influenzae type b vaccine	99.0	93.1
Sri Lanka	LKA	HepB vaccine	99.0	80.6
Sri Lanka	LKA	Measles vaccine	99.0	80.6
Sri Lanka	LKA	Global wasting (<-2 SD) rate	10.6	11.7
Sri Lanka	LKA	Contraceptive prevalence (CPR)	67.55	61.0
Sudan	SDN	Safe abortion services	2.2	2.0
Sudan	SDN	TT - Tetanus toxoid vaccination	80.0	65.2
Sudan	SDN	IPTp - Intermittent preventive treatment of malaria during pregnancy	1.9	1.5

Sudan	SDN	Syphilis detection and treatment	19.6	16.0
Sudan	SDN	Hypertensive disorder case management	12.2	9.9
Sudan	SDN	Diabetes case management	9.5	7.7
Sudan	SDN	Malaria case management	39.3	32.0
Sudan	SDN	MgSO4 management of pre-eclampsia	24.1	19.6
Sudan	SDN	Thermal protection	27.4	23.5
Sudan	SDN	Clean cord care	26.5	22.7
Sudan	SDN	Clean birth environment	22.7	19.5
Sudan	SDN	Immediate drying and additional stimulation	25.4	21.8
Sudan	SDN	Neonatal resuscitation	15.2	13.0
Sudan	SDN	Antibiotics for preterm or prolonged PROM	20.7	17.7
Sudan	SDN	Parenteral administration of anti-convulsants	19.8	17.0
Sudan	SDN	Parenteral administration of uterotonics	24.8	21.3
Sudan	SDN	Parenteral administration of antibiotics	20.7	17.7
Sudan	SDN	Assisted vaginal delivery	7.0	6.0
Sudan	SDN	Manual removal of placenta	10.4	8.9
Sudan	SDN	Removal of retained products of conception	9.2	7.9
Sudan	SDN	Cesarean delivery	2.4	2.1
Sudan	SDN	Blood transfusion	3.5	3.0
Sudan	SDN	Induction of labor for pregnancies lasting 41+ weeks	0.5	0.4
Sudan	SDN	Complementary feeding - education only	28.0	24.0
Sudan	SDN	Complementary feeding - supplementary feeding and education	28.0	24.0
Sudan	SDN	Vitamin A supplementation	20.0	17.1
Sudan	SDN	Improved sanitation - Utilization of latrines or toilets	36.6	36.6
Sudan	SDN	Improved water source	60.3	60.3
Sudan	SDN	Water connection in the home	27.6	27.6
Sudan	SDN	Hand washing with soap	25.9	25.9
Sudan	SDN	Hygienic disposal of children's stools	53.0	53.0
Sudan	SDN	ITN/IRS - Households protected from malaria	41.4	35.5
Sudan	SDN	Injectable antibiotics for neonatal sepsis	27.7	22.6
Sudan	SDN	ORS - oral rehydration solution	19.6	16.8
Sudan	SDN	Antibiotics for treatment of dysentery	40.8	35.0
Sudan	SDN	Zinc for treatment of diarrhea	15.2	13.0
Sudan	SDN	Oral antibiotics for pneumonia	48.3	41.4

Sudan	SDN	Vitamin A for treatment of measles	20.0	17.1
Sudan	SDN	ACTs- Artemisinin compounds for treatment of malaria	14.6	12.5
Sudan	SDN	BCG vaccine	88.0	71.7
Sudan	SDN	Polio vaccine	93.0	75.7
Sudan	SDN	DPT vaccine	93.0	75.7
Sudan	SDN	H. influenzae type b vaccine	93.0	87.5
Sudan	SDN	HepB vaccine	93.0	75.7
Sudan	SDN	Pneumococcal vaccine	93.0	87.5
Sudan	SDN	Rotavirus vaccine	94.0	88.4
Sudan	SDN	Meningococcal A	84.0	68.4
Sudan	SDN	Measles vaccine	90.0	73.3
Sudan	SDN	Global wasting (<-2 SD) rate	16.2	17.8
Sudan	SDN	Contraceptive prevalence (CPR)	15.5	14.0
Suriname	SUR	Safe abortion services	0.2	0.2
Suriname	SUR	TT - Tetanus toxoid vaccination	93.0	75.7
Suriname	SUR	Syphilis detection and treatment	21.0	17.1
Suriname	SUR	Hypertensive disorder case management	16.2	13.2
Suriname	SUR	Diabetes case management	12.6	10.3
Suriname	SUR	Malaria case management	52.3	42.6
Suriname	SUR	MgSO4 management of pre-eclampsia	32.1	26.1
Suriname	SUR	Thermal protection	91.8	78.7
Suriname	SUR	Clean cord care	88.6	76.0
Suriname	SUR	Clean birth environment	76.2	65.3
Suriname	SUR	Immediate drying and additional stimulation	85.1	73.0
Suriname	SUR	Neonatal resuscitation	51.1	43.8
Suriname	SUR	Antibiotics for preterm or prolonged PROM	69.5	59.6
Suriname	SUR	Parenteral administration of anti-convulsants	66.4	56.9
Suriname	SUR	Parenteral administration of uterotonics	83.0	71.2
Suriname	SUR	Parenteral administration of antibiotics	69.5	59.6
Suriname	SUR	Assisted vaginal delivery	23.5	20.1
Suriname	SUR	Manual removal of placenta	34.7	29.8
Suriname	SUR	Removal of retained products of conception	30.8	26.4
Suriname	SUR	Cesarean delivery	8.1	6.9
Suriname	SUR	Blood transfusion	11.7	10.0

Suriname	SUR	Induction of labor for pregnancies lasting 41+ weeks	1.6	1.4
Suriname	SUR	Improved sanitation - Utilization of latrines or toilets	84.5	84.5
Suriname	SUR	Improved water source	95.4	95.4
Suriname	SUR	Water connection in the home	75.1	75.1
Suriname	SUR	Hand washing with soap	75.0	75.0
Suriname	SUR	Hygienic disposal of children's stools	22.0	22.0
Suriname	SUR	ITN/IRS - Households protected from malaria	0.4	0.3
Suriname	SUR	Injectable antibiotics for neonatal sepsis	92.9	75.7
Suriname	SUR	ORS - oral rehydration solution	45.6	39.1
Suriname	SUR	Oral antibiotics for pneumonia	89.1	76.4
Suriname	SUR	Polio vaccine	95.0	77.4
Suriname	SUR	DPT vaccine	95.0	77.4
Suriname	SUR	H. influenzae type b vaccine	95.0	89.4
Suriname	SUR	HepB vaccine	95.0	77.4
Suriname	SUR	Measles vaccine	97.0	79.0
Suriname	SUR	Global wasting (<-2 SD) rate	5.0	5.5
Suriname	SUR	Contraceptive prevalence (CPR)	48.05	43.4
Swaziland	SWZ	Safe abortion services	42.5	38.4
Swaziland	SWZ	TT - Tetanus toxoid vaccination	88.0	71.7
Swaziland	SWZ	IPTp - Intermittent preventive treatment of malaria during pregnancy	0.1	0.1
Swaziland	SWZ	Syphilis detection and treatment	24.4	19.9
Swaziland	SWZ	Iron supplementation in pregnancy	33.6	27.4
Swaziland	SWZ	Hypertensive disorder case management	18.3	14.9
Swaziland	SWZ	Diabetes case management	14.3	11.6
Swaziland	SWZ	Malaria case management	59.0	48.1
Swaziland	SWZ	MgSO4 management of pre-eclampsia	36.2	29.5
Swaziland	SWZ	Thermal protection	86.7	74.3
Swaziland	SWZ	Clean cord care	83.7	71.8
Swaziland	SWZ	Clean birth environment	71.9	61.6
Swaziland	SWZ	Immediate drying and additional stimulation	80.3	68.8
Swaziland	SWZ	Neonatal resuscitation	48.2	41.3
Swaziland	SWZ	Antibiotics for preterm or prolonged PROM	65.6	56.2
Swaziland	SWZ	Parenteral administration of anti-convulsants	62.7	53.8
Swaziland	SWZ	Parenteral administration of uterotonics	78.4	67.2

Swaziland	SWZ	Parenteral administration of antibiotics	65.6	56.2
Swaziland	SWZ	Assisted vaginal delivery	22.2	19.0
Swaziland	SWZ	Manual removal of placenta	32.8	28.1
Swaziland	SWZ	Removal of retained products of conception	29.1	24.9
Swaziland	SWZ	Cesarean delivery	7.6	6.5
Swaziland	SWZ	Blood transfusion	11.0	9.4
Swaziland	SWZ	Induction of labor for pregnancies lasting 41+ weeks	1.5	1.3
Swaziland	SWZ	Complementary feeding - education only	62.4	53.5
Swaziland	SWZ	Complementary feeding - supplementary feeding and education	62.4	53.5
Swaziland	SWZ	Vitamin A supplementation	33.0	28.3
Swaziland	SWZ	Improved sanitation - Utilization of latrines or toilets	58.4	58.4
Swaziland	SWZ	Improved water source	69.0	69.0
Swaziland	SWZ	Water connection in the home	42.1	42.1
Swaziland	SWZ	Hand washing with soap	26.7	26.7
Swaziland	SWZ	Hygienic disposal of children's stools	55.3	55.3
Swaziland	SWZ	ITN/IRS - Households protected from malaria	16.2	13.9
Swaziland	SWZ	Injectable antibiotics for neonatal sepsis	87.7	71.4
Swaziland	SWZ	ORS - oral rehydration solution	84.0	72.0
Swaziland	SWZ	Antibiotics for treatment of dysentery	22.3	19.1
Swaziland	SWZ	Zinc for treatment of diarrhea	45.2	38.8
Swaziland	SWZ	Oral antibiotics for pneumonia	59.6	51.1
Swaziland	SWZ	Vitamin A for treatment of measles	33.0	28.3
Swaziland	SWZ	ACTs- Artemisinin compounds for treatment of malaria	0.1	0.1
Swaziland	SWZ	BCG vaccine	98.0	79.8
Swaziland	SWZ	Polio vaccine	90.0	73.3
Swaziland	SWZ	DPT vaccine	90.0	73.3
Swaziland	SWZ	H. influenzae type b vaccine	90.0	84.7
Swaziland	SWZ	HepB vaccine	90.0	73.3
Swaziland	SWZ	Pneumococcal vaccine	88.0	82.8
Swaziland	SWZ	Rotavirus vaccine	86.0	80.9
Swaziland	SWZ	Measles vaccine	89.0	72.5
Swaziland	SWZ	Global wasting (<-2 SD) rate	2.1	2.3
Swaziland	SWZ	Contraceptive prevalence (CPR)	67.9	61.3

Syrian Arab Republic	SYR	Safe abortion services	40.0	36.1
Syrian Arab Republic	SYR	TT - Tetanus toxoid vaccination	91.0	74.1
Syrian Arab Republic	SYR	Syphilis detection and treatment	15.7	12.8
Syrian Arab Republic	SYR	Hypertensive disorder case management	15.3	12.5
Syrian Arab Republic	SYR	Diabetes case management	11.9	9.7
Syrian Arab Republic	SYR	Malaria case management	49.4	40.2
Syrian Arab Republic	SYR	MgSO4 management of pre-eclampsia	30.3	24.7
Syrian Arab Republic	SYR	Thermal protection	69.6	59.7
Syrian Arab Republic	SYR	Clean cord care	67.2	57.6
Syrian Arab Republic	SYR	Clean birth environment	57.7	49.5
Syrian Arab Republic	SYR	Immediate drying and additional stimulation	64.5	55.3
Syrian Arab Republic	SYR	Neonatal resuscitation	38.7	33.2
Syrian Arab Republic	SYR	Antibiotics for preterm or prolonged PROM	52.7	45.2
Syrian Arab Republic	SYR	Parenteral administration of anti-convulsants	50.3	43.1
Syrian Arab Republic	SYR	Parenteral administration of uterotonics	62.9	53.9
Syrian Arab Republic	SYR	Parenteral administration of antibiotics	52.7	45.2
Syrian Arab Republic	SYR	Assisted vaginal delivery	17.8	15.3
Syrian Arab Republic	SYR	Manual removal of placenta	26.3	22.5
Syrian Arab Republic	SYR	Removal of retained products of conception	23.3	20.0
Syrian Arab Republic	SYR	Cesarean delivery	6.1	5.2
Syrian Arab Republic	SYR	Blood transfusion	8.9	7.6
Syrian Arab Republic	SYR	Induction of labor for pregnancies lasting 41+ weeks	1.2	1.0
Syrian Arab Republic	SYR	Improved sanitation - Utilization of latrines or toilets	91.2	91.2
Syrian Arab Republic	SYR	Improved water source	97.2	97.2
Syrian Arab Republic	SYR	Water connection in the home	70.9	70.9
Syrian Arab Republic	SYR	Injectable antibiotics for neonatal sepsis	70.4	57.3
Syrian Arab Republic	SYR	ORS - oral rehydration solution	49.8	42.7
Syrian Arab Republic	SYR	Oral antibiotics for pneumonia	76.8	65.8
Syrian Arab Republic	SYR	BCG vaccine	79.0	64.3
Syrian Arab Republic	SYR	Polio vaccine	53.0	43.2
Syrian Arab Republic	SYR	DPT vaccine	47.0	38.3
Syrian Arab Republic	SYR	H. influenzae type b vaccine	48.0	45.2
Syrian Arab Republic	SYR	HepB vaccine	47.0	38.3
Syrian Arab Republic	SYR	Measles vaccine	67.0	54.6

Syrian Arab Republic	SYR	Global wasting (<-2 SD) rate	10.3	11.3
Syrian Arab Republic	SYR	Contraceptive prevalence (CPR)	59.75	53.9
Tajikistan	TJK	Safe abortion services	35.1	31.7
Tajikistan	TJK	Syphilis detection and treatment	23.0	18.7
Tajikistan	TJK	Iron supplementation in pregnancy	2.2	1.8
Tajikistan	TJK	Hypertensive disorder case management	15.5	12.6
Tajikistan	TJK	Diabetes case management	12.1	9.9
Tajikistan	TJK	Malaria case management	50.1	40.8
Tajikistan	TJK	MgSO4 management of pre-eclampsia	30.7	25.0
Tajikistan	TJK	Thermal protection	87.1	74.7
Tajikistan	TJK	Clean cord care	84.1	72.1
Tajikistan	TJK	Clean birth environment	72.3	62.0
Tajikistan	TJK	Immediate drying and additional stimulation	80.7	69.2
Tajikistan	TJK	Neonatal resuscitation	48.5	41.6
Tajikistan	TJK	Antibiotics for preterm or prolonged PROM	66.0	56.6
Tajikistan	TJK	Parenteral administration of anti-convulsants	63.0	54.0
Tajikistan	TJK	Parenteral administration of uterotonics	78.8	67.6
Tajikistan	TJK	Parenteral administration of antibiotics	66.0	56.6
Tajikistan	TJK	Assisted vaginal delivery	22.3	19.1
Tajikistan	TJK	Manual removal of placenta	33.0	28.3
Tajikistan	TJK	Removal of retained products of conception	29.2	25.0
Tajikistan	TJK	Cesarean delivery	7.7	6.6
Tajikistan	TJK	Blood transfusion	11.1	9.5
Tajikistan	TJK	Induction of labor for pregnancies lasting 41+ weeks	1.6	1.4
Tajikistan	TJK	Complementary feeding - education only	29.1	24.9
Tajikistan	TJK	Complementary feeding - supplementary feeding and education	29.1	24.9
Tajikistan	TJK	Vitamin A supplementation	91.0	78.0
Tajikistan	TJK	Improved sanitation - Utilization of latrines or toilets	97.0	97.0
Tajikistan	TJK	Improved water source	81.2	81.2
Tajikistan	TJK	Water connection in the home	52.2	52.2
Tajikistan	TJK	Hygienic disposal of children's stools	52.0	52.0
Tajikistan	TJK	ITN/IRS - Households protected from malaria	2.0	1.7
Tajikistan	TJK	Injectable antibiotics for neonatal sepsis	88.2	71.8
Tajikistan	TJK	ORS - oral rehydration solution	61.7	52.9

Tajikistan	TJK	Antibiotics for treatment of dysentery	59.1	50.7
Tajikistan	TJK	Zinc for treatment of diarrhea	19.7	16.9
Tajikistan	TJK	Oral antibiotics for pneumonia	68.5	58.7
Tajikistan	TJK	Vitamin A for treatment of measles	91.0	78.0
Tajikistan	TJK	ACTs- Artemisinin compounds for treatment of malaria	0.1	0.1
Tajikistan	TJK	BCG vaccine	99.0	80.6
Tajikistan	TJK	Polio vaccine	96.0	78.2
Tajikistan	TJK	DPT vaccine	96.0	78.2
Tajikistan	TJK	H. influenzae type b vaccine	96.0	90.3
Tajikistan	TJK	HepB vaccine	96.0	78.2
Tajikistan	TJK	Rotavirus vaccine	96.0	90.3
Tajikistan	TJK	Measles vaccine	98.0	79.8
Tajikistan	TJK	Global wasting (<-2 SD) rate	5.5	6.0
Tajikistan	TJK	Contraceptive prevalence (CPR)	31.5	28.4
Thailand	THA	Safe abortion services	39.0	35.2
Thailand	THA	TT - Tetanus toxoid vaccination	98.0	79.8
Thailand	THA	Syphilis detection and treatment	24.3	19.8
Thailand	THA	Hypertensive disorder case management	21.8	17.8
Thailand	THA	Diabetes case management	17.0	13.8
Thailand	THA	Malaria case management	70.4	57.3
Thailand	THA	MgSO4 management of pre-eclampsia	43.2	35.2
Thailand	THA	Thermal protection	97.0	83.2
Thailand	THA	Clean cord care	93.6	80.3
Thailand	THA	Clean birth environment	80.5	69.0
Thailand	THA	Immediate drying and additional stimulation	89.8	77.0
Thailand	THA	Neonatal resuscitation	54.0	46.3
Thailand	THA	Antibiotics for preterm or prolonged PROM	73.4	62.9
Thailand	THA	Parenteral administration of anti-convulsants	70.2	60.2
Thailand	THA	Parenteral administration of uterotonics	87.7	75.2
Thailand	THA	Parenteral administration of antibiotics	73.4	62.9
Thailand	THA	Assisted vaginal delivery	24.8	21.3
Thailand	THA	Manual removal of placenta	36.7	31.5
Thailand	THA	Removal of retained products of conception	32.5	27.9
Thailand	THA	Cesarean delivery	8.5	7.3
Thailand	THA	Blood transfusion	12.4	10.6

Thailand	THA	Induction of labor for pregnancies lasting 41+ weeks	1.7	1.5
Thailand	THA	Complementary feeding - education only	75.0	64.3
Thailand	THA	Complementary feeding - supplementary feeding and education	75.0	64.3
Thailand	THA	Improved sanitation - Utilization of latrines or toilets	98.8	98.8
Thailand	THA	Improved water source	99.0	99.0
Thailand	THA	Water connection in the home	69.6	69.6
Thailand	THA	Hand washing with soap	81.2	81.2
Thailand	THA	Hygienic disposal of children's stools	42.0	42.0
Thailand	THA	Injectable antibiotics for neonatal sepsis	98.1	79.9
Thailand	THA	ORS - oral rehydration solution	72.5	62.2
Thailand	THA	Oral antibiotics for pneumonia	79.5	68.2
Thailand	THA	BCG vaccine	99.0	80.6
Thailand	THA	Polio vaccine	97.0	79.0
Thailand	THA	DPT vaccine	97.0	79.0
Thailand	THA	HepB vaccine	97.0	79.0
Thailand	THA	Measles vaccine	99.0	80.6
Thailand	THA	Global wasting (<-2 SD) rate	5.5	6.0
Thailand	THA	Contraceptive prevalence (CPR)	79.3	71.6
Timor-Leste	TLS	Safe abortion services	39.0	35.2
Timor-Leste	TLS	TT - Tetanus toxoid vaccination	83.0	67.6
Timor-Leste	TLS	Syphilis detection and treatment	20.9	17.0
Timor-Leste	TLS	Iron supplementation in pregnancy	12.9	10.5
Timor-Leste	TLS	Hypertensive disorder case management	18.5	15.1
Timor-Leste	TLS	Diabetes case management	14.4	11.7
Timor-Leste	TLS	Malaria case management	59.6	48.5
Timor-Leste	TLS	MgSO4 management of pre-eclampsia	36.5	29.7
Timor-Leste	TLS	Thermal protection	48.0	41.2
Timor-Leste	TLS	Clean cord care	46.3	39.7
Timor-Leste	TLS	Clean birth environment	39.8	34.1
Timor-Leste	TLS	Immediate drying and additional stimulation	44.4	38.1
Timor-Leste	TLS	Neonatal resuscitation	26.7	22.9
Timor-Leste	TLS	Antibiotics for preterm or prolonged PROM	36.3	31.1
Timor-Leste	TLS	Parenteral administration of anti-convulsants	34.7	29.8
Timor-Leste	TLS	Parenteral administration of uterotonics	43.4	37.2

Timor-Leste	TLS	Parenteral administration of antibiotics	36.3	31.1
Timor-Leste	TLS	Assisted vaginal delivery	12.3	10.5
Timor-Leste	TLS	Manual removal of placenta	18.1	15.5
Timor-Leste	TLS	Removal of retained products of conception	16.1	13.8
Timor-Leste	TLS	Cesarean delivery	4.2	3.6
Timor-Leste	TLS	Blood transfusion	6.1	5.2
Timor-Leste	TLS	Induction of labor for pregnancies lasting 41+ weeks	0.9	0.8
Timor-Leste	TLS	Complementary feeding - education only	33.6	28.8
Timor-Leste	TLS	Complementary feeding - supplementary feeding and education	33.6	28.8
Timor-Leste	TLS	Vitamin A supplementation	66.0	56.6
Timor-Leste	TLS	Improved sanitation - Utilization of latrines or toilets	53.5	53.5
Timor-Leste	TLS	Improved water source	78.3	78.3
Timor-Leste	TLS	Water connection in the home	50.6	50.6
Timor-Leste	TLS	Hygienic disposal of children's stools	25.5	25.5
Timor-Leste	TLS	ITN/IRS - Households protected from malaria	64.0	54.9
Timor-Leste	TLS	Injectable antibiotics for neonatal sepsis	48.5	39.5
Timor-Leste	TLS	ORS - oral rehydration solution	69.9	59.9
Timor-Leste	TLS	Antibiotics for treatment of dysentery	6.7	5.7
Timor-Leste	TLS	Zinc for treatment of diarrhea	50.2	43.0
Timor-Leste	TLS	Oral antibiotics for pneumonia	70.3	60.3
Timor-Leste	TLS	Vitamin A for treatment of measles	66.0	56.6
Timor-Leste	TLS	ACTs- Artemisinin compounds for treatment of malaria	1.1	0.9
Timor-Leste	TLS	SAM - treatment for severe acute malnutrition	5.6	4.8
Timor-Leste	TLS	BCG vaccine	95.0	77.4
Timor-Leste	TLS	Polio vaccine	83.0	67.6
Timor-Leste	TLS	DPT vaccine	83.0	67.6
Timor-Leste	TLS	H. influenzae type b vaccine	83.0	78.1
Timor-Leste	TLS	HepB vaccine	83.0	67.6
Timor-Leste	TLS	Measles vaccine	70.0	57.0
Timor-Leste	TLS	Global wasting (<-2 SD) rate	24.2	26.6
Timor-Leste	TLS	Contraceptive prevalence (CPR)	30.5	27.5
Togo	TGO	TT - Tetanus toxoid vaccination	83.0	67.6

Togo	TGO	IPTp - Intermittent preventive treatment of malaria during pregnancy	68.2	55.5
Togo	TGO	Syphilis detection and treatment	18.4	15.0
Togo	TGO	Iron supplementation in pregnancy	37.1	30.2
Togo	TGO	Hypertensive disorder case management	16.7	13.6
Togo	TGO	Diabetes case management	18.3	14.9
Togo	TGO	Malaria case management	49.1	40.0
Togo	TGO	MgSO4 management of pre-eclampsia	9.5	7.7
Togo	TGO	Thermal protection	60.7	52.0
Togo	TGO	Clean cord care	46.4	39.8
Togo	TGO	Clean birth environment	42.8	36.7
Togo	TGO	Immediate drying and additional stimulation	55.4	47.5
Togo	TGO	Neonatal resuscitation	12.8	11.0
Togo	TGO	Antibiotics for preterm or prolonged PROM	48.5	41.6
Togo	TGO	Parenteral administration of anti-convulsants	10.9	9.3
Togo	TGO	Parenteral administration of uterotonics	55.4	47.5
Togo	TGO	Parenteral administration of antibiotics	48.5	41.6
Togo	TGO	Assisted vaginal delivery	8.1	6.9
Togo	TGO	Manual removal of placenta	36.1	31.0
Togo	TGO	Removal of retained products of conception	16.9	14.5
Togo	TGO	Cesarean delivery	0.8	0.7
Togo	TGO	Blood transfusion	7.7	6.6
Togo	TGO	Induction of labor for pregnancies lasting 41+ weeks	1.1	0.9
Togo	TGO	Complementary feeding - education only	20.3	17.4
Togo	TGO	Complementary feeding - supplementary feeding and education	20.3	17.4
Togo	TGO	Vitamin A supplementation	87.0	74.6
Togo	TGO	Improved sanitation - Utilization of latrines or toilets	16.1	16.1
Togo	TGO	Improved water source	65.1	65.1
Togo	TGO	Water connection in the home	16.7	16.7
Togo	TGO	Hand washing with soap	62.0	62.0
Togo	TGO	Hygienic disposal of children's stools	34.8	34.8
Togo	TGO	ITN/IRS - Households protected from malaria	65.4	56.1
Togo	TGO	Injectable antibiotics for neonatal sepsis	61.4	50.0
Togo	TGO	ORS - oral rehydration solution	18.5	15.9

Togo	TGO	Antibiotics for treatment of dysentery	29.7	25.5
Togo	TGO	Zinc for treatment of diarrhea	0.1	0.1
Togo	TGO	Oral antibiotics for pneumonia	48.5	41.6
Togo	TGO	Vitamin A for treatment of measles	87.0	74.6
Togo	TGO	ACTs- Artemisinin compounds for treatment of malaria	6.3	5.4
Togo	TGO	SAM - treatment for severe acute malnutrition	6.4	5.5
Togo	TGO	BCG vaccine	83.0	67.6
Togo	TGO	Polio vaccine	66.0	53.8
Togo	TGO	DPT vaccine	88.0	71.7
Togo	TGO	H. influenzae type b vaccine	88.0	82.8
Togo	TGO	HepB vaccine	88.0	71.7
Togo	TGO	Pneumococcal vaccine	88.0	82.8
Togo	TGO	Rotavirus vaccine	89.0	83.7
Togo	TGO	Measles vaccine	91.0	74.1
Togo	TGO	Global wasting (<-2 SD) rate	6.6	7.3
Togo	TGO	Contraceptive prevalence (CPR)	24.65	22.2
Tonga	TON	Safe abortion services	85.0	76.7
Tonga	TON	TT - Tetanus toxoid vaccination	71.2	58.0
Tonga	TON	Syphilis detection and treatment	19.3	15.7
Tonga	TON	Hypertensive disorder case management	16.9	13.8
Tonga	TON	Diabetes case management	13.2	10.8
Tonga	TON	Malaria case management	54.6	44.5
Tonga	TON	MgSO4 management of pre-eclampsia	33.5	27.3
Tonga	TON	Thermal protection	96.8	83.0
Tonga	TON	Clean cord care	93.4	80.1
Tonga	TON	Clean birth environment	80.3	68.8
Tonga	TON	Immediate drying and additional stimulation	89.6	76.8
Tonga	TON	Neonatal resuscitation	53.8	46.1
Tonga	TON	Antibiotics for preterm or prolonged PROM	73.3	62.8
Tonga	TON	Parenteral administration of anti-convulsants	70.0	60.0
Tonga	TON	Parenteral administration of uterotonics	87.5	75.0
Tonga	TON	Parenteral administration of antibiotics	73.3	62.8
Tonga	TON	Assisted vaginal delivery	24.7	21.2
Tonga	TON	Manual removal of placenta	36.6	31.4
Tonga	TON	Removal of retained products of conception	32.4	27.8

Tonga	TON	Cesarean delivery	8.5	7.3
Tonga	TON	Blood transfusion	12.3	10.5
Tonga	TON	Induction of labor for pregnancies lasting 41+ weeks	1.7	1.5
Tonga	TON	Improved sanitation - Utilization of latrines or toilets	93.4	93.4
Tonga	TON	Improved water source	99.0	99.0
Tonga	TON	Water connection in the home	78.2	78.2
Tonga	TON	Hygienic disposal of children's stools	40.4	40.4
Tonga	TON	Injectable antibiotics for neonatal sepsis	97.9	79.7
Tonga	TON	BCG vaccine	88.0	71.7
Tonga	TON	Polio vaccine	83.0	67.6
Tonga	TON	DPT vaccine	81.0	66.0
Tonga	TON	H. influenzae type b vaccine	81.0	76.2
Tonga	TON	HepB vaccine	81.0	66.0
Tonga	TON	Measles vaccine	85.0	69.2
Tonga	TON	Global wasting (<-2 SD) rate	10.6	11.7
Tonga	TON	Contraceptive prevalence (CPR)	37.55	33.9
Tunisia	TUN	Safe abortion services	2.2	2.0
Tunisia	TUN	TT - Tetanus toxoid vaccination	96.0	78.2
Tunisia	TUN	Syphilis detection and treatment	23.6	19.2
Tunisia	TUN	Hypertensive disorder case management	20.2	16.5
Tunisia	TUN	Diabetes case management	15.8	12.9
Tunisia	TUN	Malaria case management	65.2	53.1
Tunisia	TUN	MgSO4 management of pre-eclampsia	40.0	32.6
Tunisia	TUN	Thermal protection	98.5	84.5
Tunisia	TUN	Clean cord care	95.1	81.5
Tunisia	TUN	Clean birth environment	81.8	70.1
Tunisia	TUN	Immediate drying and additional stimulation	91.3	78.3
Tunisia	TUN	Neonatal resuscitation	54.8	47.0
Tunisia	TUN	Antibiotics for preterm or prolonged PROM	74.6	64.0
Tunisia	TUN	Parenteral administration of anti-convulsants	71.3	61.1
Tunisia	TUN	Parenteral administration of uterotonics	89.1	76.4
Tunisia	TUN	Parenteral administration of antibiotics	74.6	64.0
Tunisia	TUN	Assisted vaginal delivery	25.2	21.6
Tunisia	TUN	Manual removal of placenta	37.3	32.0

Tunisia	TUN	Removal of retained products of conception	33.0	28.3
Tunisia	TUN	Cesarean delivery	8.7	7.5
Tunisia	TUN	Blood transfusion	12.6	10.8
Tunisia	TUN	Induction of labor for pregnancies lasting 41+ weeks	1.8	1.5
Tunisia	TUN	Complementary feeding - education only	69.5	59.6
Tunisia	TUN	Complementary feeding - supplementary feeding and education	69.5	59.6
Tunisia	TUN	Improved sanitation - Utilization of latrines or toilets	90.9	90.9
Tunisia	TUN	Improved water source	96.3	96.3
Tunisia	TUN	Water connection in the home	88.9	88.9
Tunisia	TUN	Hand washing with soap	94.2	94.2
Tunisia	TUN	Hygienic disposal of children's stools	13.8	13.8
Tunisia	TUN	Injectable antibiotics for neonatal sepsis	99.7	81.2
Tunisia	TUN	ORS - oral rehydration solution	39.6	34.0
Tunisia	TUN	Zinc for treatment of diarrhea	3.9	3.3
Tunisia	TUN	Oral antibiotics for pneumonia	77.0	66.0
Tunisia	TUN	BCG vaccine	92.0	74.9
Tunisia	TUN	Polio vaccine	97.0	79.0
Tunisia	TUN	DPT vaccine	97.0	79.0
Tunisia	TUN	H. influenzae type b vaccine	97.0	91.2
Tunisia	TUN	HepB vaccine	97.0	79.0
Tunisia	TUN	Measles vaccine	98.0	79.8
Tunisia	TUN	Global wasting (<-2 SD) rate	2.8	3.0
Tunisia	TUN	Contraceptive prevalence (CPR)	59.25	53.5
Turkey	TUR	Safe abortion services	40.0	36.1
Turkey	TUR	TT - Tetanus toxoid vaccination	95.0	77.4
Turkey	TUR	Syphilis detection and treatment	23.9	19.5
Turkey	TUR	Hypertensive disorder case management	21.4	17.4
Turkey	TUR	Diabetes case management	16.7	13.6
Turkey	TUR	Malaria case management	68.9	56.1
Turkey	TUR	MgSO4 management of pre-eclampsia	42.3	34.5
Turkey	TUR	Thermal protection	96.1	82.4
Turkey	TUR	Clean cord care	92.8	79.6
Turkey	TUR	Clean birth environment	79.7	68.3
Turkey	TUR	Immediate drying and additional stimulation	89.0	76.3

Turkey	TUR	Neonatal resuscitation	53.4	45.8
Turkey	TUR	Antibiotics for preterm or prolonged PROM	72.7	62.3
Turkey	TUR	Parenteral administration of anti-convulsants	69.5	59.6
Turkey	TUR	Parenteral administration of uterotonics	86.9	74.5
Turkey	TUR	Parenteral administration of antibiotics	72.7	62.3
Turkey	TUR	Assisted vaginal delivery	24.6	21.1
Turkey	TUR	Manual removal of placenta	36.4	31.2
Turkey	TUR	Removal of retained products of conception	32.2	27.6
Turkey	TUR	Cesarean delivery	8.5	7.3
Turkey	TUR	Blood transfusion	12.2	10.5
Turkey	TUR	Induction of labor for pregnancies lasting 41+ weeks	1.7	1.5
Turkey	TUR	Improved sanitation - Utilization of latrines or toilets	97.3	97.3
Turkey	TUR	Improved water source	98.9	98.9
Turkey	TUR	Water connection in the home	97.8	97.8
Turkey	TUR	Injectable antibiotics for neonatal sepsis	97.2	79.2
Turkey	TUR	ORS - oral rehydration solution	20.4	17.5
Turkey	TUR	Antibiotics for treatment of dysentery	7.5	6.4
Turkey	TUR	BCG vaccine	96.0	78.2
Turkey	TUR	Polio vaccine	98.0	79.8
Turkey	TUR	DPT vaccine	98.0	79.8
Turkey	TUR	H. influenzae type b vaccine	98.0	92.2
Turkey	TUR	HepB vaccine	98.0	79.8
Turkey	TUR	Pneumococcal vaccine	97.0	91.2
Turkey	TUR	Measles vaccine	96.0	78.2
Turkey	TUR	Global wasting (<-2 SD) rate	1.1	1.2
Turkey	TUR	Contraceptive prevalence (CPR)	74.45	67.2
Turkmenistan	TKM	Safe abortion services	35.1	31.7
Turkmenistan	TKM	Syphilis detection and treatment	24.7	20.1
Turkmenistan	TKM	Hypertensive disorder case management	23.2	18.9
Turkmenistan	TKM	Diabetes case management	18.1	14.7
Turkmenistan	TKM	Malaria case management	74.7	60.8
Turkmenistan	TKM	MgSO4 management of pre-eclampsia	45.8	37.3
Turkmenistan	TKM	Thermal protection	98.3	84.3
Turkmenistan	TKM	Clean cord care	94.9	81.4

Turkmenistan	TKM	Clean birth environment	81.6	70.0
Turkmenistan	TKM	Immediate drying and additional stimulation	91.1	78.1
Turkmenistan	TKM	Neonatal resuscitation	54.7	46.9
Turkmenistan	TKM	Antibiotics for preterm or prolonged PROM	74.4	63.8
Turkmenistan	TKM	Parenteral administration of anti-convulsants	71.1	61.0
Turkmenistan	TKM	Parenteral administration of uterotonics	88.9	76.2
Turkmenistan	TKM	Parenteral administration of antibiotics	74.4	63.8
Turkmenistan	TKM	Assisted vaginal delivery	25.1	21.5
Turkmenistan	TKM	Manual removal of placenta	37.2	31.9
Turkmenistan	TKM	Removal of retained products of conception	33.0	28.3
Turkmenistan	TKM	Cesarean delivery	8.7	7.5
Turkmenistan	TKM	Blood transfusion	12.5	10.7
Turkmenistan	TKM	Induction of labor for pregnancies lasting 41+ weeks	1.8	1.5
Turkmenistan	TKM	Complementary feeding - education only	85.2	73.0
Turkmenistan	TKM	Complementary feeding - supplementary feeding and education	85.2	73.0
Turkmenistan	TKM	Improved sanitation - Utilization of latrines or toilets	98.7	98.7
Turkmenistan	TKM	Improved water source	98.8	98.8
Turkmenistan	TKM	Water connection in the home	56.3	56.3
Turkmenistan	TKM	Hand washing with soap	99.3	99.3
Turkmenistan	TKM	Hygienic disposal of children's stools	60.5	60.5
Turkmenistan	TKM	Injectable antibiotics for neonatal sepsis	99.5	81.0
Turkmenistan	TKM	ORS - oral rehydration solution	47.1	40.4
Turkmenistan	TKM	Zinc for treatment of diarrhea	10.9	9.3
Turkmenistan	TKM	Oral antibiotics for pneumonia	82.7	70.9
Turkmenistan	TKM	BCG vaccine	98.0	79.8
Turkmenistan	TKM	Polio vaccine	99.0	80.6
Turkmenistan	TKM	DPT vaccine	99.0	80.6
Turkmenistan	TKM	H. influenzae type b vaccine	99.0	93.1
Turkmenistan	TKM	HepB vaccine	99.0	80.6
Turkmenistan	TKM	Measles vaccine	99.0	80.6
Turkmenistan	TKM	Global wasting (<-2 SD) rate	4.3	4.7
Turkmenistan	TKM	Contraceptive prevalence (CPR)	53.65	48.4
Uganda	UGA	Safe abortion services	3.3	3.0
Uganda	UGA	TT - Tetanus toxoid vaccination	85.0	69.2

Uganda	UGA	IPTp - Intermittent preventive treatment of malaria during pregnancy	45.9	37.4
Uganda	UGA	Syphilis detection and treatment	28.1	22.9
Uganda	UGA	Iron supplementation in pregnancy	22.6	18.4
Uganda	UGA	Hypertensive disorder case management	17.7	14.4
Uganda	UGA	Diabetes case management	10.1	8.2
Uganda	UGA	Malaria case management	40.6	33.1
Uganda	UGA	MgSO4 management of pre-eclampsia	23.1	18.8
Uganda	UGA	Thermal protection	72.5	62.2
Uganda	UGA	Clean cord care	63.6	54.5
Uganda	UGA	Clean birth environment	57.7	49.5
Uganda	UGA	Immediate drying and additional stimulation	56.8	48.7
Uganda	UGA	Neonatal resuscitation	36.2	31.0
Uganda	UGA	Antibiotics for preterm or prolonged PROM	50.0	42.9
Uganda	UGA	Parenteral administration of anti-convulsants	40.9	35.1
Uganda	UGA	Parenteral administration of uterotonics	65.6	56.2
Uganda	UGA	Parenteral administration of antibiotics	50.0	42.9
Uganda	UGA	Assisted vaginal delivery	25.6	21.9
Uganda	UGA	Manual removal of placenta	24.2	20.7
Uganda	UGA	Removal of retained products of conception	28.4	24.3
Uganda	UGA	Cesarean delivery	2.3	2.0
Uganda	UGA	Blood transfusion	25.3	21.7
Uganda	UGA	Induction of labor for pregnancies lasting 41+ weeks	11.5	9.9
Uganda	UGA	Complementary feeding - education only	30.2	25.9
Uganda	UGA	Complementary feeding - supplementary feeding and education	30.2	25.9
Uganda	UGA	Vitamin A supplementation	27.0	23.1
Uganda	UGA	Improved sanitation - Utilization of latrines or toilets	18.5	18.5
Uganda	UGA	Improved water source	49.1	49.1
Uganda	UGA	Water connection in the home	10.4	10.4
Uganda	UGA	Hand washing with soap	27.4	27.4
Uganda	UGA	Hygienic disposal of children's stools	76.3	76.3
Uganda	UGA	ITN/IRS - Households protected from malaria	80.8	69.3
Uganda	UGA	Injectable antibiotics for neonatal sepsis	73.4	59.8
Uganda	UGA	ORS - oral rehydration solution	46.7	40.0

Uganda	UGA	Antibiotics for treatment of dysentery	32.1	27.5
Uganda	UGA	Zinc for treatment of diarrhea	40.3	34.6
Uganda	UGA	Oral antibiotics for pneumonia	71.3	61.1
Uganda	UGA	Vitamin A for treatment of measles	27.0	23.1
Uganda	UGA	ACTs- Artemisinin compounds for treatment of malaria	45.9	39.4
Uganda	UGA	SAM - treatment for severe acute malnutrition	2.8	2.4
Uganda	UGA	BCG vaccine	88.0	71.7
Uganda	UGA	Polio vaccine	88.0	71.7
Uganda	UGA	DPT vaccine	93.0	75.7
Uganda	UGA	H. influenzae type b vaccine	93.0	87.5
Uganda	UGA	HepB vaccine	93.0	75.7
Uganda	UGA	Pneumococcal vaccine	92.0	86.5
Uganda	UGA	Rotavirus vaccine	36.0	33.9
Uganda	UGA	Measles vaccine	80.0	65.2
Uganda	UGA	Global wasting (<-2 SD) rate	3.6	3.9
Uganda	UGA	Contraceptive prevalence (CPR)	43.55	39.3
Ukraine	UKR	Safe abortion services	87.5	79.0
Ukraine	UKR	IPTp - Intermittent preventive treatment of malaria during pregnancy	41.1	33.5
Ukraine	UKR	Syphilis detection and treatment	24.4	19.9
Ukraine	UKR	Iron supplementation in pregnancy	3.4	2.8
Ukraine	UKR	Hypertensive disorder case management	21.0	17.1
Ukraine	UKR	Diabetes case management	16.3	13.3
Ukraine	UKR	Malaria case management	67.6	55.1
Ukraine	UKR	MgSO4 management of pre-eclampsia	41.5	33.8
Ukraine	UKR	Thermal protection	97.7	83.8
Ukraine	UKR	Clean cord care	94.4	80.9
Ukraine	UKR	Clean birth environment	81.1	69.5
Ukraine	UKR	Immediate drying and additional stimulation	90.5	77.6
Ukraine	UKR	Neonatal resuscitation	54.4	46.6
Ukraine	UKR	Antibiotics for preterm or prolonged PROM	74.0	63.4
Ukraine	UKR	Parenteral administration of anti-convulsants	70.7	60.6
Ukraine	UKR	Parenteral administration of uterotonics	88.4	75.8
Ukraine	UKR	Parenteral administration of antibiotics	74.0	63.4
Ukraine	UKR	Assisted vaginal delivery	25.0	21.4

Ukraine	UKR	Manual removal of placenta	37.0	31.7
Ukraine	UKR	Removal of retained products of conception	32.8	28.1
Ukraine	UKR	Cesarean delivery	8.6	7.4
Ukraine	UKR	Blood transfusion	12.5	10.7
Ukraine	UKR	Induction of labor for pregnancies lasting 41+ weeks	1.7	1.5
Ukraine	UKR	Complementary feeding - education only	58.5	50.2
Ukraine	UKR	Complementary feeding - supplementary feeding and education	58.5	50.2
Ukraine	UKR	Improved sanitation - Utilization of latrines or toilets	96.2	96.2
Ukraine	UKR	Improved water source	93.8	93.8
Ukraine	UKR	Water connection in the home	62.0	62.0
Ukraine	UKR	Injectable antibiotics for neonatal sepsis	98.9	80.6
Ukraine	UKR	ORS - oral rehydration solution	59.2	50.8
Ukraine	UKR	Oral antibiotics for pneumonia	92.3	79.1
Ukraine	UKR	BCG vaccine	90.0	73.3
Ukraine	UKR	Polio vaccine	48.0	39.1
Ukraine	UKR	DPT vaccine	50.0	40.7
Ukraine	UKR	H. influenzae type b vaccine	39.0	36.7
Ukraine	UKR	HepB vaccine	52.0	42.4
Ukraine	UKR	Measles vaccine	86.0	70.0
Ukraine	UKR	Global wasting (<-2 SD) rate	4.5	5.0
Ukraine	UKR	Contraceptive prevalence (CPR)	67.4	60.8
United Republic of Tanzania	TZA	Safe abortion services	3.3	3.0
United Republic of Tanzania	TZA	TT - Tetanus toxoid vaccination	90.0	73.3
United Republic of Tanzania	TZA	IPTp - Intermittent preventive treatment of malaria during pregnancy	56.1	45.7
United Republic of Tanzania	TZA	Syphilis detection and treatment	56.2	45.8
United Republic of Tanzania	TZA	Iron supplementation in pregnancy	21.4	17.4
United Republic of Tanzania	TZA	Hypertensive disorder case management	11.8	9.6
United Republic of Tanzania	TZA	Diabetes case management	9.2	7.5

United Republic of Tanzania	TZA	Malaria case management	42.3	34.5
United Republic of Tanzania	TZA	MgSO4 management of pre-eclampsia	34.8	28.3
United Republic of Tanzania	TZA	Thermal protection	61.7	52.9
United Republic of Tanzania	TZA	Clean cord care	60.8	52.1
United Republic of Tanzania	TZA	Clean birth environment	51.6	44.2
United Republic of Tanzania	TZA	Immediate drying and additional stimulation	57.4	49.2
United Republic of Tanzania	TZA	Neonatal resuscitation	53.6	46.0
United Republic of Tanzania	TZA	Antibiotics for preterm or prolonged PROM	32.2	27.6
United Republic of Tanzania	TZA	Parenteral administration of anti-convulsants	49.9	42.8
United Republic of Tanzania	TZA	Parenteral administration of uterotonics	57.1	49.0
United Republic of Tanzania	TZA	Parenteral administration of antibiotics	32.2	27.6
United Republic of Tanzania	TZA	Assisted vaginal delivery	15.8	13.5
United Republic of Tanzania	TZA	Manual removal of placenta	24.5	21.0
United Republic of Tanzania	TZA	Removal of retained products of conception	15.3	13.1
United Republic of Tanzania	TZA	Cesarean delivery	5.4	4.6
United Republic of Tanzania	TZA	Blood transfusion	16.9	14.5
United Republic of Tanzania	TZA	Induction of labor for pregnancies lasting 41+ weeks	6.5	5.6
United Republic of Tanzania	TZA	Complementary feeding - education only	26.0	22.3
United Republic of Tanzania	TZA	Complementary feeding - supplementary feeding and education	26.0	22.3
United Republic of Tanzania	TZA	Vitamin A supplementation	87.0	74.6
United Republic of Tanzania	TZA	Improved sanitation - Utilization of latrines or toilets	29.9	29.9

United Republic of Tanzania	TZA	Improved water source	56.7	56.7
United Republic of Tanzania	TZA	Water connection in the home	20.3	20.3
United Republic of Tanzania	TZA	Hand washing with soap	51.9	51.9
United Republic of Tanzania	TZA	Hygienic disposal of children's stools	67.9	67.9
United Republic of Tanzania	TZA	ITN/IRS - Households protected from malaria	77.9	66.8
United Republic of Tanzania	TZA	Injectable antibiotics for neonatal sepsis	62.6	51.0
United Republic of Tanzania	TZA	ORS - oral rehydration solution	44.8	38.4
United Republic of Tanzania	TZA	Antibiotics for treatment of dysentery	2.8	2.4
United Republic of Tanzania	TZA	Zinc for treatment of diarrhea	17.5	15.0
United Republic of Tanzania	TZA	Oral antibiotics for pneumonia	55.4	47.5
United Republic of Tanzania	TZA	Vitamin A for treatment of measles	87.0	74.6
United Republic of Tanzania	TZA	ACTs- Artemisinin compounds for treatment of malaria	30.0	25.7
United Republic of Tanzania	TZA	SAM - treatment for severe acute malnutrition	0.3	0.3
United Republic of Tanzania	TZA	BCG vaccine	99.0	80.6
United Republic of Tanzania	TZA	Polio vaccine	91.0	74.1
United Republic of Tanzania	TZA	DPT vaccine	98.0	79.8
United Republic of Tanzania	TZA	H. influenzae type b vaccine	98.0	92.2
United Republic of Tanzania	TZA	HepB vaccine	98.0	79.8
United Republic of Tanzania	TZA	Pneumococcal vaccine	98.0	92.2
United Republic of Tanzania	TZA	Rotavirus vaccine	98.0	92.2
United Republic of Tanzania	TZA	Measles vaccine	99.0	80.6

United Republic of Tanzania	TZA	Global wasting (<-2 SD) rate	4.6	5.1
United Republic of Tanzania	TZA	Contraceptive prevalence (CPR)	43.8	39.5
Uzbekistan	UZB	Safe abortion services	35.1	31.7
Uzbekistan	UZB	Syphilis detection and treatment	24.5	20.0
Uzbekistan	UZB	Hypertensive disorder case management	18.8	15.3
Uzbekistan	UZB	Diabetes case management	14.7	12.0
Uzbekistan	UZB	Malaria case management	60.7	49.4
Uzbekistan	UZB	MgSO4 management of pre-eclampsia	37.2	30.3
Uzbekistan	UZB	Thermal protection	96.2	82.5
Uzbekistan	UZB	Clean cord care	92.9	79.7
Uzbekistan	UZB	Clean birth environment	79.8	68.4
Uzbekistan	UZB	Immediate drying and additional stimulation	89.1	76.4
Uzbekistan	UZB	Neonatal resuscitation	53.5	45.9
Uzbekistan	UZB	Antibiotics for preterm or prolonged PROM	72.8	62.4
Uzbekistan	UZB	Parenteral administration of anti-convulsants	69.6	59.7
Uzbekistan	UZB	Parenteral administration of uterotonics	87.0	74.6
Uzbekistan	UZB	Parenteral administration of antibiotics	72.8	62.4
Uzbekistan	UZB	Assisted vaginal delivery	24.6	21.1
Uzbekistan	UZB	Manual removal of placenta	36.4	31.2
Uzbekistan	UZB	Removal of retained products of conception	32.3	27.7
Uzbekistan	UZB	Cesarean delivery	8.5	7.3
Uzbekistan	UZB	Blood transfusion	12.3	10.5
Uzbekistan	UZB	Induction of labor for pregnancies lasting 41+ weeks	1.7	1.5
Uzbekistan	UZB	Complementary feeding - education only	27.6	23.7
Uzbekistan	UZB	Complementary feeding - supplementary feeding and education	27.6	23.7
Uzbekistan	UZB	Vitamin A supplementation	99.0	84.9
Uzbekistan	UZB	Improved sanitation - Utilization of latrines or toilets	99.0	99.0
Uzbekistan	UZB	Improved water source	97.8	97.8
Uzbekistan	UZB	Water connection in the home	68.7	68.7
Uzbekistan	UZB	Hygienic disposal of children's stools	58.6	58.6
Uzbekistan	UZB	Injectable antibiotics for neonatal sepsis	97.3	79.3
Uzbekistan	UZB	ORS - oral rehydration solution	27.8	23.8

Uzbekistan	UZB	Oral antibiotics for pneumonia	67.7	58.0
Uzbekistan	UZB	Vitamin A for treatment of measles	99.0	84.9
Uzbekistan	UZB	BCG vaccine	96.0	78.2
Uzbekistan	UZB	Polio vaccine	98.0	79.8
Uzbekistan	UZB	DPT vaccine	98.0	79.8
Uzbekistan	UZB	H. influenzae type b vaccine	98.0	92.2
Uzbekistan	UZB	HepB vaccine	98.0	79.8
Uzbekistan	UZB	Pneumococcal vaccine	96.0	90.3
Uzbekistan	UZB	Rotavirus vaccine	84.0	79.0
Uzbekistan	UZB	Measles vaccine	99.0	80.6
Uzbekistan	UZB	Global wasting (<-2 SD) rate	4.4	4.9
Uzbekistan	UZB	Contraceptive prevalence (CPR)	68.35	61.7
Vanuatu	VUT	Safe abortion services	85.0	76.7
Vanuatu	VUT	TT - Tetanus toxoid vaccination	78.0	63.5
Vanuatu	VUT	Syphilis detection and treatment	19.1	15.6
Vanuatu	VUT	Hypertensive disorder case management	12.4	10.1
Vanuatu	VUT	Diabetes case management	9.7	7.9
Vanuatu	VUT	Malaria case management	40.2	32.7
Vanuatu	VUT	MgSO4 management of pre-eclampsia	24.6	20.0
Vanuatu	VUT	Thermal protection	87.5	75.0
Vanuatu	VUT	Clean cord care	84.5	72.4
Vanuatu	VUT	Clean birth environment	72.6	62.2
Vanuatu	VUT	Immediate drying and additional stimulation	81.0	69.4
Vanuatu	VUT	Neonatal resuscitation	48.7	41.8
Vanuatu	VUT	Antibiotics for preterm or prolonged PROM	66.2	56.8
Vanuatu	VUT	Parenteral administration of anti-convulsants	63.3	54.3
Vanuatu	VUT	Parenteral administration of uterotonics	79.1	67.8
Vanuatu	VUT	Parenteral administration of antibiotics	66.2	56.8
Vanuatu	VUT	Assisted vaginal delivery	22.4	19.2
Vanuatu	VUT	Manual removal of placenta	33.1	28.4
Vanuatu	VUT	Removal of retained products of conception	29.3	25.1
Vanuatu	VUT	Cesarean delivery	7.7	6.6
Vanuatu	VUT	Blood transfusion	11.1	9.5
Vanuatu	VUT	Induction of labor for pregnancies lasting 41+ weeks	1.6	1.4

Vanuatu	VUT	Improved sanitation - Utilization of latrines or toilets	34.1	34.1
Vanuatu	VUT	Improved water source	91.3	91.3
Vanuatu	VUT	Water connection in the home	43.5	43.5
Vanuatu	VUT	Hygienic disposal of children's stools	62.7	62.7
Vanuatu	VUT	ITN/IRS - Households protected from malaria	69.8	59.8
Vanuatu	VUT	Injectable antibiotics for neonatal sepsis	88.5	72.1
Vanuatu	VUT	ORS - oral rehydration solution	47.6	40.8
Vanuatu	VUT	Antibiotics for treatment of dysentery	3.0	2.6
Vanuatu	VUT	Zinc for treatment of diarrhea	1.0	0.9
Vanuatu	VUT	Oral antibiotics for pneumonia	72.1	61.8
Vanuatu	VUT	BCG vaccine	94.0	76.6
Vanuatu	VUT	Polio vaccine	85.0	69.2
Vanuatu	VUT	DPT vaccine	85.0	69.2
Vanuatu	VUT	H. influenzae type b vaccine	85.0	80.0
Vanuatu	VUT	HepB vaccine	85.0	69.2
Vanuatu	VUT	Measles vaccine	80.0	65.2
Vanuatu	VUT	Global wasting (<-2 SD) rate	5.7	6.3
Vanuatu	VUT	Contraceptive prevalence (CPR)	50.25	45.4
Venezuela	VEN	Safe abortion services	0.2	0.2
Venezuela	VEN	TT - Tetanus toxoid vaccination	70.0	57.0
Venezuela	VEN	Thermal protection	93.9	80.5
Venezuela	VEN	Clean cord care	90.7	77.8
Venezuela	VEN	Clean birth environment	77.9	66.8
Venezuela	VEN	Immediate drying and additional stimulation	87.0	74.6
Venezuela	VEN	Neonatal resuscitation	52.2	44.8
Venezuela	VEN	Antibiotics for preterm or prolonged PROM	71.1	61.0
Venezuela	VEN	Parenteral administration of anti-convulsants	67.9	58.2
Venezuela	VEN	Parenteral administration of uterotonics	84.9	72.8
Venezuela	VEN	Parenteral administration of antibiotics	71.1	61.0
Venezuela	VEN	Assisted vaginal delivery	24.0	20.6
Venezuela	VEN	Manual removal of placenta	35.5	30.4
Venezuela	VEN	Removal of retained products of conception	31.5	27.0
Venezuela	VEN	Cesarean delivery	8.3	7.1
Venezuela	VEN	Blood transfusion	12.0	10.3

Venezuela	VEN	Induction of labor for pregnancies lasting 41+ weeks	1.7	1.5
Venezuela	VEN	Improved sanitation - Utilization of latrines or toilets	93.9	93.9
Venezuela	VEN	Improved water source	95.7	95.7
Venezuela	VEN	Water connection in the home	83.7	83.7
Venezuela	VEN	Injectable antibiotics for neonatal sepsis	95.0	77.4
Venezuela	VEN	BCG vaccine	92.0	74.9
Venezuela	VEN	Polio vaccine	53.0	43.2
Venezuela	VEN	DPT vaccine	60.0	48.9
Venezuela	VEN	H. influenzae type b vaccine	60.0	56.4
Venezuela	VEN	HepB vaccine	60.0	48.9
Venezuela	VEN	Measles vaccine	96.0	78.2
Venezuela	VEN	Global wasting (<-2 SD) rate	2.4	2.7
Venezuela	VEN	Contraceptive prevalence (CPR)	75.7	68.3
Viet Nam	VNM	Safe abortion services	39.0	35.2
Viet Nam	VNM	TT - Tetanus toxoid vaccination	94.0	76.6
Viet Nam	VNM	IPTp - Intermittent preventive treatment of malaria during pregnancy	0.5	0.4
Viet Nam	VNM	Syphilis detection and treatment	23.7	19.3
Viet Nam	VNM	Hypertensive disorder case management	17.7	14.4
Viet Nam	VNM	Diabetes case management	13.8	11.2
Viet Nam	VNM	Malaria case management	57.1	46.5
Viet Nam	VNM	MgSO4 management of pre-eclampsia	35.0	28.5
Viet Nam	VNM	Thermal protection	92.5	79.3
Viet Nam	VNM	Clean cord care	89.4	76.6
Viet Nam	VNM	Clean birth environment	76.8	65.8
Viet Nam	VNM	Immediate drying and additional stimulation	85.7	73.5
Viet Nam	VNM	Neonatal resuscitation	51.5	44.2
Viet Nam	VNM	Antibiotics for preterm or prolonged PROM	70.1	60.1
Viet Nam	VNM	Parenteral administration of anti-convulsants	67.0	57.4
Viet Nam	VNM	Parenteral administration of uterotonics	83.7	71.8
Viet Nam	VNM	Parenteral administration of antibiotics	70.1	60.1
Viet Nam	VNM	Assisted vaginal delivery	23.7	20.3
Viet Nam	VNM	Manual removal of placenta	35.0	30.0
Viet Nam	VNM	Removal of retained products of conception	31.0	26.6
Viet Nam	VNM	Cesarean delivery	8.1	6.9

Viet Nam	VNM	Blood transfusion	11.8	10.1
Viet Nam	VNM	Induction of labor for pregnancies lasting 41+ weeks	1.6	1.4
Viet Nam	VNM	Complementary feeding - education only	76.9	65.9
Viet Nam	VNM	Complementary feeding - supplementary feeding and education	76.9	65.9
Viet Nam	VNM	Vitamin A supplementation	99.0	84.9
Viet Nam	VNM	Improved sanitation - Utilization of latrines or toilets	83.5	83.5
Viet Nam	VNM	Improved water source	94.7	94.7
Viet Nam	VNM	Water connection in the home	40.6	40.6
Viet Nam	VNM	Hand washing with soap	86.3	86.3
Viet Nam	VNM	Hygienic disposal of children's stools	57.7	57.7
Viet Nam	VNM	ITN/IRS - Households protected from malaria	24.9	21.3
Viet Nam	VNM	Injectable antibiotics for neonatal sepsis	93.6	76.2
Viet Nam	VNM	ORS - oral rehydration solution	50.9	43.6
Viet Nam	VNM	Zinc for treatment of diarrhea	16.9	14.5
Viet Nam	VNM	Oral antibiotics for pneumonia	81.1	69.5
Viet Nam	VNM	Vitamin A for treatment of measles	99.0	84.9
Viet Nam	VNM	ACTs- Artemisinin compounds for treatment of malaria	0.1	0.1
Viet Nam	VNM	SAM - treatment for severe acute malnutrition	0.2	0.2
Viet Nam	VNM	BCG vaccine	95.0	77.4
Viet Nam	VNM	Polio vaccine	90.0	73.3
Viet Nam	VNM	DPT vaccine	75.0	61.1
Viet Nam	VNM	H. influenzae type b vaccine	75.0	70.5
Viet Nam	VNM	HepB vaccine	75.0	61.1
Viet Nam	VNM	Measles vaccine	97.0	79.0
Viet Nam	VNM	Global wasting (<-2 SD) rate	4.2	4.6
Viet Nam	VNM	Contraceptive prevalence (CPR)	76.8	69.3
Yemen	YEM	Safe abortion services	40.0	36.1
Yemen	YEM	TT - Tetanus toxoid vaccination	70.0	57.0
Yemen	YEM	Syphilis detection and treatment	14.7	12.0
Yemen	YEM	Iron supplementation in pregnancy	5.5	4.5
Yemen	YEM	Hypertensive disorder case management	5.9	4.8
Yemen	YEM	Diabetes case management	4.6	3.7
Yemen	YEM	Malaria case management	19.1	15.6

Yemen	YEM	MgSO4 management of pre-eclampsia	11.7	9.5
Yemen	YEM	Thermal protection	30.3	26.0
Yemen	YEM	Clean cord care	29.3	25.1
Yemen	YEM	Clean birth environment	25.2	21.6
Yemen	YEM	Immediate drying and additional stimulation	28.1	24.1
Yemen	YEM	Neonatal resuscitation	16.9	14.5
Yemen	YEM	Antibiotics for preterm or prolonged PROM	22.9	19.6
Yemen	YEM	Parenteral administration of anti-convulsants	21.9	18.8
Yemen	YEM	Parenteral administration of uterotonics	27.4	23.5
Yemen	YEM	Parenteral administration of antibiotics	22.9	19.6
Yemen	YEM	Assisted vaginal delivery	7.8	6.7
Yemen	YEM	Manual removal of placenta	11.5	9.9
Yemen	YEM	Removal of retained products of conception	10.2	8.7
Yemen	YEM	Cesarean delivery	2.7	2.3
Yemen	YEM	Blood transfusion	3.9	3.3
Yemen	YEM	Induction of labor for pregnancies lasting 41+ weeks	0.5	0.4
Yemen	YEM	Complementary feeding - education only	26.6	22.8
Yemen	YEM	Complementary feeding - supplementary feeding and education	26.6	22.8
Yemen	YEM	Vitamin A supplementation	81.0	69.4
Yemen	YEM	Improved sanitation - Utilization of latrines or toilets	59.1	59.1
Yemen	YEM	Improved water source	63.5	63.5
Yemen	YEM	Water connection in the home	30.7	30.7
Yemen	YEM	Hand washing with soap	62.0	62.0
Yemen	YEM	Hygienic disposal of children's stools	18.6	18.6
Yemen	YEM	Injectable antibiotics for neonatal sepsis	30.7	25.0
Yemen	YEM	ORS - oral rehydration solution	25.3	21.7
Yemen	YEM	Antibiotics for treatment of dysentery	38.2	32.8
Yemen	YEM	Zinc for treatment of diarrhea	0.4	0.3
Yemen	YEM	Oral antibiotics for pneumonia	34.0	29.2
Yemen	YEM	Vitamin A for treatment of measles	81.0	69.4
Yemen	YEM	BCG vaccine	64.0	52.1
Yemen	YEM	Polio vaccine	59.0	48.1
Yemen	YEM	DPT vaccine	65.0	52.9
Yemen	YEM	H. influenzae type b vaccine	65.0	61.1

Yemen	YEM	HepB vaccine	65.0	52.9
Yemen	YEM	Pneumococcal vaccine	64.0	60.2
Yemen	YEM	Rotavirus vaccine	64.0	60.2
Yemen	YEM	Measles vaccine	65.0	52.9
Yemen	YEM	Global wasting (<-2 SD) rate	16.3	18.0
Yemen	YEM	Contraceptive prevalence (CPR)	41.4	37.4
Zambia	ZMB	Safe abortion services	3.3	3.0
Zambia	ZMB	TT - Tetanus toxoid vaccination	85.0	69.2
Zambia	ZMB	IPTp - Intermittent preventive treatment of malaria during pregnancy	81.3	66.2
Zambia	ZMB	Syphilis detection and treatment	23.6	19.2
Zambia	ZMB	Iron supplementation in pregnancy	59.1	48.1
Zambia	ZMB	Hypertensive disorder case management	13.0	10.6
Zambia	ZMB	Diabetes case management	10.1	8.2
Zambia	ZMB	Malaria case management	42.0	34.2
Zambia	ZMB	MgSO4 management of pre-eclampsia	25.7	20.9
Zambia	ZMB	Thermal protection	66.4	56.9
Zambia	ZMB	Clean cord care	64.1	55.0
Zambia	ZMB	Clean birth environment	55.1	47.2
Zambia	ZMB	Immediate drying and additional stimulation	61.5	52.7
Zambia	ZMB	Neonatal resuscitation	36.9	31.6
Zambia	ZMB	Antibiotics for preterm or prolonged PROM	50.2	43.0
Zambia	ZMB	Parenteral administration of anti-convulsants	48.0	41.2
Zambia	ZMB	Parenteral administration of uterotonics	60.0	51.4
Zambia	ZMB	Parenteral administration of antibiotics	50.2	43.0
Zambia	ZMB	Assisted vaginal delivery	17.0	14.6
Zambia	ZMB	Manual removal of placenta	25.1	21.5
Zambia	ZMB	Removal of retained products of conception	22.2	19.0
Zambia	ZMB	Cesarean delivery	5.8	5.0
Zambia	ZMB	Blood transfusion	8.5	7.3
Zambia	ZMB	Induction of labor for pregnancies lasting 41+ weeks	1.2	1.0
Zambia	ZMB	Complementary feeding - education only	22.0	18.9
Zambia	ZMB	Complementary feeding - supplementary feeding and education	22.0	18.9
Zambia	ZMB	Vitamin A supplementation	99.0	84.9

Zambia	ZMB	Improved sanitation - Utilization of latrines or toilets	26.4	26.4
Zambia	ZMB	Improved water source	60.0	60.0
Zambia	ZMB	Water connection in the home	19.1	19.1
Zambia	ZMB	Hand washing with soap	33.7	33.7
Zambia	ZMB	Hygienic disposal of children's stools	70.8	70.8
Zambia	ZMB	ITN/IRS - Households protected from malaria	84.0	72.0
Zambia	ZMB	Injectable antibiotics for neonatal sepsis	67.1	54.7
Zambia	ZMB	ORS - oral rehydration solution	52.5	45.0
Zambia	ZMB	Antibiotics for treatment of dysentery	37.8	32.4
Zambia	ZMB	Oral antibiotics for pneumonia	69.7	59.8
Zambia	ZMB	Vitamin A for treatment of measles	99.0	84.9
Zambia	ZMB	ACTs- Artemisinin compounds for treatment of malaria	18.4	15.8
Zambia	ZMB	BCG vaccine	91.0	74.1
Zambia	ZMB	Polio vaccine	90.0	73.3
Zambia	ZMB	DPT vaccine	90.0	73.3
Zambia	ZMB	H. influenzae type b vaccine	90.0	84.7
Zambia	ZMB	HepB vaccine	90.0	73.3
Zambia	ZMB	Pneumococcal vaccine	90.0	84.7
Zambia	ZMB	Rotavirus vaccine	91.0	85.6
Zambia	ZMB	Measles vaccine	96.0	78.2
Zambia	ZMB	Global wasting (<-2 SD) rate	6.3	6.9
Zambia	ZMB	Contraceptive prevalence (CPR)	55.3	49.9
Zimbabwe	ZWE	Safe abortion services	3.3	3.0
Zimbabwe	ZWE	TT - Tetanus toxoid vaccination	87.0	70.9
Zimbabwe	ZWE	IPTp - Intermittent preventive treatment of malaria during pregnancy	25.9	21.1
Zimbabwe	ZWE	Syphilis detection and treatment	80.5	65.6
Zimbabwe	ZWE	Iron supplementation in pregnancy	39.7	32.3
Zimbabwe	ZWE	Hypertensive disorder case management	28.7	23.4
Zimbabwe	ZWE	Diabetes case management	27.8	22.6
Zimbabwe	ZWE	Malaria case management	68.3	55.6
Zimbabwe	ZWE	MgSO4 management of pre-eclampsia	69.4	56.5
Zimbabwe	ZWE	Thermal protection	85.5	73.3
Zimbabwe	ZWE	Clean cord care	75.5	64.7
Zimbabwe	ZWE	Clean birth environment	67.6	58.0

Zimbabwe	ZWE	Immediate drying and additional stimulation	76.3	65.4
Zimbabwe	ZWE	Neonatal resuscitation	70.4	60.4
Zimbabwe	ZWE	Antibiotics for preterm or prolonged PROM	45.9	39.4
Zimbabwe	ZWE	Parenteral administration of anti-convulsants	85.3	73.1
Zimbabwe	ZWE	Parenteral administration of uterotonics	82.2	70.5
Zimbabwe	ZWE	Parenteral administration of antibiotics	45.9	39.4
Zimbabwe	ZWE	Assisted vaginal delivery	18.0	15.4
Zimbabwe	ZWE	Manual removal of placenta	8.5	7.3
Zimbabwe	ZWE	Removal of retained products of conception	16.8	14.4
Zimbabwe	ZWE	Cesarean delivery	0.5	0.4
Zimbabwe	ZWE	Blood transfusion	10.8	9.3
Zimbabwe	ZWE	Induction of labor for pregnancies lasting 41+ weeks	1.5	1.3
Zimbabwe	ZWE	Complementary feeding - education only	16.7	14.3
Zimbabwe	ZWE	Complementary feeding - supplementary feeding and education	16.7	14.3
Zimbabwe	ZWE	Vitamin A supplementation	43.0	36.9
Zimbabwe	ZWE	Improved sanitation - Utilization of latrines or toilets	36.2	36.2
Zimbabwe	ZWE	Improved water source	64.1	64.1
Zimbabwe	ZWE	Water connection in the home	19.9	19.9
Zimbabwe	ZWE	Hand washing with soap	64.2	64.2
Zimbabwe	ZWE	Hygienic disposal of children's stools	64.4	64.4
Zimbabwe	ZWE	ITN/IRS - Households protected from malaria	36.8	31.6
Zimbabwe	ZWE	Injectable antibiotics for neonatal sepsis	85.5	69.6
Zimbabwe	ZWE	ORS - oral rehydration solution	32.8	28.1
Zimbabwe	ZWE	Antibiotics for treatment of dysentery	8.0	6.9
Zimbabwe	ZWE	Zinc for treatment of diarrhea	22.5	19.3
Zimbabwe	ZWE	Oral antibiotics for pneumonia	50.9	43.6
Zimbabwe	ZWE	Vitamin A for treatment of measles	43.0	36.9
Zimbabwe	ZWE	ACTs- Artemisinin compounds for treatment of malaria	0.4	0.3
Zimbabwe	ZWE	SAM - treatment for severe acute malnutrition	8.9	7.6
Zimbabwe	ZWE	BCG vaccine	95.0	77.4
Zimbabwe	ZWE	Polio vaccine	89.0	72.5
Zimbabwe	ZWE	DPT vaccine	89.0	72.5
Zimbabwe	ZWE	H. influenzae type b vaccine	89.0	83.7

Zimbabwe	ZWE	HepB vaccine	89.0	72.5
Zimbabwe	ZWE	Pneumococcal vaccine	89.0	83.7
Zimbabwe	ZWE	Rotavirus vaccine	90.0	84.7
Zimbabwe	ZWE	Measles vaccine	90.0	73.3
Zimbabwe	ZWE	Global wasting (<-2 SD) rate	3.6	3.9
Zimbabwe	ZWE	Contraceptive prevalence (CPR)	68.3	61.6

Appendix B2. Country-specific coverage reductions for Scenario 2

Country	ISO 3166-1 alpha-3	Intervention Name	Baseline coverage (%)	Ending coverage (%)
Afghanistan	AFG	Safe abortion services	35.1	28.5
Afghanistan	AFG	TT - Tetanus toxoid vaccination	70.0	51.2
Afghanistan	AFG	Syphilis detection and treatment	14.6	10.7
Afghanistan	AFG	Iron supplementation in pregnancy	6.8	5.0
Afghanistan	AFG	Hypertensive disorder case management	4.3	3.1
Afghanistan	AFG	Diabetes case management	3.3	2.4
Afghanistan	AFG	Malaria case management	13.8	10.1
Afghanistan	AFG	MgSO4 management of pre-eclampsia	8.5	6.2
Afghanistan	AFG	Thermal protection	47.7	36.7
Afghanistan	AFG	Clean cord care	46.1	35.5
Afghanistan	AFG	Clean birth environment	39.6	30.5
Afghanistan	AFG	Immediate drying and additional stimulation	44.2	34.0
Afghanistan	AFG	Neonatal resuscitation	26.6	20.5
Afghanistan	AFG	Antibiotics for preterm or prolonged PROM	36.1	27.8
Afghanistan	AFG	Parenteral administration of anti-convulsants	34.5	26.5
Afghanistan	AFG	Parenteral administration of uterotonics	43.2	33.2
Afghanistan	AFG	Parenteral administration of antibiotics	36.1	27.8
Afghanistan	AFG	Assisted vaginal delivery	12.2	9.4
Afghanistan	AFG	Manual removal of placenta	18.1	13.9
Afghanistan	AFG	Removal of retained products of conception	16.0	12.3
Afghanistan	AFG	Cesarean delivery	4.2	3.2
Afghanistan	AFG	Blood transfusion	6.1	4.7
Afghanistan	AFG	Induction of labor for pregnancies lasting 41+ weeks	0.9	0.7
Afghanistan	AFG	Complementary feeding - education only	24.3	18.8
Afghanistan	AFG	Complementary feeding - supplementary feeding and education	24.3	18.8
Afghanistan	AFG	Vitamin A supplementation	95.0	73.3
Afghanistan	AFG	Improved sanitation - Utilization of latrines or toilets	43.4	43.4
Afghanistan	AFG	Improved water source	67.1	67.1
Afghanistan	AFG	Water connection in the home	14.5	14.5
Afghanistan	AFG	Hand washing with soap	45.6	45.6
Afghanistan	AFG	Hygienic disposal of children's stools	30.9	30.9
Afghanistan	AFG	ITN/IRS - Households protected from malaria	26.0	20.1
Afghanistan	AFG	Injectable antibiotics for neonatal sepsis	48.3	35.3
Afghanistan	AFG	ORS - oral rehydration solution	46.2	35.6
Afghanistan	AFG	Antibiotics for treatment of dysentery	8.2	6.3
Afghanistan	AFG	Zinc for treatment of diarrhea	9.6	7.4
Afghanistan	AFG	Oral antibiotics for pneumonia	61.4	47.2
Afghanistan	AFG	Vitamin A for treatment of measles	95.0	73.1

Afghanistan	AFG	SAM - treatment for severe acute malnutrition	9.2	7.1
Afghanistan	AFG	BCG vaccine	78.0	57.0
Afghanistan	AFG	Polio vaccine	73.0	53.4
Afghanistan	AFG	DPT vaccine	66.0	48.2
Afghanistan	AFG	H. influenzae type b vaccine	66.0	60.3
Afghanistan	AFG	HepB vaccine	66.0	48.2
Afghanistan	AFG	Pneumococcal vaccine	65.0	59.4
Afghanistan	AFG	Rotavirus vaccine	60.0	54.8
Afghanistan	AFG	Measles vaccine	64.0	46.8
Afghanistan	AFG	Global wasting (<-2 SD) rate	10.6	12.7
Afghanistan	AFG	Contraceptive prevalence (CPR)	27.8	22.6
Albania	ALB	Safe abortion services	100.0	81.2
Albania	ALB	TT - Tetanus toxoid vaccination	95.0	69.4
Albania	ALB	Syphilis detection and treatment	21.9	16.0
Albania	ALB	Iron supplementation in pregnancy	18.5	13.5
Albania	ALB	Hypertensive disorder case management	19.0	13.9
Albania	ALB	Diabetes case management	14.8	10.8
Albania	ALB	Malaria case management	61.2	44.7
Albania	ALB	MgSO4 management of pre-eclampsia	37.5	27.4
Albania	ALB	Thermal protection	97.4	74.9
Albania	ALB	Clean cord care	94.1	72.4
Albania	ALB	Clean birth environment	80.9	62.3
Albania	ALB	Immediate drying and additional stimulation	90.3	69.5
Albania	ALB	Neonatal resuscitation	54.2	41.7
Albania	ALB	Antibiotics for preterm or prolonged PROM	73.8	56.8
Albania	ALB	Parenteral administration of anti-convulsants	70.5	54.2
Albania	ALB	Parenteral administration of uterotonics	88.1	67.8
Albania	ALB	Parenteral administration of antibiotics	73.8	56.8
Albania	ALB	Assisted vaginal delivery	24.9	19.2
Albania	ALB	Manual removal of placenta	36.9	28.4
Albania	ALB	Removal of retained products of conception	32.7	25.2
Albania	ALB	Cesarean delivery	8.6	6.6
Albania	ALB	Blood transfusion	12.4	9.5
Albania	ALB	Induction of labor for pregnancies lasting 41+ weeks	1.7	1.3
Albania	ALB	Complementary feeding - education only	59.3	45.8
Albania	ALB	Complementary feeding - supplementary feeding and education	59.3	45.8
Albania	ALB	Improved sanitation - Utilization of latrines or toilets	97.7	97.7
Albania	ALB	Improved water source	91.0	91.0
Albania	ALB	Water connection in the home	78.5	78.5
Albania	ALB	Hygienic disposal of children's stools	13.5	13.5
Albania	ALB	Injectable antibiotics for neonatal sepsis	98.6	72.1

Albania	ALB	ORS - oral rehydration solution	34.5	26.5
Albania	ALB	Antibiotics for treatment of dysentery	38.7	29.8
Albania	ALB	Zinc for treatment of diarrhea	14.9	11.5
Albania	ALB	Oral antibiotics for pneumonia	81.8	62.9
Albania	ALB	BCG vaccine	99.0	72.4
Albania	ALB	Polio vaccine	99.0	72.4
Albania	ALB	DPT vaccine	99.0	72.4
Albania	ALB	H. influenzae type b vaccine	99.0	90.5
Albania	ALB	HepB vaccine	99.0	72.4
Albania	ALB	Pneumococcal vaccine	98.0	89.6
Albania	ALB	Measles vaccine	94.0	68.7
Albania	ALB	Global wasting (<-2 SD) rate	1.4	1.7
Albania	ALB	Contraceptive prevalence (CPR)	44	35.7
Algeria	DZA	Safe abortion services	2.2	1.8
Algeria	DZA	TT - Tetanus toxoid vaccination	98.0	71.6
Algeria	DZA	Syphilis detection and treatment	22.9	16.7
Algeria	DZA	Hypertensive disorder case management	16.2	11.8
Algeria	DZA	Diabetes case management	12.6	9.2
Algeria	DZA	Malaria case management	52.1	38.1
Algeria	DZA	MgSO4 management of pre-eclampsia	32.0	23.4
Algeria	DZA	Thermal protection	95.5	73.5
Algeria	DZA	Clean cord care	92.2	70.9
Algeria	DZA	Clean birth environment	79.2	60.9
Algeria	DZA	Immediate drying and additional stimulation	88.5	68.1
Algeria	DZA	Neonatal resuscitation	53.1	40.9
Algeria	DZA	Antibiotics for preterm or prolonged PROM	72.3	55.6
Algeria	DZA	Parenteral administration of anti-convulsants	69.1	53.2
Algeria	DZA	Parenteral administration of uterotonics	86.3	66.4
Algeria	DZA	Parenteral administration of antibiotics	72.3	55.6
Algeria	DZA	Assisted vaginal delivery	24.4	18.8
Algeria	DZA	Manual removal of placenta	36.1	27.8
Algeria	DZA	Removal of retained products of conception	32.0	24.6
Algeria	DZA	Cesarean delivery	8.4	6.5
Algeria	DZA	Blood transfusion	12.2	9.4
Algeria	DZA	Induction of labor for pregnancies lasting 41+ weeks	1.7	1.3
Algeria	DZA	Improved sanitation - Utilization of latrines or toilets	87.6	87.6
Algeria	DZA	Improved water source	93.6	93.6
Algeria	DZA	Water connection in the home	71.8	71.8
Algeria	DZA	Hand washing with soap	84.2	84.2
Algeria	DZA	Hygienic disposal of children's stools	17.7	17.7
Algeria	DZA	Injectable antibiotics for neonatal sepsis	96.6	70.6
Algeria	DZA	ORS - oral rehydration solution	33.9	26.1

Algeria	DZA	Oral antibiotics for pneumonia	66.4	51.1
Algeria	DZA	BCG vaccine	99.0	72.4
Algeria	DZA	Polio vaccine	91.0	66.5
Algeria	DZA	DPT vaccine	91.0	66.5
Algeria	DZA	H. influenzae type b vaccine	91.0	83.2
Algeria	DZA	HepB vaccine	91.0	66.5
Algeria	DZA	Pneumococcal vaccine	91.0	83.2
Algeria	DZA	Measles vaccine	80.0	58.5
Algeria	DZA	Global wasting (<-2 SD) rate	4.0	4.8
Algeria	DZA	Contraceptive prevalence (CPR)	61.05	49.6
Angola	AGO	TT - Tetanus toxoid vaccination	78.0	57.0
Angola	AGO	IPTp - Intermittent preventive treatment of malaria during pregnancy	38.4	28.1
Angola	AGO	Syphilis detection and treatment	20.0	14.6
Angola	AGO	Iron supplementation in pregnancy	32.1	23.5
Angola	AGO	Hypertensive disorder case management	14.5	10.6
Angola	AGO	Diabetes case management	11.3	8.3
Angola	AGO	Malaria case management	46.7	34.1
Angola	AGO	MgSO4 management of pre-eclampsia	28.6	20.9
Angola	AGO	Thermal protection	45.1	34.7
Angola	AGO	Clean cord care	43.5	33.5
Angola	AGO	Clean birth environment	37.4	28.8
Angola	AGO	Immediate drying and additional stimulation	41.8	32.2
Angola	AGO	Neonatal resuscitation	25.1	19.3
Angola	AGO	Antibiotics for preterm or prolonged PROM	34.1	26.2
Angola	AGO	Parenteral administration of anti-convulsants	32.6	25.1
Angola	AGO	Parenteral administration of uterotonics	40.8	31.4
Angola	AGO	Parenteral administration of antibiotics	34.1	26.2
Angola	AGO	Assisted vaginal delivery	11.5	8.8
Angola	AGO	Manual removal of placenta	17.1	13.2
Angola	AGO	Removal of retained products of conception	15.1	11.6
Angola	AGO	Cesarean delivery	4.0	3.1
Angola	AGO	Blood transfusion	5.7	4.4
Angola	AGO	Induction of labor for pregnancies lasting 41+ weeks	0.8	0.6
Angola	AGO	Complementary feeding - education only	33.3	25.7
Angola	AGO	Complementary feeding - supplementary feeding and education	33.3	25.7
Angola	AGO	Vitamin A supplementation	3.0	2.3
Angola	AGO	Improved sanitation - Utilization of latrines or toilets	49.9	49.9
Angola	AGO	Improved water source	55.8	55.8
Angola	AGO	Water connection in the home	22.6	22.6
Angola	AGO	Hand washing with soap	27.0	27.0
Angola	AGO	Hygienic disposal of children's stools	27.5	27.5

Angola	AGO	ITN/IRS - Households protected from malaria	31.8	24.5
Angola	AGO	Injectable antibiotics for neonatal sepsis	45.6	33.3
Angola	AGO	ORS - oral rehydration solution	42.6	32.8
Angola	AGO	Oral antibiotics for pneumonia	58.7	45.2
Angola	AGO	Vitamin A for treatment of measles	3.0	2.3
Angola	AGO	ACTs- Artemisinin compounds for treatment of malaria	9.0	6.9
Angola	AGO	SAM - treatment for severe acute malnutrition	1.4	1.1
Angola	AGO	BCG vaccine	86.0	62.9
Angola	AGO	Polio vaccine	56.0	40.9
Angola	AGO	DPT vaccine	59.0	43.1
Angola	AGO	H. influenzae type b vaccine	59.0	53.9
Angola	AGO	HepB vaccine	59.0	43.1
Angola	AGO	Pneumococcal vaccine	67.0	61.2
Angola	AGO	Rotavirus vaccine	65.0	59.4
Angola	AGO	Measles vaccine	50.0	36.6
Angola	AGO	Global wasting (<-2 SD) rate	4.9	5.9
Angola	AGO	Contraceptive prevalence (CPR)	16.25	13.2
Argentina	ARG	Safe abortion services	0.2	0.2
Argentina	ARG	Syphilis detection and treatment	24.2	17.7
Argentina	ARG	Hypertensive disorder case management	21.6	15.8
Argentina	ARG	Diabetes case management	16.8	12.3
Argentina	ARG	Malaria case management	69.6	50.9
Argentina	ARG	MgSO4 management of pre-eclampsia	42.7	31.2
Argentina	ARG	Thermal protection	97.1	74.7
Argentina	ARG	Clean cord care	93.7	72.1
Argentina	ARG	Clean birth environment	80.5	61.9
Argentina	ARG	Immediate drying and additional stimulation	89.9	69.2
Argentina	ARG	Neonatal resuscitation	54.0	41.6
Argentina	ARG	Antibiotics for preterm or prolonged PROM	73.5	56.6
Argentina	ARG	Parenteral administration of anti-convulsants	70.2	54.0
Argentina	ARG	Parenteral administration of uterotonics	87.8	67.6
Argentina	ARG	Parenteral administration of antibiotics	73.5	56.6
Argentina	ARG	Assisted vaginal delivery	24.8	19.1
Argentina	ARG	Manual removal of placenta	36.7	28.2
Argentina	ARG	Removal of retained products of conception	32.5	25.0
Argentina	ARG	Cesarean delivery	8.5	6.5
Argentina	ARG	Blood transfusion	12.4	9.5
Argentina	ARG	Induction of labor for pregnancies lasting 41+ weeks	1.7	1.3
Argentina	ARG	Improved sanitation - Utilization of latrines or toilets	94.3	94.3
Argentina	ARG	Improved water source	99.0	99.0
Argentina	ARG	Water connection in the home	95.7	95.7

Argentina	ARG	Injectable antibiotics for neonatal sepsis	98.2	71.8
Argentina	ARG	ORS - oral rehydration solution	17.5	13.5
Argentina	ARG	Oral antibiotics for pneumonia	94.5	72.7
Argentina	ARG	BCG vaccine	93.0	68.0
Argentina	ARG	Polio vaccine	84.0	61.4
Argentina	ARG	DPT vaccine	86.0	62.9
Argentina	ARG	H. influenzae type b vaccine	86.0	78.6
Argentina	ARG	HepB vaccine	86.0	62.9
Argentina	ARG	Pneumococcal vaccine	88.0	80.4
Argentina	ARG	Rotavirus vaccine	80.0	73.1
Argentina	ARG	Measles vaccine	89.0	65.1
Argentina	ARG	Global wasting (<-2 SD) rate	2.4	2.9
Argentina	ARG	Contraceptive prevalence (CPR)	63.95	51.9
Armenia	ARM	Safe abortion services	40.0	32.5
Armenia	ARM	Syphilis detection and treatment	24.7	18.1
Armenia	ARM	Iron supplementation in pregnancy	4.5	3.3
Armenia	ARM	Hypertensive disorder case management	23.2	17.0
Armenia	ARM	Diabetes case management	18.1	13.2
Armenia	ARM	Malaria case management	74.7	54.6
Armenia	ARM	MgSO4 management of pre-eclampsia	45.8	33.5
Armenia	ARM	Thermal protection	98.5	75.8
Armenia	ARM	Clean cord care	95.1	73.2
Armenia	ARM	Clean birth environment	81.8	62.9
Armenia	ARM	Immediate drying and additional stimulation	91.3	70.3
Armenia	ARM	Neonatal resuscitation	54.8	42.2
Armenia	ARM	Antibiotics for preterm or prolonged PROM	74.6	57.4
Armenia	ARM	Parenteral administration of anti-convulsants	71.3	54.9
Armenia	ARM	Parenteral administration of uterotonics	89.1	68.6
Armenia	ARM	Parenteral administration of antibiotics	74.6	57.4
Armenia	ARM	Assisted vaginal delivery	25.2	19.4
Armenia	ARM	Manual removal of placenta	37.3	28.7
Armenia	ARM	Removal of retained products of conception	33.0	25.4
Armenia	ARM	Cesarean delivery	8.7	6.7
Armenia	ARM	Blood transfusion	12.6	9.7
Armenia	ARM	Induction of labor for pregnancies lasting 41+ weeks	1.8	1.4
Armenia	ARM	Complementary feeding - education only	50.2	38.7
Armenia	ARM	Complementary feeding - supplementary feeding and education	50.2	38.7
Armenia	ARM	Improved sanitation - Utilization of latrines or toilets	93.6	93.6
Armenia	ARM	Improved water source	99.0	99.0
Armenia	ARM	Water connection in the home	97.9	97.9
Armenia	ARM	Hand washing with soap	95.8	95.8

Armenia	ARM	Hygienic disposal of children's stools	43.4	43.4
Armenia	ARM	Injectable antibiotics for neonatal sepsis	99.7	72.9
Armenia	ARM	ORS - oral rehydration solution	36.9	28.4
Armenia	ARM	Antibiotics for treatment of dysentery	1.3	1.0
Armenia	ARM	Zinc for treatment of diarrhea	3.7	2.8
Armenia	ARM	Oral antibiotics for pneumonia	91.7	70.6
Armenia	ARM	BCG vaccine	99.0	72.4
Armenia	ARM	Polio vaccine	92.0	67.3
Armenia	ARM	DPT vaccine	92.0	67.3
Armenia	ARM	H. influenzae type b vaccine	92.0	84.1
Armenia	ARM	HepB vaccine	92.0	67.3
Armenia	ARM	Pneumococcal vaccine	92.0	84.1
Armenia	ARM	Rotavirus vaccine	93.0	85.0
Armenia	ARM	Meningococcal A	22.0	16.1
Armenia	ARM	Measles vaccine	95.0	69.4
Armenia	ARM	Global wasting (<-2 SD) rate	4.5	5.4
Armenia	ARM	Contraceptive prevalence (CPR)	58.55	47.6
Azerbaijan	AZE	Safe abortion services	40.0	32.5
Azerbaijan	AZE	Syphilis detection and treatment	19.6	14.3
Azerbaijan	AZE	Iron supplementation in pregnancy	1.6	1.2
Azerbaijan	AZE	Hypertensive disorder case management	11.8	8.6
Azerbaijan	AZE	Diabetes case management	9.2	6.7
Azerbaijan	AZE	Malaria case management	38.1	27.9
Azerbaijan	AZE	MgSO4 management of pre-eclampsia	23.4	17.1
Azerbaijan	AZE	Thermal protection	76.8	59.1
Azerbaijan	AZE	Clean cord care	74.1	57.0
Azerbaijan	AZE	Clean birth environment	63.7	49.0
Azerbaijan	AZE	Immediate drying and additional stimulation	71.1	54.7
Azerbaijan	AZE	Neonatal resuscitation	42.7	32.9
Azerbaijan	AZE	Antibiotics for preterm or prolonged PROM	58.1	44.7
Azerbaijan	AZE	Parenteral administration of anti-convulsants	55.5	42.7
Azerbaijan	AZE	Parenteral administration of uterotonics	69.4	53.4
Azerbaijan	AZE	Parenteral administration of antibiotics	58.1	44.7
Azerbaijan	AZE	Assisted vaginal delivery	19.6	15.1
Azerbaijan	AZE	Manual removal of placenta	29.0	22.3
Azerbaijan	AZE	Removal of retained products of conception	25.7	19.8
Azerbaijan	AZE	Cesarean delivery	6.8	5.2
Azerbaijan	AZE	Blood transfusion	9.8	7.5
Azerbaijan	AZE	Induction of labor for pregnancies lasting 41+ weeks	1.4	1.1
Azerbaijan	AZE	Complementary feeding - education only	50.9	39.3
Azerbaijan	AZE	Complementary feeding - supplementary feeding and education	50.9	39.3
Azerbaijan	AZE	Vitamin A supplementation	41.0	31.6

Azerbaijan	AZE	Improved sanitation - Utilization of latrines or toilets	92.5	92.5
Azerbaijan	AZE	Improved water source	91.4	91.4
Azerbaijan	AZE	Water connection in the home	78.5	78.5
Azerbaijan	AZE	Injectable antibiotics for neonatal sepsis	77.7	56.8
Azerbaijan	AZE	ORS - oral rehydration solution	21.1	16.2
Azerbaijan	AZE	Oral antibiotics for pneumonia	32.5	25.0
Azerbaijan	AZE	Vitamin A for treatment of measles	41.0	31.5
Azerbaijan	AZE	BCG vaccine	97.0	70.9
Azerbaijan	AZE	Polio vaccine	96.0	70.2
Azerbaijan	AZE	DPT vaccine	95.0	69.4
Azerbaijan	AZE	H. influenzae type b vaccine	95.0	86.8
Azerbaijan	AZE	HepB vaccine	95.0	69.4
Azerbaijan	AZE	Pneumococcal vaccine	95.0	86.8
Azerbaijan	AZE	Measles vaccine	96.0	70.2
Azerbaijan	AZE	Global wasting (<-2 SD) rate	6.6	7.9
Azerbaijan	AZE	Contraceptive prevalence (CPR)	57	46.3
Bangladesh	BGD	Safe abortion services	35.1	28.5
Bangladesh	BGD	TT - Tetanus toxoid vaccination	98.0	71.6
Bangladesh	BGD	Syphilis detection and treatment	7.3	5.3
Bangladesh	BGD	Hypertensive disorder case management	8.9	6.5
Bangladesh	BGD	Diabetes case management	12.6	9.2
Bangladesh	BGD	Malaria case management	28.6	20.9
Bangladesh	BGD	MgSO4 management of pre-eclampsia	8.0	5.8
Bangladesh	BGD	Thermal protection	51.3	39.5
Bangladesh	BGD	Clean cord care	51.0	39.2
Bangladesh	BGD	Clean birth environment	37.8	29.1
Bangladesh	BGD	Immediate drying and additional stimulation	39.3	30.2
Bangladesh	BGD	Neonatal resuscitation	40.0	30.8
Bangladesh	BGD	Antibiotics for preterm or prolonged PROM	23.9	18.4
Bangladesh	BGD	Parenteral administration of anti-convulsants	37.8	29.1
Bangladesh	BGD	Parenteral administration of uterotonics	42.0	32.3
Bangladesh	BGD	Parenteral administration of antibiotics	23.9	18.4
Bangladesh	BGD	Assisted vaginal delivery	26.5	20.4
Bangladesh	BGD	Manual removal of placenta	12.2	9.4
Bangladesh	BGD	Removal of retained products of conception	30.2	23.2
Bangladesh	BGD	Cesarean delivery	7.6	5.8
Bangladesh	BGD	Blood transfusion	10.8	8.3
Bangladesh	BGD	Induction of labor for pregnancies lasting 41+ weeks	10.2	7.8
Bangladesh	BGD	Complementary feeding - education only	27.6	21.3
Bangladesh	BGD	Complementary feeding - supplementary feeding and education	27.6	21.3
Bangladesh	BGD	Vitamin A supplementation	99.0	76.4

Bangladesh	BGD	Improved sanitation - Utilization of latrines or toilets	48.2	48.2
Bangladesh	BGD	Improved water source	97.0	97.0
Bangladesh	BGD	Water connection in the home	14.5	14.5
Bangladesh	BGD	Hand washing with soap	74.8	74.8
Bangladesh	BGD	Hygienic disposal of children's stools	38.7	38.7
Bangladesh	BGD	Injectable antibiotics for neonatal sepsis	53.4	39.0
Bangladesh	BGD	ORS - oral rehydration solution	72.4	55.7
Bangladesh	BGD	Antibiotics for treatment of dysentery	9.7	7.5
Bangladesh	BGD	Zinc for treatment of diarrhea	43.6	33.6
Bangladesh	BGD	Oral antibiotics for pneumonia	46.4	35.7
Bangladesh	BGD	Vitamin A for treatment of measles	99.0	76.2
Bangladesh	BGD	SAM - treatment for severe acute malnutrition	0.3	0.2
Bangladesh	BGD	BCG vaccine	99.0	72.4
Bangladesh	BGD	Polio vaccine	98.0	71.6
Bangladesh	BGD	DPT vaccine	98.0	71.6
Bangladesh	BGD	H. influenzae type b vaccine	98.0	89.6
Bangladesh	BGD	HepB vaccine	98.0	71.6
Bangladesh	BGD	Pneumococcal vaccine	97.0	88.7
Bangladesh	BGD	Measles vaccine	97.0	70.9
Bangladesh	BGD	Global wasting (<-2 SD) rate	14.4	17.3
Bangladesh	BGD	Contraceptive prevalence (CPR)	65.15	52.9
Belarus	BLR	Safe abortion services	87.5	71.1
Belarus	BLR	Syphilis detection and treatment	24.6	18.0
Belarus	BLR	Hypertensive disorder case management	23.9	17.5
Belarus	BLR	Diabetes case management	18.7	13.7
Belarus	BLR	Malaria case management	77.3	56.5
Belarus	BLR	MgSO4 management of pre-eclampsia	47.4	34.7
Belarus	BLR	Thermal protection	98.7	75.9
Belarus	BLR	Clean cord care	95.3	73.3
Belarus	BLR	Clean birth environment	81.9	63.0
Belarus	BLR	Immediate drying and additional stimulation	91.5	70.4
Belarus	BLR	Neonatal resuscitation	54.9	42.2
Belarus	BLR	Antibiotics for preterm or prolonged PROM	74.8	57.6
Belarus	BLR	Parenteral administration of anti-convulsants	71.4	54.9
Belarus	BLR	Parenteral administration of uterotonics	89.3	68.7
Belarus	BLR	Parenteral administration of antibiotics	74.8	57.6
Belarus	BLR	Assisted vaginal delivery	25.3	19.5
Belarus	BLR	Manual removal of placenta	37.4	28.8
Belarus	BLR	Removal of retained products of conception	33.1	25.5
Belarus	BLR	Cesarean delivery	8.7	6.7
Belarus	BLR	Blood transfusion	12.6	9.7
Belarus	BLR	Induction of labor for pregnancies lasting 41+ weeks	1.8	1.4

Belarus	BLR	Improved sanitation - Utilization of latrines or toilets	97.8	97.8
Belarus	BLR	Improved water source	96.5	96.5
Belarus	BLR	Water connection in the home	89.6	89.6
Belarus	BLR	Hygienic disposal of children's stools	56.0	56.0
Belarus	BLR	Injectable antibiotics for neonatal sepsis	99.9	73.0
Belarus	BLR	ORS - oral rehydration solution	45.3	34.9
Belarus	BLR	Antibiotics for treatment of dysentery	22.3	17.2
Belarus	BLR	Oral antibiotics for pneumonia	93.4	71.9
Belarus	BLR	BCG vaccine	98.0	71.6
Belarus	BLR	Polio vaccine	98.0	71.6
Belarus	BLR	DPT vaccine	97.0	70.9
Belarus	BLR	H. influenzae type b vaccine	9.0	8.2
Belarus	BLR	HepB vaccine	98.0	71.6
Belarus	BLR	Measles vaccine	97.0	70.9
Belarus	BLR	Global wasting (<-2 SD) rate	2.2	2.6
Belarus	BLR	Contraceptive prevalence (CPR)	66.6	54.1
Belize	BLZ	Safe abortion services	0.6	0.5
Belize	BLZ	TT - Tetanus toxoid vaccination	91.0	66.5
Belize	BLZ	Syphilis detection and treatment	24.0	17.5
Belize	BLZ	Hypertensive disorder case management	22.3	16.3
Belize	BLZ	Diabetes case management	17.4	12.7
Belize	BLZ	Malaria case management	71.8	52.5
Belize	BLZ	MgSO4 management of pre-eclampsia	44.0	32.2
Belize	BLZ	Thermal protection	95.3	73.3
Belize	BLZ	Clean cord care	92.0	70.8
Belize	BLZ	Clean birth environment	79.1	60.9
Belize	BLZ	Immediate drying and additional stimulation	88.3	67.9
Belize	BLZ	Neonatal resuscitation	53.0	40.8
Belize	BLZ	Antibiotics for preterm or prolonged PROM	72.1	55.5
Belize	BLZ	Parenteral administration of anti-convulsants	68.9	53.0
Belize	BLZ	Parenteral administration of uterotonics	86.1	66.3
Belize	BLZ	Parenteral administration of antibiotics	72.1	55.5
Belize	BLZ	Assisted vaginal delivery	24.4	18.8
Belize	BLZ	Manual removal of placenta	36.1	27.8
Belize	BLZ	Removal of retained products of conception	31.9	24.5
Belize	BLZ	Cesarean delivery	8.4	6.5
Belize	BLZ	Blood transfusion	12.1	9.3
Belize	BLZ	Induction of labor for pregnancies lasting 41+ weeks	1.7	1.3
Belize	BLZ	Complementary feeding - education only	66.3	51.2
Belize	BLZ	Complementary feeding - supplementary feeding and education	66.3	51.2
Belize	BLZ	Vitamin A supplementation	44.0	34.0

Belize	BLZ	Improved sanitation - Utilization of latrines or toilets	87.9	87.9
Belize	BLZ	Improved water source	98.0	98.0
Belize	BLZ	Water connection in the home	84.1	84.1
Belize	BLZ	Hand washing with soap	90.5	90.5
Belize	BLZ	Hygienic disposal of children's stools	16.4	16.4
Belize	BLZ	Injectable antibiotics for neonatal sepsis	96.4	70.5
Belize	BLZ	ORS - oral rehydration solution	55.2	42.5
Belize	BLZ	Zinc for treatment of diarrhea	9.6	7.4
Belize	BLZ	Oral antibiotics for pneumonia	67.4	51.9
Belize	BLZ	Vitamin A for treatment of measles	44.0	33.9
Belize	BLZ	BCG vaccine	99.0	72.4
Belize	BLZ	Polio vaccine	96.0	70.2
Belize	BLZ	DPT vaccine	96.0	70.2
Belize	BLZ	H. influenzae type b vaccine	96.0	87.7
Belize	BLZ	HepB vaccine	96.0	70.2
Belize	BLZ	Measles vaccine	97.0	70.9
Belize	BLZ	Global wasting (<-2 SD) rate	1.9	2.3
Belize	BLZ	Contraceptive prevalence (CPR)	55.5	45.1
Benin	BEN	TT - Tetanus toxoid vaccination	85.0	62.1
Benin	BEN	IPTp - Intermittent preventive treatment of malaria during pregnancy	33.4	24.4
Benin	BEN	Syphilis detection and treatment	20.9	15.3
Benin	BEN	Iron supplementation in pregnancy	28.6	20.9
Benin	BEN	Hypertensive disorder case management	12.1	8.8
Benin	BEN	Diabetes case management	7.1	5.2
Benin	BEN	Malaria case management	42.7	31.2
Benin	BEN	MgSO4 management of pre-eclampsia	28.5	20.8
Benin	BEN	Thermal protection	77.2	59.4
Benin	BEN	Clean cord care	75.8	58.3
Benin	BEN	Clean birth environment	75.8	58.3
Benin	BEN	Immediate drying and additional stimulation	77.6	59.7
Benin	BEN	Neonatal resuscitation	42.9	33.0
Benin	BEN	Antibiotics for preterm or prolonged PROM	68.9	53.0
Benin	BEN	Parenteral administration of anti-convulsants	44.1	33.9
Benin	BEN	Parenteral administration of uterotonics	75.8	58.3
Benin	BEN	Parenteral administration of antibiotics	68.9	53.0
Benin	BEN	Assisted vaginal delivery	24.0	18.5
Benin	BEN	Manual removal of placenta	64.4	49.6
Benin	BEN	Removal of retained products of conception	49.9	38.4
Benin	BEN	Cesarean delivery	4.3	3.3
Benin	BEN	Blood transfusion	16.4	12.6
Benin	BEN	Induction of labor for pregnancies lasting 41+ weeks	1.5	1.2

Benin	BEN	Complementary feeding - education only	25.3	19.5
Benin	BEN	Complementary feeding - supplementary feeding and education	25.3	19.5
Benin	BEN	Vitamin A supplementation	99.0	76.4
Benin	BEN	Improved sanitation - Utilization of latrines or toilets	16.5	16.5
Benin	BEN	Improved water source	66.4	66.4
Benin	BEN	Water connection in the home	26.4	26.4
Benin	BEN	Hand washing with soap	8.4	8.4
Benin	BEN	Hygienic disposal of children's stools	34.8	34.8
Benin	BEN	ITN/IRS - Households protected from malaria	92.0	71.0
Benin	BEN	Injectable antibiotics for neonatal sepsis	78.1	57.1
Benin	BEN	ORS - oral rehydration solution	22.2	17.1
Benin	BEN	Antibiotics for treatment of dysentery	29.2	22.5
Benin	BEN	Zinc for treatment of diarrhea	17.0	13.1
Benin	BEN	Oral antibiotics for pneumonia	46.1	35.5
Benin	BEN	Vitamin A for treatment of measles	99.0	76.2
Benin	BEN	ACTs- Artemisinin compounds for treatment of malaria	2.5	1.9
Benin	BEN	SAM - treatment for severe acute malnutrition	0.8	0.6
Benin	BEN	BCG vaccine	89.0	65.1
Benin	BEN	Polio vaccine	75.0	54.8
Benin	BEN	DPT vaccine	76.0	55.6
Benin	BEN	H. influenzae type b vaccine	76.0	69.5
Benin	BEN	HepB vaccine	76.0	55.6
Benin	BEN	Pneumococcal vaccine	73.0	66.7
Benin	BEN	Measles vaccine	71.0	51.9
Benin	BEN	Global wasting (<-2 SD) rate	4.5	5.4
Benin	BEN	Contraceptive prevalence (CPR)	17.3	14.1
Bhutan	BTN	Safe abortion services	35.1	28.5
Bhutan	BTN	TT - Tetanus toxoid vaccination	89.0	65.1
Bhutan	BTN	Syphilis detection and treatment	24.0	17.5
Bhutan	BTN	Hypertensive disorder case management	18.6	13.6
Bhutan	BTN	Diabetes case management	14.5	10.6
Bhutan	BTN	Malaria case management	59.9	43.8
Bhutan	BTN	MgSO4 management of pre-eclampsia	36.8	26.9
Bhutan	BTN	Thermal protection	62.4	48.0
Bhutan	BTN	Clean cord care	60.2	46.3
Bhutan	BTN	Clean birth environment	51.8	39.9
Bhutan	BTN	Immediate drying and additional stimulation	57.8	44.5
Bhutan	BTN	Neonatal resuscitation	34.7	26.7
Bhutan	BTN	Antibiotics for preterm or prolonged PROM	47.2	36.3
Bhutan	BTN	Parenteral administration of anti-convulsants	45.1	34.7
Bhutan	BTN	Parenteral administration of uterotonics	56.4	43.4

Bhutan	BTN	Parenteral administration of antibiotics	47.2	36.3
Bhutan	BTN	Assisted vaginal delivery	16.0	12.3
Bhutan	BTN	Manual removal of placenta	23.6	18.2
Bhutan	BTN	Removal of retained products of conception	20.9	16.1
Bhutan	BTN	Cesarean delivery	5.5	4.2
Bhutan	BTN	Blood transfusion	7.9	6.1
Bhutan	BTN	Induction of labor for pregnancies lasting 41+ weeks	1.1	0.8
Bhutan	BTN	Complementary feeding - education only	88.7	68.4
Bhutan	BTN	Complementary feeding - supplementary feeding and education	88.7	68.4
Bhutan	BTN	Vitamin A supplementation	45.0	34.7
Bhutan	BTN	Improved sanitation - Utilization of latrines or toilets	69.3	69.3
Bhutan	BTN	Improved water source	97.2	97.2
Bhutan	BTN	Water connection in the home	96.3	96.3
Bhutan	BTN	Hand washing with soap	79.4	79.4
Bhutan	BTN	Hygienic disposal of children's stools	57.5	57.5
Bhutan	BTN	Injectable antibiotics for neonatal sepsis	63.1	46.1
Bhutan	BTN	ORS - oral rehydration solution	60.9	46.9
Bhutan	BTN	Zinc for treatment of diarrhea	0.8	0.6
Bhutan	BTN	Oral antibiotics for pneumonia	74.2	57.1
Bhutan	BTN	Vitamin A for treatment of measles	45.0	34.6
Bhutan	BTN	BCG vaccine	99.0	72.4
Bhutan	BTN	Polio vaccine	97.0	70.9
Bhutan	BTN	DPT vaccine	97.0	70.9
Bhutan	BTN	H. influenzae type b vaccine	97.0	88.7
Bhutan	BTN	HepB vaccine	97.0	70.9
Bhutan	BTN	Measles vaccine	97.0	70.9
Bhutan	BTN	Global wasting (<-2 SD) rate	6.1	7.3
Bhutan	BTN	Contraceptive prevalence (CPR)	68.35	55.5
Bolivia	BOL	Safe abortion services	0.2	0.2
Bolivia	BOL	TT - Tetanus toxoid vaccination	87.0	63.6
Bolivia	BOL	Syphilis detection and treatment	22.3	16.3
Bolivia	BOL	Iron supplementation in pregnancy	25.1	18.3
Bolivia	BOL	Hypertensive disorder case management	17.2	12.6
Bolivia	BOL	Diabetes case management	13.4	9.8
Bolivia	BOL	Malaria case management	55.4	40.5
Bolivia	BOL	MgSO4 management of pre-eclampsia	34.0	24.9
Bolivia	BOL	Thermal protection	66.7	51.3
Bolivia	BOL	Clean cord care	64.4	49.6
Bolivia	BOL	Clean birth environment	55.4	42.6
Bolivia	BOL	Immediate drying and additional stimulation	61.8	47.6
Bolivia	BOL	Neonatal resuscitation	37.1	28.5

Bolivia	BOL	Antibiotics for preterm or prolonged PROM	50.5	38.9
Bolivia	BOL	Parenteral administration of anti-convulsants	48.3	37.2
Bolivia	BOL	Parenteral administration of uterotonics	60.3	46.4
Bolivia	BOL	Parenteral administration of antibiotics	50.5	38.9
Bolivia	BOL	Assisted vaginal delivery	17.1	13.2
Bolivia	BOL	Manual removal of placenta	25.2	19.4
Bolivia	BOL	Removal of retained products of conception	22.4	17.2
Bolivia	BOL	Cesarean delivery	5.9	4.5
Bolivia	BOL	Blood transfusion	8.5	6.5
Bolivia	BOL	Induction of labor for pregnancies lasting 41+ weeks	1.2	0.9
Bolivia	BOL	Complementary feeding - education only	70.8	54.6
Bolivia	BOL	Complementary feeding - supplementary feeding and education	70.8	54.6
Bolivia	BOL	Vitamin A supplementation	31.0	23.9
Bolivia	BOL	Improved sanitation - Utilization of latrines or toilets	60.7	60.7
Bolivia	BOL	Improved water source	92.8	92.8
Bolivia	BOL	Water connection in the home	65.9	65.9
Bolivia	BOL	Hygienic disposal of children's stools	16.6	16.6
Bolivia	BOL	Injectable antibiotics for neonatal sepsis	67.5	49.3
Bolivia	BOL	ORS - oral rehydration solution	34.9	26.9
Bolivia	BOL	Antibiotics for treatment of dysentery	43.9	33.8
Bolivia	BOL	Vitamin A for treatment of measles	31.0	23.9
Bolivia	BOL	BCG vaccine	90.0	65.8
Bolivia	BOL	Polio vaccine	83.0	60.7
Bolivia	BOL	DPT vaccine	83.0	60.7
Bolivia	BOL	H. influenzae type b vaccine	83.0	75.9
Bolivia	BOL	HepB vaccine	83.0	60.7
Bolivia	BOL	Pneumococcal vaccine	83.0	75.9
Bolivia	BOL	Rotavirus vaccine	87.0	79.5
Bolivia	BOL	Measles vaccine	89.0	65.1
Bolivia	BOL	Global wasting (<-2 SD) rate	1.4	1.7
Bolivia	BOL	Contraceptive prevalence (CPR)	66.1	53.7
Bosnia and Herzegovina	BIH	Safe abortion services	100.0	81.2
Bosnia and Herzegovina	BIH	Syphilis detection and treatment	21.5	15.7
Bosnia and Herzegovina	BIH	Hypertensive disorder case management	20.2	14.8
Bosnia and Herzegovina	BIH	Diabetes case management	15.8	11.6
Bosnia and Herzegovina	BIH	Malaria case management	65.3	47.7
Bosnia and Herzegovina	BIH	MgSO4 management of pre-eclampsia	40.0	29.2

Bosnia and Herzegovina	BIH	Thermal protection	98.6	75.9
Bosnia and Herzegovina	BIH	Clean cord care	95.2	73.3
Bosnia and Herzegovina	BIH	Clean birth environment	81.8	62.9
Bosnia and Herzegovina	BIH	Immediate drying and additional stimulation	91.3	70.3
Bosnia and Herzegovina	BIH	Neonatal resuscitation	54.8	42.2
Bosnia and Herzegovina	BIH	Antibiotics for preterm or prolonged PROM	74.6	57.4
Bosnia and Herzegovina	BIH	Parenteral administration of anti-convulsants	71.3	54.9
Bosnia and Herzegovina	BIH	Parenteral administration of uterotonics	89.1	68.6
Bosnia and Herzegovina	BIH	Parenteral administration of antibiotics	74.6	57.4
Bosnia and Herzegovina	BIH	Assisted vaginal delivery	25.2	19.4
Bosnia and Herzegovina	BIH	Manual removal of placenta	37.3	28.7
Bosnia and Herzegovina	BIH	Removal of retained products of conception	33.0	25.4
Bosnia and Herzegovina	BIH	Cesarean delivery	8.7	6.7
Bosnia and Herzegovina	BIH	Blood transfusion	12.6	9.7
Bosnia and Herzegovina	BIH	Induction of labor for pregnancies lasting 41+ weeks	1.8	1.4
Bosnia and Herzegovina	BIH	Improved sanitation - Utilization of latrines or toilets	95.4	95.4
Bosnia and Herzegovina	BIH	Improved water source	96.1	96.1
Bosnia and Herzegovina	BIH	Water connection in the home	91.5	91.5
Bosnia and Herzegovina	BIH	Hand washing with soap	97.0	97.0
Bosnia and Herzegovina	BIH	Hygienic disposal of children's stools	19.6	19.6
Bosnia and Herzegovina	BIH	Injectable antibiotics for neonatal sepsis	99.7	72.9
Bosnia and Herzegovina	BIH	ORS - oral rehydration solution	36.3	27.9
Bosnia and Herzegovina	BIH	Antibiotics for treatment of dysentery	2.9	2.2
Bosnia and Herzegovina	BIH	Oral antibiotics for pneumonia	86.9	66.9
Bosnia and Herzegovina	BIH	BCG vaccine	95.0	69.4
Bosnia and Herzegovina	BIH	Polio vaccine	73.0	53.4
Bosnia and Herzegovina	BIH	DPT vaccine	73.0	53.4

Bosnia and Herzegovina	BIH	H. influenzae type b vaccine	62.0	56.7
Bosnia and Herzegovina	BIH	HepB vaccine	80.0	58.5
Bosnia and Herzegovina	BIH	Measles vaccine	68.0	49.7
Bosnia and Herzegovina	BIH	Global wasting (<-2 SD) rate	2.4	2.8
Bosnia and Herzegovina	BIH	Contraceptive prevalence (CPR)	48.6	39.5
Botswana	BWA	Safe abortion services	42.4	34.4
Botswana	BWA	TT - Tetanus toxoid vaccination	93.0	68.0
Botswana	BWA	Syphilis detection and treatment	18.1	13.2
Botswana	BWA	Hypertensive disorder case management	17.6	12.9
Botswana	BWA	Diabetes case management	13.7	10.0
Botswana	BWA	Malaria case management	56.8	41.5
Botswana	BWA	MgSO4 management of pre-eclampsia	34.8	25.4
Botswana	BWA	Thermal protection	92.7	71.3
Botswana	BWA	Clean cord care	89.5	68.9
Botswana	BWA	Clean birth environment	76.9	59.2
Botswana	BWA	Immediate drying and additional stimulation	85.9	66.1
Botswana	BWA	Neonatal resuscitation	51.6	39.7
Botswana	BWA	Antibiotics for preterm or prolonged PROM	70.2	54.0
Botswana	BWA	Parenteral administration of anti-convulsants	67.1	51.6
Botswana	BWA	Parenteral administration of uterotonics	83.8	64.5
Botswana	BWA	Parenteral administration of antibiotics	70.2	54.0
Botswana	BWA	Assisted vaginal delivery	23.7	18.2
Botswana	BWA	Manual removal of placenta	35.1	27.0
Botswana	BWA	Removal of retained products of conception	31.1	23.9
Botswana	BWA	Cesarean delivery	8.2	6.3
Botswana	BWA	Blood transfusion	11.8	9.1
Botswana	BWA	Induction of labor for pregnancies lasting 41+ weeks	1.7	1.3
Botswana	BWA	Complementary feeding - education only	45.5	35.1
Botswana	BWA	Complementary feeding - supplementary feeding and education	45.5	35.1
Botswana	BWA	Vitamin A supplementation	83.0	64.0
Botswana	BWA	Improved sanitation - Utilization of latrines or toilets	77.3	77.3
Botswana	BWA	Improved water source	90.3	90.3
Botswana	BWA	Water connection in the home	81.7	81.7
Botswana	BWA	Injectable antibiotics for neonatal sepsis	93.8	68.6
Botswana	BWA	ORS - oral rehydration solution	42.9	33.0
Botswana	BWA	Oral antibiotics for pneumonia	14.0	10.8
Botswana	BWA	Vitamin A for treatment of measles	83.0	63.9
Botswana	BWA	BCG vaccine	98.0	71.6

Botswana	BWA	Polio vaccine	96.0	70.2
Botswana	BWA	DPT vaccine	95.0	69.4
Botswana	BWA	H. influenzae type b vaccine	95.0	86.8
Botswana	BWA	HepB vaccine	95.0	69.4
Botswana	BWA	Pneumococcal vaccine	91.0	83.2
Botswana	BWA	Rotavirus vaccine	87.0	79.5
Botswana	BWA	Measles vaccine	97.0	70.9
Botswana	BWA	Global wasting (<-2 SD) rate	4.9	5.8
Botswana	BWA	Contraceptive prevalence (CPR)	61.45	49.9
Brazil	BRA	Safe abortion services	0.2	0.2
Brazil	BRA	TT - Tetanus toxoid vaccination	94.0	68.7
Brazil	BRA	Syphilis detection and treatment	22.5	16.4
Brazil	BRA	Hypertensive disorder case management	21.8	15.9
Brazil	BRA	Diabetes case management	17.0	12.4
Brazil	BRA	Malaria case management	70.5	51.5
Brazil	BRA	MgSO4 management of pre-eclampsia	43.2	31.6
Brazil	BRA	Thermal protection	97.0	74.6
Brazil	BRA	Clean cord care	93.6	72.0
Brazil	BRA	Clean birth environment	80.5	61.9
Brazil	BRA	Immediate drying and additional stimulation	89.8	69.1
Brazil	BRA	Neonatal resuscitation	53.9	41.5
Brazil	BRA	Antibiotics for preterm or prolonged PROM	73.4	56.5
Brazil	BRA	Parenteral administration of anti-convulsants	70.1	53.9
Brazil	BRA	Parenteral administration of uterotonics	87.7	67.5
Brazil	BRA	Parenteral administration of antibiotics	73.4	56.5
Brazil	BRA	Assisted vaginal delivery	24.8	19.1
Brazil	BRA	Manual removal of placenta	36.7	28.2
Brazil	BRA	Removal of retained products of conception	32.5	25.0
Brazil	BRA	Cesarean delivery	8.5	6.5
Brazil	BRA	Blood transfusion	12.4	9.5
Brazil	BRA	Induction of labor for pregnancies lasting 41+ weeks	1.7	1.3
Brazil	BRA	Complementary feeding - education only	56.7	43.8
Brazil	BRA	Complementary feeding - supplementary feeding and education	56.7	43.8
Brazil	BRA	Improved sanitation - Utilization of latrines or toilets	88.3	88.3
Brazil	BRA	Improved water source	98.2	98.2
Brazil	BRA	Water connection in the home	95.5	95.5
Brazil	BRA	Injectable antibiotics for neonatal sepsis	98.1	71.7
Brazil	BRA	ORS - oral rehydration solution	43.6	33.6
Brazil	BRA	Antibiotics for treatment of dysentery	7.9	6.1
Brazil	BRA	Oral antibiotics for pneumonia	49.7	38.2
Brazil	BRA	BCG vaccine	90.0	65.8

Brazil	BRA	Polio vaccine	85.0	62.1
Brazil	BRA	DPT vaccine	83.0	60.7
Brazil	BRA	H. influenzae type b vaccine	83.0	75.9
Brazil	BRA	HepB vaccine	83.0	60.7
Brazil	BRA	Pneumococcal vaccine	84.0	76.8
Brazil	BRA	Rotavirus vaccine	80.0	73.1
Brazil	BRA	Meningococcal A	84.0	61.4
Brazil	BRA	Measles vaccine	84.0	61.4
Brazil	BRA	Global wasting (<-2 SD) rate	2.7	3.2
Brazil	BRA	Contraceptive prevalence (CPR)	79.9	64.9
Burkina Faso	BFA	TT - Tetanus toxoid vaccination	92.0	67.3
Burkina Faso	BFA	IPTp - Intermittent preventive treatment of malaria during pregnancy	47.6	34.8
Burkina Faso	BFA	Syphilis detection and treatment	8.4	6.1
Burkina Faso	BFA	Iron supplementation in pregnancy	50.2	36.7
Burkina Faso	BFA	Hypertensive disorder case management	6.4	4.7
Burkina Faso	BFA	Diabetes case management	0.7	0.5
Burkina Faso	BFA	Malaria case management	22.6	16.5
Burkina Faso	BFA	MgSO4 management of pre-eclampsia	6.9	5.0
Burkina Faso	BFA	Thermal protection	65.6	50.5
Burkina Faso	BFA	Clean cord care	65.5	50.4
Burkina Faso	BFA	Clean birth environment	64.0	49.2
Burkina Faso	BFA	Immediate drying and additional stimulation	63.9	49.2
Burkina Faso	BFA	Neonatal resuscitation	13.0	10.0
Burkina Faso	BFA	Antibiotics for preterm or prolonged PROM	52.6	40.5
Burkina Faso	BFA	Parenteral administration of anti-convulsants	15.9	12.2
Burkina Faso	BFA	Parenteral administration of uterotonics	64.1	49.3
Burkina Faso	BFA	Parenteral administration of antibiotics	52.6	40.5
Burkina Faso	BFA	Assisted vaginal delivery	7.0	5.4
Burkina Faso	BFA	Manual removal of placenta	49.1	37.8
Burkina Faso	BFA	Removal of retained products of conception	11.1	8.5
Burkina Faso	BFA	Cesarean delivery	0.8	0.6
Burkina Faso	BFA	Blood transfusion	5.8	4.5
Burkina Faso	BFA	Induction of labor for pregnancies lasting 41+ weeks	0.9	0.7
Burkina Faso	BFA	Complementary feeding - education only	5.9	4.6
Burkina Faso	BFA	Complementary feeding - supplementary feeding and education	5.9	4.6
Burkina Faso	BFA	Improved sanitation - Utilization of latrines or toilets	19.4	19.4
Burkina Faso	BFA	Improved water source	47.9	47.9
Burkina Faso	BFA	Water connection in the home	12.2	12.2
Burkina Faso	BFA	Hand washing with soap	12.8	12.8
Burkina Faso	BFA	Hygienic disposal of children's stools	20.8	20.8
Burkina Faso	BFA	ITN/IRS - Households protected from malaria	75.3	58.1

Burkina Faso	BFA	Injectable antibiotics for neonatal sepsis	66.3	48.5
Burkina Faso	BFA	ORS - oral rehydration solution	21.2	16.3
Burkina Faso	BFA	Antibiotics for treatment of dysentery	34.5	26.5
Burkina Faso	BFA	Zinc for treatment of diarrhea	0.4	0.3
Burkina Faso	BFA	Oral antibiotics for pneumonia	55.5	42.7
Burkina Faso	BFA	ACTs- Artemisinin compounds for treatment of malaria	5.5	4.2
Burkina Faso	BFA	SAM - treatment for severe acute malnutrition	26.1	20.1
Burkina Faso	BFA	BCG vaccine	98.0	71.6
Burkina Faso	BFA	Polio vaccine	91.0	66.5
Burkina Faso	BFA	DPT vaccine	91.0	66.5
Burkina Faso	BFA	H. influenzae type b vaccine	91.0	83.2
Burkina Faso	BFA	HepB vaccine	91.0	66.5
Burkina Faso	BFA	Pneumococcal vaccine	91.0	83.2
Burkina Faso	BFA	Rotavirus vaccine	91.0	83.2
Burkina Faso	BFA	Meningococcal A	87.0	63.6
Burkina Faso	BFA	Measles vaccine	88.0	64.3
Burkina Faso	BFA	Global wasting (<-2 SD) rate	15.5	18.6
Burkina Faso	BFA	Contraceptive prevalence (CPR)	27.95	22.7
Burundi	BDI	Safe abortion services	3.3	2.7
Burundi	BDI	TT - Tetanus toxoid vaccination	87.0	63.6
Burundi	BDI	IPTp - Intermittent preventive treatment of malaria during pregnancy	20.7	15.1
Burundi	BDI	Syphilis detection and treatment	24.5	17.9
Burundi	BDI	Iron supplementation in pregnancy	1.4	1.0
Burundi	BDI	Hypertensive disorder case management	12.2	8.9
Burundi	BDI	Diabetes case management	9.5	6.9
Burundi	BDI	Malaria case management	39.3	28.7
Burundi	BDI	MgSO4 management of pre-eclampsia	24.1	17.6
Burundi	BDI	Thermal protection	83.0	63.9
Burundi	BDI	Clean cord care	80.1	61.6
Burundi	BDI	Clean birth environment	68.8	52.9
Burundi	BDI	Immediate drying and additional stimulation	76.9	59.2
Burundi	BDI	Neonatal resuscitation	46.2	35.6
Burundi	BDI	Antibiotics for preterm or prolonged PROM	62.8	48.3
Burundi	BDI	Parenteral administration of anti-convulsants	60.0	46.2
Burundi	BDI	Parenteral administration of uterotonics	75.0	57.7
Burundi	BDI	Parenteral administration of antibiotics	62.8	48.3
Burundi	BDI	Assisted vaginal delivery	21.2	16.3
Burundi	BDI	Manual removal of placenta	31.4	24.2
Burundi	BDI	Removal of retained products of conception	27.8	21.4
Burundi	BDI	Cesarean delivery	7.3	5.6
Burundi	BDI	Blood transfusion	10.6	8.2

Burundi	BDI	Induction of labor for pregnancies lasting 41+ weeks	1.5	1.2
Burundi	BDI	Complementary feeding - education only	19.3	14.9
Burundi	BDI	Complementary feeding - supplementary feeding and education	19.3	14.9
Burundi	BDI	Vitamin A supplementation	79.0	61.0
Burundi	BDI	Improved sanitation - Utilization of latrines or toilets	45.8	45.8
Burundi	BDI	Improved water source	60.8	60.8
Burundi	BDI	Water connection in the home	21.6	21.6
Burundi	BDI	Hand washing with soap	5.8	5.8
Burundi	BDI	Hygienic disposal of children's stools	73.7	73.7
Burundi	BDI	ITN/IRS - Households protected from malaria	46.8	36.1
Burundi	BDI	Injectable antibiotics for neonatal sepsis	83.9	61.3
Burundi	BDI	ORS - oral rehydration solution	35.6	27.4
Burundi	BDI	Antibiotics for treatment of dysentery	32.9	25.3
Burundi	BDI	Zinc for treatment of diarrhea	15.0	11.5
Burundi	BDI	Oral antibiotics for pneumonia	58.5	45.0
Burundi	BDI	Vitamin A for treatment of measles	79.0	60.8
Burundi	BDI	ACTs- Artemisinin compounds for treatment of malaria	5.0	3.8
Burundi	BDI	SAM - treatment for severe acute malnutrition	6.9	5.3
Burundi	BDI	BCG vaccine	91.0	66.5
Burundi	BDI	Polio vaccine	90.0	65.8
Burundi	BDI	DPT vaccine	90.0	65.8
Burundi	BDI	H. influenzae type b vaccine	90.0	82.3
Burundi	BDI	HepB vaccine	90.0	65.8
Burundi	BDI	Pneumococcal vaccine	90.0	82.3
Burundi	BDI	Rotavirus vaccine	92.0	84.1
Burundi	BDI	Measles vaccine	88.0	64.3
Burundi	BDI	Global wasting (<-2 SD) rate	5.0	6.0
Burundi	BDI	Contraceptive prevalence (CPR)	31.85	25.9
Cambodia	KHM	Safe abortion services	39.0	31.7
Cambodia	KHM	TT - Tetanus toxoid vaccination	93.0	68.0
Cambodia	KHM	Syphilis detection and treatment	23.7	17.3
Cambodia	KHM	Iron supplementation in pregnancy	75.5	55.2
Cambodia	KHM	Hypertensive disorder case management	18.2	13.3
Cambodia	KHM	Diabetes case management	14.2	10.4
Cambodia	KHM	Malaria case management	58.7	42.9
Cambodia	KHM	MgSO4 management of pre-eclampsia	36.0	26.3
Cambodia	KHM	Thermal protection	82.3	63.3
Cambodia	KHM	Clean cord care	79.4	61.1
Cambodia	KHM	Clean birth environment	68.3	52.6
Cambodia	KHM	Immediate drying and additional stimulation	76.2	58.6
Cambodia	KHM	Neonatal resuscitation	45.8	35.2

Cambodia	KHM	Antibiotics for preterm or prolonged PROM	62.3	47.9
Cambodia	KHM	Parenteral administration of anti-convulsants	59.5	45.8
Cambodia	KHM	Parenteral administration of uterotonics	74.4	57.3
Cambodia	KHM	Parenteral administration of antibiotics	62.3	47.9
Cambodia	KHM	Assisted vaginal delivery	21.0	16.2
Cambodia	KHM	Manual removal of placenta	31.1	23.9
Cambodia	KHM	Removal of retained products of conception	27.6	21.2
Cambodia	KHM	Cesarean delivery	7.2	5.5
Cambodia	KHM	Blood transfusion	10.5	8.1
Cambodia	KHM	Induction of labor for pregnancies lasting 41+ weeks	1.5	1.2
Cambodia	KHM	Complementary feeding - education only	47.7	36.8
Cambodia	KHM	Complementary feeding - supplementary feeding and education	47.7	36.8
Cambodia	KHM	Vitamin A supplementation	73.0	56.3
Cambodia	KHM	Improved sanitation - Utilization of latrines or toilets	59.2	59.2
Cambodia	KHM	Improved water source	78.5	78.5
Cambodia	KHM	Water connection in the home	20.5	20.5
Cambodia	KHM	Hand washing with soap	67.9	67.9
Cambodia	KHM	Hygienic disposal of children's stools	22.6	22.6
Cambodia	KHM	ITN/IRS - Households protected from malaria	4.5	3.5
Cambodia	KHM	Injectable antibiotics for neonatal sepsis	83.2	60.8
Cambodia	KHM	ORS - oral rehydration solution	35.2	27.1
Cambodia	KHM	Antibiotics for treatment of dysentery	7.0	5.4
Cambodia	KHM	Zinc for treatment of diarrhea	5.4	4.2
Cambodia	KHM	Oral antibiotics for pneumonia	68.8	52.9
Cambodia	KHM	Vitamin A for treatment of measles	73.0	56.2
Cambodia	KHM	SAM - treatment for severe acute malnutrition	2.5	1.9
Cambodia	KHM	BCG vaccine	93.0	68.0
Cambodia	KHM	Polio vaccine	90.0	65.8
Cambodia	KHM	DPT vaccine	92.0	67.3
Cambodia	KHM	H. influenzae type b vaccine	92.0	84.1
Cambodia	KHM	HepB vaccine	92.0	67.3
Cambodia	KHM	Pneumococcal vaccine	84.0	76.8
Cambodia	KHM	Measles vaccine	84.0	61.4
Cambodia	KHM	Global wasting (<-2 SD) rate	9.9	11.8
Cambodia	KHM	Contraceptive prevalence (CPR)	61.2	49.7
Cameroon	CMR	TT - Tetanus toxoid vaccination	85.0	62.1
Cameroon	CMR	IPTp - Intermittent preventive treatment of malaria during pregnancy	44.3	32.4
Cameroon	CMR	Syphilis detection and treatment	20.5	15.0
Cameroon	CMR	Iron supplementation in pregnancy	54.1	39.5
Cameroon	CMR	Hypertensive disorder case management	14.1	10.3
Cameroon	CMR	Diabetes case management	11.0	8.0

Cameroon	CMR	Malaria case management	45.6	33.3
Cameroon	CMR	MgSO4 management of pre-eclampsia	28.0	20.5
Cameroon	CMR	Thermal protection	60.5	46.6
Cameroon	CMR	Clean cord care	58.5	45.0
Cameroon	CMR	Clean birth environment	50.2	38.6
Cameroon	CMR	Immediate drying and additional stimulation	56.1	43.2
Cameroon	CMR	Neonatal resuscitation	33.7	25.9
Cameroon	CMR	Antibiotics for preterm or prolonged PROM	45.8	35.2
Cameroon	CMR	Parenteral administration of anti-convulsants	43.8	33.7
Cameroon	CMR	Parenteral administration of uterotonics	54.7	42.1
Cameroon	CMR	Parenteral administration of antibiotics	45.8	35.2
Cameroon	CMR	Assisted vaginal delivery	15.5	11.9
Cameroon	CMR	Manual removal of placenta	22.9	17.6
Cameroon	CMR	Removal of retained products of conception	20.3	15.6
Cameroon	CMR	Cesarean delivery	5.3	4.1
Cameroon	CMR	Blood transfusion	7.7	5.9
Cameroon	CMR	Induction of labor for pregnancies lasting 41+ weeks	1.1	0.8
Cameroon	CMR	Complementary feeding - education only	32.9	25.4
Cameroon	CMR	Complementary feeding - supplementary feeding and education	32.9	25.4
Cameroon	CMR	Vitamin A supplementation	9.0	6.9
Cameroon	CMR	Improved sanitation - Utilization of latrines or toilets	39.1	39.1
Cameroon	CMR	Improved water source	60.4	60.4
Cameroon	CMR	Water connection in the home	24.0	24.0
Cameroon	CMR	Hand washing with soap	14.9	14.9
Cameroon	CMR	Hygienic disposal of children's stools	70.6	70.6
Cameroon	CMR	ITN/IRS - Households protected from malaria	70.9	54.7
Cameroon	CMR	Injectable antibiotics for neonatal sepsis	61.3	44.8
Cameroon	CMR	ORS - oral rehydration solution	15.8	12.2
Cameroon	CMR	Antibiotics for treatment of dysentery	15.0	11.5
Cameroon	CMR	Zinc for treatment of diarrhea	12.0	9.2
Cameroon	CMR	Oral antibiotics for pneumonia	26.7	20.5
Cameroon	CMR	Vitamin A for treatment of measles	9.0	6.9
Cameroon	CMR	ACTs- Artemisinin compounds for treatment of malaria	3.6	2.8
Cameroon	CMR	SAM - treatment for severe acute malnutrition	11.3	8.7
Cameroon	CMR	BCG vaccine	88.0	64.3
Cameroon	CMR	Polio vaccine	78.0	57.0
Cameroon	CMR	DPT vaccine	79.0	57.8
Cameroon	CMR	H. influenzae type b vaccine	79.0	72.2
Cameroon	CMR	HepB vaccine	79.0	57.8
Cameroon	CMR	Pneumococcal vaccine	79.0	72.2
Cameroon	CMR	Rotavirus vaccine	78.0	71.3

Cameroon	CMR	Measles vaccine	71.0	51.9
Cameroon	CMR	Global wasting (<-2 SD) rate	5.0	6.0
Cameroon	CMR	Contraceptive prevalence (CPR)	37.95	30.8
Cape Verde	CPV	TT - Tetanus toxoid vaccination	92.0	67.3
Cape Verde	CPV	Syphilis detection and treatment	22.3	16.3
Cape Verde	CPV	Iron supplementation in pregnancy	10.2	7.5
Cape Verde	CPV	Hypertensive disorder case management	17.4	12.7
Cape Verde	CPV	Diabetes case management	13.5	9.9
Cape Verde	CPV	Malaria case management	56.0	40.9
Cape Verde	CPV	MgSO4 management of pre-eclampsia	34.4	25.1
Cape Verde	CPV	Thermal protection	76.6	58.9
Cape Verde	CPV	Clean cord care	74.0	56.9
Cape Verde	CPV	Clean birth environment	63.6	48.9
Cape Verde	CPV	Immediate drying and additional stimulation	71.0	54.6
Cape Verde	CPV	Neonatal resuscitation	42.6	32.8
Cape Verde	CPV	Antibiotics for preterm or prolonged PROM	58.0	44.6
Cape Verde	CPV	Parenteral administration of anti-convulsants	55.4	42.6
Cape Verde	CPV	Parenteral administration of uterotonics	69.3	53.3
Cape Verde	CPV	Parenteral administration of antibiotics	58.0	44.6
Cape Verde	CPV	Assisted vaginal delivery	19.6	15.1
Cape Verde	CPV	Manual removal of placenta	29.0	22.3
Cape Verde	CPV	Removal of retained products of conception	25.7	19.8
Cape Verde	CPV	Cesarean delivery	6.7	5.2
Cape Verde	CPV	Blood transfusion	9.8	7.5
Cape Verde	CPV	Induction of labor for pregnancies lasting 41+ weeks	1.4	1.1
Cape Verde	CPV	Improved sanitation - Utilization of latrines or toilets	73.9	73.9
Cape Verde	CPV	Improved water source	87.1	87.1
Cape Verde	CPV	Water connection in the home	77.1	77.1
Cape Verde	CPV	Injectable antibiotics for neonatal sepsis	77.5	56.7
Cape Verde	CPV	ORS - oral rehydration solution	99.8	76.8
Cape Verde	CPV	BCG vaccine	96.0	70.2
Cape Verde	CPV	Polio vaccine	98.0	71.6
Cape Verde	CPV	DPT vaccine	98.0	71.6
Cape Verde	CPV	H. influenzae type b vaccine	99.0	90.5
Cape Verde	CPV	HepB vaccine	99.0	72.4
Cape Verde	CPV	Measles vaccine	99.0	72.4
Cape Verde	CPV	Global wasting (<-2 SD) rate	10.0	12.0
Cape Verde	CPV	Contraceptive prevalence (CPR)	66.5	54.0
Central African Republic	CAF	TT - Tetanus toxoid vaccination	60.0	43.9
Central African Republic	CAF	IPTp - Intermittent preventive treatment of malaria during pregnancy	26.4	19.3

Central African Republic	CAF	Syphilis detection and treatment	16.9	12.4
Central African Republic	CAF	Hypertensive disorder case management	9.2	6.7
Central African Republic	CAF	Diabetes case management	7.1	5.2
Central African Republic	CAF	Malaria case management	29.6	21.6
Central African Republic	CAF	MgSO4 management of pre-eclampsia	18.1	13.2
Central African Republic	CAF	Thermal protection	51.5	39.6
Central African Republic	CAF	Clean cord care	49.7	38.2
Central African Republic	CAF	Clean birth environment	42.7	32.9
Central African Republic	CAF	Immediate drying and additional stimulation	47.7	36.7
Central African Republic	CAF	Neonatal resuscitation	28.6	22.0
Central African Republic	CAF	Antibiotics for preterm or prolonged PROM	39.0	30.0
Central African Republic	CAF	Parenteral administration of anti-convulsants	37.2	28.6
Central African Republic	CAF	Parenteral administration of uterotonics	46.5	35.8
Central African Republic	CAF	Parenteral administration of antibiotics	39.0	30.0
Central African Republic	CAF	Assisted vaginal delivery	13.2	10.2
Central African Republic	CAF	Manual removal of placenta	19.5	15.0
Central African Republic	CAF	Removal of retained products of conception	17.3	13.3
Central African Republic	CAF	Cesarean delivery	4.5	3.5
Central African Republic	CAF	Blood transfusion	6.6	5.1
Central African Republic	CAF	Induction of labor for pregnancies lasting 41+ weeks	0.9	0.7
Central African Republic	CAF	Complementary feeding - education only	33.9	26.2
Central African Republic	CAF	Complementary feeding - supplementary feeding and education	33.9	26.2
Central African Republic	CAF	Improved sanitation - Utilization of latrines or toilets	25.3	25.3
Central African Republic	CAF	Improved water source	46.3	46.3
Central African Republic	CAF	Water connection in the home	8.4	8.4
Central African Republic	CAF	Hand washing with soap	15.4	15.4
Central African Republic	CAF	Hygienic disposal of children's stools	50.2	50.2

Central African Republic	CAF	ITN/IRS - Households protected from malaria	47.2	36.4
Central African Republic	CAF	Injectable antibiotics for neonatal sepsis	52.1	38.1
Central African Republic	CAF	ORS - oral rehydration solution	15.6	12.0
Central African Republic	CAF	Antibiotics for treatment of dysentery	4.4	3.4
Central African Republic	CAF	Zinc for treatment of diarrhea	0.5	0.4
Central African Republic	CAF	Oral antibiotics for pneumonia	29.8	22.9
Central African Republic	CAF	ACTs- Artemisinin compounds for treatment of malaria	2.3	1.8
Central African Republic	CAF	SAM - treatment for severe acute malnutrition	17.6	13.5
Central African Republic	CAF	BCG vaccine	74.0	54.1
Central African Republic	CAF	Polio vaccine	47.0	34.4
Central African Republic	CAF	DPT vaccine	47.0	34.4
Central African Republic	CAF	H. influenzae type b vaccine	47.0	43.0
Central African Republic	CAF	HepB vaccine	47.0	34.4
Central African Republic	CAF	Pneumococcal vaccine	47.0	43.0
Central African Republic	CAF	Meningococcal A	66.0	48.2
Central African Republic	CAF	Measles vaccine	49.0	35.8
Central African Republic	CAF	Global wasting (<-2 SD) rate	7.3	8.7
Central African Republic	CAF	Contraceptive prevalence (CPR)	23.5	19.1
Chad	TCD	TT - Tetanus toxoid vaccination	78.0	57.0
Chad	TCD	IPTp - Intermittent preventive treatment of malaria during pregnancy	18.1	13.2
Chad	TCD	Syphilis detection and treatment	13.7	10.0
Chad	TCD	Iron supplementation in pregnancy	11.0	8.0
Chad	TCD	Hypertensive disorder case management	7.5	5.5
Chad	TCD	Diabetes case management	5.9	4.3
Chad	TCD	Malaria case management	24.3	17.8
Chad	TCD	MgSO4 management of pre-eclampsia	14.9	10.9
Chad	TCD	Thermal protection	21.6	16.6
Chad	TCD	Clean cord care	20.9	16.1
Chad	TCD	Clean birth environment	17.9	13.8
Chad	TCD	Immediate drying and additional stimulation	20.0	15.4
Chad	TCD	Neonatal resuscitation	12.0	9.2
Chad	TCD	Antibiotics for preterm or prolonged PROM	16.4	12.6

Chad	TCD	Parenteral administration of anti-convulsants	15.6	12.0
Chad	TCD	Parenteral administration of uterotonics	19.5	15.0
Chad	TCD	Parenteral administration of antibiotics	16.4	12.6
Chad	TCD	Assisted vaginal delivery	5.5	4.2
Chad	TCD	Manual removal of placenta	8.2	6.3
Chad	TCD	Removal of retained products of conception	7.2	5.5
Chad	TCD	Cesarean delivery	1.9	1.5
Chad	TCD	Blood transfusion	2.8	2.2
Chad	TCD	Induction of labor for pregnancies lasting 41+ weeks	0.4	0.3
Chad	TCD	Complementary feeding - education only	10.4	8.0
Chad	TCD	Complementary feeding - supplementary feeding and education	10.4	8.0
Chad	TCD	Vitamin A supplementation	67.0	51.7
Chad	TCD	Improved sanitation - Utilization of latrines or toilets	8.3	8.3
Chad	TCD	Improved water source	38.7	38.7
Chad	TCD	Water connection in the home	7.4	7.4
Chad	TCD	Hand washing with soap	23.7	23.7
Chad	TCD	Hygienic disposal of children's stools	18.9	18.9
Chad	TCD	ITN/IRS - Households protected from malaria	77.3	59.6
Chad	TCD	Injectable antibiotics for neonatal sepsis	21.9	16.0
Chad	TCD	ORS - oral rehydration solution	20.4	15.7
Chad	TCD	Antibiotics for treatment of dysentery	15.9	12.2
Chad	TCD	Zinc for treatment of diarrhea	1.3	1.0
Chad	TCD	Oral antibiotics for pneumonia	25.8	19.9
Chad	TCD	Vitamin A for treatment of measles	67.0	51.6
Chad	TCD	ACTs- Artemisinin compounds for treatment of malaria	1.7	1.3
Chad	TCD	SAM - treatment for severe acute malnutrition	18.3	14.1
Chad	TCD	BCG vaccine	59.0	43.1
Chad	TCD	Polio vaccine	44.0	32.2
Chad	TCD	DPT vaccine	41.0	30.0
Chad	TCD	H. influenzae type b vaccine	41.0	37.5
Chad	TCD	HepB vaccine	41.0	30.0
Chad	TCD	Meningococcal A	70.0	51.2
Chad	TCD	Measles vaccine	37.0	27.0
Chad	TCD	Global wasting (<-2 SD) rate	13.5	16.2
Chad	TCD	Contraceptive prevalence (CPR)	7.25	5.9
Colombia	COL	Safe abortion services	0.2	0.2
Colombia	COL	TT - Tetanus toxoid vaccination	95.0	69.4
Colombia	COL	Syphilis detection and treatment	24.0	17.5
Colombia	COL	Iron supplementation in pregnancy	80.7	59.0
Colombia	COL	Hypertensive disorder case management	21.5	15.7
Colombia	COL	Diabetes case management	16.8	12.3

Colombia	COL	Malaria case management	69.5	50.8
Colombia	COL	MgSO4 management of pre-eclampsia	42.6	31.1
Colombia	COL	Thermal protection	95.2	73.3
Colombia	COL	Clean cord care	91.9	70.7
Colombia	COL	Clean birth environment	79.0	60.8
Colombia	COL	Immediate drying and additional stimulation	88.2	67.9
Colombia	COL	Neonatal resuscitation	53.0	40.8
Colombia	COL	Antibiotics for preterm or prolonged PROM	72.1	55.5
Colombia	COL	Parenteral administration of anti-convulsants	68.9	53.0
Colombia	COL	Parenteral administration of uterotonics	86.1	66.3
Colombia	COL	Parenteral administration of antibiotics	72.1	55.5
Colombia	COL	Assisted vaginal delivery	24.3	18.7
Colombia	COL	Manual removal of placenta	36.0	27.7
Colombia	COL	Removal of retained products of conception	31.9	24.5
Colombia	COL	Cesarean delivery	8.4	6.5
Colombia	COL	Blood transfusion	12.1	9.3
Colombia	COL	Induction of labor for pregnancies lasting 41+ weeks	1.7	1.3
Colombia	COL	Complementary feeding - education only	72.3	55.8
Colombia	COL	Complementary feeding - supplementary feeding and education	72.3	55.8
Colombia	COL	Improved sanitation - Utilization of latrines or toilets	89.6	89.6
Colombia	COL	Improved water source	97.3	97.3
Colombia	COL	Water connection in the home	86.3	86.3
Colombia	COL	Hygienic disposal of children's stools	14.4	14.4
Colombia	COL	Injectable antibiotics for neonatal sepsis	96.3	70.4
Colombia	COL	ORS - oral rehydration solution	54.1	41.6
Colombia	COL	Antibiotics for treatment of dysentery	5.8	4.5
Colombia	COL	SAM - treatment for severe acute malnutrition	0.1	0.1
Colombia	COL	BCG vaccine	89.0	65.1
Colombia	COL	Polio vaccine	92.0	67.3
Colombia	COL	DPT vaccine	92.0	67.3
Colombia	COL	H. influenzae type b vaccine	92.0	84.1
Colombia	COL	HepB vaccine	92.0	67.3
Colombia	COL	Pneumococcal vaccine	94.0	85.9
Colombia	COL	Rotavirus vaccine	90.0	82.3
Colombia	COL	Measles vaccine	95.0	69.4
Colombia	COL	Global wasting (<-2 SD) rate	0.9	1.1
Colombia	COL	Contraceptive prevalence (CPR)	81.4	66.1
Comoros	COM	Safe abortion services	3.3	2.7
Comoros	COM	TT - Tetanus toxoid vaccination	85.0	62.1
Comoros	COM	IPTp - Intermittent preventive treatment of malaria during pregnancy	30.8	22.5
Comoros	COM	Syphilis detection and treatment	22.8	16.7

Comoros	COM	Iron supplementation in pregnancy	12.6	9.2
Comoros	COM	Hypertensive disorder case management	11.8	8.6
Comoros	COM	Diabetes case management	9.2	6.7
Comoros	COM	Malaria case management	38.2	27.9
Comoros	COM	MgSO4 management of pre-eclampsia	23.4	17.1
Comoros	COM	Thermal protection	75.3	57.9
Comoros	COM	Clean cord care	72.7	55.9
Comoros	COM	Clean birth environment	62.4	48.0
Comoros	COM	Immediate drying and additional stimulation	69.7	53.6
Comoros	COM	Neonatal resuscitation	41.9	32.2
Comoros	COM	Antibiotics for preterm or prolonged PROM	57.0	43.9
Comoros	COM	Parenteral administration of anti-convulsants	54.4	41.9
Comoros	COM	Parenteral administration of uterotonics	68.0	52.3
Comoros	COM	Parenteral administration of antibiotics	57.0	43.9
Comoros	COM	Assisted vaginal delivery	19.2	14.8
Comoros	COM	Manual removal of placenta	28.5	21.9
Comoros	COM	Removal of retained products of conception	25.2	19.4
Comoros	COM	Cesarean delivery	6.6	5.1
Comoros	COM	Blood transfusion	9.6	7.4
Comoros	COM	Induction of labor for pregnancies lasting 41+ weeks	1.3	1.0
Comoros	COM	Complementary feeding - education only	25.2	19.4
Comoros	COM	Complementary feeding - supplementary feeding and education	25.2	19.4
Comoros	COM	Vitamin A supplementation	21.0	16.2
Comoros	COM	Improved sanitation - Utilization of latrines or toilets	35.9	35.9
Comoros	COM	Improved water source	80.2	80.2
Comoros	COM	Water connection in the home	47.6	47.6
Comoros	COM	Hand washing with soap	20.0	20.0
Comoros	COM	Hygienic disposal of children's stools	60.4	60.4
Comoros	COM	ITN/IRS - Households protected from malaria	60.6	46.8
Comoros	COM	Injectable antibiotics for neonatal sepsis	76.1	55.6
Comoros	COM	ORS - oral rehydration solution	37.5	28.9
Comoros	COM	Antibiotics for treatment of dysentery	19.4	14.9
Comoros	COM	Zinc for treatment of diarrhea	0.4	0.3
Comoros	COM	Oral antibiotics for pneumonia	38.1	29.3
Comoros	COM	Vitamin A for treatment of measles	21.0	16.2
Comoros	COM	ACTs- Artemisinin compounds for treatment of malaria	3.9	3.0
Comoros	COM	SAM - treatment for severe acute malnutrition	25.0	19.2
Comoros	COM	BCG vaccine	94.0	68.7
Comoros	COM	Polio vaccine	94.0	68.7
Comoros	COM	DPT vaccine	91.0	66.5
Comoros	COM	H. influenzae type b vaccine	91.0	83.2

Comoros	COM	HepB vaccine	91.0	66.5
Comoros	COM	Measles vaccine	90.0	65.8
Comoros	COM	Global wasting (<-2 SD) rate	11.2	13.4
Comoros	COM	Contraceptive prevalence (CPR)	26.4	21.4
Congo	COG	TT - Tetanus toxoid vaccination	85.0	62.1
Congo	COG	IPTp - Intermittent preventive treatment of malaria during pregnancy	23.2	17.0
Congo	COG	Syphilis detection and treatment	23.0	16.8
Congo	COG	Iron supplementation in pregnancy	42.9	31.4
Congo	COG	Hypertensive disorder case management	19.0	13.9
Congo	COG	Diabetes case management	14.8	10.8
Congo	COG	Malaria case management	61.2	44.7
Congo	COG	MgSO4 management of pre-eclampsia	37.5	27.4
Congo	COG	Thermal protection	90.4	69.6
Congo	COG	Clean cord care	87.3	67.2
Congo	COG	Clean birth environment	75.0	57.7
Congo	COG	Immediate drying and additional stimulation	83.8	64.5
Congo	COG	Neonatal resuscitation	50.3	38.7
Congo	COG	Antibiotics for preterm or prolonged PROM	68.5	52.7
Congo	COG	Parenteral administration of anti-convulsants	65.4	50.3
Congo	COG	Parenteral administration of uterotonics	81.8	62.9
Congo	COG	Parenteral administration of antibiotics	68.5	52.7
Congo	COG	Assisted vaginal delivery	23.1	17.8
Congo	COG	Manual removal of placenta	34.2	26.3
Congo	COG	Removal of retained products of conception	30.3	23.3
Congo	COG	Cesarean delivery	8.0	6.2
Congo	COG	Blood transfusion	11.5	8.8
Congo	COG	Induction of labor for pregnancies lasting 41+ weeks	1.6	1.2
Congo	COG	Complementary feeding - education only	23.9	18.4
Congo	COG	Complementary feeding - supplementary feeding and education	23.9	18.4
Congo	COG	Vitamin A supplementation	12.0	9.3
Congo	COG	Improved sanitation - Utilization of latrines or toilets	20.2	20.2
Congo	COG	Improved water source	73.2	73.2
Congo	COG	Water connection in the home	38.0	38.0
Congo	COG	Hand washing with soap	49.2	49.2
Congo	COG	Hygienic disposal of children's stools	62.8	62.8
Congo	COG	ITN/IRS - Households protected from malaria	66.1	51.0
Congo	COG	Injectable antibiotics for neonatal sepsis	91.5	66.9
Congo	COG	ORS - oral rehydration solution	26.9	20.7
Congo	COG	Antibiotics for treatment of dysentery	4.9	3.8
Congo	COG	Zinc for treatment of diarrhea	16.0	12.3
Congo	COG	Oral antibiotics for pneumonia	28.2	21.7

Congo	COG	Vitamin A for treatment of measles	12.0	9.2
Congo	COG	ACTs- Artemisinin compounds for treatment of malaria	8.8	6.8
Congo	COG	SAM - treatment for severe acute malnutrition	1.0	0.8
Congo	COG	BCG vaccine	81.0	59.2
Congo	COG	Polio vaccine	75.0	54.8
Congo	COG	DPT vaccine	75.0	54.8
Congo	COG	H. influenzae type b vaccine	75.0	68.5
Congo	COG	HepB vaccine	75.0	54.8
Congo	COG	Pneumococcal vaccine	73.0	66.7
Congo	COG	Rotavirus vaccine	72.0	65.8
Congo	COG	Meningococcal A	83.0	60.7
Congo	COG	Measles vaccine	75.0	54.8
Congo	COG	Global wasting (<-2 SD) rate	8.1	9.8
Congo	COG	Contraceptive prevalence (CPR)	38.6	31.4
Costa Rica	CRI	Safe abortion services	0.6	0.5
Costa Rica	CRI	Syphilis detection and treatment	24.2	17.7
Costa Rica	CRI	Hypertensive disorder case management	21.7	15.9
Costa Rica	CRI	Diabetes case management	16.9	12.4
Costa Rica	CRI	Malaria case management	69.9	51.1
Costa Rica	CRI	MgSO4 management of pre-eclampsia	42.9	31.4
Costa Rica	CRI	Thermal protection	97.0	74.6
Costa Rica	CRI	Clean cord care	93.6	72.0
Costa Rica	CRI	Clean birth environment	80.5	61.9
Costa Rica	CRI	Immediate drying and additional stimulation	89.8	69.1
Costa Rica	CRI	Neonatal resuscitation	53.9	41.5
Costa Rica	CRI	Antibiotics for preterm or prolonged PROM	73.4	56.5
Costa Rica	CRI	Parenteral administration of anti-convulsants	70.1	53.9
Costa Rica	CRI	Parenteral administration of uterotonics	87.7	67.5
Costa Rica	CRI	Parenteral administration of antibiotics	73.4	56.5
Costa Rica	CRI	Assisted vaginal delivery	24.8	19.1
Costa Rica	CRI	Manual removal of placenta	36.7	28.2
Costa Rica	CRI	Removal of retained products of conception	32.5	25.0
Costa Rica	CRI	Cesarean delivery	8.5	6.5
Costa Rica	CRI	Blood transfusion	12.4	9.5
Costa Rica	CRI	Induction of labor for pregnancies lasting 41+ weeks	1.7	1.3
Costa Rica	CRI	Improved sanitation - Utilization of latrines or toilets	97.8	97.8
Costa Rica	CRI	Improved water source	99.0	99.0
Costa Rica	CRI	Water connection in the home	99.0	99.0
Costa Rica	CRI	Hand washing with soap	89.4	89.4
Costa Rica	CRI	Hygienic disposal of children's stools	27.9	27.9
Costa Rica	CRI	Injectable antibiotics for neonatal sepsis	98.1	71.7

Costa Rica	CRI	ORS - oral rehydration solution	40.0	30.8
Costa Rica	CRI	Antibiotics for treatment of dysentery	8.7	6.7
Costa Rica	CRI	Zinc for treatment of diarrhea	1.3	1.0
Costa Rica	CRI	Oral antibiotics for pneumonia	77.2	59.4
Costa Rica	CRI	BCG vaccine	92.0	67.3
Costa Rica	CRI	Polio vaccine	94.0	68.7
Costa Rica	CRI	DPT vaccine	94.0	68.7
Costa Rica	CRI	H. influenzae type b vaccine	94.0	85.9
Costa Rica	CRI	HepB vaccine	98.0	71.6
Costa Rica	CRI	Pneumococcal vaccine	96.0	87.7
Costa Rica	CRI	Measles vaccine	94.0	68.7
Costa Rica	CRI	Global wasting (<-2 SD) rate	2.4	2.9
Costa Rica	CRI	Contraceptive prevalence (CPR)	77.45	62.9
Côte d'Ivoire	CIV	TT - Tetanus toxoid vaccination	85.0	62.1
Côte d'Ivoire	CIV	IPTp - Intermittent preventive treatment of malaria during pregnancy	43.8	32.0
Côte d'Ivoire	CIV	Syphilis detection and treatment	23.0	16.8
Côte d'Ivoire	CIV	Iron supplementation in pregnancy	25.0	18.3
Côte d'Ivoire	CIV	Hypertensive disorder case management	12.3	9.0
Côte d'Ivoire	CIV	Diabetes case management	9.6	7.0
Côte d'Ivoire	CIV	Malaria case management	39.7	29.0
Côte d'Ivoire	CIV	MgSO4 management of pre-eclampsia	24.4	17.8
Côte d'Ivoire	CIV	Thermal protection	69.0	53.1
Côte d'Ivoire	CIV	Clean cord care	66.6	51.2
Côte d'Ivoire	CIV	Clean birth environment	57.2	44.0
Côte d'Ivoire	CIV	Immediate drying and additional stimulation	63.9	49.2
Côte d'Ivoire	CIV	Neonatal resuscitation	38.4	29.5
Côte d'Ivoire	CIV	Antibiotics for preterm or prolonged PROM	52.2	40.2
Côte d'Ivoire	CIV	Parenteral administration of anti-convulsants	49.9	38.4
Côte d'Ivoire	CIV	Parenteral administration of uterotonics	62.4	48.0
Côte d'Ivoire	CIV	Parenteral administration of antibiotics	52.2	40.2
Côte d'Ivoire	CIV	Assisted vaginal delivery	17.6	13.5
Côte d'Ivoire	CIV	Manual removal of placenta	26.1	20.1
Côte d'Ivoire	CIV	Removal of retained products of conception	23.1	17.8
Côte d'Ivoire	CIV	Cesarean delivery	6.1	4.7
Côte d'Ivoire	CIV	Blood transfusion	8.8	6.8
Côte d'Ivoire	CIV	Induction of labor for pregnancies lasting 41+ weeks	1.2	0.9
Côte d'Ivoire	CIV	Complementary feeding - education only	26.0	20.1
Côte d'Ivoire	CIV	Complementary feeding - supplementary feeding and education	26.0	20.1
Côte d'Ivoire	CIV	Vitamin A supplementation	94.0	72.5
Côte d'Ivoire	CIV	Improved sanitation - Utilization of latrines or toilets	32.1	32.1
Côte d'Ivoire	CIV	Improved water source	72.9	72.9

Côte d'Ivoire	CIV	Water connection in the home	30.2	30.2
Côte d'Ivoire	CIV	Hand washing with soap	18.0	18.0
Côte d'Ivoire	CIV	Hygienic disposal of children's stools	54.4	54.4
Côte d'Ivoire	CIV	ITN/IRS - Households protected from malaria	75.8	58.5
Côte d'Ivoire	CIV	Injectable antibiotics for neonatal sepsis	69.8	51.0
Côte d'Ivoire	CIV	ORS - oral rehydration solution	16.5	12.7
Côte d'Ivoire	CIV	Antibiotics for treatment of dysentery	17.0	13.1
Côte d'Ivoire	CIV	Zinc for treatment of diarrhea	18.3	14.1
Côte d'Ivoire	CIV	Oral antibiotics for pneumonia	44.0	33.9
Côte d'Ivoire	CIV	Vitamin A for treatment of measles	94.0	72.3
Côte d'Ivoire	CIV	ACTs- Artemisinin compounds for treatment of malaria	0.3	0.2
Côte d'Ivoire	CIV	BCG vaccine	98.0	71.6
Côte d'Ivoire	CIV	Polio vaccine	82.0	59.9
Côte d'Ivoire	CIV	DPT vaccine	82.0	59.9
Côte d'Ivoire	CIV	H. influenzae type b vaccine	82.0	74.9
Côte d'Ivoire	CIV	HepB vaccine	82.0	59.9
Côte d'Ivoire	CIV	Pneumococcal vaccine	81.0	74.0
Côte d'Ivoire	CIV	Rotavirus vaccine	59.0	53.9
Côte d'Ivoire	CIV	Measles vaccine	71.0	51.9
Côte d'Ivoire	CIV	Global wasting (<-2 SD) rate	6.0	7.2
Côte d'Ivoire	CIV	Contraceptive prevalence (CPR)	21.25	17.3
Cuba	CUB	Safe abortion services	53.7	43.6
Cuba	CUB	Syphilis detection and treatment	24.3	17.8
Cuba	CUB	Hypertensive disorder case management	23.5	17.2
Cuba	CUB	Diabetes case management	18.3	13.4
Cuba	CUB	Malaria case management	75.8	55.4
Cuba	CUB	MgSO4 management of pre-eclampsia	46.5	34.0
Cuba	CUB	Thermal protection	97.9	75.3
Cuba	CUB	Clean cord care	94.5	72.7
Cuba	CUB	Clean birth environment	81.2	62.5
Cuba	CUB	Immediate drying and additional stimulation	90.7	69.8
Cuba	CUB	Neonatal resuscitation	54.5	41.9
Cuba	CUB	Antibiotics for preterm or prolonged PROM	74.1	57.0
Cuba	CUB	Parenteral administration of anti-convulsants	70.8	54.5
Cuba	CUB	Parenteral administration of uterotonics	88.5	68.1
Cuba	CUB	Parenteral administration of antibiotics	74.1	57.0
Cuba	CUB	Assisted vaginal delivery	25.0	19.2
Cuba	CUB	Manual removal of placenta	37.0	28.5
Cuba	CUB	Removal of retained products of conception	32.8	25.2
Cuba	CUB	Cesarean delivery	8.6	6.6
Cuba	CUB	Blood transfusion	12.5	9.6
Cuba	CUB	Induction of labor for pregnancies lasting 41+ weeks	1.7	1.3

Cuba	CUB	Complementary feeding - education only	80.3	62.0
Cuba	CUB	Complementary feeding - supplementary feeding and education	80.3	62.0
Cuba	CUB	Improved sanitation - Utilization of latrines or toilets	92.8	92.8
Cuba	CUB	Improved water source	95.3	95.3
Cuba	CUB	Water connection in the home	75.8	75.8
Cuba	CUB	Hand washing with soap	85.2	85.2
Cuba	CUB	Hygienic disposal of children's stools	87.9	87.9
Cuba	CUB	Injectable antibiotics for neonatal sepsis	99.0	72.4
Cuba	CUB	ORS - oral rehydration solution	60.9	46.9
Cuba	CUB	Antibiotics for treatment of dysentery	2.0	1.5
Cuba	CUB	Zinc for treatment of diarrhea	17.3	13.3
Cuba	CUB	Oral antibiotics for pneumonia	92.6	71.3
Cuba	CUB	BCG vaccine	99.0	72.4
Cuba	CUB	Polio vaccine	99.0	72.4
Cuba	CUB	DPT vaccine	99.0	72.4
Cuba	CUB	H. influenzae type b vaccine	99.0	90.5
Cuba	CUB	HepB vaccine	99.0	72.4
Cuba	CUB	Measles vaccine	99.0	72.4
Cuba	CUB	Global wasting (<-2 SD) rate	2.2	2.6
Cuba	CUB	Contraceptive prevalence (CPR)	74.4	60.4
Dem. People's Republic of Korea	PRK	Safe abortion services	100.0	81.2
Dem. People's Republic of Korea	PRK	TT - Tetanus toxoid vaccination	98.0	71.6
Dem. People's Republic of Korea	PRK	Syphilis detection and treatment	24.4	17.8
Dem. People's Republic of Korea	PRK	Hypertensive disorder case management	22.5	16.4
Dem. People's Republic of Korea	PRK	Diabetes case management	17.6	12.9
Dem. People's Republic of Korea	PRK	Malaria case management	72.6	53.1
Dem. People's Republic of Korea	PRK	MgSO4 management of pre-eclampsia	44.5	32.5
Dem. People's Republic of Korea	PRK	Thermal protection	91.1	70.1
Dem. People's Republic of Korea	PRK	Clean cord care	88.0	67.7
Dem. People's Republic of Korea	PRK	Clean birth environment	75.6	58.2
Dem. People's Republic of Korea	PRK	Immediate drying and additional stimulation	84.4	64.9
Dem. People's Republic of Korea	PRK	Neonatal resuscitation	50.7	39.0
Dem. People's Republic of Korea	PRK	Antibiotics for preterm or prolonged PROM	69.0	53.1
Dem. People's Republic of Korea	PRK	Parenteral administration of anti-convulsants	65.9	50.7

Dem. People's Republic of Korea	PRK	Parenteral administration of uterotonics	82.4	63.4
Dem. People's Republic of Korea	PRK	Parenteral administration of antibiotics	69.0	53.1
Dem. People's Republic of Korea	PRK	Assisted vaginal delivery	23.3	17.9
Dem. People's Republic of Korea	PRK	Manual removal of placenta	34.5	26.5
Dem. People's Republic of Korea	PRK	Removal of retained products of conception	30.6	23.5
Dem. People's Republic of Korea	PRK	Cesarean delivery	8.0	6.2
Dem. People's Republic of Korea	PRK	Blood transfusion	11.6	8.9
Dem. People's Republic of Korea	PRK	Induction of labor for pregnancies lasting 41+ weeks	1.6	1.2
Dem. People's Republic of Korea	PRK	Complementary feeding - education only	48.7	37.6
Dem. People's Republic of Korea	PRK	Complementary feeding - supplementary feeding and education	48.7	37.6
Dem. People's Republic of Korea	PRK	Vitamin A supplementation	90.0	69.4
Dem. People's Republic of Korea	PRK	Improved sanitation - Utilization of latrines or toilets	83.2	83.2
Dem. People's Republic of Korea	PRK	Improved water source	99.0	99.0
Dem. People's Republic of Korea	PRK	Water connection in the home	63.8	63.8
Dem. People's Republic of Korea	PRK	Hygienic disposal of children's stools	74.2	74.2
Dem. People's Republic of Korea	PRK	Injectable antibiotics for neonatal sepsis	92.2	67.4
Dem. People's Republic of Korea	PRK	ORS - oral rehydration solution	74.1	57.0
Dem. People's Republic of Korea	PRK	Zinc for treatment of diarrhea	50.7	39.0
Dem. People's Republic of Korea	PRK	Oral antibiotics for pneumonia	79.8	61.4
Dem. People's Republic of Korea	PRK	Vitamin A for treatment of measles	90.0	69.3
Dem. People's Republic of Korea	PRK	BCG vaccine	96.0	70.2
Dem. People's Republic of Korea	PRK	Polio vaccine	99.0	72.4
Dem. People's Republic of Korea	PRK	DPT vaccine	97.0	70.9
Dem. People's Republic of Korea	PRK	H. influenzae type b vaccine	97.0	88.7
Dem. People's Republic of Korea	PRK	HepB vaccine	97.0	70.9
Dem. People's Republic of Korea	PRK	Measles vaccine	98.0	71.6
Dem. People's Republic of Korea	PRK	Global wasting (<-2 SD) rate	10.6	12.7

Dem. People's Republic of Korea	PRK	Contraceptive prevalence (CPR)	74.1	60.2
Democratic Republic of the Congo	COD	TT - Tetanus toxoid vaccination	85.0	62.1
Democratic Republic of the Congo	COD	IPTp - Intermittent preventive treatment of malaria during pregnancy	15.0	11.0
Democratic Republic of the Congo	COD	Syphilis detection and treatment	34.9	25.5
Democratic Republic of the Congo	COD	Iron supplementation in pregnancy	4.7	3.4
Democratic Republic of the Congo	COD	Hypertensive disorder case management	4.6	3.4
Democratic Republic of the Congo	COD	Diabetes case management	4.7	3.4
Democratic Republic of the Congo	COD	Malaria case management	31.9	23.3
Democratic Republic of the Congo	COD	MgSO4 management of pre-eclampsia	6.6	4.8
Democratic Republic of the Congo	COD	Thermal protection	78.9	60.7
Democratic Republic of the Congo	COD	Clean cord care	66.6	51.2
Democratic Republic of the Congo	COD	Clean birth environment	40.9	31.5
Democratic Republic of the Congo	COD	Immediate drying and additional stimulation	63.2	48.6
Democratic Republic of the Congo	COD	Neonatal resuscitation	4.4	3.4
Democratic Republic of the Congo	COD	Antibiotics for preterm or prolonged PROM	12.2	9.4
Democratic Republic of the Congo	COD	Parenteral administration of anti-convulsants	14.2	10.9
Democratic Republic of the Congo	COD	Parenteral administration of uterotonics	61.3	47.2
Democratic Republic of the Congo	COD	Parenteral administration of antibiotics	12.2	9.4
Democratic Republic of the Congo	COD	Assisted vaginal delivery	8.9	6.8
Democratic Republic of the Congo	COD	Manual removal of placenta	21.2	16.3
Democratic Republic of the Congo	COD	Removal of retained products of conception	19.9	15.3
Democratic Republic of the Congo	COD	Cesarean delivery	0.8	0.6
Democratic Republic of the Congo	COD	Blood transfusion	6.6	5.1
Democratic Republic of the Congo	COD	Induction of labor for pregnancies lasting 41+ weeks	1.2	0.9
Democratic Republic of the Congo	COD	Complementary feeding - education only	19.9	15.4
Democratic Republic of the Congo	COD	Complementary feeding - supplementary feeding and education	19.9	15.4
Democratic Republic of the Congo	COD	Vitamin A supplementation	1.0	0.8

Democratic Republic of the Congo	COD	Improved sanitation - Utilization of latrines or toilets	20.5	20.5
Democratic Republic of the Congo	COD	Improved water source	43.2	43.2
Democratic Republic of the Congo	COD	Water connection in the home	13.9	13.9
Democratic Republic of the Congo	COD	Hand washing with soap	4.4	4.4
Democratic Republic of the Congo	COD	Hygienic disposal of children's stools	61.1	61.1
Democratic Republic of the Congo	COD	ITN/IRS - Households protected from malaria	70.0	54.0
Democratic Republic of the Congo	COD	Injectable antibiotics for neonatal sepsis	79.9	58.4
Democratic Republic of the Congo	COD	ORS - oral rehydration solution	39.1	30.1
Democratic Republic of the Congo	COD	Antibiotics for treatment of dysentery	38.4	29.5
Democratic Republic of the Congo	COD	Zinc for treatment of diarrhea	2.4	1.8
Democratic Republic of the Congo	COD	Oral antibiotics for pneumonia	41.6	32.0
Democratic Republic of the Congo	COD	Vitamin A for treatment of measles	1.0	0.8
Democratic Republic of the Congo	COD	ACTs- Artemisinin compounds for treatment of malaria	2.0	1.5
Democratic Republic of the Congo	COD	SAM - treatment for severe acute malnutrition	11.2	8.6
Democratic Republic of the Congo	COD	BCG vaccine	83.0	60.7
Democratic Republic of the Congo	COD	Polio vaccine	79.0	57.8
Democratic Republic of the Congo	COD	DPT vaccine	81.0	59.2
Democratic Republic of the Congo	COD	H. influenzae type b vaccine	81.0	74.0
Democratic Republic of the Congo	COD	HepB vaccine	81.0	59.2
Democratic Republic of the Congo	COD	Pneumococcal vaccine	81.0	74.0
Democratic Republic of the Congo	COD	Meningococcal A	99.0	72.4
Democratic Republic of the Congo	COD	Measles vaccine	80.0	58.5
Democratic Republic of the Congo	COD	Global wasting (<-2 SD) rate	8.1	9.7
Democratic Republic of the Congo	COD	Contraceptive prevalence (CPR)	24.55	19.9
Djibouti	DJI	Safe abortion services	3.3	2.7
Djibouti	DJI	TT - Tetanus toxoid vaccination	98.0	71.6
Djibouti	DJI	Syphilis detection and treatment	5.6	4.1
Djibouti	DJI	Hypertensive disorder case management	5.4	3.9
Djibouti	DJI	Diabetes case management	4.2	3.1

Djibouti	DJI	Malaria case management	17.5	12.8
Djibouti	DJI	MgSO4 management of pre-eclampsia	10.7	7.8
Djibouti	DJI	Thermal protection	86.4	66.5
Djibouti	DJI	Clean cord care	83.4	64.2
Djibouti	DJI	Clean birth environment	71.7	55.2
Djibouti	DJI	Immediate drying and additional stimulation	80.0	61.6
Djibouti	DJI	Neonatal resuscitation	48.1	37.0
Djibouti	DJI	Antibiotics for preterm or prolonged PROM	65.4	50.3
Djibouti	DJI	Parenteral administration of anti-convulsants	62.5	48.1
Djibouti	DJI	Parenteral administration of uterotonics	78.1	60.1
Djibouti	DJI	Parenteral administration of antibiotics	65.4	50.3
Djibouti	DJI	Assisted vaginal delivery	22.1	17.0
Djibouti	DJI	Manual removal of placenta	32.7	25.2
Djibouti	DJI	Removal of retained products of conception	29.0	22.3
Djibouti	DJI	Cesarean delivery	7.6	5.8
Djibouti	DJI	Blood transfusion	11.0	8.5
Djibouti	DJI	Induction of labor for pregnancies lasting 41+ weeks	1.5	1.2
Djibouti	DJI	Complementary feeding - education only	23.1	17.8
Djibouti	DJI	Complementary feeding - supplementary feeding and education	23.1	17.8
Djibouti	DJI	Vitamin A supplementation	78.0	60.2
Djibouti	DJI	Improved sanitation - Utilization of latrines or toilets	63.6	63.6
Djibouti	DJI	Improved water source	75.6	75.6
Djibouti	DJI	Water connection in the home	61.8	61.8
Djibouti	DJI	ITN/IRS - Households protected from malaria	30.2	23.3
Djibouti	DJI	Injectable antibiotics for neonatal sepsis	87.4	63.9
Djibouti	DJI	ORS - oral rehydration solution	61.6	47.4
Djibouti	DJI	Oral antibiotics for pneumonia	62.1	47.8
Djibouti	DJI	Vitamin A for treatment of measles	78.0	60.0
Djibouti	DJI	SAM - treatment for severe acute malnutrition	32.4	24.9
Djibouti	DJI	BCG vaccine	93.0	68.0
Djibouti	DJI	Polio vaccine	84.0	61.4
Djibouti	DJI	DPT vaccine	84.0	61.4
Djibouti	DJI	H. influenzae type b vaccine	84.0	76.8
Djibouti	DJI	HepB vaccine	84.0	61.4
Djibouti	DJI	Pneumococcal vaccine	84.0	76.8
Djibouti	DJI	Rotavirus vaccine	87.0	79.5
Djibouti	DJI	Measles vaccine	86.0	62.9
Djibouti	DJI	Global wasting (<-2 SD) rate	26.8	32.1
Djibouti	DJI	Contraceptive prevalence (CPR)	27.6	22.4
Dominican Republic	DOM	Safe abortion services	53.7	43.6
Dominican Republic	DOM	TT - Tetanus toxoid vaccination	99.0	72.4

Dominican Republic	DOM	Syphilis detection and treatment	24.2	17.7
Dominican Republic	DOM	Iron supplementation in pregnancy	81.5	59.6
Dominican Republic	DOM	Hypertensive disorder case management	22.3	16.3
Dominican Republic	DOM	Diabetes case management	17.4	12.7
Dominican Republic	DOM	Malaria case management	72.0	52.6
Dominican Republic	DOM	MgSO4 management of pre-eclampsia	44.2	32.3
Dominican Republic	DOM	Thermal protection	96.7	74.4
Dominican Republic	DOM	Clean cord care	93.4	71.9
Dominican Republic	DOM	Clean birth environment	80.3	61.8
Dominican Republic	DOM	Immediate drying and additional stimulation	89.6	68.9
Dominican Republic	DOM	Neonatal resuscitation	53.8	41.4
Dominican Republic	DOM	Antibiotics for preterm or prolonged PROM	73.2	56.3
Dominican Republic	DOM	Parenteral administration of anti-convulsants	70.0	53.9
Dominican Republic	DOM	Parenteral administration of uterotonics	87.5	67.3
Dominican Republic	DOM	Parenteral administration of antibiotics	73.2	56.3
Dominican Republic	DOM	Assisted vaginal delivery	24.7	19.0
Dominican Republic	DOM	Manual removal of placenta	36.6	28.2
Dominican Republic	DOM	Removal of retained products of conception	32.4	24.9
Dominican Republic	DOM	Cesarean delivery	8.5	6.5
Dominican Republic	DOM	Blood transfusion	12.3	9.5
Dominican Republic	DOM	Induction of labor for pregnancies lasting 41+ weeks	1.7	1.3
Dominican Republic	DOM	Complementary feeding - education only	68.5	52.9
Dominican Republic	DOM	Complementary feeding - supplementary feeding and education	68.5	52.9
Dominican Republic	DOM	Improved sanitation - Utilization of latrines or toilets	83.9	83.9
Dominican Republic	DOM	Improved water source	96.7	96.7
Dominican Republic	DOM	Water connection in the home	77.9	77.9
Dominican Republic	DOM	Hand washing with soap	57.1	57.1
Dominican Republic	DOM	Hygienic disposal of children's stools	27.4	27.4
Dominican Republic	DOM	Injectable antibiotics for neonatal sepsis	97.9	71.6
Dominican Republic	DOM	ORS - oral rehydration solution	47.5	36.6
Dominican Republic	DOM	Antibiotics for treatment of dysentery	30.5	23.5
Dominican Republic	DOM	Oral antibiotics for pneumonia	72.4	55.7
Dominican Republic	DOM	BCG vaccine	99.0	72.4
Dominican Republic	DOM	Polio vaccine	89.0	65.1
Dominican Republic	DOM	DPT vaccine	94.0	68.7
Dominican Republic	DOM	H. influenzae type b vaccine	90.0	82.3
Dominican Republic	DOM	HepB vaccine	92.0	67.3
Dominican Republic	DOM	Pneumococcal vaccine	70.0	64.0
Dominican Republic	DOM	Rotavirus vaccine	82.0	74.9
Dominican Republic	DOM	Measles vaccine	95.0	69.4
Dominican Republic	DOM	Global wasting (<-2 SD) rate	2.5	3.0

Dominican Republic	DOM	Contraceptive prevalence (CPR)	71.15	57.8
Ecuador	ECU	Safe abortion services	0.2	0.2
Ecuador	ECU	TT - Tetanus toxoid vaccination	88.0	64.3
Ecuador	ECU	Syphilis detection and treatment	23.9	17.5
Ecuador	ECU	Hypertensive disorder case management	21.2	15.5
Ecuador	ECU	Diabetes case management	16.5	12.1
Ecuador	ECU	Malaria case management	68.4	50.0
Ecuador	ECU	MgSO4 management of pre-eclampsia	41.9	30.6
Ecuador	ECU	Thermal protection	89.3	68.7
Ecuador	ECU	Clean cord care	86.2	66.3
Ecuador	ECU	Clean birth environment	74.1	57.0
Ecuador	ECU	Immediate drying and additional stimulation	82.7	63.6
Ecuador	ECU	Neonatal resuscitation	49.7	38.2
Ecuador	ECU	Antibiotics for preterm or prolonged PROM	67.6	52.0
Ecuador	ECU	Parenteral administration of anti-convulsants	64.6	49.7
Ecuador	ECU	Parenteral administration of uterotonics	80.8	62.2
Ecuador	ECU	Parenteral administration of antibiotics	67.6	52.0
Ecuador	ECU	Assisted vaginal delivery	22.8	17.5
Ecuador	ECU	Manual removal of placenta	33.8	26.0
Ecuador	ECU	Removal of retained products of conception	29.9	23.0
Ecuador	ECU	Cesarean delivery	7.9	6.1
Ecuador	ECU	Blood transfusion	11.4	8.8
Ecuador	ECU	Induction of labor for pregnancies lasting 41+ weeks	1.6	1.2
Ecuador	ECU	Complementary feeding - education only	69.4	53.6
Ecuador	ECU	Complementary feeding - supplementary feeding and education	69.4	53.6
Ecuador	ECU	Improved sanitation - Utilization of latrines or toilets	88.0	88.0
Ecuador	ECU	Improved water source	94.0	94.0
Ecuador	ECU	Water connection in the home	82.6	82.6
Ecuador	ECU	Injectable antibiotics for neonatal sepsis	90.4	66.1
Ecuador	ECU	ORS - oral rehydration solution	27.3	21.0
Ecuador	ECU	Antibiotics for treatment of dysentery	32.4	24.9
Ecuador	ECU	BCG vaccine	90.0	65.8
Ecuador	ECU	Polio vaccine	85.0	62.1
Ecuador	ECU	DPT vaccine	85.0	62.1
Ecuador	ECU	H. influenzae type b vaccine	85.0	77.7
Ecuador	ECU	HepB vaccine	85.0	62.1
Ecuador	ECU	Pneumococcal vaccine	85.0	77.7
Ecuador	ECU	Rotavirus vaccine	85.0	77.7
Ecuador	ECU	Measles vaccine	83.0	60.7
Ecuador	ECU	Global wasting (<-2 SD) rate	2.5	3.0
Ecuador	ECU	Contraceptive prevalence (CPR)	79.35	64.4

Egypt	EGY	Safe abortion services	2.2	1.8
Egypt	EGY	TT - Tetanus toxoid vaccination	86.0	62.9
Egypt	EGY	Syphilis detection and treatment	22.6	16.5
Egypt	EGY	Iron supplementation in pregnancy	36.1	26.4
Egypt	EGY	Hypertensive disorder case management	20.1	14.7
Egypt	EGY	Diabetes case management	15.7	11.5
Egypt	EGY	Malaria case management	65.0	47.5
Egypt	EGY	MgSO4 management of pre-eclampsia	39.8	29.1
Egypt	EGY	Thermal protection	85.7	65.9
Egypt	EGY	Clean cord care	82.8	63.7
Egypt	EGY	Clean birth environment	71.1	54.7
Egypt	EGY	Immediate drying and additional stimulation	79.4	61.1
Egypt	EGY	Neonatal resuscitation	47.7	36.7
Egypt	EGY	Antibiotics for preterm or prolonged PROM	64.9	49.9
Egypt	EGY	Parenteral administration of anti-convulsants	62.0	47.7
Egypt	EGY	Parenteral administration of uterotonics	77.5	59.6
Egypt	EGY	Parenteral administration of antibiotics	64.9	49.9
Egypt	EGY	Assisted vaginal delivery	21.9	16.9
Egypt	EGY	Manual removal of placenta	32.4	24.9
Egypt	EGY	Removal of retained products of conception	28.7	22.1
Egypt	EGY	Cesarean delivery	7.5	5.8
Egypt	EGY	Blood transfusion	10.9	8.4
Egypt	EGY	Induction of labor for pregnancies lasting 41+ weeks	1.5	1.2
Egypt	EGY	Complementary feeding - education only	43.2	33.3
Egypt	EGY	Complementary feeding - supplementary feeding and education	43.2	33.3
Egypt	EGY	Vitamin A supplementation	68.0	52.5
Egypt	EGY	Improved sanitation - Utilization of latrines or toilets	94.2	94.2
Egypt	EGY	Improved water source	99.0	99.0
Egypt	EGY	Water connection in the home	97.2	97.2
Egypt	EGY	Hand washing with soap	89.0	89.0
Egypt	EGY	Hygienic disposal of children's stools	35.7	35.7
Egypt	EGY	Injectable antibiotics for neonatal sepsis	86.7	63.4
Egypt	EGY	ORS - oral rehydration solution	28.4	21.9
Egypt	EGY	Antibiotics for treatment of dysentery	13.2	10.2
Egypt	EGY	Zinc for treatment of diarrhea	1.7	1.3
Egypt	EGY	Oral antibiotics for pneumonia	67.7	52.1
Egypt	EGY	Vitamin A for treatment of measles	68.0	52.3
Egypt	EGY	BCG vaccine	95.0	69.4
Egypt	EGY	Polio vaccine	95.0	69.4
Egypt	EGY	DPT vaccine	95.0	69.4
Egypt	EGY	H. influenzae type b vaccine	95.0	86.8

Egypt	EGY	HepB vaccine	95.0	69.4
Egypt	EGY	Measles vaccine	94.0	68.7
Egypt	EGY	Global wasting (<-2 SD) rate	9.4	11.3
Egypt	EGY	Contraceptive prevalence (CPR)	60.95	49.5
El Salvador	SLV	Safe abortion services	0.6	0.5
El Salvador	SLV	TT - Tetanus toxoid vaccination	92.0	67.3
El Salvador	SLV	Syphilis detection and treatment	23.7	17.3
El Salvador	SLV	Hypertensive disorder case management	21.6	15.8
El Salvador	SLV	Diabetes case management	16.9	12.4
El Salvador	SLV	Malaria case management	69.9	51.1
El Salvador	SLV	MgSO4 management of pre-eclampsia	42.8	31.3
El Salvador	SLV	Thermal protection	96.4	74.2
El Salvador	SLV	Clean cord care	93.1	71.6
El Salvador	SLV	Clean birth environment	80.0	61.6
El Salvador	SLV	Immediate drying and additional stimulation	89.3	68.7
El Salvador	SLV	Neonatal resuscitation	53.6	41.2
El Salvador	SLV	Antibiotics for preterm or prolonged PROM	73.0	56.2
El Salvador	SLV	Parenteral administration of anti-convulsants	69.7	53.6
El Salvador	SLV	Parenteral administration of uterotonics	87.2	67.1
El Salvador	SLV	Parenteral administration of antibiotics	73.0	56.2
El Salvador	SLV	Assisted vaginal delivery	24.7	19.0
El Salvador	SLV	Manual removal of placenta	36.5	28.1
El Salvador	SLV	Removal of retained products of conception	32.3	24.9
El Salvador	SLV	Cesarean delivery	8.5	6.5
El Salvador	SLV	Blood transfusion	12.3	9.5
El Salvador	SLV	Induction of labor for pregnancies lasting 41+ weeks	1.7	1.3
El Salvador	SLV	Complementary feeding - education only	78.2	60.3
El Salvador	SLV	Complementary feeding - supplementary feeding and education	78.2	60.3
El Salvador	SLV	Vitamin A supplementation	81.0	62.5
El Salvador	SLV	Improved sanitation - Utilization of latrines or toilets	87.4	87.4
El Salvador	SLV	Improved water source	97.4	97.4
El Salvador	SLV	Water connection in the home	87.4	87.4
El Salvador	SLV	Hand washing with soap	91.3	91.3
El Salvador	SLV	Hygienic disposal of children's stools	41.8	41.8
El Salvador	SLV	Injectable antibiotics for neonatal sepsis	97.5	71.3
El Salvador	SLV	ORS - oral rehydration solution	69.8	53.7
El Salvador	SLV	Antibiotics for treatment of dysentery	40.8	31.4
El Salvador	SLV	Zinc for treatment of diarrhea	32.7	25.2
El Salvador	SLV	Oral antibiotics for pneumonia	79.7	61.3
El Salvador	SLV	Vitamin A for treatment of measles	81.0	62.3
El Salvador	SLV	SAM - treatment for severe acute malnutrition	1.9	1.5

El Salvador	SLV	BCG vaccine	81.0	59.2
El Salvador	SLV	Polio vaccine	83.0	60.7
El Salvador	SLV	DPT vaccine	81.0	59.2
El Salvador	SLV	H. influenzae type b vaccine	81.0	74.0
El Salvador	SLV	HepB vaccine	81.0	59.2
El Salvador	SLV	Pneumococcal vaccine	75.0	68.5
El Salvador	SLV	Rotavirus vaccine	82.0	74.9
El Salvador	SLV	Measles vaccine	81.0	59.2
El Salvador	SLV	Global wasting (<-2 SD) rate	2.2	2.6
El Salvador	SLV	Contraceptive prevalence (CPR)	73.05	59.3
Equatorial Guinea	GNQ	TT - Tetanus toxoid vaccination	70.0	51.2
Equatorial Guinea	GNQ	IPTp - Intermittent preventive treatment of malaria during pregnancy	27.6	20.2
Equatorial Guinea	GNQ	Syphilis detection and treatment	21.9	16.0
Equatorial Guinea	GNQ	Iron supplementation in pregnancy	8.7	6.4
Equatorial Guinea	GNQ	Hypertensive disorder case management	16.1	11.8
Equatorial Guinea	GNQ	Diabetes case management	12.5	9.1
Equatorial Guinea	GNQ	Malaria case management	51.9	37.9
Equatorial Guinea	GNQ	MgSO4 management of pre-eclampsia	31.8	23.2
Equatorial Guinea	GNQ	Thermal protection	66.5	51.2
Equatorial Guinea	GNQ	Clean cord care	64.2	49.4
Equatorial Guinea	GNQ	Clean birth environment	55.2	42.5
Equatorial Guinea	GNQ	Immediate drying and additional stimulation	61.6	47.4
Equatorial Guinea	GNQ	Neonatal resuscitation	37.0	28.5
Equatorial Guinea	GNQ	Antibiotics for preterm or prolonged PROM	50.4	38.8
Equatorial Guinea	GNQ	Parenteral administration of anti-convulsants	48.1	37.0
Equatorial Guinea	GNQ	Parenteral administration of uterotonics	60.1	46.2
Equatorial Guinea	GNQ	Parenteral administration of antibiotics	50.4	38.8
Equatorial Guinea	GNQ	Assisted vaginal delivery	17.0	13.1
Equatorial Guinea	GNQ	Manual removal of placenta	25.2	19.4
Equatorial Guinea	GNQ	Removal of retained products of conception	22.3	17.2
Equatorial Guinea	GNQ	Cesarean delivery	5.9	4.5
Equatorial Guinea	GNQ	Blood transfusion	8.5	6.5
Equatorial Guinea	GNQ	Induction of labor for pregnancies lasting 41+ weeks	1.2	0.9
Equatorial Guinea	GNQ	Complementary feeding - education only	40.7	31.4
Equatorial Guinea	GNQ	Complementary feeding - supplementary feeding and education	40.7	31.4
Equatorial Guinea	GNQ	Vitamin A supplementation	30.0	23.1
Equatorial Guinea	GNQ	Improved sanitation - Utilization of latrines or toilets	66.3	66.3
Equatorial Guinea	GNQ	Improved water source	64.7	64.7
Equatorial Guinea	GNQ	Water connection in the home	26.4	26.4
Equatorial Guinea	GNQ	ITN/IRS - Households protected from malaria	63.7	49.2
Equatorial Guinea	GNQ	Injectable antibiotics for neonatal sepsis	67.3	49.2

Equatorial Guinea	GNQ	ORS - oral rehydration solution	40.4	31.1
Equatorial Guinea	GNQ	Antibiotics for treatment of dysentery	9.3	7.2
Equatorial Guinea	GNQ	Zinc for treatment of diarrhea	0.2	0.2
Equatorial Guinea	GNQ	Oral antibiotics for pneumonia	54.3	41.8
Equatorial Guinea	GNQ	Vitamin A for treatment of measles	30.0	23.1
Equatorial Guinea	GNQ	ACTs- Artemisinin compounds for treatment of malaria	9.0	6.9
Equatorial Guinea	GNQ	BCG vaccine	63.0	46.1
Equatorial Guinea	GNQ	Polio vaccine	27.0	19.7
Equatorial Guinea	GNQ	DPT vaccine	25.0	18.3
Equatorial Guinea	GNQ	H. influenzae type b vaccine	25.0	22.8
Equatorial Guinea	GNQ	HepB vaccine	25.0	18.3
Equatorial Guinea	GNQ	Measles vaccine	30.0	21.9
Equatorial Guinea	GNQ	Global wasting (<-2 SD) rate	10.0	12.0
Equatorial Guinea	GNQ	Contraceptive prevalence (CPR)	17.35	14.1
Eritrea	ERI	Safe abortion services	3.3	2.7
Eritrea	ERI	TT - Tetanus toxoid vaccination	99.0	72.4
Eritrea	ERI	Syphilis detection and treatment	17.5	12.8
Eritrea	ERI	Hypertensive disorder case management	13.8	10.1
Eritrea	ERI	Diabetes case management	10.8	7.9
Eritrea	ERI	Malaria case management	44.5	32.5
Eritrea	ERI	MgSO4 management of pre-eclampsia	27.3	20.0
Eritrea	ERI	Thermal protection	33.2	25.5
Eritrea	ERI	Clean cord care	32.1	24.7
Eritrea	ERI	Clean birth environment	27.6	21.2
Eritrea	ERI	Immediate drying and additional stimulation	30.8	23.7
Eritrea	ERI	Neonatal resuscitation	18.5	14.2
Eritrea	ERI	Antibiotics for preterm or prolonged PROM	25.1	19.3
Eritrea	ERI	Parenteral administration of anti-convulsants	24.0	18.5
Eritrea	ERI	Parenteral administration of uterotonics	30.0	23.1
Eritrea	ERI	Parenteral administration of antibiotics	25.1	19.3
Eritrea	ERI	Assisted vaginal delivery	8.5	6.5
Eritrea	ERI	Manual removal of placenta	12.6	9.7
Eritrea	ERI	Removal of retained products of conception	11.1	8.5
Eritrea	ERI	Cesarean delivery	2.9	2.2
Eritrea	ERI	Blood transfusion	4.2	3.2
Eritrea	ERI	Induction of labor for pregnancies lasting 41+ weeks	0.6	0.5
Eritrea	ERI	Complementary feeding - education only	42.5	32.8
Eritrea	ERI	Complementary feeding - supplementary feeding and education	42.5	32.8
Eritrea	ERI	Vitamin A supplementation	51.0	39.4
Eritrea	ERI	Improved sanitation - Utilization of latrines or toilets	11.9	11.9
Eritrea	ERI	Improved water source	51.8	51.8

Eritrea	ERI	Water connection in the home	26.8	26.8
Eritrea	ERI	Hygienic disposal of children's stools	26.8	26.8
Eritrea	ERI	ITN/IRS - Households protected from malaria	70.9	54.7
Eritrea	ERI	Injectable antibiotics for neonatal sepsis	33.6	24.6
Eritrea	ERI	ORS - oral rehydration solution	43.4	33.4
Eritrea	ERI	Vitamin A for treatment of measles	51.0	39.2
Eritrea	ERI	BCG vaccine	97.0	70.9
Eritrea	ERI	Polio vaccine	95.0	69.4
Eritrea	ERI	DPT vaccine	95.0	69.4
Eritrea	ERI	H. influenzae type b vaccine	95.0	86.8
Eritrea	ERI	HepB vaccine	95.0	69.4
Eritrea	ERI	Pneumococcal vaccine	95.0	86.8
Eritrea	ERI	Rotavirus vaccine	96.0	87.7
Eritrea	ERI	Measles vaccine	99.0	72.4
Eritrea	ERI	Global wasting (<-2 SD) rate	7.2	8.6
Eritrea	ERI	Contraceptive prevalence (CPR)	13.8	11.2
Ethiopia	ETH	Safe abortion services	3.3	2.7
Ethiopia	ETH	TT - Tetanus toxoid vaccination	93.0	68.0
Ethiopia	ETH	IPTp - Intermittent preventive treatment of malaria during pregnancy	1.2	0.9
Ethiopia	ETH	Syphilis detection and treatment	15.7	11.5
Ethiopia	ETH	Iron supplementation in pregnancy	5.1	3.7
Ethiopia	ETH	Hypertensive disorder case management	7.9	5.8
Ethiopia	ETH	Diabetes case management	6.1	4.5
Ethiopia	ETH	Malaria case management	25.4	18.6
Ethiopia	ETH	MgSO4 management of pre-eclampsia	15.6	11.4
Ethiopia	ETH	Thermal protection	25.9	19.9
Ethiopia	ETH	Clean cord care	25.0	19.2
Ethiopia	ETH	Clean birth environment	21.5	16.5
Ethiopia	ETH	Immediate drying and additional stimulation	24.0	18.5
Ethiopia	ETH	Neonatal resuscitation	14.4	11.1
Ethiopia	ETH	Antibiotics for preterm or prolonged PROM	19.6	15.1
Ethiopia	ETH	Parenteral administration of anti-convulsants	18.8	14.5
Ethiopia	ETH	Parenteral administration of uterotonics	23.4	18.0
Ethiopia	ETH	Parenteral administration of antibiotics	19.6	15.1
Ethiopia	ETH	Assisted vaginal delivery	6.6	5.1
Ethiopia	ETH	Manual removal of placenta	9.8	7.5
Ethiopia	ETH	Removal of retained products of conception	8.7	6.7
Ethiopia	ETH	Cesarean delivery	2.3	1.8
Ethiopia	ETH	Blood transfusion	3.3	2.5
Ethiopia	ETH	Induction of labor for pregnancies lasting 41+ weeks	0.5	0.4
Ethiopia	ETH	Complementary feeding - education only	13.8	10.6

Ethiopia	ETH	Complementary feeding - supplementary feeding and education	13.8	10.6
Ethiopia	ETH	Vitamin A supplementation	77.0	59.4
Ethiopia	ETH	Improved sanitation - Utilization of latrines or toilets	7.3	7.3
Ethiopia	ETH	Improved water source	41.1	41.1
Ethiopia	ETH	Water connection in the home	14.8	14.8
Ethiopia	ETH	Hand washing with soap	8.3	8.3
Ethiopia	ETH	Hygienic disposal of children's stools	36.9	36.9
Ethiopia	ETH	ITN/IRS - Households protected from malaria	70.5	54.4
Ethiopia	ETH	Injectable antibiotics for neonatal sepsis	26.2	19.2
Ethiopia	ETH	ORS - oral rehydration solution	29.5	22.7
Ethiopia	ETH	Antibiotics for treatment of dysentery	9.3	7.2
Ethiopia	ETH	Zinc for treatment of diarrhea	33.3	25.6
Ethiopia	ETH	Oral antibiotics for pneumonia	29.4	22.6
Ethiopia	ETH	Vitamin A for treatment of measles	77.0	59.3
Ethiopia	ETH	SAM - treatment for severe acute malnutrition	12.0	9.2
Ethiopia	ETH	BCG vaccine	85.0	62.1
Ethiopia	ETH	Polio vaccine	67.0	49.0
Ethiopia	ETH	DPT vaccine	72.0	52.6
Ethiopia	ETH	H. influenzae type b vaccine	72.0	65.8
Ethiopia	ETH	HepB vaccine	72.0	52.6
Ethiopia	ETH	Pneumococcal vaccine	67.0	61.2
Ethiopia	ETH	Rotavirus vaccine	79.0	72.2
Ethiopia	ETH	Measles vaccine	61.0	44.6
Ethiopia	ETH	Global wasting (<-2 SD) rate	10.0	12.0
Ethiopia	ETH	Contraceptive prevalence (CPR)	39.95	32.4
Gabon	GAB	TT - Tetanus toxoid vaccination	85.0	62.1
Gabon	GAB	IPTp - Intermittent preventive treatment of malaria during pregnancy	13.1	9.6
Gabon	GAB	Syphilis detection and treatment	23.4	17.1
Gabon	GAB	Iron supplementation in pregnancy	56.8	41.5
Gabon	GAB	Hypertensive disorder case management	18.5	13.5
Gabon	GAB	Diabetes case management	14.5	10.6
Gabon	GAB	Malaria case management	59.8	43.7
Gabon	GAB	MgSO4 management of pre-eclampsia	36.7	26.8
Gabon	GAB	Thermal protection	89.2	68.6
Gabon	GAB	Clean cord care	86.1	66.3
Gabon	GAB	Clean birth environment	74.0	56.9
Gabon	GAB	Immediate drying and additional stimulation	82.6	63.6
Gabon	GAB	Neonatal resuscitation	49.6	38.2
Gabon	GAB	Antibiotics for preterm or prolonged PROM	67.5	51.9
Gabon	GAB	Parenteral administration of anti-convulsants	64.5	49.6
Gabon	GAB	Parenteral administration of uterotonics	80.6	62.0

Gabon	GAB	Parenteral administration of antibiotics	67.5	51.9
Gabon	GAB	Assisted vaginal delivery	22.8	17.5
Gabon	GAB	Manual removal of placenta	33.7	25.9
Gabon	GAB	Removal of retained products of conception	29.9	23.0
Gabon	GAB	Cesarean delivery	7.9	6.1
Gabon	GAB	Blood transfusion	11.4	8.8
Gabon	GAB	Induction of labor for pregnancies lasting 41+ weeks	1.6	1.2
Gabon	GAB	Complementary feeding - education only	29.9	23.1
Gabon	GAB	Complementary feeding - supplementary feeding and education	29.9	23.1
Gabon	GAB	Improved sanitation - Utilization of latrines or toilets	47.4	47.4
Gabon	GAB	Improved water source	85.8	85.8
Gabon	GAB	Water connection in the home	74.4	74.4
Gabon	GAB	ITN/IRS - Households protected from malaria	38.9	30.0
Gabon	GAB	Injectable antibiotics for neonatal sepsis	90.2	65.9
Gabon	GAB	ORS - oral rehydration solution	26.1	20.1
Gabon	GAB	Antibiotics for treatment of dysentery	0.5	0.4
Gabon	GAB	Oral antibiotics for pneumonia	49.5	38.1
Gabon	GAB	ACTs- Artemisinin compounds for treatment of malaria	4.4	3.4
Gabon	GAB	BCG vaccine	87.0	63.6
Gabon	GAB	Polio vaccine	64.0	46.8
Gabon	GAB	DPT vaccine	70.0	51.2
Gabon	GAB	H. influenzae type b vaccine	70.0	64.0
Gabon	GAB	HepB vaccine	70.0	51.2
Gabon	GAB	Measles vaccine	59.0	43.1
Gabon	GAB	Global wasting (<-2 SD) rate	3.4	4.1
Gabon	GAB	Contraceptive prevalence (CPR)	37.15	30.2
Gambia	GMB	TT - Tetanus toxoid vaccination	92.0	67.3
Gambia	GMB	IPTp - Intermittent preventive treatment of malaria during pregnancy	75.2	55.0
Gambia	GMB	Syphilis detection and treatment	24.5	17.9
Gambia	GMB	Iron supplementation in pregnancy	44.6	32.6
Gambia	GMB	Hypertensive disorder case management	18.2	13.3
Gambia	GMB	Diabetes case management	14.2	10.4
Gambia	GMB	Malaria case management	58.6	42.8
Gambia	GMB	MgSO4 management of pre-eclampsia	35.9	26.2
Gambia	GMB	Thermal protection	80.6	62.0
Gambia	GMB	Clean cord care	77.8	59.9
Gambia	GMB	Clean birth environment	66.8	51.4
Gambia	GMB	Immediate drying and additional stimulation	74.6	57.4
Gambia	GMB	Neonatal resuscitation	44.8	34.5
Gambia	GMB	Antibiotics for preterm or prolonged PROM	61.0	46.9

Gambia	GMB	Parenteral administration of anti-convulsants	58.3	44.9
Gambia	GMB	Parenteral administration of uterotonics	72.8	56.0
Gambia	GMB	Parenteral administration of antibiotics	61.0	46.9
Gambia	GMB	Assisted vaginal delivery	20.6	15.9
Gambia	GMB	Manual removal of placenta	30.5	23.5
Gambia	GMB	Removal of retained products of conception	27.0	20.8
Gambia	GMB	Cesarean delivery	7.1	5.5
Gambia	GMB	Blood transfusion	10.3	7.9
Gambia	GMB	Induction of labor for pregnancies lasting 41+ weeks	1.4	1.1
Gambia	GMB	Complementary feeding - education only	13.0	10.0
Gambia	GMB	Complementary feeding - supplementary feeding and education	13.0	10.0
Gambia	GMB	Vitamin A supplementation	32.0	24.7
Gambia	GMB	Improved sanitation - Utilization of latrines or toilets	39.2	39.2
Gambia	GMB	Improved water source	78.0	78.0
Gambia	GMB	Water connection in the home	56.1	56.1
Gambia	GMB	Hand washing with soap	6.3	6.3
Gambia	GMB	Hygienic disposal of children's stools	76.8	76.8
Gambia	GMB	ITN/IRS - Households protected from malaria	71.2	54.9
Gambia	GMB	Injectable antibiotics for neonatal sepsis	81.5	59.6
Gambia	GMB	ORS - oral rehydration solution	43.9	33.8
Gambia	GMB	Antibiotics for treatment of dysentery	35.7	27.5
Gambia	GMB	Zinc for treatment of diarrhea	23.1	17.8
Gambia	GMB	Oral antibiotics for pneumonia	68.0	52.3
Gambia	GMB	Vitamin A for treatment of measles	32.0	24.6
Gambia	GMB	ACTs- Artemisinin compounds for treatment of malaria	0.8	0.6
Gambia	GMB	SAM - treatment for severe acute malnutrition	25.1	19.3
Gambia	GMB	BCG vaccine	94.0	68.7
Gambia	GMB	Polio vaccine	93.0	68.0
Gambia	GMB	DPT vaccine	93.0	68.0
Gambia	GMB	H. influenzae type b vaccine	93.0	85.0
Gambia	GMB	HepB vaccine	93.0	68.0
Gambia	GMB	Pneumococcal vaccine	93.0	85.0
Gambia	GMB	Rotavirus vaccine	93.0	85.0
Gambia	GMB	Measles vaccine	91.0	66.5
Gambia	GMB	Global wasting (<-2 SD) rate	10.8	12.9
Gambia	GMB	Contraceptive prevalence (CPR)	14.25	11.6
Georgia	GEO	Safe abortion services	40.0	32.5
Georgia	GEO	Syphilis detection and treatment	20.8	15.2
Georgia	GEO	Hypertensive disorder case management	20.2	14.8
Georgia	GEO	Diabetes case management	15.8	11.6
Georgia	GEO	Malaria case management	65.3	47.7

Georgia	GEO	MgSO4 management of pre-eclampsia	40.0	29.2
Georgia	GEO	Thermal protection	98.2	75.6
Georgia	GEO	Clean cord care	94.9	73.0
Georgia	GEO	Clean birth environment	81.5	62.7
Georgia	GEO	Immediate drying and additional stimulation	91.0	70.0
Georgia	GEO	Neonatal resuscitation	54.7	42.1
Georgia	GEO	Antibiotics for preterm or prolonged PROM	74.4	57.3
Georgia	GEO	Parenteral administration of anti-convulsants	71.1	54.7
Georgia	GEO	Parenteral administration of uterotonics	88.8	68.3
Georgia	GEO	Parenteral administration of antibiotics	74.4	57.3
Georgia	GEO	Assisted vaginal delivery	25.1	19.3
Georgia	GEO	Manual removal of placenta	37.2	28.6
Georgia	GEO	Removal of retained products of conception	32.9	25.3
Georgia	GEO	Cesarean delivery	8.7	6.7
Georgia	GEO	Blood transfusion	12.5	9.6
Georgia	GEO	Induction of labor for pregnancies lasting 41+ weeks	1.7	1.3
Georgia	GEO	Complementary feeding - education only	49.9	38.5
Georgia	GEO	Complementary feeding - supplementary feeding and education	49.9	38.5
Georgia	GEO	Improved sanitation - Utilization of latrines or toilets	90.0	90.0
Georgia	GEO	Improved water source	98.4	98.4
Georgia	GEO	Water connection in the home	79.1	79.1
Georgia	GEO	Hygienic disposal of children's stools	57.1	57.1
Georgia	GEO	Injectable antibiotics for neonatal sepsis	99.4	72.7
Georgia	GEO	ORS - oral rehydration solution	42.4	32.6
Georgia	GEO	Zinc for treatment of diarrhea	10.9	8.4
Georgia	GEO	Oral antibiotics for pneumonia	73.6	56.6
Georgia	GEO	BCG vaccine	97.0	70.9
Georgia	GEO	Polio vaccine	93.0	68.0
Georgia	GEO	DPT vaccine	93.0	68.0
Georgia	GEO	H. influenzae type b vaccine	93.0	85.0
Georgia	GEO	HepB vaccine	93.0	68.0
Georgia	GEO	Pneumococcal vaccine	81.0	74.0
Georgia	GEO	Rotavirus vaccine	79.0	72.2
Georgia	GEO	Measles vaccine	98.0	71.6
Georgia	GEO	Global wasting (<-2 SD) rate	3.0	3.6
Georgia	GEO	Contraceptive prevalence (CPR)	47.3	38.4
Ghana	GHA	TT - Tetanus toxoid vaccination	89.0	65.1
Ghana	GHA	IPTp - Intermittent preventive treatment of malaria during pregnancy	78.0	57.0
Ghana	GHA	Syphilis detection and treatment	24.1	17.6
Ghana	GHA	Iron supplementation in pregnancy	59.4	43.4
Ghana	GHA	Hypertensive disorder case management	21.5	15.7

Ghana	GHA	Diabetes case management	16.7	12.2
Ghana	GHA	Malaria case management	69.2	50.6
Ghana	GHA	MgSO4 management of pre-eclampsia	42.4	31.0
Ghana	GHA	Thermal protection	77.8	59.9
Ghana	GHA	Clean cord care	75.1	57.8
Ghana	GHA	Clean birth environment	64.5	49.6
Ghana	GHA	Immediate drying and additional stimulation	72.1	55.5
Ghana	GHA	Neonatal resuscitation	43.3	33.3
Ghana	GHA	Antibiotics for preterm or prolonged PROM	58.9	45.3
Ghana	GHA	Parenteral administration of anti-convulsants	56.3	43.3
Ghana	GHA	Parenteral administration of uterotonics	70.3	54.1
Ghana	GHA	Parenteral administration of antibiotics	58.9	45.3
Ghana	GHA	Assisted vaginal delivery	19.9	15.3
Ghana	GHA	Manual removal of placenta	29.4	22.6
Ghana	GHA	Removal of retained products of conception	26.1	20.1
Ghana	GHA	Cesarean delivery	6.8	5.2
Ghana	GHA	Blood transfusion	9.9	7.6
Ghana	GHA	Induction of labor for pregnancies lasting 41+ weeks	1.4	1.1
Ghana	GHA	Complementary feeding - education only	28.1	21.7
Ghana	GHA	Complementary feeding - supplementary feeding and education	28.1	21.7
Ghana	GHA	Vitamin A supplementation	50.0	38.6
Ghana	GHA	Improved sanitation - Utilization of latrines or toilets	18.5	18.5
Ghana	GHA	Improved water source	81.5	81.5
Ghana	GHA	Water connection in the home	26.6	26.6
Ghana	GHA	Hand washing with soap	23.6	23.6
Ghana	GHA	Hygienic disposal of children's stools	24.5	24.5
Ghana	GHA	ITN/IRS - Households protected from malaria	73.0	56.3
Ghana	GHA	Injectable antibiotics for neonatal sepsis	78.7	57.5
Ghana	GHA	ORS - oral rehydration solution	48.6	37.4
Ghana	GHA	Antibiotics for treatment of dysentery	42.4	32.6
Ghana	GHA	Zinc for treatment of diarrhea	7.4	5.7
Ghana	GHA	Oral antibiotics for pneumonia	52.6	40.5
Ghana	GHA	Vitamin A for treatment of measles	50.0	38.5
Ghana	GHA	ACTs- Artemisinin compounds for treatment of malaria	26.2	20.2
Ghana	GHA	BCG vaccine	98.0	71.6
Ghana	GHA	Polio vaccine	98.0	71.6
Ghana	GHA	DPT vaccine	97.0	70.9
Ghana	GHA	H. influenzae type b vaccine	97.0	88.7
Ghana	GHA	HepB vaccine	97.0	70.9
Ghana	GHA	Pneumococcal vaccine	96.0	87.7
Ghana	GHA	Rotavirus vaccine	94.0	85.9

Ghana	GHA	Meningococcal A	83.0	60.7
Ghana	GHA	Measles vaccine	92.0	67.3
Ghana	GHA	Global wasting (<-2 SD) rate	4.7	5.6
Ghana	GHA	Contraceptive prevalence (CPR)	32.5	26.4
Guatemala	GTM	Safe abortion services	0.6	0.5
Guatemala	GTM	TT - Tetanus toxoid vaccination	90.0	65.8
Guatemala	GTM	Syphilis detection and treatment	22.6	16.5
Guatemala	GTM	Iron supplementation in pregnancy	29.3	21.4
Guatemala	GTM	Hypertensive disorder case management	20.6	15.1
Guatemala	GTM	Diabetes case management	16.1	11.8
Guatemala	GTM	Malaria case management	66.6	48.7
Guatemala	GTM	MgSO4 management of pre-eclampsia	40.8	29.8
Guatemala	GTM	Thermal protection	64.2	49.4
Guatemala	GTM	Clean cord care	62.0	47.7
Guatemala	GTM	Clean birth environment	53.3	41.0
Guatemala	GTM	Immediate drying and additional stimulation	59.5	45.8
Guatemala	GTM	Neonatal resuscitation	35.7	27.5
Guatemala	GTM	Antibiotics for preterm or prolonged PROM	48.6	37.4
Guatemala	GTM	Parenteral administration of anti-convulsants	46.5	35.8
Guatemala	GTM	Parenteral administration of uterotonics	58.1	44.7
Guatemala	GTM	Parenteral administration of antibiotics	48.6	37.4
Guatemala	GTM	Assisted vaginal delivery	16.4	12.6
Guatemala	GTM	Manual removal of placenta	24.3	18.7
Guatemala	GTM	Removal of retained products of conception	21.5	16.5
Guatemala	GTM	Cesarean delivery	5.7	4.4
Guatemala	GTM	Blood transfusion	8.2	6.3
Guatemala	GTM	Induction of labor for pregnancies lasting 41+ weeks	1.1	0.8
Guatemala	GTM	Complementary feeding - education only	62.6	48.3
Guatemala	GTM	Complementary feeding - supplementary feeding and education	62.6	48.3
Guatemala	GTM	Vitamin A supplementation	26.0	20.1
Guatemala	GTM	Improved sanitation - Utilization of latrines or toilets	65.1	65.1
Guatemala	GTM	Improved water source	94.2	94.2
Guatemala	GTM	Water connection in the home	75.5	75.5
Guatemala	GTM	Hand washing with soap	78.5	78.5
Guatemala	GTM	Hygienic disposal of children's stools	38.2	38.2
Guatemala	GTM	Injectable antibiotics for neonatal sepsis	65.0	47.5
Guatemala	GTM	ORS - oral rehydration solution	48.8	37.6
Guatemala	GTM	Antibiotics for treatment of dysentery	7.0	5.4
Guatemala	GTM	Zinc for treatment of diarrhea	1.3	1.0
Guatemala	GTM	Oral antibiotics for pneumonia	52.0	40.0
Guatemala	GTM	Vitamin A for treatment of measles	26.0	20.0

Guatemala	GTM	SAM - treatment for severe acute malnutrition	1.4	1.1
Guatemala	GTM	BCG vaccine	88.0	64.3
Guatemala	GTM	Polio vaccine	85.0	62.1
Guatemala	GTM	DPT vaccine	86.0	62.9
Guatemala	GTM	H. influenzae type b vaccine	86.0	78.6
Guatemala	GTM	HepB vaccine	86.0	62.9
Guatemala	GTM	Pneumococcal vaccine	85.0	77.7
Guatemala	GTM	Rotavirus vaccine	87.0	79.5
Guatemala	GTM	Measles vaccine	86.0	62.9
Guatemala	GTM	Global wasting (<-2 SD) rate	0.8	0.9
Guatemala	GTM	Contraceptive prevalence (CPR)	63.55	51.6
Guinea	GIN	TT - Tetanus toxoid vaccination	80.0	58.5
Guinea	GIN	IPTp - Intermittent preventive treatment of malaria during pregnancy	62.7	45.8
Guinea	GIN	Syphilis detection and treatment	20.6	15.1
Guinea	GIN	Iron supplementation in pregnancy	41.5	30.3
Guinea	GIN	Hypertensive disorder case management	8.5	6.2
Guinea	GIN	Diabetes case management	6.6	4.8
Guinea	GIN	Malaria case management	27.4	20.0
Guinea	GIN	MgSO4 management of pre-eclampsia	16.8	12.3
Guinea	GIN	Thermal protection	52.0	40.0
Guinea	GIN	Clean cord care	50.2	38.6
Guinea	GIN	Clean birth environment	43.1	33.2
Guinea	GIN	Immediate drying and additional stimulation	48.2	37.1
Guinea	GIN	Neonatal resuscitation	28.9	22.2
Guinea	GIN	Antibiotics for preterm or prolonged PROM	39.4	30.3
Guinea	GIN	Parenteral administration of anti-convulsants	37.6	28.9
Guinea	GIN	Parenteral administration of uterotonics	47.0	36.2
Guinea	GIN	Parenteral administration of antibiotics	39.4	30.3
Guinea	GIN	Assisted vaginal delivery	13.3	10.2
Guinea	GIN	Manual removal of placenta	19.7	15.2
Guinea	GIN	Removal of retained products of conception	17.4	13.4
Guinea	GIN	Cesarean delivery	4.6	3.5
Guinea	GIN	Blood transfusion	6.6	5.1
Guinea	GIN	Induction of labor for pregnancies lasting 41+ weeks	0.9	0.7
Guinea	GIN	Complementary feeding - education only	15.9	12.3
Guinea	GIN	Complementary feeding - supplementary feeding and education	15.9	12.3
Guinea	GIN	Vitamin A supplementation	64.0	49.4
Guinea	GIN	Improved sanitation - Utilization of latrines or toilets	22.7	22.7
Guinea	GIN	Improved water source	61.9	61.9
Guinea	GIN	Water connection in the home	17.4	17.4
Guinea	GIN	Hand washing with soap	20.9	20.9

Guinea	GIN	Hygienic disposal of children's stools	57.9	57.9
Guinea	GIN	ITN/IRS - Households protected from malaria	43.9	33.9
Guinea	GIN	Injectable antibiotics for neonatal sepsis	52.6	38.5
Guinea	GIN	ORS - oral rehydration solution	54.9	42.2
Guinea	GIN	Antibiotics for treatment of dysentery	1.5	1.2
Guinea	GIN	Zinc for treatment of diarrhea	26.0	20.0
Guinea	GIN	Oral antibiotics for pneumonia	30.1	23.2
Guinea	GIN	Vitamin A for treatment of measles	64.0	49.2
Guinea	GIN	ACTs- Artemisinin compounds for treatment of malaria	2.1	1.6
Guinea	GIN	SAM - treatment for severe acute malnutrition	13.1	10.1
Guinea	GIN	BCG vaccine	72.0	52.6
Guinea	GIN	Polio vaccine	45.0	32.9
Guinea	GIN	DPT vaccine	45.0	32.9
Guinea	GIN	H. influenzae type b vaccine	45.0	41.1
Guinea	GIN	HepB vaccine	45.0	32.9
Guinea	GIN	Measles vaccine	48.0	35.1
Guinea	GIN	Global wasting (<-2 SD) rate	8.2	9.8
Guinea	GIN	Contraceptive prevalence (CPR)	10.3	8.4
Guinea-Bissau	GNB	TT - Tetanus toxoid vaccination	83.0	60.7
Guinea-Bissau	GNB	IPTp - Intermittent preventive treatment of malaria during pregnancy	45.5	33.3
Guinea-Bissau	GNB	Syphilis detection and treatment	22.9	16.7
Guinea-Bissau	GNB	Hypertensive disorder case management	15.6	11.4
Guinea-Bissau	GNB	Diabetes case management	12.2	8.9
Guinea-Bissau	GNB	Malaria case management	50.3	36.8
Guinea-Bissau	GNB	MgSO4 management of pre-eclampsia	30.8	22.5
Guinea-Bissau	GNB	Thermal protection	43.5	33.5
Guinea-Bissau	GNB	Clean cord care	42.0	32.3
Guinea-Bissau	GNB	Clean birth environment	36.1	27.8
Guinea-Bissau	GNB	Immediate drying and additional stimulation	40.3	31.0
Guinea-Bissau	GNB	Neonatal resuscitation	24.2	18.6
Guinea-Bissau	GNB	Antibiotics for preterm or prolonged PROM	32.9	25.3
Guinea-Bissau	GNB	Parenteral administration of anti-convulsants	31.4	24.2
Guinea-Bissau	GNB	Parenteral administration of uterotonics	39.3	30.2
Guinea-Bissau	GNB	Parenteral administration of antibiotics	32.9	25.3
Guinea-Bissau	GNB	Assisted vaginal delivery	11.1	8.5
Guinea-Bissau	GNB	Manual removal of placenta	16.4	12.6
Guinea-Bissau	GNB	Removal of retained products of conception	14.6	11.2
Guinea-Bissau	GNB	Cesarean delivery	3.8	2.9
Guinea-Bissau	GNB	Blood transfusion	5.5	4.2
Guinea-Bissau	GNB	Induction of labor for pregnancies lasting 41+ weeks	0.8	0.6
Guinea-Bissau	GNB	Complementary feeding - education only	12.7	9.8

Guinea-Bissau	GNB	Complementary feeding - supplementary feeding and education	12.7	9.8
Guinea-Bissau	GNB	Vitamin A supplementation	95.0	73.3
Guinea-Bissau	GNB	Improved sanitation - Utilization of latrines or toilets	20.5	20.5
Guinea-Bissau	GNB	Improved water source	66.6	66.6
Guinea-Bissau	GNB	Water connection in the home	11.9	11.9
Guinea-Bissau	GNB	Hand washing with soap	10.6	10.6
Guinea-Bissau	GNB	Hygienic disposal of children's stools	62.6	62.6
Guinea-Bissau	GNB	ITN/IRS - Households protected from malaria	90.1	69.5
Guinea-Bissau	GNB	Injectable antibiotics for neonatal sepsis	44.0	32.2
Guinea-Bissau	GNB	ORS - oral rehydration solution	35.1	27.0
Guinea-Bissau	GNB	Zinc for treatment of diarrhea	27.8	21.4
Guinea-Bissau	GNB	Oral antibiotics for pneumonia	34.3	26.4
Guinea-Bissau	GNB	Vitamin A for treatment of measles	95.0	73.1
Guinea-Bissau	GNB	ACTs- Artemisinin compounds for treatment of malaria	10.0	7.7
Guinea-Bissau	GNB	SAM - treatment for severe acute malnutrition	2.7	2.1
Guinea-Bissau	GNB	BCG vaccine	91.0	66.5
Guinea-Bissau	GNB	Polio vaccine	89.0	65.1
Guinea-Bissau	GNB	DPT vaccine	88.0	64.3
Guinea-Bissau	GNB	H. influenzae type b vaccine	88.0	80.4
Guinea-Bissau	GNB	HepB vaccine	88.0	64.3
Guinea-Bissau	GNB	Pneumococcal vaccine	88.0	80.4
Guinea-Bissau	GNB	Rotavirus vaccine	88.0	80.4
Guinea-Bissau	GNB	Measles vaccine	86.0	62.9
Guinea-Bissau	GNB	Global wasting (<-2 SD) rate	5.9	7.1
Guinea-Bissau	GNB	Contraceptive prevalence (CPR)	19.15	15.6
Guyana	GUY	Safe abortion services	0.2	0.2
Guyana	GUY	TT - Tetanus toxoid vaccination	99.0	72.4
Guyana	GUY	IPTp - Intermittent preventive treatment of malaria during pregnancy	0.1	0.1
Guyana	GUY	Syphilis detection and treatment	22.4	16.4
Guyana	GUY	Iron supplementation in pregnancy	34.2	25.0
Guyana	GUY	Hypertensive disorder case management	20.8	15.2
Guyana	GUY	Diabetes case management	16.2	11.8
Guyana	GUY	Malaria case management	67.2	49.1
Guyana	GUY	MgSO4 management of pre-eclampsia	41.2	30.1
Guyana	GUY	Thermal protection	91.4	70.3
Guyana	GUY	Clean cord care	88.2	67.9
Guyana	GUY	Clean birth environment	75.8	58.3
Guyana	GUY	Immediate drying and additional stimulation	84.6	65.1
Guyana	GUY	Neonatal resuscitation	50.8	39.1
Guyana	GUY	Antibiotics for preterm or prolonged PROM	69.2	53.2
Guyana	GUY	Parenteral administration of anti-convulsants	66.1	50.9

Guyana	GUY	Parenteral administration of uterotonics	82.6	63.6
Guyana	GUY	Parenteral administration of antibiotics	69.2	53.2
Guyana	GUY	Assisted vaginal delivery	23.4	18.0
Guyana	GUY	Manual removal of placenta	34.6	26.6
Guyana	GUY	Removal of retained products of conception	30.6	23.5
Guyana	GUY	Cesarean delivery	8.0	6.2
Guyana	GUY	Blood transfusion	11.6	8.9
Guyana	GUY	Induction of labor for pregnancies lasting 41+ weeks	1.6	1.2
Guyana	GUY	Complementary feeding - education only	52.6	40.6
Guyana	GUY	Complementary feeding - supplementary feeding and education	52.6	40.6
Guyana	GUY	Improved sanitation - Utilization of latrines or toilets	85.8	85.8
Guyana	GUY	Improved water source	95.5	95.5
Guyana	GUY	Water connection in the home	62.4	62.4
Guyana	GUY	Hand washing with soap	78.8	78.8
Guyana	GUY	Hygienic disposal of children's stools	43.0	43.0
Guyana	GUY	ITN/IRS - Households protected from malaria	5.3	4.1
Guyana	GUY	Injectable antibiotics for neonatal sepsis	92.4	67.5
Guyana	GUY	ORS - oral rehydration solution	42.5	32.7
Guyana	GUY	Antibiotics for treatment of dysentery	12.2	9.4
Guyana	GUY	Zinc for treatment of diarrhea	1.3	1.0
Guyana	GUY	Oral antibiotics for pneumonia	83.6	64.3
Guyana	GUY	BCG vaccine	99.0	72.4
Guyana	GUY	Polio vaccine	94.0	68.7
Guyana	GUY	DPT vaccine	95.0	69.4
Guyana	GUY	H. influenzae type b vaccine	95.0	86.8
Guyana	GUY	HepB vaccine	95.0	69.4
Guyana	GUY	Pneumococcal vaccine	91.0	83.2
Guyana	GUY	Rotavirus vaccine	91.0	83.2
Guyana	GUY	Measles vaccine	98.0	71.6
Guyana	GUY	Global wasting (<-2 SD) rate	6.4	7.7
Guyana	GUY	Contraceptive prevalence (CPR)	41.95	34.1
Haiti	HTI	Safe abortion services	53.7	43.6
Haiti	HTI	TT - Tetanus toxoid vaccination	81.0	59.2
Haiti	HTI	IPTp - Intermittent preventive treatment of malaria during pregnancy	0.2	0.1
Haiti	HTI	Syphilis detection and treatment	19.4	14.2
Haiti	HTI	Iron supplementation in pregnancy	43.2	31.6
Haiti	HTI	Hypertensive disorder case management	15.5	11.3
Haiti	HTI	Diabetes case management	15.0	11.0
Haiti	HTI	Malaria case management	32.8	24.0
Haiti	HTI	MgSO4 management of pre-eclampsia	17.1	12.5
Haiti	HTI	Thermal protection	37.8	29.1

Haiti	HTI	Clean cord care	38.2	29.4
Haiti	HTI	Clean birth environment	33.8	26.0
Haiti	HTI	Immediate drying and additional stimulation	32.6	25.1
Haiti	HTI	Neonatal resuscitation	14.9	11.5
Haiti	HTI	Antibiotics for preterm or prolonged PROM	21.4	16.5
Haiti	HTI	Parenteral administration of anti-convulsants	28.2	21.7
Haiti	HTI	Parenteral administration of uterotonics	25.5	19.6
Haiti	HTI	Parenteral administration of antibiotics	21.4	16.5
Haiti	HTI	Assisted vaginal delivery	7.7	5.9
Haiti	HTI	Manual removal of placenta	14.8	11.4
Haiti	HTI	Removal of retained products of conception	11.0	8.5
Haiti	HTI	Cesarean delivery	9.4	7.2
Haiti	HTI	Blood transfusion	5.0	3.8
Haiti	HTI	Induction of labor for pregnancies lasting 41+ weeks	0.6	0.5
Haiti	HTI	Complementary feeding - education only	25.4	19.6
Haiti	HTI	Complementary feeding - supplementary feeding and education	25.4	19.6
Haiti	HTI	Vitamin A supplementation	17.0	13.1
Haiti	HTI	Improved sanitation - Utilization of latrines or toilets	34.7	34.7
Haiti	HTI	Improved water source	65.5	65.5
Haiti	HTI	Water connection in the home	14.8	14.8
Haiti	HTI	Hand washing with soap	21.5	21.5
Haiti	HTI	Hygienic disposal of children's stools	63.9	63.9
Haiti	HTI	ITN/IRS - Households protected from malaria	32.0	24.7
Haiti	HTI	Injectable antibiotics for neonatal sepsis	39.4	28.8
Haiti	HTI	ORS - oral rehydration solution	39.3	30.2
Haiti	HTI	Antibiotics for treatment of dysentery	16.1	12.4
Haiti	HTI	Zinc for treatment of diarrhea	6.2	4.8
Haiti	HTI	Oral antibiotics for pneumonia	39.4	30.3
Haiti	HTI	Vitamin A for treatment of measles	17.0	13.1
Haiti	HTI	SAM - treatment for severe acute malnutrition	18.1	13.9
Haiti	HTI	BCG vaccine	83.0	60.7
Haiti	HTI	Polio vaccine	64.0	46.8
Haiti	HTI	DPT vaccine	64.0	46.8
Haiti	HTI	H. influenzae type b vaccine	64.0	58.5
Haiti	HTI	HepB vaccine	64.0	46.8
Haiti	HTI	Pneumococcal vaccine	1.0	0.9
Haiti	HTI	Rotavirus vaccine	58.0	53.0
Haiti	HTI	Measles vaccine	69.0	50.4
Haiti	HTI	Global wasting (<-2 SD) rate	3.7	4.5
Haiti	HTI	Contraceptive prevalence (CPR)	37.5	30.5
Honduras	HND	Safe abortion services	0.6	0.5

Honduras	HND	TT - Tetanus toxoid vaccination	99.0	72.4
Honduras	HND	Syphilis detection and treatment	23.9	17.5
Honduras	HND	Iron supplementation in pregnancy	37.4	27.3
Honduras	HND	Hypertensive disorder case management	21.2	15.5
Honduras	HND	Diabetes case management	16.6	12.1
Honduras	HND	Malaria case management	68.5	50.1
Honduras	HND	MgSO4 management of pre-eclampsia	42.0	30.7
Honduras	HND	Thermal protection	81.8	62.9
Honduras	HND	Clean cord care	79.0	60.8
Honduras	HND	Clean birth environment	67.9	52.2
Honduras	HND	Immediate drying and additional stimulation	75.8	58.3
Honduras	HND	Neonatal resuscitation	45.5	35.0
Honduras	HND	Antibiotics for preterm or prolonged PROM	61.9	47.6
Honduras	HND	Parenteral administration of anti-convulsants	59.2	45.6
Honduras	HND	Parenteral administration of uterotonics	73.9	56.9
Honduras	HND	Parenteral administration of antibiotics	61.9	47.6
Honduras	HND	Assisted vaginal delivery	20.9	16.1
Honduras	HND	Manual removal of placenta	30.9	23.8
Honduras	HND	Removal of retained products of conception	27.4	21.1
Honduras	HND	Cesarean delivery	7.2	5.5
Honduras	HND	Blood transfusion	10.4	8.0
Honduras	HND	Induction of labor for pregnancies lasting 41+ weeks	1.5	1.2
Honduras	HND	Complementary feeding - education only	68.2	52.6
Honduras	HND	Complementary feeding - supplementary feeding and education	68.2	52.6
Honduras	HND	Vitamin A supplementation	40.0	30.9
Honduras	HND	Improved sanitation - Utilization of latrines or toilets	81.3	81.3
Honduras	HND	Improved water source	94.8	94.8
Honduras	HND	Water connection in the home	85.5	85.5
Honduras	HND	Hand washing with soap	86.3	86.3
Honduras	HND	Hygienic disposal of children's stools	16.1	16.1
Honduras	HND	Injectable antibiotics for neonatal sepsis	82.7	60.5
Honduras	HND	ORS - oral rehydration solution	59.7	45.9
Honduras	HND	Antibiotics for treatment of dysentery	43.6	33.6
Honduras	HND	Zinc for treatment of diarrhea	0.4	0.3
Honduras	HND	Oral antibiotics for pneumonia	63.9	49.2
Honduras	HND	Vitamin A for treatment of measles	40.0	30.8
Honduras	HND	BCG vaccine	94.0	68.7
Honduras	HND	Polio vaccine	90.0	65.8
Honduras	HND	DPT vaccine	90.0	65.8
Honduras	HND	H. influenzae type b vaccine	90.0	82.3
Honduras	HND	HepB vaccine	90.0	65.8

Honduras	HND	Pneumococcal vaccine	90.0	82.3
Honduras	HND	Rotavirus vaccine	91.0	83.2
Honduras	HND	Measles vaccine	89.0	65.1
Honduras	HND	Global wasting (<-2 SD) rate	1.4	1.6
Honduras	HND	Contraceptive prevalence (CPR)	74.9	60.8
India	IND	Safe abortion services	35.1	28.5
India	IND	TT - Tetanus toxoid vaccination	90.0	65.8
India	IND	Syphilis detection and treatment	19.7	14.4
India	IND	Iron supplementation in pregnancy	38.8	28.4
India	IND	Hypertensive disorder case management	12.2	8.9
India	IND	Diabetes case management	9.5	6.9
India	IND	Malaria case management	39.3	28.7
India	IND	MgSO4 management of pre-eclampsia	24.1	17.6
India	IND	Thermal protection	78.0	60.0
India	IND	Clean cord care	75.3	57.9
India	IND	Clean birth environment	64.7	49.8
India	IND	Immediate drying and additional stimulation	72.3	55.6
India	IND	Neonatal resuscitation	43.4	33.4
India	IND	Antibiotics for preterm or prolonged PROM	59.1	45.5
India	IND	Parenteral administration of anti-convulsants	56.4	43.4
India	IND	Parenteral administration of uterotonics	70.5	54.2
India	IND	Parenteral administration of antibiotics	59.1	45.5
India	IND	Assisted vaginal delivery	20.0	15.4
India	IND	Manual removal of placenta	29.5	22.7
India	IND	Removal of retained products of conception	26.2	20.2
India	IND	Cesarean delivery	6.9	5.3
India	IND	Blood transfusion	9.9	7.6
India	IND	Induction of labor for pregnancies lasting 41+ weeks	1.4	1.1
India	IND	Complementary feeding - education only	22.0	17.0
India	IND	Complementary feeding - supplementary feeding and education	22.0	17.0
India	IND	Vitamin A supplementation	71.0	54.8
India	IND	Improved sanitation - Utilization of latrines or toilets	59.5	59.5
India	IND	Improved water source	92.7	92.7
India	IND	Water connection in the home	40.5	40.5
India	IND	Hand washing with soap	67.6	67.6
India	IND	Hygienic disposal of children's stools	26.0	26.0
India	IND	Injectable antibiotics for neonatal sepsis	78.9	57.7
India	IND	ORS - oral rehydration solution	50.6	38.9
India	IND	Antibiotics for treatment of dysentery	21.8	16.8
India	IND	Zinc for treatment of diarrhea	20.3	15.6
India	IND	Oral antibiotics for pneumonia	78.1	60.1

India	IND	Vitamin A for treatment of measles	71.0	54.6
India	IND	ACTs- Artemisinin compounds for treatment of malaria	1.6	1.2
India	IND	BCG vaccine	92.0	67.3
India	IND	Polio vaccine	89.0	65.1
India	IND	DPT vaccine	89.0	65.1
India	IND	H. influenzae type b vaccine	89.0	81.3
India	IND	HepB vaccine	89.0	65.1
India	IND	Pneumococcal vaccine	6.0	5.5
India	IND	Rotavirus vaccine	35.0	32.0
India	IND	Measles vaccine	90.0	65.8
India	IND	Global wasting (<-2 SD) rate	21.0	25.2
India	IND	Contraceptive prevalence (CPR)	56.65	46.0
Indonesia	IDN	Safe abortion services	39.0	31.7
Indonesia	IDN	TT - Tetanus toxoid vaccination	90.0	65.8
Indonesia	IDN	Syphilis detection and treatment	23.7	17.3
Indonesia	IDN	Iron supplementation in pregnancy	32.9	24.1
Indonesia	IDN	Hypertensive disorder case management	21.0	15.4
Indonesia	IDN	Diabetes case management	16.4	12.0
Indonesia	IDN	Malaria case management	67.7	49.5
Indonesia	IDN	MgSO4 management of pre-eclampsia	41.5	30.3
Indonesia	IDN	Thermal protection	62.9	48.4
Indonesia	IDN	Clean cord care	60.7	46.7
Indonesia	IDN	Clean birth environment	52.2	40.2
Indonesia	IDN	Immediate drying and additional stimulation	58.2	44.8
Indonesia	IDN	Neonatal resuscitation	35.0	26.9
Indonesia	IDN	Antibiotics for preterm or prolonged PROM	47.6	36.6
Indonesia	IDN	Parenteral administration of anti-convulsants	45.5	35.0
Indonesia	IDN	Parenteral administration of uterotonics	56.8	43.7
Indonesia	IDN	Parenteral administration of antibiotics	47.6	36.6
Indonesia	IDN	Assisted vaginal delivery	16.1	12.4
Indonesia	IDN	Manual removal of placenta	23.8	18.3
Indonesia	IDN	Removal of retained products of conception	21.1	16.2
Indonesia	IDN	Cesarean delivery	5.5	4.2
Indonesia	IDN	Blood transfusion	8.0	6.2
Indonesia	IDN	Induction of labor for pregnancies lasting 41+ weeks	1.1	0.8
Indonesia	IDN	Complementary feeding - education only	58.2	44.9
Indonesia	IDN	Complementary feeding - supplementary feeding and education	58.2	44.9
Indonesia	IDN	Vitamin A supplementation	62.0	47.8
Indonesia	IDN	Improved sanitation - Utilization of latrines or toilets	73.1	73.1
Indonesia	IDN	Improved water source	89.3	89.3
Indonesia	IDN	Water connection in the home	16.3	16.3

Indonesia	IDN	Hand washing with soap	75.6	75.6
Indonesia	IDN	Hygienic disposal of children's stools	43.7	43.7
Indonesia	IDN	ITN/IRS - Households protected from malaria	3.1	2.4
Indonesia	IDN	Injectable antibiotics for neonatal sepsis	63.6	46.5
Indonesia	IDN	ORS - oral rehydration solution	38.8	29.9
Indonesia	IDN	Antibiotics for treatment of dysentery	19.7	15.2
Indonesia	IDN	Zinc for treatment of diarrhea	1.1	0.8
Indonesia	IDN	Oral antibiotics for pneumonia	75.3	57.9
Indonesia	IDN	Vitamin A for treatment of measles	62.0	47.7
Indonesia	IDN	BCG vaccine	81.0	59.2
Indonesia	IDN	Polio vaccine	80.0	58.5
Indonesia	IDN	DPT vaccine	79.0	57.8
Indonesia	IDN	H. influenzae type b vaccine	79.0	72.2
Indonesia	IDN	HepB vaccine	79.0	57.8
Indonesia	IDN	Pneumococcal vaccine	8.0	7.3
Indonesia	IDN	Measles vaccine	75.0	54.8
Indonesia	IDN	Global wasting (<-2 SD) rate	10.6	12.7
Indonesia	IDN	Contraceptive prevalence (CPR)	63.2	51.3
Iraq	IRQ	Safe abortion services	40.0	32.5
Iraq	IRQ	TT - Tetanus toxoid vaccination	75.0	54.8
Iraq	IRQ	Syphilis detection and treatment	21.7	15.9
Iraq	IRQ	Hypertensive disorder case management	16.3	11.9
Iraq	IRQ	Diabetes case management	12.7	9.3
Iraq	IRQ	Malaria case management	52.7	38.5
Iraq	IRQ	MgSO4 management of pre-eclampsia	32.3	23.6
Iraq	IRQ	Thermal protection	85.6	65.9
Iraq	IRQ	Clean cord care	82.6	63.6
Iraq	IRQ	Clean birth environment	71.0	54.6
Iraq	IRQ	Immediate drying and additional stimulation	79.3	61.0
Iraq	IRQ	Neonatal resuscitation	47.6	36.6
Iraq	IRQ	Antibiotics for preterm or prolonged PROM	64.8	49.9
Iraq	IRQ	Parenteral administration of anti-convulsants	61.9	47.6
Iraq	IRQ	Parenteral administration of uterotonics	77.4	59.6
Iraq	IRQ	Parenteral administration of antibiotics	64.8	49.9
Iraq	IRQ	Assisted vaginal delivery	21.9	16.9
Iraq	IRQ	Manual removal of placenta	32.4	24.9
Iraq	IRQ	Removal of retained products of conception	28.7	22.1
Iraq	IRQ	Cesarean delivery	7.5	5.8
Iraq	IRQ	Blood transfusion	10.9	8.4
Iraq	IRQ	Induction of labor for pregnancies lasting 41+ weeks	1.5	1.2
Iraq	IRQ	Complementary feeding - education only	60.1	46.4
Iraq	IRQ	Complementary feeding - supplementary feeding and education	60.1	46.4

Iraq	IRQ	Improved sanitation - Utilization of latrines or toilets	94.1	94.1
Iraq	IRQ	Improved water source	96.5	96.5
Iraq	IRQ	Water connection in the home	76.8	76.8
Iraq	IRQ	Hand washing with soap	97.7	97.7
Iraq	IRQ	Hygienic disposal of children's stools	15.8	15.8
Iraq	IRQ	Injectable antibiotics for neonatal sepsis	86.6	63.3
Iraq	IRQ	ORS - oral rehydration solution	25.4	19.5
Iraq	IRQ	Zinc for treatment of diarrhea	7.4	5.7
Iraq	IRQ	Oral antibiotics for pneumonia	40.3	31.0
Iraq	IRQ	BCG vaccine	95.0	69.4
Iraq	IRQ	Polio vaccine	71.0	51.9
Iraq	IRQ	DPT vaccine	84.0	61.4
Iraq	IRQ	H. influenzae type b vaccine	84.0	76.8
Iraq	IRQ	HepB vaccine	84.0	61.4
Iraq	IRQ	Pneumococcal vaccine	32.0	29.2
Iraq	IRQ	Rotavirus vaccine	60.0	54.8
Iraq	IRQ	Measles vaccine	83.0	60.7
Iraq	IRQ	Global wasting (<-2 SD) rate	3.1	3.8
Iraq	IRQ	Contraceptive prevalence (CPR)	47.05	38.2
Jamaica	JAM	Safe abortion services	53.7	43.6
Jamaica	JAM	TT - Tetanus toxoid vaccination	90.0	65.8
Jamaica	JAM	Syphilis detection and treatment	24.1	17.6
Jamaica	JAM	Hypertensive disorder case management	20.6	15.1
Jamaica	JAM	Diabetes case management	16.0	11.7
Jamaica	JAM	Malaria case management	66.3	48.5
Jamaica	JAM	MgSO4 management of pre-eclampsia	40.7	29.8
Jamaica	JAM	Thermal protection	97.4	74.9
Jamaica	JAM	Clean cord care	94.0	72.3
Jamaica	JAM	Clean birth environment	80.8	62.2
Jamaica	JAM	Immediate drying and additional stimulation	90.2	69.4
Jamaica	JAM	Neonatal resuscitation	54.2	41.7
Jamaica	JAM	Antibiotics for preterm or prolonged PROM	73.8	56.8
Jamaica	JAM	Parenteral administration of anti-convulsants	70.5	54.2
Jamaica	JAM	Parenteral administration of uterotonics	88.1	67.8
Jamaica	JAM	Parenteral administration of antibiotics	73.8	56.8
Jamaica	JAM	Assisted vaginal delivery	24.9	19.2
Jamaica	JAM	Manual removal of placenta	36.9	28.4
Jamaica	JAM	Removal of retained products of conception	32.7	25.2
Jamaica	JAM	Cesarean delivery	8.6	6.6
Jamaica	JAM	Blood transfusion	12.4	9.5
Jamaica	JAM	Induction of labor for pregnancies lasting 41+ weeks	1.7	1.3

Jamaica	JAM	Improved sanitation - Utilization of latrines or toilets	87.3	87.3
Jamaica	JAM	Improved water source	90.6	90.6
Jamaica	JAM	Water connection in the home	76.0	76.0
Jamaica	JAM	Hand washing with soap	74.7	74.7
Jamaica	JAM	Hygienic disposal of children's stools	28.2	28.2
Jamaica	JAM	Injectable antibiotics for neonatal sepsis	98.6	72.1
Jamaica	JAM	ORS - oral rehydration solution	64.1	49.3
Jamaica	JAM	Oral antibiotics for pneumonia	82.3	63.3
Jamaica	JAM	BCG vaccine	93.0	68.0
Jamaica	JAM	Polio vaccine	98.0	71.6
Jamaica	JAM	DPT vaccine	97.0	70.9
Jamaica	JAM	H. influenzae type b vaccine	98.0	89.6
Jamaica	JAM	HepB vaccine	97.0	70.9
Jamaica	JAM	Measles vaccine	89.0	65.1
Jamaica	JAM	Global wasting (<-2 SD) rate	2.4	2.9
Jamaica	JAM	Contraceptive prevalence (CPR)	70.95	57.6
Jordan	JOR	Safe abortion services	40.0	32.5
Jordan	JOR	TT - Tetanus toxoid vaccination	90.0	65.8
Jordan	JOR	Syphilis detection and treatment	24.2	17.7
Jordan	JOR	Iron supplementation in pregnancy	48.9	35.7
Jordan	JOR	Hypertensive disorder case management	22.2	16.2
Jordan	JOR	Diabetes case management	17.3	12.6
Jordan	JOR	Malaria case management	71.6	52.3
Jordan	JOR	MgSO4 management of pre-eclampsia	43.9	32.1
Jordan	JOR	Thermal protection	96.9	74.6
Jordan	JOR	Clean cord care	93.6	72.0
Jordan	JOR	Clean birth environment	80.4	61.9
Jordan	JOR	Immediate drying and additional stimulation	89.8	69.1
Jordan	JOR	Neonatal resuscitation	53.9	41.5
Jordan	JOR	Antibiotics for preterm or prolonged PROM	73.4	56.5
Jordan	JOR	Parenteral administration of anti-convulsants	70.1	53.9
Jordan	JOR	Parenteral administration of uterotonics	87.6	67.4
Jordan	JOR	Parenteral administration of antibiotics	73.4	56.5
Jordan	JOR	Assisted vaginal delivery	24.8	19.1
Jordan	JOR	Manual removal of placenta	36.7	28.2
Jordan	JOR	Removal of retained products of conception	32.5	25.0
Jordan	JOR	Cesarean delivery	8.5	6.5
Jordan	JOR	Blood transfusion	12.3	9.5
Jordan	JOR	Induction of labor for pregnancies lasting 41+ weeks	1.7	1.3
Jordan	JOR	Complementary feeding - education only	51.4	39.7
Jordan	JOR	Complementary feeding - supplementary feeding and education	51.4	39.7

Jordan	JOR	Vitamin A supplementation	17.0	13.1
Jordan	JOR	Improved sanitation - Utilization of latrines or toilets	97.3	97.3
Jordan	JOR	Improved water source	98.9	98.9
Jordan	JOR	Water connection in the home	86.9	86.9
Jordan	JOR	Injectable antibiotics for neonatal sepsis	98.1	71.7
Jordan	JOR	ORS - oral rehydration solution	44.4	34.2
Jordan	JOR	Antibiotics for treatment of dysentery	54.5	41.9
Jordan	JOR	Oral antibiotics for pneumonia	60.9	46.9
Jordan	JOR	Vitamin A for treatment of measles	17.0	13.1
Jordan	JOR	BCG vaccine	94.0	68.7
Jordan	JOR	Polio vaccine	92.0	67.3
Jordan	JOR	DPT vaccine	96.0	70.2
Jordan	JOR	H. influenzae type b vaccine	96.0	87.7
Jordan	JOR	HepB vaccine	96.0	70.2
Jordan	JOR	Rotavirus vaccine	93.0	85.0
Jordan	JOR	Measles vaccine	92.0	67.3
Jordan	JOR	Global wasting (<-2 SD) rate	2.5	3.0
Jordan	JOR	Contraceptive prevalence (CPR)	53.2	43.2
Kazakhstan	KAZ	Safe abortion services	35.1	28.5
Kazakhstan	KAZ	Syphilis detection and treatment	24.5	17.9
Kazakhstan	KAZ	Hypertensive disorder case management	22.8	16.7
Kazakhstan	KAZ	Diabetes case management	17.8	13.0
Kazakhstan	KAZ	Malaria case management	73.7	53.9
Kazakhstan	KAZ	MgSO4 management of pre-eclampsia	45.2	33.0
Kazakhstan	KAZ	Thermal protection	98.2	75.6
Kazakhstan	KAZ	Clean cord care	94.8	72.9
Kazakhstan	KAZ	Clean birth environment	81.5	62.7
Kazakhstan	KAZ	Immediate drying and additional stimulation	91.0	70.0
Kazakhstan	KAZ	Neonatal resuscitation	54.6	42.0
Kazakhstan	KAZ	Antibiotics for preterm or prolonged PROM	74.3	57.2
Kazakhstan	KAZ	Parenteral administration of anti-convulsants	71.0	54.6
Kazakhstan	KAZ	Parenteral administration of uterotonics	88.8	68.3
Kazakhstan	KAZ	Parenteral administration of antibiotics	74.3	57.2
Kazakhstan	KAZ	Assisted vaginal delivery	25.1	19.3
Kazakhstan	KAZ	Manual removal of placenta	37.2	28.6
Kazakhstan	KAZ	Removal of retained products of conception	32.9	25.3
Kazakhstan	KAZ	Cesarean delivery	8.6	6.6
Kazakhstan	KAZ	Blood transfusion	12.5	9.6
Kazakhstan	KAZ	Induction of labor for pregnancies lasting 41+ weeks	1.7	1.3
Kazakhstan	KAZ	Complementary feeding - education only	64.2	49.5
Kazakhstan	KAZ	Complementary feeding - supplementary feeding and education	64.2	49.5

Kazakhstan	KAZ	Improved sanitation - Utilization of latrines or toilets	97.9	97.9
Kazakhstan	KAZ	Improved water source	95.6	95.6
Kazakhstan	KAZ	Water connection in the home	77.3	77.3
Kazakhstan	KAZ	Hand washing with soap	99.0	99.0
Kazakhstan	KAZ	Hygienic disposal of children's stools	66.7	66.7
Kazakhstan	KAZ	Injectable antibiotics for neonatal sepsis	99.3	72.6
Kazakhstan	KAZ	ORS - oral rehydration solution	61.8	47.6
Kazakhstan	KAZ	Antibiotics for treatment of dysentery	17.2	13.2
Kazakhstan	KAZ	Oral antibiotics for pneumonia	81.2	62.5
Kazakhstan	KAZ	BCG vaccine	95.0	69.4
Kazakhstan	KAZ	Polio vaccine	98.0	71.6
Kazakhstan	KAZ	DPT vaccine	98.0	71.6
Kazakhstan	KAZ	H. influenzae type b vaccine	98.0	89.6
Kazakhstan	KAZ	HepB vaccine	98.0	71.6
Kazakhstan	KAZ	Pneumococcal vaccine	95.0	86.8
Kazakhstan	KAZ	Measles vaccine	99.0	72.4
Kazakhstan	KAZ	Global wasting (<-2 SD) rate	3.3	3.9
Kazakhstan	KAZ	Contraceptive prevalence (CPR)	56.2	45.6
Kenya	KEN	Safe abortion services	3.3	2.7
Kenya	KEN	TT - Tetanus toxoid vaccination	88.0	64.3
Kenya	KEN	IPTp - Intermittent preventive treatment of malaria during pregnancy	34.7	25.4
Kenya	KEN	Syphilis detection and treatment	52.9	38.7
Kenya	KEN	Iron supplementation in pregnancy	7.5	5.5
Kenya	KEN	Hypertensive disorder case management	14.0	10.2
Kenya	KEN	Diabetes case management	12.5	9.1
Kenya	KEN	Malaria case management	32.7	23.9
Kenya	KEN	MgSO4 management of pre-eclampsia	24.6	18.0
Kenya	KEN	Thermal protection	60.7	46.7
Kenya	KEN	Clean cord care	58.7	45.2
Kenya	KEN	Clean birth environment	50.4	38.8
Kenya	KEN	Immediate drying and additional stimulation	53.6	41.2
Kenya	KEN	Neonatal resuscitation	50.9	39.2
Kenya	KEN	Antibiotics for preterm or prolonged PROM	47.0	36.2
Kenya	KEN	Parenteral administration of anti-convulsants	60.0	46.2
Kenya	KEN	Parenteral administration of uterotonics	46.6	35.9
Kenya	KEN	Parenteral administration of antibiotics	47.0	36.2
Kenya	KEN	Assisted vaginal delivery	5.2	4.0
Kenya	KEN	Manual removal of placenta	42.8	32.9
Kenya	KEN	Removal of retained products of conception	31.3	24.1
Kenya	KEN	Cesarean delivery	11.3	8.7
Kenya	KEN	Blood transfusion	7.7	5.9

Kenya	KEN	Induction of labor for pregnancies lasting 41+ weeks	1.1	0.8
Kenya	KEN	Complementary feeding - education only	40.9	31.6
Kenya	KEN	Complementary feeding - supplementary feeding and education	40.9	31.6
Kenya	KEN	Vitamin A supplementation	44.0	34.0
Kenya	KEN	Improved sanitation - Utilization of latrines or toilets	29.1	29.1
Kenya	KEN	Improved water source	58.9	58.9
Kenya	KEN	Water connection in the home	18.9	18.9
Kenya	KEN	Hand washing with soap	25.2	25.2
Kenya	KEN	Hygienic disposal of children's stools	70.2	70.2
Kenya	KEN	ITN/IRS - Households protected from malaria	62.5	48.2
Kenya	KEN	Injectable antibiotics for neonatal sepsis	61.5	45.0
Kenya	KEN	ORS - oral rehydration solution	53.8	41.4
Kenya	KEN	Antibiotics for treatment of dysentery	15.7	12.1
Kenya	KEN	Zinc for treatment of diarrhea	8.1	6.2
Kenya	KEN	Oral antibiotics for pneumonia	65.7	50.6
Kenya	KEN	Vitamin A for treatment of measles	44.0	33.9
Kenya	KEN	ACTs- Artemisinin compounds for treatment of malaria	15.0	11.5
Kenya	KEN	SAM - treatment for severe acute malnutrition	7.2	5.5
Kenya	KEN	BCG vaccine	95.0	69.4
Kenya	KEN	Polio vaccine	81.0	59.2
Kenya	KEN	DPT vaccine	92.0	67.3
Kenya	KEN	H. influenzae type b vaccine	92.0	84.1
Kenya	KEN	HepB vaccine	92.0	67.3
Kenya	KEN	Pneumococcal vaccine	81.0	74.0
Kenya	KEN	Rotavirus vaccine	78.0	71.3
Kenya	KEN	Measles vaccine	89.0	65.1
Kenya	KEN	Global wasting (<-2 SD) rate	4.2	5.0
Kenya	KEN	Contraceptive prevalence (CPR)	61.55	50.0
Kyrgyzstan	KGZ	Safe abortion services	35.1	28.5
Kyrgyzstan	KGZ	Syphilis detection and treatment	24.7	18.1
Kyrgyzstan	KGZ	Iron supplementation in pregnancy	2.3	1.7
Kyrgyzstan	KGZ	Hypertensive disorder case management	24.0	17.5
Kyrgyzstan	KGZ	Diabetes case management	18.7	13.7
Kyrgyzstan	KGZ	Malaria case management	77.4	56.6
Kyrgyzstan	KGZ	MgSO4 management of pre-eclampsia	47.4	34.7
Kyrgyzstan	KGZ	Thermal protection	98.4	75.7
Kyrgyzstan	KGZ	Clean cord care	95.0	73.1
Kyrgyzstan	KGZ	Clean birth environment	81.7	62.9
Kyrgyzstan	KGZ	Immediate drying and additional stimulation	91.2	70.2
Kyrgyzstan	KGZ	Neonatal resuscitation	54.8	42.2
Kyrgyzstan	KGZ	Antibiotics for preterm or prolonged PROM	74.5	57.3

Kyrgyzstan	KGZ	Parenteral administration of anti-convulsants	71.2	54.8
Kyrgyzstan	KGZ	Parenteral administration of uterotonics	89.0	68.5
Kyrgyzstan	KGZ	Parenteral administration of antibiotics	74.5	57.3
Kyrgyzstan	KGZ	Assisted vaginal delivery	25.2	19.4
Kyrgyzstan	KGZ	Manual removal of placenta	37.3	28.7
Kyrgyzstan	KGZ	Removal of retained products of conception	33.0	25.4
Kyrgyzstan	KGZ	Cesarean delivery	8.7	6.7
Kyrgyzstan	KGZ	Blood transfusion	12.5	9.6
Kyrgyzstan	KGZ	Induction of labor for pregnancies lasting 41+ weeks	1.8	1.4
Kyrgyzstan	KGZ	Complementary feeding - education only	61.7	47.6
Kyrgyzstan	KGZ	Complementary feeding - supplementary feeding and education	61.7	47.6
Kyrgyzstan	KGZ	Vitamin A supplementation	97.0	74.8
Kyrgyzstan	KGZ	Improved sanitation - Utilization of latrines or toilets	96.5	96.5
Kyrgyzstan	KGZ	Improved water source	87.5	87.5
Kyrgyzstan	KGZ	Water connection in the home	78.1	78.1
Kyrgyzstan	KGZ	Hand washing with soap	98.1	98.1
Kyrgyzstan	KGZ	Hygienic disposal of children's stools	75.8	75.8
Kyrgyzstan	KGZ	Injectable antibiotics for neonatal sepsis	99.6	72.8
Kyrgyzstan	KGZ	ORS - oral rehydration solution	36.4	28.0
Kyrgyzstan	KGZ	Zinc for treatment of diarrhea	21.8	16.8
Kyrgyzstan	KGZ	Oral antibiotics for pneumonia	59.7	45.9
Kyrgyzstan	KGZ	Vitamin A for treatment of measles	97.0	74.6
Kyrgyzstan	KGZ	BCG vaccine	97.0	70.9
Kyrgyzstan	KGZ	Polio vaccine	92.0	67.3
Kyrgyzstan	KGZ	DPT vaccine	94.0	68.7
Kyrgyzstan	KGZ	H. influenzae type b vaccine	92.0	84.1
Kyrgyzstan	KGZ	HepB vaccine	92.0	67.3
Kyrgyzstan	KGZ	Pneumococcal vaccine	92.0	84.1
Kyrgyzstan	KGZ	Measles vaccine	96.0	70.2
Kyrgyzstan	KGZ	Global wasting (<-2 SD) rate	2.8	3.4
Kyrgyzstan	KGZ	Contraceptive prevalence (CPR)	41.6	33.8
Lao People's Democratic Republic	LAO	Safe abortion services	39.0	31.7
Lao People's Democratic Republic	LAO	TT - Tetanus toxoid vaccination	90.0	65.8
Lao People's Democratic Republic	LAO	IPTp - Intermittent preventive treatment of malaria during pregnancy	2.5	1.8
Lao People's Democratic Republic	LAO	Syphilis detection and treatment	19.4	14.2
Lao People's Democratic Republic	LAO	Iron supplementation in pregnancy	55.1	40.3
Lao People's Democratic Republic	LAO	Hypertensive disorder case management	14.9	10.9

Lao People's Democratic Republic	LAO	Diabetes case management	11.7	8.6
Lao People's Democratic Republic	LAO	Malaria case management	48.2	35.2
Lao People's Democratic Republic	LAO	MgSO4 management of pre-eclampsia	29.6	21.6
Lao People's Democratic Republic	LAO	Thermal protection	63.7	49.0
Lao People's Democratic Republic	LAO	Clean cord care	61.5	47.3
Lao People's Democratic Republic	LAO	Clean birth environment	52.8	40.6
Lao People's Democratic Republic	LAO	Immediate drying and additional stimulation	59.0	45.4
Lao People's Democratic Republic	LAO	Neonatal resuscitation	35.4	27.2
Lao People's Democratic Republic	LAO	Antibiotics for preterm or prolonged PROM	48.2	37.1
Lao People's Democratic Republic	LAO	Parenteral administration of anti-convulsants	46.0	35.4
Lao People's Democratic Republic	LAO	Parenteral administration of uterotonics	57.5	44.2
Lao People's Democratic Republic	LAO	Parenteral administration of antibiotics	48.2	37.1
Lao People's Democratic Republic	LAO	Assisted vaginal delivery	16.3	12.5
Lao People's Democratic Republic	LAO	Manual removal of placenta	24.1	18.5
Lao People's Democratic Republic	LAO	Removal of retained products of conception	21.3	16.4
Lao People's Democratic Republic	LAO	Cesarean delivery	5.6	4.3
Lao People's Democratic Republic	LAO	Blood transfusion	8.1	6.2
Lao People's Democratic Republic	LAO	Induction of labor for pregnancies lasting 41+ weeks	1.1	0.8
Lao People's Democratic Republic	LAO	Complementary feeding - education only	33.5	25.8
Lao People's Democratic Republic	LAO	Complementary feeding - supplementary feeding and education	33.5	25.8
Lao People's Democratic Republic	LAO	Vitamin A supplementation	57.0	44.0
Lao People's Democratic Republic	LAO	Improved sanitation - Utilization of latrines or toilets	74.5	74.5
Lao People's Democratic Republic	LAO	Improved water source	82.1	82.1
Lao People's Democratic Republic	LAO	Water connection in the home	40.6	40.6
Lao People's Democratic Republic	LAO	Hand washing with soap	91.8	91.8
Lao People's Democratic Republic	LAO	Hygienic disposal of children's stools	27.9	27.9
Lao People's Democratic Republic	LAO	ITN/IRS - Households protected from malaria	62.5	48.2

Lao People's Democratic Republic	LAO	Injectable antibiotics for neonatal sepsis	64.4	47.1
Lao People's Democratic Republic	LAO	ORS - oral rehydration solution	56.1	43.2
Lao People's Democratic Republic	LAO	Zinc for treatment of diarrhea	15.2	11.7
Lao People's Democratic Republic	LAO	Oral antibiotics for pneumonia	38.5	29.6
Lao People's Democratic Republic	LAO	Vitamin A for treatment of measles	57.0	43.9
Lao People's Democratic Republic	LAO	ACTs- Artemisinin compounds for treatment of malaria	3.5	2.7
Lao People's Democratic Republic	LAO	SAM - treatment for severe acute malnutrition	2.4	1.8
Lao People's Democratic Republic	LAO	BCG vaccine	79.0	57.8
Lao People's Democratic Republic	LAO	Polio vaccine	67.0	49.0
Lao People's Democratic Republic	LAO	DPT vaccine	68.0	49.7
Lao People's Democratic Republic	LAO	H. influenzae type b vaccine	68.0	62.1
Lao People's Democratic Republic	LAO	HepB vaccine	68.0	49.7
Lao People's Democratic Republic	LAO	Pneumococcal vaccine	56.0	51.2
Lao People's Democratic Republic	LAO	Measles vaccine	82.0	59.9
Lao People's Democratic Republic	LAO	Global wasting (<-2 SD) rate	9.1	10.9
Lao People's Democratic Republic	LAO	Contraceptive prevalence (CPR)	57.2	46.5
Lesotho	LSO	Safe abortion services	42.5	34.5
Lesotho	LSO	TT - Tetanus toxoid vaccination	85.0	62.1
Lesotho	LSO	Syphilis detection and treatment	23.5	17.2
Lesotho	LSO	Iron supplementation in pregnancy	51.4	37.6
Lesotho	LSO	Hypertensive disorder case management	18.4	13.5
Lesotho	LSO	Diabetes case management	14.4	10.5
Lesotho	LSO	Malaria case management	59.4	43.4
Lesotho	LSO	MgSO4 management of pre-eclampsia	36.4	26.6
Lesotho	LSO	Thermal protection	88.4	68.0
Lesotho	LSO	Clean cord care	85.3	65.6
Lesotho	LSO	Clean birth environment	73.3	56.4
Lesotho	LSO	Immediate drying and additional stimulation	81.9	63.0
Lesotho	LSO	Neonatal resuscitation	49.2	37.9
Lesotho	LSO	Antibiotics for preterm or prolonged PROM	66.9	51.5
Lesotho	LSO	Parenteral administration of anti-convulsants	63.9	49.2
Lesotho	LSO	Parenteral administration of uterotonics	79.9	61.5
Lesotho	LSO	Parenteral administration of antibiotics	66.9	51.5
Lesotho	LSO	Assisted vaginal delivery	22.6	17.4

Lesotho	LSO	Manual removal of placenta	33.4	25.7
Lesotho	LSO	Removal of retained products of conception	29.6	22.8
Lesotho	LSO	Cesarean delivery	7.8	6.0
Lesotho	LSO	Blood transfusion	11.3	8.7
Lesotho	LSO	Induction of labor for pregnancies lasting 41+ weeks	1.6	1.2
Lesotho	LSO	Complementary feeding - education only	23.1	17.8
Lesotho	LSO	Complementary feeding - supplementary feeding and education	23.1	17.8
Lesotho	LSO	Vitamin A supplementation	18.0	13.9
Lesotho	LSO	Improved sanitation - Utilization of latrines or toilets	42.8	42.8
Lesotho	LSO	Improved water source	68.6	68.6
Lesotho	LSO	Water connection in the home	43.5	43.5
Lesotho	LSO	Hand washing with soap	36.3	36.3
Lesotho	LSO	Hygienic disposal of children's stools	54.6	54.6
Lesotho	LSO	Injectable antibiotics for neonatal sepsis	89.4	65.4
Lesotho	LSO	ORS - oral rehydration solution	39.7	30.5
Lesotho	LSO	Antibiotics for treatment of dysentery	26.1	20.1
Lesotho	LSO	Zinc for treatment of diarrhea	17.4	13.4
Lesotho	LSO	Oral antibiotics for pneumonia	57.5	44.2
Lesotho	LSO	Vitamin A for treatment of measles	18.0	13.9
Lesotho	LSO	SAM - treatment for severe acute malnutrition	6.2	4.8
Lesotho	LSO	BCG vaccine	98.0	71.6
Lesotho	LSO	Polio vaccine	90.0	65.8
Lesotho	LSO	DPT vaccine	93.0	68.0
Lesotho	LSO	H. influenzae type b vaccine	93.0	85.0
Lesotho	LSO	HepB vaccine	93.0	68.0
Lesotho	LSO	Pneumococcal vaccine	93.0	85.0
Lesotho	LSO	Rotavirus vaccine	70.0	64.0
Lesotho	LSO	Measles vaccine	90.0	65.8
Lesotho	LSO	Global wasting (<-2 SD) rate	3.0	3.6
Lesotho	LSO	Contraceptive prevalence (CPR)	65.45	53.2
Liberia	LBR	TT - Tetanus toxoid vaccination	89.0	65.1
Liberia	LBR	IPTp - Intermittent preventive treatment of malaria during pregnancy	54.5	39.8
Liberia	LBR	Syphilis detection and treatment	23.7	17.3
Liberia	LBR	Iron supplementation in pregnancy	21.2	15.5
Liberia	LBR	Hypertensive disorder case management	18.6	13.6
Liberia	LBR	Diabetes case management	14.5	10.6
Liberia	LBR	Malaria case management	60.1	43.9
Liberia	LBR	MgSO4 management of pre-eclampsia	36.9	27.0
Liberia	LBR	Thermal protection	55.2	42.5
Liberia	LBR	Clean cord care	53.3	41.0
Liberia	LBR	Clean birth environment	45.8	35.2

Liberia	LBR	Immediate drying and additional stimulation	51.1	39.3
Liberia	LBR	Neonatal resuscitation	30.7	23.6
Liberia	LBR	Antibiotics for preterm or prolonged PROM	41.8	32.2
Liberia	LBR	Parenteral administration of anti-convulsants	39.9	30.7
Liberia	LBR	Parenteral administration of uterotonics	49.9	38.4
Liberia	LBR	Parenteral administration of antibiotics	41.8	32.2
Liberia	LBR	Assisted vaginal delivery	14.1	10.8
Liberia	LBR	Manual removal of placenta	20.9	16.1
Liberia	LBR	Removal of retained products of conception	18.5	14.2
Liberia	LBR	Cesarean delivery	4.9	3.8
Liberia	LBR	Blood transfusion	7.0	5.4
Liberia	LBR	Induction of labor for pregnancies lasting 41+ weeks	1.0	0.8
Liberia	LBR	Complementary feeding - education only	13.5	10.4
Liberia	LBR	Complementary feeding - supplementary feeding and education	13.5	10.4
Liberia	LBR	Vitamin A supplementation	97.0	74.8
Liberia	LBR	Improved sanitation - Utilization of latrines or toilets	17.0	17.0
Liberia	LBR	Improved water source	72.9	72.9
Liberia	LBR	Water connection in the home	3.4	3.4
Liberia	LBR	Hand washing with soap	3.8	3.8
Liberia	LBR	Hygienic disposal of children's stools	23.2	23.2
Liberia	LBR	ITN/IRS - Households protected from malaria	62.1	47.9
Liberia	LBR	Injectable antibiotics for neonatal sepsis	55.8	40.8
Liberia	LBR	ORS - oral rehydration solution	60.4	46.5
Liberia	LBR	Antibiotics for treatment of dysentery	18.7	14.4
Liberia	LBR	Zinc for treatment of diarrhea	3.1	2.4
Liberia	LBR	Oral antibiotics for pneumonia	50.7	39.0
Liberia	LBR	Vitamin A for treatment of measles	97.0	74.6
Liberia	LBR	ACTs- Artemisinin compounds for treatment of malaria	16.7	12.9
Liberia	LBR	SAM - treatment for severe acute malnutrition	15.1	11.6
Liberia	LBR	BCG vaccine	92.0	67.3
Liberia	LBR	Polio vaccine	84.0	61.4
Liberia	LBR	DPT vaccine	84.0	61.4
Liberia	LBR	H. influenzae type b vaccine	84.0	76.8
Liberia	LBR	HepB vaccine	84.0	61.4
Liberia	LBR	Pneumococcal vaccine	84.0	76.8
Liberia	LBR	Rotavirus vaccine	74.0	67.6
Liberia	LBR	Measles vaccine	87.0	63.6
Liberia	LBR	Global wasting (<-2 SD) rate	5.4	6.4
Liberia	LBR	Contraceptive prevalence (CPR)	30.45	24.7
Madagascar	MDG	Safe abortion services	3.3	2.7
Madagascar	MDG	TT - Tetanus toxoid vaccination	78.0	57.0

Madagascar	MDG	IPTp - Intermittent preventive treatment of malaria during pregnancy	22.3	16.3
Madagascar	MDG	Syphilis detection and treatment	12.6	9.2
Madagascar	MDG	Iron supplementation in pregnancy	7.6	5.6
Madagascar	MDG	Hypertensive disorder case management	11.3	8.3
Madagascar	MDG	Diabetes case management	8.8	6.4
Madagascar	MDG	Malaria case management	36.6	26.8
Madagascar	MDG	MgSO4 management of pre-eclampsia	22.4	16.4
Madagascar	MDG	Thermal protection	37.5	28.9
Madagascar	MDG	Clean cord care	36.2	27.9
Madagascar	MDG	Clean birth environment	31.1	23.9
Madagascar	MDG	Immediate drying and additional stimulation	34.7	26.7
Madagascar	MDG	Neonatal resuscitation	20.8	16.0
Madagascar	MDG	Antibiotics for preterm or prolonged PROM	28.4	21.9
Madagascar	MDG	Parenteral administration of anti-convulsants	27.1	20.9
Madagascar	MDG	Parenteral administration of uterotonics	33.9	26.1
Madagascar	MDG	Parenteral administration of antibiotics	28.4	21.9
Madagascar	MDG	Assisted vaginal delivery	9.6	7.4
Madagascar	MDG	Manual removal of placenta	14.2	10.9
Madagascar	MDG	Removal of retained products of conception	12.6	9.7
Madagascar	MDG	Cesarean delivery	3.3	2.5
Madagascar	MDG	Blood transfusion	4.8	3.7
Madagascar	MDG	Induction of labor for pregnancies lasting 41+ weeks	0.7	0.5
Madagascar	MDG	Complementary feeding - education only	24.1	18.6
Madagascar	MDG	Complementary feeding - supplementary feeding and education	24.1	18.6
Madagascar	MDG	Vitamin A supplementation	87.0	67.1
Madagascar	MDG	Improved sanitation - Utilization of latrines or toilets	10.5	10.5
Madagascar	MDG	Improved water source	54.4	54.4
Madagascar	MDG	Water connection in the home	19.3	19.3
Madagascar	MDG	Hand washing with soap	4.0	4.0
Madagascar	MDG	Hygienic disposal of children's stools	39.0	39.0
Madagascar	MDG	ITN/IRS - Households protected from malaria	79.5	61.3
Madagascar	MDG	Injectable antibiotics for neonatal sepsis	37.9	27.7
Madagascar	MDG	ORS - oral rehydration solution	44.5	34.2
Madagascar	MDG	Antibiotics for treatment of dysentery	34.1	26.2
Madagascar	MDG	Zinc for treatment of diarrhea	3.0	2.3
Madagascar	MDG	Oral antibiotics for pneumonia	42.0	32.3
Madagascar	MDG	Vitamin A for treatment of measles	87.0	66.9
Madagascar	MDG	ACTs- Artemisinin compounds for treatment of malaria	3.4	2.6
Madagascar	MDG	SAM - treatment for severe acute malnutrition	1.4	1.1
Madagascar	MDG	BCG vaccine	70.0	51.2

Madagascar	MDG	Polio vaccine	76.0	55.6
Madagascar	MDG	DPT vaccine	75.0	54.8
Madagascar	MDG	H. influenzae type b vaccine	75.0	68.5
Madagascar	MDG	HepB vaccine	75.0	54.8
Madagascar	MDG	Pneumococcal vaccine	75.0	68.5
Madagascar	MDG	Rotavirus vaccine	78.0	71.3
Madagascar	MDG	Measles vaccine	58.0	42.4
Madagascar	MDG	Global wasting (<-2 SD) rate	14.5	17.4
Madagascar	MDG	Contraceptive prevalence (CPR)	48.2	39.1
Malawi	MWI	Safe abortion services	3.3	2.7
Malawi	MWI	TT - Tetanus toxoid vaccination	89.0	65.1
Malawi	MWI	IPTp - Intermittent preventive treatment of malaria during pregnancy	76.1	55.6
Malawi	MWI	Syphilis detection and treatment	15.2	11.1
Malawi	MWI	Iron supplementation in pregnancy	33.4	24.4
Malawi	MWI	Hypertensive disorder case management	5.0	3.7
Malawi	MWI	Diabetes case management	6.4	4.7
Malawi	MWI	Malaria case management	41.0	30.0
Malawi	MWI	MgSO4 management of pre-eclampsia	40.6	29.7
Malawi	MWI	Thermal protection	90.5	69.6
Malawi	MWI	Clean cord care	84.1	64.7
Malawi	MWI	Clean birth environment	64.6	49.7
Malawi	MWI	Immediate drying and additional stimulation	83.0	63.9
Malawi	MWI	Neonatal resuscitation	83.7	64.4
Malawi	MWI	Antibiotics for preterm or prolonged PROM	80.3	61.8
Malawi	MWI	Parenteral administration of anti-convulsants	88.0	67.7
Malawi	MWI	Parenteral administration of uterotonics	89.5	68.9
Malawi	MWI	Parenteral administration of antibiotics	80.3	61.8
Malawi	MWI	Assisted vaginal delivery	46.1	35.5
Malawi	MWI	Manual removal of placenta	4.5	3.5
Malawi	MWI	Removal of retained products of conception	30.0	23.1
Malawi	MWI	Cesarean delivery	1.9	1.5
Malawi	MWI	Blood transfusion	12.2	9.4
Malawi	MWI	Induction of labor for pregnancies lasting 41+ weeks	6.1	4.7
Malawi	MWI	Complementary feeding - education only	25.0	19.3
Malawi	MWI	Complementary feeding - supplementary feeding and education	25.0	19.3
Malawi	MWI	Vitamin A supplementation	91.0	70.2
Malawi	MWI	Improved sanitation - Utilization of latrines or toilets	26.2	26.2
Malawi	MWI	Improved water source	68.8	68.8
Malawi	MWI	Water connection in the home	14.9	14.9
Malawi	MWI	Hand washing with soap	10.7	10.7
Malawi	MWI	Hygienic disposal of children's stools	85.5	85.5

Malawi	MWI	ITN/IRS - Households protected from malaria	82.1	63.4
Malawi	MWI	Injectable antibiotics for neonatal sepsis	90.7	66.3
Malawi	MWI	ORS - oral rehydration solution	64.7	49.8
Malawi	MWI	Antibiotics for treatment of dysentery	19.5	15.0
Malawi	MWI	Zinc for treatment of diarrhea	28.1	21.6
Malawi	MWI	Oral antibiotics for pneumonia	74.4	57.3
Malawi	MWI	Vitamin A for treatment of measles	91.0	70.0
Malawi	MWI	ACTs- Artemisinin compounds for treatment of malaria	27.8	21.4
Malawi	MWI	SAM - treatment for severe acute malnutrition	7.2	5.5
Malawi	MWI	BCG vaccine	92.0	67.3
Malawi	MWI	Polio vaccine	91.0	66.5
Malawi	MWI	DPT vaccine	92.0	67.3
Malawi	MWI	H. influenzae type b vaccine	92.0	84.1
Malawi	MWI	HepB vaccine	92.0	67.3
Malawi	MWI	Pneumococcal vaccine	92.0	84.1
Malawi	MWI	Rotavirus vaccine	90.0	82.3
Malawi	MWI	Measles vaccine	83.0	60.7
Malawi	MWI	Global wasting (<-2 SD) rate	2.8	3.4
Malawi	MWI	Contraceptive prevalence (CPR)	63	51.2
Maldives	MDV	Safe abortion services	35.1	28.5
Maldives	MDV	TT - Tetanus toxoid vaccination	99.0	72.4
Maldives	MDV	Syphilis detection and treatment	24.4	17.8
Maldives	MDV	Iron supplementation in pregnancy	64.6	47.2
Maldives	MDV	Hypertensive disorder case management	19.7	14.4
Maldives	MDV	Diabetes case management	15.3	11.2
Maldives	MDV	Malaria case management	63.5	46.4
Maldives	MDV	MgSO4 management of pre-eclampsia	38.9	28.4
Maldives	MDV	Thermal protection	98.3	75.6
Maldives	MDV	Clean cord care	94.9	73.0
Maldives	MDV	Clean birth environment	81.6	62.8
Maldives	MDV	Immediate drying and additional stimulation	91.1	70.1
Maldives	MDV	Neonatal resuscitation	54.7	42.1
Maldives	MDV	Antibiotics for preterm or prolonged PROM	74.4	57.3
Maldives	MDV	Parenteral administration of anti-convulsants	71.1	54.7
Maldives	MDV	Parenteral administration of uterotonics	88.9	68.4
Maldives	MDV	Parenteral administration of antibiotics	74.4	57.3
Maldives	MDV	Assisted vaginal delivery	25.1	19.3
Maldives	MDV	Manual removal of placenta	37.2	28.6
Maldives	MDV	Removal of retained products of conception	33.0	25.4
Maldives	MDV	Cesarean delivery	8.7	6.7
Maldives	MDV	Blood transfusion	12.5	9.6
Maldives	MDV	Induction of labor for pregnancies lasting 41+ weeks	1.8	1.4

Maldives	MDV	Complementary feeding - education only	75.8	58.5
Maldives	MDV	Complementary feeding - supplementary feeding and education	75.8	58.5
Maldives	MDV	Vitamin A supplementation	69.0	53.2
Maldives	MDV	Improved sanitation - Utilization of latrines or toilets	99.0	99.0
Maldives	MDV	Improved water source	99.0	99.0
Maldives	MDV	Water connection in the home	47.3	47.3
Maldives	MDV	Hand washing with soap	95.8	95.8
Maldives	MDV	Hygienic disposal of children's stools	9.1	9.1
Maldives	MDV	Injectable antibiotics for neonatal sepsis	99.5	72.7
Maldives	MDV	ORS - oral rehydration solution	74.8	57.6
Maldives	MDV	Zinc for treatment of diarrhea	48.3	37.2
Maldives	MDV	Oral antibiotics for pneumonia	74.1	57.0
Maldives	MDV	Vitamin A for treatment of measles	69.0	53.1
Maldives	MDV	BCG vaccine	99.0	72.4
Maldives	MDV	Polio vaccine	99.0	72.4
Maldives	MDV	DPT vaccine	99.0	72.4
Maldives	MDV	H. influenzae type b vaccine	99.0	90.5
Maldives	MDV	HepB vaccine	99.0	72.4
Maldives	MDV	Measles vaccine	99.0	72.4
Maldives	MDV	Global wasting (<-2 SD) rate	9.1	10.9
Maldives	MDV	Contraceptive prevalence (CPR)	34.55	28.1
Mali	MLI	TT - Tetanus toxoid vaccination	85.0	62.1
Mali	MLI	IPTp - Intermittent preventive treatment of malaria during pregnancy	55.4	40.5
Mali	MLI	Syphilis detection and treatment	19.4	14.2
Mali	MLI	Iron supplementation in pregnancy	18.3	13.4
Mali	MLI	Hypertensive disorder case management	10.4	7.6
Mali	MLI	Diabetes case management	8.1	5.9
Mali	MLI	Malaria case management	33.6	24.6
Mali	MLI	MgSO4 management of pre-eclampsia	20.6	15.1
Mali	MLI	Thermal protection	66.0	50.8
Mali	MLI	Clean cord care	63.7	49.0
Mali	MLI	Clean birth environment	54.8	42.2
Mali	MLI	Immediate drying and additional stimulation	61.2	47.1
Mali	MLI	Neonatal resuscitation	36.7	28.2
Mali	MLI	Antibiotics for preterm or prolonged PROM	50.0	38.5
Mali	MLI	Parenteral administration of anti-convulsants	47.8	36.8
Mali	MLI	Parenteral administration of uterotonics	59.7	45.9
Mali	MLI	Parenteral administration of antibiotics	50.0	38.5
Mali	MLI	Assisted vaginal delivery	16.9	13.0
Mali	MLI	Manual removal of placenta	25.0	19.2
Mali	MLI	Removal of retained products of conception	22.1	17.0

Mali	MLI	Cesarean delivery	5.8	4.5
Mali	MLI	Blood transfusion	8.4	6.5
Mali	MLI	Induction of labor for pregnancies lasting 41+ weeks	1.2	0.9
Mali	MLI	Complementary feeding - education only	21.8	16.8
Mali	MLI	Complementary feeding - supplementary feeding and education	21.8	16.8
Mali	MLI	Vitamin A supplementation	9.0	6.9
Mali	MLI	Improved sanitation - Utilization of latrines or toilets	39.3	39.3
Mali	MLI	Improved water source	78.3	78.3
Mali	MLI	Water connection in the home	35.6	35.6
Mali	MLI	Hand washing with soap	60.9	60.9
Mali	MLI	Hygienic disposal of children's stools	65.0	65.0
Mali	MLI	ITN/IRS - Households protected from malaria	89.8	69.3
Mali	MLI	Injectable antibiotics for neonatal sepsis	66.8	48.8
Mali	MLI	ORS - oral rehydration solution	21.4	16.5
Mali	MLI	Antibiotics for treatment of dysentery	18.5	14.2
Mali	MLI	Zinc for treatment of diarrhea	15.4	11.9
Mali	MLI	Oral antibiotics for pneumonia	70.9	54.6
Mali	MLI	Vitamin A for treatment of measles	9.0	6.9
Mali	MLI	ACTs- Artemisinin compounds for treatment of malaria	0.9	0.7
Mali	MLI	SAM - treatment for severe acute malnutrition	41.8	32.2
Mali	MLI	BCG vaccine	83.0	60.7
Mali	MLI	Polio vaccine	73.0	53.4
Mali	MLI	DPT vaccine	71.0	51.9
Mali	MLI	H. influenzae type b vaccine	71.0	64.9
Mali	MLI	HepB vaccine	71.0	51.9
Mali	MLI	Pneumococcal vaccine	68.0	62.1
Mali	MLI	Rotavirus vaccine	55.0	50.3
Mali	MLI	Meningococcal A	70.0	51.2
Mali	MLI	Measles vaccine	61.0	44.6
Mali	MLI	Global wasting (<-2 SD) rate	13.4	16.1
Mali	MLI	Contraceptive prevalence (CPR)	17.05	13.8
Mauritania	MRT	TT - Tetanus toxoid vaccination	80.0	58.5
Mauritania	MRT	IPTp - Intermittent preventive treatment of malaria during pregnancy	25.2	18.4
Mauritania	MRT	Syphilis detection and treatment	21.5	15.7
Mauritania	MRT	Iron supplementation in pregnancy	6.1	4.5
Mauritania	MRT	Hypertensive disorder case management	15.1	11.0
Mauritania	MRT	Diabetes case management	11.8	8.6
Mauritania	MRT	Malaria case management	48.8	35.7
Mauritania	MRT	MgSO4 management of pre-eclampsia	29.9	21.9
Mauritania	MRT	Thermal protection	68.5	52.7

Mauritania	MRT	Clean cord care	55.2	42.5
Mauritania	MRT	Clean birth environment	44.0	33.9
Mauritania	MRT	Immediate drying and additional stimulation	63.7	49.0
Mauritania	MRT	Neonatal resuscitation	32.4	24.9
Mauritania	MRT	Antibiotics for preterm or prolonged PROM	47.5	36.6
Mauritania	MRT	Parenteral administration of anti-convulsants	25.5	19.6
Mauritania	MRT	Parenteral administration of uterotonics	54.8	42.2
Mauritania	MRT	Parenteral administration of antibiotics	47.5	36.6
Mauritania	MRT	Assisted vaginal delivery	23.3	17.9
Mauritania	MRT	Manual removal of placenta	46.2	35.6
Mauritania	MRT	Removal of retained products of conception	28.1	21.6
Mauritania	MRT	Cesarean delivery	8.4	6.5
Mauritania	MRT	Blood transfusion	7.6	5.8
Mauritania	MRT	Induction of labor for pregnancies lasting 41+ weeks	6.7	5.2
Mauritania	MRT	Complementary feeding - education only	34.3	26.5
Mauritania	MRT	Complementary feeding - supplementary feeding and education	34.3	26.5
Mauritania	MRT	Improved sanitation - Utilization of latrines or toilets	48.4	48.4
Mauritania	MRT	Improved water source	70.7	70.7
Mauritania	MRT	Water connection in the home	37.0	37.0
Mauritania	MRT	Hand washing with soap	41.0	41.0
Mauritania	MRT	Hygienic disposal of children's stools	20.2	20.2
Mauritania	MRT	ITN/IRS - Households protected from malaria	48.9	37.7
Mauritania	MRT	Injectable antibiotics for neonatal sepsis	69.3	50.7
Mauritania	MRT	ORS - oral rehydration solution	25.4	19.5
Mauritania	MRT	Zinc for treatment of diarrhea	23.2	17.9
Mauritania	MRT	Oral antibiotics for pneumonia	33.7	25.9
Mauritania	MRT	ACTs- Artemisinin compounds for treatment of malaria	0.7	0.5
Mauritania	MRT	SAM - treatment for severe acute malnutrition	22.4	17.2
Mauritania	MRT	BCG vaccine	90.0	65.8
Mauritania	MRT	Polio vaccine	81.0	59.2
Mauritania	MRT	DPT vaccine	81.0	59.2
Mauritania	MRT	H. influenzae type b vaccine	81.0	74.0
Mauritania	MRT	HepB vaccine	81.0	59.2
Mauritania	MRT	Pneumococcal vaccine	77.0	70.4
Mauritania	MRT	Rotavirus vaccine	76.0	69.5
Mauritania	MRT	Measles vaccine	78.0	57.0
Mauritania	MRT	Global wasting (<-2 SD) rate	14.9	17.9
Mauritania	MRT	Contraceptive prevalence (CPR)	18.1	14.7
Mexico	MEX	Safe abortion services	0.6	0.5
Mexico	MEX	TT - Tetanus toxoid vaccination	96.0	70.2
Mexico	MEX	Syphilis detection and treatment	24.3	17.8

Mexico	MEX	Hypertensive disorder case management	22.6	16.5
Mexico	MEX	Diabetes case management	17.7	12.9
Mexico	MEX	Malaria case management	73.1	53.4
Mexico	MEX	MgSO4 management of pre-eclampsia	44.8	32.7
Mexico	MEX	Thermal protection	95.8	73.7
Mexico	MEX	Clean cord care	92.5	71.2
Mexico	MEX	Clean birth environment	79.5	61.2
Mexico	MEX	Immediate drying and additional stimulation	88.8	68.3
Mexico	MEX	Neonatal resuscitation	53.3	41.0
Mexico	MEX	Antibiotics for preterm or prolonged PROM	72.5	55.8
Mexico	MEX	Parenteral administration of anti-convulsants	69.3	53.3
Mexico	MEX	Parenteral administration of uterotonics	86.6	66.6
Mexico	MEX	Parenteral administration of antibiotics	72.5	55.8
Mexico	MEX	Assisted vaginal delivery	24.5	18.9
Mexico	MEX	Manual removal of placenta	36.3	27.9
Mexico	MEX	Removal of retained products of conception	32.1	24.7
Mexico	MEX	Cesarean delivery	8.4	6.5
Mexico	MEX	Blood transfusion	12.2	9.4
Mexico	MEX	Induction of labor for pregnancies lasting 41+ weeks	1.7	1.3
Mexico	MEX	Complementary feeding - education only	72.1	55.6
Mexico	MEX	Complementary feeding - supplementary feeding and education	72.1	55.6
Mexico	MEX	Improved sanitation - Utilization of latrines or toilets	91.2	91.2
Mexico	MEX	Improved water source	99.0	99.0
Mexico	MEX	Water connection in the home	95.0	95.0
Mexico	MEX	Hand washing with soap	90.3	90.3
Mexico	MEX	Hygienic disposal of children's stools	14.0	14.0
Mexico	MEX	Injectable antibiotics for neonatal sepsis	96.9	70.8
Mexico	MEX	ORS - oral rehydration solution	61.4	47.2
Mexico	MEX	Zinc for treatment of diarrhea	10.8	8.3
Mexico	MEX	Oral antibiotics for pneumonia	73.1	56.3
Mexico	MEX	BCG vaccine	96.0	70.2
Mexico	MEX	Polio vaccine	88.0	64.3
Mexico	MEX	DPT vaccine	88.0	64.3
Mexico	MEX	H. influenzae type b vaccine	88.0	80.4
Mexico	MEX	HepB vaccine	55.0	40.2
Mexico	MEX	Pneumococcal vaccine	88.0	80.4
Mexico	MEX	Rotavirus vaccine	77.0	70.4
Mexico	MEX	Measles vaccine	96.0	70.2
Mexico	MEX	Global wasting (<-2 SD) rate	1.1	1.3
Mexico	MEX	Contraceptive prevalence (CPR)	69.75	56.7
Mongolia	MNG	Safe abortion services	100.0	81.2

Mongolia	MNG	Syphilis detection and treatment	24.5	17.9
Mongolia	MNG	Iron supplementation in pregnancy	25.9	18.9
Mongolia	MNG	Hypertensive disorder case management	21.3	15.6
Mongolia	MNG	Diabetes case management	16.6	12.1
Mongolia	MNG	Malaria case management	68.6	50.1
Mongolia	MNG	MgSO4 management of pre-eclampsia	42.1	30.8
Mongolia	MNG	Thermal protection	97.3	74.9
Mongolia	MNG	Clean cord care	93.9	72.3
Mongolia	MNG	Clean birth environment	80.7	62.1
Mongolia	MNG	Immediate drying and additional stimulation	90.1	69.3
Mongolia	MNG	Neonatal resuscitation	54.1	41.6
Mongolia	MNG	Antibiotics for preterm or prolonged PROM	73.6	56.6
Mongolia	MNG	Parenteral administration of anti-convulsants	70.4	54.2
Mongolia	MNG	Parenteral administration of uterotonics	87.9	67.6
Mongolia	MNG	Parenteral administration of antibiotics	73.6	56.6
Mongolia	MNG	Assisted vaginal delivery	24.9	19.2
Mongolia	MNG	Manual removal of placenta	36.8	28.3
Mongolia	MNG	Removal of retained products of conception	32.6	25.1
Mongolia	MNG	Cesarean delivery	8.6	6.6
Mongolia	MNG	Blood transfusion	12.4	9.5
Mongolia	MNG	Induction of labor for pregnancies lasting 41+ weeks	1.7	1.3
Mongolia	MNG	Complementary feeding - education only	50.8	39.2
Mongolia	MNG	Complementary feeding - supplementary feeding and education	50.8	39.2
Mongolia	MNG	Vitamin A supplementation	83.0	64.0
Mongolia	MNG	Improved sanitation - Utilization of latrines or toilets	58.5	58.5
Mongolia	MNG	Improved water source	83.3	83.3
Mongolia	MNG	Water connection in the home	20.9	20.9
Mongolia	MNG	Hand washing with soap	78.9	78.9
Mongolia	MNG	Hygienic disposal of children's stools	51.3	51.3
Mongolia	MNG	Injectable antibiotics for neonatal sepsis	98.4	71.9
Mongolia	MNG	ORS - oral rehydration solution	58.2	44.8
Mongolia	MNG	Antibiotics for treatment of dysentery	20.1	15.5
Mongolia	MNG	Zinc for treatment of diarrhea	16.5	12.7
Mongolia	MNG	Oral antibiotics for pneumonia	70.3	54.1
Mongolia	MNG	Vitamin A for treatment of measles	83.0	63.9
Mongolia	MNG	BCG vaccine	99.0	72.4
Mongolia	MNG	Polio vaccine	99.0	72.4
Mongolia	MNG	DPT vaccine	99.0	72.4
Mongolia	MNG	H. influenzae type b vaccine	99.0	90.5
Mongolia	MNG	HepB vaccine	99.0	72.4
Mongolia	MNG	Pneumococcal vaccine	26.0	23.8

Mongolia	MNG	Measles vaccine	99.0	72.4
Mongolia	MNG	Global wasting (<-2 SD) rate	1.0	1.2
Mongolia	MNG	Contraceptive prevalence (CPR)	55.4	45.0
Montenegro	MNE	Safe abortion services	100.0	81.2
Montenegro	MNE	Syphilis detection and treatment	24.0	17.5
Montenegro	MNE	Hypertensive disorder case management	22.6	16.5
Montenegro	MNE	Diabetes case management	17.6	12.9
Montenegro	MNE	Malaria case management	73.0	53.4
Montenegro	MNE	MgSO4 management of pre-eclampsia	44.8	32.7
Montenegro	MNE	Thermal protection	97.5	75.0
Montenegro	MNE	Clean cord care	94.1	72.4
Montenegro	MNE	Clean birth environment	80.9	62.3
Montenegro	MNE	Immediate drying and additional stimulation	90.3	69.5
Montenegro	MNE	Neonatal resuscitation	54.2	41.7
Montenegro	MNE	Antibiotics for preterm or prolonged PROM	73.8	56.8
Montenegro	MNE	Parenteral administration of anti-convulsants	70.5	54.2
Montenegro	MNE	Parenteral administration of uterotonics	88.1	67.8
Montenegro	MNE	Parenteral administration of antibiotics	73.8	56.8
Montenegro	MNE	Assisted vaginal delivery	24.9	19.2
Montenegro	MNE	Manual removal of placenta	36.9	28.4
Montenegro	MNE	Removal of retained products of conception	32.7	25.2
Montenegro	MNE	Cesarean delivery	8.6	6.6
Montenegro	MNE	Blood transfusion	12.4	9.5
Montenegro	MNE	Induction of labor for pregnancies lasting 41+ weeks	1.7	1.3
Montenegro	MNE	Complementary feeding - education only	81.3	62.7
Montenegro	MNE	Complementary feeding - supplementary feeding and education	81.3	62.7
Montenegro	MNE	Improved sanitation - Utilization of latrines or toilets	97.8	97.8
Montenegro	MNE	Improved water source	97.0	97.0
Montenegro	MNE	Water connection in the home	82.4	82.4
Montenegro	MNE	Hand washing with soap	99.6	99.6
Montenegro	MNE	Hygienic disposal of children's stools	21.3	21.3
Montenegro	MNE	Injectable antibiotics for neonatal sepsis	98.6	72.1
Montenegro	MNE	ORS - oral rehydration solution	31.9	24.5
Montenegro	MNE	Oral antibiotics for pneumonia	89.4	68.8
Montenegro	MNE	BCG vaccine	83.0	60.7
Montenegro	MNE	Polio vaccine	87.0	63.6
Montenegro	MNE	DPT vaccine	87.0	63.6
Montenegro	MNE	H. influenzae type b vaccine	87.0	79.5
Montenegro	MNE	HepB vaccine	73.0	53.4
Montenegro	MNE	Measles vaccine	58.0	42.4
Montenegro	MNE	Global wasting (<-2 SD) rate	2.9	3.5

Montenegro	MNE	Contraceptive prevalence (CPR)	27.3	22.2
Morocco	MAR	Safe abortion services	2.2	1.8
Morocco	MAR	TT - Tetanus toxoid vaccination	88.0	64.3
Morocco	MAR	Syphilis detection and treatment	16.7	12.2
Morocco	MAR	Iron supplementation in pregnancy	5.2	3.8
Morocco	MAR	Hypertensive disorder case management	7.2	5.3
Morocco	MAR	Diabetes case management	5.6	4.1
Morocco	MAR	Malaria case management	23.3	17.0
Morocco	MAR	MgSO4 management of pre-eclampsia	14.3	10.5
Morocco	MAR	Thermal protection	71.9	55.3
Morocco	MAR	Clean cord care	69.4	53.4
Morocco	MAR	Clean birth environment	59.6	45.9
Morocco	MAR	Immediate drying and additional stimulation	66.6	51.2
Morocco	MAR	Neonatal resuscitation	40.0	30.8
Morocco	MAR	Antibiotics for preterm or prolonged PROM	54.4	41.9
Morocco	MAR	Parenteral administration of anti-convulsants	52.0	40.0
Morocco	MAR	Parenteral administration of uterotonics	65.0	50.0
Morocco	MAR	Parenteral administration of antibiotics	54.4	41.9
Morocco	MAR	Assisted vaginal delivery	18.4	14.2
Morocco	MAR	Manual removal of placenta	27.2	20.9
Morocco	MAR	Removal of retained products of conception	24.1	18.5
Morocco	MAR	Cesarean delivery	6.3	4.8
Morocco	MAR	Blood transfusion	9.2	7.1
Morocco	MAR	Induction of labor for pregnancies lasting 41+ weeks	1.3	1.0
Morocco	MAR	Complementary feeding - education only	22.3	17.2
Morocco	MAR	Complementary feeding - supplementary feeding and education	22.3	17.2
Morocco	MAR	Vitamin A supplementation	99.0	76.4
Morocco	MAR	Improved sanitation - Utilization of latrines or toilets	88.5	88.5
Morocco	MAR	Improved water source	86.8	86.8
Morocco	MAR	Water connection in the home	67.7	67.7
Morocco	MAR	Hygienic disposal of children's stools	6.3	6.3
Morocco	MAR	Injectable antibiotics for neonatal sepsis	72.7	53.1
Morocco	MAR	ORS - oral rehydration solution	22.9	17.6
Morocco	MAR	Vitamin A for treatment of measles	99.0	76.2
Morocco	MAR	BCG vaccine	99.0	72.4
Morocco	MAR	Polio vaccine	99.0	72.4
Morocco	MAR	DPT vaccine	99.0	72.4
Morocco	MAR	H. influenzae type b vaccine	99.0	90.5
Morocco	MAR	HepB vaccine	99.0	72.4
Morocco	MAR	Pneumococcal vaccine	99.0	90.5
Morocco	MAR	Rotavirus vaccine	99.0	90.5

Morocco	MAR	Measles vaccine	99.0	72.4
Morocco	MAR	Global wasting (<-2 SD) rate	10.1	12.1
Morocco	MAR	Contraceptive prevalence (CPR)	69.85	56.7
Mozambique	MOZ	Safe abortion services	3.3	2.7
Mozambique	MOZ	TT - Tetanus toxoid vaccination	86.0	62.9
Mozambique	MOZ	IPTp - Intermittent preventive treatment of malaria during pregnancy	35.8	26.2
Mozambique	MOZ	Syphilis detection and treatment	22.4	16.4
Mozambique	MOZ	Iron supplementation in pregnancy	25.9	18.9
Mozambique	MOZ	Hypertensive disorder case management	12.9	9.4
Mozambique	MOZ	Diabetes case management	10.1	7.4
Mozambique	MOZ	Malaria case management	41.7	30.5
Mozambique	MOZ	MgSO4 management of pre-eclampsia	25.6	18.7
Mozambique	MOZ	Thermal protection	64.1	49.3
Mozambique	MOZ	Clean cord care	61.9	47.6
Mozambique	MOZ	Clean birth environment	53.2	40.9
Mozambique	MOZ	Immediate drying and additional stimulation	59.4	45.7
Mozambique	MOZ	Neonatal resuscitation	35.7	27.5
Mozambique	MOZ	Antibiotics for preterm or prolonged PROM	48.5	37.3
Mozambique	MOZ	Parenteral administration of anti-convulsants	46.4	35.7
Mozambique	MOZ	Parenteral administration of uterotonics	57.9	44.6
Mozambique	MOZ	Parenteral administration of antibiotics	48.5	37.3
Mozambique	MOZ	Assisted vaginal delivery	16.4	12.6
Mozambique	MOZ	Manual removal of placenta	24.3	18.7
Mozambique	MOZ	Removal of retained products of conception	21.5	16.5
Mozambique	MOZ	Cesarean delivery	5.6	4.3
Mozambique	MOZ	Blood transfusion	8.2	6.3
Mozambique	MOZ	Induction of labor for pregnancies lasting 41+ weeks	1.1	0.8
Mozambique	MOZ	Complementary feeding - education only	30.4	23.5
Mozambique	MOZ	Complementary feeding - supplementary feeding and education	30.4	23.5
Mozambique	MOZ	Vitamin A supplementation	61.0	47.1
Mozambique	MOZ	Improved sanitation - Utilization of latrines or toilets	29.4	29.4
Mozambique	MOZ	Improved water source	55.7	55.7
Mozambique	MOZ	Water connection in the home	19.8	19.8
Mozambique	MOZ	Hand washing with soap	31.7	31.7
Mozambique	MOZ	Hygienic disposal of children's stools	45.0	45.0
Mozambique	MOZ	ITN/IRS - Households protected from malaria	68.7	53.0
Mozambique	MOZ	Injectable antibiotics for neonatal sepsis	64.8	47.4
Mozambique	MOZ	ORS - oral rehydration solution	45.9	35.3
Mozambique	MOZ	Antibiotics for treatment of dysentery	31.6	24.3
Mozambique	MOZ	Zinc for treatment of diarrhea	30.9	23.8
Mozambique	MOZ	Oral antibiotics for pneumonia	59.4	45.7

Mozambique	MOZ	Vitamin A for treatment of measles	61.0	46.9
Mozambique	MOZ	ACTs- Artemisinin compounds for treatment of malaria	15.4	11.9
Mozambique	MOZ	BCG vaccine	95.0	69.4
Mozambique	MOZ	Polio vaccine	80.0	58.5
Mozambique	MOZ	DPT vaccine	80.0	58.5
Mozambique	MOZ	H. influenzae type b vaccine	80.0	73.1
Mozambique	MOZ	HepB vaccine	80.0	58.5
Mozambique	MOZ	Pneumococcal vaccine	80.0	73.1
Mozambique	MOZ	Rotavirus vaccine	80.0	73.1
Mozambique	MOZ	Measles vaccine	85.0	62.1
Mozambique	MOZ	Global wasting (<-2 SD) rate	6.0	7.2
Mozambique	MOZ	Contraceptive prevalence (CPR)	29.95	24.3
Myanmar	MMR	Safe abortion services	39.0	31.7
Myanmar	MMR	TT - Tetanus toxoid vaccination	90.0	65.8
Myanmar	MMR	Syphilis detection and treatment	20.5	15.0
Myanmar	MMR	Iron supplementation in pregnancy	59.3	43.3
Myanmar	MMR	Hypertensive disorder case management	14.3	10.5
Myanmar	MMR	Diabetes case management	11.2	8.2
Myanmar	MMR	Malaria case management	46.1	33.7
Myanmar	MMR	MgSO4 management of pre-eclampsia	28.3	20.7
Myanmar	MMR	Thermal protection	36.6	28.2
Myanmar	MMR	Clean cord care	35.4	27.2
Myanmar	MMR	Clean birth environment	30.4	23.4
Myanmar	MMR	Immediate drying and additional stimulation	33.9	26.1
Myanmar	MMR	Neonatal resuscitation	20.4	15.7
Myanmar	MMR	Antibiotics for preterm or prolonged PROM	27.7	21.3
Myanmar	MMR	Parenteral administration of anti-convulsants	26.5	20.4
Myanmar	MMR	Parenteral administration of uterotonics	33.1	25.5
Myanmar	MMR	Parenteral administration of antibiotics	27.7	21.3
Myanmar	MMR	Assisted vaginal delivery	9.4	7.2
Myanmar	MMR	Manual removal of placenta	13.9	10.7
Myanmar	MMR	Removal of retained products of conception	12.3	9.5
Myanmar	MMR	Cesarean delivery	3.2	2.5
Myanmar	MMR	Blood transfusion	4.7	3.6
Myanmar	MMR	Induction of labor for pregnancies lasting 41+ weeks	0.7	0.5
Myanmar	MMR	Complementary feeding - education only	24.8	19.1
Myanmar	MMR	Complementary feeding - supplementary feeding and education	24.8	19.1
Myanmar	MMR	Vitamin A supplementation	89.0	68.7
Myanmar	MMR	Improved sanitation - Utilization of latrines or toilets	64.3	64.3
Myanmar	MMR	Improved water source	81.8	81.8
Myanmar	MMR	Water connection in the home	20.1	20.1

Myanmar	MMR	Hand washing with soap	79.9	79.9
Myanmar	MMR	Hygienic disposal of children's stools	42.1	42.1
Myanmar	MMR	ITN/IRS - Households protected from malaria	26.8	20.7
Myanmar	MMR	Injectable antibiotics for neonatal sepsis	37.1	27.1
Myanmar	MMR	ORS - oral rehydration solution	61.9	47.6
Myanmar	MMR	Zinc for treatment of diarrhea	8.4	6.5
Myanmar	MMR	Oral antibiotics for pneumonia	58.6	45.1
Myanmar	MMR	Vitamin A for treatment of measles	89.0	68.5
Myanmar	MMR	ACTs- Artemisinin compounds for treatment of malaria	0.2	0.2
Myanmar	MMR	SAM - treatment for severe acute malnutrition	3.5	2.7
Myanmar	MMR	BCG vaccine	90.0	65.8
Myanmar	MMR	Polio vaccine	91.0	66.5
Myanmar	MMR	DPT vaccine	91.0	66.5
Myanmar	MMR	H. influenzae type b vaccine	91.0	83.2
Myanmar	MMR	HepB vaccine	91.0	66.5
Myanmar	MMR	Pneumococcal vaccine	91.0	83.2
Myanmar	MMR	Measles vaccine	83.0	60.7
Myanmar	MMR	Global wasting (<-2 SD) rate	6.8	8.1
Myanmar	MMR	Contraceptive prevalence (CPR)	56.65	46.0
Namibia	NAM	Safe abortion services	42.5	34.5
Namibia	NAM	TT - Tetanus toxoid vaccination	88.0	64.3
Namibia	NAM	IPTp - Intermittent preventive treatment of malaria during pregnancy	5.1	3.7
Namibia	NAM	Syphilis detection and treatment	92.6	67.7
Namibia	NAM	Iron supplementation in pregnancy	38.6	28.2
Namibia	NAM	Hypertensive disorder case management	42.1	30.8
Namibia	NAM	Diabetes case management	32.2	23.5
Namibia	NAM	Malaria case management	45.5	33.3
Namibia	NAM	MgSO4 management of pre-eclampsia	31.8	23.2
Namibia	NAM	Thermal protection	86.7	66.7
Namibia	NAM	Clean cord care	87.2	67.1
Namibia	NAM	Clean birth environment	69.1	53.2
Namibia	NAM	Immediate drying and additional stimulation	86.5	66.6
Namibia	NAM	Neonatal resuscitation	78.2	60.2
Namibia	NAM	Antibiotics for preterm or prolonged PROM	81.9	63.0
Namibia	NAM	Parenteral administration of anti-convulsants	86.0	66.2
Namibia	NAM	Parenteral administration of uterotonics	76.9	59.2
Namibia	NAM	Parenteral administration of antibiotics	81.9	63.0
Namibia	NAM	Assisted vaginal delivery	39.4	30.3
Namibia	NAM	Manual removal of placenta	65.3	50.2
Namibia	NAM	Removal of retained products of conception	23.0	17.7
Namibia	NAM	Cesarean delivery	41.8	32.2
Namibia	NAM	Blood transfusion	18.9	14.5

Namibia	NAM	Induction of labor for pregnancies lasting 41+ weeks	1.5	1.2
Namibia	NAM	Complementary feeding - education only	31.1	24.0
Namibia	NAM	Complementary feeding - supplementary feeding and education	31.1	24.0
Namibia	NAM	Vitamin A supplementation	27.0	20.8
Namibia	NAM	Improved sanitation - Utilization of latrines or toilets	34.5	34.5
Namibia	NAM	Improved water source	82.5	82.5
Namibia	NAM	Water connection in the home	65.4	65.4
Namibia	NAM	Hand washing with soap	54.1	54.1
Namibia	NAM	Hygienic disposal of children's stools	12.6	12.6
Namibia	NAM	ITN/IRS - Households protected from malaria	32.7	25.2
Namibia	NAM	Injectable antibiotics for neonatal sepsis	87.7	64.1
Namibia	NAM	ORS - oral rehydration solution	71.6	55.1
Namibia	NAM	Antibiotics for treatment of dysentery	13.4	10.3
Namibia	NAM	Zinc for treatment of diarrhea	0.2	0.2
Namibia	NAM	Oral antibiotics for pneumonia	67.7	52.1
Namibia	NAM	Vitamin A for treatment of measles	27.0	20.8
Namibia	NAM	ACTs- Artemisinin compounds for treatment of malaria	3.2	2.5
Namibia	NAM	BCG vaccine	94.0	68.7
Namibia	NAM	Polio vaccine	84.0	61.4
Namibia	NAM	DPT vaccine	89.0	65.1
Namibia	NAM	H. influenzae type b vaccine	89.0	81.3
Namibia	NAM	HepB vaccine	89.0	65.1
Namibia	NAM	Pneumococcal vaccine	61.0	55.7
Namibia	NAM	Rotavirus vaccine	92.0	84.1
Namibia	NAM	Measles vaccine	80.0	58.5
Namibia	NAM	Global wasting (<-2 SD) rate	7.1	8.6
Namibia	NAM	Contraceptive prevalence (CPR)	60.3	49.0
Nepal	NPL	Safe abortion services	35.1	28.5
Nepal	NPL	TT - Tetanus toxoid vaccination	89.0	65.1
Nepal	NPL	Syphilis detection and treatment	8.2	6.0
Nepal	NPL	Iron supplementation in pregnancy	70.9	51.8
Nepal	NPL	Hypertensive disorder case management	15.2	11.1
Nepal	NPL	Diabetes case management	8.5	6.2
Nepal	NPL	Malaria case management	21.8	15.9
Nepal	NPL	MgSO4 management of pre-eclampsia	41.6	30.4
Nepal	NPL	Thermal protection	56.7	43.6
Nepal	NPL	Clean cord care	56.6	43.6
Nepal	NPL	Clean birth environment	54.1	41.6
Nepal	NPL	Immediate drying and additional stimulation	55.5	42.7
Nepal	NPL	Neonatal resuscitation	53.3	41.0
Nepal	NPL	Antibiotics for preterm or prolonged PROM	44.3	34.1

Nepal	NPL	Parenteral administration of anti-convulsants	52.8	40.6
Nepal	NPL	Parenteral administration of uterotonics	55.8	42.9
Nepal	NPL	Parenteral administration of antibiotics	44.3	34.1
Nepal	NPL	Assisted vaginal delivery	39.9	30.7
Nepal	NPL	Manual removal of placenta	15.3	11.8
Nepal	NPL	Removal of retained products of conception	37.5	28.9
Nepal	NPL	Cesarean delivery	7.8	6.0
Nepal	NPL	Blood transfusion	13.8	10.6
Nepal	NPL	Induction of labor for pregnancies lasting 41+ weeks	18.9	14.5
Nepal	NPL	Complementary feeding - education only	46.5	35.9
Nepal	NPL	Complementary feeding - supplementary feeding and education	46.5	35.9
Nepal	NPL	Vitamin A supplementation	81.0	62.5
Nepal	NPL	Improved sanitation - Utilization of latrines or toilets	62.1	62.1
Nepal	NPL	Improved water source	88.8	88.8
Nepal	NPL	Water connection in the home	42.1	42.1
Nepal	NPL	Hand washing with soap	72.5	72.5
Nepal	NPL	Hygienic disposal of children's stools	44.4	44.4
Nepal	NPL	Injectable antibiotics for neonatal sepsis	57.4	42.0
Nepal	NPL	ORS - oral rehydration solution	37.0	28.5
Nepal	NPL	Antibiotics for treatment of dysentery	21.3	16.4
Nepal	NPL	Zinc for treatment of diarrhea	17.6	13.5
Nepal	NPL	Oral antibiotics for pneumonia	55.1	42.4
Nepal	NPL	Vitamin A for treatment of measles	81.0	62.3
Nepal	NPL	ACTs- Artemisinin compounds for treatment of malaria	0.2	0.2
Nepal	NPL	SAM - treatment for severe acute malnutrition	0.9	0.7
Nepal	NPL	BCG vaccine	96.0	70.2
Nepal	NPL	Polio vaccine	91.0	66.5
Nepal	NPL	DPT vaccine	91.0	66.5
Nepal	NPL	H. influenzae type b vaccine	91.0	83.2
Nepal	NPL	HepB vaccine	91.0	66.5
Nepal	NPL	Pneumococcal vaccine	82.0	74.9
Nepal	NPL	Measles vaccine	90.0	65.8
Nepal	NPL	Global wasting (<-2 SD) rate	9.8	11.7
Nepal	NPL	Contraceptive prevalence (CPR)	55.1	44.8
Nicaragua	NIC	Safe abortion services	0.6	0.5
Nicaragua	NIC	TT - Tetanus toxoid vaccination	90.0	65.8
Nicaragua	NIC	Syphilis detection and treatment	21.7	15.9
Nicaragua	NIC	Iron supplementation in pregnancy	61.6	45.0
Nicaragua	NIC	Hypertensive disorder case management	21.1	15.4
Nicaragua	NIC	Diabetes case management	16.4	12.0
Nicaragua	NIC	Malaria case management	68.1	49.8

Nicaragua	NIC	MgSO4 management of pre-eclampsia	41.7	30.5
Nicaragua	NIC	Thermal protection	74.4	57.3
Nicaragua	NIC	Clean cord care	71.8	55.3
Nicaragua	NIC	Clean birth environment	61.7	47.5
Nicaragua	NIC	Immediate drying and additional stimulation	68.9	53.0
Nicaragua	NIC	Neonatal resuscitation	41.4	31.9
Nicaragua	NIC	Antibiotics for preterm or prolonged PROM	56.3	43.3
Nicaragua	NIC	Parenteral administration of anti-convulsants	53.8	41.4
Nicaragua	NIC	Parenteral administration of uterotonics	67.2	51.7
Nicaragua	NIC	Parenteral administration of antibiotics	56.3	43.3
Nicaragua	NIC	Assisted vaginal delivery	19.0	14.6
Nicaragua	NIC	Manual removal of placenta	28.1	21.6
Nicaragua	NIC	Removal of retained products of conception	24.9	19.2
Nicaragua	NIC	Cesarean delivery	6.5	5.0
Nicaragua	NIC	Blood transfusion	9.5	7.3
Nicaragua	NIC	Induction of labor for pregnancies lasting 41+ weeks	1.3	1.0
Nicaragua	NIC	Vitamin A supplementation	3.0	2.3
Nicaragua	NIC	Improved sanitation - Utilization of latrines or toilets	74.4	74.4
Nicaragua	NIC	Improved water source	81.5	81.5
Nicaragua	NIC	Water connection in the home	56.5	56.5
Nicaragua	NIC	Hygienic disposal of children's stools	43.2	43.2
Nicaragua	NIC	Injectable antibiotics for neonatal sepsis	75.2	55.0
Nicaragua	NIC	ORS - oral rehydration solution	65.4	50.3
Nicaragua	NIC	Antibiotics for treatment of dysentery	10.6	8.2
Nicaragua	NIC	Vitamin A for treatment of measles	3.0	2.3
Nicaragua	NIC	BCG vaccine	98.0	71.6
Nicaragua	NIC	Polio vaccine	99.0	72.4
Nicaragua	NIC	DPT vaccine	98.0	71.6
Nicaragua	NIC	H. influenzae type b vaccine	98.0	89.6
Nicaragua	NIC	HepB vaccine	98.0	71.6
Nicaragua	NIC	Pneumococcal vaccine	98.0	89.6
Nicaragua	NIC	Rotavirus vaccine	98.0	89.6
Nicaragua	NIC	Measles vaccine	99.0	72.4
Nicaragua	NIC	Global wasting (<-2 SD) rate	1.4	1.7
Nicaragua	NIC	Contraceptive prevalence (CPR)	81.7	66.4
Niger	NER	TT - Tetanus toxoid vaccination	81.0	59.2
Niger	NER	IPTp - Intermittent preventive treatment of malaria during pregnancy	36.9	27.0
Niger	NER	Syphilis detection and treatment	20.8	15.2
Niger	NER	Iron supplementation in pregnancy	28.6	20.9
Niger	NER	Hypertensive disorder case management	8.0	5.8
Niger	NER	Diabetes case management	6.2	4.5

Niger	NER	Malaria case management	25.8	18.9
Niger	NER	MgSO4 management of pre-eclampsia	15.8	11.6
Niger	NER	Thermal protection	29.4	22.6
Niger	NER	Clean cord care	28.4	21.9
Niger	NER	Clean birth environment	24.4	18.8
Niger	NER	Immediate drying and additional stimulation	27.2	20.9
Niger	NER	Neonatal resuscitation	16.4	12.6
Niger	NER	Antibiotics for preterm or prolonged PROM	22.3	17.2
Niger	NER	Parenteral administration of anti-convulsants	21.3	16.4
Niger	NER	Parenteral administration of uterotonics	26.6	20.5
Niger	NER	Parenteral administration of antibiotics	22.3	17.2
Niger	NER	Assisted vaginal delivery	7.5	5.8
Niger	NER	Manual removal of placenta	11.1	8.5
Niger	NER	Removal of retained products of conception	9.9	7.6
Niger	NER	Cesarean delivery	2.6	2.0
Niger	NER	Blood transfusion	3.7	2.8
Niger	NER	Induction of labor for pregnancies lasting 41+ weeks	0.5	0.4
Niger	NER	Complementary feeding - education only	9.9	7.6
Niger	NER	Complementary feeding - supplementary feeding and education	9.9	7.6
Niger	NER	Vitamin A supplementation	53.0	40.9
Niger	NER	Improved sanitation - Utilization of latrines or toilets	13.6	13.6
Niger	NER	Improved water source	50.3	50.3
Niger	NER	Water connection in the home	16.3	16.3
Niger	NER	Hygienic disposal of children's stools	19.4	19.4
Niger	NER	ITN/IRS - Households protected from malaria	61.5	47.5
Niger	NER	Injectable antibiotics for neonatal sepsis	29.8	21.8
Niger	NER	ORS - oral rehydration solution	44.3	34.1
Niger	NER	Antibiotics for treatment of dysentery	16.8	12.9
Niger	NER	Zinc for treatment of diarrhea	10.3	7.9
Niger	NER	Oral antibiotics for pneumonia	53.2	40.9
Niger	NER	Vitamin A for treatment of measles	53.0	40.8
Niger	NER	ACTs- Artemisinin compounds for treatment of malaria	11.6	8.9
Niger	NER	SAM - treatment for severe acute malnutrition	40.0	30.8
Niger	NER	BCG vaccine	87.0	63.6
Niger	NER	Polio vaccine	79.0	57.8
Niger	NER	DPT vaccine	79.0	57.8
Niger	NER	H. influenzae type b vaccine	79.0	72.2
Niger	NER	HepB vaccine	79.0	57.8
Niger	NER	Pneumococcal vaccine	79.0	72.2
Niger	NER	Rotavirus vaccine	79.0	72.2
Niger	NER	Meningococcal A	80.0	58.5

Niger	NER	Measles vaccine	78.0	57.0
Niger	NER	Global wasting (<-2 SD) rate	18.5	22.2
Niger	NER	Contraceptive prevalence (CPR)	17.75	14.4
Nigeria	NGA	TT - Tetanus toxoid vaccination	55.0	40.2
Nigeria	NGA	IPTp - Intermittent preventive treatment of malaria during pregnancy	40.4	29.5
Nigeria	NGA	Syphilis detection and treatment	18.3	13.4
Nigeria	NGA	Iron supplementation in pregnancy	20.5	15.0
Nigeria	NGA	Hypertensive disorder case management	13.6	9.9
Nigeria	NGA	Diabetes case management	10.6	7.7
Nigeria	NGA	Malaria case management	44.0	32.2
Nigeria	NGA	MgSO4 management of pre-eclampsia	27.0	19.7
Nigeria	NGA	Thermal protection	38.9	29.9
Nigeria	NGA	Clean cord care	37.6	28.9
Nigeria	NGA	Clean birth environment	32.3	24.9
Nigeria	NGA	Immediate drying and additional stimulation	36.1	27.8
Nigeria	NGA	Neonatal resuscitation	21.7	16.7
Nigeria	NGA	Antibiotics for preterm or prolonged PROM	29.5	22.7
Nigeria	NGA	Parenteral administration of anti-convulsants	28.2	21.7
Nigeria	NGA	Parenteral administration of uterotonics	35.2	27.1
Nigeria	NGA	Parenteral administration of antibiotics	29.5	22.7
Nigeria	NGA	Assisted vaginal delivery	10.0	7.7
Nigeria	NGA	Manual removal of placenta	14.7	11.3
Nigeria	NGA	Removal of retained products of conception	13.1	10.1
Nigeria	NGA	Cesarean delivery	3.4	2.6
Nigeria	NGA	Blood transfusion	5.0	3.8
Nigeria	NGA	Induction of labor for pregnancies lasting 41+ weeks	0.7	0.5
Nigeria	NGA	Complementary feeding - education only	22.6	17.4
Nigeria	NGA	Complementary feeding - supplementary feeding and education	22.6	17.4
Nigeria	NGA	Vitamin A supplementation	83.0	64.0
Nigeria	NGA	Improved sanitation - Utilization of latrines or toilets	39.2	39.2
Nigeria	NGA	Improved water source	71.4	71.4
Nigeria	NGA	Water connection in the home	8.0	8.0
Nigeria	NGA	Hand washing with soap	29.3	29.3
Nigeria	NGA	Hygienic disposal of children's stools	56.5	56.5
Nigeria	NGA	ITN/IRS - Households protected from malaria	60.6	46.8
Nigeria	NGA	Injectable antibiotics for neonatal sepsis	39.4	28.8
Nigeria	NGA	ORS - oral rehydration solution	40.0	30.8
Nigeria	NGA	Antibiotics for treatment of dysentery	40.8	31.4
Nigeria	NGA	Zinc for treatment of diarrhea	31.1	23.9
Nigeria	NGA	Oral antibiotics for pneumonia	74.5	57.3
Nigeria	NGA	Vitamin A for treatment of measles	83.0	63.9

Nigeria	NGA	ACTs- Artemisinin compounds for treatment of malaria	5.6	4.3
Nigeria	NGA	SAM - treatment for severe acute malnutrition	5.8	4.5
Nigeria	NGA	BCG vaccine	53.0	38.7
Nigeria	NGA	Polio vaccine	57.0	41.7
Nigeria	NGA	DPT vaccine	57.0	41.7
Nigeria	NGA	H. influenzae type b vaccine	57.0	52.1
Nigeria	NGA	HepB vaccine	57.0	41.7
Nigeria	NGA	Pneumococcal vaccine	57.0	52.1
Nigeria	NGA	Measles vaccine	42.0	30.7
Nigeria	NGA	Global wasting (<-2 SD) rate	10.9	13.1
Nigeria	NGA	Contraceptive prevalence (CPR)	22.3	18.1
Pakistan	PAK	Safe abortion services	35.1	28.5
Pakistan	PAK	TT - Tetanus toxoid vaccination	85.0	62.1
Pakistan	PAK	Syphilis detection and treatment	21.6	15.8
Pakistan	PAK	Iron supplementation in pregnancy	29.4	21.5
Pakistan	PAK	Hypertensive disorder case management	12.6	9.2
Pakistan	PAK	Diabetes case management	9.8	7.2
Pakistan	PAK	Malaria case management	40.5	29.6
Pakistan	PAK	MgSO4 management of pre-eclampsia	24.8	18.1
Pakistan	PAK	Thermal protection	65.1	50.1
Pakistan	PAK	Clean cord care	62.9	48.4
Pakistan	PAK	Clean birth environment	54.0	41.6
Pakistan	PAK	Immediate drying and additional stimulation	60.3	46.4
Pakistan	PAK	Neonatal resuscitation	36.2	27.9
Pakistan	PAK	Antibiotics for preterm or prolonged PROM	49.3	37.9
Pakistan	PAK	Parenteral administration of anti-convulsants	47.1	36.2
Pakistan	PAK	Parenteral administration of uterotonics	58.9	45.3
Pakistan	PAK	Parenteral administration of antibiotics	49.3	37.9
Pakistan	PAK	Assisted vaginal delivery	16.7	12.9
Pakistan	PAK	Manual removal of placenta	24.6	18.9
Pakistan	PAK	Removal of retained products of conception	21.8	16.8
Pakistan	PAK	Cesarean delivery	5.7	4.4
Pakistan	PAK	Blood transfusion	8.3	6.4
Pakistan	PAK	Induction of labor for pregnancies lasting 41+ weeks	1.2	0.9
Pakistan	PAK	Complementary feeding - education only	20.8	16.1
Pakistan	PAK	Complementary feeding - supplementary feeding and education	20.8	16.1
Pakistan	PAK	Vitamin A supplementation	92.0	71.0
Pakistan	PAK	Improved sanitation - Utilization of latrines or toilets	59.9	59.9
Pakistan	PAK	Improved water source	91.5	91.5
Pakistan	PAK	Water connection in the home	26.0	26.0
Pakistan	PAK	Hand washing with soap	65.6	65.6

Pakistan	PAK	Hygienic disposal of children's stools	35.7	35.7
Pakistan	PAK	ITN/IRS - Households protected from malaria	8.4	6.5
Pakistan	PAK	Injectable antibiotics for neonatal sepsis	65.9	48.2
Pakistan	PAK	ORS - oral rehydration solution	37.4	28.8
Pakistan	PAK	Antibiotics for treatment of dysentery	33.7	25.9
Pakistan	PAK	Zinc for treatment of diarrhea	12.5	9.6
Pakistan	PAK	Oral antibiotics for pneumonia	78.6	60.5
Pakistan	PAK	Vitamin A for treatment of measles	92.0	70.8
Pakistan	PAK	BCG vaccine	86.0	62.9
Pakistan	PAK	Polio vaccine	75.0	54.8
Pakistan	PAK	DPT vaccine	75.0	54.8
Pakistan	PAK	H. influenzae type b vaccine	75.0	68.5
Pakistan	PAK	HepB vaccine	75.0	54.8
Pakistan	PAK	Pneumococcal vaccine	79.0	72.2
Pakistan	PAK	Rotavirus vaccine	58.0	53.0
Pakistan	PAK	Measles vaccine	76.0	55.6
Pakistan	PAK	Global wasting (<-2 SD) rate	7.0	8.4
Pakistan	PAK	Contraceptive prevalence (CPR)	37.05	30.1
Panama	PAN	Safe abortion services	0.6	0.5
Panama	PAN	Syphilis detection and treatment	23.1	16.9
Panama	PAN	Hypertensive disorder case management	21.1	15.4
Panama	PAN	Diabetes case management	16.5	12.1
Panama	PAN	Malaria case management	68.1	49.8
Panama	PAN	MgSO4 management of pre-eclampsia	41.8	30.6
Panama	PAN	Thermal protection	90.2	69.4
Panama	PAN	Clean cord care	87.0	66.9
Panama	PAN	Clean birth environment	74.8	57.6
Panama	PAN	Immediate drying and additional stimulation	83.5	64.3
Panama	PAN	Neonatal resuscitation	50.2	38.6
Panama	PAN	Antibiotics for preterm or prolonged PROM	68.3	52.6
Panama	PAN	Parenteral administration of anti-convulsants	65.2	50.2
Panama	PAN	Parenteral administration of uterotonics	81.5	62.7
Panama	PAN	Parenteral administration of antibiotics	68.3	52.6
Panama	PAN	Assisted vaginal delivery	23.1	17.8
Panama	PAN	Manual removal of placenta	34.1	26.2
Panama	PAN	Removal of retained products of conception	30.2	23.2
Panama	PAN	Cesarean delivery	7.9	6.1
Panama	PAN	Blood transfusion	11.5	8.8
Panama	PAN	Induction of labor for pregnancies lasting 41+ weeks	1.6	1.2
Panama	PAN	Improved sanitation - Utilization of latrines or toilets	83.3	83.3
Panama	PAN	Improved water source	96.4	96.4
Panama	PAN	Water connection in the home	89.6	89.6

Panama	PAN	Hygienic disposal of children's stools	42.6	42.6
Panama	PAN	Injectable antibiotics for neonatal sepsis	91.2	66.7
Panama	PAN	ORS - oral rehydration solution	52.4	40.3
Panama	PAN	Oral antibiotics for pneumonia	81.6	62.8
Panama	PAN	SAM - treatment for severe acute malnutrition	4.5	3.5
Panama	PAN	BCG vaccine	99.0	72.4
Panama	PAN	Polio vaccine	88.0	64.3
Panama	PAN	DPT vaccine	88.0	64.3
Panama	PAN	H. influenzae type b vaccine	88.0	80.4
Panama	PAN	HepB vaccine	88.0	64.3
Panama	PAN	Pneumococcal vaccine	92.0	84.1
Panama	PAN	Rotavirus vaccine	95.0	86.8
Panama	PAN	Measles vaccine	98.0	71.6
Panama	PAN	Global wasting (<-2 SD) rate	2.4	2.9
Panama	PAN	Contraceptive prevalence (CPR)	64	52.0
Papua New Guinea	PNG	Safe abortion services	85.0	69.0
Papua New Guinea	PNG	TT - Tetanus toxoid vaccination	70.0	51.2
Papua New Guinea	PNG	Syphilis detection and treatment	17.9	13.1
Papua New Guinea	PNG	Hypertensive disorder case management	11.8	8.6
Papua New Guinea	PNG	Diabetes case management	9.2	6.7
Papua New Guinea	PNG	Malaria case management	38.0	27.8
Papua New Guinea	PNG	MgSO4 management of pre-eclampsia	23.3	17.0
Papua New Guinea	PNG	Thermal protection	54.1	41.6
Papua New Guinea	PNG	Clean cord care	52.2	40.2
Papua New Guinea	PNG	Clean birth environment	44.9	34.6
Papua New Guinea	PNG	Immediate drying and additional stimulation	50.1	38.6
Papua New Guinea	PNG	Neonatal resuscitation	30.1	23.2
Papua New Guinea	PNG	Antibiotics for preterm or prolonged PROM	40.9	31.5
Papua New Guinea	PNG	Parenteral administration of anti-convulsants	39.1	30.1
Papua New Guinea	PNG	Parenteral administration of uterotonics	48.9	37.6
Papua New Guinea	PNG	Parenteral administration of antibiotics	40.9	31.5
Papua New Guinea	PNG	Assisted vaginal delivery	13.8	10.6
Papua New Guinea	PNG	Manual removal of placenta	20.5	15.8
Papua New Guinea	PNG	Removal of retained products of conception	18.1	13.9
Papua New Guinea	PNG	Cesarean delivery	4.8	3.7
Papua New Guinea	PNG	Blood transfusion	6.9	5.3
Papua New Guinea	PNG	Induction of labor for pregnancies lasting 41+ weeks	1.0	0.8
Papua New Guinea	PNG	Complementary feeding - education only	57.1	44.1
Papua New Guinea	PNG	Complementary feeding - supplementary feeding and education	57.1	44.1
Papua New Guinea	PNG	Vitamin A supplementation	15.0	11.6
Papua New Guinea	PNG	Improved sanitation - Utilization of latrines or toilets	12.9	12.9

Papua New Guinea	PNG	Improved water source	41.3	41.3
Papua New Guinea	PNG	Water connection in the home	7.6	7.6
Papua New Guinea	PNG	Hygienic disposal of children's stools	33.2	33.2
Papua New Guinea	PNG	ITN/IRS - Households protected from malaria	32.9	25.4
Papua New Guinea	PNG	Injectable antibiotics for neonatal sepsis	54.7	40.0
Papua New Guinea	PNG	ORS - oral rehydration solution	30.0	23.1
Papua New Guinea	PNG	Zinc for treatment of diarrhea	7.3	5.6
Papua New Guinea	PNG	Oral antibiotics for pneumonia	63.0	48.5
Papua New Guinea	PNG	Vitamin A for treatment of measles	15.0	11.5
Papua New Guinea	PNG	SAM - treatment for severe acute malnutrition	1.7	1.3
Papua New Guinea	PNG	BCG vaccine	69.0	50.4
Papua New Guinea	PNG	Polio vaccine	67.0	49.0
Papua New Guinea	PNG	DPT vaccine	61.0	44.6
Papua New Guinea	PNG	H. influenzae type b vaccine	61.0	55.7
Papua New Guinea	PNG	HepB vaccine	61.0	44.6
Papua New Guinea	PNG	Pneumococcal vaccine	43.0	39.3
Papua New Guinea	PNG	Measles vaccine	62.0	45.3
Papua New Guinea	PNG	Global wasting (<-2 SD) rate	10.6	12.7
Papua New Guinea	PNG	Contraceptive prevalence (CPR)	38.05	30.9
Paraguay	PRY	Safe abortion services	0.2	0.2
Paraguay	PRY	TT - Tetanus toxoid vaccination	95.0	69.4
Paraguay	PRY	Syphilis detection and treatment	24.4	17.8
Paraguay	PRY	Hypertensive disorder case management	22.5	16.4
Paraguay	PRY	Diabetes case management	17.5	12.8
Paraguay	PRY	Malaria case management	72.5	53.0
Paraguay	PRY	MgSO4 management of pre-eclampsia	44.5	32.5
Paraguay	PRY	Thermal protection	92.1	70.9
Paraguay	PRY	Clean cord care	88.9	68.4
Paraguay	PRY	Clean birth environment	76.4	58.8
Paraguay	PRY	Immediate drying and additional stimulation	85.3	65.6
Paraguay	PRY	Neonatal resuscitation	51.3	39.5
Paraguay	PRY	Antibiotics for preterm or prolonged PROM	69.8	53.7
Paraguay	PRY	Parenteral administration of anti-convulsants	66.7	51.3
Paraguay	PRY	Parenteral administration of uterotonics	83.3	64.1
Paraguay	PRY	Parenteral administration of antibiotics	69.8	53.7
Paraguay	PRY	Assisted vaginal delivery	23.6	18.2
Paraguay	PRY	Manual removal of placenta	34.9	26.9
Paraguay	PRY	Removal of retained products of conception	30.9	23.8
Paraguay	PRY	Cesarean delivery	8.1	6.2
Paraguay	PRY	Blood transfusion	11.7	9.0
Paraguay	PRY	Induction of labor for pregnancies lasting 41+ weeks	1.6	1.2
Paraguay	PRY	Complementary feeding - education only	70.4	54.3

Paraguay	PRY	Complementary feeding - supplementary feeding and education	70.4	54.3
Paraguay	PRY	Improved sanitation - Utilization of latrines or toilets	89.8	89.8
Paraguay	PRY	Improved water source	99.0	99.0
Paraguay	PRY	Water connection in the home	92.7	92.7
Paraguay	PRY	Hygienic disposal of children's stools	22.2	22.2
Paraguay	PRY	Injectable antibiotics for neonatal sepsis	93.2	68.1
Paraguay	PRY	ORS - oral rehydration solution	28.2	21.7
Paraguay	PRY	Zinc for treatment of diarrhea	7.1	5.5
Paraguay	PRY	Oral antibiotics for pneumonia	89.4	68.8
Paraguay	PRY	BCG vaccine	91.0	66.5
Paraguay	PRY	Polio vaccine	88.0	64.3
Paraguay	PRY	DPT vaccine	88.0	64.3
Paraguay	PRY	H. influenzae type b vaccine	88.0	80.4
Paraguay	PRY	HepB vaccine	88.0	64.3
Paraguay	PRY	Pneumococcal vaccine	94.0	85.9
Paraguay	PRY	Rotavirus vaccine	91.0	83.2
Paraguay	PRY	Measles vaccine	92.0	67.3
Paraguay	PRY	Global wasting (<-2 SD) rate	1.0	1.2
Paraguay	PRY	Contraceptive prevalence (CPR)	69.85	56.7
Peru	PER	Safe abortion services	0.2	0.2
Peru	PER	TT - Tetanus toxoid vaccination	95.0	69.4
Peru	PER	Syphilis detection and treatment	24.1	17.6
Peru	PER	Iron supplementation in pregnancy	59.7	43.6
Peru	PER	Hypertensive disorder case management	23.0	16.8
Peru	PER	Diabetes case management	17.9	13.1
Peru	PER	Malaria case management	74.2	54.2
Peru	PER	MgSO4 management of pre-eclampsia	45.5	33.3
Peru	PER	Thermal protection	89.7	69.0
Peru	PER	Clean cord care	86.6	66.6
Peru	PER	Clean birth environment	74.4	57.3
Peru	PER	Immediate drying and additional stimulation	83.1	63.9
Peru	PER	Neonatal resuscitation	49.9	38.4
Peru	PER	Antibiotics for preterm or prolonged PROM	67.9	52.2
Peru	PER	Parenteral administration of anti-convulsants	64.9	49.9
Peru	PER	Parenteral administration of uterotonics	81.1	62.4
Peru	PER	Parenteral administration of antibiotics	67.9	52.2
Peru	PER	Assisted vaginal delivery	22.9	17.6
Peru	PER	Manual removal of placenta	33.9	26.1
Peru	PER	Removal of retained products of conception	30.1	23.2
Peru	PER	Cesarean delivery	7.9	6.1
Peru	PER	Blood transfusion	11.4	8.8

Peru	PER	Induction of labor for pregnancies lasting 41+ weeks	1.6	1.2
Peru	PER	Complementary feeding - education only	82.6	63.7
Peru	PER	Complementary feeding - supplementary feeding and education	82.6	63.7
Peru	PER	Vitamin A supplementation	4.5	3.5
Peru	PER	Improved sanitation - Utilization of latrines or toilets	74.3	74.3
Peru	PER	Improved water source	91.1	91.1
Peru	PER	Water connection in the home	79.4	79.4
Peru	PER	Hand washing with soap	14.0	14.0
Peru	PER	Hygienic disposal of children's stools	12.8	12.8
Peru	PER	Injectable antibiotics for neonatal sepsis	90.7	66.3
Peru	PER	ORS - oral rehydration solution	34.6	26.6
Peru	PER	Antibiotics for treatment of dysentery	18.9	14.5
Peru	PER	Zinc for treatment of diarrhea	0.9	0.7
Peru	PER	Oral antibiotics for pneumonia	67.5	51.9
Peru	PER	Vitamin A for treatment of measles	4.5	3.5
Peru	PER	BCG vaccine	81.0	59.2
Peru	PER	Polio vaccine	83.0	60.7
Peru	PER	DPT vaccine	84.0	61.4
Peru	PER	H. influenzae type b vaccine	84.0	76.8
Peru	PER	HepB vaccine	84.0	61.4
Peru	PER	Pneumococcal vaccine	82.0	74.9
Peru	PER	Rotavirus vaccine	85.0	77.7
Peru	PER	Measles vaccine	83.0	60.7
Peru	PER	Global wasting (<-2 SD) rate	0.7	0.8
Peru	PER	Contraceptive prevalence (CPR)	75.5	61.3
Philippines	PHL	Safe abortion services	39.0	31.7
Philippines	PHL	TT - Tetanus toxoid vaccination	90.0	65.8
Philippines	PHL	Syphilis detection and treatment	23.1	16.9
Philippines	PHL	Iron supplementation in pregnancy	50.6	37.0
Philippines	PHL	Hypertensive disorder case management	20.7	15.1
Philippines	PHL	Diabetes case management	16.2	11.8
Philippines	PHL	Malaria case management	66.8	48.8
Philippines	PHL	MgSO4 management of pre-eclampsia	41.0	30.0
Philippines	PHL	Thermal protection	76.8	59.1
Philippines	PHL	Clean cord care	74.2	57.1
Philippines	PHL	Clean birth environment	63.8	49.1
Philippines	PHL	Immediate drying and additional stimulation	71.2	54.8
Philippines	PHL	Neonatal resuscitation	42.8	32.9
Philippines	PHL	Antibiotics for preterm or prolonged PROM	58.2	44.8
Philippines	PHL	Parenteral administration of anti-convulsants	55.6	42.8
Philippines	PHL	Parenteral administration of uterotonics	69.5	53.5

Philippines	PHL	Parenteral administration of antibiotics	58.2	44.8
Philippines	PHL	Assisted vaginal delivery	19.7	15.2
Philippines	PHL	Manual removal of placenta	29.1	22.4
Philippines	PHL	Removal of retained products of conception	25.8	19.9
Philippines	PHL	Cesarean delivery	6.8	5.2
Philippines	PHL	Blood transfusion	9.8	7.5
Philippines	PHL	Induction of labor for pregnancies lasting 41+ weeks	1.4	1.1
Philippines	PHL	Complementary feeding - education only	64.8	50.0
Philippines	PHL	Complementary feeding - supplementary feeding and education	64.8	50.0
Philippines	PHL	Vitamin A supplementation	68.0	52.5
Philippines	PHL	Improved sanitation - Utilization of latrines or toilets	76.5	76.5
Philippines	PHL	Improved water source	93.6	93.6
Philippines	PHL	Water connection in the home	37.5	37.5
Philippines	PHL	Hand washing with soap	86.6	86.6
Philippines	PHL	Hygienic disposal of children's stools	10.2	10.2
Philippines	PHL	Injectable antibiotics for neonatal sepsis	77.7	56.8
Philippines	PHL	ORS - oral rehydration solution	44.8	34.5
Philippines	PHL	Antibiotics for treatment of dysentery	38.7	29.8
Philippines	PHL	Zinc for treatment of diarrhea	24.4	18.8
Philippines	PHL	Oral antibiotics for pneumonia	66.6	51.2
Philippines	PHL	Vitamin A for treatment of measles	68.0	52.3
Philippines	PHL	SAM - treatment for severe acute malnutrition	0.1	0.1
Philippines	PHL	BCG vaccine	75.0	54.8
Philippines	PHL	Polio vaccine	66.0	48.2
Philippines	PHL	DPT vaccine	65.0	47.5
Philippines	PHL	H. influenzae type b vaccine	65.0	59.4
Philippines	PHL	HepB vaccine	65.0	47.5
Philippines	PHL	Pneumococcal vaccine	43.0	39.3
Philippines	PHL	Rotavirus vaccine	2.0	1.8
Philippines	PHL	Measles vaccine	89.0	65.1
Philippines	PHL	Global wasting (<-2 SD) rate	6.9	8.3
Philippines	PHL	Contraceptive prevalence (CPR)	56	45.5
Republic of Moldova	MDA	Safe abortion services	87.5	71.1
Republic of Moldova	MDA	Syphilis detection and treatment	24.4	17.8
Republic of Moldova	MDA	Iron supplementation in pregnancy	40.9	29.9
Republic of Moldova	MDA	Hypertensive disorder case management	22.9	16.7
Republic of Moldova	MDA	Diabetes case management	17.9	13.1
Republic of Moldova	MDA	Malaria case management	73.9	54.0
Republic of Moldova	MDA	MgSO4 management of pre-eclampsia	45.3	33.1
Republic of Moldova	MDA	Thermal protection	97.7	75.2
Republic of Moldova	MDA	Clean cord care	94.3	72.6

Republic of Moldova	MDA	Clean birth environment	81.1	62.4
Republic of Moldova	MDA	Immediate drying and additional stimulation	90.5	69.6
Republic of Moldova	MDA	Neonatal resuscitation	54.4	41.9
Republic of Moldova	MDA	Antibiotics for preterm or prolonged PROM	74.0	56.9
Republic of Moldova	MDA	Parenteral administration of anti-convulsants	70.7	54.4
Republic of Moldova	MDA	Parenteral administration of uterotonics	88.3	67.9
Republic of Moldova	MDA	Parenteral administration of antibiotics	74.0	56.9
Republic of Moldova	MDA	Assisted vaginal delivery	25.0	19.2
Republic of Moldova	MDA	Manual removal of placenta	37.0	28.5
Republic of Moldova	MDA	Removal of retained products of conception	32.8	25.2
Republic of Moldova	MDA	Cesarean delivery	8.6	6.6
Republic of Moldova	MDA	Blood transfusion	12.4	9.5
Republic of Moldova	MDA	Induction of labor for pregnancies lasting 41+ weeks	1.7	1.3
Republic of Moldova	MDA	Complementary feeding - education only	82.0	63.3
Republic of Moldova	MDA	Complementary feeding - supplementary feeding and education	82.0	63.3
Republic of Moldova	MDA	Improved sanitation - Utilization of latrines or toilets	76.3	76.3
Republic of Moldova	MDA	Improved water source	89.1	89.1
Republic of Moldova	MDA	Water connection in the home	58.9	58.9
Republic of Moldova	MDA	Hand washing with soap	89.4	89.4
Republic of Moldova	MDA	Hygienic disposal of children's stools	45.9	45.9
Republic of Moldova	MDA	Injectable antibiotics for neonatal sepsis	98.9	72.3
Republic of Moldova	MDA	ORS - oral rehydration solution	41.5	31.9
Republic of Moldova	MDA	Antibiotics for treatment of dysentery	24.0	18.5
Republic of Moldova	MDA	Oral antibiotics for pneumonia	79.2	60.9
Republic of Moldova	MDA	BCG vaccine	96.0	70.2
Republic of Moldova	MDA	Polio vaccine	94.0	68.7
Republic of Moldova	MDA	DPT vaccine	93.0	68.0
Republic of Moldova	MDA	H. influenzae type b vaccine	92.0	84.1
Republic of Moldova	MDA	HepB vaccine	94.0	68.7
Republic of Moldova	MDA	Pneumococcal vaccine	94.0	85.9
Republic of Moldova	MDA	Rotavirus vaccine	75.0	68.5
Republic of Moldova	MDA	Measles vaccine	93.0	68.0
Republic of Moldova	MDA	Global wasting (<-2 SD) rate	1.9	2.3
Republic of Moldova	MDA	Contraceptive prevalence (CPR)	62.9	51.1
Republic of North Macedonia	MKD	Safe abortion services	100.0	81.2
Republic of North Macedonia	MKD	Syphilis detection and treatment	24.4	17.8
Republic of North Macedonia	MKD	Hypertensive disorder case management	22.6	16.5
Republic of North Macedonia	MKD	Diabetes case management	17.6	12.9

Republic of North Macedonia	MKD	Malaria case management	72.8	53.2
Republic of North Macedonia	MKD	MgSO4 management of pre-eclampsia	44.6	32.6
Republic of North Macedonia	MKD	Thermal protection	97.1	74.7
Republic of North Macedonia	MKD	Clean cord care	93.8	72.2
Republic of North Macedonia	MKD	Clean birth environment	80.6	62.0
Republic of North Macedonia	MKD	Immediate drying and additional stimulation	90.0	69.3
Republic of North Macedonia	MKD	Neonatal resuscitation	54.0	41.6
Republic of North Macedonia	MKD	Antibiotics for preterm or prolonged PROM	73.5	56.6
Republic of North Macedonia	MKD	Parenteral administration of anti-convulsants	70.3	54.1
Republic of North Macedonia	MKD	Parenteral administration of uterotonics	87.8	67.6
Republic of North Macedonia	MKD	Parenteral administration of antibiotics	73.5	56.6
Republic of North Macedonia	MKD	Assisted vaginal delivery	24.8	19.1
Republic of North Macedonia	MKD	Manual removal of placenta	36.8	28.3
Republic of North Macedonia	MKD	Removal of retained products of conception	32.6	25.1
Republic of North Macedonia	MKD	Cesarean delivery	8.6	6.6
Republic of North Macedonia	MKD	Blood transfusion	12.4	9.5
Republic of North Macedonia	MKD	Induction of labor for pregnancies lasting 41+ weeks	1.7	1.3
Republic of North Macedonia	MKD	Improved sanitation - Utilization of latrines or toilets	99.0	99.0
Republic of North Macedonia	MKD	Improved water source	93.1	93.1
Republic of North Macedonia	MKD	Water connection in the home	86.2	86.2
Republic of North Macedonia	MKD	Hygienic disposal of children's stools	17.3	17.3
Republic of North Macedonia	MKD	Injectable antibiotics for neonatal sepsis	98.3	71.9
Republic of North Macedonia	MKD	ORS - oral rehydration solution	62.0	47.7
Republic of North Macedonia	MKD	Oral antibiotics for pneumonia	77.4	59.6
Republic of North Macedonia	MKD	BCG vaccine	97.0	70.9
Republic of North Macedonia	MKD	Polio vaccine	91.0	66.5
Republic of North Macedonia	MKD	DPT vaccine	91.0	66.5

Republic of North Macedonia	MKD	H. influenzae type b vaccine	91.0	83.2
Republic of North Macedonia	MKD	HepB vaccine	91.0	66.5
Republic of North Macedonia	MKD	Measles vaccine	83.0	60.7
Republic of North Macedonia	MKD	Global wasting (<-2 SD) rate	1.8	2.2
Republic of North Macedonia	MKD	Contraceptive prevalence (CPR)	46.35	37.6
Rwanda	RWA	Safe abortion services	3.3	2.7
Rwanda	RWA	TT - Tetanus toxoid vaccination	90.0	65.8
Rwanda	RWA	IPTp - Intermittent preventive treatment of malaria during pregnancy	17.7	12.9
Rwanda	RWA	Syphilis detection and treatment	52.0	38.0
Rwanda	RWA	Iron supplementation in pregnancy	3.4	2.5
Rwanda	RWA	Hypertensive disorder case management	1.9	1.4
Rwanda	RWA	Diabetes case management	8.3	6.1
Rwanda	RWA	Malaria case management	34.4	25.1
Rwanda	RWA	MgSO4 management of pre-eclampsia	7.7	5.6
Rwanda	RWA	Thermal protection	89.6	68.9
Rwanda	RWA	Clean cord care	81.4	62.6
Rwanda	RWA	Clean birth environment	78.6	60.5
Rwanda	RWA	Immediate drying and additional stimulation	73.4	56.5
Rwanda	RWA	Neonatal resuscitation	36.2	27.9
Rwanda	RWA	Antibiotics for preterm or prolonged PROM	36.7	28.2
Rwanda	RWA	Parenteral administration of anti-convulsants	60.1	46.2
Rwanda	RWA	Parenteral administration of uterotonics	33.5	25.8
Rwanda	RWA	Parenteral administration of antibiotics	36.7	28.2
Rwanda	RWA	Assisted vaginal delivery	20.3	15.6
Rwanda	RWA	Manual removal of placenta	25.7	19.8
Rwanda	RWA	Removal of retained products of conception	30.0	23.1
Rwanda	RWA	Cesarean delivery	12.1	9.3
Rwanda	RWA	Blood transfusion	6.0	4.6
Rwanda	RWA	Induction of labor for pregnancies lasting 41+ weeks	1.6	1.2
Rwanda	RWA	Complementary feeding - education only	30.1	23.2
Rwanda	RWA	Complementary feeding - supplementary feeding and education	30.1	23.2
Rwanda	RWA	Vitamin A supplementation	98.0	75.6
Rwanda	RWA	Improved sanitation - Utilization of latrines or toilets	66.6	66.6
Rwanda	RWA	Improved water source	57.7	57.7
Rwanda	RWA	Water connection in the home	21.5	21.5
Rwanda	RWA	Hand washing with soap	37.6	37.6
Rwanda	RWA	Hygienic disposal of children's stools	77.9	77.9
Rwanda	RWA	ITN/IRS - Households protected from malaria	84.1	64.9

Rwanda	RWA	Injectable antibiotics for neonatal sepsis	90.7	66.3
Rwanda	RWA	ORS - oral rehydration solution	27.5	21.2
Rwanda	RWA	Antibiotics for treatment of dysentery	15.1	11.6
Rwanda	RWA	Zinc for treatment of diarrhea	0.2	0.2
Rwanda	RWA	Oral antibiotics for pneumonia	53.9	41.5
Rwanda	RWA	Vitamin A for treatment of measles	98.0	75.4
Rwanda	RWA	ACTs- Artemisinin compounds for treatment of malaria	7.4	5.7
Rwanda	RWA	BCG vaccine	97.0	70.9
Rwanda	RWA	Polio vaccine	97.0	70.9
Rwanda	RWA	DPT vaccine	89.0	65.1
Rwanda	RWA	H. influenzae type b vaccine	97.0	88.7
Rwanda	RWA	HepB vaccine	97.0	70.9
Rwanda	RWA	Pneumococcal vaccine	97.0	88.7
Rwanda	RWA	Rotavirus vaccine	98.0	89.6
Rwanda	RWA	Measles vaccine	95.0	69.4
Rwanda	RWA	Global wasting (<-2 SD) rate	2.3	2.8
Rwanda	RWA	Contraceptive prevalence (CPR)	57.35	46.6
Saint Lucia	LCA	Safe abortion services	53.7	43.6
Saint Lucia	LCA	Syphilis detection and treatment	24.0	17.5
Saint Lucia	LCA	Hypertensive disorder case management	21.7	15.9
Saint Lucia	LCA	Diabetes case management	16.9	12.4
Saint Lucia	LCA	Malaria case management	70.0	51.2
Saint Lucia	LCA	MgSO4 management of pre-eclampsia	42.9	31.4
Saint Lucia	LCA	Thermal protection	97.6	75.1
Saint Lucia	LCA	Clean cord care	94.2	72.5
Saint Lucia	LCA	Clean birth environment	81.0	62.3
Saint Lucia	LCA	Immediate drying and additional stimulation	90.4	69.6
Saint Lucia	LCA	Neonatal resuscitation	54.3	41.8
Saint Lucia	LCA	Antibiotics for preterm or prolonged PROM	73.9	56.9
Saint Lucia	LCA	Parenteral administration of anti-convulsants	70.6	54.3
Saint Lucia	LCA	Parenteral administration of uterotonics	88.2	67.9
Saint Lucia	LCA	Parenteral administration of antibiotics	73.9	56.9
Saint Lucia	LCA	Assisted vaginal delivery	25.0	19.2
Saint Lucia	LCA	Manual removal of placenta	36.9	28.4
Saint Lucia	LCA	Removal of retained products of conception	32.7	25.2
Saint Lucia	LCA	Cesarean delivery	8.6	6.6
Saint Lucia	LCA	Blood transfusion	12.4	9.5
Saint Lucia	LCA	Induction of labor for pregnancies lasting 41+ weeks	1.7	1.3
Saint Lucia	LCA	Improved sanitation - Utilization of latrines or toilets	88.4	88.4
Saint Lucia	LCA	Improved water source	98.2	98.2
Saint Lucia	LCA	Water connection in the home	94.2	94.2

Saint Lucia	LCA	Hand washing with soap	89.0	89.0
Saint Lucia	LCA	Hygienic disposal of children's stools	26.6	26.6
Saint Lucia	LCA	Injectable antibiotics for neonatal sepsis	98.7	72.2
Saint Lucia	LCA	Oral antibiotics for pneumonia	27.0	20.8
Saint Lucia	LCA	BCG vaccine	99.0	72.4
Saint Lucia	LCA	Polio vaccine	95.0	69.4
Saint Lucia	LCA	DPT vaccine	95.0	69.4
Saint Lucia	LCA	H. influenzae type b vaccine	95.0	86.8
Saint Lucia	LCA	HepB vaccine	95.0	69.4
Saint Lucia	LCA	Measles vaccine	87.0	63.6
Saint Lucia	LCA	Global wasting (<-2 SD) rate	2.4	2.9
Saint Lucia	LCA	Contraceptive prevalence (CPR)	59.4	48.2
Samoa	WSM	Safe abortion services	85.0	69.0
Samoa	WSM	Syphilis detection and treatment	14.4	10.5
Samoa	WSM	Iron supplementation in pregnancy	3.4	2.5
Samoa	WSM	Hypertensive disorder case management	14.0	10.2
Samoa	WSM	Diabetes case management	10.9	8.0
Samoa	WSM	Malaria case management	45.3	33.1
Samoa	WSM	MgSO4 management of pre-eclampsia	27.8	20.3
Samoa	WSM	Thermal protection	79.6	61.3
Samoa	WSM	Clean cord care	76.8	59.1
Samoa	WSM	Clean birth environment	66.0	50.8
Samoa	WSM	Immediate drying and additional stimulation	73.7	56.7
Samoa	WSM	Neonatal resuscitation	44.3	34.1
Samoa	WSM	Antibiotics for preterm or prolonged PROM	60.2	46.3
Samoa	WSM	Parenteral administration of anti-convulsants	57.6	44.3
Samoa	WSM	Parenteral administration of uterotonics	71.9	55.3
Samoa	WSM	Parenteral administration of antibiotics	60.2	46.3
Samoa	WSM	Assisted vaginal delivery	20.3	15.6
Samoa	WSM	Manual removal of placenta	30.1	23.2
Samoa	WSM	Removal of retained products of conception	26.7	20.5
Samoa	WSM	Cesarean delivery	7.0	5.4
Samoa	WSM	Blood transfusion	10.1	7.8
Samoa	WSM	Induction of labor for pregnancies lasting 41+ weeks	1.4	1.1
Samoa	WSM	Improved sanitation - Utilization of latrines or toilets	98.2	98.2
Samoa	WSM	Improved water source	97.4	97.4
Samoa	WSM	Water connection in the home	82.7	82.7
Samoa	WSM	Hygienic disposal of children's stools	38.0	38.0
Samoa	WSM	Injectable antibiotics for neonatal sepsis	80.5	58.8
Samoa	WSM	ORS - oral rehydration solution	68.0	52.3
Samoa	WSM	Antibiotics for treatment of dysentery	1.0	0.8
Samoa	WSM	BCG vaccine	62.0	45.3

Samoa	WSM	Polio vaccine	31.0	22.7
Samoa	WSM	DPT vaccine	34.0	24.9
Samoa	WSM	H. influenzae type b vaccine	34.0	31.1
Samoa	WSM	HepB vaccine	34.0	24.9
Samoa	WSM	Measles vaccine	58.0	42.4
Samoa	WSM	Global wasting (<-2 SD) rate	10.6	12.7
Samoa	WSM	Contraceptive prevalence (CPR)	30.2	24.5
São Tomé and Príncipe	STP	TT - Tetanus toxoid vaccination	99.0	72.4
São Tomé and Príncipe	STP	IPTp - Intermittent preventive treatment of malaria during pregnancy	56.9	41.6
São Tomé and Príncipe	STP	Syphilis detection and treatment	24.1	17.6
São Tomé and Príncipe	STP	Iron supplementation in pregnancy	53.5	39.1
São Tomé and Príncipe	STP	Hypertensive disorder case management	20.1	14.7
São Tomé and Príncipe	STP	Diabetes case management	15.7	11.5
São Tomé and Príncipe	STP	Malaria case management	64.8	47.4
São Tomé and Príncipe	STP	MgSO4 management of pre-eclampsia	39.8	29.1
São Tomé and Príncipe	STP	Thermal protection	89.9	69.2
São Tomé and Príncipe	STP	Clean cord care	86.8	66.8
São Tomé and Príncipe	STP	Clean birth environment	74.6	57.4
São Tomé and Príncipe	STP	Immediate drying and additional stimulation	83.3	64.1
São Tomé and Príncipe	STP	Neonatal resuscitation	50.0	38.5
São Tomé and Príncipe	STP	Antibiotics for preterm or prolonged PROM	68.1	52.4
São Tomé and Príncipe	STP	Parenteral administration of anti-convulsants	65.1	50.1
São Tomé and Príncipe	STP	Parenteral administration of uterotonics	81.3	62.6
São Tomé and Príncipe	STP	Parenteral administration of antibiotics	68.1	52.4
São Tomé and Príncipe	STP	Assisted vaginal delivery	23.0	17.7
São Tomé and Príncipe	STP	Manual removal of placenta	34.0	26.2
São Tomé and Príncipe	STP	Removal of retained products of conception	30.1	23.2
São Tomé and Príncipe	STP	Cesarean delivery	7.9	6.1
São Tomé and Príncipe	STP	Blood transfusion	11.5	8.8
São Tomé and Príncipe	STP	Induction of labor for pregnancies lasting 41+ weeks	1.6	1.2
São Tomé and Príncipe	STP	Complementary feeding - education only	47.4	36.6
São Tomé and Príncipe	STP	Complementary feeding - supplementary feeding and education	47.4	36.6
São Tomé and Príncipe	STP	Vitamin A supplementation	23.0	17.7
São Tomé and Príncipe	STP	Improved sanitation - Utilization of latrines or toilets	43.0	43.0
São Tomé and Príncipe	STP	Improved water source	84.3	84.3
São Tomé and Príncipe	STP	Water connection in the home	79.0	79.0
São Tomé and Príncipe	STP	Hand washing with soap	40.6	40.6
São Tomé and Príncipe	STP	Hygienic disposal of children's stools	28.9	28.9
São Tomé and Príncipe	STP	ITN/IRS - Households protected from malaria	96.7	74.6
São Tomé and Príncipe	STP	Injectable antibiotics for neonatal sepsis	91.0	66.5
São Tomé and Príncipe	STP	ORS - oral rehydration solution	49.1	37.8

São Tomé and Príncipe	STP	Oral antibiotics for pneumonia	68.9	53.0
São Tomé and Príncipe	STP	Vitamin A for treatment of measles	23.0	17.7
São Tomé and Príncipe	STP	ACTs- Artemisinin compounds for treatment of malaria	0.1	0.1
São Tomé and Príncipe	STP	BCG vaccine	96.0	70.2
São Tomé and Príncipe	STP	Polio vaccine	95.0	69.4
São Tomé and Príncipe	STP	DPT vaccine	95.0	69.4
São Tomé and Príncipe	STP	H. influenzae type b vaccine	95.0	86.8
São Tomé and Príncipe	STP	HepB vaccine	95.0	69.4
São Tomé and Príncipe	STP	Pneumococcal vaccine	95.0	86.8
São Tomé and Príncipe	STP	Rotavirus vaccine	95.0	86.8
São Tomé and Príncipe	STP	Measles vaccine	90.0	65.8
São Tomé and Príncipe	STP	Global wasting (<-2 SD) rate	4.2	5.0
São Tomé and Príncipe	STP	Contraceptive prevalence (CPR)	45.3	36.8
Senegal	SEN	TT - Tetanus toxoid vaccination	95.0	69.4
Senegal	SEN	IPTp - Intermittent preventive treatment of malaria during pregnancy	62.6	45.8
Senegal	SEN	Syphilis detection and treatment	5.9	4.3
Senegal	SEN	Iron supplementation in pregnancy	63.1	46.1
Senegal	SEN	Hypertensive disorder case management	0.3	0.2
Senegal	SEN	Diabetes case management	33.5	24.5
Senegal	SEN	Malaria case management	47.7	34.9
Senegal	SEN	MgSO4 management of pre-eclampsia	36.5	26.7
Senegal	SEN	Thermal protection	69.1	53.2
Senegal	SEN	Clean cord care	68.7	52.9
Senegal	SEN	Clean birth environment	68.5	52.7
Senegal	SEN	Immediate drying and additional stimulation	68.9	53.0
Senegal	SEN	Neonatal resuscitation	50.5	38.9
Senegal	SEN	Antibiotics for preterm or prolonged PROM	52.3	40.2
Senegal	SEN	Parenteral administration of anti-convulsants	59.2	45.6
Senegal	SEN	Parenteral administration of uterotonics	61.1	47.0
Senegal	SEN	Parenteral administration of antibiotics	52.3	40.2
Senegal	SEN	Assisted vaginal delivery	7.4	5.7
Senegal	SEN	Manual removal of placenta	36.0	27.7
Senegal	SEN	Removal of retained products of conception	35.1	27.0
Senegal	SEN	Cesarean delivery	6.4	4.9
Senegal	SEN	Blood transfusion	7.6	5.8
Senegal	SEN	Induction of labor for pregnancies lasting 41+ weeks	8.1	6.2
Senegal	SEN	Complementary feeding - education only	25.3	19.5
Senegal	SEN	Complementary feeding - supplementary feeding and education	25.3	19.5
Senegal	SEN	Vitamin A supplementation	58.0	44.8
Senegal	SEN	Improved sanitation - Utilization of latrines or toilets	51.5	51.5

Senegal	SEN	Improved water source	80.7	80.7
Senegal	SEN	Water connection in the home	58.9	58.9
Senegal	SEN	Hand washing with soap	28.5	28.5
Senegal	SEN	Hygienic disposal of children's stools	57.0	57.0
Senegal	SEN	ITN/IRS - Households protected from malaria	84.5	65.2
Senegal	SEN	Injectable antibiotics for neonatal sepsis	69.9	51.1
Senegal	SEN	ORS - oral rehydration solution	27.9	21.5
Senegal	SEN	Antibiotics for treatment of dysentery	12.8	9.8
Senegal	SEN	Zinc for treatment of diarrhea	26.9	20.7
Senegal	SEN	Oral antibiotics for pneumonia	52.0	40.0
Senegal	SEN	Vitamin A for treatment of measles	58.0	44.6
Senegal	SEN	ACTs- Artemisinin compounds for treatment of malaria	1.5	1.2
Senegal	SEN	BCG vaccine	83.0	60.7
Senegal	SEN	Polio vaccine	81.0	59.2
Senegal	SEN	DPT vaccine	81.0	59.2
Senegal	SEN	H. influenzae type b vaccine	82.0	74.9
Senegal	SEN	HepB vaccine	82.0	59.9
Senegal	SEN	Pneumococcal vaccine	81.0	74.0
Senegal	SEN	Rotavirus vaccine	80.0	73.1
Senegal	SEN	Measles vaccine	90.0	65.8
Senegal	SEN	Global wasting (<-2 SD) rate	9.0	10.7
Senegal	SEN	Contraceptive prevalence (CPR)	29.95	24.3
Serbia	SRB	Safe abortion services	100.0	81.2
Serbia	SRB	Syphilis detection and treatment	6.0	4.4
Serbia	SRB	Hypertensive disorder case management	0.6	0.4
Serbia	SRB	Diabetes case management	56.3	41.2
Serbia	SRB	Malaria case management	80.1	58.6
Serbia	SRB	MgSO4 management of pre-eclampsia	61.2	44.7
Serbia	SRB	Thermal protection	97.1	74.7
Serbia	SRB	Clean cord care	93.8	72.2
Serbia	SRB	Clean birth environment	80.6	62.0
Serbia	SRB	Immediate drying and additional stimulation	90.0	69.3
Serbia	SRB	Neonatal resuscitation	54.0	41.6
Serbia	SRB	Antibiotics for preterm or prolonged PROM	73.5	56.6
Serbia	SRB	Parenteral administration of anti-convulsants	70.3	54.1
Serbia	SRB	Parenteral administration of uterotonics	87.8	67.6
Serbia	SRB	Parenteral administration of antibiotics	73.5	56.6
Serbia	SRB	Assisted vaginal delivery	24.8	19.1
Serbia	SRB	Manual removal of placenta	36.8	28.3
Serbia	SRB	Removal of retained products of conception	32.6	25.1
Serbia	SRB	Cesarean delivery	8.6	6.6
Serbia	SRB	Blood transfusion	12.4	9.5

Serbia	SRB	Induction of labor for pregnancies lasting 41+ weeks	1.7	1.3
Serbia	SRB	Complementary feeding - education only	89.6	69.1
Serbia	SRB	Complementary feeding - supplementary feeding and education	89.6	69.1
Serbia	SRB	Improved sanitation - Utilization of latrines or toilets	97.6	97.6
Serbia	SRB	Improved water source	85.5	85.5
Serbia	SRB	Water connection in the home	82.1	82.1
Serbia	SRB	Hand washing with soap	98.6	98.6
Serbia	SRB	Hygienic disposal of children's stools	25.7	25.7
Serbia	SRB	Injectable antibiotics for neonatal sepsis	98.3	71.9
Serbia	SRB	ORS - oral rehydration solution	36.0	27.7
Serbia	SRB	Oral antibiotics for pneumonia	89.7	69.0
Serbia	SRB	BCG vaccine	98.0	71.6
Serbia	SRB	Polio vaccine	96.0	70.2
Serbia	SRB	DPT vaccine	96.0	70.2
Serbia	SRB	H. influenzae type b vaccine	96.0	87.7
Serbia	SRB	HepB vaccine	91.0	66.5
Serbia	SRB	Pneumococcal vaccine	48.0	43.9
Serbia	SRB	Measles vaccine	86.0	62.9
Serbia	SRB	Global wasting (<-2 SD) rate	3.8	4.6
Serbia	SRB	Contraceptive prevalence (CPR)	59.25	48.1
Sierra Leone	SLE	TT - Tetanus toxoid vaccination	90.0	65.8
Sierra Leone	SLE	IPTp - Intermittent preventive treatment of malaria during pregnancy	66.6	48.7
Sierra Leone	SLE	Syphilis detection and treatment	19.9	14.5
Sierra Leone	SLE	Iron supplementation in pregnancy	30.0	21.9
Sierra Leone	SLE	Hypertensive disorder case management	40.8	29.8
Sierra Leone	SLE	Diabetes case management	11.4	8.3
Sierra Leone	SLE	Malaria case management	70.0	51.2
Sierra Leone	SLE	MgSO4 management of pre-eclampsia	67.1	49.1
Sierra Leone	SLE	Thermal protection	75.7	58.3
Sierra Leone	SLE	Clean cord care	69.8	53.7
Sierra Leone	SLE	Clean birth environment	65.3	50.2
Sierra Leone	SLE	Immediate drying and additional stimulation	71.3	54.9
Sierra Leone	SLE	Neonatal resuscitation	32.5	25.0
Sierra Leone	SLE	Antibiotics for preterm or prolonged PROM	66.2	50.9
Sierra Leone	SLE	Parenteral administration of anti-convulsants	67.6	52.0
Sierra Leone	SLE	Parenteral administration of uterotonics	69.5	53.5
Sierra Leone	SLE	Parenteral administration of antibiotics	66.2	50.9
Sierra Leone	SLE	Assisted vaginal delivery	43.5	33.5
Sierra Leone	SLE	Manual removal of placenta	16.2	12.5
Sierra Leone	SLE	Removal of retained products of conception	34.4	26.5
Sierra Leone	SLE	Cesarean delivery	1.7	1.3

Sierra Leone	SLE	Blood transfusion	11.7	9.0
Sierra Leone	SLE	Induction of labor for pregnancies lasting 41+ weeks	0.8	0.6
Sierra Leone	SLE	Complementary feeding - education only	24.2	18.7
Sierra Leone	SLE	Complementary feeding - supplementary feeding and education	24.2	18.7
Sierra Leone	SLE	Vitamin A supplementation	98.0	75.6
Sierra Leone	SLE	Improved sanitation - Utilization of latrines or toilets	15.7	15.7
Sierra Leone	SLE	Improved water source	60.8	60.8
Sierra Leone	SLE	Water connection in the home	14.9	14.9
Sierra Leone	SLE	Hand washing with soap	34.3	34.3
Sierra Leone	SLE	Hygienic disposal of children's stools	63.0	63.0
Sierra Leone	SLE	ITN/IRS - Households protected from malaria	72.0	55.6
Sierra Leone	SLE	Injectable antibiotics for neonatal sepsis	76.6	56.0
Sierra Leone	SLE	ORS - oral rehydration solution	77.7	59.8
Sierra Leone	SLE	Antibiotics for treatment of dysentery	50.5	38.9
Sierra Leone	SLE	Zinc for treatment of diarrhea	50.0	38.5
Sierra Leone	SLE	Oral antibiotics for pneumonia	73.3	56.4
Sierra Leone	SLE	Vitamin A for treatment of measles	98.0	75.4
Sierra Leone	SLE	ACTs- Artemisinin compounds for treatment of malaria	13.9	10.7
Sierra Leone	SLE	BCG vaccine	90.0	65.8
Sierra Leone	SLE	Polio vaccine	90.0	65.8
Sierra Leone	SLE	DPT vaccine	90.0	65.8
Sierra Leone	SLE	H. influenzae type b vaccine	90.0	82.3
Sierra Leone	SLE	HepB vaccine	90.0	65.8
Sierra Leone	SLE	Pneumococcal vaccine	90.0	82.3
Sierra Leone	SLE	Rotavirus vaccine	92.0	84.1
Sierra Leone	SLE	Measles vaccine	80.0	58.5
Sierra Leone	SLE	Global wasting (<-2 SD) rate	6.9	8.3
Sierra Leone	SLE	Contraceptive prevalence (CPR)	23.85	19.4
Solomon Islands	SLB	Safe abortion services	85.0	69.0
Solomon Islands	SLB	TT - Tetanus toxoid vaccination	85.0	62.1
Solomon Islands	SLB	IPTp - Intermittent preventive treatment of malaria during pregnancy	1.2	0.9
Solomon Islands	SLB	Syphilis detection and treatment	19.8	14.5
Solomon Islands	SLB	Hypertensive disorder case management	15.5	11.3
Solomon Islands	SLB	Diabetes case management	12.1	8.8
Solomon Islands	SLB	Malaria case management	50.1	36.6
Solomon Islands	SLB	MgSO4 management of pre-eclampsia	30.7	22.4
Solomon Islands	SLB	Thermal protection	83.5	64.3
Solomon Islands	SLB	Clean cord care	80.6	62.0
Solomon Islands	SLB	Clean birth environment	69.3	53.3
Solomon Islands	SLB	Immediate drying and additional stimulation	77.4	59.6

Solomon Islands	SLB	Neonatal resuscitation	46.5	35.8
Solomon Islands	SLB	Antibiotics for preterm or prolonged PROM	63.2	48.6
Solomon Islands	SLB	Parenteral administration of anti-convulsants	60.4	46.5
Solomon Islands	SLB	Parenteral administration of uterotonics	75.5	58.1
Solomon Islands	SLB	Parenteral administration of antibiotics	63.2	48.6
Solomon Islands	SLB	Assisted vaginal delivery	21.4	16.5
Solomon Islands	SLB	Manual removal of placenta	31.6	24.3
Solomon Islands	SLB	Removal of retained products of conception	28.0	21.5
Solomon Islands	SLB	Cesarean delivery	7.4	5.7
Solomon Islands	SLB	Blood transfusion	10.6	8.2
Solomon Islands	SLB	Induction of labor for pregnancies lasting 41+ weeks	1.5	1.2
Solomon Islands	SLB	Complementary feeding - education only	36.9	28.5
Solomon Islands	SLB	Complementary feeding - supplementary feeding and education	36.9	28.5
Solomon Islands	SLB	Vitamin A supplementation	7.4	5.7
Solomon Islands	SLB	Improved sanitation - Utilization of latrines or toilets	33.5	33.5
Solomon Islands	SLB	Improved water source	67.8	67.8
Solomon Islands	SLB	Water connection in the home	31.3	31.3
Solomon Islands	SLB	Hygienic disposal of children's stools	29.4	29.4
Solomon Islands	SLB	ITN/IRS - Households protected from malaria	48.5	37.4
Solomon Islands	SLB	Injectable antibiotics for neonatal sepsis	84.5	61.8
Solomon Islands	SLB	ORS - oral rehydration solution	37.7	29.0
Solomon Islands	SLB	Antibiotics for treatment of dysentery	3.9	3.0
Solomon Islands	SLB	Vitamin A for treatment of measles	7.4	5.7
Solomon Islands	SLB	BCG vaccine	83.0	60.7
Solomon Islands	SLB	Polio vaccine	85.0	62.1
Solomon Islands	SLB	DPT vaccine	85.0	62.1
Solomon Islands	SLB	H. influenzae type b vaccine	85.0	77.7
Solomon Islands	SLB	HepB vaccine	85.0	62.1
Solomon Islands	SLB	Pneumococcal vaccine	84.0	76.8
Solomon Islands	SLB	Measles vaccine	84.0	61.4
Solomon Islands	SLB	Global wasting (<-2 SD) rate	10.6	12.7
Solomon Islands	SLB	Contraceptive prevalence (CPR)	33.8	27.5
Somalia	SOM	Safe abortion services	3.3	2.7
Somalia	SOM	TT - Tetanus toxoid vaccination	67.0	49.0
Somalia	SOM	IPTp - Intermittent preventive treatment of malaria during pregnancy	0.9	0.7
Somalia	SOM	Syphilis detection and treatment	6.5	4.8
Somalia	SOM	Hypertensive disorder case management	0.5	0.4
Somalia	SOM	Diabetes case management	0.4	0.3
Somalia	SOM	Malaria case management	1.5	1.1
Somalia	SOM	MgSO4 management of pre-eclampsia	0.9	0.7
Somalia	SOM	Thermal protection	9.3	7.2

Somalia	SOM	Clean cord care	8.9	6.8
Somalia	SOM	Clean birth environment	7.7	5.9
Somalia	SOM	Immediate drying and additional stimulation	8.6	6.6
Somalia	SOM	Neonatal resuscitation	5.1	3.9
Somalia	SOM	Antibiotics for preterm or prolonged PROM	7.0	5.4
Somalia	SOM	Parenteral administration of anti-convulsants	6.7	5.2
Somalia	SOM	Parenteral administration of uterotonics	8.4	6.5
Somalia	SOM	Parenteral administration of antibiotics	7.0	5.4
Somalia	SOM	Assisted vaginal delivery	2.4	1.8
Somalia	SOM	Manual removal of placenta	3.5	2.7
Somalia	SOM	Removal of retained products of conception	3.1	2.4
Somalia	SOM	Cesarean delivery	0.8	0.6
Somalia	SOM	Blood transfusion	1.2	0.9
Somalia	SOM	Induction of labor for pregnancies lasting 41+ weeks	0.2	0.2
Somalia	SOM	Complementary feeding - education only	11.0	8.5
Somalia	SOM	Complementary feeding - supplementary feeding and education	11.0	8.5
Somalia	SOM	Vitamin A supplementation	11.0	8.5
Somalia	SOM	Improved sanitation - Utilization of latrines or toilets	38.3	38.3
Somalia	SOM	Improved water source	52.4	52.4
Somalia	SOM	Water connection in the home	21.7	21.7
Somalia	SOM	Hygienic disposal of children's stools	34.7	34.7
Somalia	SOM	ITN/IRS - Households protected from malaria	12.2	9.4
Somalia	SOM	Injectable antibiotics for neonatal sepsis	9.4	6.9
Somalia	SOM	ORS - oral rehydration solution	13.2	10.2
Somalia	SOM	Oral antibiotics for pneumonia	13.0	10.0
Somalia	SOM	Vitamin A for treatment of measles	11.0	8.5
Somalia	SOM	ACTs- Artemisinin compounds for treatment of malaria	0.2	0.2
Somalia	SOM	SAM - treatment for severe acute malnutrition	20.1	15.5
Somalia	SOM	BCG vaccine	37.0	27.0
Somalia	SOM	Polio vaccine	47.0	34.4
Somalia	SOM	DPT vaccine	42.0	30.7
Somalia	SOM	H. influenzae type b vaccine	42.0	38.4
Somalia	SOM	HepB vaccine	42.0	30.7
Somalia	SOM	Measles vaccine	46.0	33.6
Somalia	SOM	Global wasting (<-2 SD) rate	13.3	15.9
Somalia	SOM	Contraceptive prevalence (CPR)	26.75	21.7
South Africa	ZAF	Safe abortion services	42.5	34.5
South Africa	ZAF	TT - Tetanus toxoid vaccination	90.0	65.8
South Africa	ZAF	Syphilis detection and treatment	23.2	17.0
South Africa	ZAF	Iron supplementation in pregnancy	50.5	36.9
South Africa	ZAF	Hypertensive disorder case management	18.4	13.5

South Africa	ZAF	Diabetes case management	14.4	10.5
South Africa	ZAF	Malaria case management	59.4	43.4
South Africa	ZAF	MgSO4 management of pre-eclampsia	36.4	26.6
South Africa	ZAF	Thermal protection	94.8	72.9
South Africa	ZAF	Clean cord care	91.6	70.5
South Africa	ZAF	Clean birth environment	78.7	60.6
South Africa	ZAF	Immediate drying and additional stimulation	87.8	67.6
South Africa	ZAF	Neonatal resuscitation	52.8	40.6
South Africa	ZAF	Antibiotics for preterm or prolonged PROM	71.8	55.3
South Africa	ZAF	Parenteral administration of anti-convulsants	68.6	52.8
South Africa	ZAF	Parenteral administration of uterotonics	85.7	65.9
South Africa	ZAF	Parenteral administration of antibiotics	71.8	55.3
South Africa	ZAF	Assisted vaginal delivery	24.3	18.7
South Africa	ZAF	Manual removal of placenta	35.9	27.6
South Africa	ZAF	Removal of retained products of conception	31.8	24.5
South Africa	ZAF	Cesarean delivery	8.4	6.5
South Africa	ZAF	Blood transfusion	12.1	9.3
South Africa	ZAF	Induction of labor for pregnancies lasting 41+ weeks	1.7	1.3
South Africa	ZAF	Complementary feeding - education only	49.3	38.0
South Africa	ZAF	Complementary feeding - supplementary feeding and education	49.3	38.0
South Africa	ZAF	Vitamin A supplementation	47.0	36.3
South Africa	ZAF	Improved sanitation - Utilization of latrines or toilets	75.7	75.7
South Africa	ZAF	Improved water source	92.7	92.7
South Africa	ZAF	Water connection in the home	83.4	83.4
South Africa	ZAF	Injectable antibiotics for neonatal sepsis	95.9	70.1
South Africa	ZAF	ORS - oral rehydration solution	51.4	39.6
South Africa	ZAF	Antibiotics for treatment of dysentery	8.0	6.2
South Africa	ZAF	Zinc for treatment of diarrhea	36.8	28.3
South Africa	ZAF	Oral antibiotics for pneumonia	65.7	50.6
South Africa	ZAF	Vitamin A for treatment of measles	47.0	36.2
South Africa	ZAF	BCG vaccine	70.0	51.2
South Africa	ZAF	Polio vaccine	74.0	54.1
South Africa	ZAF	DPT vaccine	74.0	54.1
South Africa	ZAF	H. influenzae type b vaccine	74.0	67.6
South Africa	ZAF	HepB vaccine	74.0	54.1
South Africa	ZAF	Pneumococcal vaccine	73.0	66.7
South Africa	ZAF	Rotavirus vaccine	70.0	64.0
South Africa	ZAF	Measles vaccine	60.0	43.9
South Africa	ZAF	Global wasting (<-2 SD) rate	2.6	3.1
South Africa	ZAF	Contraceptive prevalence (CPR)	56.95	46.3
South Sudan	SSD	Safe abortion services	2.2	1.8

South Sudan	SSD	TT - Tetanus toxoid vaccination	68.0	49.7
South Sudan	SSD	IPTp - Intermittent preventive treatment of malaria during pregnancy	10.2	7.5
South Sudan	SSD	Syphilis detection and treatment	10.0	7.3
South Sudan	SSD	Hypertensive disorder case management	4.2	3.1
South Sudan	SSD	Diabetes case management	3.2	2.3
South Sudan	SSD	Malaria case management	13.4	9.8
South Sudan	SSD	MgSO4 management of pre-eclampsia	8.2	6.0
South Sudan	SSD	Thermal protection	11.5	8.8
South Sudan	SSD	Clean cord care	11.1	8.5
South Sudan	SSD	Clean birth environment	9.6	7.4
South Sudan	SSD	Immediate drying and additional stimulation	10.7	8.2
South Sudan	SSD	Neonatal resuscitation	6.4	4.9
South Sudan	SSD	Antibiotics for preterm or prolonged PROM	8.7	6.7
South Sudan	SSD	Parenteral administration of anti-convulsants	8.3	6.4
South Sudan	SSD	Parenteral administration of uterotonics	10.4	8.0
South Sudan	SSD	Parenteral administration of antibiotics	8.7	6.7
South Sudan	SSD	Assisted vaginal delivery	2.9	2.2
South Sudan	SSD	Manual removal of placenta	4.4	3.4
South Sudan	SSD	Removal of retained products of conception	3.9	3.0
South Sudan	SSD	Cesarean delivery	1.0	0.8
South Sudan	SSD	Blood transfusion	1.5	1.2
South Sudan	SSD	Induction of labor for pregnancies lasting 41+ weeks	0.2	0.2
South Sudan	SSD	Complementary feeding - education only	49.4	38.1
South Sudan	SSD	Complementary feeding - supplementary feeding and education	49.4	38.1
South Sudan	SSD	Vitamin A supplementation	51.0	39.4
South Sudan	SSD	Improved sanitation - Utilization of latrines or toilets	11.3	11.3
South Sudan	SSD	Improved water source	40.7	40.7
South Sudan	SSD	Water connection in the home	1.3	1.3
South Sudan	SSD	Hygienic disposal of children's stools	15.7	15.7
South Sudan	SSD	ITN/IRS - Households protected from malaria	38.9	30.0
South Sudan	SSD	Injectable antibiotics for neonatal sepsis	11.7	8.6
South Sudan	SSD	ORS - oral rehydration solution	38.6	29.7
South Sudan	SSD	Zinc for treatment of diarrhea	3.1	2.4
South Sudan	SSD	Oral antibiotics for pneumonia	47.6	36.6
South Sudan	SSD	Vitamin A for treatment of measles	51.0	39.2
South Sudan	SSD	ACTs- Artemisinin compounds for treatment of malaria	2.8	2.2
South Sudan	SSD	SAM - treatment for severe acute malnutrition	46.1	35.5
South Sudan	SSD	BCG vaccine	47.0	34.4
South Sudan	SSD	Polio vaccine	50.0	36.6
South Sudan	SSD	DPT vaccine	49.0	35.8

South Sudan	SSD	H. influenzae type b vaccine	49.0	44.8
South Sudan	SSD	HepB vaccine	49.0	35.8
South Sudan	SSD	Measles vaccine	20.0	14.6
South Sudan	SSD	Global wasting (<-2 SD) rate	22.3	26.7
South Sudan	SSD	Contraceptive prevalence (CPR)	6.7	5.4
Sri Lanka	LKA	Safe abortion services	35.1	28.5
Sri Lanka	LKA	TT - Tetanus toxoid vaccination	99.0	72.4
Sri Lanka	LKA	Syphilis detection and treatment	24.4	17.8
Sri Lanka	LKA	Hypertensive disorder case management	22.2	16.2
Sri Lanka	LKA	Diabetes case management	17.3	12.6
Sri Lanka	LKA	Malaria case management	71.7	52.4
Sri Lanka	LKA	MgSO4 management of pre-eclampsia	44.0	32.2
Sri Lanka	LKA	Thermal protection	98.3	75.6
Sri Lanka	LKA	Clean cord care	94.9	73.0
Sri Lanka	LKA	Clean birth environment	81.6	62.8
Sri Lanka	LKA	Immediate drying and additional stimulation	91.1	70.1
Sri Lanka	LKA	Neonatal resuscitation	54.7	42.1
Sri Lanka	LKA	Antibiotics for preterm or prolonged PROM	74.5	57.3
Sri Lanka	LKA	Parenteral administration of anti-convulsants	71.1	54.7
Sri Lanka	LKA	Parenteral administration of uterotonics	88.9	68.4
Sri Lanka	LKA	Parenteral administration of antibiotics	74.5	57.3
Sri Lanka	LKA	Assisted vaginal delivery	25.2	19.4
Sri Lanka	LKA	Manual removal of placenta	37.2	28.6
Sri Lanka	LKA	Removal of retained products of conception	33.0	25.4
Sri Lanka	LKA	Cesarean delivery	8.7	6.7
Sri Lanka	LKA	Blood transfusion	12.5	9.6
Sri Lanka	LKA	Induction of labor for pregnancies lasting 41+ weeks	1.8	1.4
Sri Lanka	LKA	Complementary feeding - education only	72.6	56.0
Sri Lanka	LKA	Complementary feeding - supplementary feeding and education	72.6	56.0
Sri Lanka	LKA	Vitamin A supplementation	93.0	71.8
Sri Lanka	LKA	Improved sanitation - Utilization of latrines or toilets	95.8	95.8
Sri Lanka	LKA	Improved water source	89.4	89.4
Sri Lanka	LKA	Water connection in the home	34.2	34.2
Sri Lanka	LKA	Hygienic disposal of children's stools	90.6	90.6
Sri Lanka	LKA	ITN/IRS - Households protected from malaria	6.4	4.9
Sri Lanka	LKA	Injectable antibiotics for neonatal sepsis	99.5	72.7
Sri Lanka	LKA	ORS - oral rehydration solution	54.0	41.6
Sri Lanka	LKA	Oral antibiotics for pneumonia	52.3	40.2
Sri Lanka	LKA	Vitamin A for treatment of measles	93.0	71.6
Sri Lanka	LKA	BCG vaccine	99.0	72.4
Sri Lanka	LKA	Polio vaccine	99.0	72.4

Sri Lanka	LKA	DPT vaccine	99.0	72.4
Sri Lanka	LKA	H. influenzae type b vaccine	99.0	90.5
Sri Lanka	LKA	HepB vaccine	99.0	72.4
Sri Lanka	LKA	Measles vaccine	99.0	72.4
Sri Lanka	LKA	Global wasting (<-2 SD) rate	10.6	12.7
Sri Lanka	LKA	Contraceptive prevalence (CPR)	67.55	54.9
Sudan	SDN	Safe abortion services	2.2	1.8
Sudan	SDN	TT - Tetanus toxoid vaccination	80.0	58.5
Sudan	SDN	IPTp - Intermittent preventive treatment of malaria during pregnancy	1.9	1.4
Sudan	SDN	Syphilis detection and treatment	19.6	14.3
Sudan	SDN	Hypertensive disorder case management	12.2	8.9
Sudan	SDN	Diabetes case management	9.5	6.9
Sudan	SDN	Malaria case management	39.3	28.7
Sudan	SDN	MgSO4 management of pre-eclampsia	24.1	17.6
Sudan	SDN	Thermal protection	27.4	21.1
Sudan	SDN	Clean cord care	26.5	20.4
Sudan	SDN	Clean birth environment	22.7	17.5
Sudan	SDN	Immediate drying and additional stimulation	25.4	19.5
Sudan	SDN	Neonatal resuscitation	15.2	11.7
Sudan	SDN	Antibiotics for preterm or prolonged PROM	20.7	15.9
Sudan	SDN	Parenteral administration of anti-convulsants	19.8	15.2
Sudan	SDN	Parenteral administration of uterotonics	24.8	19.1
Sudan	SDN	Parenteral administration of antibiotics	20.7	15.9
Sudan	SDN	Assisted vaginal delivery	7.0	5.4
Sudan	SDN	Manual removal of placenta	10.4	8.0
Sudan	SDN	Removal of retained products of conception	9.2	7.1
Sudan	SDN	Cesarean delivery	2.4	1.8
Sudan	SDN	Blood transfusion	3.5	2.7
Sudan	SDN	Induction of labor for pregnancies lasting 41+ weeks	0.5	0.4
Sudan	SDN	Complementary feeding - education only	28.0	21.6
Sudan	SDN	Complementary feeding - supplementary feeding and education	28.0	21.6
Sudan	SDN	Vitamin A supplementation	20.0	15.4
Sudan	SDN	Improved sanitation - Utilization of latrines or toilets	36.6	36.6
Sudan	SDN	Improved water source	60.3	60.3
Sudan	SDN	Water connection in the home	27.6	27.6
Sudan	SDN	Hand washing with soap	25.9	25.9
Sudan	SDN	Hygienic disposal of children's stools	53.0	53.0
Sudan	SDN	ITN/IRS - Households protected from malaria	41.4	31.9
Sudan	SDN	Injectable antibiotics for neonatal sepsis	27.7	20.2
Sudan	SDN	ORS - oral rehydration solution	19.6	15.1
Sudan	SDN	Antibiotics for treatment of dysentery	40.8	31.4

Sudan	SDN	Zinc for treatment of diarrhea	15.2	11.7
Sudan	SDN	Oral antibiotics for pneumonia	48.3	37.2
Sudan	SDN	Vitamin A for treatment of measles	20.0	15.4
Sudan	SDN	ACTs- Artemisinin compounds for treatment of malaria	14.6	11.2
Sudan	SDN	BCG vaccine	88.0	64.3
Sudan	SDN	Polio vaccine	93.0	68.0
Sudan	SDN	DPT vaccine	93.0	68.0
Sudan	SDN	H. influenzae type b vaccine	93.0	85.0
Sudan	SDN	HepB vaccine	93.0	68.0
Sudan	SDN	Pneumococcal vaccine	93.0	85.0
Sudan	SDN	Rotavirus vaccine	94.0	85.9
Sudan	SDN	Meningococcal A	84.0	61.4
Sudan	SDN	Measles vaccine	90.0	65.8
Sudan	SDN	Global wasting (<-2 SD) rate	16.2	19.5
Sudan	SDN	Contraceptive prevalence (CPR)	15.5	12.6
Suriname	SUR	Safe abortion services	0.2	0.2
Suriname	SUR	TT - Tetanus toxoid vaccination	93.0	68.0
Suriname	SUR	Syphilis detection and treatment	21.0	15.4
Suriname	SUR	Hypertensive disorder case management	16.2	11.8
Suriname	SUR	Diabetes case management	12.6	9.2
Suriname	SUR	Malaria case management	52.3	38.2
Suriname	SUR	MgSO4 management of pre-eclampsia	32.1	23.5
Suriname	SUR	Thermal protection	91.8	70.6
Suriname	SUR	Clean cord care	88.6	68.2
Suriname	SUR	Clean birth environment	76.2	58.6
Suriname	SUR	Immediate drying and additional stimulation	85.1	65.5
Suriname	SUR	Neonatal resuscitation	51.1	39.3
Suriname	SUR	Antibiotics for preterm or prolonged PROM	69.5	53.5
Suriname	SUR	Parenteral administration of anti-convulsants	66.4	51.1
Suriname	SUR	Parenteral administration of uterotonics	83.0	63.9
Suriname	SUR	Parenteral administration of antibiotics	69.5	53.5
Suriname	SUR	Assisted vaginal delivery	23.5	18.1
Suriname	SUR	Manual removal of placenta	34.7	26.7
Suriname	SUR	Removal of retained products of conception	30.8	23.7
Suriname	SUR	Cesarean delivery	8.1	6.2
Suriname	SUR	Blood transfusion	11.7	9.0
Suriname	SUR	Induction of labor for pregnancies lasting 41+ weeks	1.6	1.2
Suriname	SUR	Improved sanitation - Utilization of latrines or toilets	84.5	84.5
Suriname	SUR	Improved water source	95.4	95.4
Suriname	SUR	Water connection in the home	75.1	75.1
Suriname	SUR	Hand washing with soap	75.0	75.0

Suriname	SUR	Hygienic disposal of children's stools	22.0	22.0
Suriname	SUR	ITN/IRS - Households protected from malaria	0.4	0.3
Suriname	SUR	Injectable antibiotics for neonatal sepsis	92.9	67.9
Suriname	SUR	ORS - oral rehydration solution	45.6	35.1
Suriname	SUR	Oral antibiotics for pneumonia	89.1	68.6
Suriname	SUR	Polio vaccine	95.0	69.4
Suriname	SUR	DPT vaccine	95.0	69.4
Suriname	SUR	H. influenzae type b vaccine	95.0	86.8
Suriname	SUR	HepB vaccine	95.0	69.4
Suriname	SUR	Measles vaccine	97.0	70.9
Suriname	SUR	Global wasting (<-2 SD) rate	5.0	6.0
Suriname	SUR	Contraceptive prevalence (CPR)	48.05	39.0
Swaziland	SWZ	Safe abortion services	42.5	34.5
Swaziland	SWZ	TT - Tetanus toxoid vaccination	88.0	64.3
Swaziland	SWZ	IPTp - Intermittent preventive treatment of malaria during pregnancy	0.1	0.1
Swaziland	SWZ	Syphilis detection and treatment	24.4	17.8
Swaziland	SWZ	Iron supplementation in pregnancy	33.6	24.6
Swaziland	SWZ	Hypertensive disorder case management	18.3	13.4
Swaziland	SWZ	Diabetes case management	14.3	10.5
Swaziland	SWZ	Malaria case management	59.0	43.1
Swaziland	SWZ	MgSO4 management of pre-eclampsia	36.2	26.5
Swaziland	SWZ	Thermal protection	86.7	66.7
Swaziland	SWZ	Clean cord care	83.7	64.4
Swaziland	SWZ	Clean birth environment	71.9	55.3
Swaziland	SWZ	Immediate drying and additional stimulation	80.3	61.8
Swaziland	SWZ	Neonatal resuscitation	48.2	37.1
Swaziland	SWZ	Antibiotics for preterm or prolonged PROM	65.6	50.5
Swaziland	SWZ	Parenteral administration of anti-convulsants	62.7	48.2
Swaziland	SWZ	Parenteral administration of uterotonics	78.4	60.3
Swaziland	SWZ	Parenteral administration of antibiotics	65.6	50.5
Swaziland	SWZ	Assisted vaginal delivery	22.2	17.1
Swaziland	SWZ	Manual removal of placenta	32.8	25.2
Swaziland	SWZ	Removal of retained products of conception	29.1	22.4
Swaziland	SWZ	Cesarean delivery	7.6	5.8
Swaziland	SWZ	Blood transfusion	11.0	8.5
Swaziland	SWZ	Induction of labor for pregnancies lasting 41+ weeks	1.5	1.2
Swaziland	SWZ	Complementary feeding - education only	62.4	48.2
Swaziland	SWZ	Complementary feeding - supplementary feeding and education	62.4	48.2
Swaziland	SWZ	Vitamin A supplementation	33.0	25.5
Swaziland	SWZ	Improved sanitation - Utilization of latrines or toilets	58.4	58.4
Swaziland	SWZ	Improved water source	69.0	69.0

Swaziland	SWZ	Water connection in the home	42.1	42.1
Swaziland	SWZ	Hand washing with soap	26.7	26.7
Swaziland	SWZ	Hygienic disposal of children's stools	55.3	55.3
Swaziland	SWZ	ITN/IRS - Households protected from malaria	16.2	12.5
Swaziland	SWZ	Injectable antibiotics for neonatal sepsis	87.7	64.1
Swaziland	SWZ	ORS - oral rehydration solution	84.0	64.6
Swaziland	SWZ	Antibiotics for treatment of dysentery	22.3	17.2
Swaziland	SWZ	Zinc for treatment of diarrhea	45.2	34.8
Swaziland	SWZ	Oral antibiotics for pneumonia	59.6	45.9
Swaziland	SWZ	Vitamin A for treatment of measles	33.0	25.4
Swaziland	SWZ	ACTs- Artemisinin compounds for treatment of malaria	0.1	0.1
Swaziland	SWZ	BCG vaccine	98.0	71.6
Swaziland	SWZ	Polio vaccine	90.0	65.8
Swaziland	SWZ	DPT vaccine	90.0	65.8
Swaziland	SWZ	H. influenzae type b vaccine	90.0	82.3
Swaziland	SWZ	HepB vaccine	90.0	65.8
Swaziland	SWZ	Pneumococcal vaccine	88.0	80.4
Swaziland	SWZ	Rotavirus vaccine	86.0	78.6
Swaziland	SWZ	Measles vaccine	89.0	65.1
Swaziland	SWZ	Global wasting (<-2 SD) rate	2.1	2.5
Swaziland	SWZ	Contraceptive prevalence (CPR)	67.9	55.1
Syrian Arab Republic	SYR	Safe abortion services	40.0	32.5
Syrian Arab Republic	SYR	TT - Tetanus toxoid vaccination	91.0	66.5
Syrian Arab Republic	SYR	Syphilis detection and treatment	15.7	11.5
Syrian Arab Republic	SYR	Hypertensive disorder case management	15.3	11.2
Syrian Arab Republic	SYR	Diabetes case management	11.9	8.7
Syrian Arab Republic	SYR	Malaria case management	49.4	36.1
Syrian Arab Republic	SYR	MgSO4 management of pre-eclampsia	30.3	22.2
Syrian Arab Republic	SYR	Thermal protection	69.6	53.6
Syrian Arab Republic	SYR	Clean cord care	67.2	51.7
Syrian Arab Republic	SYR	Clean birth environment	57.7	44.4
Syrian Arab Republic	SYR	Immediate drying and additional stimulation	64.5	49.6
Syrian Arab Republic	SYR	Neonatal resuscitation	38.7	29.8
Syrian Arab Republic	SYR	Antibiotics for preterm or prolonged PROM	52.7	40.6
Syrian Arab Republic	SYR	Parenteral administration of anti-convulsants	50.3	38.7
Syrian Arab Republic	SYR	Parenteral administration of uterotonics	62.9	48.4
Syrian Arab Republic	SYR	Parenteral administration of antibiotics	52.7	40.6
Syrian Arab Republic	SYR	Assisted vaginal delivery	17.8	13.7
Syrian Arab Republic	SYR	Manual removal of placenta	26.3	20.2
Syrian Arab Republic	SYR	Removal of retained products of conception	23.3	17.9
Syrian Arab Republic	SYR	Cesarean delivery	6.1	4.7
Syrian Arab Republic	SYR	Blood transfusion	8.9	6.8

Syrian Arab Republic	SYR	Induction of labor for pregnancies lasting 41+ weeks	1.2	0.9
Syrian Arab Republic	SYR	Improved sanitation - Utilization of latrines or toilets	91.2	91.2
Syrian Arab Republic	SYR	Improved water source	97.2	97.2
Syrian Arab Republic	SYR	Water connection in the home	70.9	70.9
Syrian Arab Republic	SYR	Injectable antibiotics for neonatal sepsis	70.4	51.5
Syrian Arab Republic	SYR	ORS - oral rehydration solution	49.8	38.3
Syrian Arab Republic	SYR	Oral antibiotics for pneumonia	76.8	59.1
Syrian Arab Republic	SYR	BCG vaccine	79.0	57.8
Syrian Arab Republic	SYR	Polio vaccine	53.0	38.7
Syrian Arab Republic	SYR	DPT vaccine	47.0	34.4
Syrian Arab Republic	SYR	H. influenzae type b vaccine	48.0	43.9
Syrian Arab Republic	SYR	HepB vaccine	47.0	34.4
Syrian Arab Republic	SYR	Measles vaccine	67.0	49.0
Syrian Arab Republic	SYR	Global wasting (<-2 SD) rate	10.3	12.4
Syrian Arab Republic	SYR	Contraceptive prevalence (CPR)	59.75	48.5
Tajikistan	TJK	Safe abortion services	35.1	28.5
Tajikistan	TJK	Syphilis detection and treatment	23.0	16.8
Tajikistan	TJK	Iron supplementation in pregnancy	2.2	1.6
Tajikistan	TJK	Hypertensive disorder case management	15.5	11.3
Tajikistan	TJK	Diabetes case management	12.1	8.8
Tajikistan	TJK	Malaria case management	50.1	36.6
Tajikistan	TJK	MgSO4 management of pre-eclampsia	30.7	22.4
Tajikistan	TJK	Thermal protection	87.1	67.0
Tajikistan	TJK	Clean cord care	84.1	64.7
Tajikistan	TJK	Clean birth environment	72.3	55.6
Tajikistan	TJK	Immediate drying and additional stimulation	80.7	62.1
Tajikistan	TJK	Neonatal resuscitation	48.5	37.3
Tajikistan	TJK	Antibiotics for preterm or prolonged PROM	66.0	50.8
Tajikistan	TJK	Parenteral administration of anti-convulsants	63.0	48.5
Tajikistan	TJK	Parenteral administration of uterotonics	78.8	60.6
Tajikistan	TJK	Parenteral administration of antibiotics	66.0	50.8
Tajikistan	TJK	Assisted vaginal delivery	22.3	17.2
Tajikistan	TJK	Manual removal of placenta	33.0	25.4
Tajikistan	TJK	Removal of retained products of conception	29.2	22.5
Tajikistan	TJK	Cesarean delivery	7.7	5.9
Tajikistan	TJK	Blood transfusion	11.1	8.5
Tajikistan	TJK	Induction of labor for pregnancies lasting 41+ weeks	1.6	1.2
Tajikistan	TJK	Complementary feeding - education only	29.1	22.5
Tajikistan	TJK	Complementary feeding - supplementary feeding and education	29.1	22.5
Tajikistan	TJK	Vitamin A supplementation	91.0	70.2

Tajikistan	TJK	Improved sanitation - Utilization of latrines or toilets	97.0	97.0
Tajikistan	TJK	Improved water source	81.2	81.2
Tajikistan	TJK	Water connection in the home	52.2	52.2
Tajikistan	TJK	Hygienic disposal of children's stools	52.0	52.0
Tajikistan	TJK	ITN/IRS - Households protected from malaria	2.0	1.5
Tajikistan	TJK	Injectable antibiotics for neonatal sepsis	88.2	64.5
Tajikistan	TJK	ORS - oral rehydration solution	61.7	47.5
Tajikistan	TJK	Antibiotics for treatment of dysentery	59.1	45.5
Tajikistan	TJK	Zinc for treatment of diarrhea	19.7	15.2
Tajikistan	TJK	Oral antibiotics for pneumonia	68.5	52.7
Tajikistan	TJK	Vitamin A for treatment of measles	91.0	70.0
Tajikistan	TJK	ACTs- Artemisinin compounds for treatment of malaria	0.1	0.1
Tajikistan	TJK	BCG vaccine	99.0	72.4
Tajikistan	TJK	Polio vaccine	96.0	70.2
Tajikistan	TJK	DPT vaccine	96.0	70.2
Tajikistan	TJK	H. influenzae type b vaccine	96.0	87.7
Tajikistan	TJK	HepB vaccine	96.0	70.2
Tajikistan	TJK	Rotavirus vaccine	96.0	87.7
Tajikistan	TJK	Measles vaccine	98.0	71.6
Tajikistan	TJK	Global wasting (<-2 SD) rate	5.5	6.6
Tajikistan	TJK	Contraceptive prevalence (CPR)	31.5	25.6
Thailand	THA	Safe abortion services	39.0	31.7
Thailand	THA	TT - Tetanus toxoid vaccination	98.0	71.6
Thailand	THA	Syphilis detection and treatment	24.3	17.8
Thailand	THA	Hypertensive disorder case management	21.8	15.9
Thailand	THA	Diabetes case management	17.0	12.4
Thailand	THA	Malaria case management	70.4	51.5
Thailand	THA	MgSO4 management of pre-eclampsia	43.2	31.6
Thailand	THA	Thermal protection	97.0	74.6
Thailand	THA	Clean cord care	93.6	72.0
Thailand	THA	Clean birth environment	80.5	61.9
Thailand	THA	Immediate drying and additional stimulation	89.8	69.1
Thailand	THA	Neonatal resuscitation	54.0	41.6
Thailand	THA	Antibiotics for preterm or prolonged PROM	73.4	56.5
Thailand	THA	Parenteral administration of anti-convulsants	70.2	54.0
Thailand	THA	Parenteral administration of uterotonics	87.7	67.5
Thailand	THA	Parenteral administration of antibiotics	73.4	56.5
Thailand	THA	Assisted vaginal delivery	24.8	19.1
Thailand	THA	Manual removal of placenta	36.7	28.2
Thailand	THA	Removal of retained products of conception	32.5	25.0
Thailand	THA	Cesarean delivery	8.5	6.5
Thailand	THA	Blood transfusion	12.4	9.5

Thailand	THA	Induction of labor for pregnancies lasting 41+ weeks	1.7	1.3
Thailand	THA	Complementary feeding - education only	75.0	57.9
Thailand	THA	Complementary feeding - supplementary feeding and education	75.0	57.9
Thailand	THA	Improved sanitation - Utilization of latrines or toilets	98.8	98.8
Thailand	THA	Improved water source	99.0	99.0
Thailand	THA	Water connection in the home	69.6	69.6
Thailand	THA	Hand washing with soap	81.2	81.2
Thailand	THA	Hygienic disposal of children's stools	42.0	42.0
Thailand	THA	Injectable antibiotics for neonatal sepsis	98.1	71.7
Thailand	THA	ORS - oral rehydration solution	72.5	55.8
Thailand	THA	Oral antibiotics for pneumonia	79.5	61.2
Thailand	THA	BCG vaccine	99.0	72.4
Thailand	THA	Polio vaccine	97.0	70.9
Thailand	THA	DPT vaccine	97.0	70.9
Thailand	THA	HepB vaccine	97.0	70.9
Thailand	THA	Measles vaccine	99.0	72.4
Thailand	THA	Global wasting (<-2 SD) rate	5.5	6.6
Thailand	THA	Contraceptive prevalence (CPR)	79.3	64.4
Timor-Leste	TLS	Safe abortion services	39.0	31.7
Timor-Leste	TLS	TT - Tetanus toxoid vaccination	83.0	60.7
Timor-Leste	TLS	Syphilis detection and treatment	20.9	15.3
Timor-Leste	TLS	Iron supplementation in pregnancy	12.9	9.4
Timor-Leste	TLS	Hypertensive disorder case management	18.5	13.5
Timor-Leste	TLS	Diabetes case management	14.4	10.5
Timor-Leste	TLS	Malaria case management	59.6	43.6
Timor-Leste	TLS	MgSO4 management of pre-eclampsia	36.5	26.7
Timor-Leste	TLS	Thermal protection	48.0	36.9
Timor-Leste	TLS	Clean cord care	46.3	35.6
Timor-Leste	TLS	Clean birth environment	39.8	30.6
Timor-Leste	TLS	Immediate drying and additional stimulation	44.4	34.2
Timor-Leste	TLS	Neonatal resuscitation	26.7	20.5
Timor-Leste	TLS	Antibiotics for preterm or prolonged PROM	36.3	27.9
Timor-Leste	TLS	Parenteral administration of anti-convulsants	34.7	26.7
Timor-Leste	TLS	Parenteral administration of uterotonics	43.4	33.4
Timor-Leste	TLS	Parenteral administration of antibiotics	36.3	27.9
Timor-Leste	TLS	Assisted vaginal delivery	12.3	9.5
Timor-Leste	TLS	Manual removal of placenta	18.1	13.9
Timor-Leste	TLS	Removal of retained products of conception	16.1	12.4
Timor-Leste	TLS	Cesarean delivery	4.2	3.2
Timor-Leste	TLS	Blood transfusion	6.1	4.7
Timor-Leste	TLS	Induction of labor for pregnancies lasting 41+ weeks	0.9	0.7

Timor-Leste	TLS	Complementary feeding - education only	33.6	25.9
Timor-Leste	TLS	Complementary feeding - supplementary feeding and education	33.6	25.9
Timor-Leste	TLS	Vitamin A supplementation	66.0	50.9
Timor-Leste	TLS	Improved sanitation - Utilization of latrines or toilets	53.5	53.5
Timor-Leste	TLS	Improved water source	78.3	78.3
Timor-Leste	TLS	Water connection in the home	50.6	50.6
Timor-Leste	TLS	Hygienic disposal of children's stools	25.5	25.5
Timor-Leste	TLS	ITN/IRS - Households protected from malaria	64.0	49.4
Timor-Leste	TLS	Injectable antibiotics for neonatal sepsis	48.5	35.5
Timor-Leste	TLS	ORS - oral rehydration solution	69.9	53.8
Timor-Leste	TLS	Antibiotics for treatment of dysentery	6.7	5.2
Timor-Leste	TLS	Zinc for treatment of diarrhea	50.2	38.6
Timor-Leste	TLS	Oral antibiotics for pneumonia	70.3	54.1
Timor-Leste	TLS	Vitamin A for treatment of measles	66.0	50.8
Timor-Leste	TLS	ACTs- Artemisinin compounds for treatment of malaria	1.1	0.8
Timor-Leste	TLS	SAM - treatment for severe acute malnutrition	5.6	4.3
Timor-Leste	TLS	BCG vaccine	95.0	69.4
Timor-Leste	TLS	Polio vaccine	83.0	60.7
Timor-Leste	TLS	DPT vaccine	83.0	60.7
Timor-Leste	TLS	H. influenzae type b vaccine	83.0	75.9
Timor-Leste	TLS	HepB vaccine	83.0	60.7
Timor-Leste	TLS	Measles vaccine	70.0	51.2
Timor-Leste	TLS	Global wasting (<-2 SD) rate	24.2	29.1
Timor-Leste	TLS	Contraceptive prevalence (CPR)	30.5	24.8
Togo	TGO	TT - Tetanus toxoid vaccination	83.0	60.7
Togo	TGO	IPTp - Intermittent preventive treatment of malaria during pregnancy	68.2	49.9
Togo	TGO	Syphilis detection and treatment	18.4	13.5
Togo	TGO	Iron supplementation in pregnancy	37.1	27.1
Togo	TGO	Hypertensive disorder case management	16.7	12.2
Togo	TGO	Diabetes case management	18.3	13.4
Togo	TGO	Malaria case management	49.1	35.9
Togo	TGO	MgSO4 management of pre-eclampsia	9.5	6.9
Togo	TGO	Thermal protection	60.7	46.7
Togo	TGO	Clean cord care	46.4	35.7
Togo	TGO	Clean birth environment	42.8	32.9
Togo	TGO	Immediate drying and additional stimulation	55.4	42.6
Togo	TGO	Neonatal resuscitation	12.8	9.8
Togo	TGO	Antibiotics for preterm or prolonged PROM	48.5	37.3
Togo	TGO	Parenteral administration of anti-convulsants	10.9	8.4
Togo	TGO	Parenteral administration of uterotonics	55.4	42.6
Togo	TGO	Parenteral administration of antibiotics	48.5	37.3

Togo	TGO	Assisted vaginal delivery	8.1	6.2
Togo	TGO	Manual removal of placenta	36.1	27.8
Togo	TGO	Removal of retained products of conception	16.9	13.0
Togo	TGO	Cesarean delivery	0.8	0.6
Togo	TGO	Blood transfusion	7.7	5.9
Togo	TGO	Induction of labor for pregnancies lasting 41+ weeks	1.1	0.8
Togo	TGO	Complementary feeding - education only	20.3	15.7
Togo	TGO	Complementary feeding - supplementary feeding and education	20.3	15.7
Togo	TGO	Vitamin A supplementation	87.0	67.1
Togo	TGO	Improved sanitation - Utilization of latrines or toilets	16.1	16.1
Togo	TGO	Improved water source	65.1	65.1
Togo	TGO	Water connection in the home	16.7	16.7
Togo	TGO	Hand washing with soap	62.0	62.0
Togo	TGO	Hygienic disposal of children's stools	34.8	34.8
Togo	TGO	ITN/IRS - Households protected from malaria	65.4	50.5
Togo	TGO	Injectable antibiotics for neonatal sepsis	61.4	44.9
Togo	TGO	ORS - oral rehydration solution	18.5	14.2
Togo	TGO	Antibiotics for treatment of dysentery	29.7	22.9
Togo	TGO	Zinc for treatment of diarrhea	0.1	0.1
Togo	TGO	Oral antibiotics for pneumonia	48.5	37.3
Togo	TGO	Vitamin A for treatment of measles	87.0	66.9
Togo	TGO	ACTs- Artemisinin compounds for treatment of malaria	6.3	4.8
Togo	TGO	SAM - treatment for severe acute malnutrition	6.4	4.9
Togo	TGO	BCG vaccine	83.0	60.7
Togo	TGO	Polio vaccine	66.0	48.2
Togo	TGO	DPT vaccine	88.0	64.3
Togo	TGO	H. influenzae type b vaccine	88.0	80.4
Togo	TGO	HepB vaccine	88.0	64.3
Togo	TGO	Pneumococcal vaccine	88.0	80.4
Togo	TGO	Rotavirus vaccine	89.0	81.3
Togo	TGO	Measles vaccine	91.0	66.5
Togo	TGO	Global wasting (<-2 SD) rate	6.6	7.9
Togo	TGO	Contraceptive prevalence (CPR)	24.65	20.0
Tonga	TON	Safe abortion services	85.0	69.0
Tonga	TON	TT - Tetanus toxoid vaccination	71.2	52.0
Tonga	TON	Syphilis detection and treatment	19.3	14.1
Tonga	TON	Hypertensive disorder case management	16.9	12.4
Tonga	TON	Diabetes case management	13.2	9.6
Tonga	TON	Malaria case management	54.6	39.9
Tonga	TON	MgSO4 management of pre-eclampsia	33.5	24.5
Tonga	TON	Thermal protection	96.8	74.5

Tonga	TON	Clean cord care	93.4	71.9
Tonga	TON	Clean birth environment	80.3	61.8
Tonga	TON	Immediate drying and additional stimulation	89.6	68.9
Tonga	TON	Neonatal resuscitation	53.8	41.4
Tonga	TON	Antibiotics for preterm or prolonged PROM	73.3	56.4
Tonga	TON	Parenteral administration of anti-convulsants	70.0	53.9
Tonga	TON	Parenteral administration of uterotonics	87.5	67.3
Tonga	TON	Parenteral administration of antibiotics	73.3	56.4
Tonga	TON	Assisted vaginal delivery	24.7	19.0
Tonga	TON	Manual removal of placenta	36.6	28.2
Tonga	TON	Removal of retained products of conception	32.4	24.9
Tonga	TON	Cesarean delivery	8.5	6.5
Tonga	TON	Blood transfusion	12.3	9.5
Tonga	TON	Induction of labor for pregnancies lasting 41+ weeks	1.7	1.3
Tonga	TON	Improved sanitation - Utilization of latrines or toilets	93.4	93.4
Tonga	TON	Improved water source	99.0	99.0
Tonga	TON	Water connection in the home	78.2	78.2
Tonga	TON	Hygienic disposal of children's stools	40.4	40.4
Tonga	TON	Injectable antibiotics for neonatal sepsis	97.9	71.6
Tonga	TON	BCG vaccine	88.0	64.3
Tonga	TON	Polio vaccine	83.0	60.7
Tonga	TON	DPT vaccine	81.0	59.2
Tonga	TON	H. influenzae type b vaccine	81.0	74.0
Tonga	TON	HepB vaccine	81.0	59.2
Tonga	TON	Measles vaccine	85.0	62.1
Tonga	TON	Global wasting (<-2 SD) rate	10.6	12.7
Tonga	TON	Contraceptive prevalence (CPR)	37.55	30.5
Tunisia	TUN	Safe abortion services	2.2	1.8
Tunisia	TUN	TT - Tetanus toxoid vaccination	96.0	70.2
Tunisia	TUN	Syphilis detection and treatment	23.6	17.3
Tunisia	TUN	Hypertensive disorder case management	20.2	14.8
Tunisia	TUN	Diabetes case management	15.8	11.6
Tunisia	TUN	Malaria case management	65.2	47.7
Tunisia	TUN	MgSO4 management of pre-eclampsia	40.0	29.2
Tunisia	TUN	Thermal protection	98.5	75.8
Tunisia	TUN	Clean cord care	95.1	73.2
Tunisia	TUN	Clean birth environment	81.8	62.9
Tunisia	TUN	Immediate drying and additional stimulation	91.3	70.3
Tunisia	TUN	Neonatal resuscitation	54.8	42.2
Tunisia	TUN	Antibiotics for preterm or prolonged PROM	74.6	57.4
Tunisia	TUN	Parenteral administration of anti-convulsants	71.3	54.9
Tunisia	TUN	Parenteral administration of uterotonics	89.1	68.6

Tunisia	TUN	Parenteral administration of antibiotics	74.6	57.4
Tunisia	TUN	Assisted vaginal delivery	25.2	19.4
Tunisia	TUN	Manual removal of placenta	37.3	28.7
Tunisia	TUN	Removal of retained products of conception	33.0	25.4
Tunisia	TUN	Cesarean delivery	8.7	6.7
Tunisia	TUN	Blood transfusion	12.6	9.7
Tunisia	TUN	Induction of labor for pregnancies lasting 41+ weeks	1.8	1.4
Tunisia	TUN	Complementary feeding - education only	69.5	53.6
Tunisia	TUN	Complementary feeding - supplementary feeding and education	69.5	53.6
Tunisia	TUN	Improved sanitation - Utilization of latrines or toilets	90.9	90.9
Tunisia	TUN	Improved water source	96.3	96.3
Tunisia	TUN	Water connection in the home	88.9	88.9
Tunisia	TUN	Hand washing with soap	94.2	94.2
Tunisia	TUN	Hygienic disposal of children's stools	13.8	13.8
Tunisia	TUN	Injectable antibiotics for neonatal sepsis	99.7	72.9
Tunisia	TUN	ORS - oral rehydration solution	39.6	30.5
Tunisia	TUN	Zinc for treatment of diarrhea	3.9	3.0
Tunisia	TUN	Oral antibiotics for pneumonia	77.0	59.3
Tunisia	TUN	BCG vaccine	92.0	67.3
Tunisia	TUN	Polio vaccine	97.0	70.9
Tunisia	TUN	DPT vaccine	97.0	70.9
Tunisia	TUN	H. influenzae type b vaccine	97.0	88.7
Tunisia	TUN	HepB vaccine	97.0	70.9
Tunisia	TUN	Measles vaccine	98.0	71.6
Tunisia	TUN	Global wasting (<-2 SD) rate	2.8	3.3
Tunisia	TUN	Contraceptive prevalence (CPR)	59.25	48.1
Turkey	TUR	Safe abortion services	40.0	32.5
Turkey	TUR	TT - Tetanus toxoid vaccination	95.0	69.4
Turkey	TUR	Syphilis detection and treatment	23.9	17.5
Turkey	TUR	Hypertensive disorder case management	21.4	15.6
Turkey	TUR	Diabetes case management	16.7	12.2
Turkey	TUR	Malaria case management	68.9	50.4
Turkey	TUR	MgSO4 management of pre-eclampsia	42.3	30.9
Turkey	TUR	Thermal protection	96.1	73.9
Turkey	TUR	Clean cord care	92.8	71.4
Turkey	TUR	Clean birth environment	79.7	61.3
Turkey	TUR	Immediate drying and additional stimulation	89.0	68.5
Turkey	TUR	Neonatal resuscitation	53.4	41.1
Turkey	TUR	Antibiotics for preterm or prolonged PROM	72.7	55.9
Turkey	TUR	Parenteral administration of anti-convulsants	69.5	53.5
Turkey	TUR	Parenteral administration of uterotonics	86.9	66.9

Turkey	TUR	Parenteral administration of antibiotics	72.7	55.9
Turkey	TUR	Assisted vaginal delivery	24.6	18.9
Turkey	TUR	Manual removal of placenta	36.4	28.0
Turkey	TUR	Removal of retained products of conception	32.2	24.8
Turkey	TUR	Cesarean delivery	8.5	6.5
Turkey	TUR	Blood transfusion	12.2	9.4
Turkey	TUR	Induction of labor for pregnancies lasting 41+ weeks	1.7	1.3
Turkey	TUR	Improved sanitation - Utilization of latrines or toilets	97.3	97.3
Turkey	TUR	Improved water source	98.9	98.9
Turkey	TUR	Water connection in the home	97.8	97.8
Turkey	TUR	Injectable antibiotics for neonatal sepsis	97.2	71.1
Turkey	TUR	ORS - oral rehydration solution	20.4	15.7
Turkey	TUR	Antibiotics for treatment of dysentery	7.5	5.8
Turkey	TUR	BCG vaccine	96.0	70.2
Turkey	TUR	Polio vaccine	98.0	71.6
Turkey	TUR	DPT vaccine	98.0	71.6
Turkey	TUR	H. influenzae type b vaccine	98.0	89.6
Turkey	TUR	HepB vaccine	98.0	71.6
Turkey	TUR	Pneumococcal vaccine	97.0	88.7
Turkey	TUR	Measles vaccine	96.0	70.2
Turkey	TUR	Global wasting (<-2 SD) rate	1.1	1.3
Turkey	TUR	Contraceptive prevalence (CPR)	74.45	60.5
Turkmenistan	TKM	Safe abortion services	35.1	28.5
Turkmenistan	TKM	Syphilis detection and treatment	24.7	18.1
Turkmenistan	TKM	Hypertensive disorder case management	23.2	17.0
Turkmenistan	TKM	Diabetes case management	18.1	13.2
Turkmenistan	TKM	Malaria case management	74.7	54.6
Turkmenistan	TKM	MgSO4 management of pre-eclampsia	45.8	33.5
Turkmenistan	TKM	Thermal protection	98.3	75.6
Turkmenistan	TKM	Clean cord care	94.9	73.0
Turkmenistan	TKM	Clean birth environment	81.6	62.8
Turkmenistan	TKM	Immediate drying and additional stimulation	91.1	70.1
Turkmenistan	TKM	Neonatal resuscitation	54.7	42.1
Turkmenistan	TKM	Antibiotics for preterm or prolonged PROM	74.4	57.3
Turkmenistan	TKM	Parenteral administration of anti-convulsants	71.1	54.7
Turkmenistan	TKM	Parenteral administration of uterotonics	88.9	68.4
Turkmenistan	TKM	Parenteral administration of antibiotics	74.4	57.3
Turkmenistan	TKM	Assisted vaginal delivery	25.1	19.3
Turkmenistan	TKM	Manual removal of placenta	37.2	28.6
Turkmenistan	TKM	Removal of retained products of conception	33.0	25.4
Turkmenistan	TKM	Cesarean delivery	8.7	6.7
Turkmenistan	TKM	Blood transfusion	12.5	9.6

Turkmenistan	TKM	Induction of labor for pregnancies lasting 41+ weeks	1.8	1.4
Turkmenistan	TKM	Complementary feeding - education only	85.2	65.7
Turkmenistan	TKM	Complementary feeding - supplementary feeding and education	85.2	65.7
Turkmenistan	TKM	Improved sanitation - Utilization of latrines or toilets	98.7	98.7
Turkmenistan	TKM	Improved water source	98.8	98.8
Turkmenistan	TKM	Water connection in the home	56.3	56.3
Turkmenistan	TKM	Hand washing with soap	99.3	99.3
Turkmenistan	TKM	Hygienic disposal of children's stools	60.5	60.5
Turkmenistan	TKM	Injectable antibiotics for neonatal sepsis	99.5	72.7
Turkmenistan	TKM	ORS - oral rehydration solution	47.1	36.2
Turkmenistan	TKM	Zinc for treatment of diarrhea	10.9	8.4
Turkmenistan	TKM	Oral antibiotics for pneumonia	82.7	63.6
Turkmenistan	TKM	BCG vaccine	98.0	71.6
Turkmenistan	TKM	Polio vaccine	99.0	72.4
Turkmenistan	TKM	DPT vaccine	99.0	72.4
Turkmenistan	TKM	H. influenzae type b vaccine	99.0	90.5
Turkmenistan	TKM	HepB vaccine	99.0	72.4
Turkmenistan	TKM	Measles vaccine	99.0	72.4
Turkmenistan	TKM	Global wasting (<-2 SD) rate	4.3	5.1
Turkmenistan	TKM	Contraceptive prevalence (CPR)	53.65	43.6
Uganda	UGA	Safe abortion services	3.3	2.7
Uganda	UGA	TT - Tetanus toxoid vaccination	85.0	62.1
Uganda	UGA	IPTp - Intermittent preventive treatment of malaria during pregnancy	45.9	33.6
Uganda	UGA	Syphilis detection and treatment	28.1	20.5
Uganda	UGA	Iron supplementation in pregnancy	22.6	16.5
Uganda	UGA	Hypertensive disorder case management	17.7	12.9
Uganda	UGA	Diabetes case management	10.1	7.4
Uganda	UGA	Malaria case management	40.6	29.7
Uganda	UGA	MgSO4 management of pre-eclampsia	23.1	16.9
Uganda	UGA	Thermal protection	72.5	55.8
Uganda	UGA	Clean cord care	63.6	48.9
Uganda	UGA	Clean birth environment	57.7	44.4
Uganda	UGA	Immediate drying and additional stimulation	56.8	43.7
Uganda	UGA	Neonatal resuscitation	36.2	27.9
Uganda	UGA	Antibiotics for preterm or prolonged PROM	50.0	38.5
Uganda	UGA	Parenteral administration of anti-convulsants	40.9	31.5
Uganda	UGA	Parenteral administration of uterotonics	65.6	50.5
Uganda	UGA	Parenteral administration of antibiotics	50.0	38.5
Uganda	UGA	Assisted vaginal delivery	25.6	19.7
Uganda	UGA	Manual removal of placenta	24.2	18.6
Uganda	UGA	Removal of retained products of conception	28.4	21.9

Uganda	UGA	Cesarean delivery	2.3	1.8
Uganda	UGA	Blood transfusion	25.3	19.5
Uganda	UGA	Induction of labor for pregnancies lasting 41+ weeks	11.5	8.8
Uganda	UGA	Complementary feeding - education only	30.2	23.3
Uganda	UGA	Complementary feeding - supplementary feeding and education	30.2	23.3
Uganda	UGA	Vitamin A supplementation	27.0	20.8
Uganda	UGA	Improved sanitation - Utilization of latrines or toilets	18.5	18.5
Uganda	UGA	Improved water source	49.1	49.1
Uganda	UGA	Water connection in the home	10.4	10.4
Uganda	UGA	Hand washing with soap	27.4	27.4
Uganda	UGA	Hygienic disposal of children's stools	76.3	76.3
Uganda	UGA	ITN/IRS - Households protected from malaria	80.8	62.3
Uganda	UGA	Injectable antibiotics for neonatal sepsis	73.4	53.7
Uganda	UGA	ORS - oral rehydration solution	46.7	35.9
Uganda	UGA	Antibiotics for treatment of dysentery	32.1	24.7
Uganda	UGA	Zinc for treatment of diarrhea	40.3	31.0
Uganda	UGA	Oral antibiotics for pneumonia	71.3	54.9
Uganda	UGA	Vitamin A for treatment of measles	27.0	20.8
Uganda	UGA	ACTs- Artemisinin compounds for treatment of malaria	45.9	35.3
Uganda	UGA	SAM - treatment for severe acute malnutrition	2.8	2.2
Uganda	UGA	BCG vaccine	88.0	64.3
Uganda	UGA	Polio vaccine	88.0	64.3
Uganda	UGA	DPT vaccine	93.0	68.0
Uganda	UGA	H. influenzae type b vaccine	93.0	85.0
Uganda	UGA	HepB vaccine	93.0	68.0
Uganda	UGA	Pneumococcal vaccine	92.0	84.1
Uganda	UGA	Rotavirus vaccine	36.0	32.9
Uganda	UGA	Measles vaccine	80.0	58.5
Uganda	UGA	Global wasting (<-2 SD) rate	3.6	4.3
Uganda	UGA	Contraceptive prevalence (CPR)	43.55	35.4
Ukraine	UKR	Safe abortion services	87.5	71.1
Ukraine	UKR	IPTp - Intermittent preventive treatment of malaria during pregnancy	41.1	30.0
Ukraine	UKR	Syphilis detection and treatment	24.4	17.8
Ukraine	UKR	Iron supplementation in pregnancy	3.4	2.5
Ukraine	UKR	Hypertensive disorder case management	21.0	15.4
Ukraine	UKR	Diabetes case management	16.3	11.9
Ukraine	UKR	Malaria case management	67.6	49.4
Ukraine	UKR	MgSO4 management of pre-eclampsia	41.5	30.3
Ukraine	UKR	Thermal protection	97.7	75.2
Ukraine	UKR	Clean cord care	94.4	72.6

Ukraine	UKR	Clean birth environment	81.1	62.4
Ukraine	UKR	Immediate drying and additional stimulation	90.5	69.6
Ukraine	UKR	Neonatal resuscitation	54.4	41.9
Ukraine	UKR	Antibiotics for preterm or prolonged PROM	74.0	56.9
Ukraine	UKR	Parenteral administration of anti-convulsants	70.7	54.4
Ukraine	UKR	Parenteral administration of uterotonics	88.4	68.0
Ukraine	UKR	Parenteral administration of antibiotics	74.0	56.9
Ukraine	UKR	Assisted vaginal delivery	25.0	19.2
Ukraine	UKR	Manual removal of placenta	37.0	28.5
Ukraine	UKR	Removal of retained products of conception	32.8	25.2
Ukraine	UKR	Cesarean delivery	8.6	6.6
Ukraine	UKR	Blood transfusion	12.5	9.6
Ukraine	UKR	Induction of labor for pregnancies lasting 41+ weeks	1.7	1.3
Ukraine	UKR	Complementary feeding - education only	58.5	45.1
Ukraine	UKR	Complementary feeding - supplementary feeding and education	58.5	45.1
Ukraine	UKR	Improved sanitation - Utilization of latrines or toilets	96.2	96.2
Ukraine	UKR	Improved water source	93.8	93.8
Ukraine	UKR	Water connection in the home	62.0	62.0
Ukraine	UKR	Injectable antibiotics for neonatal sepsis	98.9	72.3
Ukraine	UKR	ORS - oral rehydration solution	59.2	45.6
Ukraine	UKR	Oral antibiotics for pneumonia	92.3	71.0
Ukraine	UKR	BCG vaccine	90.0	65.8
Ukraine	UKR	Polio vaccine	48.0	35.1
Ukraine	UKR	DPT vaccine	50.0	36.6
Ukraine	UKR	H. influenzae type b vaccine	39.0	35.6
Ukraine	UKR	HepB vaccine	52.0	38.0
Ukraine	UKR	Measles vaccine	86.0	62.9
Ukraine	UKR	Global wasting (<-2 SD) rate	4.5	5.4
Ukraine	UKR	Contraceptive prevalence (CPR)	67.4	54.7
United Republic of Tanzania	TZA	Safe abortion services	3.3	2.7
United Republic of Tanzania	TZA	TT - Tetanus toxoid vaccination	90.0	65.8
United Republic of Tanzania	TZA	IPTp - Intermittent preventive treatment of malaria during pregnancy	56.1	41.0
United Republic of Tanzania	TZA	Syphilis detection and treatment	56.2	41.1
United Republic of Tanzania	TZA	Iron supplementation in pregnancy	21.4	15.6
United Republic of Tanzania	TZA	Hypertensive disorder case management	11.8	8.6
United Republic of Tanzania	TZA	Diabetes case management	9.2	6.7
United Republic of Tanzania	TZA	Malaria case management	42.3	30.9

United Republic of Tanzania	TZA	MgSO4 management of pre-eclampsia	34.8	25.4
United Republic of Tanzania	TZA	Thermal protection	61.7	47.5
United Republic of Tanzania	TZA	Clean cord care	60.8	46.8
United Republic of Tanzania	TZA	Clean birth environment	51.6	39.7
United Republic of Tanzania	TZA	Immediate drying and additional stimulation	57.4	44.2
United Republic of Tanzania	TZA	Neonatal resuscitation	53.6	41.2
United Republic of Tanzania	TZA	Antibiotics for preterm or prolonged PROM	32.2	24.8
United Republic of Tanzania	TZA	Parenteral administration of anti-convulsants	49.9	38.4
United Republic of Tanzania	TZA	Parenteral administration of uterotonics	57.1	43.9
United Republic of Tanzania	TZA	Parenteral administration of antibiotics	32.2	24.8
United Republic of Tanzania	TZA	Assisted vaginal delivery	15.8	12.2
United Republic of Tanzania	TZA	Manual removal of placenta	24.5	18.9
United Republic of Tanzania	TZA	Removal of retained products of conception	15.3	11.8
United Republic of Tanzania	TZA	Cesarean delivery	5.4	4.2
United Republic of Tanzania	TZA	Blood transfusion	16.9	13.0
United Republic of Tanzania	TZA	Induction of labor for pregnancies lasting 41+ weeks	6.5	5.0
United Republic of Tanzania	TZA	Complementary feeding - education only	26.0	20.1
United Republic of Tanzania	TZA	Complementary feeding - supplementary feeding and education	26.0	20.1
United Republic of Tanzania	TZA	Vitamin A supplementation	87.0	67.1
United Republic of Tanzania	TZA	Improved sanitation - Utilization of latrines or toilets	29.9	29.9
United Republic of Tanzania	TZA	Improved water source	56.7	56.7
United Republic of Tanzania	TZA	Water connection in the home	20.3	20.3
United Republic of Tanzania	TZA	Hand washing with soap	51.9	51.9
United Republic of Tanzania	TZA	Hygienic disposal of children's stools	67.9	67.9
United Republic of Tanzania	TZA	ITN/IRS - Households protected from malaria	77.9	60.1
United Republic of Tanzania	TZA	Injectable antibiotics for neonatal sepsis	62.6	45.8
United Republic of Tanzania	TZA	ORS - oral rehydration solution	44.8	34.5

United Republic of Tanzania	TZA	Antibiotics for treatment of dysentery	2.8	2.2
United Republic of Tanzania	TZA	Zinc for treatment of diarrhea	17.5	13.5
United Republic of Tanzania	TZA	Oral antibiotics for pneumonia	55.4	42.6
United Republic of Tanzania	TZA	Vitamin A for treatment of measles	87.0	66.9
United Republic of Tanzania	TZA	ACTs- Artemisinin compounds for treatment of malaria	30.0	23.1
United Republic of Tanzania	TZA	SAM - treatment for severe acute malnutrition	0.3	0.2
United Republic of Tanzania	TZA	BCG vaccine	99.0	72.4
United Republic of Tanzania	TZA	Polio vaccine	91.0	66.5
United Republic of Tanzania	TZA	DPT vaccine	98.0	71.6
United Republic of Tanzania	TZA	H. influenzae type b vaccine	98.0	89.6
United Republic of Tanzania	TZA	HepB vaccine	98.0	71.6
United Republic of Tanzania	TZA	Pneumococcal vaccine	98.0	89.6
United Republic of Tanzania	TZA	Rotavirus vaccine	98.0	89.6
United Republic of Tanzania	TZA	Measles vaccine	99.0	72.4
United Republic of Tanzania	TZA	Global wasting (<-2 SD) rate	4.6	5.6
United Republic of Tanzania	TZA	Contraceptive prevalence (CPR)	43.8	35.6
Uzbekistan	UZB	Safe abortion services	35.1	28.5
Uzbekistan	UZB	Syphilis detection and treatment	24.5	17.9
Uzbekistan	UZB	Hypertensive disorder case management	18.8	13.7
Uzbekistan	UZB	Diabetes case management	14.7	10.7
Uzbekistan	UZB	Malaria case management	60.7	44.4
Uzbekistan	UZB	MgSO4 management of pre-eclampsia	37.2	27.2
Uzbekistan	UZB	Thermal protection	96.2	74.0
Uzbekistan	UZB	Clean cord care	92.9	71.5
Uzbekistan	UZB	Clean birth environment	79.8	61.4
Uzbekistan	UZB	Immediate drying and additional stimulation	89.1	68.6
Uzbekistan	UZB	Neonatal resuscitation	53.5	41.2
Uzbekistan	UZB	Antibiotics for preterm or prolonged PROM	72.8	56.0
Uzbekistan	UZB	Parenteral administration of anti-convulsants	69.6	53.6
Uzbekistan	UZB	Parenteral administration of uterotonics	87.0	66.9
Uzbekistan	UZB	Parenteral administration of antibiotics	72.8	56.0
Uzbekistan	UZB	Assisted vaginal delivery	24.6	18.9
Uzbekistan	UZB	Manual removal of placenta	36.4	28.0
Uzbekistan	UZB	Removal of retained products of conception	32.3	24.9

Uzbekistan	UZB	Cesarean delivery	8.5	6.5
Uzbekistan	UZB	Blood transfusion	12.3	9.5
Uzbekistan	UZB	Induction of labor for pregnancies lasting 41+ weeks	1.7	1.3
Uzbekistan	UZB	Complementary feeding - education only	27.6	21.3
Uzbekistan	UZB	Complementary feeding - supplementary feeding and education	27.6	21.3
Uzbekistan	UZB	Vitamin A supplementation	99.0	76.4
Uzbekistan	UZB	Improved sanitation - Utilization of latrines or toilets	99.0	99.0
Uzbekistan	UZB	Improved water source	97.8	97.8
Uzbekistan	UZB	Water connection in the home	68.7	68.7
Uzbekistan	UZB	Hygienic disposal of children's stools	58.6	58.6
Uzbekistan	UZB	Injectable antibiotics for neonatal sepsis	97.3	71.1
Uzbekistan	UZB	ORS - oral rehydration solution	27.8	21.4
Uzbekistan	UZB	Oral antibiotics for pneumonia	67.7	52.1
Uzbekistan	UZB	Vitamin A for treatment of measles	99.0	76.2
Uzbekistan	UZB	BCG vaccine	96.0	70.2
Uzbekistan	UZB	Polio vaccine	98.0	71.6
Uzbekistan	UZB	DPT vaccine	98.0	71.6
Uzbekistan	UZB	H. influenzae type b vaccine	98.0	89.6
Uzbekistan	UZB	HepB vaccine	98.0	71.6
Uzbekistan	UZB	Pneumococcal vaccine	96.0	87.7
Uzbekistan	UZB	Rotavirus vaccine	84.0	76.8
Uzbekistan	UZB	Measles vaccine	99.0	72.4
Uzbekistan	UZB	Global wasting (<-2 SD) rate	4.4	5.3
Uzbekistan	UZB	Contraceptive prevalence (CPR)	68.35	55.5
Vanuatu	VUT	Safe abortion services	85.0	69.0
Vanuatu	VUT	TT - Tetanus toxoid vaccination	78.0	57.0
Vanuatu	VUT	Syphilis detection and treatment	19.1	14.0
Vanuatu	VUT	Hypertensive disorder case management	12.4	9.1
Vanuatu	VUT	Diabetes case management	9.7	7.1
Vanuatu	VUT	Malaria case management	40.2	29.4
Vanuatu	VUT	MgSO4 management of pre-eclampsia	24.6	18.0
Vanuatu	VUT	Thermal protection	87.5	67.3
Vanuatu	VUT	Clean cord care	84.5	65.0
Vanuatu	VUT	Clean birth environment	72.6	55.9
Vanuatu	VUT	Immediate drying and additional stimulation	81.0	62.3
Vanuatu	VUT	Neonatal resuscitation	48.7	37.5
Vanuatu	VUT	Antibiotics for preterm or prolonged PROM	66.2	50.9
Vanuatu	VUT	Parenteral administration of anti-convulsants	63.3	48.7
Vanuatu	VUT	Parenteral administration of uterotonics	79.1	60.9
Vanuatu	VUT	Parenteral administration of antibiotics	66.2	50.9
Vanuatu	VUT	Assisted vaginal delivery	22.4	17.2

Vanuatu	VUT	Manual removal of placenta	33.1	25.5
Vanuatu	VUT	Removal of retained products of conception	29.3	22.5
Vanuatu	VUT	Cesarean delivery	7.7	5.9
Vanuatu	VUT	Blood transfusion	11.1	8.5
Vanuatu	VUT	Induction of labor for pregnancies lasting 41+ weeks	1.6	1.2
Vanuatu	VUT	Improved sanitation - Utilization of latrines or toilets	34.1	34.1
Vanuatu	VUT	Improved water source	91.3	91.3
Vanuatu	VUT	Water connection in the home	43.5	43.5
Vanuatu	VUT	Hygienic disposal of children's stools	62.7	62.7
Vanuatu	VUT	ITN/IRS - Households protected from malaria	69.8	53.9
Vanuatu	VUT	Injectable antibiotics for neonatal sepsis	88.5	64.7
Vanuatu	VUT	ORS - oral rehydration solution	47.6	36.6
Vanuatu	VUT	Antibiotics for treatment of dysentery	3.0	2.3
Vanuatu	VUT	Zinc for treatment of diarrhea	1.0	0.8
Vanuatu	VUT	Oral antibiotics for pneumonia	72.1	55.5
Vanuatu	VUT	BCG vaccine	94.0	68.7
Vanuatu	VUT	Polio vaccine	85.0	62.1
Vanuatu	VUT	DPT vaccine	85.0	62.1
Vanuatu	VUT	H. influenzae type b vaccine	85.0	77.7
Vanuatu	VUT	HepB vaccine	85.0	62.1
Vanuatu	VUT	Measles vaccine	80.0	58.5
Vanuatu	VUT	Global wasting (<-2 SD) rate	5.7	6.9
Vanuatu	VUT	Contraceptive prevalence (CPR)	50.25	40.8
Venezuela	VEN	Safe abortion services	0.2	0.2
Venezuela	VEN	TT - Tetanus toxoid vaccination	70.0	51.2
Venezuela	VEN	Thermal protection	93.9	72.3
Venezuela	VEN	Clean cord care	90.7	69.8
Venezuela	VEN	Clean birth environment	77.9	59.9
Venezuela	VEN	Immediate drying and additional stimulation	87.0	66.9
Venezuela	VEN	Neonatal resuscitation	52.2	40.2
Venezuela	VEN	Antibiotics for preterm or prolonged PROM	71.1	54.7
Venezuela	VEN	Parenteral administration of anti-convulsants	67.9	52.2
Venezuela	VEN	Parenteral administration of uterotonics	84.9	65.3
Venezuela	VEN	Parenteral administration of antibiotics	71.1	54.7
Venezuela	VEN	Assisted vaginal delivery	24.0	18.5
Venezuela	VEN	Manual removal of placenta	35.5	27.3
Venezuela	VEN	Removal of retained products of conception	31.5	24.2
Venezuela	VEN	Cesarean delivery	8.3	6.4
Venezuela	VEN	Blood transfusion	12.0	9.2
Venezuela	VEN	Induction of labor for pregnancies lasting 41+ weeks	1.7	1.3
Venezuela	VEN	Improved sanitation - Utilization of latrines or toilets	93.9	93.9

Venezuela	VEN	Improved water source	95.7	95.7
Venezuela	VEN	Water connection in the home	83.7	83.7
Venezuela	VEN	Injectable antibiotics for neonatal sepsis	95.0	69.4
Venezuela	VEN	BCG vaccine	92.0	67.3
Venezuela	VEN	Polio vaccine	53.0	38.7
Venezuela	VEN	DPT vaccine	60.0	43.9
Venezuela	VEN	H. influenzae type b vaccine	60.0	54.8
Venezuela	VEN	HepB vaccine	60.0	43.9
Venezuela	VEN	Measles vaccine	96.0	70.2
Venezuela	VEN	Global wasting (<-2 SD) rate	2.4	2.9
Venezuela	VEN	Contraceptive prevalence (CPR)	75.7	61.5
Viet Nam	VNM	Safe abortion services	39.0	31.7
Viet Nam	VNM	TT - Tetanus toxoid vaccination	94.0	68.7
Viet Nam	VNM	IPTp - Intermittent preventive treatment of malaria during pregnancy	0.5	0.4
Viet Nam	VNM	Syphilis detection and treatment	23.7	17.3
Viet Nam	VNM	Hypertensive disorder case management	17.7	12.9
Viet Nam	VNM	Diabetes case management	13.8	10.1
Viet Nam	VNM	Malaria case management	57.1	41.7
Viet Nam	VNM	MgSO4 management of pre-eclampsia	35.0	25.6
Viet Nam	VNM	Thermal protection	92.5	71.2
Viet Nam	VNM	Clean cord care	89.4	68.8
Viet Nam	VNM	Clean birth environment	76.8	59.1
Viet Nam	VNM	Immediate drying and additional stimulation	85.7	65.9
Viet Nam	VNM	Neonatal resuscitation	51.5	39.6
Viet Nam	VNM	Antibiotics for preterm or prolonged PROM	70.1	53.9
Viet Nam	VNM	Parenteral administration of anti-convulsants	67.0	51.6
Viet Nam	VNM	Parenteral administration of uterotonics	83.7	64.4
Viet Nam	VNM	Parenteral administration of antibiotics	70.1	53.9
Viet Nam	VNM	Assisted vaginal delivery	23.7	18.2
Viet Nam	VNM	Manual removal of placenta	35.0	26.9
Viet Nam	VNM	Removal of retained products of conception	31.0	23.9
Viet Nam	VNM	Cesarean delivery	8.1	6.2
Viet Nam	VNM	Blood transfusion	11.8	9.1
Viet Nam	VNM	Induction of labor for pregnancies lasting 41+ weeks	1.6	1.2
Viet Nam	VNM	Complementary feeding - education only	76.9	59.3
Viet Nam	VNM	Complementary feeding - supplementary feeding and education	76.9	59.3
Viet Nam	VNM	Vitamin A supplementation	99.0	76.4
Viet Nam	VNM	Improved sanitation - Utilization of latrines or toilets	83.5	83.5
Viet Nam	VNM	Improved water source	94.7	94.7
Viet Nam	VNM	Water connection in the home	40.6	40.6
Viet Nam	VNM	Hand washing with soap	86.3	86.3

Viet Nam	VNM	Hygienic disposal of children's stools	57.7	57.7
Viet Nam	VNM	ITN/IRS - Households protected from malaria	24.9	19.2
Viet Nam	VNM	Injectable antibiotics for neonatal sepsis	93.6	68.4
Viet Nam	VNM	ORS - oral rehydration solution	50.9	39.2
Viet Nam	VNM	Zinc for treatment of diarrhea	16.9	13.0
Viet Nam	VNM	Oral antibiotics for pneumonia	81.1	62.4
Viet Nam	VNM	Vitamin A for treatment of measles	99.0	76.2
Viet Nam	VNM	ACTs- Artemisinin compounds for treatment of malaria	0.1	0.1
Viet Nam	VNM	SAM - treatment for severe acute malnutrition	0.2	0.2
Viet Nam	VNM	BCG vaccine	95.0	69.4
Viet Nam	VNM	Polio vaccine	90.0	65.8
Viet Nam	VNM	DPT vaccine	75.0	54.8
Viet Nam	VNM	H. influenzae type b vaccine	75.0	68.5
Viet Nam	VNM	HepB vaccine	75.0	54.8
Viet Nam	VNM	Measles vaccine	97.0	70.9
Viet Nam	VNM	Global wasting (<-2 SD) rate	4.2	5.1
Viet Nam	VNM	Contraceptive prevalence (CPR)	76.8	62.4
Yemen	YEM	Safe abortion services	40.0	32.5
Yemen	YEM	TT - Tetanus toxoid vaccination	70.0	51.2
Yemen	YEM	Syphilis detection and treatment	14.7	10.7
Yemen	YEM	Iron supplementation in pregnancy	5.5	4.0
Yemen	YEM	Hypertensive disorder case management	5.9	4.3
Yemen	YEM	Diabetes case management	4.6	3.4
Yemen	YEM	Malaria case management	19.1	14.0
Yemen	YEM	MgSO4 management of pre-eclampsia	11.7	8.6
Yemen	YEM	Thermal protection	30.3	23.3
Yemen	YEM	Clean cord care	29.3	22.5
Yemen	YEM	Clean birth environment	25.2	19.4
Yemen	YEM	Immediate drying and additional stimulation	28.1	21.6
Yemen	YEM	Neonatal resuscitation	16.9	13.0
Yemen	YEM	Antibiotics for preterm or prolonged PROM	22.9	17.6
Yemen	YEM	Parenteral administration of anti-convulsants	21.9	16.9
Yemen	YEM	Parenteral administration of uterotonics	27.4	21.1
Yemen	YEM	Parenteral administration of antibiotics	22.9	17.6
Yemen	YEM	Assisted vaginal delivery	7.8	6.0
Yemen	YEM	Manual removal of placenta	11.5	8.8
Yemen	YEM	Removal of retained products of conception	10.2	7.8
Yemen	YEM	Cesarean delivery	2.7	2.1
Yemen	YEM	Blood transfusion	3.9	3.0
Yemen	YEM	Induction of labor for pregnancies lasting 41+ weeks	0.5	0.4
Yemen	YEM	Complementary feeding - education only	26.6	20.5

Yemen	YEM	Complementary feeding - supplementary feeding and education	26.6	20.5
Yemen	YEM	Vitamin A supplementation	81.0	62.5
Yemen	YEM	Improved sanitation - Utilization of latrines or toilets	59.1	59.1
Yemen	YEM	Improved water source	63.5	63.5
Yemen	YEM	Water connection in the home	30.7	30.7
Yemen	YEM	Hand washing with soap	62.0	62.0
Yemen	YEM	Hygienic disposal of children's stools	18.6	18.6
Yemen	YEM	Injectable antibiotics for neonatal sepsis	30.7	22.4
Yemen	YEM	ORS - oral rehydration solution	25.3	19.5
Yemen	YEM	Antibiotics for treatment of dysentery	38.2	29.4
Yemen	YEM	Zinc for treatment of diarrhea	0.4	0.3
Yemen	YEM	Oral antibiotics for pneumonia	34.0	26.2
Yemen	YEM	Vitamin A for treatment of measles	81.0	62.3
Yemen	YEM	BCG vaccine	64.0	46.8
Yemen	YEM	Polio vaccine	59.0	43.1
Yemen	YEM	DPT vaccine	65.0	47.5
Yemen	YEM	H. influenzae type b vaccine	65.0	59.4
Yemen	YEM	HepB vaccine	65.0	47.5
Yemen	YEM	Pneumococcal vaccine	64.0	58.5
Yemen	YEM	Rotavirus vaccine	64.0	58.5
Yemen	YEM	Measles vaccine	65.0	47.5
Yemen	YEM	Global wasting (<-2 SD) rate	16.3	19.6
Yemen	YEM	Contraceptive prevalence (CPR)	41.4	33.6
Zambia	ZMB	Safe abortion services	3.3	2.7
Zambia	ZMB	TT - Tetanus toxoid vaccination	85.0	62.1
Zambia	ZMB	IPTp - Intermittent preventive treatment of malaria during pregnancy	81.3	59.4
Zambia	ZMB	Syphilis detection and treatment	23.6	17.3
Zambia	ZMB	Iron supplementation in pregnancy	59.1	43.2
Zambia	ZMB	Hypertensive disorder case management	13.0	9.5
Zambia	ZMB	Diabetes case management	10.1	7.4
Zambia	ZMB	Malaria case management	42.0	30.7
Zambia	ZMB	MgSO4 management of pre-eclampsia	25.7	18.8
Zambia	ZMB	Thermal protection	66.4	51.1
Zambia	ZMB	Clean cord care	64.1	49.3
Zambia	ZMB	Clean birth environment	55.1	42.4
Zambia	ZMB	Immediate drying and additional stimulation	61.5	47.3
Zambia	ZMB	Neonatal resuscitation	36.9	28.4
Zambia	ZMB	Antibiotics for preterm or prolonged PROM	50.2	38.6
Zambia	ZMB	Parenteral administration of anti-convulsants	48.0	36.9
Zambia	ZMB	Parenteral administration of uterotonics	60.0	46.2
Zambia	ZMB	Parenteral administration of antibiotics	50.2	38.6

Zambia	ZMB	Assisted vaginal delivery	17.0	13.1
Zambia	ZMB	Manual removal of placenta	25.1	19.3
Zambia	ZMB	Removal of retained products of conception	22.2	17.1
Zambia	ZMB	Cesarean delivery	5.8	4.5
Zambia	ZMB	Blood transfusion	8.5	6.5
Zambia	ZMB	Induction of labor for pregnancies lasting 41+ weeks	1.2	0.9
Zambia	ZMB	Complementary feeding - education only	22.0	17.0
Zambia	ZMB	Complementary feeding - supplementary feeding and education	22.0	17.0
Zambia	ZMB	Vitamin A supplementation	99.0	76.4
Zambia	ZMB	Improved sanitation - Utilization of latrines or toilets	26.4	26.4
Zambia	ZMB	Improved water source	60.0	60.0
Zambia	ZMB	Water connection in the home	19.1	19.1
Zambia	ZMB	Hand washing with soap	33.7	33.7
Zambia	ZMB	Hygienic disposal of children's stools	70.8	70.8
Zambia	ZMB	ITN/IRS - Households protected from malaria	84.0	64.8
Zambia	ZMB	Injectable antibiotics for neonatal sepsis	67.1	49.1
Zambia	ZMB	ORS - oral rehydration solution	52.5	40.4
Zambia	ZMB	Antibiotics for treatment of dysentery	37.8	29.1
Zambia	ZMB	Oral antibiotics for pneumonia	69.7	53.6
Zambia	ZMB	Vitamin A for treatment of measles	99.0	76.2
Zambia	ZMB	ACTs- Artemisinin compounds for treatment of malaria	18.4	14.2
Zambia	ZMB	BCG vaccine	91.0	66.5
Zambia	ZMB	Polio vaccine	90.0	65.8
Zambia	ZMB	DPT vaccine	90.0	65.8
Zambia	ZMB	H. influenzae type b vaccine	90.0	82.3
Zambia	ZMB	HepB vaccine	90.0	65.8
Zambia	ZMB	Pneumococcal vaccine	90.0	82.3
Zambia	ZMB	Rotavirus vaccine	91.0	83.2
Zambia	ZMB	Measles vaccine	96.0	70.2
Zambia	ZMB	Global wasting (<-2 SD) rate	6.3	7.6
Zambia	ZMB	Contraceptive prevalence (CPR)	55.3	44.9
Zimbabwe	ZWE	Safe abortion services	3.3	2.7
Zimbabwe	ZWE	TT - Tetanus toxoid vaccination	87.0	63.6
Zimbabwe	ZWE	IPTp - Intermittent preventive treatment of malaria during pregnancy	25.9	18.9
Zimbabwe	ZWE	Syphilis detection and treatment	80.5	58.8
Zimbabwe	ZWE	Iron supplementation in pregnancy	39.7	29.0
Zimbabwe	ZWE	Hypertensive disorder case management	28.7	21.0
Zimbabwe	ZWE	Diabetes case management	27.8	20.3
Zimbabwe	ZWE	Malaria case management	68.3	49.9
Zimbabwe	ZWE	MgSO4 management of pre-eclampsia	69.4	50.7

Zimbabwe	ZWE	Thermal protection	85.5	65.8
Zimbabwe	ZWE	Clean cord care	75.5	58.1
Zimbabwe	ZWE	Clean birth environment	67.6	52.0
Zimbabwe	ZWE	Immediate drying and additional stimulation	76.3	58.7
Zimbabwe	ZWE	Neonatal resuscitation	70.4	54.2
Zimbabwe	ZWE	Antibiotics for preterm or prolonged PROM	45.9	35.3
Zimbabwe	ZWE	Parenteral administration of anti-convulsants	85.3	65.6
Zimbabwe	ZWE	Parenteral administration of uterotonics	82.2	63.3
Zimbabwe	ZWE	Parenteral administration of antibiotics	45.9	35.3
Zimbabwe	ZWE	Assisted vaginal delivery	18.0	13.9
Zimbabwe	ZWE	Manual removal of placenta	8.5	6.5
Zimbabwe	ZWE	Removal of retained products of conception	16.8	12.9
Zimbabwe	ZWE	Cesarean delivery	0.5	0.4
Zimbabwe	ZWE	Blood transfusion	10.8	8.3
Zimbabwe	ZWE	Induction of labor for pregnancies lasting 41+ weeks	1.5	1.2
Zimbabwe	ZWE	Complementary feeding - education only	16.7	12.9
Zimbabwe	ZWE	Complementary feeding - supplementary feeding and education	16.7	12.9
Zimbabwe	ZWE	Vitamin A supplementation	43.0	33.2
Zimbabwe	ZWE	Improved sanitation - Utilization of latrines or toilets	36.2	36.2
Zimbabwe	ZWE	Improved water source	64.1	64.1
Zimbabwe	ZWE	Water connection in the home	19.9	19.9
Zimbabwe	ZWE	Hand washing with soap	64.2	64.2
Zimbabwe	ZWE	Hygienic disposal of children's stools	64.4	64.4
Zimbabwe	ZWE	ITN/IRS - Households protected from malaria	36.8	28.4
Zimbabwe	ZWE	Injectable antibiotics for neonatal sepsis	85.5	62.5
Zimbabwe	ZWE	ORS - oral rehydration solution	32.8	25.2
Zimbabwe	ZWE	Antibiotics for treatment of dysentery	8.0	6.2
Zimbabwe	ZWE	Zinc for treatment of diarrhea	22.5	17.3
Zimbabwe	ZWE	Oral antibiotics for pneumonia	50.9	39.2
Zimbabwe	ZWE	Vitamin A for treatment of measles	43.0	33.1
Zimbabwe	ZWE	ACTs- Artemisinin compounds for treatment of malaria	0.4	0.3
Zimbabwe	ZWE	SAM - treatment for severe acute malnutrition	8.9	6.8
Zimbabwe	ZWE	BCG vaccine	95.0	69.4
Zimbabwe	ZWE	Polio vaccine	89.0	65.1
Zimbabwe	ZWE	DPT vaccine	89.0	65.1
Zimbabwe	ZWE	H. influenzae type b vaccine	89.0	81.3
Zimbabwe	ZWE	HepB vaccine	89.0	65.1
Zimbabwe	ZWE	Pneumococcal vaccine	89.0	81.3
Zimbabwe	ZWE	Rotavirus vaccine	90.0	82.3
Zimbabwe	ZWE	Measles vaccine	90.0	65.8
Zimbabwe	ZWE	Global wasting (<-2 SD) rate	3.6	4.3

Zimbabwe	ZWE	Contraceptive prevalence (CPR)	68.3	55.5

Appendix B3. Country-specific coverage reductions for Scenario 3

Country	ISO 3166-1 alpha-3	Intervention Name	Baseline coverage (%)	Ending coverage (%)
Afghanistan	AFG	Safe abortion services	35.1	21.3
Afghanistan	AFG	TT - Tetanus toxoid vaccination	70.0	33.7
Afghanistan	AFG	Syphilis detection and treatment	14.6	7.0
Afghanistan	AFG	Iron supplementation in pregnancy	6.8	3.3
Afghanistan	AFG	Hypertensive disorder case management	4.3	2.1
Afghanistan	AFG	Diabetes case management	3.3	1.6
Afghanistan	AFG	Malaria case management	13.8	6.6
Afghanistan	AFG	MgSO4 management of pre-eclampsia	8.5	4.1
Afghanistan	AFG	Thermal protection	47.7	24.1
Afghanistan	AFG	Clean cord care	46.1	23.3
Afghanistan	AFG	Clean birth environment	39.6	20.0
Afghanistan	AFG	Immediate drying and additional stimulation	44.2	22.4
Afghanistan	AFG	Neonatal resuscitation	26.6	13.5
Afghanistan	AFG	Antibiotics for preterm or prolonged PROM	36.1	18.3
Afghanistan	AFG	Parenteral administration of anti-convulsants	34.5	17.5
Afghanistan	AFG	Parenteral administration of uterotonics	43.2	21.9
Afghanistan	AFG	Parenteral administration of antibiotics	36.1	18.3
Afghanistan	AFG	Assisted vaginal delivery	12.2	6.2
Afghanistan	AFG	Manual removal of placenta	18.1	9.2
Afghanistan	AFG	Removal of retained products of conception	16.0	8.1
Afghanistan	AFG	Cesarean delivery	4.2	2.1
Afghanistan	AFG	Blood transfusion	6.1	3.1
Afghanistan	AFG	Induction of labor for pregnancies lasting 41+ weeks	0.9	0.5
Afghanistan	AFG	Complementary feeding - education only	24.3	14.0
Afghanistan	AFG	Complementary feeding - supplementary feeding and education	24.3	14.0
Afghanistan	AFG	Vitamin A supplementation	95.0	54.8
Afghanistan	AFG	Improved sanitation - Utilization of latrines or toilets	43.4	43.4
Afghanistan	AFG	Improved water source	67.1	67.1
Afghanistan	AFG	Water connection in the home	14.5	14.5
Afghanistan	AFG	Hand washing with soap	45.6	45.6
Afghanistan	AFG	Hygienic disposal of children's stools	30.9	30.9
Afghanistan	AFG	ITN/IRS - Households protected from malaria	26.0	15.0
Afghanistan	AFG	Injectable antibiotics for neonatal sepsis	48.3	23.2
Afghanistan	AFG	ORS - oral rehydration solution	46.2	23.4
Afghanistan	AFG	Antibiotics for treatment of dysentery	8.2	4.2
Afghanistan	AFG	Zinc for treatment of diarrhea	9.6	4.9
Afghanistan	AFG	Oral antibiotics for pneumonia	61.4	31.1
Afghanistan	AFG	Vitamin A for treatment of measles	95.0	48.1
Afghanistan	AFG	SAM - treatment for severe acute malnutrition	9.2	4.7

Country	ISO 3166-1 alpha-3	Intervention Name	Baseline coverage (%)	Ending coverage (%)
Afghanistan	AFG	BCG vaccine	78.0	37.5
Afghanistan	AFG	Polio vaccine	73.0	35.1
Afghanistan	AFG	DPT vaccine	66.0	31.7
Afghanistan	AFG	H. influenzae type b vaccine	66.0	55.0
Afghanistan	AFG	HepB vaccine	66.0	31.7
Afghanistan	AFG	Pneumococcal vaccine	65.0	54.2
Afghanistan	AFG	Rotavirus vaccine	60.0	50.0
Afghanistan	AFG	Measles vaccine	64.0	30.8
Afghanistan	AFG	Global wasting (<-2 SD) rate	10.6	15.9
Afghanistan	AFG	Contraceptive prevalence (CPR)	27.8	16.9
Albania	ALB	Safe abortion services	100.0	60.8
Albania	ALB	TT - Tetanus toxoid vaccination	95.0	45.7
Albania	ALB	Syphilis detection and treatment	21.9	10.5
Albania	ALB	Iron supplementation in pregnancy	18.5	8.9
Albania	ALB	Hypertensive disorder case management	19.0	9.1
Albania	ALB	Diabetes case management	14.8	7.1
Albania	ALB	Malaria case management	61.2	29.4
Albania	ALB	MgSO4 management of pre-eclampsia	37.5	18.0
Albania	ALB	Thermal protection	97.4	49.3
Albania	ALB	Clean cord care	94.1	47.6
Albania	ALB	Clean birth environment	80.9	41.0
Albania	ALB	Immediate drying and additional stimulation	90.3	45.7
Albania	ALB	Neonatal resuscitation	54.2	27.4
Albania	ALB	Antibiotics for preterm or prolonged PROM	73.8	37.4
Albania	ALB	Parenteral administration of anti-convulsants	70.5	35.7
Albania	ALB	Parenteral administration of uterotonics	88.1	44.6
Albania	ALB	Parenteral administration of antibiotics	73.8	37.4
Albania	ALB	Assisted vaginal delivery	24.9	12.6
Albania	ALB	Manual removal of placenta	36.9	18.7
Albania	ALB	Removal of retained products of conception	32.7	16.6
Albania	ALB	Cesarean delivery	8.6	4.4
Albania	ALB	Blood transfusion	12.4	6.3
Albania	ALB	Induction of labor for pregnancies lasting 41+ weeks	1.7	0.9
Albania	ALB	Complementary feeding - education only	59.3	34.2
Albania	ALB	Complementary feeding - supplementary feeding and education	59.3	34.2
Albania	ALB	Improved sanitation - Utilization of latrines or toilets	97.7	97.7
Albania	ALB	Improved water source	91.0	91.0
Albania	ALB	Water connection in the home	78.5	78.5
Albania	ALB	Hygienic disposal of children's stools	13.5	13.5
Albania	ALB	Injectable antibiotics for neonatal sepsis	98.6	47.4

Country	ISO 3166-1 alpha-3	Intervention Name	Baseline coverage (%)	Ending coverage (%)
Albania	ALB	ORS - oral rehydration solution	34.5	17.5
Albania	ALB	Antibiotics for treatment of dysentery	38.7	19.6
Albania	ALB	Zinc for treatment of diarrhea	14.9	7.5
Albania	ALB	Oral antibiotics for pneumonia	81.8	41.4
Albania	ALB	BCG vaccine	99.0	47.6
Albania	ALB	Polio vaccine	99.0	47.6
Albania	ALB	DPT vaccine	99.0	47.6
Albania	ALB	H. influenzae type b vaccine	99.0	82.6
Albania	ALB	HepB vaccine	99.0	47.6
Albania	ALB	Pneumococcal vaccine	98.0	81.7
Albania	ALB	Measles vaccine	94.0	45.2
Albania	ALB	Global wasting (<-2 SD) rate	1.4	2.1
Albania	ALB	Contraceptive prevalence (CPR)	44	26.7
Algeria	DZA	Safe abortion services	2.2	1.3
Algeria	DZA	TT - Tetanus toxoid vaccination	98.0	47.1
Algeria	DZA	Syphilis detection and treatment	22.9	11.0
Algeria	DZA	Hypertensive disorder case management	16.2	7.8
Algeria	DZA	Diabetes case management	12.6	6.1
Algeria	DZA	Malaria case management	52.1	25.1
Algeria	DZA	MgSO4 management of pre-eclampsia	32.0	15.4
Algeria	DZA	Thermal protection	95.5	48.3
Algeria	DZA	Clean cord care	92.2	46.7
Algeria	DZA	Clean birth environment	79.2	40.1
Algeria	DZA	Immediate drying and additional stimulation	88.5	44.8
Algeria	DZA	Neonatal resuscitation	53.1	26.9
Algeria	DZA	Antibiotics for preterm or prolonged PROM	72.3	36.6
Algeria	DZA	Parenteral administration of anti-convulsants	69.1	35.0
Algeria	DZA	Parenteral administration of uterotonics	86.3	43.7
Algeria	DZA	Parenteral administration of antibiotics	72.3	36.6
Algeria	DZA	Assisted vaginal delivery	24.4	12.4
Algeria	DZA	Manual removal of placenta	36.1	18.3
Algeria	DZA	Removal of retained products of conception	32.0	16.2
Algeria	DZA	Cesarean delivery	8.4	4.3
Algeria	DZA	Blood transfusion	12.2	6.2
Algeria	DZA	Induction of labor for pregnancies lasting 41+ weeks	1.7	0.9
Algeria	DZA	Improved sanitation - Utilization of latrines or toilets	87.6	87.6
Algeria	DZA	Improved water source	93.6	93.6
Algeria	DZA	Water connection in the home	71.8	71.8
Algeria	DZA	Hand washing with soap	84.2	84.2
Algeria	DZA	Hygienic disposal of children's stools	17.7	17.7
Algeria	DZA	Injectable antibiotics for neonatal sepsis	96.6	46.5

**Country**	**ISO 3166-1 alpha-3**	**Intervention Name**	**Baseline coverage (%)**	**Ending coverage (%)**
Algeria	DZA	ORS - oral rehydration solution	33.9	17.2
Algeria	DZA	Oral antibiotics for pneumonia	66.4	33.6
Algeria	DZA	BCG vaccine	99.0	47.6
Algeria	DZA	Polio vaccine	91.0	43.8
Algeria	DZA	DPT vaccine	91.0	43.8
Algeria	DZA	H. influenzae type b vaccine	91.0	75.9
Algeria	DZA	HepB vaccine	91.0	43.8
Algeria	DZA	Pneumococcal vaccine	91.0	75.9
Algeria	DZA	Measles vaccine	80.0	38.5
Algeria	DZA	Global wasting (<-2 SD) rate	4.0	6.0
Algeria	DZA	Contraceptive prevalence (CPR)	61.05	37.1
Angola	AGO	TT - Tetanus toxoid vaccination	78.0	37.5
Angola	AGO	IPTp - Intermittent preventive treatment of malaria during pregnancy	38.4	18.5
Angola	AGO	Syphilis detection and treatment	20.0	9.6
Angola	AGO	Iron supplementation in pregnancy	32.1	15.4
Angola	AGO	Hypertensive disorder case management	14.5	7.0
Angola	AGO	Diabetes case management	11.3	5.4
Angola	AGO	Malaria case management	46.7	22.5
Angola	AGO	MgSO4 management of pre-eclampsia	28.6	13.8
Angola	AGO	Thermal protection	45.1	22.8
Angola	AGO	Clean cord care	43.5	22.0
Angola	AGO	Clean birth environment	37.4	18.9
Angola	AGO	Immediate drying and additional stimulation	41.8	21.2
Angola	AGO	Neonatal resuscitation	25.1	12.7
Angola	AGO	Antibiotics for preterm or prolonged PROM	34.1	17.3
Angola	AGO	Parenteral administration of anti-convulsants	32.6	16.5
Angola	AGO	Parenteral administration of uterotonics	40.8	20.7
Angola	AGO	Parenteral administration of antibiotics	34.1	17.3
Angola	AGO	Assisted vaginal delivery	11.5	5.8
Angola	AGO	Manual removal of placenta	17.1	8.7
Angola	AGO	Removal of retained products of conception	15.1	7.6
Angola	AGO	Cesarean delivery	4.0	2.0
Angola	AGO	Blood transfusion	5.7	2.9
Angola	AGO	Induction of labor for pregnancies lasting 41+ weeks	0.8	0.4
Angola	AGO	Complementary feeding - education only	33.3	19.2
Angola	AGO	Complementary feeding - supplementary feeding and education	33.3	19.2
Angola	AGO	Vitamin A supplementation	3.0	1.7
Angola	AGO	Improved sanitation - Utilization of latrines or toilets	49.9	49.9
Angola	AGO	Improved water source	55.8	55.8
Angola	AGO	Water connection in the home	22.6	22.6

Country	ISO 3166-1 alpha-3	Intervention Name	Baseline coverage (%)	Ending coverage (%)
Angola	AGO	Hand washing with soap	27.0	27.0
Angola	AGO	Hygienic disposal of children's stools	27.5	27.5
Angola	AGO	ITN/IRS - Households protected from malaria	31.8	18.4
Angola	AGO	Injectable antibiotics for neonatal sepsis	45.6	21.9
Angola	AGO	ORS - oral rehydration solution	42.6	21.6
Angola	AGO	Oral antibiotics for pneumonia	58.7	29.7
Angola	AGO	Vitamin A for treatment of measles	3.0	1.5
Angola	AGO	ACTs- Artemisinin compounds for treatment of malaria	9.0	4.6
Angola	AGO	SAM - treatment for severe acute malnutrition	1.4	0.7
Angola	AGO	BCG vaccine	86.0	41.4
Angola	AGO	Polio vaccine	56.0	26.9
Angola	AGO	DPT vaccine	59.0	28.4
Angola	AGO	H. influenzae type b vaccine	59.0	49.2
Angola	AGO	HepB vaccine	59.0	28.4
Angola	AGO	Pneumococcal vaccine	67.0	55.9
Angola	AGO	Rotavirus vaccine	65.0	54.2
Angola	AGO	Measles vaccine	50.0	24.0
Angola	AGO	Global wasting (<-2 SD) rate	4.9	7.4
Angola	AGO	Contraceptive prevalence (CPR)	16.25	9.9
Argentina	ARG	Safe abortion services	0.2	0.1
Argentina	ARG	Syphilis detection and treatment	24.2	11.6
Argentina	ARG	Hypertensive disorder case management	21.6	10.4
Argentina	ARG	Diabetes case management	16.8	8.1
Argentina	ARG	Malaria case management	69.6	33.5
Argentina	ARG	MgSO4 management of pre-eclampsia	42.7	20.5
Argentina	ARG	Thermal protection	97.1	49.2
Argentina	ARG	Clean cord care	93.7	47.4
Argentina	ARG	Clean birth environment	80.5	40.8
Argentina	ARG	Immediate drying and additional stimulation	89.9	45.5
Argentina	ARG	Neonatal resuscitation	54.0	27.3
Argentina	ARG	Antibiotics for preterm or prolonged PROM	73.5	37.2
Argentina	ARG	Parenteral administration of anti-convulsants	70.2	35.5
Argentina	ARG	Parenteral administration of uterotonics	87.8	44.4
Argentina	ARG	Parenteral administration of antibiotics	73.5	37.2
Argentina	ARG	Assisted vaginal delivery	24.8	12.6
Argentina	ARG	Manual removal of placenta	36.7	18.6
Argentina	ARG	Removal of retained products of conception	32.5	16.5
Argentina	ARG	Cesarean delivery	8.5	4.3
Argentina	ARG	Blood transfusion	12.4	6.3
Argentina	ARG	Induction of labor for pregnancies lasting 41+ weeks	1.7	0.9
Argentina	ARG	Improved sanitation - Utilization of latrines or toilets	94.3	94.3

**Country**	**ISO 3166-1 alpha-3**	**Intervention Name**	**Baseline coverage (%)**	**Ending coverage (%)**
Argentina	ARG	Improved water source	99.0	99.0
Argentina	ARG	Water connection in the home	95.7	95.7
Argentina	ARG	Injectable antibiotics for neonatal sepsis	98.2	47.2
Argentina	ARG	ORS - oral rehydration solution	17.5	8.9
Argentina	ARG	Oral antibiotics for pneumonia	94.5	47.8
Argentina	ARG	BCG vaccine	93.0	44.7
Argentina	ARG	Polio vaccine	84.0	40.4
Argentina	ARG	DPT vaccine	86.0	41.4
Argentina	ARG	H. influenzae type b vaccine	86.0	71.7
Argentina	ARG	HepB vaccine	86.0	41.4
Argentina	ARG	Pneumococcal vaccine	88.0	73.4
Argentina	ARG	Rotavirus vaccine	80.0	66.7
Argentina	ARG	Measles vaccine	89.0	42.8
Argentina	ARG	Global wasting (<-2 SD) rate	2.4	3.6
Argentina	ARG	Contraceptive prevalence (CPR)	63.95	38.8
Armenia	ARM	Safe abortion services	40.0	24.3
Armenia	ARM	Syphilis detection and treatment	24.7	11.9
Armenia	ARM	Iron supplementation in pregnancy	4.5	2.2
Armenia	ARM	Hypertensive disorder case management	23.2	11.2
Armenia	ARM	Diabetes case management	18.1	8.7
Armenia	ARM	Malaria case management	74.7	35.9
Armenia	ARM	MgSO4 management of pre-eclampsia	45.8	22.0
Armenia	ARM	Thermal protection	98.5	49.9
Armenia	ARM	Clean cord care	95.1	48.1
Armenia	ARM	Clean birth environment	81.8	41.4
Armenia	ARM	Immediate drying and additional stimulation	91.3	46.2
Armenia	ARM	Neonatal resuscitation	54.8	27.7
Armenia	ARM	Antibiotics for preterm or prolonged PROM	74.6	37.8
Armenia	ARM	Parenteral administration of anti-convulsants	71.3	36.1
Armenia	ARM	Parenteral administration of uterotonics	89.1	45.1
Armenia	ARM	Parenteral administration of antibiotics	74.6	37.8
Armenia	ARM	Assisted vaginal delivery	25.2	12.8
Armenia	ARM	Manual removal of placenta	37.3	18.9
Armenia	ARM	Removal of retained products of conception	33.0	16.7
Armenia	ARM	Cesarean delivery	8.7	4.4
Armenia	ARM	Blood transfusion	12.6	6.4
Armenia	ARM	Induction of labor for pregnancies lasting 41+ weeks	1.8	0.9
Armenia	ARM	Complementary feeding - education only	50.2	29.0
Armenia	ARM	Complementary feeding - supplementary feeding and education	50.2	29.0
Armenia	ARM	Improved sanitation - Utilization of latrines or toilets	93.6	93.6

Country	ISO 3166-1 alpha-3	Intervention Name	Baseline coverage (%)	Ending coverage (%)
Armenia	ARM	Improved water source	99.0	99.0
Armenia	ARM	Water connection in the home	97.9	97.9
Armenia	ARM	Hand washing with soap	95.8	95.8
Armenia	ARM	Hygienic disposal of children's stools	43.4	43.4
Armenia	ARM	Injectable antibiotics for neonatal sepsis	99.7	47.9
Armenia	ARM	ORS - oral rehydration solution	36.9	18.7
Armenia	ARM	Antibiotics for treatment of dysentery	1.3	0.7
Armenia	ARM	Zinc for treatment of diarrhea	3.7	1.9
Armenia	ARM	Oral antibiotics for pneumonia	91.7	46.4
Armenia	ARM	BCG vaccine	99.0	47.6
Armenia	ARM	Polio vaccine	92.0	44.2
Armenia	ARM	DPT vaccine	92.0	44.2
Armenia	ARM	H. influenzae type b vaccine	92.0	76.7
Armenia	ARM	HepB vaccine	92.0	44.2
Armenia	ARM	Pneumococcal vaccine	92.0	76.7
Armenia	ARM	Rotavirus vaccine	93.0	77.6
Armenia	ARM	Meningococcal A	22.0	10.6
Armenia	ARM	Measles vaccine	95.0	45.7
Armenia	ARM	Global wasting (<-2 SD) rate	4.5	6.7
Armenia	ARM	Contraceptive prevalence (CPR)	58.55	35.6
Azerbaijan	AZE	Safe abortion services	40.0	24.3
Azerbaijan	AZE	Syphilis detection and treatment	19.6	9.4
Azerbaijan	AZE	Iron supplementation in pregnancy	1.6	0.8
Azerbaijan	AZE	Hypertensive disorder case management	11.8	5.7
Azerbaijan	AZE	Diabetes case management	9.2	4.4
Azerbaijan	AZE	Malaria case management	38.1	18.3
Azerbaijan	AZE	MgSO4 management of pre-eclampsia	23.4	11.3
Azerbaijan	AZE	Thermal protection	76.8	38.9
Azerbaijan	AZE	Clean cord care	74.1	37.5
Azerbaijan	AZE	Clean birth environment	63.7	32.2
Azerbaijan	AZE	Immediate drying and additional stimulation	71.1	36.0
Azerbaijan	AZE	Neonatal resuscitation	42.7	21.6
Azerbaijan	AZE	Antibiotics for preterm or prolonged PROM	58.1	29.4
Azerbaijan	AZE	Parenteral administration of anti-convulsants	55.5	28.1
Azerbaijan	AZE	Parenteral administration of uterotonics	69.4	35.1
Azerbaijan	AZE	Parenteral administration of antibiotics	58.1	29.4
Azerbaijan	AZE	Assisted vaginal delivery	19.6	9.9
Azerbaijan	AZE	Manual removal of placenta	29.0	14.7
Azerbaijan	AZE	Removal of retained products of conception	25.7	13.0
Azerbaijan	AZE	Cesarean delivery	6.8	3.4
Azerbaijan	AZE	Blood transfusion	9.8	5.0

Country	ISO 3166-1 alpha-3	Intervention Name	Baseline coverage (%)	Ending coverage (%)
Azerbaijan	AZE	Induction of labor for pregnancies lasting 41+ weeks	1.4	0.7
Azerbaijan	AZE	Complementary feeding - education only	50.9	29.4
Azerbaijan	AZE	Complementary feeding - supplementary feeding and education	50.9	29.4
Azerbaijan	AZE	Vitamin A supplementation	41.0	23.7
Azerbaijan	AZE	Improved sanitation - Utilization of latrines or toilets	92.5	92.5
Azerbaijan	AZE	Improved water source	91.4	91.4
Azerbaijan	AZE	Water connection in the home	78.5	78.5
Azerbaijan	AZE	Injectable antibiotics for neonatal sepsis	77.7	37.4
Azerbaijan	AZE	ORS - oral rehydration solution	21.1	10.7
Azerbaijan	AZE	Oral antibiotics for pneumonia	32.5	16.5
Azerbaijan	AZE	Vitamin A for treatment of measles	41.0	20.8
Azerbaijan	AZE	BCG vaccine	97.0	46.7
Azerbaijan	AZE	Polio vaccine	96.0	46.2
Azerbaijan	AZE	DPT vaccine	95.0	45.7
Azerbaijan	AZE	H. influenzae type b vaccine	95.0	79.2
Azerbaijan	AZE	HepB vaccine	95.0	45.7
Azerbaijan	AZE	Pneumococcal vaccine	95.0	79.2
Azerbaijan	AZE	Measles vaccine	96.0	46.2
Azerbaijan	AZE	Global wasting (<-2 SD) rate	6.6	9.9
Azerbaijan	AZE	Contraceptive prevalence (CPR)	57	34.6
Bangladesh	BGD	Safe abortion services	35.1	21.3
Bangladesh	BGD	TT - Tetanus toxoid vaccination	98.0	47.1
Bangladesh	BGD	Syphilis detection and treatment	7.3	3.5
Bangladesh	BGD	Hypertensive disorder case management	8.9	4.3
Bangladesh	BGD	Diabetes case management	12.6	6.1
Bangladesh	BGD	Malaria case management	28.6	13.8
Bangladesh	BGD	MgSO4 management of pre-eclampsia	8.0	3.8
Bangladesh	BGD	Thermal protection	51.3	26.0
Bangladesh	BGD	Clean cord care	51.0	25.8
Bangladesh	BGD	Clean birth environment	37.8	19.1
Bangladesh	BGD	Immediate drying and additional stimulation	39.3	19.9
Bangladesh	BGD	Neonatal resuscitation	40.0	20.3
Bangladesh	BGD	Antibiotics for preterm or prolonged PROM	23.9	12.1
Bangladesh	BGD	Parenteral administration of anti-convulsants	37.8	19.1
Bangladesh	BGD	Parenteral administration of uterotonics	42.0	21.3
Bangladesh	BGD	Parenteral administration of antibiotics	23.9	12.1
Bangladesh	BGD	Assisted vaginal delivery	26.5	13.4
Bangladesh	BGD	Manual removal of placenta	12.2	6.2
Bangladesh	BGD	Removal of retained products of conception	30.2	15.3
Bangladesh	BGD	Cesarean delivery	7.6	3.8

Country	ISO 3166-1 alpha-3	Intervention Name	Baseline coverage (%)	Ending coverage (%)
Bangladesh	BGD	Blood transfusion	10.8	5.5
Bangladesh	BGD	Induction of labor for pregnancies lasting 41+ weeks	10.2	5.2
Bangladesh	BGD	Complementary feeding - education only	27.6	15.9
Bangladesh	BGD	Complementary feeding - supplementary feeding and education	27.6	15.9
Bangladesh	BGD	Vitamin A supplementation	99.0	57.1
Bangladesh	BGD	Improved sanitation - Utilization of latrines or toilets	48.2	48.2
Bangladesh	BGD	Improved water source	97.0	97.0
Bangladesh	BGD	Water connection in the home	14.5	14.5
Bangladesh	BGD	Hand washing with soap	74.8	74.8
Bangladesh	BGD	Hygienic disposal of children's stools	38.7	38.7
Bangladesh	BGD	Injectable antibiotics for neonatal sepsis	53.4	25.7
Bangladesh	BGD	ORS - oral rehydration solution	72.4	36.7
Bangladesh	BGD	Antibiotics for treatment of dysentery	9.7	4.9
Bangladesh	BGD	Zinc for treatment of diarrhea	43.6	22.1
Bangladesh	BGD	Oral antibiotics for pneumonia	46.4	23.5
Bangladesh	BGD	Vitamin A for treatment of measles	99.0	50.1
Bangladesh	BGD	SAM - treatment for severe acute malnutrition	0.3	0.2
Bangladesh	BGD	BCG vaccine	99.0	47.6
Bangladesh	BGD	Polio vaccine	98.0	47.1
Bangladesh	BGD	DPT vaccine	98.0	47.1
Bangladesh	BGD	H. influenzae type b vaccine	98.0	81.7
Bangladesh	BGD	HepB vaccine	98.0	47.1
Bangladesh	BGD	Pneumococcal vaccine	97.0	80.9
Bangladesh	BGD	Measles vaccine	97.0	46.7
Bangladesh	BGD	Global wasting (<-2 SD) rate	14.4	21.6
Bangladesh	BGD	Contraceptive prevalence (CPR)	65.15	39.6
Belarus	BLR	Safe abortion services	87.5	53.2
Belarus	BLR	Syphilis detection and treatment	24.6	11.8
Belarus	BLR	Hypertensive disorder case management	23.9	11.5
Belarus	BLR	Diabetes case management	18.7	9.0
Belarus	BLR	Malaria case management	77.3	37.2
Belarus	BLR	MgSO4 management of pre-eclampsia	47.4	22.8
Belarus	BLR	Thermal protection	98.7	50.0
Belarus	BLR	Clean cord care	95.3	48.2
Belarus	BLR	Clean birth environment	81.9	41.5
Belarus	BLR	Immediate drying and additional stimulation	91.5	46.3
Belarus	BLR	Neonatal resuscitation	54.9	27.8
Belarus	BLR	Antibiotics for preterm or prolonged PROM	74.8	37.9
Belarus	BLR	Parenteral administration of anti-convulsants	71.4	36.1
Belarus	BLR	Parenteral administration of uterotonics	89.3	45.2

**Country**	**ISO 3166-1 alpha-3**	**Intervention Name**	**Baseline coverage (%)**	**Ending coverage (%)**
Belarus	BLR	Parenteral administration of antibiotics	74.8	37.9
Belarus	BLR	Assisted vaginal delivery	25.3	12.8
Belarus	BLR	Manual removal of placenta	37.4	18.9
Belarus	BLR	Removal of retained products of conception	33.1	16.8
Belarus	BLR	Cesarean delivery	8.7	4.4
Belarus	BLR	Blood transfusion	12.6	6.4
Belarus	BLR	Induction of labor for pregnancies lasting 41+ weeks	1.8	0.9
Belarus	BLR	Improved sanitation - Utilization of latrines or toilets	97.8	97.8
Belarus	BLR	Improved water source	96.5	96.5
Belarus	BLR	Water connection in the home	89.6	89.6
Belarus	BLR	Hygienic disposal of children's stools	56.0	56.0
Belarus	BLR	Injectable antibiotics for neonatal sepsis	99.9	48.0
Belarus	BLR	ORS - oral rehydration solution	45.3	22.9
Belarus	BLR	Antibiotics for treatment of dysentery	22.3	11.3
Belarus	BLR	Oral antibiotics for pneumonia	93.4	47.3
Belarus	BLR	BCG vaccine	98.0	47.1
Belarus	BLR	Polio vaccine	98.0	47.1
Belarus	BLR	DPT vaccine	97.0	46.7
Belarus	BLR	H. influenzae type b vaccine	9.0	7.5
Belarus	BLR	HepB vaccine	98.0	47.1
Belarus	BLR	Measles vaccine	97.0	46.7
Belarus	BLR	Global wasting (<-2 SD) rate	2.2	3.3
Belarus	BLR	Contraceptive prevalence (CPR)	66.6	40.5
Belize	BLZ	Safe abortion services	0.6	0.4
Belize	BLZ	TT - Tetanus toxoid vaccination	91.0	43.8
Belize	BLZ	Syphilis detection and treatment	24.0	11.5
Belize	BLZ	Hypertensive disorder case management	22.3	10.7
Belize	BLZ	Diabetes case management	17.4	8.4
Belize	BLZ	Malaria case management	71.8	34.5
Belize	BLZ	MgSO4 management of pre-eclampsia	44.0	21.2
Belize	BLZ	Thermal protection	95.3	48.2
Belize	BLZ	Clean cord care	92.0	46.6
Belize	BLZ	Clean birth environment	79.1	40.0
Belize	BLZ	Immediate drying and additional stimulation	88.3	44.7
Belize	BLZ	Neonatal resuscitation	53.0	26.8
Belize	BLZ	Antibiotics for preterm or prolonged PROM	72.1	36.5
Belize	BLZ	Parenteral administration of anti-convulsants	68.9	34.9
Belize	BLZ	Parenteral administration of uterotonics	86.1	43.6
Belize	BLZ	Parenteral administration of antibiotics	72.1	36.5
Belize	BLZ	Assisted vaginal delivery	24.4	12.4
Belize	BLZ	Manual removal of placenta	36.1	18.3

Country	ISO 3166-1 alpha-3	Intervention Name	Baseline coverage (%)	Ending coverage (%)
Belize	BLZ	Removal of retained products of conception	31.9	16.1
Belize	BLZ	Cesarean delivery	8.4	4.3
Belize	BLZ	Blood transfusion	12.1	6.1
Belize	BLZ	Induction of labor for pregnancies lasting 41+ weeks	1.7	0.9
Belize	BLZ	Complementary feeding - education only	66.3	38.3
Belize	BLZ	Complementary feeding - supplementary feeding and education	66.3	38.3
Belize	BLZ	Vitamin A supplementation	44.0	25.4
Belize	BLZ	Improved sanitation - Utilization of latrines or toilets	87.9	87.9
Belize	BLZ	Improved water source	98.0	98.0
Belize	BLZ	Water connection in the home	84.1	84.1
Belize	BLZ	Hand washing with soap	90.5	90.5
Belize	BLZ	Hygienic disposal of children's stools	16.4	16.4
Belize	BLZ	Injectable antibiotics for neonatal sepsis	96.4	46.4
Belize	BLZ	ORS - oral rehydration solution	55.2	27.9
Belize	BLZ	Zinc for treatment of diarrhea	9.6	4.9
Belize	BLZ	Oral antibiotics for pneumonia	67.4	34.1
Belize	BLZ	Vitamin A for treatment of measles	44.0	22.3
Belize	BLZ	BCG vaccine	99.0	47.6
Belize	BLZ	Polio vaccine	96.0	46.2
Belize	BLZ	DPT vaccine	96.0	46.2
Belize	BLZ	H. influenzae type b vaccine	96.0	80.1
Belize	BLZ	HepB vaccine	96.0	46.2
Belize	BLZ	Measles vaccine	97.0	46.7
Belize	BLZ	Global wasting (<-2 SD) rate	1.9	2.9
Belize	BLZ	Contraceptive prevalence (CPR)	55.5	33.7
Benin	BEN	TT - Tetanus toxoid vaccination	85.0	40.9
Benin	BEN	IPTp - Intermittent preventive treatment of malaria during pregnancy	33.4	16.1
Benin	BEN	Syphilis detection and treatment	20.9	10.1
Benin	BEN	Iron supplementation in pregnancy	28.6	13.8
Benin	BEN	Hypertensive disorder case management	12.1	5.8
Benin	BEN	Diabetes case management	7.1	3.4
Benin	BEN	Malaria case management	42.7	20.5
Benin	BEN	MgSO4 management of pre-eclampsia	28.5	13.7
Benin	BEN	Thermal protection	77.2	39.1
Benin	BEN	Clean cord care	75.8	38.4
Benin	BEN	Clean birth environment	75.8	38.4
Benin	BEN	Immediate drying and additional stimulation	77.6	39.3
Benin	BEN	Neonatal resuscitation	42.9	21.7
Benin	BEN	Antibiotics for preterm or prolonged PROM	68.9	34.9
Benin	BEN	Parenteral administration of anti-convulsants	44.1	22.3

Country	ISO 3166-1 alpha-3	Intervention Name	Baseline coverage (%)	Ending coverage (%)
Benin	BEN	Parenteral administration of uterotonics	75.8	38.4
Benin	BEN	Parenteral administration of antibiotics	68.9	34.9
Benin	BEN	Assisted vaginal delivery	24.0	12.2
Benin	BEN	Manual removal of placenta	64.4	32.6
Benin	BEN	Removal of retained products of conception	49.9	25.3
Benin	BEN	Cesarean delivery	4.3	2.2
Benin	BEN	Blood transfusion	16.4	8.3
Benin	BEN	Induction of labor for pregnancies lasting 41+ weeks	1.5	0.8
Benin	BEN	Complementary feeding - education only	25.3	14.6
Benin	BEN	Complementary feeding - supplementary feeding and education	25.3	14.6
Benin	BEN	Vitamin A supplementation	99.0	57.1
Benin	BEN	Improved sanitation - Utilization of latrines or toilets	16.5	16.5
Benin	BEN	Improved water source	66.4	66.4
Benin	BEN	Water connection in the home	26.4	26.4
Benin	BEN	Hand washing with soap	8.4	8.4
Benin	BEN	Hygienic disposal of children's stools	34.8	34.8
Benin	BEN	ITN/IRS - Households protected from malaria	92.0	53.1
Benin	BEN	Injectable antibiotics for neonatal sepsis	78.1	37.6
Benin	BEN	ORS - oral rehydration solution	22.2	11.2
Benin	BEN	Antibiotics for treatment of dysentery	29.2	14.8
Benin	BEN	Zinc for treatment of diarrhea	17.0	8.6
Benin	BEN	Oral antibiotics for pneumonia	46.1	23.3
Benin	BEN	Vitamin A for treatment of measles	99.0	50.1
Benin	BEN	ACTs- Artemisinin compounds for treatment of malaria	2.5	1.3
Benin	BEN	SAM - treatment for severe acute malnutrition	0.8	0.4
Benin	BEN	BCG vaccine	89.0	42.8
Benin	BEN	Polio vaccine	75.0	36.1
Benin	BEN	DPT vaccine	76.0	36.6
Benin	BEN	H. influenzae type b vaccine	76.0	63.4
Benin	BEN	HepB vaccine	76.0	36.6
Benin	BEN	Pneumococcal vaccine	73.0	60.9
Benin	BEN	Measles vaccine	71.0	34.1
Benin	BEN	Global wasting (<-2 SD) rate	4.5	6.7
Benin	BEN	Contraceptive prevalence (CPR)	17.3	10.5
Bhutan	BTN	Safe abortion services	35.1	21.3
Bhutan	BTN	TT - Tetanus toxoid vaccination	89.0	42.8
Bhutan	BTN	Syphilis detection and treatment	24.0	11.5
Bhutan	BTN	Hypertensive disorder case management	18.6	8.9
Bhutan	BTN	Diabetes case management	14.5	7.0
Bhutan	BTN	Malaria case management	59.9	28.8

Country	ISO 3166-1 alpha-3	Intervention Name	Baseline coverage (%)	Ending coverage (%)
Bhutan	BTN	MgSO4 management of pre-eclampsia	36.8	17.7
Bhutan	BTN	Thermal protection	62.4	31.6
Bhutan	BTN	Clean cord care	60.2	30.5
Bhutan	BTN	Clean birth environment	51.8	26.2
Bhutan	BTN	Immediate drying and additional stimulation	57.8	29.3
Bhutan	BTN	Neonatal resuscitation	34.7	17.6
Bhutan	BTN	Antibiotics for preterm or prolonged PROM	47.2	23.9
Bhutan	BTN	Parenteral administration of anti-convulsants	45.1	22.8
Bhutan	BTN	Parenteral administration of uterotonics	56.4	28.6
Bhutan	BTN	Parenteral administration of antibiotics	47.2	23.9
Bhutan	BTN	Assisted vaginal delivery	16.0	8.1
Bhutan	BTN	Manual removal of placenta	23.6	11.9
Bhutan	BTN	Removal of retained products of conception	20.9	10.6
Bhutan	BTN	Cesarean delivery	5.5	2.8
Bhutan	BTN	Blood transfusion	7.9	4.0
Bhutan	BTN	Induction of labor for pregnancies lasting 41+ weeks	1.1	0.6
Bhutan	BTN	Complementary feeding - education only	88.7	51.2
Bhutan	BTN	Complementary feeding - supplementary feeding and education	88.7	51.2
Bhutan	BTN	Vitamin A supplementation	45.0	26.0
Bhutan	BTN	Improved sanitation - Utilization of latrines or toilets	69.3	69.3
Bhutan	BTN	Improved water source	97.2	97.2
Bhutan	BTN	Water connection in the home	96.3	96.3
Bhutan	BTN	Hand washing with soap	79.4	79.4
Bhutan	BTN	Hygienic disposal of children's stools	57.5	57.5
Bhutan	BTN	Injectable antibiotics for neonatal sepsis	63.1	30.3
Bhutan	BTN	ORS - oral rehydration solution	60.9	30.8
Bhutan	BTN	Zinc for treatment of diarrhea	0.8	0.4
Bhutan	BTN	Oral antibiotics for pneumonia	74.2	37.6
Bhutan	BTN	Vitamin A for treatment of measles	45.0	22.8
Bhutan	BTN	BCG vaccine	99.0	47.6
Bhutan	BTN	Polio vaccine	97.0	46.7
Bhutan	BTN	DPT vaccine	97.0	46.7
Bhutan	BTN	H. influenzae type b vaccine	97.0	80.9
Bhutan	BTN	HepB vaccine	97.0	46.7
Bhutan	BTN	Measles vaccine	97.0	46.7
Bhutan	BTN	Global wasting (<-2 SD) rate	6.1	9.1
Bhutan	BTN	Contraceptive prevalence (CPR)	68.35	41.5
Bolivia	BOL	Safe abortion services	0.2	0.1
Bolivia	BOL	TT - Tetanus toxoid vaccination	87.0	41.8
Bolivia	BOL	Syphilis detection and treatment	22.3	10.7

Country	ISO 3166-1 alpha-3	Intervention Name	Baseline coverage (%)	Ending coverage (%)
Bolivia	BOL	Iron supplementation in pregnancy	25.1	12.1
Bolivia	BOL	Hypertensive disorder case management	17.2	8.3
Bolivia	BOL	Diabetes case management	13.4	6.4
Bolivia	BOL	Malaria case management	55.4	26.6
Bolivia	BOL	MgSO4 management of pre-eclampsia	34.0	16.4
Bolivia	BOL	Thermal protection	66.7	33.8
Bolivia	BOL	Clean cord care	64.4	32.6
Bolivia	BOL	Clean birth environment	55.4	28.0
Bolivia	BOL	Immediate drying and additional stimulation	61.8	31.3
Bolivia	BOL	Neonatal resuscitation	37.1	18.8
Bolivia	BOL	Antibiotics for preterm or prolonged PROM	50.5	25.6
Bolivia	BOL	Parenteral administration of anti-convulsants	48.3	24.5
Bolivia	BOL	Parenteral administration of uterotonics	60.3	30.5
Bolivia	BOL	Parenteral administration of antibiotics	50.5	25.6
Bolivia	BOL	Assisted vaginal delivery	17.1	8.7
Bolivia	BOL	Manual removal of placenta	25.2	12.8
Bolivia	BOL	Removal of retained products of conception	22.4	11.3
Bolivia	BOL	Cesarean delivery	5.9	3.0
Bolivia	BOL	Blood transfusion	8.5	4.3
Bolivia	BOL	Induction of labor for pregnancies lasting 41+ weeks	1.2	0.6
Bolivia	BOL	Complementary feeding - education only	70.8	40.9
Bolivia	BOL	Complementary feeding - supplementary feeding and education	70.8	40.9
Bolivia	BOL	Vitamin A supplementation	31.0	17.9
Bolivia	BOL	Improved sanitation - Utilization of latrines or toilets	60.7	60.7
Bolivia	BOL	Improved water source	92.8	92.8
Bolivia	BOL	Water connection in the home	65.9	65.9
Bolivia	BOL	Hygienic disposal of children's stools	16.6	16.6
Bolivia	BOL	Injectable antibiotics for neonatal sepsis	67.5	32.5
Bolivia	BOL	ORS - oral rehydration solution	34.9	17.7
Bolivia	BOL	Antibiotics for treatment of dysentery	43.9	22.2
Bolivia	BOL	Vitamin A for treatment of measles	31.0	15.7
Bolivia	BOL	BCG vaccine	90.0	43.3
Bolivia	BOL	Polio vaccine	83.0	39.9
Bolivia	BOL	DPT vaccine	83.0	39.9
Bolivia	BOL	H. influenzae type b vaccine	83.0	69.2
Bolivia	BOL	HepB vaccine	83.0	39.9
Bolivia	BOL	Pneumococcal vaccine	83.0	69.2
Bolivia	BOL	Rotavirus vaccine	87.0	72.5
Bolivia	BOL	Measles vaccine	89.0	42.8
Bolivia	BOL	Global wasting (<-2 SD) rate	1.4	2.1

Country	ISO 3166-1 alpha-3	Intervention Name	Baseline coverage (%)	Ending coverage (%)
Bolivia	BOL	Contraceptive prevalence (CPR)	66.1	40.2
Bosnia and Herzegovina	BIH	Safe abortion services	100.0	60.8
Bosnia and Herzegovina	BIH	Syphilis detection and treatment	21.5	10.3
Bosnia and Herzegovina	BIH	Hypertensive disorder case management	20.2	9.7
Bosnia and Herzegovina	BIH	Diabetes case management	15.8	7.6
Bosnia and Herzegovina	BIH	Malaria case management	65.3	31.4
Bosnia and Herzegovina	BIH	MgSO4 management of pre-eclampsia	40.0	19.2
Bosnia and Herzegovina	BIH	Thermal protection	98.6	49.9
Bosnia and Herzegovina	BIH	Clean cord care	95.2	48.2
Bosnia and Herzegovina	BIH	Clean birth environment	81.8	41.4
Bosnia and Herzegovina	BIH	Immediate drying and additional stimulation	91.3	46.2
Bosnia and Herzegovina	BIH	Neonatal resuscitation	54.8	27.7
Bosnia and Herzegovina	BIH	Antibiotics for preterm or prolonged PROM	74.6	37.8
Bosnia and Herzegovina	BIH	Parenteral administration of anti-convulsants	71.3	36.1
Bosnia and Herzegovina	BIH	Parenteral administration of uterotonics	89.1	45.1
Bosnia and Herzegovina	BIH	Parenteral administration of antibiotics	74.6	37.8
Bosnia and Herzegovina	BIH	Assisted vaginal delivery	25.2	12.8
Bosnia and Herzegovina	BIH	Manual removal of placenta	37.3	18.9
Bosnia and Herzegovina	BIH	Removal of retained products of conception	33.0	16.7
Bosnia and Herzegovina	BIH	Cesarean delivery	8.7	4.4
Bosnia and Herzegovina	BIH	Blood transfusion	12.6	6.4
Bosnia and Herzegovina	BIH	Induction of labor for pregnancies lasting 41+ weeks	1.8	0.9
Bosnia and Herzegovina	BIH	Improved sanitation - Utilization of latrines or toilets	95.4	95.4
Bosnia and Herzegovina	BIH	Improved water source	96.1	96.1
Bosnia and Herzegovina	BIH	Water connection in the home	91.5	91.5
Bosnia and Herzegovina	BIH	Hand washing with soap	97.0	97.0

Country	ISO 3166-1 alpha-3	Intervention Name	Baseline coverage (%)	Ending coverage (%)
Bosnia and Herzegovina	BIH	Hygienic disposal of children's stools	19.6	19.6
Bosnia and Herzegovina	BIH	Injectable antibiotics for neonatal sepsis	99.7	47.9
Bosnia and Herzegovina	BIH	ORS - oral rehydration solution	36.3	18.4
Bosnia and Herzegovina	BIH	Antibiotics for treatment of dysentery	2.9	1.5
Bosnia and Herzegovina	BIH	Oral antibiotics for pneumonia	86.9	44.0
Bosnia and Herzegovina	BIH	BCG vaccine	95.0	45.7
Bosnia and Herzegovina	BIH	Polio vaccine	73.0	35.1
Bosnia and Herzegovina	BIH	DPT vaccine	73.0	35.1
Bosnia and Herzegovina	BIH	H. influenzae type b vaccine	62.0	51.7
Bosnia and Herzegovina	BIH	HepB vaccine	80.0	38.5
Bosnia and Herzegovina	BIH	Measles vaccine	68.0	32.7
Bosnia and Herzegovina	BIH	Global wasting (<-2 SD) rate	2.4	3.5
Bosnia and Herzegovina	BIH	Contraceptive prevalence (CPR)	48.6	29.5
Botswana	BWA	Safe abortion services	42.4	25.8
Botswana	BWA	TT - Tetanus toxoid vaccination	93.0	44.7
Botswana	BWA	Syphilis detection and treatment	18.1	8.7
Botswana	BWA	Hypertensive disorder case management	17.6	8.5
Botswana	BWA	Diabetes case management	13.7	6.6
Botswana	BWA	Malaria case management	56.8	27.3
Botswana	BWA	MgSO4 management of pre-eclampsia	34.8	16.7
Botswana	BWA	Thermal protection	92.7	46.9
Botswana	BWA	Clean cord care	89.5	45.3
Botswana	BWA	Clean birth environment	76.9	38.9
Botswana	BWA	Immediate drying and additional stimulation	85.9	43.5
Botswana	BWA	Neonatal resuscitation	51.6	26.1
Botswana	BWA	Antibiotics for preterm or prolonged PROM	70.2	35.5
Botswana	BWA	Parenteral administration of anti-convulsants	67.1	34.0
Botswana	BWA	Parenteral administration of uterotonics	83.8	42.4
Botswana	BWA	Parenteral administration of antibiotics	70.2	35.5
Botswana	BWA	Assisted vaginal delivery	23.7	12.0
Botswana	BWA	Manual removal of placenta	35.1	17.8
Botswana	BWA	Removal of retained products of conception	31.1	15.7
Botswana	BWA	Cesarean delivery	8.2	4.2
Botswana	BWA	Blood transfusion	11.8	6.0

Country	ISO 3166-1 alpha-3	Intervention Name	Baseline coverage (%)	Ending coverage (%)
Botswana	BWA	Induction of labor for pregnancies lasting 41+ weeks	1.7	0.9
Botswana	BWA	Complementary feeding - education only	45.5	26.3
Botswana	BWA	Complementary feeding - supplementary feeding and education	45.5	26.3
Botswana	BWA	Vitamin A supplementation	83.0	47.9
Botswana	BWA	Improved sanitation - Utilization of latrines or toilets	77.3	77.3
Botswana	BWA	Improved water source	90.3	90.3
Botswana	BWA	Water connection in the home	81.7	81.7
Botswana	BWA	Injectable antibiotics for neonatal sepsis	93.8	45.1
Botswana	BWA	ORS - oral rehydration solution	42.9	21.7
Botswana	BWA	Oral antibiotics for pneumonia	14.0	7.1
Botswana	BWA	Vitamin A for treatment of measles	83.0	42.0
Botswana	BWA	BCG vaccine	98.0	47.1
Botswana	BWA	Polio vaccine	96.0	46.2
Botswana	BWA	DPT vaccine	95.0	45.7
Botswana	BWA	H. influenzae type b vaccine	95.0	79.2
Botswana	BWA	HepB vaccine	95.0	45.7
Botswana	BWA	Pneumococcal vaccine	91.0	75.9
Botswana	BWA	Rotavirus vaccine	87.0	72.5
Botswana	BWA	Measles vaccine	97.0	46.7
Botswana	BWA	Global wasting (<-2 SD) rate	4.9	7.3
Botswana	BWA	Contraceptive prevalence (CPR)	61.45	37.3
Brazil	BRA	Safe abortion services	0.2	0.1
Brazil	BRA	TT - Tetanus toxoid vaccination	94.0	45.2
Brazil	BRA	Syphilis detection and treatment	22.5	10.8
Brazil	BRA	Hypertensive disorder case management	21.8	10.5
Brazil	BRA	Diabetes case management	17.0	8.2
Brazil	BRA	Malaria case management	70.5	33.9
Brazil	BRA	MgSO4 management of pre-eclampsia	43.2	20.8
Brazil	BRA	Thermal protection	97.0	49.1
Brazil	BRA	Clean cord care	93.6	47.4
Brazil	BRA	Clean birth environment	80.5	40.8
Brazil	BRA	Immediate drying and additional stimulation	89.8	45.5
Brazil	BRA	Neonatal resuscitation	53.9	27.3
Brazil	BRA	Antibiotics for preterm or prolonged PROM	73.4	37.2
Brazil	BRA	Parenteral administration of anti-convulsants	70.1	35.5
Brazil	BRA	Parenteral administration of uterotonics	87.7	44.4
Brazil	BRA	Parenteral administration of antibiotics	73.4	37.2
Brazil	BRA	Assisted vaginal delivery	24.8	12.6
Brazil	BRA	Manual removal of placenta	36.7	18.6
Brazil	BRA	Removal of retained products of conception	32.5	16.5

Country	ISO 3166-1 alpha-3	Intervention Name	Baseline coverage (%)	Ending coverage (%)
Brazil	BRA	Cesarean delivery	8.5	4.3
Brazil	BRA	Blood transfusion	12.4	6.3
Brazil	BRA	Induction of labor for pregnancies lasting 41+ weeks	1.7	0.9
Brazil	BRA	Complementary feeding - education only	56.7	32.7
Brazil	BRA	Complementary feeding - supplementary feeding and education	56.7	32.7
Brazil	BRA	Improved sanitation - Utilization of latrines or toilets	88.3	88.3
Brazil	BRA	Improved water source	98.2	98.2
Brazil	BRA	Water connection in the home	95.5	95.5
Brazil	BRA	Injectable antibiotics for neonatal sepsis	98.1	47.2
Brazil	BRA	ORS - oral rehydration solution	43.6	22.1
Brazil	BRA	Antibiotics for treatment of dysentery	7.9	4.0
Brazil	BRA	Oral antibiotics for pneumonia	49.7	25.2
Brazil	BRA	BCG vaccine	90.0	43.3
Brazil	BRA	Polio vaccine	85.0	40.9
Brazil	BRA	DPT vaccine	83.0	39.9
Brazil	BRA	H. influenzae type b vaccine	83.0	69.2
Brazil	BRA	HepB vaccine	83.0	39.9
Brazil	BRA	Pneumococcal vaccine	84.0	70.0
Brazil	BRA	Rotavirus vaccine	80.0	66.7
Brazil	BRA	Meningococcal A	84.0	40.4
Brazil	BRA	Measles vaccine	84.0	40.4
Brazil	BRA	Global wasting (<-2 SD) rate	2.7	4.0
Brazil	BRA	Contraceptive prevalence (CPR)	79.9	48.5
Burkina Faso	BFA	TT - Tetanus toxoid vaccination	92.0	44.2
Burkina Faso	BFA	IPTp - Intermittent preventive treatment of malaria during pregnancy	47.6	22.9
Burkina Faso	BFA	Syphilis detection and treatment	8.4	4.0
Burkina Faso	BFA	Iron supplementation in pregnancy	50.2	24.1
Burkina Faso	BFA	Hypertensive disorder case management	6.4	3.1
Burkina Faso	BFA	Diabetes case management	0.7	0.3
Burkina Faso	BFA	Malaria case management	22.6	10.9
Burkina Faso	BFA	MgSO4 management of pre-eclampsia	6.9	3.3
Burkina Faso	BFA	Thermal protection	65.6	33.2
Burkina Faso	BFA	Clean cord care	65.5	33.2
Burkina Faso	BFA	Clean birth environment	64.0	32.4
Burkina Faso	BFA	Immediate drying and additional stimulation	63.9	32.3
Burkina Faso	BFA	Neonatal resuscitation	13.0	6.6
Burkina Faso	BFA	Antibiotics for preterm or prolonged PROM	52.6	26.6
Burkina Faso	BFA	Parenteral administration of anti-convulsants	15.9	8.0
Burkina Faso	BFA	Parenteral administration of uterotonics	64.1	32.5
Burkina Faso	BFA	Parenteral administration of antibiotics	52.6	26.6

**Country**	**ISO 3166-1 alpha-3**	**Intervention Name**	**Baseline coverage (%)**	**Ending coverage (%)**
Burkina Faso	BFA	Assisted vaginal delivery	7.0	3.5
Burkina Faso	BFA	Manual removal of placenta	49.1	24.9
Burkina Faso	BFA	Removal of retained products of conception	11.1	5.6
Burkina Faso	BFA	Cesarean delivery	0.8	0.4
Burkina Faso	BFA	Blood transfusion	5.8	2.9
Burkina Faso	BFA	Induction of labor for pregnancies lasting 41+ weeks	0.9	0.5
Burkina Faso	BFA	Complementary feeding - education only	5.9	3.4
Burkina Faso	BFA	Complementary feeding - supplementary feeding and education	5.9	3.4
Burkina Faso	BFA	Improved sanitation - Utilization of latrines or toilets	19.4	19.4
Burkina Faso	BFA	Improved water source	47.9	47.9
Burkina Faso	BFA	Water connection in the home	12.2	12.2
Burkina Faso	BFA	Hand washing with soap	12.8	12.8
Burkina Faso	BFA	Hygienic disposal of children's stools	20.8	20.8
Burkina Faso	BFA	ITN/IRS - Households protected from malaria	75.3	43.5
Burkina Faso	BFA	Injectable antibiotics for neonatal sepsis	66.3	31.9
Burkina Faso	BFA	ORS - oral rehydration solution	21.2	10.7
Burkina Faso	BFA	Antibiotics for treatment of dysentery	34.5	17.5
Burkina Faso	BFA	Zinc for treatment of diarrhea	0.4	0.2
Burkina Faso	BFA	Oral antibiotics for pneumonia	55.5	28.1
Burkina Faso	BFA	ACTs- Artemisinin compounds for treatment of malaria	5.5	2.8
Burkina Faso	BFA	SAM - treatment for severe acute malnutrition	26.1	13.2
Burkina Faso	BFA	BCG vaccine	98.0	47.1
Burkina Faso	BFA	Polio vaccine	91.0	43.8
Burkina Faso	BFA	DPT vaccine	91.0	43.8
Burkina Faso	BFA	H. influenzae type b vaccine	91.0	75.9
Burkina Faso	BFA	HepB vaccine	91.0	43.8
Burkina Faso	BFA	Pneumococcal vaccine	91.0	75.9
Burkina Faso	BFA	Rotavirus vaccine	91.0	75.9
Burkina Faso	BFA	Meningococcal A	87.0	41.8
Burkina Faso	BFA	Measles vaccine	88.0	42.3
Burkina Faso	BFA	Global wasting (<-2 SD) rate	15.5	23.3
Burkina Faso	BFA	Contraceptive prevalence (CPR)	27.95	17.0
Burundi	BDI	Safe abortion services	3.3	2.0
Burundi	BDI	TT - Tetanus toxoid vaccination	87.0	41.8
Burundi	BDI	IPTp - Intermittent preventive treatment of malaria during pregnancy	20.7	10.0
Burundi	BDI	Syphilis detection and treatment	24.5	11.8
Burundi	BDI	Iron supplementation in pregnancy	1.4	0.7
Burundi	BDI	Hypertensive disorder case management	12.2	5.9
Burundi	BDI	Diabetes case management	9.5	4.6
Burundi	BDI	Malaria case management	39.3	18.9

Country	ISO 3166-1 alpha-3	Intervention Name	Baseline coverage (%)	Ending coverage (%)
Burundi	BDI	MgSO4 management of pre-eclampsia	24.1	11.6
Burundi	BDI	Thermal protection	83.0	42.0
Burundi	BDI	Clean cord care	80.1	40.6
Burundi	BDI	Clean birth environment	68.8	34.8
Burundi	BDI	Immediate drying and additional stimulation	76.9	38.9
Burundi	BDI	Neonatal resuscitation	46.2	23.4
Burundi	BDI	Antibiotics for preterm or prolonged PROM	62.8	31.8
Burundi	BDI	Parenteral administration of anti-convulsants	60.0	30.4
Burundi	BDI	Parenteral administration of uterotonics	75.0	38.0
Burundi	BDI	Parenteral administration of antibiotics	62.8	31.8
Burundi	BDI	Assisted vaginal delivery	21.2	10.7
Burundi	BDI	Manual removal of placenta	31.4	15.9
Burundi	BDI	Removal of retained products of conception	27.8	14.1
Burundi	BDI	Cesarean delivery	7.3	3.7
Burundi	BDI	Blood transfusion	10.6	5.4
Burundi	BDI	Induction of labor for pregnancies lasting 41+ weeks	1.5	0.8
Burundi	BDI	Complementary feeding - education only	19.3	11.1
Burundi	BDI	Complementary feeding - supplementary feeding and education	19.3	11.1
Burundi	BDI	Vitamin A supplementation	79.0	45.6
Burundi	BDI	Improved sanitation - Utilization of latrines or toilets	45.8	45.8
Burundi	BDI	Improved water source	60.8	60.8
Burundi	BDI	Water connection in the home	21.6	21.6
Burundi	BDI	Hand washing with soap	5.8	5.8
Burundi	BDI	Hygienic disposal of children's stools	73.7	73.7
Burundi	BDI	ITN/IRS - Households protected from malaria	46.8	27.0
Burundi	BDI	Injectable antibiotics for neonatal sepsis	83.9	40.4
Burundi	BDI	ORS - oral rehydration solution	35.6	18.0
Burundi	BDI	Antibiotics for treatment of dysentery	32.9	16.7
Burundi	BDI	Zinc for treatment of diarrhea	15.0	7.6
Burundi	BDI	Oral antibiotics for pneumonia	58.5	29.6
Burundi	BDI	Vitamin A for treatment of measles	79.0	40.0
Burundi	BDI	ACTs- Artemisinin compounds for treatment of malaria	5.0	2.5
Burundi	BDI	SAM - treatment for severe acute malnutrition	6.9	3.5
Burundi	BDI	BCG vaccine	91.0	43.8
Burundi	BDI	Polio vaccine	90.0	43.3
Burundi	BDI	DPT vaccine	90.0	43.3
Burundi	BDI	H. influenzae type b vaccine	90.0	75.1
Burundi	BDI	HepB vaccine	90.0	43.3
Burundi	BDI	Pneumococcal vaccine	90.0	75.1
Burundi	BDI	Rotavirus vaccine	92.0	76.7

Country	ISO 3166-1 alpha-3	Intervention Name	Baseline coverage (%)	Ending coverage (%)
Burundi	BDI	Measles vaccine	88.0	42.3
Burundi	BDI	Global wasting (<-2 SD) rate	5.0	7.5
Burundi	BDI	Contraceptive prevalence (CPR)	31.85	19.3
Cambodia	KHM	Safe abortion services	39.0	23.7
Cambodia	KHM	TT - Tetanus toxoid vaccination	93.0	44.7
Cambodia	KHM	Syphilis detection and treatment	23.7	11.4
Cambodia	KHM	Iron supplementation in pregnancy	75.5	36.3
Cambodia	KHM	Hypertensive disorder case management	18.2	8.8
Cambodia	KHM	Diabetes case management	14.2	6.8
Cambodia	KHM	Malaria case management	58.7	28.2
Cambodia	KHM	MgSO4 management of pre-eclampsia	36.0	17.3
Cambodia	KHM	Thermal protection	82.3	41.7
Cambodia	KHM	Clean cord care	79.4	40.2
Cambodia	KHM	Clean birth environment	68.3	34.6
Cambodia	KHM	Immediate drying and additional stimulation	76.2	38.6
Cambodia	KHM	Neonatal resuscitation	45.8	23.2
Cambodia	KHM	Antibiotics for preterm or prolonged PROM	62.3	31.5
Cambodia	KHM	Parenteral administration of anti-convulsants	59.5	30.1
Cambodia	KHM	Parenteral administration of uterotonics	74.4	37.7
Cambodia	KHM	Parenteral administration of antibiotics	62.3	31.5
Cambodia	KHM	Assisted vaginal delivery	21.0	10.6
Cambodia	KHM	Manual removal of placenta	31.1	15.7
Cambodia	KHM	Removal of retained products of conception	27.6	14.0
Cambodia	KHM	Cesarean delivery	7.2	3.6
Cambodia	KHM	Blood transfusion	10.5	5.3
Cambodia	KHM	Induction of labor for pregnancies lasting 41+ weeks	1.5	0.8
Cambodia	KHM	Complementary feeding - education only	47.7	27.5
Cambodia	KHM	Complementary feeding - supplementary feeding and education	47.7	27.5
Cambodia	KHM	Vitamin A supplementation	73.0	42.1
Cambodia	KHM	Improved sanitation - Utilization of latrines or toilets	59.2	59.2
Cambodia	KHM	Improved water source	78.5	78.5
Cambodia	KHM	Water connection in the home	20.5	20.5
Cambodia	KHM	Hand washing with soap	67.9	67.9
Cambodia	KHM	Hygienic disposal of children's stools	22.6	22.6
Cambodia	KHM	ITN/IRS - Households protected from malaria	4.5	2.6
Cambodia	KHM	Injectable antibiotics for neonatal sepsis	83.2	40.0
Cambodia	KHM	ORS - oral rehydration solution	35.2	17.8
Cambodia	KHM	Antibiotics for treatment of dysentery	7.0	3.5
Cambodia	KHM	Zinc for treatment of diarrhea	5.4	2.7
Cambodia	KHM	Oral antibiotics for pneumonia	68.8	34.8

Country	ISO 3166-1 alpha-3	Intervention Name	Baseline coverage (%)	Ending coverage (%)
Cambodia	KHM	Vitamin A for treatment of measles	73.0	37.0
Cambodia	KHM	SAM - treatment for severe acute malnutrition	2.5	1.3
Cambodia	KHM	BCG vaccine	93.0	44.7
Cambodia	KHM	Polio vaccine	90.0	43.3
Cambodia	KHM	DPT vaccine	92.0	44.2
Cambodia	KHM	H. influenzae type b vaccine	92.0	76.7
Cambodia	KHM	HepB vaccine	92.0	44.2
Cambodia	KHM	Pneumococcal vaccine	84.0	70.0
Cambodia	KHM	Measles vaccine	84.0	40.4
Cambodia	KHM	Global wasting (<-2 SD) rate	9.9	14.8
Cambodia	KHM	Contraceptive prevalence (CPR)	61.2	37.2
Cameroon	CMR	TT - Tetanus toxoid vaccination	85.0	40.9
Cameroon	CMR	IPTp - Intermittent preventive treatment of malaria during pregnancy	44.3	21.3
Cameroon	CMR	Syphilis detection and treatment	20.5	9.9
Cameroon	CMR	Iron supplementation in pregnancy	54.1	26.0
Cameroon	CMR	Hypertensive disorder case management	14.1	6.8
Cameroon	CMR	Diabetes case management	11.0	5.3
Cameroon	CMR	Malaria case management	45.6	21.9
Cameroon	CMR	MgSO4 management of pre-eclampsia	28.0	13.5
Cameroon	CMR	Thermal protection	60.5	30.6
Cameroon	CMR	Clean cord care	58.5	29.6
Cameroon	CMR	Clean birth environment	50.2	25.4
Cameroon	CMR	Immediate drying and additional stimulation	56.1	28.4
Cameroon	CMR	Neonatal resuscitation	33.7	17.1
Cameroon	CMR	Antibiotics for preterm or prolonged PROM	45.8	23.2
Cameroon	CMR	Parenteral administration of anti-convulsants	43.8	22.2
Cameroon	CMR	Parenteral administration of uterotonics	54.7	27.7
Cameroon	CMR	Parenteral administration of antibiotics	45.8	23.2
Cameroon	CMR	Assisted vaginal delivery	15.5	7.8
Cameroon	CMR	Manual removal of placenta	22.9	11.6
Cameroon	CMR	Removal of retained products of conception	20.3	10.3
Cameroon	CMR	Cesarean delivery	5.3	2.7
Cameroon	CMR	Blood transfusion	7.7	3.9
Cameroon	CMR	Induction of labor for pregnancies lasting 41+ weeks	1.1	0.6
Cameroon	CMR	Complementary feeding - education only	32.9	19.0
Cameroon	CMR	Complementary feeding - supplementary feeding and education	32.9	19.0
Cameroon	CMR	Vitamin A supplementation	9.0	5.2
Cameroon	CMR	Improved sanitation - Utilization of latrines or toilets	39.1	39.1
Cameroon	CMR	Improved water source	60.4	60.4
Cameroon	CMR	Water connection in the home	24.0	24.0

Country	ISO 3166-1 alpha-3	Intervention Name	Baseline coverage (%)	Ending coverage (%)
Cameroon	CMR	Hand washing with soap	14.9	14.9
Cameroon	CMR	Hygienic disposal of children's stools	70.6	70.6
Cameroon	CMR	ITN/IRS - Households protected from malaria	70.9	40.9
Cameroon	CMR	Injectable antibiotics for neonatal sepsis	61.3	29.5
Cameroon	CMR	ORS - oral rehydration solution	15.8	8.0
Cameroon	CMR	Antibiotics for treatment of dysentery	15.0	7.6
Cameroon	CMR	Zinc for treatment of diarrhea	12.0	6.1
Cameroon	CMR	Oral antibiotics for pneumonia	26.7	13.5
Cameroon	CMR	Vitamin A for treatment of measles	9.0	4.6
Cameroon	CMR	ACTs- Artemisinin compounds for treatment of malaria	3.6	1.8
Cameroon	CMR	SAM - treatment for severe acute malnutrition	11.3	5.7
Cameroon	CMR	BCG vaccine	88.0	42.3
Cameroon	CMR	Polio vaccine	78.0	37.5
Cameroon	CMR	DPT vaccine	79.0	38.0
Cameroon	CMR	H. influenzae type b vaccine	79.0	65.9
Cameroon	CMR	HepB vaccine	79.0	38.0
Cameroon	CMR	Pneumococcal vaccine	79.0	65.9
Cameroon	CMR	Rotavirus vaccine	78.0	65.0
Cameroon	CMR	Measles vaccine	71.0	34.1
Cameroon	CMR	Global wasting (<-2 SD) rate	5.0	7.5
Cameroon	CMR	Contraceptive prevalence (CPR)	37.95	23.1
Cape Verde	CPV	TT - Tetanus toxoid vaccination	92.0	44.2
Cape Verde	CPV	Syphilis detection and treatment	22.3	10.7
Cape Verde	CPV	Iron supplementation in pregnancy	10.2	4.9
Cape Verde	CPV	Hypertensive disorder case management	17.4	8.4
Cape Verde	CPV	Diabetes case management	13.5	6.5
Cape Verde	CPV	Malaria case management	56.0	26.9
Cape Verde	CPV	MgSO4 management of pre-eclampsia	34.4	16.5
Cape Verde	CPV	Thermal protection	76.6	38.8
Cape Verde	CPV	Clean cord care	74.0	37.5
Cape Verde	CPV	Clean birth environment	63.6	32.2
Cape Verde	CPV	Immediate drying and additional stimulation	71.0	35.9
Cape Verde	CPV	Neonatal resuscitation	42.6	21.6
Cape Verde	CPV	Antibiotics for preterm or prolonged PROM	58.0	29.4
Cape Verde	CPV	Parenteral administration of anti-convulsants	55.4	28.0
Cape Verde	CPV	Parenteral administration of uterotonics	69.3	35.1
Cape Verde	CPV	Parenteral administration of antibiotics	58.0	29.4
Cape Verde	CPV	Assisted vaginal delivery	19.6	9.9
Cape Verde	CPV	Manual removal of placenta	29.0	14.7
Cape Verde	CPV	Removal of retained products of conception	25.7	13.0
Cape Verde	CPV	Cesarean delivery	6.7	3.4

Country	ISO 3166-1 alpha-3	Intervention Name	Baseline coverage (%)	Ending coverage (%)
Cape Verde	CPV	Blood transfusion	9.8	5.0
Cape Verde	CPV	Induction of labor for pregnancies lasting 41+ weeks	1.4	0.7
Cape Verde	CPV	Improved sanitation - Utilization of latrines or toilets	73.9	73.9
Cape Verde	CPV	Improved water source	87.1	87.1
Cape Verde	CPV	Water connection in the home	77.1	77.1
Cape Verde	CPV	Injectable antibiotics for neonatal sepsis	77.5	37.3
Cape Verde	CPV	ORS - oral rehydration solution	99.8	50.5
Cape Verde	CPV	BCG vaccine	96.0	46.2
Cape Verde	CPV	Polio vaccine	98.0	47.1
Cape Verde	CPV	DPT vaccine	98.0	47.1
Cape Verde	CPV	H. influenzae type b vaccine	99.0	82.6
Cape Verde	CPV	HepB vaccine	99.0	47.6
Cape Verde	CPV	Measles vaccine	99.0	47.6
Cape Verde	CPV	Global wasting (<-2 SD) rate	10.0	15.0
Cape Verde	CPV	Contraceptive prevalence (CPR)	66.5	40.4
Central African Republic	CAF	TT - Tetanus toxoid vaccination	60.0	28.9
Central African Republic	CAF	IPTp - Intermittent preventive treatment of malaria during pregnancy	26.4	12.7
Central African Republic	CAF	Syphilis detection and treatment	16.9	8.1
Central African Republic	CAF	Hypertensive disorder case management	9.2	4.4
Central African Republic	CAF	Diabetes case management	7.1	3.4
Central African Republic	CAF	Malaria case management	29.6	14.2
Central African Republic	CAF	MgSO4 management of pre-eclampsia	18.1	8.7
Central African Republic	CAF	Thermal protection	51.5	26.1
Central African Republic	CAF	Clean cord care	49.7	25.2
Central African Republic	CAF	Clean birth environment	42.7	21.6
Central African Republic	CAF	Immediate drying and additional stimulation	47.7	24.1
Central African Republic	CAF	Neonatal resuscitation	28.6	14.5
Central African Republic	CAF	Antibiotics for preterm or prolonged PROM	39.0	19.7
Central African Republic	CAF	Parenteral administration of anti-convulsants	37.2	18.8
Central African Republic	CAF	Parenteral administration of uterotonics	46.5	23.5
Central African Republic	CAF	Parenteral administration of antibiotics	39.0	19.7

**Country**	**ISO 3166-1 alpha-3**	**Intervention Name**	**Baseline coverage (%)**	**Ending coverage (%)**
Central African Republic	CAF	Assisted vaginal delivery	13.2	6.7
Central African Republic	CAF	Manual removal of placenta	19.5	9.9
Central African Republic	CAF	Removal of retained products of conception	17.3	8.8
Central African Republic	CAF	Cesarean delivery	4.5	2.3
Central African Republic	CAF	Blood transfusion	6.6	3.3
Central African Republic	CAF	Induction of labor for pregnancies lasting 41+ weeks	0.9	0.5
Central African Republic	CAF	Complementary feeding - education only	33.9	19.6
Central African Republic	CAF	Complementary feeding - supplementary feeding and education	33.9	19.6
Central African Republic	CAF	Improved sanitation - Utilization of latrines or toilets	25.3	25.3
Central African Republic	CAF	Improved water source	46.3	46.3
Central African Republic	CAF	Water connection in the home	8.4	8.4
Central African Republic	CAF	Hand washing with soap	15.4	15.4
Central African Republic	CAF	Hygienic disposal of children's stools	50.2	50.2
Central African Republic	CAF	ITN/IRS - Households protected from malaria	47.2	27.2
Central African Republic	CAF	Injectable antibiotics for neonatal sepsis	52.1	25.1
Central African Republic	CAF	ORS - oral rehydration solution	15.6	7.9
Central African Republic	CAF	Antibiotics for treatment of dysentery	4.4	2.2
Central African Republic	CAF	Zinc for treatment of diarrhea	0.5	0.3
Central African Republic	CAF	Oral antibiotics for pneumonia	29.8	15.1
Central African Republic	CAF	ACTs- Artemisinin compounds for treatment of malaria	2.3	1.2
Central African Republic	CAF	SAM - treatment for severe acute malnutrition	17.6	8.9
Central African Republic	CAF	BCG vaccine	74.0	35.6
Central African Republic	CAF	Polio vaccine	47.0	22.6
Central African Republic	CAF	DPT vaccine	47.0	22.6
Central African Republic	CAF	H. influenzae type b vaccine	47.0	39.2
Central African Republic	CAF	HepB vaccine	47.0	22.6

Country	ISO 3166-1 alpha-3	Intervention Name	Baseline coverage (%)	Ending coverage (%)
Central African Republic	CAF	Pneumococcal vaccine	47.0	39.2
Central African Republic	CAF	Meningococcal A	66.0	31.7
Central African Republic	CAF	Measles vaccine	49.0	23.6
Central African Republic	CAF	Global wasting (<-2 SD) rate	7.3	10.9
Central African Republic	CAF	Contraceptive prevalence (CPR)	23.5	14.3
Chad	TCD	TT - Tetanus toxoid vaccination	78.0	37.5
Chad	TCD	IPTp - Intermittent preventive treatment of malaria during pregnancy	18.1	8.7
Chad	TCD	Syphilis detection and treatment	13.7	6.6
Chad	TCD	Iron supplementation in pregnancy	11.0	5.3
Chad	TCD	Hypertensive disorder case management	7.5	3.6
Chad	TCD	Diabetes case management	5.9	2.8
Chad	TCD	Malaria case management	24.3	11.7
Chad	TCD	MgSO4 management of pre-eclampsia	14.9	7.2
Chad	TCD	Thermal protection	21.6	10.9
Chad	TCD	Clean cord care	20.9	10.6
Chad	TCD	Clean birth environment	17.9	9.1
Chad	TCD	Immediate drying and additional stimulation	20.0	10.1
Chad	TCD	Neonatal resuscitation	12.0	6.1
Chad	TCD	Antibiotics for preterm or prolonged PROM	16.4	8.3
Chad	TCD	Parenteral administration of anti-convulsants	15.6	7.9
Chad	TCD	Parenteral administration of uterotonics	19.5	9.9
Chad	TCD	Parenteral administration of antibiotics	16.4	8.3
Chad	TCD	Assisted vaginal delivery	5.5	2.8
Chad	TCD	Manual removal of placenta	8.2	4.2
Chad	TCD	Removal of retained products of conception	7.2	3.6
Chad	TCD	Cesarean delivery	1.9	1.0
Chad	TCD	Blood transfusion	2.8	1.4
Chad	TCD	Induction of labor for pregnancies lasting 41+ weeks	0.4	0.2
Chad	TCD	Complementary feeding - education only	10.4	6.0
Chad	TCD	Complementary feeding - supplementary feeding and education	10.4	6.0
Chad	TCD	Vitamin A supplementation	67.0	38.7
Chad	TCD	Improved sanitation - Utilization of latrines or toilets	8.3	8.3
Chad	TCD	Improved water source	38.7	38.7
Chad	TCD	Water connection in the home	7.4	7.4
Chad	TCD	Hand washing with soap	23.7	23.7
Chad	TCD	Hygienic disposal of children's stools	18.9	18.9
Chad	TCD	ITN/IRS - Households protected from malaria	77.3	44.6

Country	ISO 3166-1 alpha-3	Intervention Name	Baseline coverage (%)	Ending coverage (%)
Chad	TCD	Injectable antibiotics for neonatal sepsis	21.9	10.5
Chad	TCD	ORS - oral rehydration solution	20.4	10.3
Chad	TCD	Antibiotics for treatment of dysentery	15.9	8.0
Chad	TCD	Zinc for treatment of diarrhea	1.3	0.7
Chad	TCD	Oral antibiotics for pneumonia	25.8	13.1
Chad	TCD	Vitamin A for treatment of measles	67.0	33.9
Chad	TCD	ACTs- Artemisinin compounds for treatment of malaria	1.7	0.9
Chad	TCD	SAM - treatment for severe acute malnutrition	18.3	9.3
Chad	TCD	BCG vaccine	59.0	28.4
Chad	TCD	Polio vaccine	44.0	21.2
Chad	TCD	DPT vaccine	41.0	19.7
Chad	TCD	H. influenzae type b vaccine	41.0	34.2
Chad	TCD	HepB vaccine	41.0	19.7
Chad	TCD	Meningococcal A	70.0	33.7
Chad	TCD	Measles vaccine	37.0	17.8
Chad	TCD	Global wasting (<-2 SD) rate	13.5	20.3
Chad	TCD	Contraceptive prevalence (CPR)	7.25	4.4
Colombia	COL	Safe abortion services	0.2	0.1
Colombia	COL	TT - Tetanus toxoid vaccination	95.0	45.7
Colombia	COL	Syphilis detection and treatment	24.0	11.5
Colombia	COL	Iron supplementation in pregnancy	80.7	38.8
Colombia	COL	Hypertensive disorder case management	21.5	10.3
Colombia	COL	Diabetes case management	16.8	8.1
Colombia	COL	Malaria case management	69.5	33.4
Colombia	COL	MgSO4 management of pre-eclampsia	42.6	20.5
Colombia	COL	Thermal protection	95.2	48.2
Colombia	COL	Clean cord care	91.9	46.5
Colombia	COL	Clean birth environment	79.0	40.0
Colombia	COL	Immediate drying and additional stimulation	88.2	44.7
Colombia	COL	Neonatal resuscitation	53.0	26.8
Colombia	COL	Antibiotics for preterm or prolonged PROM	72.1	36.5
Colombia	COL	Parenteral administration of anti-convulsants	68.9	34.9
Colombia	COL	Parenteral administration of uterotonics	86.1	43.6
Colombia	COL	Parenteral administration of antibiotics	72.1	36.5
Colombia	COL	Assisted vaginal delivery	24.3	12.3
Colombia	COL	Manual removal of placenta	36.0	18.2
Colombia	COL	Removal of retained products of conception	31.9	16.1
Colombia	COL	Cesarean delivery	8.4	4.3
Colombia	COL	Blood transfusion	12.1	6.1
Colombia	COL	Induction of labor for pregnancies lasting 41+ weeks	1.7	0.9
Colombia	COL	Complementary feeding - education only	72.3	41.7

Country	ISO 3166-1 alpha-3	Intervention Name	Baseline coverage (%)	Ending coverage (%)
Colombia	COL	Complementary feeding - supplementary feeding and education	72.3	41.7
Colombia	COL	Improved sanitation - Utilization of latrines or toilets	89.6	89.6
Colombia	COL	Improved water source	97.3	97.3
Colombia	COL	Water connection in the home	86.3	86.3
Colombia	COL	Hygienic disposal of children's stools	14.4	14.4
Colombia	COL	Injectable antibiotics for neonatal sepsis	96.3	46.3
Colombia	COL	ORS - oral rehydration solution	54.1	27.4
Colombia	COL	Antibiotics for treatment of dysentery	5.8	2.9
Colombia	COL	SAM - treatment for severe acute malnutrition	0.1	0.1
Colombia	COL	BCG vaccine	89.0	42.8
Colombia	COL	Polio vaccine	92.0	44.2
Colombia	COL	DPT vaccine	92.0	44.2
Colombia	COL	H. influenzae type b vaccine	92.0	76.7
Colombia	COL	HepB vaccine	92.0	44.2
Colombia	COL	Pneumococcal vaccine	94.0	78.4
Colombia	COL	Rotavirus vaccine	90.0	75.1
Colombia	COL	Measles vaccine	95.0	45.7
Colombia	COL	Global wasting (<-2 SD) rate	0.9	1.4
Colombia	COL	Contraceptive prevalence (CPR)	81.4	49.5
Comoros	COM	Safe abortion services	3.3	2.0
Comoros	COM	TT - Tetanus toxoid vaccination	85.0	40.9
Comoros	COM	IPTp - Intermittent preventive treatment of malaria during pregnancy	30.8	14.8
Comoros	COM	Syphilis detection and treatment	22.8	11.0
Comoros	COM	Iron supplementation in pregnancy	12.6	6.1
Comoros	COM	Hypertensive disorder case management	11.8	5.7
Comoros	COM	Diabetes case management	9.2	4.4
Comoros	COM	Malaria case management	38.2	18.4
Comoros	COM	MgSO4 management of pre-eclampsia	23.4	11.3
Comoros	COM	Thermal protection	75.3	38.1
Comoros	COM	Clean cord care	72.7	36.8
Comoros	COM	Clean birth environment	62.4	31.6
Comoros	COM	Immediate drying and additional stimulation	69.7	35.3
Comoros	COM	Neonatal resuscitation	41.9	21.2
Comoros	COM	Antibiotics for preterm or prolonged PROM	57.0	28.9
Comoros	COM	Parenteral administration of anti-convulsants	54.4	27.5
Comoros	COM	Parenteral administration of uterotonics	68.0	34.4
Comoros	COM	Parenteral administration of antibiotics	57.0	28.9
Comoros	COM	Assisted vaginal delivery	19.2	9.7
Comoros	COM	Manual removal of placenta	28.5	14.4
Comoros	COM	Removal of retained products of conception	25.2	12.8

Country	ISO 3166-1 alpha-3	Intervention Name	Baseline coverage (%)	Ending coverage (%)
Comoros	COM	Cesarean delivery	6.6	3.3
Comoros	COM	Blood transfusion	9.6	4.9
Comoros	COM	Induction of labor for pregnancies lasting 41+ weeks	1.3	0.7
Comoros	COM	Complementary feeding - education only	25.2	14.5
Comoros	COM	Complementary feeding - supplementary feeding and education	25.2	14.5
Comoros	COM	Vitamin A supplementation	21.0	12.1
Comoros	COM	Improved sanitation - Utilization of latrines or toilets	35.9	35.9
Comoros	COM	Improved water source	80.2	80.2
Comoros	COM	Water connection in the home	47.6	47.6
Comoros	COM	Hand washing with soap	20.0	20.0
Comoros	COM	Hygienic disposal of children's stools	60.4	60.4
Comoros	COM	ITN/IRS - Households protected from malaria	60.6	35.0
Comoros	COM	Injectable antibiotics for neonatal sepsis	76.1	36.6
Comoros	COM	ORS - oral rehydration solution	37.5	19.0
Comoros	COM	Antibiotics for treatment of dysentery	19.4	9.8
Comoros	COM	Zinc for treatment of diarrhea	0.4	0.2
Comoros	COM	Oral antibiotics for pneumonia	38.1	19.3
Comoros	COM	Vitamin A for treatment of measles	21.0	10.6
Comoros	COM	ACTs- Artemisinin compounds for treatment of malaria	3.9	2.0
Comoros	COM	SAM - treatment for severe acute malnutrition	25.0	12.7
Comoros	COM	BCG vaccine	94.0	45.2
Comoros	COM	Polio vaccine	94.0	45.2
Comoros	COM	DPT vaccine	91.0	43.8
Comoros	COM	H. influenzae type b vaccine	91.0	75.9
Comoros	COM	HepB vaccine	91.0	43.8
Comoros	COM	Measles vaccine	90.0	43.3
Comoros	COM	Global wasting (<-2 SD) rate	11.2	16.8
Comoros	COM	Contraceptive prevalence (CPR)	26.4	16.0
Congo	COG	TT - Tetanus toxoid vaccination	85.0	40.9
Congo	COG	IPTp - Intermittent preventive treatment of malaria during pregnancy	23.2	11.2
Congo	COG	Syphilis detection and treatment	23.0	11.1
Congo	COG	Iron supplementation in pregnancy	42.9	20.6
Congo	COG	Hypertensive disorder case management	19.0	9.1
Congo	COG	Diabetes case management	14.8	7.1
Congo	COG	Malaria case management	61.2	29.4
Congo	COG	MgSO4 management of pre-eclampsia	37.5	18.0
Congo	COG	Thermal protection	90.4	45.8
Congo	COG	Clean cord care	87.3	44.2
Congo	COG	Clean birth environment	75.0	38.0
Congo	COG	Immediate drying and additional stimulation	83.8	42.4

Country	ISO 3166-1 alpha-3	Intervention Name	Baseline coverage (%)	Ending coverage (%)
Congo	COG	Neonatal resuscitation	50.3	25.5
Congo	COG	Antibiotics for preterm or prolonged PROM	68.5	34.7
Congo	COG	Parenteral administration of anti-convulsants	65.4	33.1
Congo	COG	Parenteral administration of uterotonics	81.8	41.4
Congo	COG	Parenteral administration of antibiotics	68.5	34.7
Congo	COG	Assisted vaginal delivery	23.1	11.7
Congo	COG	Manual removal of placenta	34.2	17.3
Congo	COG	Removal of retained products of conception	30.3	15.3
Congo	COG	Cesarean delivery	8.0	4.1
Congo	COG	Blood transfusion	11.5	5.8
Congo	COG	Induction of labor for pregnancies lasting 41+ weeks	1.6	0.8
Congo	COG	Complementary feeding - education only	23.9	13.8
Congo	COG	Complementary feeding - supplementary feeding and education	23.9	13.8
Congo	COG	Vitamin A supplementation	12.0	6.9
Congo	COG	Improved sanitation - Utilization of latrines or toilets	20.2	20.2
Congo	COG	Improved water source	73.2	73.2
Congo	COG	Water connection in the home	38.0	38.0
Congo	COG	Hand washing with soap	49.2	49.2
Congo	COG	Hygienic disposal of children's stools	62.8	62.8
Congo	COG	ITN/IRS - Households protected from malaria	66.1	38.1
Congo	COG	Injectable antibiotics for neonatal sepsis	91.5	44.0
Congo	COG	ORS - oral rehydration solution	26.9	13.6
Congo	COG	Antibiotics for treatment of dysentery	4.9	2.5
Congo	COG	Zinc for treatment of diarrhea	16.0	8.1
Congo	COG	Oral antibiotics for pneumonia	28.2	14.3
Congo	COG	Vitamin A for treatment of measles	12.0	6.1
Congo	COG	ACTs- Artemisinin compounds for treatment of malaria	8.8	4.5
Congo	COG	SAM - treatment for severe acute malnutrition	1.0	0.5
Congo	COG	BCG vaccine	81.0	39.0
Congo	COG	Polio vaccine	75.0	36.1
Congo	COG	DPT vaccine	75.0	36.1
Congo	COG	H. influenzae type b vaccine	75.0	62.5
Congo	COG	HepB vaccine	75.0	36.1
Congo	COG	Pneumococcal vaccine	73.0	60.9
Congo	COG	Rotavirus vaccine	72.0	60.0
Congo	COG	Meningococcal A	83.0	39.9
Congo	COG	Measles vaccine	75.0	36.1
Congo	COG	Global wasting (<-2 SD) rate	8.1	12.2
Congo	COG	Contraceptive prevalence (CPR)	38.6	23.4
Costa Rica	CRI	Safe abortion services	0.6	0.4

Country	ISO 3166-1 alpha-3	Intervention Name	Baseline coverage (%)	Ending coverage (%)
Costa Rica	CRI	Syphilis detection and treatment	24.2	11.6
Costa Rica	CRI	Hypertensive disorder case management	21.7	10.4
Costa Rica	CRI	Diabetes case management	16.9	8.1
Costa Rica	CRI	Malaria case management	69.9	33.6
Costa Rica	CRI	MgSO4 management of pre-eclampsia	42.9	20.6
Costa Rica	CRI	Thermal protection	97.0	49.1
Costa Rica	CRI	Clean cord care	93.6	47.4
Costa Rica	CRI	Clean birth environment	80.5	40.8
Costa Rica	CRI	Immediate drying and additional stimulation	89.8	45.5
Costa Rica	CRI	Neonatal resuscitation	53.9	27.3
Costa Rica	CRI	Antibiotics for preterm or prolonged PROM	73.4	37.2
Costa Rica	CRI	Parenteral administration of anti-convulsants	70.1	35.5
Costa Rica	CRI	Parenteral administration of uterotonics	87.7	44.4
Costa Rica	CRI	Parenteral administration of antibiotics	73.4	37.2
Costa Rica	CRI	Assisted vaginal delivery	24.8	12.6
Costa Rica	CRI	Manual removal of placenta	36.7	18.6
Costa Rica	CRI	Removal of retained products of conception	32.5	16.5
Costa Rica	CRI	Cesarean delivery	8.5	4.3
Costa Rica	CRI	Blood transfusion	12.4	6.3
Costa Rica	CRI	Induction of labor for pregnancies lasting 41+ weeks	1.7	0.9
Costa Rica	CRI	Improved sanitation - Utilization of latrines or toilets	97.8	97.8
Costa Rica	CRI	Improved water source	99.0	99.0
Costa Rica	CRI	Water connection in the home	99.0	99.0
Costa Rica	CRI	Hand washing with soap	89.4	89.4
Costa Rica	CRI	Hygienic disposal of children's stools	27.9	27.9
Costa Rica	CRI	Injectable antibiotics for neonatal sepsis	98.1	47.2
Costa Rica	CRI	ORS - oral rehydration solution	40.0	20.3
Costa Rica	CRI	Antibiotics for treatment of dysentery	8.7	4.4
Costa Rica	CRI	Zinc for treatment of diarrhea	1.3	0.7
Costa Rica	CRI	Oral antibiotics for pneumonia	77.2	39.1
Costa Rica	CRI	BCG vaccine	92.0	44.2
Costa Rica	CRI	Polio vaccine	94.0	45.2
Costa Rica	CRI	DPT vaccine	94.0	45.2
Costa Rica	CRI	H. influenzae type b vaccine	94.0	78.4
Costa Rica	CRI	HepB vaccine	98.0	47.1
Costa Rica	CRI	Pneumococcal vaccine	96.0	80.1
Costa Rica	CRI	Measles vaccine	94.0	45.2
Costa Rica	CRI	Global wasting (<-2 SD) rate	2.4	3.6
Costa Rica	CRI	Contraceptive prevalence (CPR)	77.45	47.1
Côte d'Ivoire	CIV	TT - Tetanus toxoid vaccination	85.0	40.9

Country	ISO 3166-1 alpha-3	Intervention Name	Baseline coverage (%)	Ending coverage (%)
Côte d'Ivoire	CIV	IPTp - Intermittent preventive treatment of malaria during pregnancy	43.8	21.1
Côte d'Ivoire	CIV	Syphilis detection and treatment	23.0	11.1
Côte d'Ivoire	CIV	Iron supplementation in pregnancy	25.0	12.0
Côte d'Ivoire	CIV	Hypertensive disorder case management	12.3	5.9
Côte d'Ivoire	CIV	Diabetes case management	9.6	4.6
Côte d'Ivoire	CIV	Malaria case management	39.7	19.1
Côte d'Ivoire	CIV	MgSO4 management of pre-eclampsia	24.4	11.7
Côte d'Ivoire	CIV	Thermal protection	69.0	34.9
Côte d'Ivoire	CIV	Clean cord care	66.6	33.7
Côte d'Ivoire	CIV	Clean birth environment	57.2	29.0
Côte d'Ivoire	CIV	Immediate drying and additional stimulation	63.9	32.3
Côte d'Ivoire	CIV	Neonatal resuscitation	38.4	19.4
Côte d'Ivoire	CIV	Antibiotics for preterm or prolonged PROM	52.2	26.4
Côte d'Ivoire	CIV	Parenteral administration of anti-convulsants	49.9	25.3
Côte d'Ivoire	CIV	Parenteral administration of uterotonics	62.4	31.6
Côte d'Ivoire	CIV	Parenteral administration of antibiotics	52.2	26.4
Côte d'Ivoire	CIV	Assisted vaginal delivery	17.6	8.9
Côte d'Ivoire	CIV	Manual removal of placenta	26.1	13.2
Côte d'Ivoire	CIV	Removal of retained products of conception	23.1	11.7
Côte d'Ivoire	CIV	Cesarean delivery	6.1	3.1
Côte d'Ivoire	CIV	Blood transfusion	8.8	4.5
Côte d'Ivoire	CIV	Induction of labor for pregnancies lasting 41+ weeks	1.2	0.6
Côte d'Ivoire	CIV	Complementary feeding - education only	26.0	15.0
Côte d'Ivoire	CIV	Complementary feeding - supplementary feeding and education	26.0	15.0
Côte d'Ivoire	CIV	Vitamin A supplementation	94.0	54.2
Côte d'Ivoire	CIV	Improved sanitation - Utilization of latrines or toilets	32.1	32.1
Côte d'Ivoire	CIV	Improved water source	72.9	72.9
Côte d'Ivoire	CIV	Water connection in the home	30.2	30.2
Côte d'Ivoire	CIV	Hand washing with soap	18.0	18.0
Côte d'Ivoire	CIV	Hygienic disposal of children's stools	54.4	54.4
Côte d'Ivoire	CIV	ITN/IRS - Households protected from malaria	75.8	43.7
Côte d'Ivoire	CIV	Injectable antibiotics for neonatal sepsis	69.8	33.6
Côte d'Ivoire	CIV	ORS - oral rehydration solution	16.5	8.4
Côte d'Ivoire	CIV	Antibiotics for treatment of dysentery	17.0	8.6
Côte d'Ivoire	CIV	Zinc for treatment of diarrhea	18.3	9.3
Côte d'Ivoire	CIV	Oral antibiotics for pneumonia	44.0	22.3
Côte d'Ivoire	CIV	Vitamin A for treatment of measles	94.0	47.6
Côte d'Ivoire	CIV	ACTs- Artemisinin compounds for treatment of malaria	0.3	0.2
Côte d'Ivoire	CIV	BCG vaccine	98.0	47.1
Côte d'Ivoire	CIV	Polio vaccine	82.0	39.4

Country	ISO 3166-1 alpha-3	Intervention Name	Baseline coverage (%)	Ending coverage (%)
Côte d'Ivoire	CIV	DPT vaccine	82.0	39.4
Côte d'Ivoire	CIV	H. influenzae type b vaccine	82.0	68.4
Côte d'Ivoire	CIV	HepB vaccine	82.0	39.4
Côte d'Ivoire	CIV	Pneumococcal vaccine	81.0	67.5
Côte d'Ivoire	CIV	Rotavirus vaccine	59.0	49.2
Côte d'Ivoire	CIV	Measles vaccine	71.0	34.1
Côte d'Ivoire	CIV	Global wasting (<-2 SD) rate	6.0	9.0
Côte d'Ivoire	CIV	Contraceptive prevalence (CPR)	21.25	12.9
Cuba	CUB	Safe abortion services	53.7	32.6
Cuba	CUB	Syphilis detection and treatment	24.3	11.7
Cuba	CUB	Hypertensive disorder case management	23.5	11.3
Cuba	CUB	Diabetes case management	18.3	8.8
Cuba	CUB	Malaria case management	75.8	36.5
Cuba	CUB	MgSO4 management of pre-eclampsia	46.5	22.4
Cuba	CUB	Thermal protection	97.9	49.6
Cuba	CUB	Clean cord care	94.5	47.8
Cuba	CUB	Clean birth environment	81.2	41.1
Cuba	CUB	Immediate drying and additional stimulation	90.7	45.9
Cuba	CUB	Neonatal resuscitation	54.5	27.6
Cuba	CUB	Antibiotics for preterm or prolonged PROM	74.1	37.5
Cuba	CUB	Parenteral administration of anti-convulsants	70.8	35.8
Cuba	CUB	Parenteral administration of uterotonics	88.5	44.8
Cuba	CUB	Parenteral administration of antibiotics	74.1	37.5
Cuba	CUB	Assisted vaginal delivery	25.0	12.7
Cuba	CUB	Manual removal of placenta	37.0	18.7
Cuba	CUB	Removal of retained products of conception	32.8	16.6
Cuba	CUB	Cesarean delivery	8.6	4.4
Cuba	CUB	Blood transfusion	12.5	6.3
Cuba	CUB	Induction of labor for pregnancies lasting 41+ weeks	1.7	0.9
Cuba	CUB	Complementary feeding - education only	80.3	46.3
Cuba	CUB	Complementary feeding - supplementary feeding and education	80.3	46.3
Cuba	CUB	Improved sanitation - Utilization of latrines or toilets	92.8	92.8
Cuba	CUB	Improved water source	95.3	95.3
Cuba	CUB	Water connection in the home	75.8	75.8
Cuba	CUB	Hand washing with soap	85.2	85.2
Cuba	CUB	Hygienic disposal of children's stools	87.9	87.9
Cuba	CUB	Injectable antibiotics for neonatal sepsis	99.0	47.6
Cuba	CUB	ORS - oral rehydration solution	60.9	30.8
Cuba	CUB	Antibiotics for treatment of dysentery	2.0	1.0
Cuba	CUB	Zinc for treatment of diarrhea	17.3	8.8

Country	ISO 3166-1 alpha-3	Intervention Name	Baseline coverage (%)	Ending coverage (%)
Cuba	CUB	Oral antibiotics for pneumonia	92.6	46.9
Cuba	CUB	BCG vaccine	99.0	47.6
Cuba	CUB	Polio vaccine	99.0	47.6
Cuba	CUB	DPT vaccine	99.0	47.6
Cuba	CUB	H. influenzae type b vaccine	99.0	82.6
Cuba	CUB	HepB vaccine	99.0	47.6
Cuba	CUB	Measles vaccine	99.0	47.6
Cuba	CUB	Global wasting (<-2 SD) rate	2.2	3.3
Cuba	CUB	Contraceptive prevalence (CPR)	74.4	45.2
Dem. People's Republic of Korea	PRK	Safe abortion services	100.0	60.8
Dem. People's Republic of Korea	PRK	TT - Tetanus toxoid vaccination	98.0	47.1
Dem. People's Republic of Korea	PRK	Syphilis detection and treatment	24.4	11.7
Dem. People's Republic of Korea	PRK	Hypertensive disorder case management	22.5	10.8
Dem. People's Republic of Korea	PRK	Diabetes case management	17.6	8.5
Dem. People's Republic of Korea	PRK	Malaria case management	72.6	34.9
Dem. People's Republic of Korea	PRK	MgSO4 management of pre-eclampsia	44.5	21.4
Dem. People's Republic of Korea	PRK	Thermal protection	91.1	46.1
Dem. People's Republic of Korea	PRK	Clean cord care	88.0	44.6
Dem. People's Republic of Korea	PRK	Clean birth environment	75.6	38.3
Dem. People's Republic of Korea	PRK	Immediate drying and additional stimulation	84.4	42.7
Dem. People's Republic of Korea	PRK	Neonatal resuscitation	50.7	25.7
Dem. People's Republic of Korea	PRK	Antibiotics for preterm or prolonged PROM	69.0	34.9
Dem. People's Republic of Korea	PRK	Parenteral administration of anti-convulsants	65.9	33.4
Dem. People's Republic of Korea	PRK	Parenteral administration of uterotonics	82.4	41.7
Dem. People's Republic of Korea	PRK	Parenteral administration of antibiotics	69.0	34.9
Dem. People's Republic of Korea	PRK	Assisted vaginal delivery	23.3	11.8
Dem. People's Republic of Korea	PRK	Manual removal of placenta	34.5	17.5
Dem. People's Republic of Korea	PRK	Removal of retained products of conception	30.6	15.5
Dem. People's Republic of Korea	PRK	Cesarean delivery	8.0	4.1

Country	ISO 3166-1 alpha-3	Intervention Name	Baseline coverage (%)	Ending coverage (%)
Dem. People's Republic of Korea	PRK	Blood transfusion	11.6	5.9
Dem. People's Republic of Korea	PRK	Induction of labor for pregnancies lasting 41+ weeks	1.6	0.8
Dem. People's Republic of Korea	PRK	Complementary feeding - education only	48.7	28.1
Dem. People's Republic of Korea	PRK	Complementary feeding - supplementary feeding and education	48.7	28.1
Dem. People's Republic of Korea	PRK	Vitamin A supplementation	90.0	51.9
Dem. People's Republic of Korea	PRK	Improved sanitation - Utilization of latrines or toilets	83.2	83.2
Dem. People's Republic of Korea	PRK	Improved water source	99.0	99.0
Dem. People's Republic of Korea	PRK	Water connection in the home	63.8	63.8
Dem. People's Republic of Korea	PRK	Hygienic disposal of children's stools	74.2	74.2
Dem. People's Republic of Korea	PRK	Injectable antibiotics for neonatal sepsis	92.2	44.3
Dem. People's Republic of Korea	PRK	ORS - oral rehydration solution	74.1	37.5
Dem. People's Republic of Korea	PRK	Zinc for treatment of diarrhea	50.7	25.7
Dem. People's Republic of Korea	PRK	Oral antibiotics for pneumonia	79.8	40.4
Dem. People's Republic of Korea	PRK	Vitamin A for treatment of measles	90.0	45.6
Dem. People's Republic of Korea	PRK	BCG vaccine	96.0	46.2
Dem. People's Republic of Korea	PRK	Polio vaccine	99.0	47.6
Dem. People's Republic of Korea	PRK	DPT vaccine	97.0	46.7
Dem. People's Republic of Korea	PRK	H. influenzae type b vaccine	97.0	80.9
Dem. People's Republic of Korea	PRK	HepB vaccine	97.0	46.7
Dem. People's Republic of Korea	PRK	Measles vaccine	98.0	47.1
Dem. People's Republic of Korea	PRK	Global wasting (<-2 SD) rate	10.6	15.9
Dem. People's Republic of Korea	PRK	Contraceptive prevalence (CPR)	74.1	45.0
Democratic Republic of the Congo	COD	TT - Tetanus toxoid vaccination	85.0	40.9
Democratic Republic of the Congo	COD	IPTp - Intermittent preventive treatment of malaria during pregnancy	15.0	7.2
Democratic Republic of the Congo	COD	Syphilis detection and treatment	34.9	16.8
Democratic Republic of the Congo	COD	Iron supplementation in pregnancy	4.7	2.3

Country	ISO 3166-1 alpha-3	Intervention Name	Baseline coverage (%)	Ending coverage (%)
Democratic Republic of the Congo	COD	Hypertensive disorder case management	4.6	2.2
Democratic Republic of the Congo	COD	Diabetes case management	4.7	2.3
Democratic Republic of the Congo	COD	Malaria case management	31.9	15.3
Democratic Republic of the Congo	COD	MgSO4 management of pre-eclampsia	6.6	3.2
Democratic Republic of the Congo	COD	Thermal protection	78.9	39.9
Democratic Republic of the Congo	COD	Clean cord care	66.6	33.7
Democratic Republic of the Congo	COD	Clean birth environment	40.9	20.7
Democratic Republic of the Congo	COD	Immediate drying and additional stimulation	63.2	32.0
Democratic Republic of the Congo	COD	Neonatal resuscitation	4.4	2.2
Democratic Republic of the Congo	COD	Antibiotics for preterm or prolonged PROM	12.2	6.2
Democratic Republic of the Congo	COD	Parenteral administration of anti-convulsants	14.2	7.2
Democratic Republic of the Congo	COD	Parenteral administration of uterotonics	61.3	31.0
Democratic Republic of the Congo	COD	Parenteral administration of antibiotics	12.2	6.2
Democratic Republic of the Congo	COD	Assisted vaginal delivery	8.9	4.5
Democratic Republic of the Congo	COD	Manual removal of placenta	21.2	10.7
Democratic Republic of the Congo	COD	Removal of retained products of conception	19.9	10.1
Democratic Republic of the Congo	COD	Cesarean delivery	0.8	0.4
Democratic Republic of the Congo	COD	Blood transfusion	6.6	3.3
Democratic Republic of the Congo	COD	Induction of labor for pregnancies lasting 41+ weeks	1.2	0.6
Democratic Republic of the Congo	COD	Complementary feeding - education only	19.9	11.5
Democratic Republic of the Congo	COD	Complementary feeding - supplementary feeding and education	19.9	11.5
Democratic Republic of the Congo	COD	Vitamin A supplementation	1.0	0.6
Democratic Republic of the Congo	COD	Improved sanitation - Utilization of latrines or toilets	20.5	20.5
Democratic Republic of the Congo	COD	Improved water source	43.2	43.2
Democratic Republic of the Congo	COD	Water connection in the home	13.9	13.9
Democratic Republic of the Congo	COD	Hand washing with soap	4.4	4.4

Country	ISO 3166-1 alpha-3	Intervention Name	Baseline coverage (%)	Ending coverage (%)
Democratic Republic of the Congo	COD	Hygienic disposal of children's stools	61.1	61.1
Democratic Republic of the Congo	COD	ITN/IRS - Households protected from malaria	70.0	40.4
Democratic Republic of the Congo	COD	Injectable antibiotics for neonatal sepsis	79.9	38.4
Democratic Republic of the Congo	COD	ORS - oral rehydration solution	39.1	19.8
Democratic Republic of the Congo	COD	Antibiotics for treatment of dysentery	38.4	19.4
Democratic Republic of the Congo	COD	Zinc for treatment of diarrhea	2.4	1.2
Democratic Republic of the Congo	COD	Oral antibiotics for pneumonia	41.6	21.1
Democratic Republic of the Congo	COD	Vitamin A for treatment of measles	1.0	0.5
Democratic Republic of the Congo	COD	ACTs- Artemisinin compounds for treatment of malaria	2.0	1.0
Democratic Republic of the Congo	COD	SAM - treatment for severe acute malnutrition	11.2	5.7
Democratic Republic of the Congo	COD	BCG vaccine	83.0	39.9
Democratic Republic of the Congo	COD	Polio vaccine	79.0	38.0
Democratic Republic of the Congo	COD	DPT vaccine	81.0	39.0
Democratic Republic of the Congo	COD	H. influenzae type b vaccine	81.0	67.5
Democratic Republic of the Congo	COD	HepB vaccine	81.0	39.0
Democratic Republic of the Congo	COD	Pneumococcal vaccine	81.0	67.5
Democratic Republic of the Congo	COD	Meningococcal A	99.0	47.6
Democratic Republic of the Congo	COD	Measles vaccine	80.0	38.5
Democratic Republic of the Congo	COD	Global wasting (<-2 SD) rate	8.1	12.1
Democratic Republic of the Congo	COD	Contraceptive prevalence (CPR)	24.55	14.9
Djibouti	DJI	Safe abortion services	3.3	2.0
Djibouti	DJI	TT - Tetanus toxoid vaccination	98.0	47.1
Djibouti	DJI	Syphilis detection and treatment	5.6	2.7
Djibouti	DJI	Hypertensive disorder case management	5.4	2.6
Djibouti	DJI	Diabetes case management	4.2	2.0
Djibouti	DJI	Malaria case management	17.5	8.4
Djibouti	DJI	MgSO4 management of pre-eclampsia	10.7	5.1
Djibouti	DJI	Thermal protection	86.4	43.7
Djibouti	DJI	Clean cord care	83.4	42.2
Djibouti	DJI	Clean birth environment	71.7	36.3

Country	ISO 3166-1 alpha-3	Intervention Name	Baseline coverage (%)	Ending coverage (%)
Djibouti	DJI	Immediate drying and additional stimulation	80.0	40.5
Djibouti	DJI	Neonatal resuscitation	48.1	24.4
Djibouti	DJI	Antibiotics for preterm or prolonged PROM	65.4	33.1
Djibouti	DJI	Parenteral administration of anti-convulsants	62.5	31.6
Djibouti	DJI	Parenteral administration of uterotonics	78.1	39.5
Djibouti	DJI	Parenteral administration of antibiotics	65.4	33.1
Djibouti	DJI	Assisted vaginal delivery	22.1	11.2
Djibouti	DJI	Manual removal of placenta	32.7	16.6
Djibouti	DJI	Removal of retained products of conception	29.0	14.7
Djibouti	DJI	Cesarean delivery	7.6	3.8
Djibouti	DJI	Blood transfusion	11.0	5.6
Djibouti	DJI	Induction of labor for pregnancies lasting 41+ weeks	1.5	0.8
Djibouti	DJI	Complementary feeding - education only	23.1	13.3
Djibouti	DJI	Complementary feeding - supplementary feeding and education	23.1	13.3
Djibouti	DJI	Vitamin A supplementation	78.0	45.0
Djibouti	DJI	Improved sanitation - Utilization of latrines or toilets	63.6	63.6
Djibouti	DJI	Improved water source	75.6	75.6
Djibouti	DJI	Water connection in the home	61.8	61.8
Djibouti	DJI	ITN/IRS - Households protected from malaria	30.2	17.4
Djibouti	DJI	Injectable antibiotics for neonatal sepsis	87.4	42.0
Djibouti	DJI	ORS - oral rehydration solution	61.6	31.2
Djibouti	DJI	Oral antibiotics for pneumonia	62.1	31.4
Djibouti	DJI	Vitamin A for treatment of measles	78.0	39.5
Djibouti	DJI	SAM - treatment for severe acute malnutrition	32.4	16.4
Djibouti	DJI	BCG vaccine	93.0	44.7
Djibouti	DJI	Polio vaccine	84.0	40.4
Djibouti	DJI	DPT vaccine	84.0	40.4
Djibouti	DJI	H. influenzae type b vaccine	84.0	70.0
Djibouti	DJI	HepB vaccine	84.0	40.4
Djibouti	DJI	Pneumococcal vaccine	84.0	70.0
Djibouti	DJI	Rotavirus vaccine	87.0	72.5
Djibouti	DJI	Measles vaccine	86.0	41.4
Djibouti	DJI	Global wasting (<-2 SD) rate	26.8	40.2
Djibouti	DJI	Contraceptive prevalence (CPR)	27.6	16.8
Dominican Republic	DOM	Safe abortion services	53.7	32.6
Dominican Republic	DOM	TT - Tetanus toxoid vaccination	99.0	47.6
Dominican Republic	DOM	Syphilis detection and treatment	24.2	11.6
Dominican Republic	DOM	Iron supplementation in pregnancy	81.5	39.2
Dominican Republic	DOM	Hypertensive disorder case management	22.3	10.7
Dominican Republic	DOM	Diabetes case management	17.4	8.4

Country	ISO 3166-1 alpha-3	Intervention Name	Baseline coverage (%)	Ending coverage (%)
Dominican Republic	DOM	Malaria case management	72.0	34.6
Dominican Republic	DOM	MgSO4 management of pre-eclampsia	44.2	21.3
Dominican Republic	DOM	Thermal protection	96.7	49.0
Dominican Republic	DOM	Clean cord care	93.4	47.3
Dominican Republic	DOM	Clean birth environment	80.3	40.7
Dominican Republic	DOM	Immediate drying and additional stimulation	89.6	45.4
Dominican Republic	DOM	Neonatal resuscitation	53.8	27.2
Dominican Republic	DOM	Antibiotics for preterm or prolonged PROM	73.2	37.1
Dominican Republic	DOM	Parenteral administration of anti-convulsants	70.0	35.4
Dominican Republic	DOM	Parenteral administration of uterotonics	87.5	44.3
Dominican Republic	DOM	Parenteral administration of antibiotics	73.2	37.1
Dominican Republic	DOM	Assisted vaginal delivery	24.7	12.5
Dominican Republic	DOM	Manual removal of placenta	36.6	18.5
Dominican Republic	DOM	Removal of retained products of conception	32.4	16.4
Dominican Republic	DOM	Cesarean delivery	8.5	4.3
Dominican Republic	DOM	Blood transfusion	12.3	6.2
Dominican Republic	DOM	Induction of labor for pregnancies lasting 41+ weeks	1.7	0.9
Dominican Republic	DOM	Complementary feeding - education only	68.5	39.5
Dominican Republic	DOM	Complementary feeding - supplementary feeding and education	68.5	39.5
Dominican Republic	DOM	Improved sanitation - Utilization of latrines or toilets	83.9	83.9
Dominican Republic	DOM	Improved water source	96.7	96.7
Dominican Republic	DOM	Water connection in the home	77.9	77.9
Dominican Republic	DOM	Hand washing with soap	57.1	57.1
Dominican Republic	DOM	Hygienic disposal of children's stools	27.4	27.4
Dominican Republic	DOM	Injectable antibiotics for neonatal sepsis	97.9	47.1
Dominican Republic	DOM	ORS - oral rehydration solution	47.5	24.0
Dominican Republic	DOM	Antibiotics for treatment of dysentery	30.5	15.4
Dominican Republic	DOM	Oral antibiotics for pneumonia	72.4	36.7
Dominican Republic	DOM	BCG vaccine	99.0	47.6
Dominican Republic	DOM	Polio vaccine	89.0	42.8
Dominican Republic	DOM	DPT vaccine	94.0	45.2
Dominican Republic	DOM	H. influenzae type b vaccine	90.0	75.1
Dominican Republic	DOM	HepB vaccine	92.0	44.2
Dominican Republic	DOM	Pneumococcal vaccine	70.0	58.4
Dominican Republic	DOM	Rotavirus vaccine	82.0	68.4
Dominican Republic	DOM	Measles vaccine	95.0	45.7
Dominican Republic	DOM	Global wasting (<-2 SD) rate	2.5	3.7
Dominican Republic	DOM	Contraceptive prevalence (CPR)	71.15	43.2
Ecuador	ECU	Safe abortion services	0.2	0.1
Ecuador	ECU	TT - Tetanus toxoid vaccination	88.0	42.3

Country	ISO 3166-1 alpha-3	Intervention Name	Baseline coverage (%)	Ending coverage (%)
Ecuador	ECU	Syphilis detection and treatment	23.9	11.5
Ecuador	ECU	Hypertensive disorder case management	21.2	10.2
Ecuador	ECU	Diabetes case management	16.5	7.9
Ecuador	ECU	Malaria case management	68.4	32.9
Ecuador	ECU	MgSO4 management of pre-eclampsia	41.9	20.2
Ecuador	ECU	Thermal protection	89.3	45.2
Ecuador	ECU	Clean cord care	86.2	43.6
Ecuador	ECU	Clean birth environment	74.1	37.5
Ecuador	ECU	Immediate drying and additional stimulation	82.7	41.9
Ecuador	ECU	Neonatal resuscitation	49.7	25.2
Ecuador	ECU	Antibiotics for preterm or prolonged PROM	67.6	34.2
Ecuador	ECU	Parenteral administration of anti-convulsants	64.6	32.7
Ecuador	ECU	Parenteral administration of uterotonics	80.8	40.9
Ecuador	ECU	Parenteral administration of antibiotics	67.6	34.2
Ecuador	ECU	Assisted vaginal delivery	22.8	11.5
Ecuador	ECU	Manual removal of placenta	33.8	17.1
Ecuador	ECU	Removal of retained products of conception	29.9	15.1
Ecuador	ECU	Cesarean delivery	7.9	4.0
Ecuador	ECU	Blood transfusion	11.4	5.8
Ecuador	ECU	Induction of labor for pregnancies lasting 41+ weeks	1.6	0.8
Ecuador	ECU	Complementary feeding - education only	69.4	40.1
Ecuador	ECU	Complementary feeding - supplementary feeding and education	69.4	40.1
Ecuador	ECU	Improved sanitation - Utilization of latrines or toilets	88.0	88.0
Ecuador	ECU	Improved water source	94.0	94.0
Ecuador	ECU	Water connection in the home	82.6	82.6
Ecuador	ECU	Injectable antibiotics for neonatal sepsis	90.4	43.5
Ecuador	ECU	ORS - oral rehydration solution	27.3	13.8
Ecuador	ECU	Antibiotics for treatment of dysentery	32.4	16.4
Ecuador	ECU	BCG vaccine	90.0	43.3
Ecuador	ECU	Polio vaccine	85.0	40.9
Ecuador	ECU	DPT vaccine	85.0	40.9
Ecuador	ECU	H. influenzae type b vaccine	85.0	70.9
Ecuador	ECU	HepB vaccine	85.0	40.9
Ecuador	ECU	Pneumococcal vaccine	85.0	70.9
Ecuador	ECU	Rotavirus vaccine	85.0	70.9
Ecuador	ECU	Measles vaccine	83.0	39.9
Ecuador	ECU	Global wasting (<-2 SD) rate	2.5	3.8
Ecuador	ECU	Contraceptive prevalence (CPR)	79.35	48.2
Egypt	EGY	Safe abortion services	2.2	1.3
Egypt	EGY	TT - Tetanus toxoid vaccination	86.0	41.4

Country	ISO 3166-1 alpha-3	Intervention Name	Baseline coverage (%)	Ending coverage (%)
Egypt	EGY	Syphilis detection and treatment	22.6	10.9
Egypt	EGY	Iron supplementation in pregnancy	36.1	17.4
Egypt	EGY	Hypertensive disorder case management	20.1	9.7
Egypt	EGY	Diabetes case management	15.7	7.6
Egypt	EGY	Malaria case management	65.0	31.3
Egypt	EGY	MgSO4 management of pre-eclampsia	39.8	19.1
Egypt	EGY	Thermal protection	85.7	43.4
Egypt	EGY	Clean cord care	82.8	41.9
Egypt	EGY	Clean birth environment	71.1	36.0
Egypt	EGY	Immediate drying and additional stimulation	79.4	40.2
Egypt	EGY	Neonatal resuscitation	47.7	24.1
Egypt	EGY	Antibiotics for preterm or prolonged PROM	64.9	32.9
Egypt	EGY	Parenteral administration of anti-convulsants	62.0	31.4
Egypt	EGY	Parenteral administration of uterotonics	77.5	39.2
Egypt	EGY	Parenteral administration of antibiotics	64.9	32.9
Egypt	EGY	Assisted vaginal delivery	21.9	11.1
Egypt	EGY	Manual removal of placenta	32.4	16.4
Egypt	EGY	Removal of retained products of conception	28.7	14.5
Egypt	EGY	Cesarean delivery	7.5	3.8
Egypt	EGY	Blood transfusion	10.9	5.5
Egypt	EGY	Induction of labor for pregnancies lasting 41+ weeks	1.5	0.8
Egypt	EGY	Complementary feeding - education only	43.2	24.9
Egypt	EGY	Complementary feeding - supplementary feeding and education	43.2	24.9
Egypt	EGY	Vitamin A supplementation	68.0	39.2
Egypt	EGY	Improved sanitation - Utilization of latrines or toilets	94.2	94.2
Egypt	EGY	Improved water source	99.0	99.0
Egypt	EGY	Water connection in the home	97.2	97.2
Egypt	EGY	Hand washing with soap	89.0	89.0
Egypt	EGY	Hygienic disposal of children's stools	35.7	35.7
Egypt	EGY	Injectable antibiotics for neonatal sepsis	86.7	41.7
Egypt	EGY	ORS - oral rehydration solution	28.4	14.4
Egypt	EGY	Antibiotics for treatment of dysentery	13.2	6.7
Egypt	EGY	Zinc for treatment of diarrhea	1.7	0.9
Egypt	EGY	Oral antibiotics for pneumonia	67.7	34.3
Egypt	EGY	Vitamin A for treatment of measles	68.0	34.4
Egypt	EGY	BCG vaccine	95.0	45.7
Egypt	EGY	Polio vaccine	95.0	45.7
Egypt	EGY	DPT vaccine	95.0	45.7
Egypt	EGY	H. influenzae type b vaccine	95.0	79.2
Egypt	EGY	HepB vaccine	95.0	45.7

Country	ISO 3166-1 alpha-3	Intervention Name	Baseline coverage (%)	Ending coverage (%)
Egypt	EGY	Measles vaccine	94.0	45.2
Egypt	EGY	Global wasting (<-2 SD) rate	9.4	14.1
Egypt	EGY	Contraceptive prevalence (CPR)	60.95	37.0
El Salvador	SLV	Safe abortion services	0.6	0.4
El Salvador	SLV	TT - Tetanus toxoid vaccination	92.0	44.2
El Salvador	SLV	Syphilis detection and treatment	23.7	11.4
El Salvador	SLV	Hypertensive disorder case management	21.6	10.4
El Salvador	SLV	Diabetes case management	16.9	8.1
El Salvador	SLV	Malaria case management	69.9	33.6
El Salvador	SLV	MgSO4 management of pre-eclampsia	42.8	20.6
El Salvador	SLV	Thermal protection	96.4	48.8
El Salvador	SLV	Clean cord care	93.1	47.1
El Salvador	SLV	Clean birth environment	80.0	40.5
El Salvador	SLV	Immediate drying and additional stimulation	89.3	45.2
El Salvador	SLV	Neonatal resuscitation	53.6	27.1
El Salvador	SLV	Antibiotics for preterm or prolonged PROM	73.0	37.0
El Salvador	SLV	Parenteral administration of anti-convulsants	69.7	35.3
El Salvador	SLV	Parenteral administration of uterotonics	87.2	44.1
El Salvador	SLV	Parenteral administration of antibiotics	73.0	37.0
El Salvador	SLV	Assisted vaginal delivery	24.7	12.5
El Salvador	SLV	Manual removal of placenta	36.5	18.5
El Salvador	SLV	Removal of retained products of conception	32.3	16.4
El Salvador	SLV	Cesarean delivery	8.5	4.3
El Salvador	SLV	Blood transfusion	12.3	6.2
El Salvador	SLV	Induction of labor for pregnancies lasting 41+ weeks	1.7	0.9
El Salvador	SLV	Complementary feeding - education only	78.2	45.1
El Salvador	SLV	Complementary feeding - supplementary feeding and education	78.2	45.1
El Salvador	SLV	Vitamin A supplementation	81.0	46.7
El Salvador	SLV	Improved sanitation - Utilization of latrines or toilets	87.4	87.4
El Salvador	SLV	Improved water source	97.4	97.4
El Salvador	SLV	Water connection in the home	87.4	87.4
El Salvador	SLV	Hand washing with soap	91.3	91.3
El Salvador	SLV	Hygienic disposal of children's stools	41.8	41.8
El Salvador	SLV	Injectable antibiotics for neonatal sepsis	97.5	46.9
El Salvador	SLV	ORS - oral rehydration solution	69.8	35.3
El Salvador	SLV	Antibiotics for treatment of dysentery	40.8	20.7
El Salvador	SLV	Zinc for treatment of diarrhea	32.7	16.6
El Salvador	SLV	Oral antibiotics for pneumonia	79.7	40.3
El Salvador	SLV	Vitamin A for treatment of measles	81.0	41.0
El Salvador	SLV	SAM - treatment for severe acute malnutrition	1.9	1.0

Country	ISO 3166-1 alpha-3	Intervention Name	Baseline coverage (%)	Ending coverage (%)
El Salvador	SLV	BCG vaccine	81.0	39.0
El Salvador	SLV	Polio vaccine	83.0	39.9
El Salvador	SLV	DPT vaccine	81.0	39.0
El Salvador	SLV	H. influenzae type b vaccine	81.0	67.5
El Salvador	SLV	HepB vaccine	81.0	39.0
El Salvador	SLV	Pneumococcal vaccine	75.0	62.5
El Salvador	SLV	Rotavirus vaccine	82.0	68.4
El Salvador	SLV	Measles vaccine	81.0	39.0
El Salvador	SLV	Global wasting (<-2 SD) rate	2.2	3.3
El Salvador	SLV	Contraceptive prevalence (CPR)	73.05	44.4
Equatorial Guinea	GNQ	TT - Tetanus toxoid vaccination	70.0	33.7
Equatorial Guinea	GNQ	IPTp - Intermittent preventive treatment of malaria during pregnancy	27.6	13.3
Equatorial Guinea	GNQ	Syphilis detection and treatment	21.9	10.5
Equatorial Guinea	GNQ	Iron supplementation in pregnancy	8.7	4.2
Equatorial Guinea	GNQ	Hypertensive disorder case management	16.1	7.7
Equatorial Guinea	GNQ	Diabetes case management	12.5	6.0
Equatorial Guinea	GNQ	Malaria case management	51.9	25.0
Equatorial Guinea	GNQ	MgSO4 management of pre-eclampsia	31.8	15.3
Equatorial Guinea	GNQ	Thermal protection	66.5	33.7
Equatorial Guinea	GNQ	Clean cord care	64.2	32.5
Equatorial Guinea	GNQ	Clean birth environment	55.2	27.9
Equatorial Guinea	GNQ	Immediate drying and additional stimulation	61.6	31.2
Equatorial Guinea	GNQ	Neonatal resuscitation	37.0	18.7
Equatorial Guinea	GNQ	Antibiotics for preterm or prolonged PROM	50.4	25.5
Equatorial Guinea	GNQ	Parenteral administration of anti-convulsants	48.1	24.4
Equatorial Guinea	GNQ	Parenteral administration of uterotonics	60.1	30.4
Equatorial Guinea	GNQ	Parenteral administration of antibiotics	50.4	25.5
Equatorial Guinea	GNQ	Assisted vaginal delivery	17.0	8.6
Equatorial Guinea	GNQ	Manual removal of placenta	25.2	12.8
Equatorial Guinea	GNQ	Removal of retained products of conception	22.3	11.3
Equatorial Guinea	GNQ	Cesarean delivery	5.9	3.0
Equatorial Guinea	GNQ	Blood transfusion	8.5	4.3
Equatorial Guinea	GNQ	Induction of labor for pregnancies lasting 41+ weeks	1.2	0.6
Equatorial Guinea	GNQ	Complementary feeding - education only	40.7	23.5
Equatorial Guinea	GNQ	Complementary feeding - supplementary feeding and education	40.7	23.5
Equatorial Guinea	GNQ	Vitamin A supplementation	30.0	17.3
Equatorial Guinea	GNQ	Improved sanitation - Utilization of latrines or toilets	66.3	66.3
Equatorial Guinea	GNQ	Improved water source	64.7	64.7
Equatorial Guinea	GNQ	Water connection in the home	26.4	26.4
Equatorial Guinea	GNQ	ITN/IRS - Households protected from malaria	63.7	36.8

Country	ISO 3166-1 alpha-3	Intervention Name	Baseline coverage (%)	Ending coverage (%)
Equatorial Guinea	GNQ	Injectable antibiotics for neonatal sepsis	67.3	32.4
Equatorial Guinea	GNQ	ORS - oral rehydration solution	40.4	20.5
Equatorial Guinea	GNQ	Antibiotics for treatment of dysentery	9.3	4.7
Equatorial Guinea	GNQ	Zinc for treatment of diarrhea	0.2	0.1
Equatorial Guinea	GNQ	Oral antibiotics for pneumonia	54.3	27.5
Equatorial Guinea	GNQ	Vitamin A for treatment of measles	30.0	15.2
Equatorial Guinea	GNQ	ACTs- Artemisinin compounds for treatment of malaria	9.0	4.6
Equatorial Guinea	GNQ	BCG vaccine	63.0	30.3
Equatorial Guinea	GNQ	Polio vaccine	27.0	13.0
Equatorial Guinea	GNQ	DPT vaccine	25.0	12.0
Equatorial Guinea	GNQ	H. influenzae type b vaccine	25.0	20.8
Equatorial Guinea	GNQ	HepB vaccine	25.0	12.0
Equatorial Guinea	GNQ	Measles vaccine	30.0	14.4
Equatorial Guinea	GNQ	Global wasting (<-2 SD) rate	10.0	15.0
Equatorial Guinea	GNQ	Contraceptive prevalence (CPR)	17.35	10.5
Eritrea	ERI	Safe abortion services	3.3	2.0
Eritrea	ERI	TT - Tetanus toxoid vaccination	99.0	47.6
Eritrea	ERI	Syphilis detection and treatment	17.5	8.4
Eritrea	ERI	Hypertensive disorder case management	13.8	6.6
Eritrea	ERI	Diabetes case management	10.8	5.2
Eritrea	ERI	Malaria case management	44.5	21.4
Eritrea	ERI	MgSO4 management of pre-eclampsia	27.3	13.1
Eritrea	ERI	Thermal protection	33.2	16.8
Eritrea	ERI	Clean cord care	32.1	16.3
Eritrea	ERI	Clean birth environment	27.6	14.0
Eritrea	ERI	Immediate drying and additional stimulation	30.8	15.6
Eritrea	ERI	Neonatal resuscitation	18.5	9.4
Eritrea	ERI	Antibiotics for preterm or prolonged PROM	25.1	12.7
Eritrea	ERI	Parenteral administration of anti-convulsants	24.0	12.2
Eritrea	ERI	Parenteral administration of uterotonics	30.0	15.2
Eritrea	ERI	Parenteral administration of antibiotics	25.1	12.7
Eritrea	ERI	Assisted vaginal delivery	8.5	4.3
Eritrea	ERI	Manual removal of placenta	12.6	6.4
Eritrea	ERI	Removal of retained products of conception	11.1	5.6
Eritrea	ERI	Cesarean delivery	2.9	1.5
Eritrea	ERI	Blood transfusion	4.2	2.1
Eritrea	ERI	Induction of labor for pregnancies lasting 41+ weeks	0.6	0.3
Eritrea	ERI	Complementary feeding - education only	42.5	24.5
Eritrea	ERI	Complementary feeding - supplementary feeding and education	42.5	24.5
Eritrea	ERI	Vitamin A supplementation	51.0	29.4

Country	ISO 3166-1 alpha-3	Intervention Name	Baseline coverage (%)	Ending coverage (%)
Eritrea	ERI	Improved sanitation - Utilization of latrines or toilets	11.9	11.9
Eritrea	ERI	Improved water source	51.8	51.8
Eritrea	ERI	Water connection in the home	26.8	26.8
Eritrea	ERI	Hygienic disposal of children's stools	26.8	26.8
Eritrea	ERI	ITN/IRS - Households protected from malaria	70.9	40.9
Eritrea	ERI	Injectable antibiotics for neonatal sepsis	33.6	16.2
Eritrea	ERI	ORS - oral rehydration solution	43.4	22.0
Eritrea	ERI	Vitamin A for treatment of measles	51.0	25.8
Eritrea	ERI	BCG vaccine	97.0	46.7
Eritrea	ERI	Polio vaccine	95.0	45.7
Eritrea	ERI	DPT vaccine	95.0	45.7
Eritrea	ERI	H. influenzae type b vaccine	95.0	79.2
Eritrea	ERI	HepB vaccine	95.0	45.7
Eritrea	ERI	Pneumococcal vaccine	95.0	79.2
Eritrea	ERI	Rotavirus vaccine	96.0	80.1
Eritrea	ERI	Measles vaccine	99.0	47.6
Eritrea	ERI	Global wasting (<-2 SD) rate	7.2	10.8
Eritrea	ERI	Contraceptive prevalence (CPR)	13.8	8.4
Ethiopia	ETH	Safe abortion services	3.3	2.0
Ethiopia	ETH	TT - Tetanus toxoid vaccination	93.0	44.7
Ethiopia	ETH	IPTp - Intermittent preventive treatment of malaria during pregnancy	1.2	0.6
Ethiopia	ETH	Syphilis detection and treatment	15.7	7.6
Ethiopia	ETH	Iron supplementation in pregnancy	5.1	2.5
Ethiopia	ETH	Hypertensive disorder case management	7.9	3.8
Ethiopia	ETH	Diabetes case management	6.1	2.9
Ethiopia	ETH	Malaria case management	25.4	12.2
Ethiopia	ETH	MgSO4 management of pre-eclampsia	15.6	7.5
Ethiopia	ETH	Thermal protection	25.9	13.1
Ethiopia	ETH	Clean cord care	25.0	12.7
Ethiopia	ETH	Clean birth environment	21.5	10.9
Ethiopia	ETH	Immediate drying and additional stimulation	24.0	12.2
Ethiopia	ETH	Neonatal resuscitation	14.4	7.3
Ethiopia	ETH	Antibiotics for preterm or prolonged PROM	19.6	9.9
Ethiopia	ETH	Parenteral administration of anti-convulsants	18.8	9.5
Ethiopia	ETH	Parenteral administration of uterotonics	23.4	11.8
Ethiopia	ETH	Parenteral administration of antibiotics	19.6	9.9
Ethiopia	ETH	Assisted vaginal delivery	6.6	3.3
Ethiopia	ETH	Manual removal of placenta	9.8	5.0
Ethiopia	ETH	Removal of retained products of conception	8.7	4.4
Ethiopia	ETH	Cesarean delivery	2.3	1.2

Country	ISO 3166-1 alpha-3	Intervention Name	Baseline coverage (%)	Ending coverage (%)
Ethiopia	ETH	Blood transfusion	3.3	1.7
Ethiopia	ETH	Induction of labor for pregnancies lasting 41+ weeks	0.5	0.3
Ethiopia	ETH	Complementary feeding - education only	13.8	8.0
Ethiopia	ETH	Complementary feeding - supplementary feeding and education	13.8	8.0
Ethiopia	ETH	Vitamin A supplementation	77.0	44.4
Ethiopia	ETH	Improved sanitation - Utilization of latrines or toilets	7.3	7.3
Ethiopia	ETH	Improved water source	41.1	41.1
Ethiopia	ETH	Water connection in the home	14.8	14.8
Ethiopia	ETH	Hand washing with soap	8.3	8.3
Ethiopia	ETH	Hygienic disposal of children's stools	36.9	36.9
Ethiopia	ETH	ITN/IRS - Households protected from malaria	70.5	40.7
Ethiopia	ETH	Injectable antibiotics for neonatal sepsis	26.2	12.6
Ethiopia	ETH	ORS - oral rehydration solution	29.5	14.9
Ethiopia	ETH	Antibiotics for treatment of dysentery	9.3	4.7
Ethiopia	ETH	Zinc for treatment of diarrhea	33.3	16.9
Ethiopia	ETH	Oral antibiotics for pneumonia	29.4	14.9
Ethiopia	ETH	Vitamin A for treatment of measles	77.0	39.0
Ethiopia	ETH	SAM - treatment for severe acute malnutrition	12.0	6.1
Ethiopia	ETH	BCG vaccine	85.0	40.9
Ethiopia	ETH	Polio vaccine	67.0	32.2
Ethiopia	ETH	DPT vaccine	72.0	34.6
Ethiopia	ETH	H. influenzae type b vaccine	72.0	60.0
Ethiopia	ETH	HepB vaccine	72.0	34.6
Ethiopia	ETH	Pneumococcal vaccine	67.0	55.9
Ethiopia	ETH	Rotavirus vaccine	79.0	65.9
Ethiopia	ETH	Measles vaccine	61.0	29.3
Ethiopia	ETH	Global wasting (<-2 SD) rate	10.0	15.0
Ethiopia	ETH	Contraceptive prevalence (CPR)	39.95	24.3
Gabon	GAB	TT - Tetanus toxoid vaccination	85.0	40.9
Gabon	GAB	IPTp - Intermittent preventive treatment of malaria during pregnancy	13.1	6.3
Gabon	GAB	Syphilis detection and treatment	23.4	11.3
Gabon	GAB	Iron supplementation in pregnancy	56.8	27.3
Gabon	GAB	Hypertensive disorder case management	18.5	8.9
Gabon	GAB	Diabetes case management	14.5	7.0
Gabon	GAB	Malaria case management	59.8	28.8
Gabon	GAB	MgSO4 management of pre-eclampsia	36.7	17.7
Gabon	GAB	Thermal protection	89.2	45.2
Gabon	GAB	Clean cord care	86.1	43.6
Gabon	GAB	Clean birth environment	74.0	37.5
Gabon	GAB	Immediate drying and additional stimulation	82.6	41.8

Country	ISO 3166-1 alpha-3	Intervention Name	Baseline coverage (%)	Ending coverage (%)
Gabon	GAB	Neonatal resuscitation	49.6	25.1
Gabon	GAB	Antibiotics for preterm or prolonged PROM	67.5	34.2
Gabon	GAB	Parenteral administration of anti-convulsants	64.5	32.7
Gabon	GAB	Parenteral administration of uterotonics	80.6	40.8
Gabon	GAB	Parenteral administration of antibiotics	67.5	34.2
Gabon	GAB	Assisted vaginal delivery	22.8	11.5
Gabon	GAB	Manual removal of placenta	33.7	17.1
Gabon	GAB	Removal of retained products of conception	29.9	15.1
Gabon	GAB	Cesarean delivery	7.9	4.0
Gabon	GAB	Blood transfusion	11.4	5.8
Gabon	GAB	Induction of labor for pregnancies lasting 41+ weeks	1.6	0.8
Gabon	GAB	Complementary feeding - education only	29.9	17.3
Gabon	GAB	Complementary feeding - supplementary feeding and education	29.9	17.3
Gabon	GAB	Improved sanitation - Utilization of latrines or toilets	47.4	47.4
Gabon	GAB	Improved water source	85.8	85.8
Gabon	GAB	Water connection in the home	74.4	74.4
Gabon	GAB	ITN/IRS - Households protected from malaria	38.9	22.5
Gabon	GAB	Injectable antibiotics for neonatal sepsis	90.2	43.4
Gabon	GAB	ORS - oral rehydration solution	26.1	13.2
Gabon	GAB	Antibiotics for treatment of dysentery	0.5	0.3
Gabon	GAB	Oral antibiotics for pneumonia	49.5	25.1
Gabon	GAB	ACTs- Artemisinin compounds for treatment of malaria	4.4	2.2
Gabon	GAB	BCG vaccine	87.0	41.8
Gabon	GAB	Polio vaccine	64.0	30.8
Gabon	GAB	DPT vaccine	70.0	33.7
Gabon	GAB	H. influenzae type b vaccine	70.0	58.4
Gabon	GAB	HepB vaccine	70.0	33.7
Gabon	GAB	Measles vaccine	59.0	28.4
Gabon	GAB	Global wasting (<-2 SD) rate	3.4	5.1
Gabon	GAB	Contraceptive prevalence (CPR)	37.15	22.6
Gambia	GMB	TT - Tetanus toxoid vaccination	92.0	44.2
Gambia	GMB	IPTp - Intermittent preventive treatment of malaria during pregnancy	75.2	36.2
Gambia	GMB	Syphilis detection and treatment	24.5	11.8
Gambia	GMB	Iron supplementation in pregnancy	44.6	21.4
Gambia	GMB	Hypertensive disorder case management	18.2	8.8
Gambia	GMB	Diabetes case management	14.2	6.8
Gambia	GMB	Malaria case management	58.6	28.2
Gambia	GMB	MgSO4 management of pre-eclampsia	35.9	17.3
Gambia	GMB	Thermal protection	80.6	40.8
Gambia	GMB	Clean cord care	77.8	39.4

Country	ISO 3166-1 alpha-3	Intervention Name	Baseline coverage (%)	Ending coverage (%)
Gambia	GMB	Clean birth environment	66.8	33.8
Gambia	GMB	Immediate drying and additional stimulation	74.6	37.8
Gambia	GMB	Neonatal resuscitation	44.8	22.7
Gambia	GMB	Antibiotics for preterm or prolonged PROM	61.0	30.9
Gambia	GMB	Parenteral administration of anti-convulsants	58.3	29.5
Gambia	GMB	Parenteral administration of uterotonics	72.8	36.9
Gambia	GMB	Parenteral administration of antibiotics	61.0	30.9
Gambia	GMB	Assisted vaginal delivery	20.6	10.4
Gambia	GMB	Manual removal of placenta	30.5	15.4
Gambia	GMB	Removal of retained products of conception	27.0	13.7
Gambia	GMB	Cesarean delivery	7.1	3.6
Gambia	GMB	Blood transfusion	10.3	5.2
Gambia	GMB	Induction of labor for pregnancies lasting 41+ weeks	1.4	0.7
Gambia	GMB	Complementary feeding - education only	13.0	7.5
Gambia	GMB	Complementary feeding - supplementary feeding and education	13.0	7.5
Gambia	GMB	Vitamin A supplementation	32.0	18.5
Gambia	GMB	Improved sanitation - Utilization of latrines or toilets	39.2	39.2
Gambia	GMB	Improved water source	78.0	78.0
Gambia	GMB	Water connection in the home	56.1	56.1
Gambia	GMB	Hand washing with soap	6.3	6.3
Gambia	GMB	Hygienic disposal of children's stools	76.8	76.8
Gambia	GMB	ITN/IRS - Households protected from malaria	71.2	41.1
Gambia	GMB	Injectable antibiotics for neonatal sepsis	81.5	39.2
Gambia	GMB	ORS - oral rehydration solution	43.9	22.2
Gambia	GMB	Antibiotics for treatment of dysentery	35.7	18.1
Gambia	GMB	Zinc for treatment of diarrhea	23.1	11.7
Gambia	GMB	Oral antibiotics for pneumonia	68.0	34.4
Gambia	GMB	Vitamin A for treatment of measles	32.0	16.2
Gambia	GMB	ACTs- Artemisinin compounds for treatment of malaria	0.8	0.4
Gambia	GMB	SAM - treatment for severe acute malnutrition	25.1	12.7
Gambia	GMB	BCG vaccine	94.0	45.2
Gambia	GMB	Polio vaccine	93.0	44.7
Gambia	GMB	DPT vaccine	93.0	44.7
Gambia	GMB	H. influenzae type b vaccine	93.0	77.6
Gambia	GMB	HepB vaccine	93.0	44.7
Gambia	GMB	Pneumococcal vaccine	93.0	77.6
Gambia	GMB	Rotavirus vaccine	93.0	77.6
Gambia	GMB	Measles vaccine	91.0	43.8
Gambia	GMB	Global wasting (<-2 SD) rate	10.8	16.2
Gambia	GMB	Contraceptive prevalence (CPR)	14.25	8.7

Country	ISO 3166-1 alpha-3	Intervention Name	Baseline coverage (%)	Ending coverage (%)
Georgia	GEO	Safe abortion services	40.0	24.3
Georgia	GEO	Syphilis detection and treatment	20.8	10.0
Georgia	GEO	Hypertensive disorder case management	20.2	9.7
Georgia	GEO	Diabetes case management	15.8	7.6
Georgia	GEO	Malaria case management	65.3	31.4
Georgia	GEO	MgSO4 management of pre-eclampsia	40.0	19.2
Georgia	GEO	Thermal protection	98.2	49.7
Georgia	GEO	Clean cord care	94.9	48.0
Georgia	GEO	Clean birth environment	81.5	41.3
Georgia	GEO	Immediate drying and additional stimulation	91.0	46.1
Georgia	GEO	Neonatal resuscitation	54.7	27.7
Georgia	GEO	Antibiotics for preterm or prolonged PROM	74.4	37.7
Georgia	GEO	Parenteral administration of anti-convulsants	71.1	36.0
Georgia	GEO	Parenteral administration of uterotonics	88.8	45.0
Georgia	GEO	Parenteral administration of antibiotics	74.4	37.7
Georgia	GEO	Assisted vaginal delivery	25.1	12.7
Georgia	GEO	Manual removal of placenta	37.2	18.8
Georgia	GEO	Removal of retained products of conception	32.9	16.7
Georgia	GEO	Cesarean delivery	8.7	4.4
Georgia	GEO	Blood transfusion	12.5	6.3
Georgia	GEO	Induction of labor for pregnancies lasting 41+ weeks	1.7	0.9
Georgia	GEO	Complementary feeding - education only	49.9	28.8
Georgia	GEO	Complementary feeding - supplementary feeding and education	49.9	28.8
Georgia	GEO	Improved sanitation - Utilization of latrines or toilets	90.0	90.0
Georgia	GEO	Improved water source	98.4	98.4
Georgia	GEO	Water connection in the home	79.1	79.1
Georgia	GEO	Hygienic disposal of children's stools	57.1	57.1
Georgia	GEO	Injectable antibiotics for neonatal sepsis	99.4	47.8
Georgia	GEO	ORS - oral rehydration solution	42.4	21.5
Georgia	GEO	Zinc for treatment of diarrhea	10.9	5.5
Georgia	GEO	Oral antibiotics for pneumonia	73.6	37.3
Georgia	GEO	BCG vaccine	97.0	46.7
Georgia	GEO	Polio vaccine	93.0	44.7
Georgia	GEO	DPT vaccine	93.0	44.7
Georgia	GEO	H. influenzae type b vaccine	93.0	77.6
Georgia	GEO	HepB vaccine	93.0	44.7
Georgia	GEO	Pneumococcal vaccine	81.0	67.5
Georgia	GEO	Rotavirus vaccine	79.0	65.9
Georgia	GEO	Measles vaccine	98.0	47.1
Georgia	GEO	Global wasting (<-2 SD) rate	3.0	4.5

Country	ISO 3166-1 alpha-3	Intervention Name	Baseline coverage (%)	Ending coverage (%)
Georgia	GEO	Contraceptive prevalence (CPR)	47.3	28.7
Ghana	GHA	TT - Tetanus toxoid vaccination	89.0	42.8
Ghana	GHA	IPTp - Intermittent preventive treatment of malaria during pregnancy	78.0	37.5
Ghana	GHA	Syphilis detection and treatment	24.1	11.6
Ghana	GHA	Iron supplementation in pregnancy	59.4	28.6
Ghana	GHA	Hypertensive disorder case management	21.5	10.3
Ghana	GHA	Diabetes case management	16.7	8.0
Ghana	GHA	Malaria case management	69.2	33.3
Ghana	GHA	MgSO4 management of pre-eclampsia	42.4	20.4
Ghana	GHA	Thermal protection	77.8	39.4
Ghana	GHA	Clean cord care	75.1	38.0
Ghana	GHA	Clean birth environment	64.5	32.7
Ghana	GHA	Immediate drying and additional stimulation	72.1	36.5
Ghana	GHA	Neonatal resuscitation	43.3	21.9
Ghana	GHA	Antibiotics for preterm or prolonged PROM	58.9	29.8
Ghana	GHA	Parenteral administration of anti-convulsants	56.3	28.5
Ghana	GHA	Parenteral administration of uterotonics	70.3	35.6
Ghana	GHA	Parenteral administration of antibiotics	58.9	29.8
Ghana	GHA	Assisted vaginal delivery	19.9	10.1
Ghana	GHA	Manual removal of placenta	29.4	14.9
Ghana	GHA	Removal of retained products of conception	26.1	13.2
Ghana	GHA	Cesarean delivery	6.8	3.4
Ghana	GHA	Blood transfusion	9.9	5.0
Ghana	GHA	Induction of labor for pregnancies lasting 41+ weeks	1.4	0.7
Ghana	GHA	Complementary feeding - education only	28.1	16.2
Ghana	GHA	Complementary feeding - supplementary feeding and education	28.1	16.2
Ghana	GHA	Vitamin A supplementation	50.0	28.9
Ghana	GHA	Improved sanitation - Utilization of latrines or toilets	18.5	18.5
Ghana	GHA	Improved water source	81.5	81.5
Ghana	GHA	Water connection in the home	26.6	26.6
Ghana	GHA	Hand washing with soap	23.6	23.6
Ghana	GHA	Hygienic disposal of children's stools	24.5	24.5
Ghana	GHA	ITN/IRS - Households protected from malaria	73.0	42.1
Ghana	GHA	Injectable antibiotics for neonatal sepsis	78.7	37.8
Ghana	GHA	ORS - oral rehydration solution	48.6	24.6
Ghana	GHA	Antibiotics for treatment of dysentery	42.4	21.5
Ghana	GHA	Zinc for treatment of diarrhea	7.4	3.7
Ghana	GHA	Oral antibiotics for pneumonia	52.6	26.6
Ghana	GHA	Vitamin A for treatment of measles	50.0	25.3
Ghana	GHA	ACTs- Artemisinin compounds for treatment of malaria	26.2	13.3

**Country**	**ISO 3166-1 alpha-3**	**Intervention Name**	**Baseline coverage (%)**	**Ending coverage (%)**
Ghana	GHA	BCG vaccine	98.0	47.1
Ghana	GHA	Polio vaccine	98.0	47.1
Ghana	GHA	DPT vaccine	97.0	46.7
Ghana	GHA	H. influenzae type b vaccine	97.0	80.9
Ghana	GHA	HepB vaccine	97.0	46.7
Ghana	GHA	Pneumococcal vaccine	96.0	80.1
Ghana	GHA	Rotavirus vaccine	94.0	78.4
Ghana	GHA	Meningococcal A	83.0	39.9
Ghana	GHA	Measles vaccine	92.0	44.2
Ghana	GHA	Global wasting (<-2 SD) rate	4.7	7.0
Ghana	GHA	Contraceptive prevalence (CPR)	32.5	19.7
Guatemala	GTM	Safe abortion services	0.6	0.4
Guatemala	GTM	TT - Tetanus toxoid vaccination	90.0	43.3
Guatemala	GTM	Syphilis detection and treatment	22.6	10.9
Guatemala	GTM	Iron supplementation in pregnancy	29.3	14.1
Guatemala	GTM	Hypertensive disorder case management	20.6	9.9
Guatemala	GTM	Diabetes case management	16.1	7.7
Guatemala	GTM	Malaria case management	66.6	32.0
Guatemala	GTM	MgSO4 management of pre-eclampsia	40.8	19.6
Guatemala	GTM	Thermal protection	64.2	32.5
Guatemala	GTM	Clean cord care	62.0	31.4
Guatemala	GTM	Clean birth environment	53.3	27.0
Guatemala	GTM	Immediate drying and additional stimulation	59.5	30.1
Guatemala	GTM	Neonatal resuscitation	35.7	18.1
Guatemala	GTM	Antibiotics for preterm or prolonged PROM	48.6	24.6
Guatemala	GTM	Parenteral administration of anti-convulsants	46.5	23.5
Guatemala	GTM	Parenteral administration of uterotonics	58.1	29.4
Guatemala	GTM	Parenteral administration of antibiotics	48.6	24.6
Guatemala	GTM	Assisted vaginal delivery	16.4	8.3
Guatemala	GTM	Manual removal of placenta	24.3	12.3
Guatemala	GTM	Removal of retained products of conception	21.5	10.9
Guatemala	GTM	Cesarean delivery	5.7	2.9
Guatemala	GTM	Blood transfusion	8.2	4.2
Guatemala	GTM	Induction of labor for pregnancies lasting 41+ weeks	1.1	0.6
Guatemala	GTM	Complementary feeding - education only	62.6	36.1
Guatemala	GTM	Complementary feeding - supplementary feeding and education	62.6	36.1
Guatemala	GTM	Vitamin A supplementation	26.0	15.0
Guatemala	GTM	Improved sanitation - Utilization of latrines or toilets	65.1	65.1
Guatemala	GTM	Improved water source	94.2	94.2
Guatemala	GTM	Water connection in the home	75.5	75.5

Country	ISO 3166-1 alpha-3	Intervention Name	Baseline coverage (%)	Ending coverage (%)
Guatemala	GTM	Hand washing with soap	78.5	78.5
Guatemala	GTM	Hygienic disposal of children's stools	38.2	38.2
Guatemala	GTM	Injectable antibiotics for neonatal sepsis	65.0	31.3
Guatemala	GTM	ORS - oral rehydration solution	48.8	24.7
Guatemala	GTM	Antibiotics for treatment of dysentery	7.0	3.5
Guatemala	GTM	Zinc for treatment of diarrhea	1.3	0.7
Guatemala	GTM	Oral antibiotics for pneumonia	52.0	26.3
Guatemala	GTM	Vitamin A for treatment of measles	26.0	13.2
Guatemala	GTM	SAM - treatment for severe acute malnutrition	1.4	0.7
Guatemala	GTM	BCG vaccine	88.0	42.3
Guatemala	GTM	Polio vaccine	85.0	40.9
Guatemala	GTM	DPT vaccine	86.0	41.4
Guatemala	GTM	H. influenzae type b vaccine	86.0	71.7
Guatemala	GTM	HepB vaccine	86.0	41.4
Guatemala	GTM	Pneumococcal vaccine	85.0	70.9
Guatemala	GTM	Rotavirus vaccine	87.0	72.5
Guatemala	GTM	Measles vaccine	86.0	41.4
Guatemala	GTM	Global wasting (<-2 SD) rate	0.8	1.1
Guatemala	GTM	Contraceptive prevalence (CPR)	63.55	38.6
Guinea	GIN	TT - Tetanus toxoid vaccination	80.0	38.5
Guinea	GIN	IPTp - Intermittent preventive treatment of malaria during pregnancy	62.7	30.2
Guinea	GIN	Syphilis detection and treatment	20.6	9.9
Guinea	GIN	Iron supplementation in pregnancy	41.5	20.0
Guinea	GIN	Hypertensive disorder case management	8.5	4.1
Guinea	GIN	Diabetes case management	6.6	3.2
Guinea	GIN	Malaria case management	27.4	13.2
Guinea	GIN	MgSO4 management of pre-eclampsia	16.8	8.1
Guinea	GIN	Thermal protection	52.0	26.3
Guinea	GIN	Clean cord care	50.2	25.4
Guinea	GIN	Clean birth environment	43.1	21.8
Guinea	GIN	Immediate drying and additional stimulation	48.2	24.4
Guinea	GIN	Neonatal resuscitation	28.9	14.6
Guinea	GIN	Antibiotics for preterm or prolonged PROM	39.4	19.9
Guinea	GIN	Parenteral administration of anti-convulsants	37.6	19.0
Guinea	GIN	Parenteral administration of uterotonics	47.0	23.8
Guinea	GIN	Parenteral administration of antibiotics	39.4	19.9
Guinea	GIN	Assisted vaginal delivery	13.3	6.7
Guinea	GIN	Manual removal of placenta	19.7	10.0
Guinea	GIN	Removal of retained products of conception	17.4	8.8
Guinea	GIN	Cesarean delivery	4.6	2.3

Country	ISO 3166-1 alpha-3	Intervention Name	Baseline coverage (%)	Ending coverage (%)
Guinea	GIN	Blood transfusion	6.6	3.3
Guinea	GIN	Induction of labor for pregnancies lasting 41+ weeks	0.9	0.5
Guinea	GIN	Complementary feeding - education only	15.9	9.2
Guinea	GIN	Complementary feeding - supplementary feeding and education	15.9	9.2
Guinea	GIN	Vitamin A supplementation	64.0	36.9
Guinea	GIN	Improved sanitation - Utilization of latrines or toilets	22.7	22.7
Guinea	GIN	Improved water source	61.9	61.9
Guinea	GIN	Water connection in the home	17.4	17.4
Guinea	GIN	Hand washing with soap	20.9	20.9
Guinea	GIN	Hygienic disposal of children's stools	57.9	57.9
Guinea	GIN	ITN/IRS - Households protected from malaria	43.9	25.3
Guinea	GIN	Injectable antibiotics for neonatal sepsis	52.6	25.3
Guinea	GIN	ORS - oral rehydration solution	54.9	27.8
Guinea	GIN	Antibiotics for treatment of dysentery	1.5	0.8
Guinea	GIN	Zinc for treatment of diarrhea	26.0	13.2
Guinea	GIN	Oral antibiotics for pneumonia	30.1	15.2
Guinea	GIN	Vitamin A for treatment of measles	64.0	32.4
Guinea	GIN	ACTs- Artemisinin compounds for treatment of malaria	2.1	1.1
Guinea	GIN	SAM - treatment for severe acute malnutrition	13.1	6.6
Guinea	GIN	BCG vaccine	72.0	34.6
Guinea	GIN	Polio vaccine	45.0	21.6
Guinea	GIN	DPT vaccine	45.0	21.6
Guinea	GIN	H. influenzae type b vaccine	45.0	37.5
Guinea	GIN	HepB vaccine	45.0	21.6
Guinea	GIN	Measles vaccine	48.0	23.1
Guinea	GIN	Global wasting (<-2 SD) rate	8.2	12.3
Guinea	GIN	Contraceptive prevalence (CPR)	10.3	6.3
Guinea-Bissau	GNB	TT - Tetanus toxoid vaccination	83.0	39.9
Guinea-Bissau	GNB	IPTp - Intermittent preventive treatment of malaria during pregnancy	45.5	21.9
Guinea-Bissau	GNB	Syphilis detection and treatment	22.9	11.0
Guinea-Bissau	GNB	Hypertensive disorder case management	15.6	7.5
Guinea-Bissau	GNB	Diabetes case management	12.2	5.9
Guinea-Bissau	GNB	Malaria case management	50.3	24.2
Guinea-Bissau	GNB	MgSO4 management of pre-eclampsia	30.8	14.8
Guinea-Bissau	GNB	Thermal protection	43.5	22.0
Guinea-Bissau	GNB	Clean cord care	42.0	21.3
Guinea-Bissau	GNB	Clean birth environment	36.1	18.3
Guinea-Bissau	GNB	Immediate drying and additional stimulation	40.3	20.4
Guinea-Bissau	GNB	Neonatal resuscitation	24.2	12.3
Guinea-Bissau	GNB	Antibiotics for preterm or prolonged PROM	32.9	16.7

Country	ISO 3166-1 alpha-3	Intervention Name	Baseline coverage (%)	Ending coverage (%)
Guinea-Bissau	GNB	Parenteral administration of anti-convulsants	31.4	15.9
Guinea-Bissau	GNB	Parenteral administration of uterotonics	39.3	19.9
Guinea-Bissau	GNB	Parenteral administration of antibiotics	32.9	16.7
Guinea-Bissau	GNB	Assisted vaginal delivery	11.1	5.6
Guinea-Bissau	GNB	Manual removal of placenta	16.4	8.3
Guinea-Bissau	GNB	Removal of retained products of conception	14.6	7.4
Guinea-Bissau	GNB	Cesarean delivery	3.8	1.9
Guinea-Bissau	GNB	Blood transfusion	5.5	2.8
Guinea-Bissau	GNB	Induction of labor for pregnancies lasting 41+ weeks	0.8	0.4
Guinea-Bissau	GNB	Complementary feeding - education only	12.7	7.3
Guinea-Bissau	GNB	Complementary feeding - supplementary feeding and education	12.7	7.3
Guinea-Bissau	GNB	Vitamin A supplementation	95.0	54.8
Guinea-Bissau	GNB	Improved sanitation - Utilization of latrines or toilets	20.5	20.5
Guinea-Bissau	GNB	Improved water source	66.6	66.6
Guinea-Bissau	GNB	Water connection in the home	11.9	11.9
Guinea-Bissau	GNB	Hand washing with soap	10.6	10.6
Guinea-Bissau	GNB	Hygienic disposal of children's stools	62.6	62.6
Guinea-Bissau	GNB	ITN/IRS - Households protected from malaria	90.1	52.0
Guinea-Bissau	GNB	Injectable antibiotics for neonatal sepsis	44.0	21.2
Guinea-Bissau	GNB	ORS - oral rehydration solution	35.1	17.8
Guinea-Bissau	GNB	Zinc for treatment of diarrhea	27.8	14.1
Guinea-Bissau	GNB	Oral antibiotics for pneumonia	34.3	17.4
Guinea-Bissau	GNB	Vitamin A for treatment of measles	95.0	48.1
Guinea-Bissau	GNB	ACTs- Artemisinin compounds for treatment of malaria	10.0	5.1
Guinea-Bissau	GNB	SAM - treatment for severe acute malnutrition	2.7	1.4
Guinea-Bissau	GNB	BCG vaccine	91.0	43.8
Guinea-Bissau	GNB	Polio vaccine	89.0	42.8
Guinea-Bissau	GNB	DPT vaccine	88.0	42.3
Guinea-Bissau	GNB	H. influenzae type b vaccine	88.0	73.4
Guinea-Bissau	GNB	HepB vaccine	88.0	42.3
Guinea-Bissau	GNB	Pneumococcal vaccine	88.0	73.4
Guinea-Bissau	GNB	Rotavirus vaccine	88.0	73.4
Guinea-Bissau	GNB	Measles vaccine	86.0	41.4
Guinea-Bissau	GNB	Global wasting (<-2 SD) rate	5.9	8.9
Guinea-Bissau	GNB	Contraceptive prevalence (CPR)	19.15	11.6
Guyana	GUY	Safe abortion services	0.2	0.1
Guyana	GUY	TT - Tetanus toxoid vaccination	99.0	47.6
Guyana	GUY	Syphilis detection and treatment	22.4	10.8
Guyana	GUY	Iron supplementation in pregnancy	34.2	16.4
Guyana	GUY	Hypertensive disorder case management	20.8	10.0

Country	ISO 3166-1 alpha-3	Intervention Name	Baseline coverage (%)	Ending coverage (%)
Guyana	GUY	Diabetes case management	16.2	7.8
Guyana	GUY	Malaria case management	67.2	32.3
Guyana	GUY	MgSO4 management of pre-eclampsia	41.2	19.8
Guyana	GUY	Thermal protection	91.4	46.3
Guyana	GUY	Clean cord care	88.2	44.7
Guyana	GUY	Clean birth environment	75.8	38.4
Guyana	GUY	Immediate drying and additional stimulation	84.6	42.8
Guyana	GUY	Neonatal resuscitation	50.8	25.7
Guyana	GUY	Antibiotics for preterm or prolonged PROM	69.2	35.0
Guyana	GUY	Parenteral administration of anti-convulsants	66.1	33.5
Guyana	GUY	Parenteral administration of uterotonics	82.6	41.8
Guyana	GUY	Parenteral administration of antibiotics	69.2	35.0
Guyana	GUY	Assisted vaginal delivery	23.4	11.8
Guyana	GUY	Manual removal of placenta	34.6	17.5
Guyana	GUY	Removal of retained products of conception	30.6	15.5
Guyana	GUY	Cesarean delivery	8.0	4.1
Guyana	GUY	Blood transfusion	11.6	5.9
Guyana	GUY	Induction of labor for pregnancies lasting 41+ weeks	1.6	0.8
Guyana	GUY	Complementary feeding - education only	52.6	30.4
Guyana	GUY	Complementary feeding - supplementary feeding and education	52.6	30.4
Guyana	GUY	Improved sanitation - Utilization of latrines or toilets	85.8	85.8
Guyana	GUY	Improved water source	95.5	95.5
Guyana	GUY	Water connection in the home	62.4	62.4
Guyana	GUY	Hand washing with soap	78.8	78.8
Guyana	GUY	Hygienic disposal of children's stools	43.0	43.0
Guyana	GUY	ITN/IRS - Households protected from malaria	5.3	3.1
Guyana	GUY	Injectable antibiotics for neonatal sepsis	92.4	44.4
Guyana	GUY	ORS - oral rehydration solution	42.5	21.5
Guyana	GUY	Antibiotics for treatment of dysentery	12.2	6.2
Guyana	GUY	Zinc for treatment of diarrhea	1.3	0.7
Guyana	GUY	Oral antibiotics for pneumonia	83.6	42.3
Guyana	GUY	BCG vaccine	99.0	47.6
Guyana	GUY	Polio vaccine	94.0	45.2
Guyana	GUY	DPT vaccine	95.0	45.7
Guyana	GUY	H. influenzae type b vaccine	95.0	79.2
Guyana	GUY	HepB vaccine	95.0	45.7
Guyana	GUY	Pneumococcal vaccine	91.0	75.9
Guyana	GUY	Rotavirus vaccine	91.0	75.9
Guyana	GUY	Measles vaccine	98.0	47.1
Guyana	GUY	Global wasting (<-2 SD) rate	6.4	9.6

**Country**	**ISO 3166-1 alpha-3**	**Intervention Name**	**Baseline coverage (%)**	**Ending coverage (%)**
Guyana	GUY	Contraceptive prevalence (CPR)	41.95	25.5
Haiti	HTI	Safe abortion services	53.7	32.6
Haiti	HTI	TT - Tetanus toxoid vaccination	81.0	39.0
Haiti	HTI	IPTp - Intermittent preventive treatment of malaria during pregnancy	0.2	0.1
Haiti	HTI	Syphilis detection and treatment	19.4	9.3
Haiti	HTI	Iron supplementation in pregnancy	43.2	20.8
Haiti	HTI	Hypertensive disorder case management	15.5	7.5
Haiti	HTI	Diabetes case management	15.0	7.2
Haiti	HTI	Malaria case management	32.8	15.8
Haiti	HTI	MgSO4 management of pre-eclampsia	17.1	8.2
Haiti	HTI	Thermal protection	37.8	19.1
Haiti	HTI	Clean cord care	38.2	19.3
Haiti	HTI	Clean birth environment	33.8	17.1
Haiti	HTI	Immediate drying and additional stimulation	32.6	16.5
Haiti	HTI	Neonatal resuscitation	14.9	7.5
Haiti	HTI	Antibiotics for preterm or prolonged PROM	21.4	10.8
Haiti	HTI	Parenteral administration of anti-convulsants	28.2	14.3
Haiti	HTI	Parenteral administration of uterotonics	25.5	12.9
Haiti	HTI	Parenteral administration of antibiotics	21.4	10.8
Haiti	HTI	Assisted vaginal delivery	7.7	3.9
Haiti	HTI	Manual removal of placenta	14.8	7.5
Haiti	HTI	Removal of retained products of conception	11.0	5.6
Haiti	HTI	Cesarean delivery	9.4	4.8
Haiti	HTI	Blood transfusion	5.0	2.5
Haiti	HTI	Induction of labor for pregnancies lasting 41+ weeks	0.6	0.3
Haiti	HTI	Complementary feeding - education only	25.4	14.7
Haiti	HTI	Complementary feeding - supplementary feeding and education	25.4	14.7
Haiti	HTI	Vitamin A supplementation	17.0	9.8
Haiti	HTI	Improved sanitation - Utilization of latrines or toilets	34.7	34.7
Haiti	HTI	Improved water source	65.5	65.5
Haiti	HTI	Water connection in the home	14.8	14.8
Haiti	HTI	Hand washing with soap	21.5	21.5
Haiti	HTI	Hygienic disposal of children's stools	63.9	63.9
Haiti	HTI	ITN/IRS - Households protected from malaria	32.0	18.5
Haiti	HTI	Injectable antibiotics for neonatal sepsis	39.4	18.9
Haiti	HTI	ORS - oral rehydration solution	39.3	19.9
Haiti	HTI	Antibiotics for treatment of dysentery	16.1	8.2
Haiti	HTI	Zinc for treatment of diarrhea	6.2	3.1
Haiti	HTI	Oral antibiotics for pneumonia	39.4	19.9
Haiti	HTI	Vitamin A for treatment of measles	17.0	8.6

Country	ISO 3166-1 alpha-3	Intervention Name	Baseline coverage (%)	Ending coverage (%)
Haiti	HTI	SAM - treatment for severe acute malnutrition	18.1	9.2
Haiti	HTI	BCG vaccine	83.0	39.9
Haiti	HTI	Polio vaccine	64.0	30.8
Haiti	HTI	DPT vaccine	64.0	30.8
Haiti	HTI	H. influenzae type b vaccine	64.0	53.4
Haiti	HTI	HepB vaccine	64.0	30.8
Haiti	HTI	Pneumococcal vaccine	1.0	0.8
Haiti	HTI	Rotavirus vaccine	58.0	48.4
Haiti	HTI	Measles vaccine	69.0	33.2
Haiti	HTI	Global wasting (<-2 SD) rate	3.7	5.6
Haiti	HTI	Contraceptive prevalence (CPR)	37.5	22.8
Honduras	HND	Safe abortion services	0.6	0.4
Honduras	HND	TT - Tetanus toxoid vaccination	99.0	47.6
Honduras	HND	Syphilis detection and treatment	23.9	11.5
Honduras	HND	Iron supplementation in pregnancy	37.4	18.0
Honduras	HND	Hypertensive disorder case management	21.2	10.2
Honduras	HND	Diabetes case management	16.6	8.0
Honduras	HND	Malaria case management	68.5	32.9
Honduras	HND	MgSO4 management of pre-eclampsia	42.0	20.2
Honduras	HND	Thermal protection	81.8	41.4
Honduras	HND	Clean cord care	79.0	40.0
Honduras	HND	Clean birth environment	67.9	34.4
Honduras	HND	Immediate drying and additional stimulation	75.8	38.4
Honduras	HND	Neonatal resuscitation	45.5	23.0
Honduras	HND	Antibiotics for preterm or prolonged PROM	61.9	31.3
Honduras	HND	Parenteral administration of anti-convulsants	59.2	30.0
Honduras	HND	Parenteral administration of uterotonics	73.9	37.4
Honduras	HND	Parenteral administration of antibiotics	61.9	31.3
Honduras	HND	Assisted vaginal delivery	20.9	10.6
Honduras	HND	Manual removal of placenta	30.9	15.6
Honduras	HND	Removal of retained products of conception	27.4	13.9
Honduras	HND	Cesarean delivery	7.2	3.6
Honduras	HND	Blood transfusion	10.4	5.3
Honduras	HND	Induction of labor for pregnancies lasting 41+ weeks	1.5	0.8
Honduras	HND	Complementary feeding - education only	68.2	39.4
Honduras	HND	Complementary feeding - supplementary feeding and education	68.2	39.4
Honduras	HND	Vitamin A supplementation	40.0	23.1
Honduras	HND	Improved sanitation - Utilization of latrines or toilets	81.3	81.3
Honduras	HND	Improved water source	94.8	94.8
Honduras	HND	Water connection in the home	85.5	85.5

Country	ISO 3166-1 alpha-3	Intervention Name	Baseline coverage (%)	Ending coverage (%)
Honduras	HND	Hand washing with soap	86.3	86.3
Honduras	HND	Hygienic disposal of children's stools	16.1	16.1
Honduras	HND	Injectable antibiotics for neonatal sepsis	82.7	39.8
Honduras	HND	ORS - oral rehydration solution	59.7	30.2
Honduras	HND	Antibiotics for treatment of dysentery	43.6	22.1
Honduras	HND	Zinc for treatment of diarrhea	0.4	0.2
Honduras	HND	Oral antibiotics for pneumonia	63.9	32.3
Honduras	HND	Vitamin A for treatment of measles	40.0	20.3
Honduras	HND	BCG vaccine	94.0	45.2
Honduras	HND	Polio vaccine	90.0	43.3
Honduras	HND	DPT vaccine	90.0	43.3
Honduras	HND	H. influenzae type b vaccine	90.0	75.1
Honduras	HND	HepB vaccine	90.0	43.3
Honduras	HND	Pneumococcal vaccine	90.0	75.1
Honduras	HND	Rotavirus vaccine	91.0	75.9
Honduras	HND	Measles vaccine	89.0	42.8
Honduras	HND	Global wasting (<-2 SD) rate	1.4	2.0
Honduras	HND	Contraceptive prevalence (CPR)	74.9	45.5
India	IND	Safe abortion services	35.1	21.3
India	IND	TT - Tetanus toxoid vaccination	90.0	43.3
India	IND	Syphilis detection and treatment	19.7	9.5
India	IND	Iron supplementation in pregnancy	38.8	18.7
India	IND	Hypertensive disorder case management	12.2	5.9
India	IND	Diabetes case management	9.5	4.6
India	IND	Malaria case management	39.3	18.9
India	IND	MgSO4 management of pre-eclampsia	24.1	11.6
India	IND	Thermal protection	78.0	39.5
India	IND	Clean cord care	75.3	38.1
India	IND	Clean birth environment	64.7	32.8
India	IND	Immediate drying and additional stimulation	72.3	36.6
India	IND	Neonatal resuscitation	43.4	22.0
India	IND	Antibiotics for preterm or prolonged PROM	59.1	29.9
India	IND	Parenteral administration of anti-convulsants	56.4	28.6
India	IND	Parenteral administration of uterotonics	70.5	35.7
India	IND	Parenteral administration of antibiotics	59.1	29.9
India	IND	Assisted vaginal delivery	20.0	10.1
India	IND	Manual removal of placenta	29.5	14.9
India	IND	Removal of retained products of conception	26.2	13.3
India	IND	Cesarean delivery	6.9	3.5
India	IND	Blood transfusion	9.9	5.0
India	IND	Induction of labor for pregnancies lasting 41+ weeks	1.4	0.7

Country	ISO 3166-1 alpha-3	Intervention Name	Baseline coverage (%)	Ending coverage (%)
India	IND	Complementary feeding - education only	22.0	12.7
India	IND	Complementary feeding - supplementary feeding and education	22.0	12.7
India	IND	Vitamin A supplementation	71.0	41.0
India	IND	Improved sanitation - Utilization of latrines or toilets	59.5	59.5
India	IND	Improved water source	92.7	92.7
India	IND	Water connection in the home	40.5	40.5
India	IND	Hand washing with soap	67.6	67.6
India	IND	Hygienic disposal of children's stools	26.0	26.0
India	IND	Injectable antibiotics for neonatal sepsis	78.9	37.9
India	IND	ORS - oral rehydration solution	50.6	25.6
India	IND	Antibiotics for treatment of dysentery	21.8	11.0
India	IND	Zinc for treatment of diarrhea	20.3	10.3
India	IND	Oral antibiotics for pneumonia	78.1	39.5
India	IND	Vitamin A for treatment of measles	71.0	35.9
India	IND	ACTs- Artemisinin compounds for treatment of malaria	1.6	0.8
India	IND	BCG vaccine	92.0	44.2
India	IND	Polio vaccine	89.0	42.8
India	IND	DPT vaccine	89.0	42.8
India	IND	H. influenzae type b vaccine	89.0	74.2
India	IND	HepB vaccine	89.0	42.8
India	IND	Pneumococcal vaccine	6.0	5.0
India	IND	Rotavirus vaccine	35.0	29.2
India	IND	Measles vaccine	90.0	43.3
India	IND	Global wasting (<-2 SD) rate	21.0	31.5
India	IND	Contraceptive prevalence (CPR)	56.65	34.4
Indonesia	IDN	Safe abortion services	39.0	23.7
Indonesia	IDN	TT - Tetanus toxoid vaccination	90.0	43.3
Indonesia	IDN	Syphilis detection and treatment	23.7	11.4
Indonesia	IDN	Iron supplementation in pregnancy	32.9	15.8
Indonesia	IDN	Hypertensive disorder case management	21.0	10.1
Indonesia	IDN	Diabetes case management	16.4	7.9
Indonesia	IDN	Malaria case management	67.7	32.6
Indonesia	IDN	MgSO4 management of pre-eclampsia	41.5	20.0
Indonesia	IDN	Thermal protection	62.9	31.8
Indonesia	IDN	Clean cord care	60.7	30.7
Indonesia	IDN	Clean birth environment	52.2	26.4
Indonesia	IDN	Immediate drying and additional stimulation	58.2	29.5
Indonesia	IDN	Neonatal resuscitation	35.0	17.7
Indonesia	IDN	Antibiotics for preterm or prolonged PROM	47.6	24.1
Indonesia	IDN	Parenteral administration of anti-convulsants	45.5	23.0

Country	ISO 3166-1 alpha-3	Intervention Name	Baseline coverage (%)	Ending coverage (%)
Indonesia	IDN	Parenteral administration of uterotonics	56.8	28.8
Indonesia	IDN	Parenteral administration of antibiotics	47.6	24.1
Indonesia	IDN	Assisted vaginal delivery	16.1	8.2
Indonesia	IDN	Manual removal of placenta	23.8	12.0
Indonesia	IDN	Removal of retained products of conception	21.1	10.7
Indonesia	IDN	Cesarean delivery	5.5	2.8
Indonesia	IDN	Blood transfusion	8.0	4.1
Indonesia	IDN	Induction of labor for pregnancies lasting 41+ weeks	1.1	0.6
Indonesia	IDN	Complementary feeding - education only	58.2	33.6
Indonesia	IDN	Complementary feeding - supplementary feeding and education	58.2	33.6
Indonesia	IDN	Vitamin A supplementation	62.0	35.8
Indonesia	IDN	Improved sanitation - Utilization of latrines or toilets	73.1	73.1
Indonesia	IDN	Improved water source	89.3	89.3
Indonesia	IDN	Water connection in the home	16.3	16.3
Indonesia	IDN	Hand washing with soap	75.6	75.6
Indonesia	IDN	Hygienic disposal of children's stools	43.7	43.7
Indonesia	IDN	ITN/IRS - Households protected from malaria	3.1	1.8
Indonesia	IDN	Injectable antibiotics for neonatal sepsis	63.6	30.6
Indonesia	IDN	ORS - oral rehydration solution	38.8	19.6
Indonesia	IDN	Antibiotics for treatment of dysentery	19.7	10.0
Indonesia	IDN	Zinc for treatment of diarrhea	1.1	0.6
Indonesia	IDN	Oral antibiotics for pneumonia	75.3	38.1
Indonesia	IDN	Vitamin A for treatment of measles	62.0	31.4
Indonesia	IDN	BCG vaccine	81.0	39.0
Indonesia	IDN	Polio vaccine	80.0	38.5
Indonesia	IDN	DPT vaccine	79.0	38.0
Indonesia	IDN	H. influenzae type b vaccine	79.0	65.9
Indonesia	IDN	HepB vaccine	79.0	38.0
Indonesia	IDN	Pneumococcal vaccine	8.0	6.7
Indonesia	IDN	Measles vaccine	75.0	36.1
Indonesia	IDN	Global wasting (<-2 SD) rate	10.6	15.9
Indonesia	IDN	Contraceptive prevalence (CPR)	63.2	38.4
Iraq	IRQ	Safe abortion services	40.0	24.3
Iraq	IRQ	TT - Tetanus toxoid vaccination	75.0	36.1
Iraq	IRQ	Syphilis detection and treatment	21.7	10.4
Iraq	IRQ	Hypertensive disorder case management	16.3	7.8
Iraq	IRQ	Diabetes case management	12.7	6.1
Iraq	IRQ	Malaria case management	52.7	25.3
Iraq	IRQ	MgSO4 management of pre-eclampsia	32.3	15.5
Iraq	IRQ	Thermal protection	85.6	43.3

Country	ISO 3166-1 alpha-3	Intervention Name	Baseline coverage (%)	Ending coverage (%)
Iraq	IRQ	Clean cord care	82.6	41.8
Iraq	IRQ	Clean birth environment	71.0	35.9
Iraq	IRQ	Immediate drying and additional stimulation	79.3	40.1
Iraq	IRQ	Neonatal resuscitation	47.6	24.1
Iraq	IRQ	Antibiotics for preterm or prolonged PROM	64.8	32.8
Iraq	IRQ	Parenteral administration of anti-convulsants	61.9	31.3
Iraq	IRQ	Parenteral administration of uterotonics	77.4	39.2
Iraq	IRQ	Parenteral administration of antibiotics	64.8	32.8
Iraq	IRQ	Assisted vaginal delivery	21.9	11.1
Iraq	IRQ	Manual removal of placenta	32.4	16.4
Iraq	IRQ	Removal of retained products of conception	28.7	14.5
Iraq	IRQ	Cesarean delivery	7.5	3.8
Iraq	IRQ	Blood transfusion	10.9	5.5
Iraq	IRQ	Induction of labor for pregnancies lasting 41+ weeks	1.5	0.8
Iraq	IRQ	Complementary feeding - education only	60.1	34.7
Iraq	IRQ	Complementary feeding - supplementary feeding and education	60.1	34.7
Iraq	IRQ	Improved sanitation - Utilization of latrines or toilets	94.1	94.1
Iraq	IRQ	Improved water source	96.5	96.5
Iraq	IRQ	Water connection in the home	76.8	76.8
Iraq	IRQ	Hand washing with soap	97.7	97.7
Iraq	IRQ	Hygienic disposal of children's stools	15.8	15.8
Iraq	IRQ	Injectable antibiotics for neonatal sepsis	86.6	41.6
Iraq	IRQ	ORS - oral rehydration solution	25.4	12.9
Iraq	IRQ	Zinc for treatment of diarrhea	7.4	3.7
Iraq	IRQ	Oral antibiotics for pneumonia	40.3	20.4
Iraq	IRQ	BCG vaccine	95.0	45.7
Iraq	IRQ	Polio vaccine	71.0	34.1
Iraq	IRQ	DPT vaccine	84.0	40.4
Iraq	IRQ	H. influenzae type b vaccine	84.0	70.0
Iraq	IRQ	HepB vaccine	84.0	40.4
Iraq	IRQ	Pneumococcal vaccine	32.0	26.7
Iraq	IRQ	Rotavirus vaccine	60.0	50.0
Iraq	IRQ	Measles vaccine	83.0	39.9
Iraq	IRQ	Global wasting (<-2 SD) rate	3.1	4.7
Iraq	IRQ	Contraceptive prevalence (CPR)	47.05	28.6
Jamaica	JAM	Safe abortion services	53.7	32.6
Jamaica	JAM	TT - Tetanus toxoid vaccination	90.0	43.3
Jamaica	JAM	Syphilis detection and treatment	24.1	11.6
Jamaica	JAM	Hypertensive disorder case management	20.6	9.9
Jamaica	JAM	Diabetes case management	16.0	7.7

Country	ISO 3166-1 alpha-3	Intervention Name	Baseline coverage (%)	Ending coverage (%)
Jamaica	JAM	Malaria case management	66.3	31.9
Jamaica	JAM	MgSO4 management of pre-eclampsia	40.7	19.6
Jamaica	JAM	Thermal protection	97.4	49.3
Jamaica	JAM	Clean cord care	94.0	47.6
Jamaica	JAM	Clean birth environment	80.8	40.9
Jamaica	JAM	Immediate drying and additional stimulation	90.2	45.7
Jamaica	JAM	Neonatal resuscitation	54.2	27.4
Jamaica	JAM	Antibiotics for preterm or prolonged PROM	73.8	37.4
Jamaica	JAM	Parenteral administration of anti-convulsants	70.5	35.7
Jamaica	JAM	Parenteral administration of uterotonics	88.1	44.6
Jamaica	JAM	Parenteral administration of antibiotics	73.8	37.4
Jamaica	JAM	Assisted vaginal delivery	24.9	12.6
Jamaica	JAM	Manual removal of placenta	36.9	18.7
Jamaica	JAM	Removal of retained products of conception	32.7	16.6
Jamaica	JAM	Cesarean delivery	8.6	4.4
Jamaica	JAM	Blood transfusion	12.4	6.3
Jamaica	JAM	Induction of labor for pregnancies lasting 41+ weeks	1.7	0.9
Jamaica	JAM	Improved sanitation - Utilization of latrines or toilets	87.3	87.3
Jamaica	JAM	Improved water source	90.6	90.6
Jamaica	JAM	Water connection in the home	76.0	76.0
Jamaica	JAM	Hand washing with soap	74.7	74.7
Jamaica	JAM	Hygienic disposal of children's stools	28.2	28.2
Jamaica	JAM	Injectable antibiotics for neonatal sepsis	98.6	47.4
Jamaica	JAM	ORS - oral rehydration solution	64.1	32.5
Jamaica	JAM	Oral antibiotics for pneumonia	82.3	41.7
Jamaica	JAM	BCG vaccine	93.0	44.7
Jamaica	JAM	Polio vaccine	98.0	47.1
Jamaica	JAM	DPT vaccine	97.0	46.7
Jamaica	JAM	H. influenzae type b vaccine	98.0	81.7
Jamaica	JAM	HepB vaccine	97.0	46.7
Jamaica	JAM	Measles vaccine	89.0	42.8
Jamaica	JAM	Global wasting (<-2 SD) rate	2.4	3.6
Jamaica	JAM	Contraceptive prevalence (CPR)	70.95	43.1
Jordan	JOR	Safe abortion services	40.0	24.3
Jordan	JOR	TT - Tetanus toxoid vaccination	90.0	43.3
Jordan	JOR	Syphilis detection and treatment	24.2	11.6
Jordan	JOR	Iron supplementation in pregnancy	48.9	23.5
Jordan	JOR	Hypertensive disorder case management	22.2	10.7
Jordan	JOR	Diabetes case management	17.3	8.3
Jordan	JOR	Malaria case management	71.6	34.4
Jordan	JOR	MgSO4 management of pre-eclampsia	43.9	21.1

Country	ISO 3166-1 alpha-3	Intervention Name	Baseline coverage (%)	Ending coverage (%)
Jordan	JOR	Thermal protection	96.9	49.1
Jordan	JOR	Clean cord care	93.6	47.4
Jordan	JOR	Clean birth environment	80.4	40.7
Jordan	JOR	Immediate drying and additional stimulation	89.8	45.5
Jordan	JOR	Neonatal resuscitation	53.9	27.3
Jordan	JOR	Antibiotics for preterm or prolonged PROM	73.4	37.2
Jordan	JOR	Parenteral administration of anti-convulsants	70.1	35.5
Jordan	JOR	Parenteral administration of uterotonics	87.6	44.3
Jordan	JOR	Parenteral administration of antibiotics	73.4	37.2
Jordan	JOR	Assisted vaginal delivery	24.8	12.6
Jordan	JOR	Manual removal of placenta	36.7	18.6
Jordan	JOR	Removal of retained products of conception	32.5	16.5
Jordan	JOR	Cesarean delivery	8.5	4.3
Jordan	JOR	Blood transfusion	12.3	6.2
Jordan	JOR	Induction of labor for pregnancies lasting 41+ weeks	1.7	0.9
Jordan	JOR	Complementary feeding - education only	51.4	29.7
Jordan	JOR	Complementary feeding - supplementary feeding and education	51.4	29.7
Jordan	JOR	Vitamin A supplementation	17.0	9.8
Jordan	JOR	Improved sanitation - Utilization of latrines or toilets	97.3	97.3
Jordan	JOR	Improved water source	98.9	98.9
Jordan	JOR	Water connection in the home	86.9	86.9
Jordan	JOR	Injectable antibiotics for neonatal sepsis	98.1	47.2
Jordan	JOR	ORS - oral rehydration solution	44.4	22.5
Jordan	JOR	Antibiotics for treatment of dysentery	54.5	27.6
Jordan	JOR	Oral antibiotics for pneumonia	60.9	30.8
Jordan	JOR	Vitamin A for treatment of measles	17.0	8.6
Jordan	JOR	BCG vaccine	94.0	45.2
Jordan	JOR	Polio vaccine	92.0	44.2
Jordan	JOR	DPT vaccine	96.0	46.2
Jordan	JOR	H. influenzae type b vaccine	96.0	80.1
Jordan	JOR	HepB vaccine	96.0	46.2
Jordan	JOR	Rotavirus vaccine	93.0	77.6
Jordan	JOR	Measles vaccine	92.0	44.2
Jordan	JOR	Global wasting (<-2 SD) rate	2.5	3.7
Jordan	JOR	Contraceptive prevalence (CPR)	53.2	32.3
Kazakhstan	KAZ	Safe abortion services	35.1	21.3
Kazakhstan	KAZ	Syphilis detection and treatment	24.5	11.8
Kazakhstan	KAZ	Hypertensive disorder case management	22.8	11.0
Kazakhstan	KAZ	Diabetes case management	17.8	8.6
Kazakhstan	KAZ	Malaria case management	73.7	35.4

Country	ISO 3166-1 alpha-3	Intervention Name	Baseline coverage (%)	Ending coverage (%)
Kazakhstan	KAZ	MgSO4 management of pre-eclampsia	45.2	21.7
Kazakhstan	KAZ	Thermal protection	98.2	49.7
Kazakhstan	KAZ	Clean cord care	94.8	48.0
Kazakhstan	KAZ	Clean birth environment	81.5	41.3
Kazakhstan	KAZ	Immediate drying and additional stimulation	91.0	46.1
Kazakhstan	KAZ	Neonatal resuscitation	54.6	27.6
Kazakhstan	KAZ	Antibiotics for preterm or prolonged PROM	74.3	37.6
Kazakhstan	KAZ	Parenteral administration of anti-convulsants	71.0	35.9
Kazakhstan	KAZ	Parenteral administration of uterotonics	88.8	45.0
Kazakhstan	KAZ	Parenteral administration of antibiotics	74.3	37.6
Kazakhstan	KAZ	Assisted vaginal delivery	25.1	12.7
Kazakhstan	KAZ	Manual removal of placenta	37.2	18.8
Kazakhstan	KAZ	Removal of retained products of conception	32.9	16.7
Kazakhstan	KAZ	Cesarean delivery	8.6	4.4
Kazakhstan	KAZ	Blood transfusion	12.5	6.3
Kazakhstan	KAZ	Induction of labor for pregnancies lasting 41+ weeks	1.7	0.9
Kazakhstan	KAZ	Complementary feeding - education only	64.2	37.1
Kazakhstan	KAZ	Complementary feeding - supplementary feeding and education	64.2	37.1
Kazakhstan	KAZ	Improved sanitation - Utilization of latrines or toilets	97.9	97.9
Kazakhstan	KAZ	Improved water source	95.6	95.6
Kazakhstan	KAZ	Water connection in the home	77.3	77.3
Kazakhstan	KAZ	Hand washing with soap	99.0	99.0
Kazakhstan	KAZ	Hygienic disposal of children's stools	66.7	66.7
Kazakhstan	KAZ	Injectable antibiotics for neonatal sepsis	99.3	47.8
Kazakhstan	KAZ	ORS - oral rehydration solution	61.8	31.3
Kazakhstan	KAZ	Antibiotics for treatment of dysentery	17.2	8.7
Kazakhstan	KAZ	Oral antibiotics for pneumonia	81.2	41.1
Kazakhstan	KAZ	BCG vaccine	95.0	45.7
Kazakhstan	KAZ	Polio vaccine	98.0	47.1
Kazakhstan	KAZ	DPT vaccine	98.0	47.1
Kazakhstan	KAZ	H. influenzae type b vaccine	98.0	81.7
Kazakhstan	KAZ	HepB vaccine	98.0	47.1
Kazakhstan	KAZ	Pneumococcal vaccine	95.0	79.2
Kazakhstan	KAZ	Measles vaccine	99.0	47.6
Kazakhstan	KAZ	Global wasting (<-2 SD) rate	3.3	4.9
Kazakhstan	KAZ	Contraceptive prevalence (CPR)	56.2	34.1
Kenya	KEN	Safe abortion services	3.3	2.0
Kenya	KEN	TT - Tetanus toxoid vaccination	88.0	42.3
Kenya	KEN	IPTp - Intermittent preventive treatment of malaria during pregnancy	34.7	16.7
Kenya	KEN	Syphilis detection and treatment	52.9	25.4

Country	ISO 3166-1 alpha-3	Intervention Name	Baseline coverage (%)	Ending coverage (%)
Kenya	KEN	Iron supplementation in pregnancy	7.5	3.6
Kenya	KEN	Hypertensive disorder case management	14.0	6.7
Kenya	KEN	Diabetes case management	12.5	6.0
Kenya	KEN	Malaria case management	32.7	15.7
Kenya	KEN	MgSO4 management of pre-eclampsia	24.6	11.8
Kenya	KEN	Thermal protection	60.7	30.7
Kenya	KEN	Clean cord care	58.7	29.7
Kenya	KEN	Clean birth environment	50.4	25.5
Kenya	KEN	Immediate drying and additional stimulation	53.6	27.1
Kenya	KEN	Neonatal resuscitation	50.9	25.8
Kenya	KEN	Antibiotics for preterm or prolonged PROM	47.0	23.8
Kenya	KEN	Parenteral administration of anti-convulsants	60.0	30.4
Kenya	KEN	Parenteral administration of uterotonics	46.6	23.6
Kenya	KEN	Parenteral administration of antibiotics	47.0	23.8
Kenya	KEN	Assisted vaginal delivery	5.2	2.6
Kenya	KEN	Manual removal of placenta	42.8	21.7
Kenya	KEN	Removal of retained products of conception	31.3	15.8
Kenya	KEN	Cesarean delivery	11.3	5.7
Kenya	KEN	Blood transfusion	7.7	3.9
Kenya	KEN	Induction of labor for pregnancies lasting 41+ weeks	1.1	0.6
Kenya	KEN	Complementary feeding - education only	40.9	23.6
Kenya	KEN	Complementary feeding - supplementary feeding and education	40.9	23.6
Kenya	KEN	Vitamin A supplementation	44.0	25.4
Kenya	KEN	Improved sanitation - Utilization of latrines or toilets	29.1	29.1
Kenya	KEN	Improved water source	58.9	58.9
Kenya	KEN	Water connection in the home	18.9	18.9
Kenya	KEN	Hand washing with soap	25.2	25.2
Kenya	KEN	Hygienic disposal of children's stools	70.2	70.2
Kenya	KEN	ITN/IRS - Households protected from malaria	62.5	36.1
Kenya	KEN	Injectable antibiotics for neonatal sepsis	61.5	29.6
Kenya	KEN	ORS - oral rehydration solution	53.8	27.2
Kenya	KEN	Antibiotics for treatment of dysentery	15.7	7.9
Kenya	KEN	Zinc for treatment of diarrhea	8.1	4.1
Kenya	KEN	Oral antibiotics for pneumonia	65.7	33.3
Kenya	KEN	Vitamin A for treatment of measles	44.0	22.3
Kenya	KEN	ACTs- Artemisinin compounds for treatment of malaria	15.0	7.6
Kenya	KEN	SAM - treatment for severe acute malnutrition	7.2	3.6
Kenya	KEN	BCG vaccine	95.0	45.7
Kenya	KEN	Polio vaccine	81.0	39.0
Kenya	KEN	DPT vaccine	92.0	44.2

Country	ISO 3166-1 alpha-3	Intervention Name	Baseline coverage (%)	Ending coverage (%)
Kenya	KEN	H. influenzae type b vaccine	92.0	76.7
Kenya	KEN	HepB vaccine	92.0	44.2
Kenya	KEN	Pneumococcal vaccine	81.0	67.5
Kenya	KEN	Rotavirus vaccine	78.0	65.0
Kenya	KEN	Measles vaccine	89.0	42.8
Kenya	KEN	Global wasting (<-2 SD) rate	4.2	6.3
Kenya	KEN	Contraceptive prevalence (CPR)	61.55	37.4
Kyrgyzstan	KGZ	Safe abortion services	35.1	21.3
Kyrgyzstan	KGZ	Syphilis detection and treatment	24.7	11.9
Kyrgyzstan	KGZ	Iron supplementation in pregnancy	2.3	1.1
Kyrgyzstan	KGZ	Hypertensive disorder case management	24.0	11.5
Kyrgyzstan	KGZ	Diabetes case management	18.7	9.0
Kyrgyzstan	KGZ	Malaria case management	77.4	37.2
Kyrgyzstan	KGZ	MgSO4 management of pre-eclampsia	47.4	22.8
Kyrgyzstan	KGZ	Thermal protection	98.4	49.8
Kyrgyzstan	KGZ	Clean cord care	95.0	48.1
Kyrgyzstan	KGZ	Clean birth environment	81.7	41.4
Kyrgyzstan	KGZ	Immediate drying and additional stimulation	91.2	46.2
Kyrgyzstan	KGZ	Neonatal resuscitation	54.8	27.7
Kyrgyzstan	KGZ	Antibiotics for preterm or prolonged PROM	74.5	37.7
Kyrgyzstan	KGZ	Parenteral administration of anti-convulsants	71.2	36.0
Kyrgyzstan	KGZ	Parenteral administration of uterotonics	89.0	45.1
Kyrgyzstan	KGZ	Parenteral administration of antibiotics	74.5	37.7
Kyrgyzstan	KGZ	Assisted vaginal delivery	25.2	12.8
Kyrgyzstan	KGZ	Manual removal of placenta	37.3	18.9
Kyrgyzstan	KGZ	Removal of retained products of conception	33.0	16.7
Kyrgyzstan	KGZ	Cesarean delivery	8.7	4.4
Kyrgyzstan	KGZ	Blood transfusion	12.5	6.3
Kyrgyzstan	KGZ	Induction of labor for pregnancies lasting 41+ weeks	1.8	0.9
Kyrgyzstan	KGZ	Complementary feeding - education only	61.7	35.6
Kyrgyzstan	KGZ	Complementary feeding - supplementary feeding and education	61.7	35.6
Kyrgyzstan	KGZ	Vitamin A supplementation	97.0	56.0
Kyrgyzstan	KGZ	Improved sanitation - Utilization of latrines or toilets	96.5	96.5
Kyrgyzstan	KGZ	Improved water source	87.5	87.5
Kyrgyzstan	KGZ	Water connection in the home	78.1	78.1
Kyrgyzstan	KGZ	Hand washing with soap	98.1	98.1
Kyrgyzstan	KGZ	Hygienic disposal of children's stools	75.8	75.8
Kyrgyzstan	KGZ	Injectable antibiotics for neonatal sepsis	99.6	47.9
Kyrgyzstan	KGZ	ORS - oral rehydration solution	36.4	18.4
Kyrgyzstan	KGZ	Zinc for treatment of diarrhea	21.8	11.0

Country	ISO 3166-1 alpha-3	Intervention Name	Baseline coverage (%)	Ending coverage (%)
Kyrgyzstan	KGZ	Oral antibiotics for pneumonia	59.7	30.2
Kyrgyzstan	KGZ	Vitamin A for treatment of measles	97.0	49.1
Kyrgyzstan	KGZ	BCG vaccine	97.0	46.7
Kyrgyzstan	KGZ	Polio vaccine	92.0	44.2
Kyrgyzstan	KGZ	DPT vaccine	94.0	45.2
Kyrgyzstan	KGZ	H. influenzae type b vaccine	92.0	76.7
Kyrgyzstan	KGZ	HepB vaccine	92.0	44.2
Kyrgyzstan	KGZ	Pneumococcal vaccine	92.0	76.7
Kyrgyzstan	KGZ	Measles vaccine	96.0	46.2
Kyrgyzstan	KGZ	Global wasting (<-2 SD) rate	2.8	4.3
Kyrgyzstan	KGZ	Contraceptive prevalence (CPR)	41.6	25.3
Lao People's Democratic Republic	LAO	Safe abortion services	39.0	23.7
Lao People's Democratic Republic	LAO	TT - Tetanus toxoid vaccination	90.0	43.3
Lao People's Democratic Republic	LAO	IPTp - Intermittent preventive treatment of malaria during pregnancy	2.5	1.2
Lao People's Democratic Republic	LAO	Syphilis detection and treatment	19.4	9.3
Lao People's Democratic Republic	LAO	Iron supplementation in pregnancy	55.1	26.5
Lao People's Democratic Republic	LAO	Hypertensive disorder case management	14.9	7.2
Lao People's Democratic Republic	LAO	Diabetes case management	11.7	5.6
Lao People's Democratic Republic	LAO	Malaria case management	48.2	23.2
Lao People's Democratic Republic	LAO	MgSO4 management of pre-eclampsia	29.6	14.2
Lao People's Democratic Republic	LAO	Thermal protection	63.7	32.2
Lao People's Democratic Republic	LAO	Clean cord care	61.5	31.1
Lao People's Democratic Republic	LAO	Clean birth environment	52.8	26.7
Lao People's Democratic Republic	LAO	Immediate drying and additional stimulation	59.0	29.9
Lao People's Democratic Republic	LAO	Neonatal resuscitation	35.4	17.9
Lao People's Democratic Republic	LAO	Antibiotics for preterm or prolonged PROM	48.2	24.4
Lao People's Democratic Republic	LAO	Parenteral administration of anti-convulsants	46.0	23.3
Lao People's Democratic Republic	LAO	Parenteral administration of uterotonics	57.5	29.1
Lao People's Democratic Republic	LAO	Parenteral administration of antibiotics	48.2	24.4
Lao People's Democratic Republic	LAO	Assisted vaginal delivery	16.3	8.3

Country	ISO 3166-1 alpha-3	Intervention Name	Baseline coverage (%)	Ending coverage (%)
Lao People's Democratic Republic	LAO	Manual removal of placenta	24.1	12.2
Lao People's Democratic Republic	LAO	Removal of retained products of conception	21.3	10.8
Lao People's Democratic Republic	LAO	Cesarean delivery	5.6	2.8
Lao People's Democratic Republic	LAO	Blood transfusion	8.1	4.1
Lao People's Democratic Republic	LAO	Induction of labor for pregnancies lasting 41+ weeks	1.1	0.6
Lao People's Democratic Republic	LAO	Complementary feeding - education only	33.5	19.3
Lao People's Democratic Republic	LAO	Complementary feeding - supplementary feeding and education	33.5	19.3
Lao People's Democratic Republic	LAO	Vitamin A supplementation	57.0	32.9
Lao People's Democratic Republic	LAO	Improved sanitation - Utilization of latrines or toilets	74.5	74.5
Lao People's Democratic Republic	LAO	Improved water source	82.1	82.1
Lao People's Democratic Republic	LAO	Water connection in the home	40.6	40.6
Lao People's Democratic Republic	LAO	Hand washing with soap	91.8	91.8
Lao People's Democratic Republic	LAO	Hygienic disposal of children's stools	27.9	27.9
Lao People's Democratic Republic	LAO	ITN/IRS - Households protected from malaria	62.5	36.1
Lao People's Democratic Republic	LAO	Injectable antibiotics for neonatal sepsis	64.4	31.0
Lao People's Democratic Republic	LAO	ORS - oral rehydration solution	56.1	28.4
Lao People's Democratic Republic	LAO	Zinc for treatment of diarrhea	15.2	7.7
Lao People's Democratic Republic	LAO	Oral antibiotics for pneumonia	38.5	19.5
Lao People's Democratic Republic	LAO	Vitamin A for treatment of measles	57.0	28.9
Lao People's Democratic Republic	LAO	ACTs- Artemisinin compounds for treatment of malaria	3.5	1.8
Lao People's Democratic Republic	LAO	SAM - treatment for severe acute malnutrition	2.4	1.2
Lao People's Democratic Republic	LAO	BCG vaccine	79.0	38.0
Lao People's Democratic Republic	LAO	Polio vaccine	67.0	32.2
Lao People's Democratic Republic	LAO	DPT vaccine	68.0	32.7
Lao People's Democratic Republic	LAO	H. influenzae type b vaccine	68.0	56.7
Lao People's Democratic Republic	LAO	HepB vaccine	68.0	32.7

Country	ISO 3166-1 alpha-3	Intervention Name	Baseline coverage (%)	Ending coverage (%)
Lao People's Democratic Republic	LAO	Pneumococcal vaccine	56.0	46.7
Lao People's Democratic Republic	LAO	Measles vaccine	82.0	39.4
Lao People's Democratic Republic	LAO	Global wasting (<-2 SD) rate	9.1	13.6
Lao People's Democratic Republic	LAO	Contraceptive prevalence (CPR)	57.2	34.7
Lesotho	LSO	Safe abortion services	42.5	25.8
Lesotho	LSO	TT - Tetanus toxoid vaccination	85.0	40.9
Lesotho	LSO	Syphilis detection and treatment	23.5	11.3
Lesotho	LSO	Iron supplementation in pregnancy	51.4	24.7
Lesotho	LSO	Hypertensive disorder case management	18.4	8.8
Lesotho	LSO	Diabetes case management	14.4	6.9
Lesotho	LSO	Malaria case management	59.4	28.6
Lesotho	LSO	MgSO4 management of pre-eclampsia	36.4	17.5
Lesotho	LSO	Thermal protection	88.4	44.8
Lesotho	LSO	Clean cord care	85.3	43.2
Lesotho	LSO	Clean birth environment	73.3	37.1
Lesotho	LSO	Immediate drying and additional stimulation	81.9	41.5
Lesotho	LSO	Neonatal resuscitation	49.2	24.9
Lesotho	LSO	Antibiotics for preterm or prolonged PROM	66.9	33.9
Lesotho	LSO	Parenteral administration of anti-convulsants	63.9	32.3
Lesotho	LSO	Parenteral administration of uterotonics	79.9	40.4
Lesotho	LSO	Parenteral administration of antibiotics	66.9	33.9
Lesotho	LSO	Assisted vaginal delivery	22.6	11.4
Lesotho	LSO	Manual removal of placenta	33.4	16.9
Lesotho	LSO	Removal of retained products of conception	29.6	15.0
Lesotho	LSO	Cesarean delivery	7.8	3.9
Lesotho	LSO	Blood transfusion	11.3	5.7
Lesotho	LSO	Induction of labor for pregnancies lasting 41+ weeks	1.6	0.8
Lesotho	LSO	Complementary feeding - education only	23.1	13.3
Lesotho	LSO	Complementary feeding - supplementary feeding and education	23.1	13.3
Lesotho	LSO	Vitamin A supplementation	18.0	10.4
Lesotho	LSO	Improved sanitation - Utilization of latrines or toilets	42.8	42.8
Lesotho	LSO	Improved water source	68.6	68.6
Lesotho	LSO	Water connection in the home	43.5	43.5
Lesotho	LSO	Hand washing with soap	36.3	36.3
Lesotho	LSO	Hygienic disposal of children's stools	54.6	54.6
Lesotho	LSO	Injectable antibiotics for neonatal sepsis	89.4	43.0
Lesotho	LSO	ORS - oral rehydration solution	39.7	20.1
Lesotho	LSO	Antibiotics for treatment of dysentery	26.1	13.2

Country	ISO 3166-1 alpha-3	Intervention Name	Baseline coverage (%)	Ending coverage (%)
Lesotho	LSO	Zinc for treatment of diarrhea	17.4	8.8
Lesotho	LSO	Oral antibiotics for pneumonia	57.5	29.1
Lesotho	LSO	Vitamin A for treatment of measles	18.0	9.1
Lesotho	LSO	SAM - treatment for severe acute malnutrition	6.2	3.1
Lesotho	LSO	BCG vaccine	98.0	47.1
Lesotho	LSO	Polio vaccine	90.0	43.3
Lesotho	LSO	DPT vaccine	93.0	44.7
Lesotho	LSO	H. influenzae type b vaccine	93.0	77.6
Lesotho	LSO	HepB vaccine	93.0	44.7
Lesotho	LSO	Pneumococcal vaccine	93.0	77.6
Lesotho	LSO	Rotavirus vaccine	70.0	58.4
Lesotho	LSO	Measles vaccine	90.0	43.3
Lesotho	LSO	Global wasting (<-2 SD) rate	3.0	4.5
Lesotho	LSO	Contraceptive prevalence (CPR)	65.45	39.8
Liberia	LBR	TT - Tetanus toxoid vaccination	89.0	42.8
Liberia	LBR	IPTp - Intermittent preventive treatment of malaria during pregnancy	54.5	26.2
Liberia	LBR	Syphilis detection and treatment	23.7	11.4
Liberia	LBR	Iron supplementation in pregnancy	21.2	10.2
Liberia	LBR	Hypertensive disorder case management	18.6	8.9
Liberia	LBR	Diabetes case management	14.5	7.0
Liberia	LBR	Malaria case management	60.1	28.9
Liberia	LBR	MgSO4 management of pre-eclampsia	36.9	17.7
Liberia	LBR	Thermal protection	55.2	27.9
Liberia	LBR	Clean cord care	53.3	27.0
Liberia	LBR	Clean birth environment	45.8	23.2
Liberia	LBR	Immediate drying and additional stimulation	51.1	25.9
Liberia	LBR	Neonatal resuscitation	30.7	15.5
Liberia	LBR	Antibiotics for preterm or prolonged PROM	41.8	21.2
Liberia	LBR	Parenteral administration of anti-convulsants	39.9	20.2
Liberia	LBR	Parenteral administration of uterotonics	49.9	25.3
Liberia	LBR	Parenteral administration of antibiotics	41.8	21.2
Liberia	LBR	Assisted vaginal delivery	14.1	7.1
Liberia	LBR	Manual removal of placenta	20.9	10.6
Liberia	LBR	Removal of retained products of conception	18.5	9.4
Liberia	LBR	Cesarean delivery	4.9	2.5
Liberia	LBR	Blood transfusion	7.0	3.5
Liberia	LBR	Induction of labor for pregnancies lasting 41+ weeks	1.0	0.5
Liberia	LBR	Complementary feeding - education only	13.5	7.8
Liberia	LBR	Complementary feeding - supplementary feeding and education	13.5	7.8
Liberia	LBR	Vitamin A supplementation	97.0	56.0

Country	ISO 3166-1 alpha-3	Intervention Name	Baseline coverage (%)	Ending coverage (%)
Liberia	LBR	Improved sanitation - Utilization of latrines or toilets	17.0	17.0
Liberia	LBR	Improved water source	72.9	72.9
Liberia	LBR	Water connection in the home	3.4	3.4
Liberia	LBR	Hand washing with soap	3.8	3.8
Liberia	LBR	Hygienic disposal of children's stools	23.2	23.2
Liberia	LBR	ITN/IRS - Households protected from malaria	62.1	35.8
Liberia	LBR	Injectable antibiotics for neonatal sepsis	55.8	26.8
Liberia	LBR	ORS - oral rehydration solution	60.4	30.6
Liberia	LBR	Antibiotics for treatment of dysentery	18.7	9.5
Liberia	LBR	Zinc for treatment of diarrhea	3.1	1.6
Liberia	LBR	Oral antibiotics for pneumonia	50.7	25.7
Liberia	LBR	Vitamin A for treatment of measles	97.0	49.1
Liberia	LBR	ACTs- Artemisinin compounds for treatment of malaria	16.7	8.5
Liberia	LBR	SAM - treatment for severe acute malnutrition	15.1	7.6
Liberia	LBR	BCG vaccine	92.0	44.2
Liberia	LBR	Polio vaccine	84.0	40.4
Liberia	LBR	DPT vaccine	84.0	40.4
Liberia	LBR	H. influenzae type b vaccine	84.0	70.0
Liberia	LBR	HepB vaccine	84.0	40.4
Liberia	LBR	Pneumococcal vaccine	84.0	70.0
Liberia	LBR	Rotavirus vaccine	74.0	61.7
Liberia	LBR	Measles vaccine	87.0	41.8
Liberia	LBR	Global wasting (<-2 SD) rate	5.4	8.1
Liberia	LBR	Contraceptive prevalence (CPR)	30.45	18.5
Madagascar	MDG	Safe abortion services	3.3	2.0
Madagascar	MDG	TT - Tetanus toxoid vaccination	78.0	37.5
Madagascar	MDG	IPTp - Intermittent preventive treatment of malaria during pregnancy	22.3	10.7
Madagascar	MDG	Syphilis detection and treatment	12.6	6.1
Madagascar	MDG	Iron supplementation in pregnancy	7.6	3.7
Madagascar	MDG	Hypertensive disorder case management	11.3	5.4
Madagascar	MDG	Diabetes case management	8.8	4.2
Madagascar	MDG	Malaria case management	36.6	17.6
Madagascar	MDG	MgSO4 management of pre-eclampsia	22.4	10.8
Madagascar	MDG	Thermal protection	37.5	19.0
Madagascar	MDG	Clean cord care	36.2	18.3
Madagascar	MDG	Clean birth environment	31.1	15.7
Madagascar	MDG	Immediate drying and additional stimulation	34.7	17.6
Madagascar	MDG	Neonatal resuscitation	20.8	10.5
Madagascar	MDG	Antibiotics for preterm or prolonged PROM	28.4	14.4
Madagascar	MDG	Parenteral administration of anti-convulsants	27.1	13.7

Country	ISO 3166-1 alpha-3	Intervention Name	Baseline coverage (%)	Ending coverage (%)
Madagascar	MDG	Parenteral administration of uterotonics	33.9	17.2
Madagascar	MDG	Parenteral administration of antibiotics	28.4	14.4
Madagascar	MDG	Assisted vaginal delivery	9.6	4.9
Madagascar	MDG	Manual removal of placenta	14.2	7.2
Madagascar	MDG	Removal of retained products of conception	12.6	6.4
Madagascar	MDG	Cesarean delivery	3.3	1.7
Madagascar	MDG	Blood transfusion	4.8	2.4
Madagascar	MDG	Induction of labor for pregnancies lasting 41+ weeks	0.7	0.4
Madagascar	MDG	Complementary feeding - education only	24.1	13.9
Madagascar	MDG	Complementary feeding - supplementary feeding and education	24.1	13.9
Madagascar	MDG	Vitamin A supplementation	87.0	50.2
Madagascar	MDG	Improved sanitation - Utilization of latrines or toilets	10.5	10.5
Madagascar	MDG	Improved water source	54.4	54.4
Madagascar	MDG	Water connection in the home	19.3	19.3
Madagascar	MDG	Hand washing with soap	4.0	4.0
Madagascar	MDG	Hygienic disposal of children's stools	39.0	39.0
Madagascar	MDG	ITN/IRS - Households protected from malaria	79.5	45.9
Madagascar	MDG	Injectable antibiotics for neonatal sepsis	37.9	18.2
Madagascar	MDG	ORS - oral rehydration solution	44.5	22.5
Madagascar	MDG	Antibiotics for treatment of dysentery	34.1	17.3
Madagascar	MDG	Zinc for treatment of diarrhea	3.0	1.5
Madagascar	MDG	Oral antibiotics for pneumonia	42.0	21.3
Madagascar	MDG	Vitamin A for treatment of measles	87.0	44.0
Madagascar	MDG	ACTs- Artemisinin compounds for treatment of malaria	3.4	1.7
Madagascar	MDG	SAM - treatment for severe acute malnutrition	1.4	0.7
Madagascar	MDG	BCG vaccine	70.0	33.7
Madagascar	MDG	Polio vaccine	76.0	36.6
Madagascar	MDG	DPT vaccine	75.0	36.1
Madagascar	MDG	H. influenzae type b vaccine	75.0	62.5
Madagascar	MDG	HepB vaccine	75.0	36.1
Madagascar	MDG	Pneumococcal vaccine	75.0	62.5
Madagascar	MDG	Rotavirus vaccine	78.0	65.0
Madagascar	MDG	Measles vaccine	58.0	27.9
Madagascar	MDG	Global wasting (<-2 SD) rate	14.5	21.7
Madagascar	MDG	Contraceptive prevalence (CPR)	48.2	29.3
Malawi	MWI	Safe abortion services	3.3	2.0
Malawi	MWI	TT - Tetanus toxoid vaccination	89.0	42.8
Malawi	MWI	IPTp - Intermittent preventive treatment of malaria during pregnancy	76.1	36.6
Malawi	MWI	Syphilis detection and treatment	15.2	7.3
Malawi	MWI	Iron supplementation in pregnancy	33.4	16.1

Country	ISO 3166-1 alpha-3	Intervention Name	Baseline coverage (%)	Ending coverage (%)
Malawi	MWI	Hypertensive disorder case management	5.0	2.4
Malawi	MWI	Diabetes case management	6.4	3.1
Malawi	MWI	Malaria case management	41.0	19.7
Malawi	MWI	MgSO4 management of pre-eclampsia	40.6	19.5
Malawi	MWI	Thermal protection	90.5	45.8
Malawi	MWI	Clean cord care	84.1	42.6
Malawi	MWI	Clean birth environment	64.6	32.7
Malawi	MWI	Immediate drying and additional stimulation	83.0	42.0
Malawi	MWI	Neonatal resuscitation	83.7	42.4
Malawi	MWI	Antibiotics for preterm or prolonged PROM	80.3	40.7
Malawi	MWI	Parenteral administration of anti-convulsants	88.0	44.6
Malawi	MWI	Parenteral administration of uterotonics	89.5	45.3
Malawi	MWI	Parenteral administration of antibiotics	80.3	40.7
Malawi	MWI	Assisted vaginal delivery	46.1	23.3
Malawi	MWI	Manual removal of placenta	4.5	2.3
Malawi	MWI	Removal of retained products of conception	30.0	15.2
Malawi	MWI	Cesarean delivery	1.9	1.0
Malawi	MWI	Blood transfusion	12.2	6.2
Malawi	MWI	Induction of labor for pregnancies lasting 41+ weeks	6.1	3.1
Malawi	MWI	Complementary feeding - education only	25.0	14.4
Malawi	MWI	Complementary feeding - supplementary feeding and education	25.0	14.4
Malawi	MWI	Vitamin A supplementation	91.0	52.5
Malawi	MWI	Improved sanitation - Utilization of latrines or toilets	26.2	26.2
Malawi	MWI	Improved water source	68.8	68.8
Malawi	MWI	Water connection in the home	14.9	14.9
Malawi	MWI	Hand washing with soap	10.7	10.7
Malawi	MWI	Hygienic disposal of children's stools	85.5	85.5
Malawi	MWI	ITN/IRS - Households protected from malaria	82.1	47.4
Malawi	MWI	Injectable antibiotics for neonatal sepsis	90.7	43.6
Malawi	MWI	ORS - oral rehydration solution	64.7	32.8
Malawi	MWI	Antibiotics for treatment of dysentery	19.5	9.9
Malawi	MWI	Zinc for treatment of diarrhea	28.1	14.2
Malawi	MWI	Oral antibiotics for pneumonia	74.4	37.7
Malawi	MWI	Vitamin A for treatment of measles	91.0	46.1
Malawi	MWI	ACTs- Artemisinin compounds for treatment of malaria	27.8	14.1
Malawi	MWI	SAM - treatment for severe acute malnutrition	7.2	3.6
Malawi	MWI	BCG vaccine	92.0	44.2
Malawi	MWI	Polio vaccine	91.0	43.8
Malawi	MWI	DPT vaccine	92.0	44.2
Malawi	MWI	H. influenzae type b vaccine	92.0	76.7

Country	ISO 3166-1 alpha-3	Intervention Name	Baseline coverage (%)	Ending coverage (%)
Malawi	MWI	HepB vaccine	92.0	44.2
Malawi	MWI	Pneumococcal vaccine	92.0	76.7
Malawi	MWI	Rotavirus vaccine	90.0	75.1
Malawi	MWI	Measles vaccine	83.0	39.9
Malawi	MWI	Global wasting (<-2 SD) rate	2.8	4.3
Malawi	MWI	Contraceptive prevalence (CPR)	63	38.3
Maldives	MDV	Safe abortion services	35.1	21.3
Maldives	MDV	TT - Tetanus toxoid vaccination	99.0	47.6
Maldives	MDV	Syphilis detection and treatment	24.4	11.7
Maldives	MDV	Iron supplementation in pregnancy	64.6	31.1
Maldives	MDV	Hypertensive disorder case management	19.7	9.5
Maldives	MDV	Diabetes case management	15.3	7.4
Maldives	MDV	Malaria case management	63.5	30.5
Maldives	MDV	MgSO4 management of pre-eclampsia	38.9	18.7
Maldives	MDV	Thermal protection	98.3	49.8
Maldives	MDV	Clean cord care	94.9	48.0
Maldives	MDV	Clean birth environment	81.6	41.3
Maldives	MDV	Immediate drying and additional stimulation	91.1	46.1
Maldives	MDV	Neonatal resuscitation	54.7	27.7
Maldives	MDV	Antibiotics for preterm or prolonged PROM	74.4	37.7
Maldives	MDV	Parenteral administration of anti-convulsants	71.1	36.0
Maldives	MDV	Parenteral administration of uterotonics	88.9	45.0
Maldives	MDV	Parenteral administration of antibiotics	74.4	37.7
Maldives	MDV	Assisted vaginal delivery	25.1	12.7
Maldives	MDV	Manual removal of placenta	37.2	18.8
Maldives	MDV	Removal of retained products of conception	33.0	16.7
Maldives	MDV	Cesarean delivery	8.7	4.4
Maldives	MDV	Blood transfusion	12.5	6.3
Maldives	MDV	Induction of labor for pregnancies lasting 41+ weeks	1.8	0.9
Maldives	MDV	Complementary feeding - education only	75.8	43.7
Maldives	MDV	Complementary feeding - supplementary feeding and education	75.8	43.7
Maldives	MDV	Vitamin A supplementation	69.0	39.8
Maldives	MDV	Improved sanitation - Utilization of latrines or toilets	99.0	99.0
Maldives	MDV	Improved water source	99.0	99.0
Maldives	MDV	Water connection in the home	47.3	47.3
Maldives	MDV	Hand washing with soap	95.8	95.8
Maldives	MDV	Hygienic disposal of children's stools	9.1	9.1
Maldives	MDV	Injectable antibiotics for neonatal sepsis	99.5	47.9
Maldives	MDV	ORS - oral rehydration solution	74.8	37.9
Maldives	MDV	Zinc for treatment of diarrhea	48.3	24.5

**Country**	**ISO 3166-1 alpha-3**	**Intervention Name**	**Baseline coverage (%)**	**Ending coverage (%)**
Maldives	MDV	Oral antibiotics for pneumonia	74.1	37.5
Maldives	MDV	Vitamin A for treatment of measles	69.0	34.9
Maldives	MDV	BCG vaccine	99.0	47.6
Maldives	MDV	Polio vaccine	99.0	47.6
Maldives	MDV	DPT vaccine	99.0	47.6
Maldives	MDV	H. influenzae type b vaccine	99.0	82.6
Maldives	MDV	HepB vaccine	99.0	47.6
Maldives	MDV	Measles vaccine	99.0	47.6
Maldives	MDV	Global wasting (<-2 SD) rate	9.1	13.7
Maldives	MDV	Contraceptive prevalence (CPR)	34.55	21.0
Mali	MLI	TT - Tetanus toxoid vaccination	85.0	40.9
Mali	MLI	IPTp - Intermittent preventive treatment of malaria during pregnancy	55.4	26.6
Mali	MLI	Syphilis detection and treatment	19.4	9.3
Mali	MLI	Iron supplementation in pregnancy	18.3	8.8
Mali	MLI	Hypertensive disorder case management	10.4	5.0
Mali	MLI	Diabetes case management	8.1	3.9
Mali	MLI	Malaria case management	33.6	16.2
Mali	MLI	MgSO4 management of pre-eclampsia	20.6	9.9
Mali	MLI	Thermal protection	66.0	33.4
Mali	MLI	Clean cord care	63.7	32.2
Mali	MLI	Clean birth environment	54.8	27.7
Mali	MLI	Immediate drying and additional stimulation	61.2	31.0
Mali	MLI	Neonatal resuscitation	36.7	18.6
Mali	MLI	Antibiotics for preterm or prolonged PROM	50.0	25.3
Mali	MLI	Parenteral administration of anti-convulsants	47.8	24.2
Mali	MLI	Parenteral administration of uterotonics	59.7	30.2
Mali	MLI	Parenteral administration of antibiotics	50.0	25.3
Mali	MLI	Assisted vaginal delivery	16.9	8.6
Mali	MLI	Manual removal of placenta	25.0	12.7
Mali	MLI	Removal of retained products of conception	22.1	11.2
Mali	MLI	Cesarean delivery	5.8	2.9
Mali	MLI	Blood transfusion	8.4	4.3
Mali	MLI	Induction of labor for pregnancies lasting 41+ weeks	1.2	0.6
Mali	MLI	Complementary feeding - education only	21.8	12.6
Mali	MLI	Complementary feeding - supplementary feeding and education	21.8	12.6
Mali	MLI	Vitamin A supplementation	9.0	5.2
Mali	MLI	Improved sanitation - Utilization of latrines or toilets	39.3	39.3
Mali	MLI	Improved water source	78.3	78.3
Mali	MLI	Water connection in the home	35.6	35.6
Mali	MLI	Hand washing with soap	60.9	60.9

Country	ISO 3166-1 alpha-3	Intervention Name	Baseline coverage (%)	Ending coverage (%)
Mali	MLI	Hygienic disposal of children's stools	65.0	65.0
Mali	MLI	ITN/IRS - Households protected from malaria	89.8	51.8
Mali	MLI	Injectable antibiotics for neonatal sepsis	66.8	32.1
Mali	MLI	ORS - oral rehydration solution	21.4	10.8
Mali	MLI	Antibiotics for treatment of dysentery	18.5	9.4
Mali	MLI	Zinc for treatment of diarrhea	15.4	7.8
Mali	MLI	Oral antibiotics for pneumonia	70.9	35.9
Mali	MLI	Vitamin A for treatment of measles	9.0	4.6
Mali	MLI	ACTs- Artemisinin compounds for treatment of malaria	0.9	0.5
Mali	MLI	SAM - treatment for severe acute malnutrition	41.8	21.2
Mali	MLI	BCG vaccine	83.0	39.9
Mali	MLI	Polio vaccine	73.0	35.1
Mali	MLI	DPT vaccine	71.0	34.1
Mali	MLI	H. influenzae type b vaccine	71.0	59.2
Mali	MLI	HepB vaccine	71.0	34.1
Mali	MLI	Pneumococcal vaccine	68.0	56.7
Mali	MLI	Rotavirus vaccine	55.0	45.9
Mali	MLI	Meningococcal A	70.0	33.7
Mali	MLI	Measles vaccine	61.0	29.3
Mali	MLI	Global wasting (<-2 SD) rate	13.4	20.1
Mali	MLI	Contraceptive prevalence (CPR)	17.05	10.4
Mauritania	MRT	TT - Tetanus toxoid vaccination	80.0	38.5
Mauritania	MRT	IPTp - Intermittent preventive treatment of malaria during pregnancy	25.2	12.1
Mauritania	MRT	Syphilis detection and treatment	21.5	10.3
Mauritania	MRT	Iron supplementation in pregnancy	6.1	2.9
Mauritania	MRT	Hypertensive disorder case management	15.1	7.3
Mauritania	MRT	Diabetes case management	11.8	5.7
Mauritania	MRT	Malaria case management	48.8	23.5
Mauritania	MRT	MgSO4 management of pre-eclampsia	29.9	14.4
Mauritania	MRT	Thermal protection	68.5	34.7
Mauritania	MRT	Clean cord care	55.2	27.9
Mauritania	MRT	Clean birth environment	44.0	22.3
Mauritania	MRT	Immediate drying and additional stimulation	63.7	32.2
Mauritania	MRT	Neonatal resuscitation	32.4	16.4
Mauritania	MRT	Antibiotics for preterm or prolonged PROM	47.5	24.0
Mauritania	MRT	Parenteral administration of anti-convulsants	25.5	12.9
Mauritania	MRT	Parenteral administration of uterotonics	54.8	27.7
Mauritania	MRT	Parenteral administration of antibiotics	47.5	24.0
Mauritania	MRT	Assisted vaginal delivery	23.3	11.8
Mauritania	MRT	Manual removal of placenta	46.2	23.4

Country	ISO 3166-1 alpha-3	Intervention Name	Baseline coverage (%)	Ending coverage (%)
Mauritania	MRT	Removal of retained products of conception	28.1	14.2
Mauritania	MRT	Cesarean delivery	8.4	4.3
Mauritania	MRT	Blood transfusion	7.6	3.8
Mauritania	MRT	Induction of labor for pregnancies lasting 41+ weeks	6.7	3.4
Mauritania	MRT	Complementary feeding - education only	34.3	19.8
Mauritania	MRT	Complementary feeding - supplementary feeding and education	34.3	19.8
Mauritania	MRT	Improved sanitation - Utilization of latrines or toilets	48.4	48.4
Mauritania	MRT	Improved water source	70.7	70.7
Mauritania	MRT	Water connection in the home	37.0	37.0
Mauritania	MRT	Hand washing with soap	41.0	41.0
Mauritania	MRT	Hygienic disposal of children's stools	20.2	20.2
Mauritania	MRT	ITN/IRS - Households protected from malaria	48.9	28.2
Mauritania	MRT	Injectable antibiotics for neonatal sepsis	69.3	33.3
Mauritania	MRT	ORS - oral rehydration solution	25.4	12.9
Mauritania	MRT	Zinc for treatment of diarrhea	23.2	11.7
Mauritania	MRT	Oral antibiotics for pneumonia	33.7	17.1
Mauritania	MRT	ACTs- Artemisinin compounds for treatment of malaria	0.7	0.4
Mauritania	MRT	SAM - treatment for severe acute malnutrition	22.4	11.3
Mauritania	MRT	BCG vaccine	90.0	43.3
Mauritania	MRT	Polio vaccine	81.0	39.0
Mauritania	MRT	DPT vaccine	81.0	39.0
Mauritania	MRT	H. influenzae type b vaccine	81.0	67.5
Mauritania	MRT	HepB vaccine	81.0	39.0
Mauritania	MRT	Pneumococcal vaccine	77.0	64.2
Mauritania	MRT	Rotavirus vaccine	76.0	63.4
Mauritania	MRT	Measles vaccine	78.0	37.5
Mauritania	MRT	Global wasting (<-2 SD) rate	14.9	22.4
Mauritania	MRT	Contraceptive prevalence (CPR)	18.1	11.0
Mexico	MEX	Safe abortion services	0.6	0.4
Mexico	MEX	TT - Tetanus toxoid vaccination	96.0	46.2
Mexico	MEX	Syphilis detection and treatment	24.3	11.7
Mexico	MEX	Hypertensive disorder case management	22.6	10.9
Mexico	MEX	Diabetes case management	17.7	8.5
Mexico	MEX	Malaria case management	73.1	35.2
Mexico	MEX	MgSO4 management of pre-eclampsia	44.8	21.5
Mexico	MEX	Thermal protection	95.8	48.5
Mexico	MEX	Clean cord care	92.5	46.8
Mexico	MEX	Clean birth environment	79.5	40.2
Mexico	MEX	Immediate drying and additional stimulation	88.8	45.0
Mexico	MEX	Neonatal resuscitation	53.3	27.0

Country	ISO 3166-1 alpha-3	Intervention Name	Baseline coverage (%)	Ending coverage (%)
Mexico	MEX	Antibiotics for preterm or prolonged PROM	72.5	36.7
Mexico	MEX	Parenteral administration of anti-convulsants	69.3	35.1
Mexico	MEX	Parenteral administration of uterotonics	86.6	43.8
Mexico	MEX	Parenteral administration of antibiotics	72.5	36.7
Mexico	MEX	Assisted vaginal delivery	24.5	12.4
Mexico	MEX	Manual removal of placenta	36.3	18.4
Mexico	MEX	Removal of retained products of conception	32.1	16.3
Mexico	MEX	Cesarean delivery	8.4	4.3
Mexico	MEX	Blood transfusion	12.2	6.2
Mexico	MEX	Induction of labor for pregnancies lasting 41+ weeks	1.7	0.9
Mexico	MEX	Complementary feeding - education only	72.1	41.6
Mexico	MEX	Complementary feeding - supplementary feeding and education	72.1	41.6
Mexico	MEX	Improved sanitation - Utilization of latrines or toilets	91.2	91.2
Mexico	MEX	Improved water source	99.0	99.0
Mexico	MEX	Water connection in the home	95.0	95.0
Mexico	MEX	Hand washing with soap	90.3	90.3
Mexico	MEX	Hygienic disposal of children's stools	14.0	14.0
Mexico	MEX	Injectable antibiotics for neonatal sepsis	96.9	46.6
Mexico	MEX	ORS - oral rehydration solution	61.4	31.1
Mexico	MEX	Zinc for treatment of diarrhea	10.8	5.5
Mexico	MEX	Oral antibiotics for pneumonia	73.1	37.0
Mexico	MEX	BCG vaccine	96.0	46.2
Mexico	MEX	Polio vaccine	88.0	42.3
Mexico	MEX	DPT vaccine	88.0	42.3
Mexico	MEX	H. influenzae type b vaccine	88.0	73.4
Mexico	MEX	HepB vaccine	55.0	26.5
Mexico	MEX	Pneumococcal vaccine	88.0	73.4
Mexico	MEX	Rotavirus vaccine	77.0	64.2
Mexico	MEX	Measles vaccine	96.0	46.2
Mexico	MEX	Global wasting (<-2 SD) rate	1.1	1.6
Mexico	MEX	Contraceptive prevalence (CPR)	69.75	42.4
Mongolia	MNG	Safe abortion services	100.0	60.8
Mongolia	MNG	Syphilis detection and treatment	24.5	11.8
Mongolia	MNG	Iron supplementation in pregnancy	25.9	12.5
Mongolia	MNG	Hypertensive disorder case management	21.3	10.2
Mongolia	MNG	Diabetes case management	16.6	8.0
Mongolia	MNG	Malaria case management	68.6	33.0
Mongolia	MNG	MgSO4 management of pre-eclampsia	42.1	20.2
Mongolia	MNG	Thermal protection	97.3	49.3
Mongolia	MNG	Clean cord care	93.9	47.5

**Country**	**ISO 3166-1 alpha-3**	**Intervention Name**	**Baseline coverage (%)**	**Ending coverage (%)**
Mongolia	MNG	Clean birth environment	80.7	40.9
Mongolia	MNG	Immediate drying and additional stimulation	90.1	45.6
Mongolia	MNG	Neonatal resuscitation	54.1	27.4
Mongolia	MNG	Antibiotics for preterm or prolonged PROM	73.6	37.3
Mongolia	MNG	Parenteral administration of anti-convulsants	70.4	35.6
Mongolia	MNG	Parenteral administration of uterotonics	87.9	44.5
Mongolia	MNG	Parenteral administration of antibiotics	73.6	37.3
Mongolia	MNG	Assisted vaginal delivery	24.9	12.6
Mongolia	MNG	Manual removal of placenta	36.8	18.6
Mongolia	MNG	Removal of retained products of conception	32.6	16.5
Mongolia	MNG	Cesarean delivery	8.6	4.4
Mongolia	MNG	Blood transfusion	12.4	6.3
Mongolia	MNG	Induction of labor for pregnancies lasting 41+ weeks	1.7	0.9
Mongolia	MNG	Complementary feeding - education only	50.8	29.3
Mongolia	MNG	Complementary feeding - supplementary feeding and education	50.8	29.3
Mongolia	MNG	Vitamin A supplementation	83.0	47.9
Mongolia	MNG	Improved sanitation - Utilization of latrines or toilets	58.5	58.5
Mongolia	MNG	Improved water source	83.3	83.3
Mongolia	MNG	Water connection in the home	20.9	20.9
Mongolia	MNG	Hand washing with soap	78.9	78.9
Mongolia	MNG	Hygienic disposal of children's stools	51.3	51.3
Mongolia	MNG	Injectable antibiotics for neonatal sepsis	98.4	47.3
Mongolia	MNG	ORS - oral rehydration solution	58.2	29.5
Mongolia	MNG	Antibiotics for treatment of dysentery	20.1	10.2
Mongolia	MNG	Zinc for treatment of diarrhea	16.5	8.4
Mongolia	MNG	Oral antibiotics for pneumonia	70.3	35.6
Mongolia	MNG	Vitamin A for treatment of measles	83.0	42.0
Mongolia	MNG	BCG vaccine	99.0	47.6
Mongolia	MNG	Polio vaccine	99.0	47.6
Mongolia	MNG	DPT vaccine	99.0	47.6
Mongolia	MNG	H. influenzae type b vaccine	99.0	82.6
Mongolia	MNG	HepB vaccine	99.0	47.6
Mongolia	MNG	Pneumococcal vaccine	26.0	21.7
Mongolia	MNG	Measles vaccine	99.0	47.6
Mongolia	MNG	Global wasting (<-2 SD) rate	1.0	1.5
Mongolia	MNG	Contraceptive prevalence (CPR)	55.4	33.7
Montenegro	MNE	Safe abortion services	100.0	60.8
Montenegro	MNE	Syphilis detection and treatment	24.0	11.5
Montenegro	MNE	Hypertensive disorder case management	22.6	10.9
Montenegro	MNE	Diabetes case management	17.6	8.5

Country	ISO 3166-1 alpha-3	Intervention Name	Baseline coverage (%)	Ending coverage (%)
Montenegro	MNE	Malaria case management	73.0	35.1
Montenegro	MNE	MgSO4 management of pre-eclampsia	44.8	21.5
Montenegro	MNE	Thermal protection	97.5	49.4
Montenegro	MNE	Clean cord care	94.1	47.6
Montenegro	MNE	Clean birth environment	80.9	41.0
Montenegro	MNE	Immediate drying and additional stimulation	90.3	45.7
Montenegro	MNE	Neonatal resuscitation	54.2	27.4
Montenegro	MNE	Antibiotics for preterm or prolonged PROM	73.8	37.4
Montenegro	MNE	Parenteral administration of anti-convulsants	70.5	35.7
Montenegro	MNE	Parenteral administration of uterotonics	88.1	44.6
Montenegro	MNE	Parenteral administration of antibiotics	73.8	37.4
Montenegro	MNE	Assisted vaginal delivery	24.9	12.6
Montenegro	MNE	Manual removal of placenta	36.9	18.7
Montenegro	MNE	Removal of retained products of conception	32.7	16.6
Montenegro	MNE	Cesarean delivery	8.6	4.4
Montenegro	MNE	Blood transfusion	12.4	6.3
Montenegro	MNE	Induction of labor for pregnancies lasting 41+ weeks	1.7	0.9
Montenegro	MNE	Complementary feeding - education only	81.3	46.9
Montenegro	MNE	Complementary feeding - supplementary feeding and education	81.3	46.9
Montenegro	MNE	Improved sanitation - Utilization of latrines or toilets	97.8	97.8
Montenegro	MNE	Improved water source	97.0	97.0
Montenegro	MNE	Water connection in the home	82.4	82.4
Montenegro	MNE	Hand washing with soap	99.6	99.6
Montenegro	MNE	Hygienic disposal of children's stools	21.3	21.3
Montenegro	MNE	Injectable antibiotics for neonatal sepsis	98.6	47.4
Montenegro	MNE	ORS - oral rehydration solution	31.9	16.1
Montenegro	MNE	Oral antibiotics for pneumonia	89.4	45.3
Montenegro	MNE	BCG vaccine	83.0	39.9
Montenegro	MNE	Polio vaccine	87.0	41.8
Montenegro	MNE	DPT vaccine	87.0	41.8
Montenegro	MNE	H. influenzae type b vaccine	87.0	72.5
Montenegro	MNE	HepB vaccine	73.0	35.1
Montenegro	MNE	Measles vaccine	58.0	27.9
Montenegro	MNE	Global wasting (<-2 SD) rate	2.9	4.4
Montenegro	MNE	Contraceptive prevalence (CPR)	27.3	16.6
Morocco	MAR	Safe abortion services	2.2	1.3
Morocco	MAR	TT - Tetanus toxoid vaccination	88.0	42.3
Morocco	MAR	Syphilis detection and treatment	16.7	8.0
Morocco	MAR	Iron supplementation in pregnancy	5.2	2.5
Morocco	MAR	Hypertensive disorder case management	7.2	3.5

Country	ISO 3166-1 alpha-3	Intervention Name	Baseline coverage (%)	Ending coverage (%)
Morocco	MAR	Diabetes case management	5.6	2.7
Morocco	MAR	Malaria case management	23.3	11.2
Morocco	MAR	MgSO4 management of pre-eclampsia	14.3	6.9
Morocco	MAR	Thermal protection	71.9	36.4
Morocco	MAR	Clean cord care	69.4	35.1
Morocco	MAR	Clean birth environment	59.6	30.2
Morocco	MAR	Immediate drying and additional stimulation	66.6	33.7
Morocco	MAR	Neonatal resuscitation	40.0	20.3
Morocco	MAR	Antibiotics for preterm or prolonged PROM	54.4	27.5
Morocco	MAR	Parenteral administration of anti-convulsants	52.0	26.3
Morocco	MAR	Parenteral administration of uterotonics	65.0	32.9
Morocco	MAR	Parenteral administration of antibiotics	54.4	27.5
Morocco	MAR	Assisted vaginal delivery	18.4	9.3
Morocco	MAR	Manual removal of placenta	27.2	13.8
Morocco	MAR	Removal of retained products of conception	24.1	12.2
Morocco	MAR	Cesarean delivery	6.3	3.2
Morocco	MAR	Blood transfusion	9.2	4.7
Morocco	MAR	Induction of labor for pregnancies lasting 41+ weeks	1.3	0.7
Morocco	MAR	Complementary feeding - education only	22.3	12.9
Morocco	MAR	Complementary feeding - supplementary feeding and education	22.3	12.9
Morocco	MAR	Vitamin A supplementation	99.0	57.1
Morocco	MAR	Improved sanitation - Utilization of latrines or toilets	88.5	88.5
Morocco	MAR	Improved water source	86.8	86.8
Morocco	MAR	Water connection in the home	67.7	67.7
Morocco	MAR	Hygienic disposal of children's stools	6.3	6.3
Morocco	MAR	Injectable antibiotics for neonatal sepsis	72.7	35.0
Morocco	MAR	ORS - oral rehydration solution	22.9	11.6
Morocco	MAR	Vitamin A for treatment of measles	99.0	50.1
Morocco	MAR	BCG vaccine	99.0	47.6
Morocco	MAR	Polio vaccine	99.0	47.6
Morocco	MAR	DPT vaccine	99.0	47.6
Morocco	MAR	H. influenzae type b vaccine	99.0	82.6
Morocco	MAR	HepB vaccine	99.0	47.6
Morocco	MAR	Pneumococcal vaccine	99.0	82.6
Morocco	MAR	Rotavirus vaccine	99.0	82.6
Morocco	MAR	Measles vaccine	99.0	47.6
Morocco	MAR	Global wasting (<-2 SD) rate	10.1	15.1
Morocco	MAR	Contraceptive prevalence (CPR)	69.85	42.4
Mozambique	MOZ	Safe abortion services	3.3	2.0
Mozambique	MOZ	TT - Tetanus toxoid vaccination	86.0	41.4

Country	ISO 3166-1 alpha-3	Intervention Name	Baseline coverage (%)	Ending coverage (%)
Mozambique	MOZ	IPTp - Intermittent preventive treatment of malaria during pregnancy	35.8	17.2
Mozambique	MOZ	Syphilis detection and treatment	22.4	10.8
Mozambique	MOZ	Iron supplementation in pregnancy	25.9	12.5
Mozambique	MOZ	Hypertensive disorder case management	12.9	6.2
Mozambique	MOZ	Diabetes case management	10.1	4.9
Mozambique	MOZ	Malaria case management	41.7	20.1
Mozambique	MOZ	MgSO4 management of pre-eclampsia	25.6	12.3
Mozambique	MOZ	Thermal protection	64.1	32.5
Mozambique	MOZ	Clean cord care	61.9	31.3
Mozambique	MOZ	Clean birth environment	53.2	26.9
Mozambique	MOZ	Immediate drying and additional stimulation	59.4	30.1
Mozambique	MOZ	Neonatal resuscitation	35.7	18.1
Mozambique	MOZ	Antibiotics for preterm or prolonged PROM	48.5	24.6
Mozambique	MOZ	Parenteral administration of anti-convulsants	46.4	23.5
Mozambique	MOZ	Parenteral administration of uterotonics	57.9	29.3
Mozambique	MOZ	Parenteral administration of antibiotics	48.5	24.6
Mozambique	MOZ	Assisted vaginal delivery	16.4	8.3
Mozambique	MOZ	Manual removal of placenta	24.3	12.3
Mozambique	MOZ	Removal of retained products of conception	21.5	10.9
Mozambique	MOZ	Cesarean delivery	5.6	2.8
Mozambique	MOZ	Blood transfusion	8.2	4.2
Mozambique	MOZ	Induction of labor for pregnancies lasting 41+ weeks	1.1	0.6
Mozambique	MOZ	Complementary feeding - education only	30.4	17.5
Mozambique	MOZ	Complementary feeding - supplementary feeding and education	30.4	17.5
Mozambique	MOZ	Vitamin A supplementation	61.0	35.2
Mozambique	MOZ	Improved sanitation - Utilization of latrines or toilets	29.4	29.4
Mozambique	MOZ	Improved water source	55.7	55.7
Mozambique	MOZ	Water connection in the home	19.8	19.8
Mozambique	MOZ	Hand washing with soap	31.7	31.7
Mozambique	MOZ	Hygienic disposal of children's stools	45.0	45.0
Mozambique	MOZ	ITN/IRS - Households protected from malaria	68.7	39.6
Mozambique	MOZ	Injectable antibiotics for neonatal sepsis	64.8	31.2
Mozambique	MOZ	ORS - oral rehydration solution	45.9	23.2
Mozambique	MOZ	Antibiotics for treatment of dysentery	31.6	16.0
Mozambique	MOZ	Zinc for treatment of diarrhea	30.9	15.6
Mozambique	MOZ	Oral antibiotics for pneumonia	59.4	30.1
Mozambique	MOZ	Vitamin A for treatment of measles	61.0	30.9
Mozambique	MOZ	ACTs- Artemisinin compounds for treatment of malaria	15.4	7.8
Mozambique	MOZ	BCG vaccine	95.0	45.7
Mozambique	MOZ	Polio vaccine	80.0	38.5

Country	ISO 3166-1 alpha-3	Intervention Name	Baseline coverage (%)	Ending coverage (%)
Mozambique	MOZ	DPT vaccine	80.0	38.5
Mozambique	MOZ	H. influenzae type b vaccine	80.0	66.7
Mozambique	MOZ	HepB vaccine	80.0	38.5
Mozambique	MOZ	Pneumococcal vaccine	80.0	66.7
Mozambique	MOZ	Rotavirus vaccine	80.0	66.7
Mozambique	MOZ	Measles vaccine	85.0	40.9
Mozambique	MOZ	Global wasting (<-2 SD) rate	6.0	9.1
Mozambique	MOZ	Contraceptive prevalence (CPR)	29.95	18.2
Myanmar	MMR	Safe abortion services	39.0	23.7
Myanmar	MMR	TT - Tetanus toxoid vaccination	90.0	43.3
Myanmar	MMR	Syphilis detection and treatment	20.5	9.9
Myanmar	MMR	Iron supplementation in pregnancy	59.3	28.5
Myanmar	MMR	Hypertensive disorder case management	14.3	6.9
Myanmar	MMR	Diabetes case management	11.2	5.4
Myanmar	MMR	Malaria case management	46.1	22.2
Myanmar	MMR	MgSO4 management of pre-eclampsia	28.3	13.6
Myanmar	MMR	Thermal protection	36.6	18.5
Myanmar	MMR	Clean cord care	35.4	17.9
Myanmar	MMR	Clean birth environment	30.4	15.4
Myanmar	MMR	Immediate drying and additional stimulation	33.9	17.2
Myanmar	MMR	Neonatal resuscitation	20.4	10.3
Myanmar	MMR	Antibiotics for preterm or prolonged PROM	27.7	14.0
Myanmar	MMR	Parenteral administration of anti-convulsants	26.5	13.4
Myanmar	MMR	Parenteral administration of uterotonics	33.1	16.8
Myanmar	MMR	Parenteral administration of antibiotics	27.7	14.0
Myanmar	MMR	Assisted vaginal delivery	9.4	4.8
Myanmar	MMR	Manual removal of placenta	13.9	7.0
Myanmar	MMR	Removal of retained products of conception	12.3	6.2
Myanmar	MMR	Cesarean delivery	3.2	1.6
Myanmar	MMR	Blood transfusion	4.7	2.4
Myanmar	MMR	Induction of labor for pregnancies lasting 41+ weeks	0.7	0.4
Myanmar	MMR	Complementary feeding - education only	24.8	14.3
Myanmar	MMR	Complementary feeding - supplementary feeding and education	24.8	14.3
Myanmar	MMR	Vitamin A supplementation	89.0	51.4
Myanmar	MMR	Improved sanitation - Utilization of latrines or toilets	64.3	64.3
Myanmar	MMR	Improved water source	81.8	81.8
Myanmar	MMR	Water connection in the home	20.1	20.1
Myanmar	MMR	Hand washing with soap	79.9	79.9
Myanmar	MMR	Hygienic disposal of children's stools	42.1	42.1
Myanmar	MMR	ITN/IRS - Households protected from malaria	26.8	15.5

Country	ISO 3166-1 alpha-3	Intervention Name	Baseline coverage (%)	Ending coverage (%)
Myanmar	MMR	Injectable antibiotics for neonatal sepsis	37.1	17.8
Myanmar	MMR	ORS - oral rehydration solution	61.9	31.3
Myanmar	MMR	Zinc for treatment of diarrhea	8.4	4.3
Myanmar	MMR	Oral antibiotics for pneumonia	58.6	29.7
Myanmar	MMR	Vitamin A for treatment of measles	89.0	45.1
Myanmar	MMR	ACTs- Artemisinin compounds for treatment of malaria	0.2	0.1
Myanmar	MMR	SAM - treatment for severe acute malnutrition	3.5	1.8
Myanmar	MMR	BCG vaccine	90.0	43.3
Myanmar	MMR	Polio vaccine	91.0	43.8
Myanmar	MMR	DPT vaccine	91.0	43.8
Myanmar	MMR	H. influenzae type b vaccine	91.0	75.9
Myanmar	MMR	HepB vaccine	91.0	43.8
Myanmar	MMR	Pneumococcal vaccine	91.0	75.9
Myanmar	MMR	Measles vaccine	83.0	39.9
Myanmar	MMR	Global wasting (<-2 SD) rate	6.8	10.1
Myanmar	MMR	Contraceptive prevalence (CPR)	56.65	34.4
Namibia	NAM	Safe abortion services	42.5	25.8
Namibia	NAM	TT - Tetanus toxoid vaccination	88.0	42.3
Namibia	NAM	IPTp - Intermittent preventive treatment of malaria during pregnancy	5.1	2.5
Namibia	NAM	Syphilis detection and treatment	92.6	44.5
Namibia	NAM	Iron supplementation in pregnancy	38.6	18.6
Namibia	NAM	Hypertensive disorder case management	42.1	20.2
Namibia	NAM	Diabetes case management	32.2	15.5
Namibia	NAM	Malaria case management	45.5	21.9
Namibia	NAM	MgSO4 management of pre-eclampsia	31.8	15.3
Namibia	NAM	Thermal protection	86.7	43.9
Namibia	NAM	Clean cord care	87.2	44.1
Namibia	NAM	Clean birth environment	69.1	35.0
Namibia	NAM	Immediate drying and additional stimulation	86.5	43.8
Namibia	NAM	Neonatal resuscitation	78.2	39.6
Namibia	NAM	Antibiotics for preterm or prolonged PROM	81.9	41.5
Namibia	NAM	Parenteral administration of anti-convulsants	86.0	43.5
Namibia	NAM	Parenteral administration of uterotonics	76.9	38.9
Namibia	NAM	Parenteral administration of antibiotics	81.9	41.5
Namibia	NAM	Assisted vaginal delivery	39.4	19.9
Namibia	NAM	Manual removal of placenta	65.3	33.1
Namibia	NAM	Removal of retained products of conception	23.0	11.6
Namibia	NAM	Cesarean delivery	41.8	21.2
Namibia	NAM	Blood transfusion	18.9	9.6
Namibia	NAM	Induction of labor for pregnancies lasting 41+ weeks	1.5	0.8

Country	ISO 3166-1 alpha-3	Intervention Name	Baseline coverage (%)	Ending coverage (%)
Namibia	NAM	Complementary feeding - education only	31.1	17.9
Namibia	NAM	Complementary feeding - supplementary feeding and education	31.1	17.9
Namibia	NAM	Vitamin A supplementation	27.0	15.6
Namibia	NAM	Improved sanitation - Utilization of latrines or toilets	34.5	34.5
Namibia	NAM	Improved water source	82.5	82.5
Namibia	NAM	Water connection in the home	65.4	65.4
Namibia	NAM	Hand washing with soap	54.1	54.1
Namibia	NAM	Hygienic disposal of children's stools	12.6	12.6
Namibia	NAM	ITN/IRS - Households protected from malaria	32.7	18.9
Namibia	NAM	Injectable antibiotics for neonatal sepsis	87.7	42.2
Namibia	NAM	ORS - oral rehydration solution	71.6	36.2
Namibia	NAM	Antibiotics for treatment of dysentery	13.4	6.8
Namibia	NAM	Zinc for treatment of diarrhea	0.2	0.1
Namibia	NAM	Oral antibiotics for pneumonia	67.7	34.3
Namibia	NAM	Vitamin A for treatment of measles	27.0	13.7
Namibia	NAM	ACTs- Artemisinin compounds for treatment of malaria	3.2	1.6
Namibia	NAM	BCG vaccine	94.0	45.2
Namibia	NAM	Polio vaccine	84.0	40.4
Namibia	NAM	DPT vaccine	89.0	42.8
Namibia	NAM	H. influenzae type b vaccine	89.0	74.2
Namibia	NAM	HepB vaccine	89.0	42.8
Namibia	NAM	Pneumococcal vaccine	61.0	50.9
Namibia	NAM	Rotavirus vaccine	92.0	76.7
Namibia	NAM	Measles vaccine	80.0	38.5
Namibia	NAM	Global wasting (<-2 SD) rate	7.1	10.7
Namibia	NAM	Contraceptive prevalence (CPR)	60.3	36.6
Nepal	NPL	Safe abortion services	35.1	21.3
Nepal	NPL	TT - Tetanus toxoid vaccination	89.0	42.8
Nepal	NPL	Syphilis detection and treatment	8.2	3.9
Nepal	NPL	Iron supplementation in pregnancy	70.9	34.1
Nepal	NPL	Hypertensive disorder case management	15.2	7.3
Nepal	NPL	Diabetes case management	8.5	4.1
Nepal	NPL	Malaria case management	21.8	10.5
Nepal	NPL	MgSO4 management of pre-eclampsia	41.6	20.0
Nepal	NPL	Thermal protection	56.7	28.7
Nepal	NPL	Clean cord care	56.6	28.7
Nepal	NPL	Clean birth environment	54.1	27.4
Nepal	NPL	Immediate drying and additional stimulation	55.5	28.1
Nepal	NPL	Neonatal resuscitation	53.3	27.0
Nepal	NPL	Antibiotics for preterm or prolonged PROM	44.3	22.4

Country	ISO 3166-1 alpha-3	Intervention Name	Baseline coverage (%)	Ending coverage (%)
Nepal	NPL	Parenteral administration of anti-convulsants	52.8	26.7
Nepal	NPL	Parenteral administration of uterotonics	55.8	28.2
Nepal	NPL	Parenteral administration of antibiotics	44.3	22.4
Nepal	NPL	Assisted vaginal delivery	39.9	20.2
Nepal	NPL	Manual removal of placenta	15.3	7.7
Nepal	NPL	Removal of retained products of conception	37.5	19.0
Nepal	NPL	Cesarean delivery	7.8	3.9
Nepal	NPL	Blood transfusion	13.8	7.0
Nepal	NPL	Induction of labor for pregnancies lasting 41+ weeks	18.9	9.6
Nepal	NPL	Complementary feeding - education only	46.5	26.8
Nepal	NPL	Complementary feeding - supplementary feeding and education	46.5	26.8
Nepal	NPL	Vitamin A supplementation	81.0	46.7
Nepal	NPL	Improved sanitation - Utilization of latrines or toilets	62.1	62.1
Nepal	NPL	Improved water source	88.8	88.8
Nepal	NPL	Water connection in the home	42.1	42.1
Nepal	NPL	Hand washing with soap	72.5	72.5
Nepal	NPL	Hygienic disposal of children's stools	44.4	44.4
Nepal	NPL	Injectable antibiotics for neonatal sepsis	57.4	27.6
Nepal	NPL	ORS - oral rehydration solution	37.0	18.7
Nepal	NPL	Antibiotics for treatment of dysentery	21.3	10.8
Nepal	NPL	Zinc for treatment of diarrhea	17.6	8.9
Nepal	NPL	Oral antibiotics for pneumonia	55.1	27.9
Nepal	NPL	Vitamin A for treatment of measles	81.0	41.0
Nepal	NPL	ACTs- Artemisinin compounds for treatment of malaria	0.2	0.1
Nepal	NPL	SAM - treatment for severe acute malnutrition	0.9	0.5
Nepal	NPL	BCG vaccine	96.0	46.2
Nepal	NPL	Polio vaccine	91.0	43.8
Nepal	NPL	DPT vaccine	91.0	43.8
Nepal	NPL	H. influenzae type b vaccine	91.0	75.9
Nepal	NPL	HepB vaccine	91.0	43.8
Nepal	NPL	Pneumococcal vaccine	82.0	68.4
Nepal	NPL	Measles vaccine	90.0	43.3
Nepal	NPL	Global wasting (<-2 SD) rate	9.8	14.7
Nepal	NPL	Contraceptive prevalence (CPR)	55.1	33.5
Nicaragua	NIC	Safe abortion services	0.6	0.4
Nicaragua	NIC	TT - Tetanus toxoid vaccination	90.0	43.3
Nicaragua	NIC	Syphilis detection and treatment	21.7	10.4
Nicaragua	NIC	Iron supplementation in pregnancy	61.6	29.6
Nicaragua	NIC	Hypertensive disorder case management	21.1	10.1
Nicaragua	NIC	Diabetes case management	16.4	7.9

Country	ISO 3166-1 alpha-3	Intervention Name	Baseline coverage (%)	Ending coverage (%)
Nicaragua	NIC	Malaria case management	68.1	32.8
Nicaragua	NIC	MgSO4 management of pre-eclampsia	41.7	20.1
Nicaragua	NIC	Thermal protection	74.4	37.7
Nicaragua	NIC	Clean cord care	71.8	36.3
Nicaragua	NIC	Clean birth environment	61.7	31.2
Nicaragua	NIC	Immediate drying and additional stimulation	68.9	34.9
Nicaragua	NIC	Neonatal resuscitation	41.4	21.0
Nicaragua	NIC	Antibiotics for preterm or prolonged PROM	56.3	28.5
Nicaragua	NIC	Parenteral administration of anti-convulsants	53.8	27.2
Nicaragua	NIC	Parenteral administration of uterotonics	67.2	34.0
Nicaragua	NIC	Parenteral administration of antibiotics	56.3	28.5
Nicaragua	NIC	Assisted vaginal delivery	19.0	9.6
Nicaragua	NIC	Manual removal of placenta	28.1	14.2
Nicaragua	NIC	Removal of retained products of conception	24.9	12.6
Nicaragua	NIC	Cesarean delivery	6.5	3.3
Nicaragua	NIC	Blood transfusion	9.5	4.8
Nicaragua	NIC	Induction of labor for pregnancies lasting 41+ weeks	1.3	0.7
Nicaragua	NIC	Vitamin A supplementation	3.0	1.7
Nicaragua	NIC	Improved sanitation - Utilization of latrines or toilets	74.4	74.4
Nicaragua	NIC	Improved water source	81.5	81.5
Nicaragua	NIC	Water connection in the home	56.5	56.5
Nicaragua	NIC	Hygienic disposal of children's stools	43.2	43.2
Nicaragua	NIC	Injectable antibiotics for neonatal sepsis	75.2	36.2
Nicaragua	NIC	ORS - oral rehydration solution	65.4	33.1
Nicaragua	NIC	Antibiotics for treatment of dysentery	10.6	5.4
Nicaragua	NIC	Vitamin A for treatment of measles	3.0	1.5
Nicaragua	NIC	BCG vaccine	98.0	47.1
Nicaragua	NIC	Polio vaccine	99.0	47.6
Nicaragua	NIC	DPT vaccine	98.0	47.1
Nicaragua	NIC	H. influenzae type b vaccine	98.0	81.7
Nicaragua	NIC	HepB vaccine	98.0	47.1
Nicaragua	NIC	Pneumococcal vaccine	98.0	81.7
Nicaragua	NIC	Rotavirus vaccine	98.0	81.7
Nicaragua	NIC	Measles vaccine	99.0	47.6
Nicaragua	NIC	Global wasting (<-2 SD) rate	1.4	2.1
Nicaragua	NIC	Contraceptive prevalence (CPR)	81.7	49.6
Niger	NER	TT - Tetanus toxoid vaccination	81.0	39.0
Niger	NER	IPTp - Intermittent preventive treatment of malaria during pregnancy	36.9	17.7
Niger	NER	Syphilis detection and treatment	20.8	10.0
Niger	NER	Iron supplementation in pregnancy	28.6	13.8

Country	ISO 3166-1 alpha-3	Intervention Name	Baseline coverage (%)	Ending coverage (%)
Niger	NER	Hypertensive disorder case management	8.0	3.8
Niger	NER	Diabetes case management	6.2	3.0
Niger	NER	Malaria case management	25.8	12.4
Niger	NER	MgSO4 management of pre-eclampsia	15.8	7.6
Niger	NER	Thermal protection	29.4	14.9
Niger	NER	Clean cord care	28.4	14.4
Niger	NER	Clean birth environment	24.4	12.4
Niger	NER	Immediate drying and additional stimulation	27.2	13.8
Niger	NER	Neonatal resuscitation	16.4	8.3
Niger	NER	Antibiotics for preterm or prolonged PROM	22.3	11.3
Niger	NER	Parenteral administration of anti-convulsants	21.3	10.8
Niger	NER	Parenteral administration of uterotonics	26.6	13.5
Niger	NER	Parenteral administration of antibiotics	22.3	11.3
Niger	NER	Assisted vaginal delivery	7.5	3.8
Niger	NER	Manual removal of placenta	11.1	5.6
Niger	NER	Removal of retained products of conception	9.9	5.0
Niger	NER	Cesarean delivery	2.6	1.3
Niger	NER	Blood transfusion	3.7	1.9
Niger	NER	Induction of labor for pregnancies lasting 41+ weeks	0.5	0.3
Niger	NER	Complementary feeding - education only	9.9	5.7
Niger	NER	Complementary feeding - supplementary feeding and education	9.9	5.7
Niger	NER	Vitamin A supplementation	53.0	30.6
Niger	NER	Improved sanitation - Utilization of latrines or toilets	13.6	13.6
Niger	NER	Improved water source	50.3	50.3
Niger	NER	Water connection in the home	16.3	16.3
Niger	NER	Hygienic disposal of children's stools	19.4	19.4
Niger	NER	ITN/IRS - Households protected from malaria	61.5	35.5
Niger	NER	Injectable antibiotics for neonatal sepsis	29.8	14.3
Niger	NER	ORS - oral rehydration solution	44.3	22.4
Niger	NER	Antibiotics for treatment of dysentery	16.8	8.5
Niger	NER	Zinc for treatment of diarrhea	10.3	5.2
Niger	NER	Oral antibiotics for pneumonia	53.2	26.9
Niger	NER	Vitamin A for treatment of measles	53.0	26.8
Niger	NER	ACTs- Artemisinin compounds for treatment of malaria	11.6	5.9
Niger	NER	SAM - treatment for severe acute malnutrition	40.0	20.3
Niger	NER	BCG vaccine	87.0	41.8
Niger	NER	Polio vaccine	79.0	38.0
Niger	NER	DPT vaccine	79.0	38.0
Niger	NER	H. influenzae type b vaccine	79.0	65.9
Niger	NER	HepB vaccine	79.0	38.0

Country	ISO 3166-1 alpha-3	Intervention Name	Baseline coverage (%)	Ending coverage (%)
Niger	NER	Pneumococcal vaccine	79.0	65.9
Niger	NER	Rotavirus vaccine	79.0	65.9
Niger	NER	Meningococcal A	80.0	38.5
Niger	NER	Measles vaccine	78.0	37.5
Niger	NER	Global wasting (<-2 SD) rate	18.5	27.7
Niger	NER	Contraceptive prevalence (CPR)	17.75	10.8
Nigeria	NGA	TT - Tetanus toxoid vaccination	55.0	26.5
Nigeria	NGA	IPTp - Intermittent preventive treatment of malaria during pregnancy	40.4	19.4
Nigeria	NGA	Syphilis detection and treatment	18.3	8.8
Nigeria	NGA	Iron supplementation in pregnancy	20.5	9.9
Nigeria	NGA	Hypertensive disorder case management	13.6	6.5
Nigeria	NGA	Diabetes case management	10.6	5.1
Nigeria	NGA	Malaria case management	44.0	21.2
Nigeria	NGA	MgSO4 management of pre-eclampsia	27.0	13.0
Nigeria	NGA	Thermal protection	38.9	19.7
Nigeria	NGA	Clean cord care	37.6	19.0
Nigeria	NGA	Clean birth environment	32.3	16.4
Nigeria	NGA	Immediate drying and additional stimulation	36.1	18.3
Nigeria	NGA	Neonatal resuscitation	21.7	11.0
Nigeria	NGA	Antibiotics for preterm or prolonged PROM	29.5	14.9
Nigeria	NGA	Parenteral administration of anti-convulsants	28.2	14.3
Nigeria	NGA	Parenteral administration of uterotonics	35.2	17.8
Nigeria	NGA	Parenteral administration of antibiotics	29.5	14.9
Nigeria	NGA	Assisted vaginal delivery	10.0	5.1
Nigeria	NGA	Manual removal of placenta	14.7	7.4
Nigeria	NGA	Removal of retained products of conception	13.1	6.6
Nigeria	NGA	Cesarean delivery	3.4	1.7
Nigeria	NGA	Blood transfusion	5.0	2.5
Nigeria	NGA	Induction of labor for pregnancies lasting 41+ weeks	0.7	0.4
Nigeria	NGA	Complementary feeding - education only	22.6	13.0
Nigeria	NGA	Complementary feeding - supplementary feeding and education	22.6	13.0
Nigeria	NGA	Vitamin A supplementation	83.0	47.9
Nigeria	NGA	Improved sanitation - Utilization of latrines or toilets	39.2	39.2
Nigeria	NGA	Improved water source	71.4	71.4
Nigeria	NGA	Water connection in the home	8.0	8.0
Nigeria	NGA	Hand washing with soap	29.3	29.3
Nigeria	NGA	Hygienic disposal of children's stools	56.5	56.5
Nigeria	NGA	ITN/IRS - Households protected from malaria	60.6	35.0
Nigeria	NGA	Injectable antibiotics for neonatal sepsis	39.4	18.9
Nigeria	NGA	ORS - oral rehydration solution	40.0	20.3

Country	ISO 3166-1 alpha-3	Intervention Name	Baseline coverage (%)	Ending coverage (%)
Nigeria	NGA	Antibiotics for treatment of dysentery	40.8	20.7
Nigeria	NGA	Zinc for treatment of diarrhea	31.1	15.7
Nigeria	NGA	Oral antibiotics for pneumonia	74.5	37.7
Nigeria	NGA	Vitamin A for treatment of measles	83.0	42.0
Nigeria	NGA	ACTs- Artemisinin compounds for treatment of malaria	5.6	2.8
Nigeria	NGA	SAM - treatment for severe acute malnutrition	5.8	2.9
Nigeria	NGA	BCG vaccine	53.0	25.5
Nigeria	NGA	Polio vaccine	57.0	27.4
Nigeria	NGA	DPT vaccine	57.0	27.4
Nigeria	NGA	H. influenzae type b vaccine	57.0	47.5
Nigeria	NGA	HepB vaccine	57.0	27.4
Nigeria	NGA	Pneumococcal vaccine	57.0	47.5
Nigeria	NGA	Measles vaccine	42.0	20.2
Nigeria	NGA	Global wasting (<-2 SD) rate	10.9	16.4
Nigeria	NGA	Contraceptive prevalence (CPR)	22.3	13.5
Pakistan	PAK	Safe abortion services	35.1	21.3
Pakistan	PAK	TT - Tetanus toxoid vaccination	85.0	40.9
Pakistan	PAK	Syphilis detection and treatment	21.6	10.4
Pakistan	PAK	Iron supplementation in pregnancy	29.4	14.1
Pakistan	PAK	Hypertensive disorder case management	12.6	6.1
Pakistan	PAK	Diabetes case management	9.8	4.7
Pakistan	PAK	Malaria case management	40.5	19.5
Pakistan	PAK	MgSO4 management of pre-eclampsia	24.8	11.9
Pakistan	PAK	Thermal protection	65.1	33.0
Pakistan	PAK	Clean cord care	62.9	31.8
Pakistan	PAK	Clean birth environment	54.0	27.3
Pakistan	PAK	Immediate drying and additional stimulation	60.3	30.5
Pakistan	PAK	Neonatal resuscitation	36.2	18.3
Pakistan	PAK	Antibiotics for preterm or prolonged PROM	49.3	25.0
Pakistan	PAK	Parenteral administration of anti-convulsants	47.1	23.8
Pakistan	PAK	Parenteral administration of uterotonics	58.9	29.8
Pakistan	PAK	Parenteral administration of antibiotics	49.3	25.0
Pakistan	PAK	Assisted vaginal delivery	16.7	8.5
Pakistan	PAK	Manual removal of placenta	24.6	12.5
Pakistan	PAK	Removal of retained products of conception	21.8	11.0
Pakistan	PAK	Cesarean delivery	5.7	2.9
Pakistan	PAK	Blood transfusion	8.3	4.2
Pakistan	PAK	Induction of labor for pregnancies lasting 41+ weeks	1.2	0.6
Pakistan	PAK	Complementary feeding - education only	20.8	12.0
Pakistan	PAK	Complementary feeding - supplementary feeding and education	20.8	12.0

Country	ISO 3166-1 alpha-3	Intervention Name	Baseline coverage (%)	Ending coverage (%)
Pakistan	PAK	Vitamin A supplementation	92.0	53.1
Pakistan	PAK	Improved sanitation - Utilization of latrines or toilets	59.9	59.9
Pakistan	PAK	Improved water source	91.5	91.5
Pakistan	PAK	Water connection in the home	26.0	26.0
Pakistan	PAK	Hand washing with soap	65.6	65.6
Pakistan	PAK	Hygienic disposal of children's stools	35.7	35.7
Pakistan	PAK	ITN/IRS - Households protected from malaria	8.4	4.8
Pakistan	PAK	Injectable antibiotics for neonatal sepsis	65.9	31.7
Pakistan	PAK	ORS - oral rehydration solution	37.4	18.9
Pakistan	PAK	Antibiotics for treatment of dysentery	33.7	17.1
Pakistan	PAK	Zinc for treatment of diarrhea	12.5	6.3
Pakistan	PAK	Oral antibiotics for pneumonia	78.6	39.8
Pakistan	PAK	Vitamin A for treatment of measles	92.0	46.6
Pakistan	PAK	BCG vaccine	86.0	41.4
Pakistan	PAK	Polio vaccine	75.0	36.1
Pakistan	PAK	DPT vaccine	75.0	36.1
Pakistan	PAK	H. influenzae type b vaccine	75.0	62.5
Pakistan	PAK	HepB vaccine	75.0	36.1
Pakistan	PAK	Pneumococcal vaccine	79.0	65.9
Pakistan	PAK	Rotavirus vaccine	58.0	48.4
Pakistan	PAK	Measles vaccine	76.0	36.6
Pakistan	PAK	Global wasting (<-2 SD) rate	7.0	10.5
Pakistan	PAK	Contraceptive prevalence (CPR)	37.05	22.5
Panama	PAN	Safe abortion services	0.6	0.4
Panama	PAN	Syphilis detection and treatment	23.1	11.1
Panama	PAN	Hypertensive disorder case management	21.1	10.1
Panama	PAN	Diabetes case management	16.5	7.9
Panama	PAN	Malaria case management	68.1	32.8
Panama	PAN	MgSO4 management of pre-eclampsia	41.8	20.1
Panama	PAN	Thermal protection	90.2	45.7
Panama	PAN	Clean cord care	87.0	44.0
Panama	PAN	Clean birth environment	74.8	37.9
Panama	PAN	Immediate drying and additional stimulation	83.5	42.3
Panama	PAN	Neonatal resuscitation	50.2	25.4
Panama	PAN	Antibiotics for preterm or prolonged PROM	68.3	34.6
Panama	PAN	Parenteral administration of anti-convulsants	65.2	33.0
Panama	PAN	Parenteral administration of uterotonics	81.5	41.3
Panama	PAN	Parenteral administration of antibiotics	68.3	34.6
Panama	PAN	Assisted vaginal delivery	23.1	11.7
Panama	PAN	Manual removal of placenta	34.1	17.3
Panama	PAN	Removal of retained products of conception	30.2	15.3

Country	ISO 3166-1 alpha-3	Intervention Name	Baseline coverage (%)	Ending coverage (%)
Panama	PAN	Cesarean delivery	7.9	4.0
Panama	PAN	Blood transfusion	11.5	5.8
Panama	PAN	Induction of labor for pregnancies lasting 41+ weeks	1.6	0.8
Panama	PAN	Improved sanitation - Utilization of latrines or toilets	83.3	83.3
Panama	PAN	Improved water source	96.4	96.4
Panama	PAN	Water connection in the home	89.6	89.6
Panama	PAN	Hygienic disposal of children's stools	42.6	42.6
Panama	PAN	Injectable antibiotics for neonatal sepsis	91.2	43.9
Panama	PAN	ORS - oral rehydration solution	52.4	26.5
Panama	PAN	Oral antibiotics for pneumonia	81.6	41.3
Panama	PAN	SAM - treatment for severe acute malnutrition	4.5	2.3
Panama	PAN	BCG vaccine	99.0	47.6
Panama	PAN	Polio vaccine	88.0	42.3
Panama	PAN	DPT vaccine	88.0	42.3
Panama	PAN	H. influenzae type b vaccine	88.0	73.4
Panama	PAN	HepB vaccine	88.0	42.3
Panama	PAN	Pneumococcal vaccine	92.0	76.7
Panama	PAN	Rotavirus vaccine	95.0	79.2
Panama	PAN	Measles vaccine	98.0	47.1
Panama	PAN	Global wasting (<-2 SD) rate	2.4	3.6
Panama	PAN	Contraceptive prevalence (CPR)	64	38.9
Papua New Guinea	PNG	Safe abortion services	85.0	51.6
Papua New Guinea	PNG	TT - Tetanus toxoid vaccination	70.0	33.7
Papua New Guinea	PNG	Syphilis detection and treatment	17.9	8.6
Papua New Guinea	PNG	Hypertensive disorder case management	11.8	5.7
Papua New Guinea	PNG	Diabetes case management	9.2	4.4
Papua New Guinea	PNG	Malaria case management	38.0	18.3
Papua New Guinea	PNG	MgSO4 management of pre-eclampsia	23.3	11.2
Papua New Guinea	PNG	Thermal protection	54.1	27.4
Papua New Guinea	PNG	Clean cord care	52.2	26.4
Papua New Guinea	PNG	Clean birth environment	44.9	22.7
Papua New Guinea	PNG	Immediate drying and additional stimulation	50.1	25.4
Papua New Guinea	PNG	Neonatal resuscitation	30.1	15.2
Papua New Guinea	PNG	Antibiotics for preterm or prolonged PROM	40.9	20.7
Papua New Guinea	PNG	Parenteral administration of anti-convulsants	39.1	19.8
Papua New Guinea	PNG	Parenteral administration of uterotonics	48.9	24.8
Papua New Guinea	PNG	Parenteral administration of antibiotics	40.9	20.7
Papua New Guinea	PNG	Assisted vaginal delivery	13.8	7.0
Papua New Guinea	PNG	Manual removal of placenta	20.5	10.4
Papua New Guinea	PNG	Removal of retained products of conception	18.1	9.2
Papua New Guinea	PNG	Cesarean delivery	4.8	2.4

Country	ISO 3166-1 alpha-3	Intervention Name	Baseline coverage (%)	Ending coverage (%)
Papua New Guinea	PNG	Blood transfusion	6.9	3.5
Papua New Guinea	PNG	Induction of labor for pregnancies lasting 41+ weeks	1.0	0.5
Papua New Guinea	PNG	Complementary feeding - education only	57.1	33.0
Papua New Guinea	PNG	Complementary feeding - supplementary feeding and education	57.1	33.0
Papua New Guinea	PNG	Vitamin A supplementation	15.0	8.7
Papua New Guinea	PNG	Improved sanitation - Utilization of latrines or toilets	12.9	12.9
Papua New Guinea	PNG	Improved water source	41.3	41.3
Papua New Guinea	PNG	Water connection in the home	7.6	7.6
Papua New Guinea	PNG	Hygienic disposal of children's stools	33.2	33.2
Papua New Guinea	PNG	ITN/IRS - Households protected from malaria	32.9	19.0
Papua New Guinea	PNG	Injectable antibiotics for neonatal sepsis	54.7	26.3
Papua New Guinea	PNG	ORS - oral rehydration solution	30.0	15.2
Papua New Guinea	PNG	Zinc for treatment of diarrhea	7.3	3.7
Papua New Guinea	PNG	Oral antibiotics for pneumonia	63.0	31.9
Papua New Guinea	PNG	Vitamin A for treatment of measles	15.0	7.6
Papua New Guinea	PNG	SAM - treatment for severe acute malnutrition	1.7	0.9
Papua New Guinea	PNG	BCG vaccine	69.0	33.2
Papua New Guinea	PNG	Polio vaccine	67.0	32.2
Papua New Guinea	PNG	DPT vaccine	61.0	29.3
Papua New Guinea	PNG	H. influenzae type b vaccine	61.0	50.9
Papua New Guinea	PNG	HepB vaccine	61.0	29.3
Papua New Guinea	PNG	Pneumococcal vaccine	43.0	35.9
Papua New Guinea	PNG	Measles vaccine	62.0	29.8
Papua New Guinea	PNG	Global wasting (<-2 SD) rate	10.6	15.9
Papua New Guinea	PNG	Contraceptive prevalence (CPR)	38.05	23.1
Paraguay	PRY	Safe abortion services	0.2	0.1
Paraguay	PRY	TT - Tetanus toxoid vaccination	95.0	45.7
Paraguay	PRY	Syphilis detection and treatment	24.4	11.7
Paraguay	PRY	Hypertensive disorder case management	22.5	10.8
Paraguay	PRY	Diabetes case management	17.5	8.4
Paraguay	PRY	Malaria case management	72.5	34.9
Paraguay	PRY	MgSO4 management of pre-eclampsia	44.5	21.4
Paraguay	PRY	Thermal protection	92.1	46.6
Paraguay	PRY	Clean cord care	88.9	45.0
Paraguay	PRY	Clean birth environment	76.4	38.7
Paraguay	PRY	Immediate drying and additional stimulation	85.3	43.2
Paraguay	PRY	Neonatal resuscitation	51.3	26.0
Paraguay	PRY	Antibiotics for preterm or prolonged PROM	69.8	35.3
Paraguay	PRY	Parenteral administration of anti-convulsants	66.7	33.8
Paraguay	PRY	Parenteral administration of uterotonics	83.3	42.2

Country	ISO 3166-1 alpha-3	Intervention Name	Baseline coverage (%)	Ending coverage (%)
Paraguay	PRY	Parenteral administration of antibiotics	69.8	35.3
Paraguay	PRY	Assisted vaginal delivery	23.6	11.9
Paraguay	PRY	Manual removal of placenta	34.9	17.7
Paraguay	PRY	Removal of retained products of conception	30.9	15.6
Paraguay	PRY	Cesarean delivery	8.1	4.1
Paraguay	PRY	Blood transfusion	11.7	5.9
Paraguay	PRY	Induction of labor for pregnancies lasting 41+ weeks	1.6	0.8
Paraguay	PRY	Complementary feeding - education only	70.4	40.6
Paraguay	PRY	Complementary feeding - supplementary feeding and education	70.4	40.6
Paraguay	PRY	Improved sanitation - Utilization of latrines or toilets	89.8	89.8
Paraguay	PRY	Improved water source	99.0	99.0
Paraguay	PRY	Water connection in the home	92.7	92.7
Paraguay	PRY	Hygienic disposal of children's stools	22.2	22.2
Paraguay	PRY	Injectable antibiotics for neonatal sepsis	93.2	44.8
Paraguay	PRY	ORS - oral rehydration solution	28.2	14.3
Paraguay	PRY	Zinc for treatment of diarrhea	7.1	3.6
Paraguay	PRY	Oral antibiotics for pneumonia	89.4	45.3
Paraguay	PRY	BCG vaccine	91.0	43.8
Paraguay	PRY	Polio vaccine	88.0	42.3
Paraguay	PRY	DPT vaccine	88.0	42.3
Paraguay	PRY	H. influenzae type b vaccine	88.0	73.4
Paraguay	PRY	HepB vaccine	88.0	42.3
Paraguay	PRY	Pneumococcal vaccine	94.0	78.4
Paraguay	PRY	Rotavirus vaccine	91.0	75.9
Paraguay	PRY	Measles vaccine	92.0	44.2
Paraguay	PRY	Global wasting (<-2 SD) rate	1.0	1.5
Paraguay	PRY	Contraceptive prevalence (CPR)	69.85	42.4
Peru	PER	Safe abortion services	0.2	0.1
Peru	PER	TT - Tetanus toxoid vaccination	95.0	45.7
Peru	PER	Syphilis detection and treatment	24.1	11.6
Peru	PER	Iron supplementation in pregnancy	59.7	28.7
Peru	PER	Hypertensive disorder case management	23.0	11.1
Peru	PER	Diabetes case management	17.9	8.6
Peru	PER	Malaria case management	74.2	35.7
Peru	PER	MgSO4 management of pre-eclampsia	45.5	21.9
Peru	PER	Thermal protection	89.7	45.4
Peru	PER	Clean cord care	86.6	43.8
Peru	PER	Clean birth environment	74.4	37.7
Peru	PER	Immediate drying and additional stimulation	83.1	42.1
Peru	PER	Neonatal resuscitation	49.9	25.3

Country	ISO 3166-1 alpha-3	Intervention Name	Baseline coverage (%)	Ending coverage (%)
Peru	PER	Antibiotics for preterm or prolonged PROM	67.9	34.4
Peru	PER	Parenteral administration of anti-convulsants	64.9	32.9
Peru	PER	Parenteral administration of uterotonics	81.1	41.1
Peru	PER	Parenteral administration of antibiotics	67.9	34.4
Peru	PER	Assisted vaginal delivery	22.9	11.6
Peru	PER	Manual removal of placenta	33.9	17.2
Peru	PER	Removal of retained products of conception	30.1	15.2
Peru	PER	Cesarean delivery	7.9	4.0
Peru	PER	Blood transfusion	11.4	5.8
Peru	PER	Induction of labor for pregnancies lasting 41+ weeks	1.6	0.8
Peru	PER	Complementary feeding - education only	82.6	47.7
Peru	PER	Complementary feeding - supplementary feeding and education	82.6	47.7
Peru	PER	Vitamin A supplementation	4.5	2.6
Peru	PER	Improved sanitation - Utilization of latrines or toilets	74.3	74.3
Peru	PER	Improved water source	91.1	91.1
Peru	PER	Water connection in the home	79.4	79.4
Peru	PER	Hand washing with soap	14.0	14.0
Peru	PER	Hygienic disposal of children's stools	12.8	12.8
Peru	PER	Injectable antibiotics for neonatal sepsis	90.7	43.6
Peru	PER	ORS - oral rehydration solution	34.6	17.5
Peru	PER	Antibiotics for treatment of dysentery	18.9	9.6
Peru	PER	Zinc for treatment of diarrhea	0.9	0.5
Peru	PER	Oral antibiotics for pneumonia	67.5	34.2
Peru	PER	Vitamin A for treatment of measles	4.5	2.3
Peru	PER	BCG vaccine	81.0	39.0
Peru	PER	Polio vaccine	83.0	39.9
Peru	PER	DPT vaccine	84.0	40.4
Peru	PER	H. influenzae type b vaccine	84.0	70.0
Peru	PER	HepB vaccine	84.0	40.4
Peru	PER	Pneumococcal vaccine	82.0	68.4
Peru	PER	Rotavirus vaccine	85.0	70.9
Peru	PER	Measles vaccine	83.0	39.9
Peru	PER	Global wasting (<-2 SD) rate	0.7	1.0
Peru	PER	Contraceptive prevalence (CPR)	75.5	45.9
Philippines	PHL	Safe abortion services	39.0	23.7
Philippines	PHL	TT - Tetanus toxoid vaccination	90.0	43.3
Philippines	PHL	Syphilis detection and treatment	23.1	11.1
Philippines	PHL	Iron supplementation in pregnancy	50.6	24.3
Philippines	PHL	Hypertensive disorder case management	20.7	10.0
Philippines	PHL	Diabetes case management	16.2	7.8

Country	ISO 3166-1 alpha-3	Intervention Name	Baseline coverage (%)	Ending coverage (%)
Philippines	PHL	Malaria case management	66.8	32.1
Philippines	PHL	MgSO4 management of pre-eclampsia	41.0	19.7
Philippines	PHL	Thermal protection	76.8	38.9
Philippines	PHL	Clean cord care	74.2	37.6
Philippines	PHL	Clean birth environment	63.8	32.3
Philippines	PHL	Immediate drying and additional stimulation	71.2	36.0
Philippines	PHL	Neonatal resuscitation	42.8	21.7
Philippines	PHL	Antibiotics for preterm or prolonged PROM	58.2	29.5
Philippines	PHL	Parenteral administration of anti-convulsants	55.6	28.1
Philippines	PHL	Parenteral administration of uterotonics	69.5	35.2
Philippines	PHL	Parenteral administration of antibiotics	58.2	29.5
Philippines	PHL	Assisted vaginal delivery	19.7	10.0
Philippines	PHL	Manual removal of placenta	29.1	14.7
Philippines	PHL	Removal of retained products of conception	25.8	13.1
Philippines	PHL	Cesarean delivery	6.8	3.4
Philippines	PHL	Blood transfusion	9.8	5.0
Philippines	PHL	Induction of labor for pregnancies lasting 41+ weeks	1.4	0.7
Philippines	PHL	Complementary feeding - education only	64.8	37.4
Philippines	PHL	Complementary feeding - supplementary feeding and education	64.8	37.4
Philippines	PHL	Vitamin A supplementation	68.0	39.2
Philippines	PHL	Improved sanitation - Utilization of latrines or toilets	76.5	76.5
Philippines	PHL	Improved water source	93.6	93.6
Philippines	PHL	Water connection in the home	37.5	37.5
Philippines	PHL	Hand washing with soap	86.6	86.6
Philippines	PHL	Hygienic disposal of children's stools	10.2	10.2
Philippines	PHL	Injectable antibiotics for neonatal sepsis	77.7	37.4
Philippines	PHL	ORS - oral rehydration solution	44.8	22.7
Philippines	PHL	Antibiotics for treatment of dysentery	38.7	19.6
Philippines	PHL	Zinc for treatment of diarrhea	24.4	12.4
Philippines	PHL	Oral antibiotics for pneumonia	66.6	33.7
Philippines	PHL	Vitamin A for treatment of measles	68.0	34.4
Philippines	PHL	SAM - treatment for severe acute malnutrition	0.1	0.1
Philippines	PHL	BCG vaccine	75.0	36.1
Philippines	PHL	Polio vaccine	66.0	31.7
Philippines	PHL	DPT vaccine	65.0	31.3
Philippines	PHL	H. influenzae type b vaccine	65.0	54.2
Philippines	PHL	HepB vaccine	65.0	31.3
Philippines	PHL	Pneumococcal vaccine	43.0	35.9
Philippines	PHL	Rotavirus vaccine	2.0	1.7
Philippines	PHL	Measles vaccine	89.0	42.8

Country	ISO 3166-1 alpha-3	Intervention Name	Baseline coverage (%)	Ending coverage (%)
Philippines	PHL	Global wasting (<-2 SD) rate	6.9	10.3
Philippines	PHL	Contraceptive prevalence (CPR)	56	34.0
Republic of Moldova	MDA	Safe abortion services	87.5	53.2
Republic of Moldova	MDA	Syphilis detection and treatment	24.4	11.7
Republic of Moldova	MDA	Iron supplementation in pregnancy	40.9	19.7
Republic of Moldova	MDA	Hypertensive disorder case management	22.9	11.0
Republic of Moldova	MDA	Diabetes case management	17.9	8.6
Republic of Moldova	MDA	Malaria case management	73.9	35.5
Republic of Moldova	MDA	MgSO4 management of pre-eclampsia	45.3	21.8
Republic of Moldova	MDA	Thermal protection	97.7	49.5
Republic of Moldova	MDA	Clean cord care	94.3	47.7
Republic of Moldova	MDA	Clean birth environment	81.1	41.1
Republic of Moldova	MDA	Immediate drying and additional stimulation	90.5	45.8
Republic of Moldova	MDA	Neonatal resuscitation	54.4	27.5
Republic of Moldova	MDA	Antibiotics for preterm or prolonged PROM	74.0	37.5
Republic of Moldova	MDA	Parenteral administration of anti-convulsants	70.7	35.8
Republic of Moldova	MDA	Parenteral administration of uterotonics	88.3	44.7
Republic of Moldova	MDA	Parenteral administration of antibiotics	74.0	37.5
Republic of Moldova	MDA	Assisted vaginal delivery	25.0	12.7
Republic of Moldova	MDA	Manual removal of placenta	37.0	18.7
Republic of Moldova	MDA	Removal of retained products of conception	32.8	16.6
Republic of Moldova	MDA	Cesarean delivery	8.6	4.4
Republic of Moldova	MDA	Blood transfusion	12.4	6.3
Republic of Moldova	MDA	Induction of labor for pregnancies lasting 41+ weeks	1.7	0.9
Republic of Moldova	MDA	Complementary feeding - education only	82.0	47.3
Republic of Moldova	MDA	Complementary feeding - supplementary feeding and education	82.0	47.3
Republic of Moldova	MDA	Improved sanitation - Utilization of latrines or toilets	76.3	76.3
Republic of Moldova	MDA	Improved water source	89.1	89.1
Republic of Moldova	MDA	Water connection in the home	58.9	58.9
Republic of Moldova	MDA	Hand washing with soap	89.4	89.4
Republic of Moldova	MDA	Hygienic disposal of children's stools	45.9	45.9
Republic of Moldova	MDA	Injectable antibiotics for neonatal sepsis	98.9	47.6
Republic of Moldova	MDA	ORS - oral rehydration solution	41.5	21.0
Republic of Moldova	MDA	Antibiotics for treatment of dysentery	24.0	12.2
Republic of Moldova	MDA	Oral antibiotics for pneumonia	79.2	40.1
Republic of Moldova	MDA	BCG vaccine	96.0	46.2
Republic of Moldova	MDA	Polio vaccine	94.0	45.2
Republic of Moldova	MDA	DPT vaccine	93.0	44.7
Republic of Moldova	MDA	H. influenzae type b vaccine	92.0	76.7
Republic of Moldova	MDA	HepB vaccine	94.0	45.2

Country	ISO 3166-1 alpha-3	Intervention Name	Baseline coverage (%)	Ending coverage (%)
Republic of Moldova	MDA	Pneumococcal vaccine	94.0	78.4
Republic of Moldova	MDA	Rotavirus vaccine	75.0	62.5
Republic of Moldova	MDA	Measles vaccine	93.0	44.7
Republic of Moldova	MDA	Global wasting (<-2 SD) rate	1.9	2.8
Republic of Moldova	MDA	Contraceptive prevalence (CPR)	62.9	38.2
Republic of North Macedonia	MKD	Safe abortion services	100.0	60.8
Republic of North Macedonia	MKD	Syphilis detection and treatment	24.4	11.7
Republic of North Macedonia	MKD	Hypertensive disorder case management	22.6	10.9
Republic of North Macedonia	MKD	Diabetes case management	17.6	8.5
Republic of North Macedonia	MKD	Malaria case management	72.8	35.0
Republic of North Macedonia	MKD	MgSO4 management of pre-eclampsia	44.6	21.4
Republic of North Macedonia	MKD	Thermal protection	97.1	49.2
Republic of North Macedonia	MKD	Clean cord care	93.8	47.5
Republic of North Macedonia	MKD	Clean birth environment	80.6	40.8
Republic of North Macedonia	MKD	Immediate drying and additional stimulation	90.0	45.6
Republic of North Macedonia	MKD	Neonatal resuscitation	54.0	27.3
Republic of North Macedonia	MKD	Antibiotics for preterm or prolonged PROM	73.5	37.2
Republic of North Macedonia	MKD	Parenteral administration of anti-convulsants	70.3	35.6
Republic of North Macedonia	MKD	Parenteral administration of uterotonics	87.8	44.4
Republic of North Macedonia	MKD	Parenteral administration of antibiotics	73.5	37.2
Republic of North Macedonia	MKD	Assisted vaginal delivery	24.8	12.6
Republic of North Macedonia	MKD	Manual removal of placenta	36.8	18.6
Republic of North Macedonia	MKD	Removal of retained products of conception	32.6	16.5
Republic of North Macedonia	MKD	Cesarean delivery	8.6	4.4
Republic of North Macedonia	MKD	Blood transfusion	12.4	6.3
Republic of North Macedonia	MKD	Induction of labor for pregnancies lasting 41+ weeks	1.7	0.9
Republic of North Macedonia	MKD	Improved sanitation - Utilization of latrines or toilets	99.0	99.0
Republic of North Macedonia	MKD	Improved water source	93.1	93.1

Country	ISO 3166-1 alpha-3	Intervention Name	Baseline coverage (%)	Ending coverage (%)
Republic of North Macedonia	MKD	Water connection in the home	86.2	86.2
Republic of North Macedonia	MKD	Hygienic disposal of children's stools	17.3	17.3
Republic of North Macedonia	MKD	Injectable antibiotics for neonatal sepsis	98.3	47.3
Republic of North Macedonia	MKD	ORS - oral rehydration solution	62.0	31.4
Republic of North Macedonia	MKD	Oral antibiotics for pneumonia	77.4	39.2
Republic of North Macedonia	MKD	BCG vaccine	97.0	46.7
Republic of North Macedonia	MKD	Polio vaccine	91.0	43.8
Republic of North Macedonia	MKD	DPT vaccine	91.0	43.8
Republic of North Macedonia	MKD	H. influenzae type b vaccine	91.0	75.9
Republic of North Macedonia	MKD	HepB vaccine	91.0	43.8
Republic of North Macedonia	MKD	Measles vaccine	83.0	39.9
Republic of North Macedonia	MKD	Global wasting (<-2 SD) rate	1.8	2.7
Republic of North Macedonia	MKD	Contraceptive prevalence (CPR)	46.35	28.2
Rwanda	RWA	Safe abortion services	3.3	2.0
Rwanda	RWA	TT - Tetanus toxoid vaccination	90.0	43.3
Rwanda	RWA	IPTp - Intermittent preventive treatment of malaria during pregnancy	17.7	8.5
Rwanda	RWA	Syphilis detection and treatment	52.0	25.0
Rwanda	RWA	Iron supplementation in pregnancy	3.4	1.6
Rwanda	RWA	Hypertensive disorder case management	1.9	0.9
Rwanda	RWA	Diabetes case management	8.3	4.0
Rwanda	RWA	Malaria case management	34.4	16.5
Rwanda	RWA	MgSO4 management of pre-eclampsia	7.7	3.7
Rwanda	RWA	Thermal protection	89.6	45.4
Rwanda	RWA	Clean cord care	81.4	41.2
Rwanda	RWA	Clean birth environment	78.6	39.8
Rwanda	RWA	Immediate drying and additional stimulation	73.4	37.2
Rwanda	RWA	Neonatal resuscitation	36.2	18.3
Rwanda	RWA	Antibiotics for preterm or prolonged PROM	36.7	18.6
Rwanda	RWA	Parenteral administration of anti-convulsants	60.1	30.4
Rwanda	RWA	Parenteral administration of uterotonics	33.5	17.0
Rwanda	RWA	Parenteral administration of antibiotics	36.7	18.6
Rwanda	RWA	Assisted vaginal delivery	20.3	10.3
Rwanda	RWA	Manual removal of placenta	25.7	13.0

Country	ISO 3166-1 alpha-3	Intervention Name	Baseline coverage (%)	Ending coverage (%)
Rwanda	RWA	Removal of retained products of conception	30.0	15.2
Rwanda	RWA	Cesarean delivery	12.1	6.1
Rwanda	RWA	Blood transfusion	6.0	3.0
Rwanda	RWA	Induction of labor for pregnancies lasting 41+ weeks	1.6	0.8
Rwanda	RWA	Complementary feeding - education only	30.1	17.4
Rwanda	RWA	Complementary feeding - supplementary feeding and education	30.1	17.4
Rwanda	RWA	Vitamin A supplementation	98.0	56.6
Rwanda	RWA	Improved sanitation - Utilization of latrines or toilets	66.6	66.6
Rwanda	RWA	Improved water source	57.7	57.7
Rwanda	RWA	Water connection in the home	21.5	21.5
Rwanda	RWA	Hand washing with soap	37.6	37.6
Rwanda	RWA	Hygienic disposal of children's stools	77.9	77.9
Rwanda	RWA	ITN/IRS - Households protected from malaria	84.1	48.5
Rwanda	RWA	Injectable antibiotics for neonatal sepsis	90.7	43.6
Rwanda	RWA	ORS - oral rehydration solution	27.5	13.9
Rwanda	RWA	Antibiotics for treatment of dysentery	15.1	7.6
Rwanda	RWA	Zinc for treatment of diarrhea	0.2	0.1
Rwanda	RWA	Oral antibiotics for pneumonia	53.9	27.3
Rwanda	RWA	Vitamin A for treatment of measles	98.0	49.6
Rwanda	RWA	ACTs- Artemisinin compounds for treatment of malaria	7.4	3.7
Rwanda	RWA	BCG vaccine	97.0	46.7
Rwanda	RWA	Polio vaccine	97.0	46.7
Rwanda	RWA	DPT vaccine	89.0	42.8
Rwanda	RWA	H. influenzae type b vaccine	97.0	80.9
Rwanda	RWA	HepB vaccine	97.0	46.7
Rwanda	RWA	Pneumococcal vaccine	97.0	80.9
Rwanda	RWA	Rotavirus vaccine	98.0	81.7
Rwanda	RWA	Measles vaccine	95.0	45.7
Rwanda	RWA	Global wasting (<-2 SD) rate	2.3	3.5
Rwanda	RWA	Contraceptive prevalence (CPR)	57.35	34.8
Saint Lucia	LCA	Safe abortion services	53.7	32.6
Saint Lucia	LCA	Syphilis detection and treatment	24.0	11.5
Saint Lucia	LCA	Hypertensive disorder case management	21.7	10.4
Saint Lucia	LCA	Diabetes case management	16.9	8.1
Saint Lucia	LCA	Malaria case management	70.0	33.7
Saint Lucia	LCA	MgSO4 management of pre-eclampsia	42.9	20.6
Saint Lucia	LCA	Thermal protection	97.6	49.4
Saint Lucia	LCA	Clean cord care	94.2	47.7
Saint Lucia	LCA	Clean birth environment	81.0	41.0
Saint Lucia	LCA	Immediate drying and additional stimulation	90.4	45.8

Country	ISO 3166-1 alpha-3	Intervention Name	Baseline coverage (%)	Ending coverage (%)
Saint Lucia	LCA	Neonatal resuscitation	54.3	27.5
Saint Lucia	LCA	Antibiotics for preterm or prolonged PROM	73.9	37.4
Saint Lucia	LCA	Parenteral administration of anti-convulsants	70.6	35.7
Saint Lucia	LCA	Parenteral administration of uterotonics	88.2	44.7
Saint Lucia	LCA	Parenteral administration of antibiotics	73.9	37.4
Saint Lucia	LCA	Assisted vaginal delivery	25.0	12.7
Saint Lucia	LCA	Manual removal of placenta	36.9	18.7
Saint Lucia	LCA	Removal of retained products of conception	32.7	16.6
Saint Lucia	LCA	Cesarean delivery	8.6	4.4
Saint Lucia	LCA	Blood transfusion	12.4	6.3
Saint Lucia	LCA	Induction of labor for pregnancies lasting 41+ weeks	1.7	0.9
Saint Lucia	LCA	Improved sanitation - Utilization of latrines or toilets	88.4	88.4
Saint Lucia	LCA	Improved water source	98.2	98.2
Saint Lucia	LCA	Water connection in the home	94.2	94.2
Saint Lucia	LCA	Hand washing with soap	89.0	89.0
Saint Lucia	LCA	Hygienic disposal of children's stools	26.6	26.6
Saint Lucia	LCA	Injectable antibiotics for neonatal sepsis	98.7	47.5
Saint Lucia	LCA	Oral antibiotics for pneumonia	27.0	13.7
Saint Lucia	LCA	BCG vaccine	99.0	47.6
Saint Lucia	LCA	Polio vaccine	95.0	45.7
Saint Lucia	LCA	DPT vaccine	95.0	45.7
Saint Lucia	LCA	H. influenzae type b vaccine	95.0	79.2
Saint Lucia	LCA	HepB vaccine	95.0	45.7
Saint Lucia	LCA	Measles vaccine	87.0	41.8
Saint Lucia	LCA	Global wasting (<-2 SD) rate	2.4	3.6
Saint Lucia	LCA	Contraceptive prevalence (CPR)	59.4	36.1
Samoa	WSM	Safe abortion services	85.0	51.6
Samoa	WSM	Syphilis detection and treatment	14.4	6.9
Samoa	WSM	Iron supplementation in pregnancy	3.4	1.6
Samoa	WSM	Hypertensive disorder case management	14.0	6.7
Samoa	WSM	Diabetes case management	10.9	5.2
Samoa	WSM	Malaria case management	45.3	21.8
Samoa	WSM	MgSO4 management of pre-eclampsia	27.8	13.4
Samoa	WSM	Thermal protection	79.6	40.3
Samoa	WSM	Clean cord care	76.8	38.9
Samoa	WSM	Clean birth environment	66.0	33.4
Samoa	WSM	Immediate drying and additional stimulation	73.7	37.3
Samoa	WSM	Neonatal resuscitation	44.3	22.4
Samoa	WSM	Antibiotics for preterm or prolonged PROM	60.2	30.5
Samoa	WSM	Parenteral administration of anti-convulsants	57.6	29.2
Samoa	WSM	Parenteral administration of uterotonics	71.9	36.4

Country	ISO 3166-1 alpha-3	Intervention Name	Baseline coverage (%)	Ending coverage (%)
Samoa	WSM	Parenteral administration of antibiotics	60.2	30.5
Samoa	WSM	Assisted vaginal delivery	20.3	10.3
Samoa	WSM	Manual removal of placenta	30.1	15.2
Samoa	WSM	Removal of retained products of conception	26.7	13.5
Samoa	WSM	Cesarean delivery	7.0	3.5
Samoa	WSM	Blood transfusion	10.1	5.1
Samoa	WSM	Induction of labor for pregnancies lasting 41+ weeks	1.4	0.7
Samoa	WSM	Improved sanitation - Utilization of latrines or toilets	98.2	98.2
Samoa	WSM	Improved water source	97.4	97.4
Samoa	WSM	Water connection in the home	82.7	82.7
Samoa	WSM	Hygienic disposal of children's stools	38.0	38.0
Samoa	WSM	Injectable antibiotics for neonatal sepsis	80.5	38.7
Samoa	WSM	ORS - oral rehydration solution	68.0	34.4
Samoa	WSM	Antibiotics for treatment of dysentery	1.0	0.5
Samoa	WSM	BCG vaccine	62.0	29.8
Samoa	WSM	Polio vaccine	31.0	14.9
Samoa	WSM	DPT vaccine	34.0	16.4
Samoa	WSM	H. influenzae type b vaccine	34.0	28.4
Samoa	WSM	HepB vaccine	34.0	16.4
Samoa	WSM	Measles vaccine	58.0	27.9
Samoa	WSM	Global wasting (<-2 SD) rate	10.6	15.9
Samoa	WSM	Contraceptive prevalence (CPR)	30.2	18.3
São Tomé and Príncipe	STP	TT - Tetanus toxoid vaccination	99.0	47.6
São Tomé and Príncipe	STP	IPTp - Intermittent preventive treatment of malaria during pregnancy	56.9	27.4
São Tomé and Príncipe	STP	Syphilis detection and treatment	24.1	11.6
São Tomé and Príncipe	STP	Iron supplementation in pregnancy	53.5	25.7
São Tomé and Príncipe	STP	Hypertensive disorder case management	20.1	9.7
São Tomé and Príncipe	STP	Diabetes case management	15.7	7.6
São Tomé and Príncipe	STP	Malaria case management	64.8	31.2
São Tomé and Príncipe	STP	MgSO4 management of pre-eclampsia	39.8	19.1
São Tomé and Príncipe	STP	Thermal protection	89.9	45.5
São Tomé and Príncipe	STP	Clean cord care	86.8	43.9
São Tomé and Príncipe	STP	Clean birth environment	74.6	37.8
São Tomé and Príncipe	STP	Immediate drying and additional stimulation	83.3	42.2
São Tomé and Príncipe	STP	Neonatal resuscitation	50.0	25.3
São Tomé and Príncipe	STP	Antibiotics for preterm or prolonged PROM	68.1	34.5
São Tomé and Príncipe	STP	Parenteral administration of anti-convulsants	65.1	33.0
São Tomé and Príncipe	STP	Parenteral administration of uterotonics	81.3	41.2
São Tomé and Príncipe	STP	Parenteral administration of antibiotics	68.1	34.5
São Tomé and Príncipe	STP	Assisted vaginal delivery	23.0	11.6

Country	ISO 3166-1 alpha-3	Intervention Name	Baseline coverage (%)	Ending coverage (%)
São Tomé and Príncipe	STP	Manual removal of placenta	34.0	17.2
São Tomé and Príncipe	STP	Removal of retained products of conception	30.1	15.2
São Tomé and Príncipe	STP	Cesarean delivery	7.9	4.0
São Tomé and Príncipe	STP	Blood transfusion	11.5	5.8
São Tomé and Príncipe	STP	Induction of labor for pregnancies lasting 41+ weeks	1.6	0.8
São Tomé and Príncipe	STP	Complementary feeding - education only	47.4	27.4
São Tomé and Príncipe	STP	Complementary feeding - supplementary feeding and education	47.4	27.4
São Tomé and Príncipe	STP	Vitamin A supplementation	23.0	13.3
São Tomé and Príncipe	STP	Improved sanitation - Utilization of latrines or toilets	43.0	43.0
São Tomé and Príncipe	STP	Improved water source	84.3	84.3
São Tomé and Príncipe	STP	Water connection in the home	79.0	79.0
São Tomé and Príncipe	STP	Hand washing with soap	40.6	40.6
São Tomé and Príncipe	STP	Hygienic disposal of children's stools	28.9	28.9
São Tomé and Príncipe	STP	ITN/IRS - Households protected from malaria	96.7	55.8
São Tomé and Príncipe	STP	Injectable antibiotics for neonatal sepsis	91.0	43.8
São Tomé and Príncipe	STP	ORS - oral rehydration solution	49.1	24.9
São Tomé and Príncipe	STP	Oral antibiotics for pneumonia	68.9	34.9
São Tomé and Príncipe	STP	Vitamin A for treatment of measles	23.0	11.6
São Tomé and Príncipe	STP	ACTs- Artemisinin compounds for treatment of malaria	0.1	0.1
São Tomé and Príncipe	STP	BCG vaccine	96.0	46.2
São Tomé and Príncipe	STP	Polio vaccine	95.0	45.7
São Tomé and Príncipe	STP	DPT vaccine	95.0	45.7
São Tomé and Príncipe	STP	H. influenzae type b vaccine	95.0	79.2
São Tomé and Príncipe	STP	HepB vaccine	95.0	45.7
São Tomé and Príncipe	STP	Pneumococcal vaccine	95.0	79.2
São Tomé and Príncipe	STP	Rotavirus vaccine	95.0	79.2
São Tomé and Príncipe	STP	Measles vaccine	90.0	43.3
São Tomé and Príncipe	STP	Global wasting (<-2 SD) rate	4.2	6.3
São Tomé and Príncipe	STP	Contraceptive prevalence (CPR)	45.3	27.5
Senegal	SEN	TT - Tetanus toxoid vaccination	95.0	45.7
Senegal	SEN	IPTp - Intermittent preventive treatment of malaria during pregnancy	62.6	30.1
Senegal	SEN	Syphilis detection and treatment	5.9	2.8
Senegal	SEN	Iron supplementation in pregnancy	63.1	30.3
Senegal	SEN	Hypertensive disorder case management	0.3	0.1
Senegal	SEN	Diabetes case management	33.5	16.1
Senegal	SEN	Malaria case management	47.7	22.9
Senegal	SEN	MgSO4 management of pre-eclampsia	36.5	17.6
Senegal	SEN	Thermal protection	69.1	35.0
Senegal	SEN	Clean cord care	68.7	34.8
Senegal	SEN	Clean birth environment	68.5	34.7

Country	ISO 3166-1 alpha-3	Intervention Name	Baseline coverage (%)	Ending coverage (%)
Senegal	SEN	Immediate drying and additional stimulation	68.9	34.9
Senegal	SEN	Neonatal resuscitation	50.5	25.6
Senegal	SEN	Antibiotics for preterm or prolonged PROM	52.3	26.5
Senegal	SEN	Parenteral administration of anti-convulsants	59.2	30.0
Senegal	SEN	Parenteral administration of uterotonics	61.1	30.9
Senegal	SEN	Parenteral administration of antibiotics	52.3	26.5
Senegal	SEN	Assisted vaginal delivery	7.4	3.7
Senegal	SEN	Manual removal of placenta	36.0	18.2
Senegal	SEN	Removal of retained products of conception	35.1	17.8
Senegal	SEN	Cesarean delivery	6.4	3.2
Senegal	SEN	Blood transfusion	7.6	3.8
Senegal	SEN	Induction of labor for pregnancies lasting 41+ weeks	8.1	4.1
Senegal	SEN	Complementary feeding - education only	25.3	14.6
Senegal	SEN	Complementary feeding - supplementary feeding and education	25.3	14.6
Senegal	SEN	Vitamin A supplementation	58.0	33.5
Senegal	SEN	Improved sanitation - Utilization of latrines or toilets	51.5	51.5
Senegal	SEN	Improved water source	80.7	80.7
Senegal	SEN	Water connection in the home	58.9	58.9
Senegal	SEN	Hand washing with soap	28.5	28.5
Senegal	SEN	Hygienic disposal of children's stools	57.0	57.0
Senegal	SEN	ITN/IRS - Households protected from malaria	84.5	48.8
Senegal	SEN	Injectable antibiotics for neonatal sepsis	69.9	33.6
Senegal	SEN	ORS - oral rehydration solution	27.9	14.1
Senegal	SEN	Antibiotics for treatment of dysentery	12.8	6.5
Senegal	SEN	Zinc for treatment of diarrhea	26.9	13.6
Senegal	SEN	Oral antibiotics for pneumonia	52.0	26.3
Senegal	SEN	Vitamin A for treatment of measles	58.0	29.4
Senegal	SEN	ACTs- Artemisinin compounds for treatment of malaria	1.5	0.8
Senegal	SEN	BCG vaccine	83.0	39.9
Senegal	SEN	Polio vaccine	81.0	39.0
Senegal	SEN	DPT vaccine	81.0	39.0
Senegal	SEN	H. influenzae type b vaccine	82.0	68.4
Senegal	SEN	HepB vaccine	82.0	39.4
Senegal	SEN	Pneumococcal vaccine	81.0	67.5
Senegal	SEN	Rotavirus vaccine	80.0	66.7
Senegal	SEN	Measles vaccine	90.0	43.3
Senegal	SEN	Global wasting (<-2 SD) rate	9.0	13.4
Senegal	SEN	Contraceptive prevalence (CPR)	29.95	18.2
Serbia	SRB	Safe abortion services	100.0	60.8
Serbia	SRB	Syphilis detection and treatment	6.0	2.9

Country	ISO 3166-1 alpha-3	Intervention Name	Baseline coverage (%)	Ending coverage (%)
Serbia	SRB	Hypertensive disorder case management	0.6	0.3
Serbia	SRB	Diabetes case management	56.3	27.1
Serbia	SRB	Malaria case management	80.1	38.5
Serbia	SRB	MgSO4 management of pre-eclampsia	61.2	29.4
Serbia	SRB	Thermal protection	97.1	49.2
Serbia	SRB	Clean cord care	93.8	47.5
Serbia	SRB	Clean birth environment	80.6	40.8
Serbia	SRB	Immediate drying and additional stimulation	90.0	45.6
Serbia	SRB	Neonatal resuscitation	54.0	27.3
Serbia	SRB	Antibiotics for preterm or prolonged PROM	73.5	37.2
Serbia	SRB	Parenteral administration of anti-convulsants	70.3	35.6
Serbia	SRB	Parenteral administration of uterotonics	87.8	44.4
Serbia	SRB	Parenteral administration of antibiotics	73.5	37.2
Serbia	SRB	Assisted vaginal delivery	24.8	12.6
Serbia	SRB	Manual removal of placenta	36.8	18.6
Serbia	SRB	Removal of retained products of conception	32.6	16.5
Serbia	SRB	Cesarean delivery	8.6	4.4
Serbia	SRB	Blood transfusion	12.4	6.3
Serbia	SRB	Induction of labor for pregnancies lasting 41+ weeks	1.7	0.9
Serbia	SRB	Complementary feeding - education only	89.6	51.7
Serbia	SRB	Complementary feeding - supplementary feeding and education	89.6	51.7
Serbia	SRB	Improved sanitation - Utilization of latrines or toilets	97.6	97.6
Serbia	SRB	Improved water source	85.5	85.5
Serbia	SRB	Water connection in the home	82.1	82.1
Serbia	SRB	Hand washing with soap	98.6	98.6
Serbia	SRB	Hygienic disposal of children's stools	25.7	25.7
Serbia	SRB	Injectable antibiotics for neonatal sepsis	98.3	47.3
Serbia	SRB	ORS - oral rehydration solution	36.0	18.2
Serbia	SRB	Oral antibiotics for pneumonia	89.7	45.4
Serbia	SRB	BCG vaccine	98.0	47.1
Serbia	SRB	Polio vaccine	96.0	46.2
Serbia	SRB	DPT vaccine	96.0	46.2
Serbia	SRB	H. influenzae type b vaccine	96.0	80.1
Serbia	SRB	HepB vaccine	91.0	43.8
Serbia	SRB	Pneumococcal vaccine	48.0	40.0
Serbia	SRB	Measles vaccine	86.0	41.4
Serbia	SRB	Global wasting (<-2 SD) rate	3.8	5.7
Serbia	SRB	Contraceptive prevalence (CPR)	59.25	36.0
Sierra Leone	SLE	TT - Tetanus toxoid vaccination	90.0	43.3
Sierra Leone	SLE	IPTp - Intermittent preventive treatment of malaria during pregnancy	66.6	32.0

Country	ISO 3166-1 alpha-3	Intervention Name	Baseline coverage (%)	Ending coverage (%)
Sierra Leone	SLE	Syphilis detection and treatment	19.9	9.6
Sierra Leone	SLE	Iron supplementation in pregnancy	30.0	14.4
Sierra Leone	SLE	Hypertensive disorder case management	40.8	19.6
Sierra Leone	SLE	Diabetes case management	11.4	5.5
Sierra Leone	SLE	Malaria case management	70.0	33.7
Sierra Leone	SLE	MgSO4 management of pre-eclampsia	67.1	32.3
Sierra Leone	SLE	Thermal protection	75.7	38.3
Sierra Leone	SLE	Clean cord care	69.8	35.3
Sierra Leone	SLE	Clean birth environment	65.3	33.1
Sierra Leone	SLE	Immediate drying and additional stimulation	71.3	36.1
Sierra Leone	SLE	Neonatal resuscitation	32.5	16.5
Sierra Leone	SLE	Antibiotics for preterm or prolonged PROM	66.2	33.5
Sierra Leone	SLE	Parenteral administration of anti-convulsants	67.6	34.2
Sierra Leone	SLE	Parenteral administration of uterotonics	69.5	35.2
Sierra Leone	SLE	Parenteral administration of antibiotics	66.2	33.5
Sierra Leone	SLE	Assisted vaginal delivery	43.5	22.0
Sierra Leone	SLE	Manual removal of placenta	16.2	8.2
Sierra Leone	SLE	Removal of retained products of conception	34.4	17.4
Sierra Leone	SLE	Cesarean delivery	1.7	0.9
Sierra Leone	SLE	Blood transfusion	11.7	5.9
Sierra Leone	SLE	Induction of labor for pregnancies lasting 41+ weeks	0.8	0.4
Sierra Leone	SLE	Complementary feeding - education only	24.2	14.0
Sierra Leone	SLE	Complementary feeding - supplementary feeding and education	24.2	14.0
Sierra Leone	SLE	Vitamin A supplementation	98.0	56.6
Sierra Leone	SLE	Improved sanitation - Utilization of latrines or toilets	15.7	15.7
Sierra Leone	SLE	Improved water source	60.8	60.8
Sierra Leone	SLE	Water connection in the home	14.9	14.9
Sierra Leone	SLE	Hand washing with soap	34.3	34.3
Sierra Leone	SLE	Hygienic disposal of children's stools	63.0	63.0
Sierra Leone	SLE	ITN/IRS - Households protected from malaria	72.0	41.6
Sierra Leone	SLE	Injectable antibiotics for neonatal sepsis	76.6	36.8
Sierra Leone	SLE	ORS - oral rehydration solution	77.7	39.3
Sierra Leone	SLE	Antibiotics for treatment of dysentery	50.5	25.6
Sierra Leone	SLE	Zinc for treatment of diarrhea	50.0	25.3
Sierra Leone	SLE	Oral antibiotics for pneumonia	73.3	37.1
Sierra Leone	SLE	Vitamin A for treatment of measles	98.0	49.6
Sierra Leone	SLE	ACTs- Artemisinin compounds for treatment of malaria	13.9	7.0
Sierra Leone	SLE	BCG vaccine	90.0	43.3
Sierra Leone	SLE	Polio vaccine	90.0	43.3
Sierra Leone	SLE	DPT vaccine	90.0	43.3

Country	ISO 3166-1 alpha-3	Intervention Name	Baseline coverage (%)	Ending coverage (%)
Sierra Leone	SLE	H. influenzae type b vaccine	90.0	75.1
Sierra Leone	SLE	HepB vaccine	90.0	43.3
Sierra Leone	SLE	Pneumococcal vaccine	90.0	75.1
Sierra Leone	SLE	Rotavirus vaccine	92.0	76.7
Sierra Leone	SLE	Measles vaccine	80.0	38.5
Sierra Leone	SLE	Global wasting (<-2 SD) rate	6.9	10.3
Sierra Leone	SLE	Contraceptive prevalence (CPR)	23.85	14.5
Solomon Islands	SLB	Safe abortion services	85.0	51.6
Solomon Islands	SLB	TT - Tetanus toxoid vaccination	85.0	40.9
Solomon Islands	SLB	IPTp - Intermittent preventive treatment of malaria during pregnancy	1.2	0.6
Solomon Islands	SLB	Syphilis detection and treatment	19.8	9.5
Solomon Islands	SLB	Hypertensive disorder case management	15.5	7.5
Solomon Islands	SLB	Diabetes case management	12.1	5.8
Solomon Islands	SLB	Malaria case management	50.1	24.1
Solomon Islands	SLB	MgSO4 management of pre-eclampsia	30.7	14.8
Solomon Islands	SLB	Thermal protection	83.5	42.3
Solomon Islands	SLB	Clean cord care	80.6	40.8
Solomon Islands	SLB	Clean birth environment	69.3	35.1
Solomon Islands	SLB	Immediate drying and additional stimulation	77.4	39.2
Solomon Islands	SLB	Neonatal resuscitation	46.5	23.5
Solomon Islands	SLB	Antibiotics for preterm or prolonged PROM	63.2	32.0
Solomon Islands	SLB	Parenteral administration of anti-convulsants	60.4	30.6
Solomon Islands	SLB	Parenteral administration of uterotonics	75.5	38.2
Solomon Islands	SLB	Parenteral administration of antibiotics	63.2	32.0
Solomon Islands	SLB	Assisted vaginal delivery	21.4	10.8
Solomon Islands	SLB	Manual removal of placenta	31.6	16.0
Solomon Islands	SLB	Removal of retained products of conception	28.0	14.2
Solomon Islands	SLB	Cesarean delivery	7.4	3.7
Solomon Islands	SLB	Blood transfusion	10.6	5.4
Solomon Islands	SLB	Induction of labor for pregnancies lasting 41+ weeks	1.5	0.8
Solomon Islands	SLB	Complementary feeding - education only	36.9	21.3
Solomon Islands	SLB	Complementary feeding - supplementary feeding and education	36.9	21.3
Solomon Islands	SLB	Vitamin A supplementation	7.4	4.3
Solomon Islands	SLB	Improved sanitation - Utilization of latrines or toilets	33.5	33.5
Solomon Islands	SLB	Improved water source	67.8	67.8
Solomon Islands	SLB	Water connection in the home	31.3	31.3
Solomon Islands	SLB	Hygienic disposal of children's stools	29.4	29.4
Solomon Islands	SLB	ITN/IRS - Households protected from malaria	48.5	28.0
Solomon Islands	SLB	Injectable antibiotics for neonatal sepsis	84.5	40.6
Solomon Islands	SLB	ORS - oral rehydration solution	37.7	19.1

Country	ISO 3166-1 alpha-3	Intervention Name	Baseline coverage (%)	Ending coverage (%)
Solomon Islands	SLB	Antibiotics for treatment of dysentery	3.9	2.0
Solomon Islands	SLB	Vitamin A for treatment of measles	7.4	3.7
Solomon Islands	SLB	BCG vaccine	83.0	39.9
Solomon Islands	SLB	Polio vaccine	85.0	40.9
Solomon Islands	SLB	DPT vaccine	85.0	40.9
Solomon Islands	SLB	H. influenzae type b vaccine	85.0	70.9
Solomon Islands	SLB	HepB vaccine	85.0	40.9
Solomon Islands	SLB	Pneumococcal vaccine	84.0	70.0
Solomon Islands	SLB	Measles vaccine	84.0	40.4
Solomon Islands	SLB	Global wasting (<-2 SD) rate	10.6	15.9
Solomon Islands	SLB	Contraceptive prevalence (CPR)	33.8	20.5
Somalia	SOM	Safe abortion services	3.3	2.0
Somalia	SOM	TT - Tetanus toxoid vaccination	67.0	32.2
Somalia	SOM	IPTp - Intermittent preventive treatment of malaria during pregnancy	0.9	0.4
Somalia	SOM	Syphilis detection and treatment	6.5	3.1
Somalia	SOM	Hypertensive disorder case management	0.5	0.2
Somalia	SOM	Diabetes case management	0.4	0.2
Somalia	SOM	Malaria case management	1.5	0.7
Somalia	SOM	MgSO4 management of pre-eclampsia	0.9	0.4
Somalia	SOM	Thermal protection	9.3	4.7
Somalia	SOM	Clean cord care	8.9	4.5
Somalia	SOM	Clean birth environment	7.7	3.9
Somalia	SOM	Immediate drying and additional stimulation	8.6	4.4
Somalia	SOM	Neonatal resuscitation	5.1	2.6
Somalia	SOM	Antibiotics for preterm or prolonged PROM	7.0	3.5
Somalia	SOM	Parenteral administration of anti-convulsants	6.7	3.4
Somalia	SOM	Parenteral administration of uterotonics	8.4	4.3
Somalia	SOM	Parenteral administration of antibiotics	7.0	3.5
Somalia	SOM	Assisted vaginal delivery	2.4	1.2
Somalia	SOM	Manual removal of placenta	3.5	1.8
Somalia	SOM	Removal of retained products of conception	3.1	1.6
Somalia	SOM	Cesarean delivery	0.8	0.4
Somalia	SOM	Blood transfusion	1.2	0.6
Somalia	SOM	Induction of labor for pregnancies lasting 41+ weeks	0.2	0.1
Somalia	SOM	Complementary feeding - education only	11.0	6.3
Somalia	SOM	Complementary feeding - supplementary feeding and education	11.0	6.3
Somalia	SOM	Vitamin A supplementation	11.0	6.3
Somalia	SOM	Improved sanitation - Utilization of latrines or toilets	38.3	38.3
Somalia	SOM	Improved water source	52.4	52.4
Somalia	SOM	Water connection in the home	21.7	21.7

Country	ISO 3166-1 alpha-3	Intervention Name	Baseline coverage (%)	Ending coverage (%)
Somalia	SOM	Hygienic disposal of children's stools	34.7	34.7
Somalia	SOM	ITN/IRS - Households protected from malaria	12.2	7.0
Somalia	SOM	Injectable antibiotics for neonatal sepsis	9.4	4.5
Somalia	SOM	ORS - oral rehydration solution	13.2	6.7
Somalia	SOM	Oral antibiotics for pneumonia	13.0	6.6
Somalia	SOM	Vitamin A for treatment of measles	11.0	5.6
Somalia	SOM	ACTs- Artemisinin compounds for treatment of malaria	0.2	0.1
Somalia	SOM	SAM - treatment for severe acute malnutrition	20.1	10.2
Somalia	SOM	BCG vaccine	37.0	17.8
Somalia	SOM	Polio vaccine	47.0	22.6
Somalia	SOM	DPT vaccine	42.0	20.2
Somalia	SOM	H. influenzae type b vaccine	42.0	35.0
Somalia	SOM	HepB vaccine	42.0	20.2
Somalia	SOM	Measles vaccine	46.0	22.1
Somalia	SOM	Global wasting (<-2 SD) rate	13.3	19.9
Somalia	SOM	Contraceptive prevalence (CPR)	26.75	16.3
South Africa	ZAF	Safe abortion services	42.5	25.8
South Africa	ZAF	TT - Tetanus toxoid vaccination	90.0	43.3
South Africa	ZAF	Syphilis detection and treatment	23.2	11.2
South Africa	ZAF	Iron supplementation in pregnancy	50.5	24.3
South Africa	ZAF	Hypertensive disorder case management	18.4	8.8
South Africa	ZAF	Diabetes case management	14.4	6.9
South Africa	ZAF	Malaria case management	59.4	28.6
South Africa	ZAF	MgSO4 management of pre-eclampsia	36.4	17.5
South Africa	ZAF	Thermal protection	94.8	48.0
South Africa	ZAF	Clean cord care	91.6	46.4
South Africa	ZAF	Clean birth environment	78.7	39.8
South Africa	ZAF	Immediate drying and additional stimulation	87.8	44.4
South Africa	ZAF	Neonatal resuscitation	52.8	26.7
South Africa	ZAF	Antibiotics for preterm or prolonged PROM	71.8	36.3
South Africa	ZAF	Parenteral administration of anti-convulsants	68.6	34.7
South Africa	ZAF	Parenteral administration of uterotonics	85.7	43.4
South Africa	ZAF	Parenteral administration of antibiotics	71.8	36.3
South Africa	ZAF	Assisted vaginal delivery	24.3	12.3
South Africa	ZAF	Manual removal of placenta	35.9	18.2
South Africa	ZAF	Removal of retained products of conception	31.8	16.1
South Africa	ZAF	Cesarean delivery	8.4	4.3
South Africa	ZAF	Blood transfusion	12.1	6.1
South Africa	ZAF	Induction of labor for pregnancies lasting 41+ weeks	1.7	0.9
South Africa	ZAF	Complementary feeding - education only	49.3	28.5

Country	ISO 3166-1 alpha-3	Intervention Name	Baseline coverage (%)	Ending coverage (%)
South Africa	ZAF	Complementary feeding - supplementary feeding and education	49.3	28.5
South Africa	ZAF	Vitamin A supplementation	47.0	27.1
South Africa	ZAF	Improved sanitation - Utilization of latrines or toilets	75.7	75.7
South Africa	ZAF	Improved water source	92.7	92.7
South Africa	ZAF	Water connection in the home	83.4	83.4
South Africa	ZAF	Injectable antibiotics for neonatal sepsis	95.9	46.1
South Africa	ZAF	ORS - oral rehydration solution	51.4	26.0
South Africa	ZAF	Antibiotics for treatment of dysentery	8.0	4.1
South Africa	ZAF	Zinc for treatment of diarrhea	36.8	18.6
South Africa	ZAF	Oral antibiotics for pneumonia	65.7	33.3
South Africa	ZAF	Vitamin A for treatment of measles	47.0	23.8
South Africa	ZAF	BCG vaccine	70.0	33.7
South Africa	ZAF	Polio vaccine	74.0	35.6
South Africa	ZAF	DPT vaccine	74.0	35.6
South Africa	ZAF	H. influenzae type b vaccine	74.0	61.7
South Africa	ZAF	HepB vaccine	74.0	35.6
South Africa	ZAF	Pneumococcal vaccine	73.0	60.9
South Africa	ZAF	Rotavirus vaccine	70.0	58.4
South Africa	ZAF	Measles vaccine	60.0	28.9
South Africa	ZAF	Global wasting (<-2 SD) rate	2.6	3.9
South Africa	ZAF	Contraceptive prevalence (CPR)	56.95	34.6
South Sudan	SSD	Safe abortion services	2.2	1.3
South Sudan	SSD	TT - Tetanus toxoid vaccination	68.0	32.7
South Sudan	SSD	IPTp - Intermittent preventive treatment of malaria during pregnancy	10.2	4.9
South Sudan	SSD	Syphilis detection and treatment	10.0	4.8
South Sudan	SSD	Hypertensive disorder case management	4.2	2.0
South Sudan	SSD	Diabetes case management	3.2	1.5
South Sudan	SSD	Malaria case management	13.4	6.4
South Sudan	SSD	MgSO4 management of pre-eclampsia	8.2	3.9
South Sudan	SSD	Thermal protection	11.5	5.8
South Sudan	SSD	Clean cord care	11.1	5.6
South Sudan	SSD	Clean birth environment	9.6	4.9
South Sudan	SSD	Immediate drying and additional stimulation	10.7	5.4
South Sudan	SSD	Neonatal resuscitation	6.4	3.2
South Sudan	SSD	Antibiotics for preterm or prolonged PROM	8.7	4.4
South Sudan	SSD	Parenteral administration of anti-convulsants	8.3	4.2
South Sudan	SSD	Parenteral administration of uterotonics	10.4	5.3
South Sudan	SSD	Parenteral administration of antibiotics	8.7	4.4
South Sudan	SSD	Assisted vaginal delivery	2.9	1.5
South Sudan	SSD	Manual removal of placenta	4.4	2.2

**Country**	**ISO 3166-1 alpha-3**	**Intervention Name**	**Baseline coverage (%)**	**Ending coverage (%)**
South Sudan	SSD	Removal of retained products of conception	3.9	2.0
South Sudan	SSD	Cesarean delivery	1.0	0.5
South Sudan	SSD	Blood transfusion	1.5	0.8
South Sudan	SSD	Induction of labor for pregnancies lasting 41+ weeks	0.2	0.1
South Sudan	SSD	Complementary feeding - education only	49.4	28.5
South Sudan	SSD	Complementary feeding - supplementary feeding and education	49.4	28.5
South Sudan	SSD	Vitamin A supplementation	51.0	29.4
South Sudan	SSD	Improved sanitation - Utilization of latrines or toilets	11.3	11.3
South Sudan	SSD	Improved water source	40.7	40.7
South Sudan	SSD	Water connection in the home	1.3	1.3
South Sudan	SSD	Hygienic disposal of children's stools	15.7	15.7
South Sudan	SSD	ITN/IRS - Households protected from malaria	38.9	22.5
South Sudan	SSD	Injectable antibiotics for neonatal sepsis	11.7	5.6
South Sudan	SSD	ORS - oral rehydration solution	38.6	19.5
South Sudan	SSD	Zinc for treatment of diarrhea	3.1	1.6
South Sudan	SSD	Oral antibiotics for pneumonia	47.6	24.1
South Sudan	SSD	Vitamin A for treatment of measles	51.0	25.8
South Sudan	SSD	ACTs- Artemisinin compounds for treatment of malaria	2.8	1.4
South Sudan	SSD	SAM - treatment for severe acute malnutrition	46.1	23.3
South Sudan	SSD	BCG vaccine	47.0	22.6
South Sudan	SSD	Polio vaccine	50.0	24.0
South Sudan	SSD	DPT vaccine	49.0	23.6
South Sudan	SSD	H. influenzae type b vaccine	49.0	40.9
South Sudan	SSD	HepB vaccine	49.0	23.6
South Sudan	SSD	Measles vaccine	20.0	9.6
South Sudan	SSD	Global wasting (<-2 SD) rate	22.3	33.4
South Sudan	SSD	Contraceptive prevalence (CPR)	6.7	4.1
Sri Lanka	LKA	Safe abortion services	35.1	21.3
Sri Lanka	LKA	TT - Tetanus toxoid vaccination	99.0	47.6
Sri Lanka	LKA	Syphilis detection and treatment	24.4	11.7
Sri Lanka	LKA	Hypertensive disorder case management	22.2	10.7
Sri Lanka	LKA	Diabetes case management	17.3	8.3
Sri Lanka	LKA	Malaria case management	71.7	34.5
Sri Lanka	LKA	MgSO4 management of pre-eclampsia	44.0	21.2
Sri Lanka	LKA	Thermal protection	98.3	49.8
Sri Lanka	LKA	Clean cord care	94.9	48.0
Sri Lanka	LKA	Clean birth environment	81.6	41.3
Sri Lanka	LKA	Immediate drying and additional stimulation	91.1	46.1
Sri Lanka	LKA	Neonatal resuscitation	54.7	27.7
Sri Lanka	LKA	Antibiotics for preterm or prolonged PROM	74.5	37.7

Country	ISO 3166-1 alpha-3	Intervention Name	Baseline coverage (%)	Ending coverage (%)
Sri Lanka	LKA	Parenteral administration of anti-convulsants	71.1	36.0
Sri Lanka	LKA	Parenteral administration of uterotonics	88.9	45.0
Sri Lanka	LKA	Parenteral administration of antibiotics	74.5	37.7
Sri Lanka	LKA	Assisted vaginal delivery	25.2	12.8
Sri Lanka	LKA	Manual removal of placenta	37.2	18.8
Sri Lanka	LKA	Removal of retained products of conception	33.0	16.7
Sri Lanka	LKA	Cesarean delivery	8.7	4.4
Sri Lanka	LKA	Blood transfusion	12.5	6.3
Sri Lanka	LKA	Induction of labor for pregnancies lasting 41+ weeks	1.8	0.9
Sri Lanka	LKA	Complementary feeding - education only	72.6	41.9
Sri Lanka	LKA	Complementary feeding - supplementary feeding and education	72.6	41.9
Sri Lanka	LKA	Vitamin A supplementation	93.0	53.7
Sri Lanka	LKA	Improved sanitation - Utilization of latrines or toilets	95.8	95.8
Sri Lanka	LKA	Improved water source	89.4	89.4
Sri Lanka	LKA	Water connection in the home	34.2	34.2
Sri Lanka	LKA	Hygienic disposal of children's stools	90.6	90.6
Sri Lanka	LKA	ITN/IRS - Households protected from malaria	6.4	3.7
Sri Lanka	LKA	Injectable antibiotics for neonatal sepsis	99.5	47.9
Sri Lanka	LKA	ORS - oral rehydration solution	54.0	27.3
Sri Lanka	LKA	Oral antibiotics for pneumonia	52.3	26.5
Sri Lanka	LKA	Vitamin A for treatment of measles	93.0	47.1
Sri Lanka	LKA	BCG vaccine	99.0	47.6
Sri Lanka	LKA	Polio vaccine	99.0	47.6
Sri Lanka	LKA	DPT vaccine	99.0	47.6
Sri Lanka	LKA	H. influenzae type b vaccine	99.0	82.6
Sri Lanka	LKA	HepB vaccine	99.0	47.6
Sri Lanka	LKA	Measles vaccine	99.0	47.6
Sri Lanka	LKA	Global wasting (<-2 SD) rate	10.6	15.9
Sri Lanka	LKA	Contraceptive prevalence (CPR)	67.55	41.0
Sudan	SDN	Safe abortion services	2.2	1.3
Sudan	SDN	TT - Tetanus toxoid vaccination	80.0	38.5
Sudan	SDN	IPTp - Intermittent preventive treatment of malaria during pregnancy	1.9	0.9
Sudan	SDN	Syphilis detection and treatment	19.6	9.4
Sudan	SDN	Hypertensive disorder case management	12.2	5.9
Sudan	SDN	Diabetes case management	9.5	4.6
Sudan	SDN	Malaria case management	39.3	18.9
Sudan	SDN	MgSO4 management of pre-eclampsia	24.1	11.6
Sudan	SDN	Thermal protection	27.4	13.9
Sudan	SDN	Clean cord care	26.5	13.4
Sudan	SDN	Clean birth environment	22.7	11.5

Country	ISO 3166-1 alpha-3	Intervention Name	Baseline coverage (%)	Ending coverage (%)
Sudan	SDN	Immediate drying and additional stimulation	25.4	12.9
Sudan	SDN	Neonatal resuscitation	15.2	7.7
Sudan	SDN	Antibiotics for preterm or prolonged PROM	20.7	10.5
Sudan	SDN	Parenteral administration of anti-convulsants	19.8	10.0
Sudan	SDN	Parenteral administration of uterotonics	24.8	12.6
Sudan	SDN	Parenteral administration of antibiotics	20.7	10.5
Sudan	SDN	Assisted vaginal delivery	7.0	3.5
Sudan	SDN	Manual removal of placenta	10.4	5.3
Sudan	SDN	Removal of retained products of conception	9.2	4.7
Sudan	SDN	Cesarean delivery	2.4	1.2
Sudan	SDN	Blood transfusion	3.5	1.8
Sudan	SDN	Induction of labor for pregnancies lasting 41+ weeks	0.5	0.3
Sudan	SDN	Complementary feeding - education only	28.0	16.2
Sudan	SDN	Complementary feeding - supplementary feeding and education	28.0	16.2
Sudan	SDN	Vitamin A supplementation	20.0	11.5
Sudan	SDN	Improved sanitation - Utilization of latrines or toilets	36.6	36.6
Sudan	SDN	Improved water source	60.3	60.3
Sudan	SDN	Water connection in the home	27.6	27.6
Sudan	SDN	Hand washing with soap	25.9	25.9
Sudan	SDN	Hygienic disposal of children's stools	53.0	53.0
Sudan	SDN	ITN/IRS - Households protected from malaria	41.4	23.9
Sudan	SDN	Injectable antibiotics for neonatal sepsis	27.7	13.3
Sudan	SDN	ORS - oral rehydration solution	19.6	9.9
Sudan	SDN	Antibiotics for treatment of dysentery	40.8	20.7
Sudan	SDN	Zinc for treatment of diarrhea	15.2	7.7
Sudan	SDN	Oral antibiotics for pneumonia	48.3	24.5
Sudan	SDN	Vitamin A for treatment of measles	20.0	10.1
Sudan	SDN	ACTs- Artemisinin compounds for treatment of malaria	14.6	7.4
Sudan	SDN	BCG vaccine	88.0	42.3
Sudan	SDN	Polio vaccine	93.0	44.7
Sudan	SDN	DPT vaccine	93.0	44.7
Sudan	SDN	H. influenzae type b vaccine	93.0	77.6
Sudan	SDN	HepB vaccine	93.0	44.7
Sudan	SDN	Pneumococcal vaccine	93.0	77.6
Sudan	SDN	Rotavirus vaccine	94.0	78.4
Sudan	SDN	Meningococcal A	84.0	40.4
Sudan	SDN	Measles vaccine	90.0	43.3
Sudan	SDN	Global wasting (<-2 SD) rate	16.2	24.3
Sudan	SDN	Contraceptive prevalence (CPR)	15.5	9.4
Suriname	SUR	Safe abortion services	0.2	0.1

Country	ISO 3166-1 alpha-3	Intervention Name	Baseline coverage (%)	Ending coverage (%)
Suriname	SUR	TT - Tetanus toxoid vaccination	93.0	44.7
Suriname	SUR	Syphilis detection and treatment	21.0	10.1
Suriname	SUR	Hypertensive disorder case management	16.2	7.8
Suriname	SUR	Diabetes case management	12.6	6.1
Suriname	SUR	Malaria case management	52.3	25.2
Suriname	SUR	MgSO4 management of pre-eclampsia	32.1	15.4
Suriname	SUR	Thermal protection	91.8	46.5
Suriname	SUR	Clean cord care	88.6	44.9
Suriname	SUR	Clean birth environment	76.2	38.6
Suriname	SUR	Immediate drying and additional stimulation	85.1	43.1
Suriname	SUR	Neonatal resuscitation	51.1	25.9
Suriname	SUR	Antibiotics for preterm or prolonged PROM	69.5	35.2
Suriname	SUR	Parenteral administration of anti-convulsants	66.4	33.6
Suriname	SUR	Parenteral administration of uterotonics	83.0	42.0
Suriname	SUR	Parenteral administration of antibiotics	69.5	35.2
Suriname	SUR	Assisted vaginal delivery	23.5	11.9
Suriname	SUR	Manual removal of placenta	34.7	17.6
Suriname	SUR	Removal of retained products of conception	30.8	15.6
Suriname	SUR	Cesarean delivery	8.1	4.1
Suriname	SUR	Blood transfusion	11.7	5.9
Suriname	SUR	Induction of labor for pregnancies lasting 41+ weeks	1.6	0.8
Suriname	SUR	Improved sanitation - Utilization of latrines or toilets	84.5	84.5
Suriname	SUR	Improved water source	95.4	95.4
Suriname	SUR	Water connection in the home	75.1	75.1
Suriname	SUR	Hand washing with soap	75.0	75.0
Suriname	SUR	Hygienic disposal of children's stools	22.0	22.0
Suriname	SUR	ITN/IRS - Households protected from malaria	0.4	0.2
Suriname	SUR	Injectable antibiotics for neonatal sepsis	92.9	44.7
Suriname	SUR	ORS - oral rehydration solution	45.6	23.1
Suriname	SUR	Oral antibiotics for pneumonia	89.1	45.1
Suriname	SUR	Polio vaccine	95.0	45.7
Suriname	SUR	DPT vaccine	95.0	45.7
Suriname	SUR	H. influenzae type b vaccine	95.0	79.2
Suriname	SUR	HepB vaccine	95.0	45.7
Suriname	SUR	Measles vaccine	97.0	46.7
Suriname	SUR	Global wasting (<-2 SD) rate	5.0	7.6
Suriname	SUR	Contraceptive prevalence (CPR)	48.05	29.2
Swaziland	SWZ	Safe abortion services	42.5	25.8
Swaziland	SWZ	TT - Tetanus toxoid vaccination	88.0	42.3
Swaziland	SWZ	Syphilis detection and treatment	24.4	11.7
Swaziland	SWZ	Iron supplementation in pregnancy	33.6	16.2

Country	ISO 3166-1 alpha-3	Intervention Name	Baseline coverage (%)	Ending coverage (%)
Swaziland	SWZ	Hypertensive disorder case management	18.3	8.8
Swaziland	SWZ	Diabetes case management	14.3	6.9
Swaziland	SWZ	Malaria case management	59.0	28.4
Swaziland	SWZ	MgSO4 management of pre-eclampsia	36.2	17.4
Swaziland	SWZ	Thermal protection	86.7	43.9
Swaziland	SWZ	Clean cord care	83.7	42.4
Swaziland	SWZ	Clean birth environment	71.9	36.4
Swaziland	SWZ	Immediate drying and additional stimulation	80.3	40.7
Swaziland	SWZ	Neonatal resuscitation	48.2	24.4
Swaziland	SWZ	Antibiotics for preterm or prolonged PROM	65.6	33.2
Swaziland	SWZ	Parenteral administration of anti-convulsants	62.7	31.7
Swaziland	SWZ	Parenteral administration of uterotonics	78.4	39.7
Swaziland	SWZ	Parenteral administration of antibiotics	65.6	33.2
Swaziland	SWZ	Assisted vaginal delivery	22.2	11.2
Swaziland	SWZ	Manual removal of placenta	32.8	16.6
Swaziland	SWZ	Removal of retained products of conception	29.1	14.7
Swaziland	SWZ	Cesarean delivery	7.6	3.8
Swaziland	SWZ	Blood transfusion	11.0	5.6
Swaziland	SWZ	Induction of labor for pregnancies lasting 41+ weeks	1.5	0.8
Swaziland	SWZ	Complementary feeding - education only	62.4	36.0
Swaziland	SWZ	Complementary feeding - supplementary feeding and education	62.4	36.0
Swaziland	SWZ	Vitamin A supplementation	33.0	19.0
Swaziland	SWZ	Improved sanitation - Utilization of latrines or toilets	58.4	58.4
Swaziland	SWZ	Improved water source	69.0	69.0
Swaziland	SWZ	Water connection in the home	42.1	42.1
Swaziland	SWZ	Hand washing with soap	26.7	26.7
Swaziland	SWZ	Hygienic disposal of children's stools	55.3	55.3
Swaziland	SWZ	ITN/IRS - Households protected from malaria	16.2	9.3
Swaziland	SWZ	Injectable antibiotics for neonatal sepsis	87.7	42.2
Swaziland	SWZ	ORS - oral rehydration solution	84.0	42.5
Swaziland	SWZ	Antibiotics for treatment of dysentery	22.3	11.3
Swaziland	SWZ	Zinc for treatment of diarrhea	45.2	22.9
Swaziland	SWZ	Oral antibiotics for pneumonia	59.6	30.2
Swaziland	SWZ	Vitamin A for treatment of measles	33.0	16.7
Swaziland	SWZ	ACTs- Artemisinin compounds for treatment of malaria	0.1	0.1
Swaziland	SWZ	BCG vaccine	98.0	47.1
Swaziland	SWZ	Polio vaccine	90.0	43.3
Swaziland	SWZ	DPT vaccine	90.0	43.3
Swaziland	SWZ	H. influenzae type b vaccine	90.0	75.1
Swaziland	SWZ	HepB vaccine	90.0	43.3

Country	ISO 3166-1 alpha-3	Intervention Name	Baseline coverage (%)	Ending coverage (%)
Swaziland	SWZ	Pneumococcal vaccine	88.0	73.4
Swaziland	SWZ	Rotavirus vaccine	86.0	71.7
Swaziland	SWZ	Measles vaccine	89.0	42.8
Swaziland	SWZ	Global wasting (<-2 SD) rate	2.1	3.1
Swaziland	SWZ	Contraceptive prevalence (CPR)	67.9	41.2
Syrian Arab Republic	SYR	Safe abortion services	40.0	24.3
Syrian Arab Republic	SYR	TT - Tetanus toxoid vaccination	91.0	43.8
Syrian Arab Republic	SYR	Syphilis detection and treatment	15.7	7.6
Syrian Arab Republic	SYR	Hypertensive disorder case management	15.3	7.4
Syrian Arab Republic	SYR	Diabetes case management	11.9	5.7
Syrian Arab Republic	SYR	Malaria case management	49.4	23.8
Syrian Arab Republic	SYR	MgSO4 management of pre-eclampsia	30.3	14.6
Syrian Arab Republic	SYR	Thermal protection	69.6	35.2
Syrian Arab Republic	SYR	Clean cord care	67.2	34.0
Syrian Arab Republic	SYR	Clean birth environment	57.7	29.2
Syrian Arab Republic	SYR	Immediate drying and additional stimulation	64.5	32.7
Syrian Arab Republic	SYR	Neonatal resuscitation	38.7	19.6
Syrian Arab Republic	SYR	Antibiotics for preterm or prolonged PROM	52.7	26.7
Syrian Arab Republic	SYR	Parenteral administration of anti-convulsants	50.3	25.5
Syrian Arab Republic	SYR	Parenteral administration of uterotonics	62.9	31.8
Syrian Arab Republic	SYR	Parenteral administration of antibiotics	52.7	26.7
Syrian Arab Republic	SYR	Assisted vaginal delivery	17.8	9.0
Syrian Arab Republic	SYR	Manual removal of placenta	26.3	13.3
Syrian Arab Republic	SYR	Removal of retained products of conception	23.3	11.8
Syrian Arab Republic	SYR	Cesarean delivery	6.1	3.1
Syrian Arab Republic	SYR	Blood transfusion	8.9	4.5
Syrian Arab Republic	SYR	Induction of labor for pregnancies lasting 41+ weeks	1.2	0.6
Syrian Arab Republic	SYR	Improved sanitation - Utilization of latrines or toilets	91.2	91.2
Syrian Arab Republic	SYR	Improved water source	97.2	97.2
Syrian Arab Republic	SYR	Water connection in the home	70.9	70.9
Syrian Arab Republic	SYR	Injectable antibiotics for neonatal sepsis	70.4	33.9
Syrian Arab Republic	SYR	ORS - oral rehydration solution	49.8	25.2
Syrian Arab Republic	SYR	Oral antibiotics for pneumonia	76.8	38.9
Syrian Arab Republic	SYR	BCG vaccine	79.0	38.0
Syrian Arab Republic	SYR	Polio vaccine	53.0	25.5
Syrian Arab Republic	SYR	DPT vaccine	47.0	22.6
Syrian Arab Republic	SYR	H. influenzae type b vaccine	48.0	40.0
Syrian Arab Republic	SYR	HepB vaccine	47.0	22.6
Syrian Arab Republic	SYR	Measles vaccine	67.0	32.2
Syrian Arab Republic	SYR	Global wasting (<-2 SD) rate	10.3	15.5
Syrian Arab Republic	SYR	Contraceptive prevalence (CPR)	59.75	36.3

Country	ISO 3166-1 alpha-3	Intervention Name	Baseline coverage (%)	Ending coverage (%)
Tajikistan	TJK	Safe abortion services	35.1	21.3
Tajikistan	TJK	Syphilis detection and treatment	23.0	11.1
Tajikistan	TJK	Iron supplementation in pregnancy	2.2	1.1
Tajikistan	TJK	Hypertensive disorder case management	15.5	7.5
Tajikistan	TJK	Diabetes case management	12.1	5.8
Tajikistan	TJK	Malaria case management	50.1	24.1
Tajikistan	TJK	MgSO4 management of pre-eclampsia	30.7	14.8
Tajikistan	TJK	Thermal protection	87.1	44.1
Tajikistan	TJK	Clean cord care	84.1	42.6
Tajikistan	TJK	Clean birth environment	72.3	36.6
Tajikistan	TJK	Immediate drying and additional stimulation	80.7	40.9
Tajikistan	TJK	Neonatal resuscitation	48.5	24.6
Tajikistan	TJK	Antibiotics for preterm or prolonged PROM	66.0	33.4
Tajikistan	TJK	Parenteral administration of anti-convulsants	63.0	31.9
Tajikistan	TJK	Parenteral administration of uterotonics	78.8	39.9
Tajikistan	TJK	Parenteral administration of antibiotics	66.0	33.4
Tajikistan	TJK	Assisted vaginal delivery	22.3	11.3
Tajikistan	TJK	Manual removal of placenta	33.0	16.7
Tajikistan	TJK	Removal of retained products of conception	29.2	14.8
Tajikistan	TJK	Cesarean delivery	7.7	3.9
Tajikistan	TJK	Blood transfusion	11.1	5.6
Tajikistan	TJK	Induction of labor for pregnancies lasting 41+ weeks	1.6	0.8
Tajikistan	TJK	Complementary feeding - education only	29.1	16.8
Tajikistan	TJK	Complementary feeding - supplementary feeding and education	29.1	16.8
Tajikistan	TJK	Vitamin A supplementation	91.0	52.5
Tajikistan	TJK	Improved sanitation - Utilization of latrines or toilets	97.0	97.0
Tajikistan	TJK	Improved water source	81.2	81.2
Tajikistan	TJK	Water connection in the home	52.2	52.2
Tajikistan	TJK	Hygienic disposal of children's stools	52.0	52.0
Tajikistan	TJK	ITN/IRS - Households protected from malaria	2.0	1.2
Tajikistan	TJK	Injectable antibiotics for neonatal sepsis	88.2	42.4
Tajikistan	TJK	ORS - oral rehydration solution	61.7	31.2
Tajikistan	TJK	Antibiotics for treatment of dysentery	59.1	29.9
Tajikistan	TJK	Zinc for treatment of diarrhea	19.7	10.0
Tajikistan	TJK	Oral antibiotics for pneumonia	68.5	34.7
Tajikistan	TJK	Vitamin A for treatment of measles	91.0	46.1
Tajikistan	TJK	ACTs- Artemisinin compounds for treatment of malaria	0.1	0.1
Tajikistan	TJK	BCG vaccine	99.0	47.6
Tajikistan	TJK	Polio vaccine	96.0	46.2
Tajikistan	TJK	DPT vaccine	96.0	46.2

Country	ISO 3166-1 alpha-3	Intervention Name	Baseline coverage (%)	Ending coverage (%)
Tajikistan	TJK	H. influenzae type b vaccine	96.0	80.1
Tajikistan	TJK	HepB vaccine	96.0	46.2
Tajikistan	TJK	Rotavirus vaccine	96.0	80.1
Tajikistan	TJK	Measles vaccine	98.0	47.1
Tajikistan	TJK	Global wasting (<-2 SD) rate	5.5	8.2
Tajikistan	TJK	Contraceptive prevalence (CPR)	31.5	19.1
Thailand	THA	Safe abortion services	39.0	23.7
Thailand	THA	TT - Tetanus toxoid vaccination	98.0	47.1
Thailand	THA	Syphilis detection and treatment	24.3	11.7
Thailand	THA	Hypertensive disorder case management	21.8	10.5
Thailand	THA	Diabetes case management	17.0	8.2
Thailand	THA	Malaria case management	70.4	33.9
Thailand	THA	MgSO4 management of pre-eclampsia	43.2	20.8
Thailand	THA	Thermal protection	97.0	49.1
Thailand	THA	Clean cord care	93.6	47.4
Thailand	THA	Clean birth environment	80.5	40.8
Thailand	THA	Immediate drying and additional stimulation	89.8	45.5
Thailand	THA	Neonatal resuscitation	54.0	27.3
Thailand	THA	Antibiotics for preterm or prolonged PROM	73.4	37.2
Thailand	THA	Parenteral administration of anti-convulsants	70.2	35.5
Thailand	THA	Parenteral administration of uterotonics	87.7	44.4
Thailand	THA	Parenteral administration of antibiotics	73.4	37.2
Thailand	THA	Assisted vaginal delivery	24.8	12.6
Thailand	THA	Manual removal of placenta	36.7	18.6
Thailand	THA	Removal of retained products of conception	32.5	16.5
Thailand	THA	Cesarean delivery	8.5	4.3
Thailand	THA	Blood transfusion	12.4	6.3
Thailand	THA	Induction of labor for pregnancies lasting 41+ weeks	1.7	0.9
Thailand	THA	Complementary feeding - education only	75.0	43.3
Thailand	THA	Complementary feeding - supplementary feeding and education	75.0	43.3
Thailand	THA	Improved sanitation - Utilization of latrines or toilets	98.8	98.8
Thailand	THA	Improved water source	99.0	99.0
Thailand	THA	Water connection in the home	69.6	69.6
Thailand	THA	Hand washing with soap	81.2	81.2
Thailand	THA	Hygienic disposal of children's stools	42.0	42.0
Thailand	THA	Injectable antibiotics for neonatal sepsis	98.1	47.2
Thailand	THA	ORS - oral rehydration solution	72.5	36.7
Thailand	THA	Oral antibiotics for pneumonia	79.5	40.2
Thailand	THA	BCG vaccine	99.0	47.6
Thailand	THA	Polio vaccine	97.0	46.7

Country	ISO 3166-1 alpha-3	Intervention Name	Baseline coverage (%)	Ending coverage (%)
Thailand	THA	DPT vaccine	97.0	46.7
Thailand	THA	HepB vaccine	97.0	46.7
Thailand	THA	Measles vaccine	99.0	47.6
Thailand	THA	Global wasting (<-2 SD) rate	5.5	8.2
Thailand	THA	Contraceptive prevalence (CPR)	79.3	48.2
Timor-Leste	TLS	Safe abortion services	39.0	23.7
Timor-Leste	TLS	TT - Tetanus toxoid vaccination	83.0	39.9
Timor-Leste	TLS	Syphilis detection and treatment	20.9	10.1
Timor-Leste	TLS	Iron supplementation in pregnancy	12.9	6.2
Timor-Leste	TLS	Hypertensive disorder case management	18.5	8.9
Timor-Leste	TLS	Diabetes case management	14.4	6.9
Timor-Leste	TLS	Malaria case management	59.6	28.7
Timor-Leste	TLS	MgSO4 management of pre-eclampsia	36.5	17.6
Timor-Leste	TLS	Thermal protection	48.0	24.3
Timor-Leste	TLS	Clean cord care	46.3	23.4
Timor-Leste	TLS	Clean birth environment	39.8	20.1
Timor-Leste	TLS	Immediate drying and additional stimulation	44.4	22.5
Timor-Leste	TLS	Neonatal resuscitation	26.7	13.5
Timor-Leste	TLS	Antibiotics for preterm or prolonged PROM	36.3	18.4
Timor-Leste	TLS	Parenteral administration of anti-convulsants	34.7	17.6
Timor-Leste	TLS	Parenteral administration of uterotonics	43.4	22.0
Timor-Leste	TLS	Parenteral administration of antibiotics	36.3	18.4
Timor-Leste	TLS	Assisted vaginal delivery	12.3	6.2
Timor-Leste	TLS	Manual removal of placenta	18.1	9.2
Timor-Leste	TLS	Removal of retained products of conception	16.1	8.2
Timor-Leste	TLS	Cesarean delivery	4.2	2.1
Timor-Leste	TLS	Blood transfusion	6.1	3.1
Timor-Leste	TLS	Induction of labor for pregnancies lasting 41+ weeks	0.9	0.5
Timor-Leste	TLS	Complementary feeding - education only	33.6	19.4
Timor-Leste	TLS	Complementary feeding - supplementary feeding and education	33.6	19.4
Timor-Leste	TLS	Vitamin A supplementation	66.0	38.1
Timor-Leste	TLS	Improved sanitation - Utilization of latrines or toilets	53.5	53.5
Timor-Leste	TLS	Improved water source	78.3	78.3
Timor-Leste	TLS	Water connection in the home	50.6	50.6
Timor-Leste	TLS	Hygienic disposal of children's stools	25.5	25.5
Timor-Leste	TLS	ITN/IRS - Households protected from malaria	64.0	36.9
Timor-Leste	TLS	Injectable antibiotics for neonatal sepsis	48.5	23.3
Timor-Leste	TLS	ORS - oral rehydration solution	69.9	35.4
Timor-Leste	TLS	Antibiotics for treatment of dysentery	6.7	3.4
Timor-Leste	TLS	Zinc for treatment of diarrhea	50.2	25.4

Country	ISO 3166-1 alpha-3	Intervention Name	Baseline coverage (%)	Ending coverage (%)
Timor-Leste	TLS	Oral antibiotics for pneumonia	70.3	35.6
Timor-Leste	TLS	Vitamin A for treatment of measles	66.0	33.4
Timor-Leste	TLS	ACTs- Artemisinin compounds for treatment of malaria	1.1	0.6
Timor-Leste	TLS	SAM - treatment for severe acute malnutrition	5.6	2.8
Timor-Leste	TLS	BCG vaccine	95.0	45.7
Timor-Leste	TLS	Polio vaccine	83.0	39.9
Timor-Leste	TLS	DPT vaccine	83.0	39.9
Timor-Leste	TLS	H. influenzae type b vaccine	83.0	69.2
Timor-Leste	TLS	HepB vaccine	83.0	39.9
Timor-Leste	TLS	Measles vaccine	70.0	33.7
Timor-Leste	TLS	Global wasting (<-2 SD) rate	24.2	36.3
Timor-Leste	TLS	Contraceptive prevalence (CPR)	30.5	18.5
Togo	TGO	TT - Tetanus toxoid vaccination	83.0	39.9
Togo	TGO	IPTp - Intermittent preventive treatment of malaria during pregnancy	68.2	32.8
Togo	TGO	Syphilis detection and treatment	18.4	8.8
Togo	TGO	Iron supplementation in pregnancy	37.1	17.8
Togo	TGO	Hypertensive disorder case management	16.7	8.0
Togo	TGO	Diabetes case management	18.3	8.8
Togo	TGO	Malaria case management	49.1	23.6
Togo	TGO	MgSO4 management of pre-eclampsia	9.5	4.6
Togo	TGO	Thermal protection	60.7	30.7
Togo	TGO	Clean cord care	46.4	23.5
Togo	TGO	Clean birth environment	42.8	21.7
Togo	TGO	Immediate drying and additional stimulation	55.4	28.0
Togo	TGO	Neonatal resuscitation	12.8	6.5
Togo	TGO	Antibiotics for preterm or prolonged PROM	48.5	24.6
Togo	TGO	Parenteral administration of anti-convulsants	10.9	5.5
Togo	TGO	Parenteral administration of uterotonics	55.4	28.0
Togo	TGO	Parenteral administration of antibiotics	48.5	24.6
Togo	TGO	Assisted vaginal delivery	8.1	4.1
Togo	TGO	Manual removal of placenta	36.1	18.3
Togo	TGO	Removal of retained products of conception	16.9	8.6
Togo	TGO	Cesarean delivery	0.8	0.4
Togo	TGO	Blood transfusion	7.7	3.9
Togo	TGO	Induction of labor for pregnancies lasting 41+ weeks	1.1	0.6
Togo	TGO	Complementary feeding - education only	20.3	11.7
Togo	TGO	Complementary feeding - supplementary feeding and education	20.3	11.7
Togo	TGO	Vitamin A supplementation	87.0	50.2
Togo	TGO	Improved sanitation - Utilization of latrines or toilets	16.1	16.1
Togo	TGO	Improved water source	65.1	65.1

Country	ISO 3166-1 alpha-3	Intervention Name	Baseline coverage (%)	Ending coverage (%)
Togo	TGO	Water connection in the home	16.7	16.7
Togo	TGO	Hand washing with soap	62.0	62.0
Togo	TGO	Hygienic disposal of children's stools	34.8	34.8
Togo	TGO	ITN/IRS - Households protected from malaria	65.4	37.7
Togo	TGO	Injectable antibiotics for neonatal sepsis	61.4	29.5
Togo	TGO	ORS - oral rehydration solution	18.5	9.4
Togo	TGO	Antibiotics for treatment of dysentery	29.7	15.0
Togo	TGO	Zinc for treatment of diarrhea	0.1	0.1
Togo	TGO	Oral antibiotics for pneumonia	48.5	24.6
Togo	TGO	Vitamin A for treatment of measles	87.0	44.0
Togo	TGO	ACTs- Artemisinin compounds for treatment of malaria	6.3	3.2
Togo	TGO	SAM - treatment for severe acute malnutrition	6.4	3.2
Togo	TGO	BCG vaccine	83.0	39.9
Togo	TGO	Polio vaccine	66.0	31.7
Togo	TGO	DPT vaccine	88.0	42.3
Togo	TGO	H. influenzae type b vaccine	88.0	73.4
Togo	TGO	HepB vaccine	88.0	42.3
Togo	TGO	Pneumococcal vaccine	88.0	73.4
Togo	TGO	Rotavirus vaccine	89.0	74.2
Togo	TGO	Measles vaccine	91.0	43.8
Togo	TGO	Global wasting (<-2 SD) rate	6.6	9.9
Togo	TGO	Contraceptive prevalence (CPR)	24.65	15.0
Tonga	TON	Safe abortion services	85.0	51.6
Tonga	TON	TT - Tetanus toxoid vaccination	71.2	34.2
Tonga	TON	Syphilis detection and treatment	19.3	9.3
Tonga	TON	Hypertensive disorder case management	16.9	8.1
Tonga	TON	Diabetes case management	13.2	6.3
Tonga	TON	Malaria case management	54.6	26.3
Tonga	TON	MgSO4 management of pre-eclampsia	33.5	16.1
Tonga	TON	Thermal protection	96.8	49.0
Tonga	TON	Clean cord care	93.4	47.3
Tonga	TON	Clean birth environment	80.3	40.7
Tonga	TON	Immediate drying and additional stimulation	89.6	45.4
Tonga	TON	Neonatal resuscitation	53.8	27.2
Tonga	TON	Antibiotics for preterm or prolonged PROM	73.3	37.1
Tonga	TON	Parenteral administration of anti-convulsants	70.0	35.4
Tonga	TON	Parenteral administration of uterotonics	87.5	44.3
Tonga	TON	Parenteral administration of antibiotics	73.3	37.1
Tonga	TON	Assisted vaginal delivery	24.7	12.5
Tonga	TON	Manual removal of placenta	36.6	18.5
Tonga	TON	Removal of retained products of conception	32.4	16.4

Country	ISO 3166-1 alpha-3	Intervention Name	Baseline coverage (%)	Ending coverage (%)
Tonga	TON	Cesarean delivery	8.5	4.3
Tonga	TON	Blood transfusion	12.3	6.2
Tonga	TON	Induction of labor for pregnancies lasting 41+ weeks	1.7	0.9
Tonga	TON	Improved sanitation - Utilization of latrines or toilets	93.4	93.4
Tonga	TON	Improved water source	99.0	99.0
Tonga	TON	Water connection in the home	78.2	78.2
Tonga	TON	Hygienic disposal of children's stools	40.4	40.4
Tonga	TON	Injectable antibiotics for neonatal sepsis	97.9	47.1
Tonga	TON	BCG vaccine	88.0	42.3
Tonga	TON	Polio vaccine	83.0	39.9
Tonga	TON	DPT vaccine	81.0	39.0
Tonga	TON	H. influenzae type b vaccine	81.0	67.5
Tonga	TON	HepB vaccine	81.0	39.0
Tonga	TON	Measles vaccine	85.0	40.9
Tonga	TON	Global wasting (<-2 SD) rate	10.6	15.9
Tonga	TON	Contraceptive prevalence (CPR)	37.55	22.8
Tunisia	TUN	Safe abortion services	2.2	1.3
Tunisia	TUN	TT - Tetanus toxoid vaccination	96.0	46.2
Tunisia	TUN	Syphilis detection and treatment	23.6	11.4
Tunisia	TUN	Hypertensive disorder case management	20.2	9.7
Tunisia	TUN	Diabetes case management	15.8	7.6
Tunisia	TUN	Malaria case management	65.2	31.4
Tunisia	TUN	MgSO4 management of pre-eclampsia	40.0	19.2
Tunisia	TUN	Thermal protection	98.5	49.9
Tunisia	TUN	Clean cord care	95.1	48.1
Tunisia	TUN	Clean birth environment	81.8	41.4
Tunisia	TUN	Immediate drying and additional stimulation	91.3	46.2
Tunisia	TUN	Neonatal resuscitation	54.8	27.7
Tunisia	TUN	Antibiotics for preterm or prolonged PROM	74.6	37.8
Tunisia	TUN	Parenteral administration of anti-convulsants	71.3	36.1
Tunisia	TUN	Parenteral administration of uterotonics	89.1	45.1
Tunisia	TUN	Parenteral administration of antibiotics	74.6	37.8
Tunisia	TUN	Assisted vaginal delivery	25.2	12.8
Tunisia	TUN	Manual removal of placenta	37.3	18.9
Tunisia	TUN	Removal of retained products of conception	33.0	16.7
Tunisia	TUN	Cesarean delivery	8.7	4.4
Tunisia	TUN	Blood transfusion	12.6	6.4
Tunisia	TUN	Induction of labor for pregnancies lasting 41+ weeks	1.8	0.9
Tunisia	TUN	Complementary feeding - education only	69.5	40.1
Tunisia	TUN	Complementary feeding - supplementary feeding and education	69.5	40.1

Country	ISO 3166-1 alpha-3	Intervention Name	Baseline coverage (%)	Ending coverage (%)
Tunisia	TUN	Improved sanitation - Utilization of latrines or toilets	90.9	90.9
Tunisia	TUN	Improved water source	96.3	96.3
Tunisia	TUN	Water connection in the home	88.9	88.9
Tunisia	TUN	Hand washing with soap	94.2	94.2
Tunisia	TUN	Hygienic disposal of children's stools	13.8	13.8
Tunisia	TUN	Injectable antibiotics for neonatal sepsis	99.7	47.9
Tunisia	TUN	ORS - oral rehydration solution	39.6	20.0
Tunisia	TUN	Zinc for treatment of diarrhea	3.9	2.0
Tunisia	TUN	Oral antibiotics for pneumonia	77.0	39.0
Tunisia	TUN	BCG vaccine	92.0	44.2
Tunisia	TUN	Polio vaccine	97.0	46.7
Tunisia	TUN	DPT vaccine	97.0	46.7
Tunisia	TUN	H. influenzae type b vaccine	97.0	80.9
Tunisia	TUN	HepB vaccine	97.0	46.7
Tunisia	TUN	Measles vaccine	98.0	47.1
Tunisia	TUN	Global wasting (<-2 SD) rate	2.8	4.2
Tunisia	TUN	Contraceptive prevalence (CPR)	59.25	36.0
Turkey	TUR	Safe abortion services	40.0	24.3
Turkey	TUR	TT - Tetanus toxoid vaccination	95.0	45.7
Turkey	TUR	Syphilis detection and treatment	23.9	11.5
Turkey	TUR	Hypertensive disorder case management	21.4	10.3
Turkey	TUR	Diabetes case management	16.7	8.0
Turkey	TUR	Malaria case management	68.9	33.1
Turkey	TUR	MgSO4 management of pre-eclampsia	42.3	20.3
Turkey	TUR	Thermal protection	96.1	48.7
Turkey	TUR	Clean cord care	92.8	47.0
Turkey	TUR	Clean birth environment	79.7	40.3
Turkey	TUR	Immediate drying and additional stimulation	89.0	45.1
Turkey	TUR	Neonatal resuscitation	53.4	27.0
Turkey	TUR	Antibiotics for preterm or prolonged PROM	72.7	36.8
Turkey	TUR	Parenteral administration of anti-convulsants	69.5	35.2
Turkey	TUR	Parenteral administration of uterotonics	86.9	44.0
Turkey	TUR	Parenteral administration of antibiotics	72.7	36.8
Turkey	TUR	Assisted vaginal delivery	24.6	12.5
Turkey	TUR	Manual removal of placenta	36.4	18.4
Turkey	TUR	Removal of retained products of conception	32.2	16.3
Turkey	TUR	Cesarean delivery	8.5	4.3
Turkey	TUR	Blood transfusion	12.2	6.2
Turkey	TUR	Induction of labor for pregnancies lasting 41+ weeks	1.7	0.9
Turkey	TUR	Improved sanitation - Utilization of latrines or toilets	97.3	97.3
Turkey	TUR	Improved water source	98.9	98.9

Country	ISO 3166-1 alpha-3	Intervention Name	Baseline coverage (%)	Ending coverage (%)
Turkey	TUR	Water connection in the home	97.8	97.8
Turkey	TUR	Injectable antibiotics for neonatal sepsis	97.2	46.7
Turkey	TUR	ORS - oral rehydration solution	20.4	10.3
Turkey	TUR	Antibiotics for treatment of dysentery	7.5	3.8
Turkey	TUR	BCG vaccine	96.0	46.2
Turkey	TUR	Polio vaccine	98.0	47.1
Turkey	TUR	DPT vaccine	98.0	47.1
Turkey	TUR	H. influenzae type b vaccine	98.0	81.7
Turkey	TUR	HepB vaccine	98.0	47.1
Turkey	TUR	Pneumococcal vaccine	97.0	80.9
Turkey	TUR	Measles vaccine	96.0	46.2
Turkey	TUR	Global wasting (<-2 SD) rate	1.1	1.6
Turkey	TUR	Contraceptive prevalence (CPR)	74.45	45.2
Turkmenistan	TKM	Safe abortion services	35.1	21.3
Turkmenistan	TKM	Syphilis detection and treatment	24.7	11.9
Turkmenistan	TKM	Hypertensive disorder case management	23.2	11.2
Turkmenistan	TKM	Diabetes case management	18.1	8.7
Turkmenistan	TKM	Malaria case management	74.7	35.9
Turkmenistan	TKM	MgSO4 management of pre-eclampsia	45.8	22.0
Turkmenistan	TKM	Thermal protection	98.3	49.8
Turkmenistan	TKM	Clean cord care	94.9	48.0
Turkmenistan	TKM	Clean birth environment	81.6	41.3
Turkmenistan	TKM	Immediate drying and additional stimulation	91.1	46.1
Turkmenistan	TKM	Neonatal resuscitation	54.7	27.7
Turkmenistan	TKM	Antibiotics for preterm or prolonged PROM	74.4	37.7
Turkmenistan	TKM	Parenteral administration of anti-convulsants	71.1	36.0
Turkmenistan	TKM	Parenteral administration of uterotonics	88.9	45.0
Turkmenistan	TKM	Parenteral administration of antibiotics	74.4	37.7
Turkmenistan	TKM	Assisted vaginal delivery	25.1	12.7
Turkmenistan	TKM	Manual removal of placenta	37.2	18.8
Turkmenistan	TKM	Removal of retained products of conception	33.0	16.7
Turkmenistan	TKM	Cesarean delivery	8.7	4.4
Turkmenistan	TKM	Blood transfusion	12.5	6.3
Turkmenistan	TKM	Induction of labor for pregnancies lasting 41+ weeks	1.8	0.9
Turkmenistan	TKM	Complementary feeding - education only	85.2	49.2
Turkmenistan	TKM	Complementary feeding - supplementary feeding and education	85.2	49.2
Turkmenistan	TKM	Improved sanitation - Utilization of latrines or toilets	98.7	98.7
Turkmenistan	TKM	Improved water source	98.8	98.8
Turkmenistan	TKM	Water connection in the home	56.3	56.3
Turkmenistan	TKM	Hand washing with soap	99.3	99.3

**Country**	**ISO 3166-1 alpha-3**	**Intervention Name**	**Baseline coverage (%)**	**Ending coverage (%)**
Turkmenistan	TKM	Hygienic disposal of children's stools	60.5	60.5
Turkmenistan	TKM	Injectable antibiotics for neonatal sepsis	99.5	47.9
Turkmenistan	TKM	ORS - oral rehydration solution	47.1	23.8
Turkmenistan	TKM	Zinc for treatment of diarrhea	10.9	5.5
Turkmenistan	TKM	Oral antibiotics for pneumonia	82.7	41.9
Turkmenistan	TKM	BCG vaccine	98.0	47.1
Turkmenistan	TKM	Polio vaccine	99.0	47.6
Turkmenistan	TKM	DPT vaccine	99.0	47.6
Turkmenistan	TKM	H. influenzae type b vaccine	99.0	82.6
Turkmenistan	TKM	HepB vaccine	99.0	47.6
Turkmenistan	TKM	Measles vaccine	99.0	47.6
Turkmenistan	TKM	Global wasting (<-2 SD) rate	4.3	6.4
Turkmenistan	TKM	Contraceptive prevalence (CPR)	53.65	32.6
Uganda	UGA	Safe abortion services	3.3	2.0
Uganda	UGA	TT - Tetanus toxoid vaccination	85.0	40.9
Uganda	UGA	IPTp - Intermittent preventive treatment of malaria during pregnancy	45.9	22.1
Uganda	UGA	Syphilis detection and treatment	28.1	13.5
Uganda	UGA	Iron supplementation in pregnancy	22.6	10.9
Uganda	UGA	Hypertensive disorder case management	17.7	8.5
Uganda	UGA	Diabetes case management	10.1	4.9
Uganda	UGA	Malaria case management	40.6	19.5
Uganda	UGA	MgSO4 management of pre-eclampsia	23.1	11.1
Uganda	UGA	Thermal protection	72.5	36.7
Uganda	UGA	Clean cord care	63.6	32.2
Uganda	UGA	Clean birth environment	57.7	29.2
Uganda	UGA	Immediate drying and additional stimulation	56.8	28.8
Uganda	UGA	Neonatal resuscitation	36.2	18.3
Uganda	UGA	Antibiotics for preterm or prolonged PROM	50.0	25.3
Uganda	UGA	Parenteral administration of anti-convulsants	40.9	20.7
Uganda	UGA	Parenteral administration of uterotonics	65.6	33.2
Uganda	UGA	Parenteral administration of antibiotics	50.0	25.3
Uganda	UGA	Assisted vaginal delivery	25.6	13.0
Uganda	UGA	Manual removal of placenta	24.2	12.3
Uganda	UGA	Removal of retained products of conception	28.4	14.4
Uganda	UGA	Cesarean delivery	2.3	1.2
Uganda	UGA	Blood transfusion	25.3	12.8
Uganda	UGA	Induction of labor for pregnancies lasting 41+ weeks	11.5	5.8
Uganda	UGA	Complementary feeding - education only	30.2	17.4
Uganda	UGA	Complementary feeding - supplementary feeding and education	30.2	17.4
Uganda	UGA	Vitamin A supplementation	27.0	15.6

Country	ISO 3166-1 alpha-3	Intervention Name	Baseline coverage (%)	Ending coverage (%)
Uganda	UGA	Improved sanitation - Utilization of latrines or toilets	18.5	18.5
Uganda	UGA	Improved water source	49.1	49.1
Uganda	UGA	Water connection in the home	10.4	10.4
Uganda	UGA	Hand washing with soap	27.4	27.4
Uganda	UGA	Hygienic disposal of children's stools	76.3	76.3
Uganda	UGA	ITN/IRS - Households protected from malaria	80.8	46.6
Uganda	UGA	Injectable antibiotics for neonatal sepsis	73.4	35.3
Uganda	UGA	ORS - oral rehydration solution	46.7	23.6
Uganda	UGA	Antibiotics for treatment of dysentery	32.1	16.3
Uganda	UGA	Zinc for treatment of diarrhea	40.3	20.4
Uganda	UGA	Oral antibiotics for pneumonia	71.3	36.1
Uganda	UGA	Vitamin A for treatment of measles	27.0	13.7
Uganda	UGA	ACTs- Artemisinin compounds for treatment of malaria	45.9	23.2
Uganda	UGA	SAM - treatment for severe acute malnutrition	2.8	1.4
Uganda	UGA	BCG vaccine	88.0	42.3
Uganda	UGA	Polio vaccine	88.0	42.3
Uganda	UGA	DPT vaccine	93.0	44.7
Uganda	UGA	H. influenzae type b vaccine	93.0	77.6
Uganda	UGA	HepB vaccine	93.0	44.7
Uganda	UGA	Pneumococcal vaccine	92.0	76.7
Uganda	UGA	Rotavirus vaccine	36.0	30.0
Uganda	UGA	Measles vaccine	80.0	38.5
Uganda	UGA	Global wasting (<-2 SD) rate	3.6	5.3
Uganda	UGA	Contraceptive prevalence (CPR)	43.55	26.5
Ukraine	UKR	Safe abortion services	87.5	53.2
Ukraine	UKR	IPTp - Intermittent preventive treatment of malaria during pregnancy	41.1	19.8
Ukraine	UKR	Syphilis detection and treatment	24.4	11.7
Ukraine	UKR	Iron supplementation in pregnancy	3.4	1.6
Ukraine	UKR	Hypertensive disorder case management	21.0	10.1
Ukraine	UKR	Diabetes case management	16.3	7.8
Ukraine	UKR	Malaria case management	67.6	32.5
Ukraine	UKR	MgSO4 management of pre-eclampsia	41.5	20.0
Ukraine	UKR	Thermal protection	97.7	49.5
Ukraine	UKR	Clean cord care	94.4	47.8
Ukraine	UKR	Clean birth environment	81.1	41.1
Ukraine	UKR	Immediate drying and additional stimulation	90.5	45.8
Ukraine	UKR	Neonatal resuscitation	54.4	27.5
Ukraine	UKR	Antibiotics for preterm or prolonged PROM	74.0	37.5
Ukraine	UKR	Parenteral administration of anti-convulsants	70.7	35.8
Ukraine	UKR	Parenteral administration of uterotonics	88.4	44.8

Country	ISO 3166-1 alpha-3	Intervention Name	Baseline coverage (%)	Ending coverage (%)
Ukraine	UKR	Parenteral administration of antibiotics	74.0	37.5
Ukraine	UKR	Assisted vaginal delivery	25.0	12.7
Ukraine	UKR	Manual removal of placenta	37.0	18.7
Ukraine	UKR	Removal of retained products of conception	32.8	16.6
Ukraine	UKR	Cesarean delivery	8.6	4.4
Ukraine	UKR	Blood transfusion	12.5	6.3
Ukraine	UKR	Induction of labor for pregnancies lasting 41+ weeks	1.7	0.9
Ukraine	UKR	Complementary feeding - education only	58.5	33.8
Ukraine	UKR	Complementary feeding - supplementary feeding and education	58.5	33.8
Ukraine	UKR	Improved sanitation - Utilization of latrines or toilets	96.2	96.2
Ukraine	UKR	Improved water source	93.8	93.8
Ukraine	UKR	Water connection in the home	62.0	62.0
Ukraine	UKR	Injectable antibiotics for neonatal sepsis	98.9	47.6
Ukraine	UKR	ORS - oral rehydration solution	59.2	30.0
Ukraine	UKR	Oral antibiotics for pneumonia	92.3	46.7
Ukraine	UKR	BCG vaccine	90.0	43.3
Ukraine	UKR	Polio vaccine	48.0	23.1
Ukraine	UKR	DPT vaccine	50.0	24.0
Ukraine	UKR	H. influenzae type b vaccine	39.0	32.5
Ukraine	UKR	HepB vaccine	52.0	25.0
Ukraine	UKR	Measles vaccine	86.0	41.4
Ukraine	UKR	Global wasting (<-2 SD) rate	4.5	6.8
Ukraine	UKR	Contraceptive prevalence (CPR)	67.4	40.9
United Republic of Tanzania	TZA	Safe abortion services	3.3	2.0
United Republic of Tanzania	TZA	TT - Tetanus toxoid vaccination	90.0	43.3
United Republic of Tanzania	TZA	IPTp - Intermittent preventive treatment of malaria during pregnancy	56.1	27.0
United Republic of Tanzania	TZA	Syphilis detection and treatment	56.2	27.0
United Republic of Tanzania	TZA	Iron supplementation in pregnancy	21.4	10.3
United Republic of Tanzania	TZA	Hypertensive disorder case management	11.8	5.7
United Republic of Tanzania	TZA	Diabetes case management	9.2	4.4
United Republic of Tanzania	TZA	Malaria case management	42.3	20.3
United Republic of Tanzania	TZA	MgSO4 management of pre-eclampsia	34.8	16.7
United Republic of Tanzania	TZA	Thermal protection	61.7	31.2
United Republic of Tanzania	TZA	Clean cord care	60.8	30.8

Country	ISO 3166-1 alpha-3	Intervention Name	Baseline coverage (%)	Ending coverage (%)
United Republic of Tanzania	TZA	Clean birth environment	51.6	26.1
United Republic of Tanzania	TZA	Immediate drying and additional stimulation	57.4	29.1
United Republic of Tanzania	TZA	Neonatal resuscitation	53.6	27.1
United Republic of Tanzania	TZA	Antibiotics for preterm or prolonged PROM	32.2	16.3
United Republic of Tanzania	TZA	Parenteral administration of anti-convulsants	49.9	25.3
United Republic of Tanzania	TZA	Parenteral administration of uterotonics	57.1	28.9
United Republic of Tanzania	TZA	Parenteral administration of antibiotics	32.2	16.3
United Republic of Tanzania	TZA	Assisted vaginal delivery	15.8	8.0
United Republic of Tanzania	TZA	Manual removal of placenta	24.5	12.4
United Republic of Tanzania	TZA	Removal of retained products of conception	15.3	7.7
United Republic of Tanzania	TZA	Cesarean delivery	5.4	2.7
United Republic of Tanzania	TZA	Blood transfusion	16.9	8.6
United Republic of Tanzania	TZA	Induction of labor for pregnancies lasting 41+ weeks	6.5	3.3
United Republic of Tanzania	TZA	Complementary feeding - education only	26.0	15.0
United Republic of Tanzania	TZA	Complementary feeding - supplementary feeding and education	26.0	15.0
United Republic of Tanzania	TZA	Vitamin A supplementation	87.0	50.2
United Republic of Tanzania	TZA	Improved sanitation - Utilization of latrines or toilets	29.9	29.9
United Republic of Tanzania	TZA	Improved water source	56.7	56.7
United Republic of Tanzania	TZA	Water connection in the home	20.3	20.3
United Republic of Tanzania	TZA	Hand washing with soap	51.9	51.9
United Republic of Tanzania	TZA	Hygienic disposal of children's stools	67.9	67.9
United Republic of Tanzania	TZA	ITN/IRS - Households protected from malaria	77.9	45.0
United Republic of Tanzania	TZA	Injectable antibiotics for neonatal sepsis	62.6	30.1
United Republic of Tanzania	TZA	ORS - oral rehydration solution	44.8	22.7
United Republic of Tanzania	TZA	Antibiotics for treatment of dysentery	2.8	1.4
United Republic of Tanzania	TZA	Zinc for treatment of diarrhea	17.5	8.9

Country	ISO 3166-1 alpha-3	Intervention Name	Baseline coverage (%)	Ending coverage (%)
United Republic of Tanzania	TZA	Oral antibiotics for pneumonia	55.4	28.0
United Republic of Tanzania	TZA	Vitamin A for treatment of measles	87.0	44.0
United Republic of Tanzania	TZA	ACTs- Artemisinin compounds for treatment of malaria	30.0	15.2
United Republic of Tanzania	TZA	SAM - treatment for severe acute malnutrition	0.3	0.2
United Republic of Tanzania	TZA	BCG vaccine	99.0	47.6
United Republic of Tanzania	TZA	Polio vaccine	91.0	43.8
United Republic of Tanzania	TZA	DPT vaccine	98.0	47.1
United Republic of Tanzania	TZA	H. influenzae type b vaccine	98.0	81.7
United Republic of Tanzania	TZA	HepB vaccine	98.0	47.1
United Republic of Tanzania	TZA	Pneumococcal vaccine	98.0	81.7
United Republic of Tanzania	TZA	Rotavirus vaccine	98.0	81.7
United Republic of Tanzania	TZA	Measles vaccine	99.0	47.6
United Republic of Tanzania	TZA	Global wasting (<-2 SD) rate	4.6	6.9
United Republic of Tanzania	TZA	Contraceptive prevalence (CPR)	43.8	26.6
Uzbekistan	UZB	Safe abortion services	35.1	21.3
Uzbekistan	UZB	Syphilis detection and treatment	24.5	11.8
Uzbekistan	UZB	Hypertensive disorder case management	18.8	9.0
Uzbekistan	UZB	Diabetes case management	14.7	7.1
Uzbekistan	UZB	Malaria case management	60.7	29.2
Uzbekistan	UZB	MgSO4 management of pre-eclampsia	37.2	17.9
Uzbekistan	UZB	Thermal protection	96.2	48.7
Uzbekistan	UZB	Clean cord care	92.9	47.0
Uzbekistan	UZB	Clean birth environment	79.8	40.4
Uzbekistan	UZB	Immediate drying and additional stimulation	89.1	45.1
Uzbekistan	UZB	Neonatal resuscitation	53.5	27.1
Uzbekistan	UZB	Antibiotics for preterm or prolonged PROM	72.8	36.9
Uzbekistan	UZB	Parenteral administration of anti-convulsants	69.6	35.2
Uzbekistan	UZB	Parenteral administration of uterotonics	87.0	44.0
Uzbekistan	UZB	Parenteral administration of antibiotics	72.8	36.9
Uzbekistan	UZB	Assisted vaginal delivery	24.6	12.5
Uzbekistan	UZB	Manual removal of placenta	36.4	18.4
Uzbekistan	UZB	Removal of retained products of conception	32.3	16.4
Uzbekistan	UZB	Cesarean delivery	8.5	4.3

Country	ISO 3166-1 alpha-3	Intervention Name	Baseline coverage (%)	Ending coverage (%)
Uzbekistan	UZB	Blood transfusion	12.3	6.2
Uzbekistan	UZB	Induction of labor for pregnancies lasting 41+ weeks	1.7	0.9
Uzbekistan	UZB	Complementary feeding - education only	27.6	15.9
Uzbekistan	UZB	Complementary feeding - supplementary feeding and education	27.6	15.9
Uzbekistan	UZB	Vitamin A supplementation	99.0	57.1
Uzbekistan	UZB	Improved sanitation - Utilization of latrines or toilets	99.0	99.0
Uzbekistan	UZB	Improved water source	97.8	97.8
Uzbekistan	UZB	Water connection in the home	68.7	68.7
Uzbekistan	UZB	Hygienic disposal of children's stools	58.6	58.6
Uzbekistan	UZB	Injectable antibiotics for neonatal sepsis	97.3	46.8
Uzbekistan	UZB	ORS - oral rehydration solution	27.8	14.1
Uzbekistan	UZB	Oral antibiotics for pneumonia	67.7	34.3
Uzbekistan	UZB	Vitamin A for treatment of measles	99.0	50.1
Uzbekistan	UZB	BCG vaccine	96.0	46.2
Uzbekistan	UZB	Polio vaccine	98.0	47.1
Uzbekistan	UZB	DPT vaccine	98.0	47.1
Uzbekistan	UZB	H. influenzae type b vaccine	98.0	81.7
Uzbekistan	UZB	HepB vaccine	98.0	47.1
Uzbekistan	UZB	Pneumococcal vaccine	96.0	80.1
Uzbekistan	UZB	Rotavirus vaccine	84.0	70.0
Uzbekistan	UZB	Measles vaccine	99.0	47.6
Uzbekistan	UZB	Global wasting (<-2 SD) rate	4.4	6.7
Uzbekistan	UZB	Contraceptive prevalence (CPR)	68.35	41.5
Vanuatu	VUT	Safe abortion services	85.0	51.6
Vanuatu	VUT	TT - Tetanus toxoid vaccination	78.0	37.5
Vanuatu	VUT	Syphilis detection and treatment	19.1	9.2
Vanuatu	VUT	Hypertensive disorder case management	12.4	6.0
Vanuatu	VUT	Diabetes case management	9.7	4.7
Vanuatu	VUT	Malaria case management	40.2	19.3
Vanuatu	VUT	MgSO4 management of pre-eclampsia	24.6	11.8
Vanuatu	VUT	Thermal protection	87.5	44.3
Vanuatu	VUT	Clean cord care	84.5	42.8
Vanuatu	VUT	Clean birth environment	72.6	36.8
Vanuatu	VUT	Immediate drying and additional stimulation	81.0	41.0
Vanuatu	VUT	Neonatal resuscitation	48.7	24.7
Vanuatu	VUT	Antibiotics for preterm or prolonged PROM	66.2	33.5
Vanuatu	VUT	Parenteral administration of anti-convulsants	63.3	32.0
Vanuatu	VUT	Parenteral administration of uterotonics	79.1	40.0
Vanuatu	VUT	Parenteral administration of antibiotics	66.2	33.5
Vanuatu	VUT	Assisted vaginal delivery	22.4	11.3

Country	ISO 3166-1 alpha-3	Intervention Name	Baseline coverage (%)	Ending coverage (%)
Vanuatu	VUT	Manual removal of placenta	33.1	16.8
Vanuatu	VUT	Removal of retained products of conception	29.3	14.8
Vanuatu	VUT	Cesarean delivery	7.7	3.9
Vanuatu	VUT	Blood transfusion	11.1	5.6
Vanuatu	VUT	Induction of labor for pregnancies lasting 41+ weeks	1.6	0.8
Vanuatu	VUT	Improved sanitation - Utilization of latrines or toilets	34.1	34.1
Vanuatu	VUT	Improved water source	91.3	91.3
Vanuatu	VUT	Water connection in the home	43.5	43.5
Vanuatu	VUT	Hygienic disposal of children's stools	62.7	62.7
Vanuatu	VUT	ITN/IRS - Households protected from malaria	69.8	40.3
Vanuatu	VUT	Injectable antibiotics for neonatal sepsis	88.5	42.6
Vanuatu	VUT	ORS - oral rehydration solution	47.6	24.1
Vanuatu	VUT	Antibiotics for treatment of dysentery	3.0	1.5
Vanuatu	VUT	Zinc for treatment of diarrhea	1.0	0.5
Vanuatu	VUT	Oral antibiotics for pneumonia	72.1	36.5
Vanuatu	VUT	BCG vaccine	94.0	45.2
Vanuatu	VUT	Polio vaccine	85.0	40.9
Vanuatu	VUT	DPT vaccine	85.0	40.9
Vanuatu	VUT	H. influenzae type b vaccine	85.0	70.9
Vanuatu	VUT	HepB vaccine	85.0	40.9
Vanuatu	VUT	Measles vaccine	80.0	38.5
Vanuatu	VUT	Global wasting (<-2 SD) rate	5.7	8.6
Vanuatu	VUT	Contraceptive prevalence (CPR)	50.25	30.5
Venezuela	VEN	Safe abortion services	0.2	0.1
Venezuela	VEN	TT - Tetanus toxoid vaccination	70.0	33.7
Venezuela	VEN	Thermal protection	93.9	47.5
Venezuela	VEN	Clean cord care	90.7	45.9
Venezuela	VEN	Clean birth environment	77.9	39.4
Venezuela	VEN	Immediate drying and additional stimulation	87.0	44.0
Venezuela	VEN	Neonatal resuscitation	52.2	26.4
Venezuela	VEN	Antibiotics for preterm or prolonged PROM	71.1	36.0
Venezuela	VEN	Parenteral administration of anti-convulsants	67.9	34.4
Venezuela	VEN	Parenteral administration of uterotonics	84.9	43.0
Venezuela	VEN	Parenteral administration of antibiotics	71.1	36.0
Venezuela	VEN	Assisted vaginal delivery	24.0	12.2
Venezuela	VEN	Manual removal of placenta	35.5	18.0
Venezuela	VEN	Removal of retained products of conception	31.5	15.9
Venezuela	VEN	Cesarean delivery	8.3	4.2
Venezuela	VEN	Blood transfusion	12.0	6.1
Venezuela	VEN	Induction of labor for pregnancies lasting 41+ weeks	1.7	0.9
Venezuela	VEN	Improved sanitation - Utilization of latrines or toilets	93.9	93.9

Country	ISO 3166-1 alpha-3	Intervention Name	Baseline coverage (%)	Ending coverage (%)
Venezuela	VEN	Improved water source	95.7	95.7
Venezuela	VEN	Water connection in the home	83.7	83.7
Venezuela	VEN	Injectable antibiotics for neonatal sepsis	95.0	45.7
Venezuela	VEN	BCG vaccine	92.0	44.2
Venezuela	VEN	Polio vaccine	53.0	25.5
Venezuela	VEN	DPT vaccine	60.0	28.9
Venezuela	VEN	H. influenzae type b vaccine	60.0	50.0
Venezuela	VEN	HepB vaccine	60.0	28.9
Venezuela	VEN	Measles vaccine	96.0	46.2
Venezuela	VEN	Global wasting (<-2 SD) rate	2.4	3.6
Venezuela	VEN	Contraceptive prevalence (CPR)	75.7	46.0
Viet Nam	VNM	Safe abortion services	39.0	23.7
Viet Nam	VNM	TT - Tetanus toxoid vaccination	94.0	45.2
Viet Nam	VNM	IPTp - Intermittent preventive treatment of malaria during pregnancy	0.5	0.2
Viet Nam	VNM	Syphilis detection and treatment	23.7	11.4
Viet Nam	VNM	Hypertensive disorder case management	17.7	8.5
Viet Nam	VNM	Diabetes case management	13.8	6.6
Viet Nam	VNM	Malaria case management	57.1	27.5
Viet Nam	VNM	MgSO4 management of pre-eclampsia	35.0	16.8
Viet Nam	VNM	Thermal protection	92.5	46.8
Viet Nam	VNM	Clean cord care	89.4	45.3
Viet Nam	VNM	Clean birth environment	76.8	38.9
Viet Nam	VNM	Immediate drying and additional stimulation	85.7	43.4
Viet Nam	VNM	Neonatal resuscitation	51.5	26.1
Viet Nam	VNM	Antibiotics for preterm or prolonged PROM	70.1	35.5
Viet Nam	VNM	Parenteral administration of anti-convulsants	67.0	33.9
Viet Nam	VNM	Parenteral administration of uterotonics	83.7	42.4
Viet Nam	VNM	Parenteral administration of antibiotics	70.1	35.5
Viet Nam	VNM	Assisted vaginal delivery	23.7	12.0
Viet Nam	VNM	Manual removal of placenta	35.0	17.7
Viet Nam	VNM	Removal of retained products of conception	31.0	15.7
Viet Nam	VNM	Cesarean delivery	8.1	4.1
Viet Nam	VNM	Blood transfusion	11.8	6.0
Viet Nam	VNM	Induction of labor for pregnancies lasting 41+ weeks	1.6	0.8
Viet Nam	VNM	Complementary feeding - education only	76.9	44.4
Viet Nam	VNM	Complementary feeding - supplementary feeding and education	76.9	44.4
Viet Nam	VNM	Vitamin A supplementation	99.0	57.1
Viet Nam	VNM	Improved sanitation - Utilization of latrines or toilets	83.5	83.5
Viet Nam	VNM	Improved water source	94.7	94.7
Viet Nam	VNM	Water connection in the home	40.6	40.6

Country	ISO 3166-1 alpha-3	Intervention Name	Baseline coverage (%)	Ending coverage (%)
Viet Nam	VNM	Hand washing with soap	86.3	86.3
Viet Nam	VNM	Hygienic disposal of children's stools	57.7	57.7
Viet Nam	VNM	ITN/IRS - Households protected from malaria	24.9	14.4
Viet Nam	VNM	Injectable antibiotics for neonatal sepsis	93.6	45.0
Viet Nam	VNM	ORS - oral rehydration solution	50.9	25.8
Viet Nam	VNM	Zinc for treatment of diarrhea	16.9	8.6
Viet Nam	VNM	Oral antibiotics for pneumonia	81.1	41.1
Viet Nam	VNM	Vitamin A for treatment of measles	99.0	50.1
Viet Nam	VNM	ACTs- Artemisinin compounds for treatment of malaria	0.1	0.1
Viet Nam	VNM	SAM - treatment for severe acute malnutrition	0.2	0.1
Viet Nam	VNM	BCG vaccine	95.0	45.7
Viet Nam	VNM	Polio vaccine	90.0	43.3
Viet Nam	VNM	DPT vaccine	75.0	36.1
Viet Nam	VNM	H. influenzae type b vaccine	75.0	62.5
Viet Nam	VNM	HepB vaccine	75.0	36.1
Viet Nam	VNM	Measles vaccine	97.0	46.7
Viet Nam	VNM	Global wasting (<-2 SD) rate	4.2	6.3
Viet Nam	VNM	Contraceptive prevalence (CPR)	76.8	46.7
Yemen	YEM	Safe abortion services	40.0	24.3
Yemen	YEM	TT - Tetanus toxoid vaccination	70.0	33.7
Yemen	YEM	Syphilis detection and treatment	14.7	7.1
Yemen	YEM	Iron supplementation in pregnancy	5.5	2.6
Yemen	YEM	Hypertensive disorder case management	5.9	2.8
Yemen	YEM	Diabetes case management	4.6	2.2
Yemen	YEM	Malaria case management	19.1	9.2
Yemen	YEM	MgSO4 management of pre-eclampsia	11.7	5.6
Yemen	YEM	Thermal protection	30.3	15.3
Yemen	YEM	Clean cord care	29.3	14.8
Yemen	YEM	Clean birth environment	25.2	12.8
Yemen	YEM	Immediate drying and additional stimulation	28.1	14.2
Yemen	YEM	Neonatal resuscitation	16.9	8.6
Yemen	YEM	Antibiotics for preterm or prolonged PROM	22.9	11.6
Yemen	YEM	Parenteral administration of anti-convulsants	21.9	11.1
Yemen	YEM	Parenteral administration of uterotonics	27.4	13.9
Yemen	YEM	Parenteral administration of antibiotics	22.9	11.6
Yemen	YEM	Assisted vaginal delivery	7.8	3.9
Yemen	YEM	Manual removal of placenta	11.5	5.8
Yemen	YEM	Removal of retained products of conception	10.2	5.2
Yemen	YEM	Cesarean delivery	2.7	1.4
Yemen	YEM	Blood transfusion	3.9	2.0
Yemen	YEM	Induction of labor for pregnancies lasting 41+ weeks	0.5	0.3

Country	ISO 3166-1 alpha-3	Intervention Name	Baseline coverage (%)	Ending coverage (%)
Yemen	YEM	Complementary feeding - education only	26.6	15.4
Yemen	YEM	Complementary feeding - supplementary feeding and education	26.6	15.4
Yemen	YEM	Vitamin A supplementation	81.0	46.7
Yemen	YEM	Improved sanitation - Utilization of latrines or toilets	59.1	59.1
Yemen	YEM	Improved water source	63.5	63.5
Yemen	YEM	Water connection in the home	30.7	30.7
Yemen	YEM	Hand washing with soap	62.0	62.0
Yemen	YEM	Hygienic disposal of children's stools	18.6	18.6
Yemen	YEM	Injectable antibiotics for neonatal sepsis	30.7	14.8
Yemen	YEM	ORS - oral rehydration solution	25.3	12.8
Yemen	YEM	Antibiotics for treatment of dysentery	38.2	19.3
Yemen	YEM	Zinc for treatment of diarrhea	0.4	0.2
Yemen	YEM	Oral antibiotics for pneumonia	34.0	17.2
Yemen	YEM	Vitamin A for treatment of measles	81.0	41.0
Yemen	YEM	BCG vaccine	64.0	30.8
Yemen	YEM	Polio vaccine	59.0	28.4
Yemen	YEM	DPT vaccine	65.0	31.3
Yemen	YEM	H. influenzae type b vaccine	65.0	54.2
Yemen	YEM	HepB vaccine	65.0	31.3
Yemen	YEM	Pneumococcal vaccine	64.0	53.4
Yemen	YEM	Rotavirus vaccine	64.0	53.4
Yemen	YEM	Measles vaccine	65.0	31.3
Yemen	YEM	Global wasting (<-2 SD) rate	16.3	24.5
Yemen	YEM	Contraceptive prevalence (CPR)	41.4	25.2
Zambia	ZMB	Safe abortion services	3.3	2.0
Zambia	ZMB	TT - Tetanus toxoid vaccination	85.0	40.9
Zambia	ZMB	IPTp - Intermittent preventive treatment of malaria during pregnancy	81.3	39.1
Zambia	ZMB	Syphilis detection and treatment	23.6	11.4
Zambia	ZMB	Iron supplementation in pregnancy	59.1	28.4
Zambia	ZMB	Hypertensive disorder case management	13.0	6.3
Zambia	ZMB	Diabetes case management	10.1	4.9
Zambia	ZMB	Malaria case management	42.0	20.2
Zambia	ZMB	MgSO4 management of pre-eclampsia	25.7	12.4
Zambia	ZMB	Thermal protection	66.4	33.6
Zambia	ZMB	Clean cord care	64.1	32.5
Zambia	ZMB	Clean birth environment	55.1	27.9
Zambia	ZMB	Immediate drying and additional stimulation	61.5	31.1
Zambia	ZMB	Neonatal resuscitation	36.9	18.7
Zambia	ZMB	Antibiotics for preterm or prolonged PROM	50.2	25.4
Zambia	ZMB	Parenteral administration of anti-convulsants	48.0	24.3

Country	ISO 3166-1 alpha-3	Intervention Name	Baseline coverage (%)	Ending coverage (%)
Zambia	ZMB	Parenteral administration of uterotonics	60.0	30.4
Zambia	ZMB	Parenteral administration of antibiotics	50.2	25.4
Zambia	ZMB	Assisted vaginal delivery	17.0	8.6
Zambia	ZMB	Manual removal of placenta	25.1	12.7
Zambia	ZMB	Removal of retained products of conception	22.2	11.2
Zambia	ZMB	Cesarean delivery	5.8	2.9
Zambia	ZMB	Blood transfusion	8.5	4.3
Zambia	ZMB	Induction of labor for pregnancies lasting 41+ weeks	1.2	0.6
Zambia	ZMB	Complementary feeding - education only	22.0	12.7
Zambia	ZMB	Complementary feeding - supplementary feeding and education	22.0	12.7
Zambia	ZMB	Vitamin A supplementation	99.0	57.1
Zambia	ZMB	Improved sanitation - Utilization of latrines or toilets	26.4	26.4
Zambia	ZMB	Improved water source	60.0	60.0
Zambia	ZMB	Water connection in the home	19.1	19.1
Zambia	ZMB	Hand washing with soap	33.7	33.7
Zambia	ZMB	Hygienic disposal of children's stools	70.8	70.8
Zambia	ZMB	ITN/IRS - Households protected from malaria	84.0	48.5
Zambia	ZMB	Injectable antibiotics for neonatal sepsis	67.1	32.3
Zambia	ZMB	ORS - oral rehydration solution	52.5	26.6
Zambia	ZMB	Antibiotics for treatment of dysentery	37.8	19.1
Zambia	ZMB	Oral antibiotics for pneumonia	69.7	35.3
Zambia	ZMB	Vitamin A for treatment of measles	99.0	50.1
Zambia	ZMB	ACTs- Artemisinin compounds for treatment of malaria	18.4	9.3
Zambia	ZMB	BCG vaccine	91.0	43.8
Zambia	ZMB	Polio vaccine	90.0	43.3
Zambia	ZMB	DPT vaccine	90.0	43.3
Zambia	ZMB	H. influenzae type b vaccine	90.0	75.1
Zambia	ZMB	HepB vaccine	90.0	43.3
Zambia	ZMB	Pneumococcal vaccine	90.0	75.1
Zambia	ZMB	Rotavirus vaccine	91.0	75.9
Zambia	ZMB	Measles vaccine	96.0	46.2
Zambia	ZMB	Global wasting (<-2 SD) rate	6.3	9.5
Zambia	ZMB	Contraceptive prevalence (CPR)	55.3	33.6
Zimbabwe	ZWE	Safe abortion services	3.3	2.0
Zimbabwe	ZWE	TT - Tetanus toxoid vaccination	87.0	41.8
Zimbabwe	ZWE	IPTp - Intermittent preventive treatment of malaria during pregnancy	25.9	12.5
Zimbabwe	ZWE	Syphilis detection and treatment	80.5	38.7
Zimbabwe	ZWE	Iron supplementation in pregnancy	39.7	19.1
Zimbabwe	ZWE	Hypertensive disorder case management	28.7	13.8
Zimbabwe	ZWE	Diabetes case management	27.8	13.4

**Country**	**ISO 3166-1 alpha-3**	**Intervention Name**	**Baseline coverage (%)**	**Ending coverage (%)**
Zimbabwe	ZWE	Malaria case management	68.3	32.8
Zimbabwe	ZWE	MgSO4 management of pre-eclampsia	69.4	33.4
Zimbabwe	ZWE	Thermal protection	85.5	43.3
Zimbabwe	ZWE	Clean cord care	75.5	38.2
Zimbabwe	ZWE	Clean birth environment	67.6	34.2
Zimbabwe	ZWE	Immediate drying and additional stimulation	76.3	38.6
Zimbabwe	ZWE	Neonatal resuscitation	70.4	35.6
Zimbabwe	ZWE	Antibiotics for preterm or prolonged PROM	45.9	23.2
Zimbabwe	ZWE	Parenteral administration of anti-convulsants	85.3	43.2
Zimbabwe	ZWE	Parenteral administration of uterotonics	82.2	41.6
Zimbabwe	ZWE	Parenteral administration of antibiotics	45.9	23.2
Zimbabwe	ZWE	Assisted vaginal delivery	18.0	9.1
Zimbabwe	ZWE	Manual removal of placenta	8.5	4.3
Zimbabwe	ZWE	Removal of retained products of conception	16.8	8.5
Zimbabwe	ZWE	Cesarean delivery	0.5	0.3
Zimbabwe	ZWE	Blood transfusion	10.8	5.5
Zimbabwe	ZWE	Induction of labor for pregnancies lasting 41+ weeks	1.5	0.8
Zimbabwe	ZWE	Complementary feeding - education only	16.7	9.6
Zimbabwe	ZWE	Complementary feeding - supplementary feeding and education	16.7	9.6
Zimbabwe	ZWE	Vitamin A supplementation	43.0	24.8
Zimbabwe	ZWE	Improved sanitation - Utilization of latrines or toilets	36.2	36.2
Zimbabwe	ZWE	Improved water source	64.1	64.1
Zimbabwe	ZWE	Water connection in the home	19.9	19.9
Zimbabwe	ZWE	Hand washing with soap	64.2	64.2
Zimbabwe	ZWE	Hygienic disposal of children's stools	64.4	64.4
Zimbabwe	ZWE	ITN/IRS - Households protected from malaria	36.8	21.2
Zimbabwe	ZWE	Injectable antibiotics for neonatal sepsis	85.5	41.1
Zimbabwe	ZWE	ORS - oral rehydration solution	32.8	16.6
Zimbabwe	ZWE	Antibiotics for treatment of dysentery	8.0	4.1
Zimbabwe	ZWE	Zinc for treatment of diarrhea	22.5	11.4
Zimbabwe	ZWE	Oral antibiotics for pneumonia	50.9	25.8
Zimbabwe	ZWE	Vitamin A for treatment of measles	43.0	21.8
Zimbabwe	ZWE	ACTs- Artemisinin compounds for treatment of malaria	0.4	0.2
Zimbabwe	ZWE	SAM - treatment for severe acute malnutrition	8.9	4.5
Zimbabwe	ZWE	BCG vaccine	95.0	45.7
Zimbabwe	ZWE	Polio vaccine	89.0	42.8
Zimbabwe	ZWE	DPT vaccine	89.0	42.8
Zimbabwe	ZWE	H. influenzae type b vaccine	89.0	74.2
Zimbabwe	ZWE	HepB vaccine	89.0	42.8
Zimbabwe	ZWE	Pneumococcal vaccine	89.0	74.2

Country	ISO 3166-1 alpha-3	Intervention Name	Baseline coverage (%)	Ending coverage (%)
Zimbabwe	ZWE	Rotavirus vaccine	90.0	75.1
Zimbabwe	ZWE	Measles vaccine	90.0	43.3
Zimbabwe	ZWE	Global wasting (<-2 SD) rate	3.6	5.4
Zimbabwe	ZWE	Contraceptive prevalence (CPR)	68.3	41.5

Appendix C. Country-specific child mortality estimates for one month by scenario (including wasting)

INCREASE IN CHILD DEATHS BY COUNTRY IN A MONTH	SCENARIO 1	SCENARIO 2	SCENARIO 3
**Total for all modeled countries (n=118)**	**42,180**	**74,450**	**192,720**
Afghanistan	520	890	2,170
Albania	-	-	10
Algeria	260	470	1,310
Angola	490	830	1,950
Argentina	60	100	290
Armenia	-	10	20
Azerbaijan	20	40	90
Bangladesh	900	1,660	4,690
Belarus	-	-	10
Belize	-	-	-
Benin	260	440	1,050
Bhutan	-	10	20
Bolivia	40	60	170
Bosnia and Herzegovina	-	-	-
Botswana	20	30	90
Brazil	390	790	2,750
Burkina Faso	380	660	1,610
Burundi	210	360	860
Cambodia	110	190	530
Cameroon	310	530	1,290
Cape Verde	-	-	10
Central African Republic	90	150	350
Chad	370	670	1,620
Colombia	110	220	810
Comoros	10	20	60
Congo	70	120	290
Costa Rica	-	10	20
Côte d'Ivoire	530	890	2,110
Cuba	10	10	40
Dem. People's Republic of Korea	80	160	500
Democratic Republic of the Congo	2,280	3,820	9,010

INCREASE IN CHILD DEATHS BY COUNTRY IN A MONTH	SCENARIO 1	SCENARIO 2	SCENARIO 3
Djibouti	10	20	60
Dominican Republic	70	140	430
Ecuador	30	60	190
Egypt	410	750	2,070
El Salvador	20	30	100
Equatorial Guinea	20	30	80
Eritrea	40	60	150
Ethiopia	1,130	1,960	4,770
Gabon	20	40	100
Gambia	50	80	200
Georgia	-	10	10
Ghana	350	590	1,430
Guatemala	70	120	320
Guinea	260	450	1,070
Guinea-Bissau	30	50	120
Guyana	10	10	20
Haiti	70	120	290
Honduras	40	70	210
India	10,120	18,300	49,850
Indonesia	1,010	1,850	5,090
Iraq	220	370	950
Jamaica	10	10	40
Jordan	30	50	130
Kazakhstan	40	70	180
Kenya	450	790	2,120
Kyrgyzstan	30	50	120
Lao People's Democratic Republic	60	110	300
Lesotho	40	70	210
Liberia	130	230	570
Madagascar	320	570	1,480
Malawi	350	640	1,810
Maldives	-	-	-
Mali	590	1,020	2,490
Mauritania	80	140	330

INCREASE IN CHILD DEATHS BY COUNTRY IN A MONTH	SCENARIO 1	SCENARIO 2	SCENARIO 3
Mexico	290	540	1,660
Mongolia	10	20	50
Montenegro	-	-	-
Morocco	110	200	580
Mozambique	540	920	2,230
Myanmar	300	540	1,410
Namibia	30	50	150
Nepal	150	260	670
Nicaragua	20	50	150
Niger	550	980	2,400
Nigeria	6,650	11,720	28,820
Pakistan	3,870	6,530	15,960
Panama	10	20	60
Papua New Guinea	70	130	310
Paraguay	30	60	170
Peru	60	110	340
Philippines	530	930	2,410
Republic of Moldova	10	20	50
Republic of North Macedonia	-	-	10
Rwanda	120	210	570
Saint Lucia	-	-	-
Samoa	-	-	-
São Tomé and Príncipe	-	-	10
Senegal	190	320	800
Serbia	-	-	10
Sierra Leone	280	480	1,210
Solomon Islands	-	10	10
Somalia	380	700	1,720
South Africa	360	640	1,710
South Sudan	200	370	900
Sri Lanka	20	40	110
Sudan	470	820	1,960
Suriname	-	-	10
Swaziland	20	40	100

INCREASE IN CHILD DEATHS BY COUNTRY IN A MONTH	SCENARIO 1	SCENARIO 2	SCENARIO 3
Syrian Arab Republic	60	100	280
Tajikistan	90	150	370
Thailand	70	140	450
Timor-Leste	20	30	80
Togo	100	160	380
Tonga	-	-	-
Tunisia	30	50	140
Turkey	60	120	370
Turkmenistan	70	120	310
Uganda	640	1,080	2,630
Ukraine	30	50	130
United Republic of Tanzania	790	1,340	3,310
Uzbekistan	140	270	800
Vanuatu	-	-	10
Venezuela	120	220	690
Viet Nam	330	630	1,900
Yemen	250	440	1,100
Zambia	300	520	1,340
Zimbabwe	160	300	850

Appendix D. Country-specific maternal mortality estimates for one month by scenario

INCREASE IN MATERNAL DEATHS BY COUNTRY IN A MONTH	SCENARIO 1	SCENARIO 2	SCENARIO 3
**Total for all modeled countries (n=118)**	**2,002**	**3,563**	**9,410**
Afghanistan	40	68	160
Albania	0	0	0
Algeria	14	26	73
Angola	15	24	54
Argentina	4	8	21
Armenia	0	0	1
Azerbaijan	0	0	1
Bangladesh	25	46	127
Belarus	0	0	0
Belize	0	0	0
Benin	15	26	66
Bhutan	0	0	1
Bolivia	4	7	21
Bosnia and Herzegovina	0	0	0
Botswana	1	1	4
Brazil	26	55	195
Burkina Faso	17	30	74
Burundi	23	39	99
Cambodia	8	14	40
Cameroon	33	56	138
Cape Verde	0	0	0
Central African Republic	6	10	24
Chad	18	29	62
Colombia	13	27	100
Comoros	1	1	2
Congo	8	15	38
Costa Rica	0	0	1
Côte d'Ivoire	40	67	161
Cuba	1	1	4
Dem. People's Republic of Korea	8	20	66
Democratic Republic of the Congo	68	114	263
Djibouti	0	1	2

INCREASE IN MATERNAL DEATHS BY COUNTRY IN A MONTH	SCENARIO 1	SCENARIO 2	SCENARIO 3
Dominican Republic	3	6	21
Ecuador	3	6	20
Egypt	17	32	94
El Salvador	1	2	6
Equatorial Guinea	1	2	4
Eritrea	2	3	7
Ethiopia	48	84	193
Gabon	2	3	8
Gambia	5	9	22
Georgia	0	0	0
Ghana	23	40	100
Guatemala	5	9	26
Guinea	14	23	52
Guinea-Bissau	2	3	8
Guyana	0	1	1
Haiti	6	11	26
Honduras	2	5	17
India	458	848	2,398
Indonesia	75	140	390
Iraq	9	17	44
Jamaica	0	1	2
Jordan	1	1	4
Kazakhstan	1	2	4
Kenya	38	70	196
Kyrgyzstan	1	2	5
Lao People's Democratic Republic	3	5	13
Lesotho	4	7	23
Liberia	7	11	27
Madagascar	14	24	59
Malawi	37	71	218
Maldives	0	0	0
Mali	31	52	123
Mauritania	7	11	26
Mexico	11	22	66

INCREASE IN MATERNAL DEATHS BY COUNTRY IN A MONTH	SCENARIO 1	SCENARIO 2	SCENARIO 3
Mongolia	1	2	4
Montenegro	0	0	0
Morocco	4	8	24
Mozambique	19	32	78
Myanmar	16	29	77
Namibia	2	4	12
Nepal	9	16	44
Nicaragua	2	5	18
Niger	19	31	68
Nigeria	301	499	1,130
Pakistan	68	119	297
Panama	0	1	3
Papua New Guinea	2	4	11
Paraguay	3	6	19
Peru	10	21	70
Philippines	27	48	129
Republic of Moldova	0	0	1
Republic of North Macedonia	0	0	0
Rwanda	7	12	33
Saint Lucia	0	0	0
Samoa	0	0	0
São Tomé and Príncipe	0	0	1
Senegal	17	29	71
Serbia	0	0	1
Sierra Leone	35	60	148
Solomon Islands	0	0	1
Somalia	4	7	15
South Africa	17	32	92
South Sudan	4	6	13
Sri Lanka	2	4	11
Sudan	9	15	32
Suriname	0	0	1
Swaziland	2	3	9
Syrian Arab Republic	1	2	6

INCREASE IN MATERNAL DEATHS BY COUNTRY IN A MONTH	SCENARIO 1	SCENARIO 2	SCENARIO 3
Tajikistan	0	1	2
Thailand	5	12	40
Timor-Leste	0	0	1
Togo	6	10	23
Tonga	0	0	0
Tunisia	2	3	8
Turkey	3	6	19
Turkmenistan	0	0	1
Uganda	49	86	224
Ukraine	1	2	6
United Republic of Tanzania	74	129	329
Uzbekistan	1	2	7
Vanuatu	0	0	0
Venezuela	4	8	26
Viet Nam	13	26	88
Yemen	3	6	13
Zambia	11	20	56
Zimbabwe	26	49	146
